# Single-nucleus transcriptome-wide association study of human brain disorders

**DOI:** 10.1038/s41586-026-10836-6

**Published:** 2026-09-23

**Authors:** Sanan Venkatesh, Roman Kosoy, Zhenyi Wu, Marios Anyfantakis, Christian Dillard, Prashant N. M., David Burstein, Deepika Mathur, Chris Chatzinakos, Bukola Ajanaku, Fotis Tsetsos, Biao Zeng, Sonali Gupta, Rachel Bercovitch, Aram Hong, Clara Casey, Marcela Alvia, Zhiping Shao, Stathis Argyriou, Karen Therrien, PsychAD Consortium, Roman Kosoy, Christian Dillard, Prashant N. M., David Burstein, Deepika Mathur, Biao Zeng, Rachel Bercovitch, Aram Hong, Clara Casey, Marcela Alvia, Zhiping Shao, Stathis Argyriou, Karen Therrien, Athan Z. Li, Chenfeng He, Chirag Gupta, Christian Porras, Colleen A. McClung, Collin Spencer, Daifeng Wang, David A. Bennett, Fotios Tsetsos, Gennadi Ryan, Hui Yang, Jaroslav Bendl, Jennifer Monteiro Fortes, Jerome J. Choi, Kalpana H. Arachchilage, Lars J. Jensen, Lisa L. Barnes, Logan C. Dumitrescu, Lyra Sheu, Madeline R. Scott, Marios Anyfantakis, Maxim Signaevsky, Mikaela Koutrouli, Milos Pjanic, Monika Ahirwar, Nicolas Y. Masse, Noah Cohen Kalafut, Pavan K. Auluck, Pavel Katsel, Pengfei Dong, Pramod B. Chandrashekar, Sanan Venkatesh, Saniya Khullar, Sarah R. Murphy, Sayali A. Alatkar, Seon Kinrot, Steven Finkbeiner, Steven P. Kleopoulos, Tereza Clarence, Timothy J. Hohman, Ting Jin, Vahram Haroutunian, Vivek G. Ramaswamy, Xiang Huang, Xinyi Wang, Zhenyi Wu, Stefano Marenco, Kiran Girdhar, Donghoon Lee, John F. Fullard, Gabriel E. Hoffman, Georgios Voloudakis, Panos Roussos, Tim Bigdeli, Pavan Auluck, David A. Bennett, Stefano Marenco, Vahram Haroutunian, Kiran Girdhar, Jaroslav Bendl, Donghoon Lee, John F. Fullard, Gabriel E. Hoffman, Georgios Voloudakis, Panos Roussos

**Affiliations:** 1 Department of Psychiatry, Icahn School of Medicine at Mount Sinai, New York, NY USA; 2 Center for Disease Neurogenomics, Icahn School of Medicine at Mount Sinai, New York, NY USA; 3 Friedman Brain Institute, Icahn School of Medicine at Mount Sinai, New York, NY USA; 4 Department of Genetics and Genomic Science, Icahn School of Medicine at Mount Sinai, New York, NY USA; 5 Mental Illness Research, Education, and Clinical Center (VISN 2 South), James J. Peters VA Medical Center, Bronx, NY USA; 6 Center for Precision Medicine and Translational Therapeutics, James J. Peters VA Medical Center, Bronx, NY USA; 7 Department of Psychiatry and Behavioral Sciences, SUNY Downstate Health Sciences University, Brooklyn, NY USA; 8 Institute for Genomics in Health (IGH), SUNY Downstate Health Sciences University, Brooklyn, NY USA; 9 VA New York Harbor Healthcare System, Brooklyn, NY USA; 10 Department of Epidemiology and Biostatistics, School of Public Health, SUNY Downstate Health Sciences University, Brooklyn, NY USA; 11 Human Brain Collection Core, National Institute of Mental Health-Intramural Research Program, Bethesda, MD USA; 12 Rush Alzheimer’s Disease Center, Rush University Medical Center, Chicago, IL USA; 13 Department of Artificial Intelligence and Human Health, Icahn School of Medicine at Mount Sinai, New York, NY USA; 14 Department of Computer Sciences, University of Wisconsin-Madison, Madison, WI USA; 15 Waisman Center, University of Wisconsin-Madison, Madison, WI USA; 16 Department of Biostatistics and Medical Informatics, University of Wisconsin-Madison, Madison, WI USA; 17 Novo Nordisk Foundation Center for Protein Research, Faculty of Health and Medical Sciences, University of Copenhagen, Copenhagen, Denmark; 18 Department of Psychiatry, University of Pittsburgh School of Medicine, Pittsburgh, PA USA; 19 Taube/Koret Center for Neurodegenerative Disease Research, Gladstone Institutes, San Francisco, CA USA; 20 Department of Population Health Sciences, University of Wisconsin-Madison, Madison, WI USA; 21 Department of Neurological Sciences, Rush University Medical Center, Chicago, IL USA; 22 Vanderbilt Genetics Institute, Vanderbilt University Medical Center, Nashville, TN USA; 23 Vanderbilt Memory & Alzheimer’s Center, Vanderbilt University Medical Center, Nashville, TN USA; 24 Center for Systems and Therapeutics, Gladstone Institutes, San Francisco, CA USA; 25 Department of Neurology, University of California San Francisco, San Francisco, CA USA; 26 Department of Physiology, University of California San Francisco, San Francisco, CA USA; 27 Neuroscience and Biomedical Sciences Graduate Programs, University of California San Francisco, San Francisco, CA USA; 28 Department of Neuroscience, Icahn School of Medicine at Mount Sinai, New York, NY USA

**Keywords:** Gene expression, Data integration, Computational models, Quantitative trait loci

## Abstract

Common brain disorders impose a substantial health burden, but localizing their genetic risk in the brain remains challenging^1^. Although genome-wide association studies have identified numerous loci associated with neuropsychiatric and neurodegenerative disorders, many of these loci lie in non-coding regions that influence gene expression in specific cell types^2–5^. Traditional bulk brain transcriptomic analyses, which often focus on European ancestry cohorts, average over cellular diversity, obscuring genetic risk-related changes in gene expression. Here we use single-nucleus gene expression profiles from the dorsolateral prefrontal cortex in the multi-ancestry PsychAD cohort to develop transcriptomic imputation models of genetically regulated expression across major brain cell types. Applying these models to neuropsychiatric and neurodegenerative disorders reveals thousands of gene–trait associations that are undetectable in bulk tissue analyses and resolves many signals to discrete neuronal, glial and immune cell populations. Cross-ancestry analyses in the Million Veteran Program confirm these associations, reveal pleiotropic effects of cell-type-specific predicted expression and demonstrate that trait-related dysregulation is conserved across ancestries, enabling mapping of causal genes and pathways. Together, these findings provide a cell-type-resolved and ancestry-aware atlas of genetically regulated expression in the human prefrontal cortex and illustrate how single-nucleus transcriptomics can sharpen gene discovery and therapeutic target prioritization for complex brain disorders.

## Abstract (web summary)

Single-nucleus transcriptomics across diverse populations is used to explore how genetic risk for brain disorders affects specific cell types, revealing previously hidden gene–trait associations and conserved, cell-type-specific mechanisms.

## Main

The genetic architecture of neuropsychiatric and neurodegenerative disorders (NPDs and NDDs, respectively) is highly polygenic, with much of their heritability attributed to common variants in non-coding regions that influence gene regulation rather than protein sequence^2–5^ (Supplementary Tables 1 and 2). Genome-wide association studies (GWAS) have identified thousands of risk variants for these disorders, yet mapping these loci to effector genes and mechanisms in the human brain remains challenging. Transcriptome-wide association studies (TWAS) integrate GWAS summary statistics with predictive models of genetically regulated gene expression (GReX) to implicate genes whose expression changes may drive disease susceptibility^6–9^. However, most brain TWAS reference panels are derived from homogenate bulk cortex RNA sequencing (RNA-seq) in predominantly European (EUR) ancestry cohorts^6–8,10^, which averages over diverse neuronal and non-neuronal populations and captures only part of the genetic regulation operating in the brain. Given the extensive cellular, transcriptional^11,12^ and regulatory^13^ heterogeneity of the human prefrontal cortex, approaches that treat it as a uniform tissue are poorly suited for resolving cell-type-specific contributions to NPD^2,14^ and NDD^5^ risk.

The PsychAD Consortium^11,15^ generated a population-scale single-nucleus RNA-seq (snRNA-seq) atlas of the dorsolateral prefrontal cortex (DLPFC), comprising over 6 million nuclei from 1,494 donors with and without major neuropsychiatric diagnoses and spanning EUR, African (AFR) and admixed American (AMR) ancestries. Leveraging this data, we constructed single-nucleus transcriptomic imputation models (snTIMs) across multiple cellular populations, enabling cell-type-resolved estimation of GReX across ancestries. We then applied snTIMs to 12 NPD and NDD GWAS to perform single-nucleus TWAS (snTWAS) and identify cell-type-specific gene–trait associations (GTAs), including previously unreported disease-linked loci. Finally, we validated and extended these findings in a large-scale phenome-wide association study (PheWAS) of approximately 600,000 participants in the Million Veteran Program (MVP). Using ancestry-matched snTIMs, we compared effect patterns across populations, characterized the pleiotropy of cell-type-specific GReX across neurological and mental health diagnoses, and refined causal gene prioritization.

## snTIMs yield ancestry and cell-type-specific GReX

**Extended Data Fig. 1:**
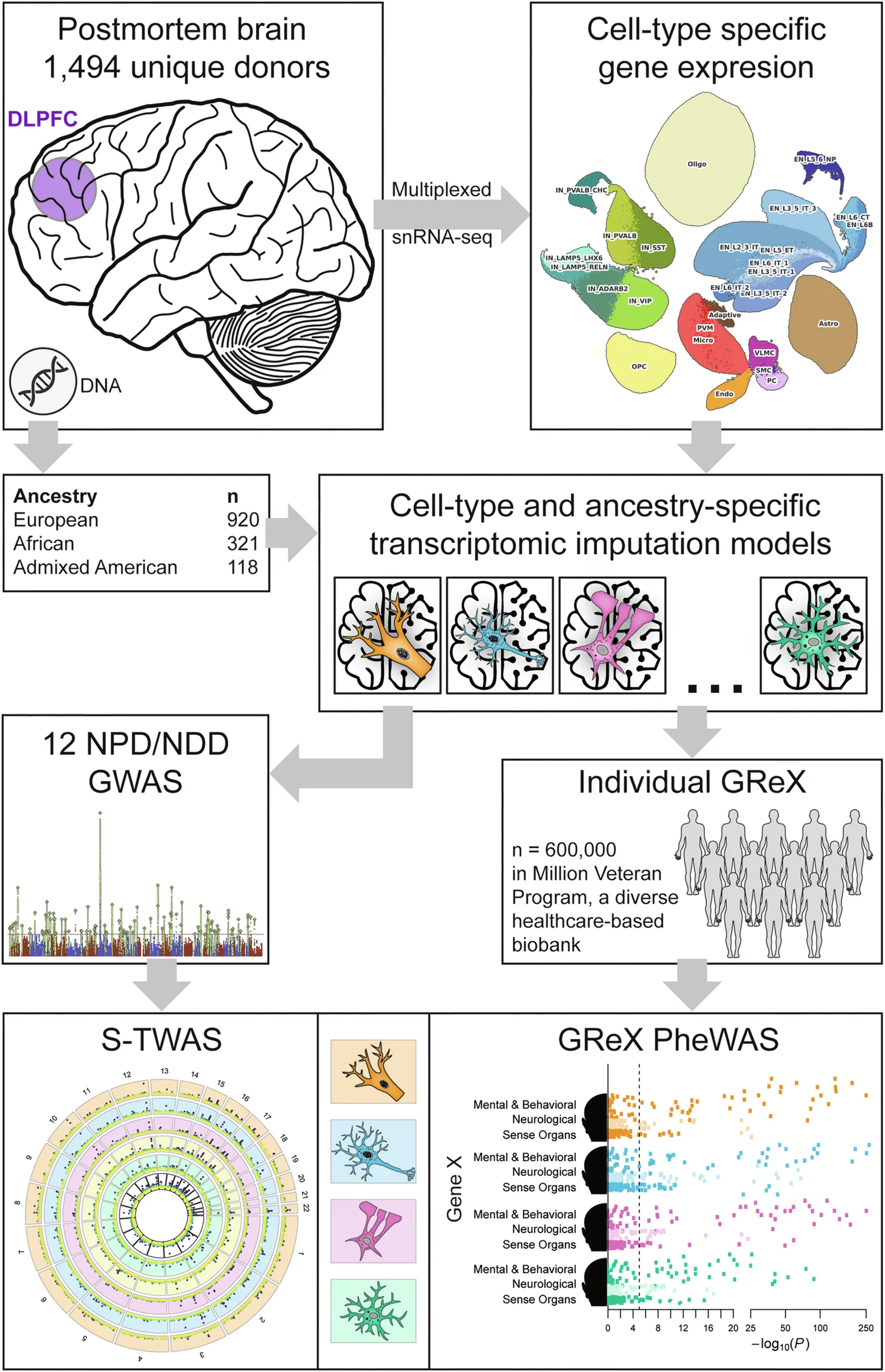
Overview of study design. Schematic of the overall analysis workflow, from postmortem dorsolateral prefrontal cortex sampling and single-nucleus RNA sequencing (snRNA-seq) in PsychAD, through construction of ancestry- and cell-type-specific single-nucleus transcriptomic imputation models (snTIMs), to summary-level snTWAS across 12 brain disorder genome-wide association studies (GWAS) and individual-level single-nucleus genetically regulated gene expression phenome-wide association studies (snGReX-PheWAS) in the Million Veteran Program (MVP).

**Extended Data Fig. 2:**
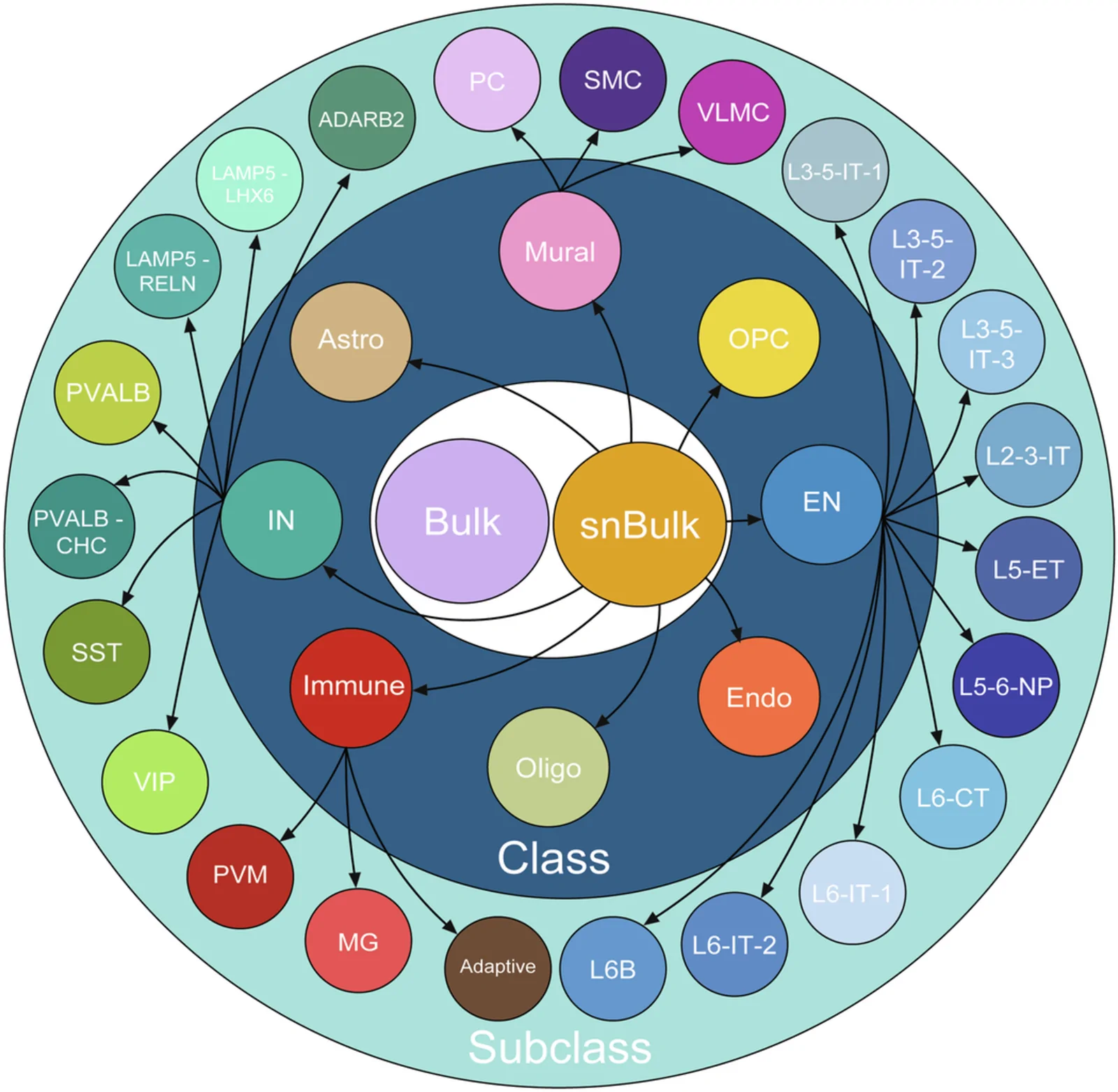
PsychAD cell-type hierarchy. Schematic of the dorsolateral prefrontal cortex (DLPFC) cell-type hierarchy. Cell-types are organized into homogenate (Bulk/snBulk), Class, and Subclass levels. Single-nucleus transcriptomic imputation models (snTIMs) based on single-nucleus RNA-seq (snRNA-seq) of the PsychAD cohort are split into 3 levels depending on level of resolution (snBulk, Class and Subclass) whereas a previously trained TIM for the same region using homogenate RNA-seq is limited to the homogenate level (Bulk). Class-OPC, Astro, Endo, and Oligo are not further subdivided at the subclass level and are therefore included in both class and subclass aggregate analyses. This results in 27 non-overlapping cellular populations across 32 cellular populations in the PsychAD cohort. Full names of all cell-types are defined in Supplementary Table 3.

We developed an analytical framework to investigate how brain cell-type-specific GReX contributes to NPDs and NDDs (Extended Data Fig. 1). Using quality-controlled (see Methods) genotype and snRNA-seq data from the PsychAD Consortium^11,15^, we trained 94 snTIMs across three ancestries and 32 cellular populations in the DLPFC (Extended Data Fig. 2 and Supplementary Table 3). For downstream TWAS, we restricted the analyses to snTIMs that showed robust cross-validated performance within each ancestry (see Methods). Limiting to protein-coding genes, we obtained reliable imputation models for 12,289 (74.1%), 10,375 (62.6%) and 5,543 (33.4%) of the 16,594 autosomal genes assayed in PsychAD^11,15^ for EUR (*N* = 920), AFR (*N* = 321) and AMR (*N* = 118) ancestry, respectively (Supplementary Tables 4–9 and Supplementary Fig. 1).

**Fig. 1:**
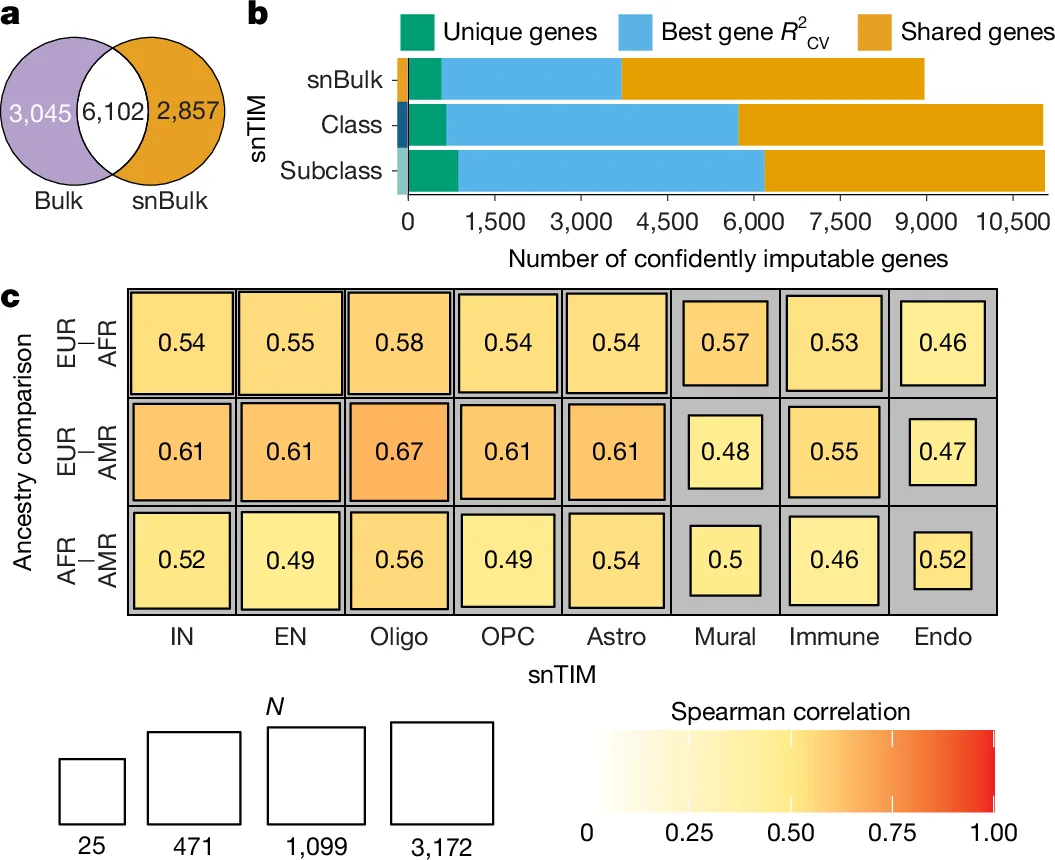
snTIM performance across cell types and ancestries. **a**, Venn diagram showing counts of confidently imputable genes unique to bulk (DLPFC homogenate) or snBulk (DLPFC pooled nuclei from snRNA-seq) and shared between them. **b**, Gene imputability across different cell-type resolutions. The bar lengths represent the number of reliably imputable genes. For each category, genes are split into three groups: uniquely captured genes (unique genes), best predicted genes (best gene *R*^2^_CV_; SNP predictors explain a higher percentage of the gene expression variance in this category) and shared genes (imputable genes that are better captured in other categories). Class and subclass utilize the best gene model (max *R*^2^_CV_) among all participating snTIMs for that level. **c**, Heatmap of cross-ancestry Spearman’s correlation coefficients (*ρ*) of *R*^2^_CV_ at the class level. For each cell type, Spearman’s correlation is assessed only among genes confidently imputed in the snTIMs of both ancestries. *N* indicates the number of shared confidently imputable genes in both ancestries. Full correlation statistics are described in Supplementary Table 9. Full names of all cell types are defined in Supplementary Table 3. Astro, astrocyte; EN, excitatory neuron; Endo, endothelial cell; IN, inhibitory neuron; Oligo, oligodendrocyte; OPC, oligodendrocyte precursor cell. Source data

The EUR single-nucleus-derived pseudobulk TIM (‘snBulk’; all nuclei pooled) and a similarly sized EUR homogenate-based TIM from the same region (‘bulk’; DLPFC EpiXcan TIM^16^; *N* = 924; 311 individuals in common with EUR snBulk) imputed a comparable number of genes (9,147 versus 8,959) with substantial but incomplete overlap (6,102 shared; 66.7–68.1%; Fig. 1a and Supplementary Note 1). Within the snTIM hierarchy, increasing the cell-type resolution from snBulk to cellular classes (‘class’) and subclasses (‘subclass’) expanded the confidently imputable set by 3,330, whereas totals remained similar between class and subclass (11,021 and 11,052, respectively; Fig. 1b, Supplementary Table S10 and Supplementary Note 2). When selecting the highest performing TIM per gene at each resolution, class and subclass snTIMs more frequently achieved the top cross-validated *R*^2^ (*R*^2^_CV_) than snBulk (Fig. 1b), indicating that finer cellular resolution improves model performance (Supplementary Fig. 2a). However, the presence of genes uniquely or best imputed in snBulk indicates that certain genes require larger effective sample sizes for reliable prediction.

To validate snTIM performance out of sample, we used two independent genotype–gene expression datasets: snRNA-seq from the Religious Orders Study/Memory and Aging Project (ROSMAP)^17^ EUR cohort and bulk RNA-seq data from fluorescence-activated cell sorting-isolated microglia (FACS-MG)^18,19^. In both datasets, cross-validated and out-of-sample *R*^2^ showed strong agreement, supporting the reproducible regulation of gene expression across cohorts (Supplementary Note 3).

We next compared snTIMs across ancestries. Despite differences in training sample size, shared gene–cell-type models showed strong correlations in *R*^2^_CV_ across ancestries (EUR–AMR: *ρ* = 0.58, two-sided *P* = 3.45 × 10^−1,158^, *N* = 13,054; EUR–AFR: *ρ* = 0.54, two-sided *P* = 4.22 × 10^−2,711^, *N* = 36,070; and AFR–AMR: *ρ* = 0.49, two-sided *P* = 1.29 × 10^−633^, *N* = 10,637; Spearman; Fig. 1c, Supplementary Table 11 and Supplementary Figs. 2b, 3 and 4), indicating that the extent to which gene expression is under genetic regulation is broadly consistent across ancestries, even when the underlying single-nucleotide polymorphisms (SNPs), which serve as gene expression predictors, differ (Supplementary Notes 4 and 5).

Together, these results show that snTIMs substantially expand the set of imputable genes compared with bulk tissue TIMs, enhance predictive performance across diverse cellular populations and capture conserved genetic regulation of brain gene expression across ancestries.

## Brain snTWAS identifies known and novel GTAs

**Fig. 2:**
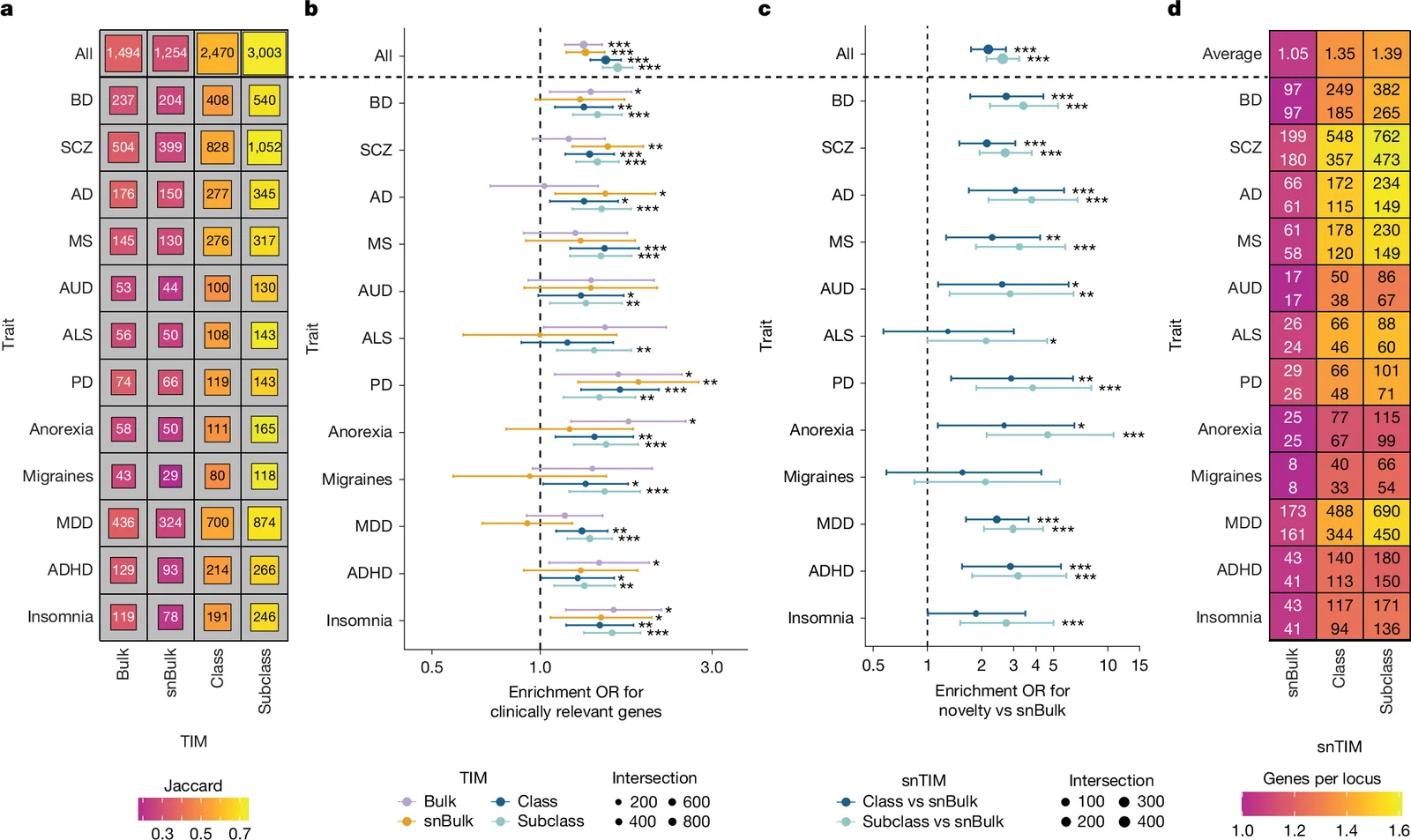
Discovery power of S-snTWAS in individuals of EUR ancestry. **a**, Heatmap of significant GTAs by TIM resolution (bulk, snBulk, class and subclass). The numbers in cells and square size represent significant GTA counts (logistic regression analysis; FDR-adjusted two-sided *P* ≤ 0.05; FDR adjustment is performed across all traits and TIMs for parity). ‘All’ refers to the union of all traits at the TIM level. The cell colour denotes the trait-specific Jaccard index, defined as the fraction of significant GTAs at that level (*x*) that overlap the union of significant GTAs across all levels for the same trait ($\frac{{|x}\cap {all}|}{{|x}\cup {all}|}$). Full names of the disorders are described in Supplementary Table 12. **b**, Enrichment OR (Fisher’s exact test) of GTAs for clinically relevant gene sets comprising genes known to be associated with central nervous system-related neurological and behavioural or psychiatric symptoms (see Methods) across different cellular resolution levels. Intersection indicates the size of the intersection between clinically relevant genes and GTAs. The asterisks indicate significance (*FDR-adjusted two-sided *P* ≤ 0.05, **FDR-adjusted two-sided *P* ≤ 0.01 and ***FDR-adjusted two-sided *P* ≤ 0.001). ‘All’ refers to the enrichment of the gene sets for each TIM across all traits. The error bars reflect the 95% confidence interval. The vertical dashed line indicates OR = 1. Accompanying statistics, including sample sizes used to derive confidence intervals (column ‘intersection’) and exact *P* value (column ‘*P* value’) are provided in Supplementary Table 13. **c**, Forest plot demonstrating preferential identification of ‘novel’ versus known GTAs in class and subclass versus snBulk. Novel genes are defined as genes that are not identified in bulk TWAS or MAGMA analyses. OR represents the enrichment (Fisher’s exact test) for novel genes at each level (across all classes or across all subclasses) versus snBulk. To identify non-novel genes for the ‘All’ analysis, we took the union of bulk TWAS significant genes (FDR-adjusted two-sided *P* ≤ 0.05) and MAGMA significant genes (FDR-adjusted two-sided *P* ≤ 0.05) across all traits, then tested enrichment of class or subclass significant genes (FDR-adjusted two-sided *P* ≤ 0.05; union across all traits) against snBulk significant genes (FDR-adjusted two-sided *P* ≤ 0.05; union across all traits). Intersection indicates the size of the intersection between novel genes and GTAs. The asterisks indicate significance (*FDR ≤ 0.05, **FDR ≤ 0.01 and ***FDR ≤ 0.001). The error bars reflect the 95% confidence interval. The vertical dashed line indicates OR = 1. Accompanying statistics, including sample sizes used to derive confidence intervals (column ‘FinerCT Novel’) and exact *P* value (column ‘*P* value’) are provided in Supplementary Table 17. **d**, Fine-mapping of S-snTWAS signals. In each heatmap cell, the top and bottom numbers give the count of fine-mapped genes and loci (PIP ≥ 0.5), respectively (after FDR adjustment). The colour indicates the number of genes per locus. Fine-mapping was performed with FOCUS for each cell type. The row ‘Average’ indicates the average genes per locus across the 12 traits. The horizontal dashed line separates the summary category (‘All’ or ‘Average’) across panels. ALS, amyotrophic lateral sclerosis; MS, multiple sclerosis. Source data

To characterize cell-type-specific GReX dysregulation in NPDs and NDDs, we applied EUR bulk and snTIMs to perform summary-level TWAS (S-TWAS) on 12 EUR GWAS (Supplementary Table 12 and external data 1 (ref. ^20^), while controlling the false discovery rate (FDR)^21^ to account for the varying number of gene–cell-type tests at different cellular resolutions. Across traits, bulk and snBulk yielded comparable numbers of significant GTAs (1,494 versus 1,254). Among genes imputable by both TIMs, 882 were significant in snBulk and 963 in bulk, with 529 overlapping (Jaccard = 0.40). At higher levels of cellular resolution, the subclass snTIMs identified the most unique significant associations (3,003 genes across all traits), followed by the class (2,470) and snBulk (1,254) snTIMs. This progression underscores the enhanced ability of finer cellular resolution to uncover GTAs (Fig. 2a and Supplementary Fig. 5).

To determine whether the greater number of GTAs identified by cell-type-specific TWAS is clinically relevant, we performed gene set enrichment analysis for genes known to be associated with central nervous system-related neurological and behavioural or psychiatric symptoms in the Online Mendelian Inheritance in Man database^22^. Bulk and snBulk analyses revealed significant enrichment for genes linked to 5 and 4 of the 12 NPDs and NDDs, respectively, whereas class and subclass captured 11 and all 12 traits, respectively (Fig. 2b and Supplementary Table 13). This stepwise increase supports the improved biological and clinical relevance of TWAS at higher cellular resolution.

We next assessed whether higher cellular resolution preferentially identified ‘novel’ versus ‘known’ GTAs. Genes were classified as novel if the GTA was not identified by bulk S-TWAS (Supplementary Table 14; homogenate DLPFC TWAS analysis of the same GWAS) or by multi-marker analysis of genomic annotation (MAGMA) gene-based analysis^23,24^ (Supplementary Table 15), which detects genes through common variant aggregation of GWAS summary statistics alone. Across all traits and snTIMs, 22.5% of significant GTAs were novel (3,711 of 16,345; Supplementary Table 16 and external data 1 (ref. ^20^). Subclass snTIMs were more likely to detect novel than known GTAs (Fig. 2c; significant enrichment in 11 of 12 traits; Fisher’s exact test FDR-adjusted two-sided *P* ≤ 0.05), particularly within subclass excitatory neurons layer 6B (L6B) for bipolar disorder (BD), and subclass inhibitory neurons vasoactive intestinal peptide-expressing interneurons for anorexia nervosa (Supplementary Table 17 and Supplementary Fig. 6). These results indicate that bulk-level analyses miss a substantial fraction of cell-type-specific disease signals that are uncovered by snTWAS.

Because multiple GTAs can occur within the same locus due to linkage disequilibrium^10,25^, we next performed probabilistic fine-mapping using fine-mapping of causal gene sets (FOCUS)^25^ to identify putatively causal genes. On average, 60.8% of significant GTAs (9,936 of 16,345) were retained after fine-mapping (Fig. 2d and Supplementary Table 18), with most loci containing one gene with posterior inclusion probability (PIP) ≥ 0.5, indicating a 50% or more likelihood of being causal (Supplementary Fig. 7). Increasing cell-type resolution raised the ratio of fine-mapped genes to loci (Supplementary Note 6). This observation indicates that distinct cellular populations may contribute uniquely within shared loci, warranting further functional studies. However, lower cell-type resolution snTIMs may benefit from increased statistical power, which could contribute to improved fine-mapping precision.

## S-TWAS identifies cell-type-specific disease signatures

**Fig. 3:**
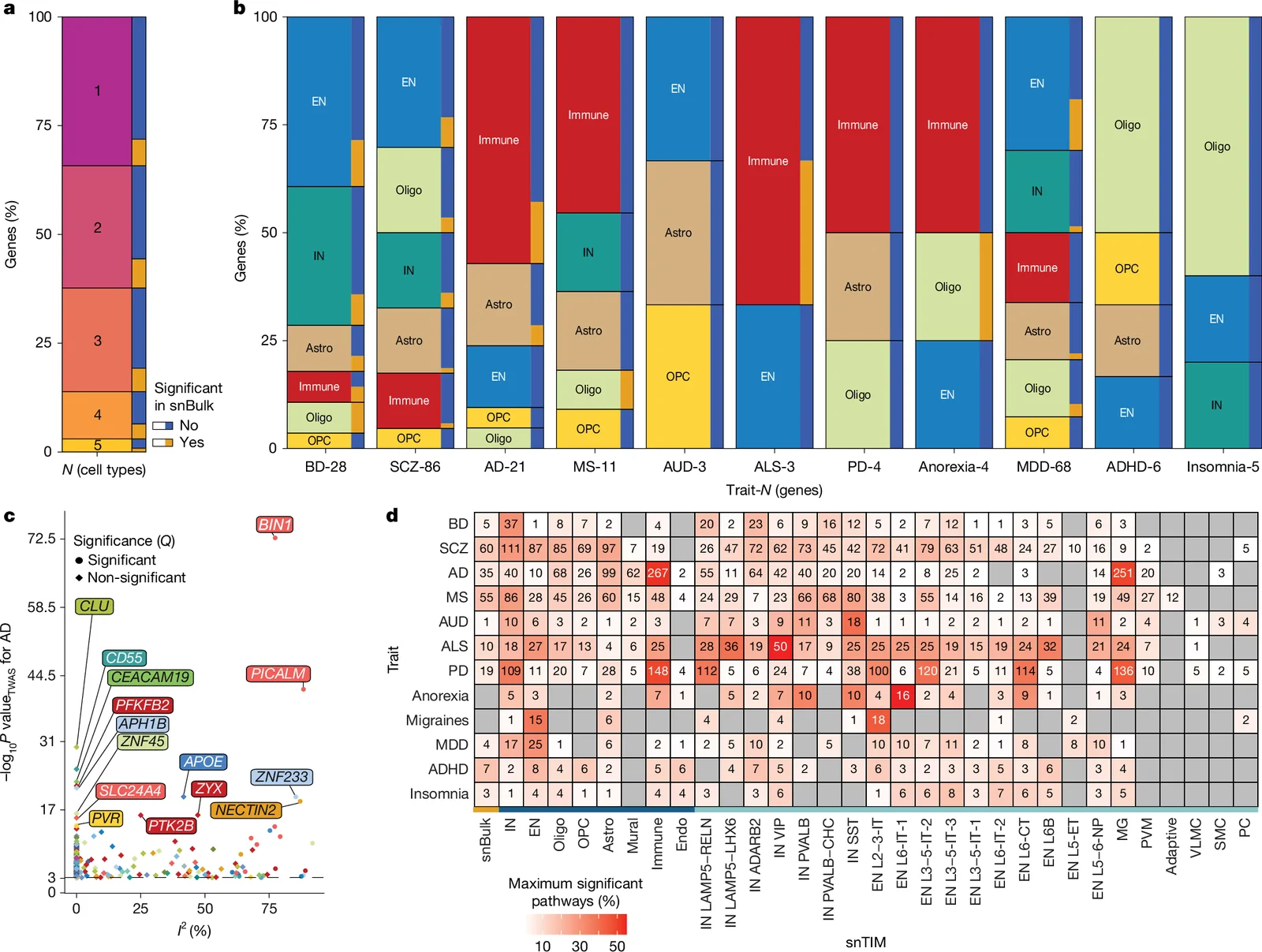
Cell-type specificity in snTWAS. **a**, mash was applied to the S-snTWAS results to jointly test GReX effects at the class level for all traits. Relative frequency stacked bar charts visualize the percentage of genes with significant post-mash GTAs in one, two, three, four and five cell types (left) and their respective breakdown into GTAs either significant (orange) or not (blue) in snBulk S-snTWAS (right). Endo and mural classes were excluded from the mash analysis due to high sparsity in comparison with other class-level snTIMs (INs, ENs, oligocyte, OPCs, astrocyte and immune). Only S-snTWAS GTAs that were mash significant (local false sign rate ≤ 0.05) in at least one out of six cell types and not mash significant in all six cell types are visualized. **b**, Relative frequency stacked bar charts showing the breakdown of post-mash cell-type-specific GTAs (GTAs detected in only one cell type) from panel **a**. The number next to each trait indicates the total number of post-mash cell-type-specific GTAs found per trait across the six analysed cell types. Cell types are ordered by their frequency in trait-specific mash GTAs. The full names of disorders are described in Supplementary Table 12. The full names of all cell types are defined in Supplementary Table 3. **c**, Gene association effect size heterogeneity (*I*^2^) across cell types for AD. Significance denotes the presence of different effects of individual cell types in the analysis (Cochran’s *Q*-test). Only genes with a significant (logistic regression analysis; FDR-adjusted two-sided *P* ≤ 0.05) GTA were visualized. The horizontal dashed line denotes this significance threshold. The colour corresponds to cell type (Extended Data Fig. 2). Points with significant heterogeneity (Cochran’s *Q*-test FDR-adjusted two-sided *P* ≤ 0.05; circles) are doubled in size for better visualization. **d**, Linkage disequilibrium-aware snTWAS competitive pathway enrichment. The number in each cell represents the number of FDR-significant pathways (corrected across all snTIMs) after hierarchical pruning (see Methods). The colour represents the proportion of all significant pathways identified in each cellular population for each trait. CT, corticothalamic; ET, extratelencephalic; IT, intratelencephalic; NP, near projecting; PC, pericyte; PVM, perivascular macrophage; SMC, smooth muscle cell; VLMC, vascular leptomeningeal cell. Source data

**Extended Data Fig. 3:**
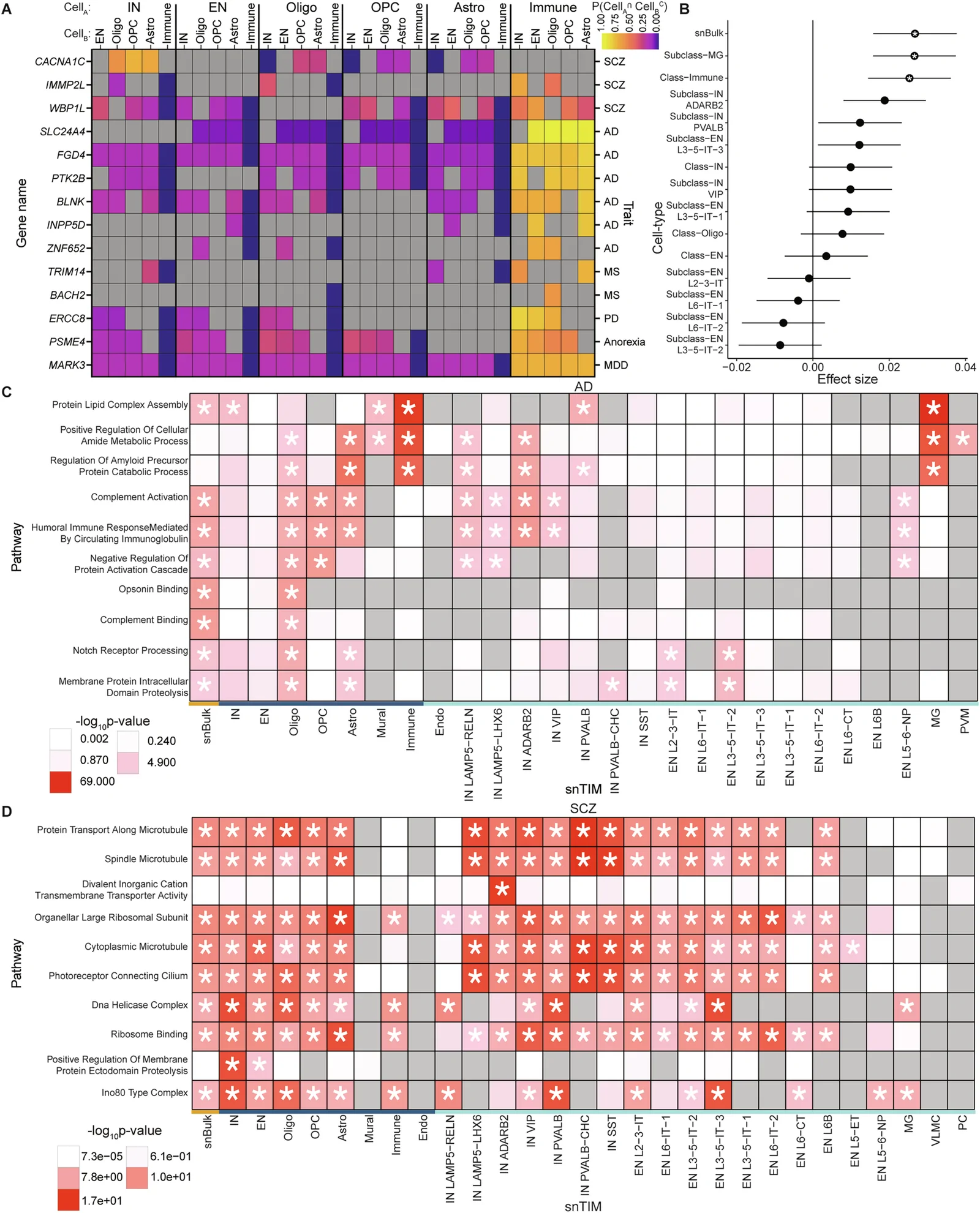
Cell-type-specific genes and pathways in single-nucleus transcriptome-wide association studies (snTWAS). **A**, Heatmap of multivariate adaptive shrinkage (mash) combinatorial posterior probabilities that a gene-trait association (GTA) is present in class-level cell-type A and absent in cell-type B ($\mbox{``} P({\mathrm{Cell}}_{A}\cap {\mathrm{Cell}}_{B}^{C})\mbox{''}$). Only gene-trait pairs with $P({\mathrm{Cell}}_{A}\cap {\mathrm{Cell}}_{B}^{C})\ge 0.6$ in at least one comparison (16 gene-trait combinations) are shown; gray cells indicate combinations without an applicable estimate. Full names of disorders are described in Supplementary Table 12. Full names of all cell-types are defined in Supplementary Table 3. **B**, Forest plot of *BIN1* association effect sizes for Alzheimer’s disease (AD) across cell-types in Million Veteran Program (MVP) individual-level snTWAS (I-snTWAS). The circle represents the effect size, the horizontal bars represent the 95% confidence intervals, and asterisks mark false discovery rate (FDR)-significant associations. Analysis is performed on 37,038 AD cases and 401,544 controls (Supplementary Table 12). **C**, Top 10 hierarchically pruned pathways enriched for AD snTWAS signals across cell-types, prioritized by Correlation Adjusted MEan RAnk (CAMERA) two-sided p-value; asterisks indicate FDR-adjusted two-sided p-value ≤ 0.05, and at most two significant pathways per cell-type are displayed to increase cell-type-specific representation. **D**, Analogous pathway enrichment results for schizophrenia (SCZ), showing the top 10 hierarchically pruned pathways across cell-types with the same plotting conventions as in panel C. Statistical test, p-value correction, etc. are performed identically to panel C. Source data

To formally quantify the degree of cell-type specificity of GTAs within each trait, we used multivariate adaptive shrinkage (mash^26^) to estimate posterior probabilities of cell-type-specific effects. Across all traits, 20.9% of GTAs (701 of 3,348) showed evidence of cell-type-specific effects (Supplementary Table 19), and 34.2% of these appeared in a single cell type (Fig. 3a). At the class level, class-immune exhibited the greatest degree of specificity across multiple traits (Fig. 3b, Extended Data Fig. 3a, Supplementary Table 20 and Supplementary Fig. 8). Among the top hits, *CACNA1C* showed highly specific effects in class-inhibitory neurons for schizophrenia (SCZ; Extended Data Fig. 3a, Supplementary Table 20 and Supplementary Fig. 8), consistent with its established functional role in SCZ pathophysiology^27^.

Existing S-TWAS methods^8,28^ have yielded well-calibrated association *z*-scores that closely recapitulate results from individual-level TWAS results (Supplementary Table 21 and Supplementary Fig. 9a). By contrast, their effect size estimates are less reliable because they depend on reference panel quantities rather than the GWAS cohort, specifically the SNP linkage disequilibrium structure used to scale the variance of predicted expression and the allele frequencies and SNP coverage used for harmonization, and they do not propagate uncertainty in the prediction weights^28^ (Supplementary Table 21 and Supplementary Fig. 9b,c). As a result, summary-based effect sizes are not appropriate for quantifying cell-type heterogeneity, and aggregation approaches that rely on *z*-scores or *P* values conflate true effect magnitude with sampling precision and power^29,30^. To overcome this limitation, we performed individual-level snTWAS (I-snTWAS) by imputing GReX across MVP EUR. This strategy allowed us to derive NPD-specific and NDD-specific heterogeneity scores for each gene–trait pair across all imputable cell types, using the *I*^2^ statistic^30^ as a proxy (ranging from 0% to 100% for minimal to very high heterogeneity, respectively; Supplementary Table 22). In Alzheimer’s disease (AD), the top gene, *BIN1*, showed marked heterogeneity (*I*^2^_cell type_ = 77.36%; 95% confidence interval 78.22−91.87%; 15 imputable cell types; Fig. 3c, Supplementary Table 22 and Supplementary Fig. 10), reflecting large variation in effect size across cell types (Extended Data Fig. 3b), largely driven by cell-type-specific single-nucleus expression quantitative trait loci^12,31^ (Supplementary Figs. 11 and 12). By contrast, other AD genes, including *CLU*, showed highly consistent effect sizes across cell types (*I*^2^_cell type_ = 0%; 95% confidence interval 0−0%; 11 imputable cell types; Fig. 3c).

We next applied a linkage disequilibrium-aware competitive pathway enrichment method^32^ to identify biological pathways perturbed in the summary-level snTWAS (S-snTWAS) analyses and to characterize their cell-type specificity. As cellular resolution increased, the number of significantly enriched pathways also increased (Fig. 3d, Supplementary Fig. 13 and external data 2 (ref. ^20^). This pattern was particularly pronounced for traits such as AD and alcohol use disorder (AUD), where snTWAS signals in MG and inhibitory neurons, respectively, provided the most biologically relevant insights. For example, in AD, one of the top pathways was ‘tau protein binding’ (GO:0048156), primarily driven by dysregulation in *BIN1* and *APOE* in class-immune (FDR-adjusted two-sided *P* = 1.36 × 10^−60^) and subclass-MG (FDR-adjusted two-sided *P* = 2.79 × 10^−64^; Extended Data Fig. 3c). Shared AD pathways across cell types were often driven by different genes. ‘Positive regulation of neuron death category’ (GO:1901216) was primarily driven by *PICALM* and *APOE* in class-immune (FDR-adjusted two-sided *P* = 8.00 × 10^−36^) and subclass-MG (FDR-adjusted two-sided *P* = 4.49 × 10^−33^), whereas *CLU* additionally contributed in other cellular populations (classes: astrocytes and oligodendrocytes; subclasses: inhibitory neuron adenosine deaminase RNA-specific B2 expressing (ADARB2)). Similarly, the ‘regulation of amyloid precursor protein catabolic process’ (GO:1902991) was driven by *PICALM*, *APOE* and *SORL1* in class-immune (FDR-adjusted two-sided *P* = 9.73 × 10^−42^) and subclass-MG (FDR-adjusted two-sided *P* = 7.78 × 10^−40^), with *ABCA7* further contributing in subclass-inhibitory neuron parvalbumin expressing (FDR-adjusted two-sided *P* = 5.83 × 10^−3^). In SCZ, which showed widespread genetically driven biological pathway dysregulation across many cellular populations (Fig. 3b), ‘divalent inorganic cation transmembrane transporter activity’ (GO:0072509) emerged as one of the strongest pathways, driven primarily by *CACNA1C* and *GPM6A* and restricted to subclass-inhibitory neuron ADARB2 (FDR-adjusted two-sided *P* = 1.41 × 10^−13^; Extended Data Fig. 3d), providing a putative cellular context for a known clinical biomarker of SCZ^33^. Of note, most of these key genes were also fine-mapped in the relevant cell types (Supplementary Table 18; PIP ≥ 0.5), supporting their probable causal role.

**Extended Data Fig. 4:**
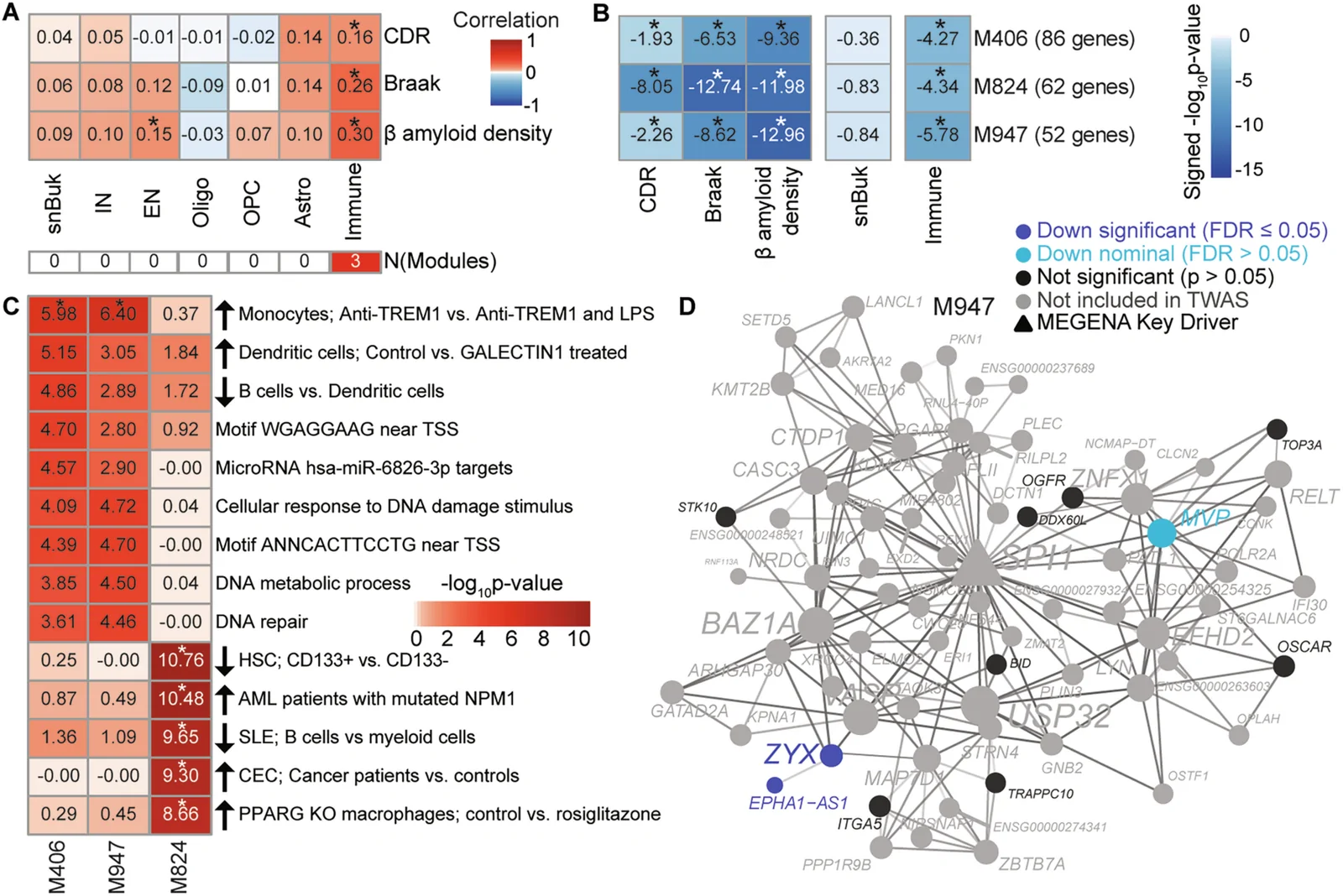
Relationship of Alzheimer’s disease (AD) summary-level single-nucleus transcriptome-wide association study (S-snTWAS) genes to AD-associated changes in Microglia. **A**, Relationship between AD S-snTWAS z-scores and Multiscale Embedded Gene Co-expression Network Analysis (MEGENA) co-expression modules derived from freshly isolated primary human microglia. The top heatmap shows Pearson’s correlations between module enrichment scores for AD S-snTWAS z-scores (x-axis) and module enrichment scores for AD-related neuropathology measures (Clinical Dementia Rating (CDR), Braak stage, and beta-amyloid plaque density; y-axis) across all 306 modules containing at least 50 genes. Asterisks mark false discovery rate (FDR)-adjusted two-sided p-value ≤ 0.05. The bottom heatmap shows, for each cellular population (snBulk and class-level analyses), the number of MEGENA modules significantly enriched (FDR-adjusted two-sided p-value ≤ 0.05) for the corresponding S-snTWAS signature. **B**, Heatmap for the Correlation Adjusted MEan RAnk (CAMERA) enrichment of neuropathology and S-snTWAS signatures in the 3 FDR significant modules from panel A (M406, M824, M947). Asterisks indicate FDR-adjusted two-sided p-value ≤ 0.05, and white text is used in selected cells to improve legibility. The color corresponds to the -log_10_(two-sided p-value) of CAMERA analyses multiplied by the sign of the observed enrichment, with negative values reflecting the enrichment of the modules for genes negatively associated with the indicated measures. **C**, Heatmap of top pathways enriched in the 3 FDR-significant MEGENA modules. Asterisks indicate fisher’s exact test FDR-adjusted two-sided p-value ≤ 0.05. Pathways were renamed for greater clarity of the underlying biology. Upward arrows denote upregulation and downward arrows denote downregulation in the differentially expressed gene signatures derived from the Molecular Signatures Database (MSigDB) C4 Immune gene set collection. Aliases for each pathway are listed in Supplementary Table 24. **D**, MEGENA co-expression network for module M947 showing gene-gene connectivity; node color encodes AD S-snTWAS significance and node shape denotes MEGENA key driver genes. Source data

Although pathway-level analyses revealed broad cell-type-specific perturbations, we next examined whether S-snTWAS recapitulate and extend known associations with brain pathology, using AD as a case study and focusing on MG contributions. We leveraged a hierarchical co-expression network constructed from freshly isolated human MG spanning healthy and neurodegenerative ageing^19^ to test MG co-expression modules for competitive enrichment of AD S-snTWAS signals (see Methods). Class-immune AD associations showed the strongest correspondence with AD pathology-linked differential expressed gene signatures^19^ (Extended Data Fig. 4a), a pattern not present in other cell types. Three modules (FDR-adjusted two-sided *P* ≤ 0.05) were significantly enriched for both AD-related differential expression and β-amyloid plaque density, and all exhibited negative enrichment (reduced predicted or observed expression in AD; Extended Data Fig. 4b and Supplementary Table 23). Functional annotation of these modules highlighted immune and myeloid activation programs, including module M824, which mapped to myeloid gene expression, and a parent–child pair (M406 → M947) pointing to *TREM1* dysregulation (Extended Data Fig. 4c and Supplementary Table 24). Both M406 and M947 contained *ZYX* (zyxin), which our S-snTWAS predicted to be downregulated in AD (class-immune: FDR-adjusted two-sided *P* = 9.18 × 10^−14^, *z*-score = −8.29; subclass-MG: FDR-adjusted two-sided *P* = 1.61 × 10^−13^, *z*-score = −8.21) and fine-mapped with essentially complete posterior support (FOCUS PIP = 1.0 and 1.0; Supplementary Table 18). Of note, *ZYX* did not emerge in differential expression analyses (Extended Data Fig. 4d). The same modules also contained *EPHA1-AS1*, recently linked to AD^19^, which lies at the same GWAS locus as *ZYX*, underscoring the genetic and functional complexity of this region. Together, these findings prioritize *ZYX* as a putative coding effector at this locus and link its downregulation to impaired MG stress responses to β-amyloid^34^.

## Cell-type specificity reveals shared GTAs

**Fig. 4:**
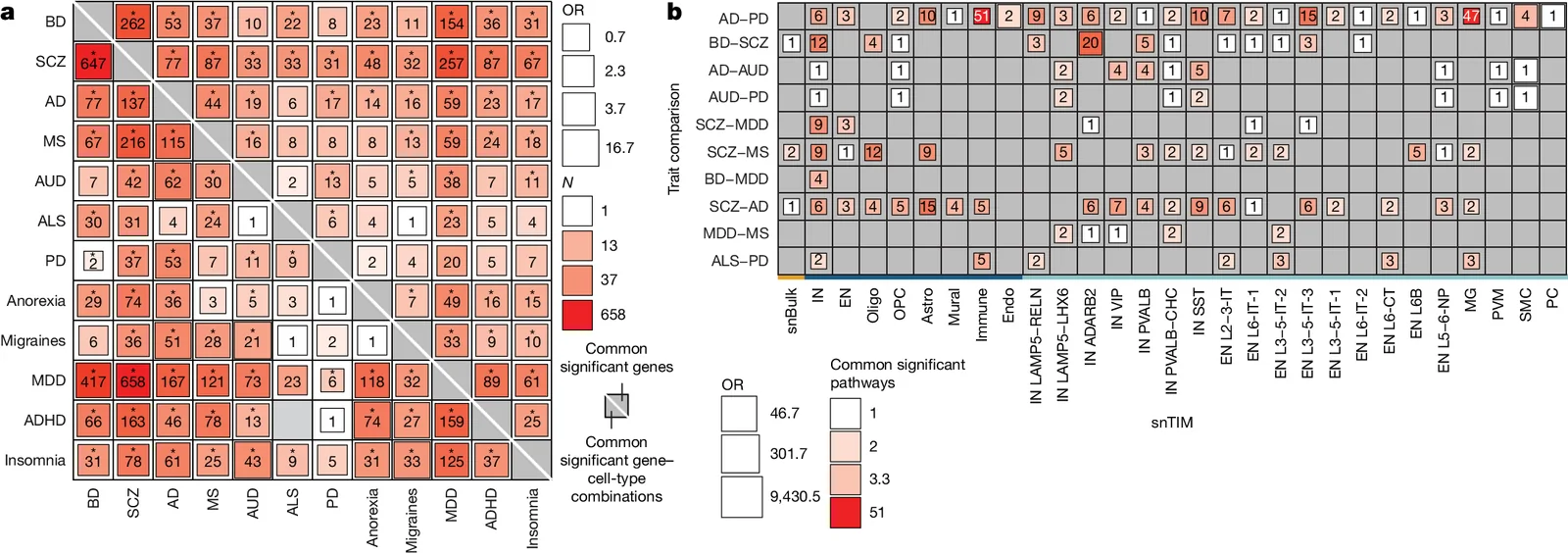
snTWAS uncovers shared genetic effects on gene expression dysregulation across disorders. **a**, Heatmap of cross-disorder sharing of significant associations for genes. The number and colour in each cell indicate either the number of shared significant gene–cell-type combinations (bottom-left triangle; genes can appear multiple times across cell types for a disorder pair) or the number of shared significant genes regardless of cell-type origin (top-right triangle) between each pair of disorders. Square size indicates the OR from Fisher’s exact test. Fisher’s exact test two-sided *P* values were FDR corrected, and significant values are annotated by asterisks. The full names of disorders are described in Supplementary Table 12. **b**, Cross-disorder pathway sharing. All trait pairs (*y* axis) with shared pathways are visualized in this heatmap across all snTIMs (*x* axis). The number and colour in each cell indicate the number of pathways significant in both traits (FDR-adjusted two-sided *P* ≤ 0.05) after hierarchical pruning (see Methods), and square size denotes the strength of enrichment for pathway sharing between the two traits within the cell type (Fisher’s exact test). The full names of all cell types are defined in Supplementary Table 3. Source data

Given the high comorbidity^35,36^ and genetic correlation^1^ among NPDs and NDDs, we next asked how cell-type-specific GReX dysregulation contributes to shared biology across traits. For each pair of traits, we tested the overlap of significant gene–cell-type associations using Fisher’s exact tests and found significant enrichment (FDR-adjusted two-sided *P* ≤ 0.05) in approximately 76% of all pairs (50 of 66; Fig. 4a and Supplementary Table 25). Comparable results were obtained when considering shared significant genes irrespective of cell type (Fig. 4a and Supplementary Table 26), indicating that these enrichments are not simply driven by cell-type redundancy. To minimize potential inflation due to overlapping samples across GWAS, we compared S-snTWAS with MVP I-snTWAS for traits with no documented sample overlap with the discovery GWAS. The direction of effects was highly consistent (90% sign concordance; 5,198 of 5,758 gene–cell-type–trait combinations; Supplementary Fig. 14). Moreover, genes shared among strongly genetically correlated disorders tend to have increasing concordance of effects at more stringent significance thresholds (Supplementary Figs. 15 and16), as illustrated in the progressive thresholding correlation analysis (PTCA; see Methods; Supplementary Fig. 17).

Extending the analysis to substance use disorders further confirmed that genome-wide correlation patterns in S-snTWAS mirror those observed in GWAS (Supplementary Note 7), grouping disorders into their expected categories of NPD, NDD and substance related. Pathway-level analyses yielded a similar pattern: approximately 47.0% of trait pairs (31 of 66) shared at least one significantly enriched pathway–cell-type combinations (Supplementary Table 27 and Supplementary Fig. 18), with similar results when pathways were aggregated across all cell types (Supplementary Table 28 and Supplementary Fig. 18). Concordance analyses (Supplementary Fig. 16) and PTCA (Supplementary Fig. 17) likewise supported, on average, shared directionality of pathway dysregulation across related traits.

Cell-type resolution greatly enhanced the detection of cross-disorder convergence (Fig. 4b and Supplementary Table 29). For example, cross-disorder comparison of AD with Parkinson’s disease (PD) revealed the highest number of shared pathways in class-immune and subclass-MG (Fig. 4b), consistent with shared inflammatory mechanisms. Similarly, BD–SCZ and SCZ–major depressive disorder (MDD) comparisons showed their strongest convergence within subclass-inhibitory neuron ADARB2 and class-inhibitory neuron, respectively (Fig. 4b and Supplementary Table 29). These cell-type-specific patterns were not apparent in snBulk analyses (Fig. 4b) despite high global (for example, for SCZ–BD–MDD^1^) or local (for example, for AD–PD^37^) genetic correlation.

Together, these findings show that integrating cell-type-specific snTIMs into TWAS sharpens cross-disorder analyses, resolving convergent molecular pathways that are largely masked in bulk-level analyses.

## Ancestry-specific snTWAS uncovers shared biology

**Fig. 5:**
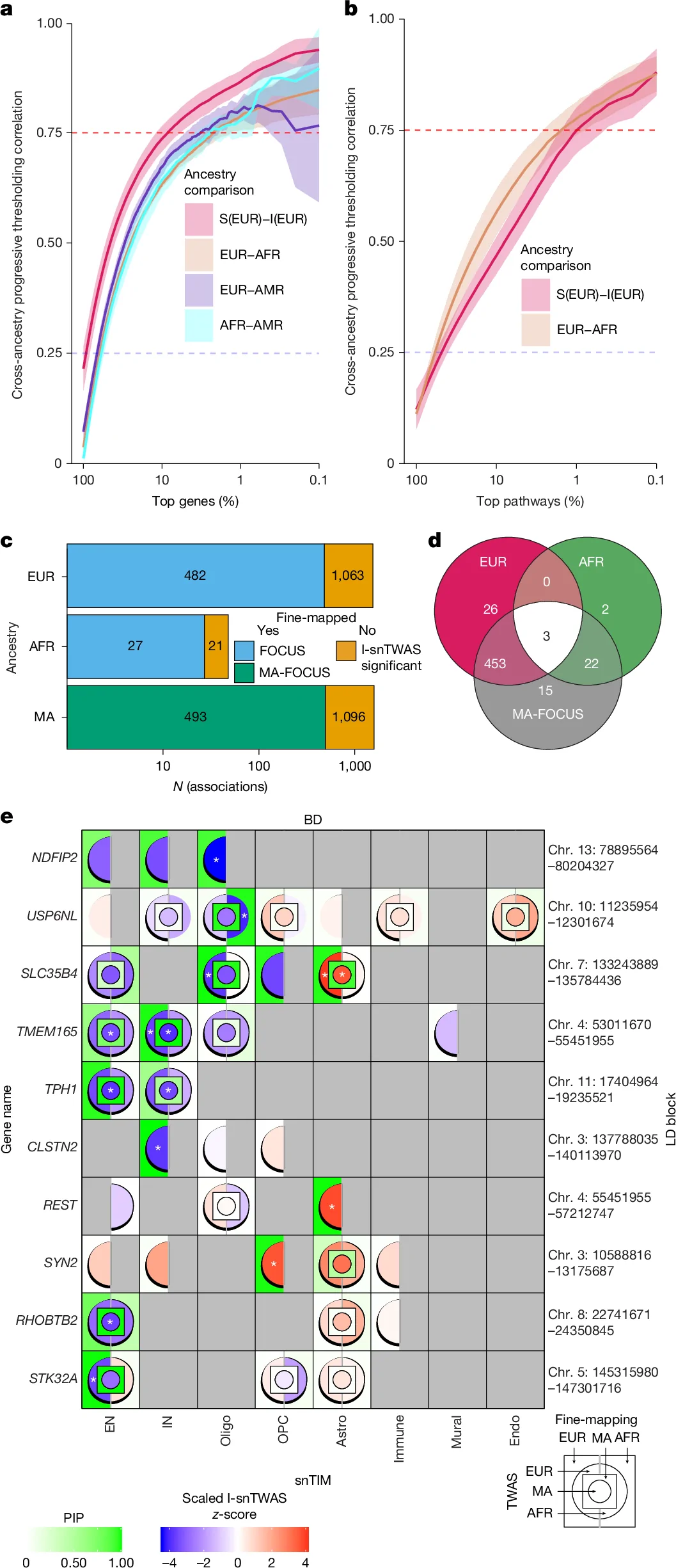
Cross-ancestry snTWAS identify similar GTAs across ancestries. **a**, Cross-ancestry PTCA. The line represents the mean correlation between the two comparison groups among the six traits intersecting between the I-snTWAS analysis and the S-snTWAS analysis (excluding MDD due to sample overlap). The shaded area represents the 95% confidence interval. The red and purple horizontal dashed lines denote Pearson’s correlation coefficients (*r*) of 0.75 and 0.25, respectively. ‘S(EUR)–I(EUR)’ corresponds to the comparison of S-snTWAS in individuals of EUR against EUR I-snTWAS. EUR, AFR and AMR correspond to their respective I-snTWAS in individuals of European, African and admixed American ancestry, respectively. **b**, Cross-ancestry I-snTWAS pathway-level PTCA. Each line tracks the cross-ancestry Pearson’s correlation of association *z*-scores for progressively higher-ranked pathway–cell-type combinations. The shaded area represents the 95% confidence interval. The red and purple horizontal dashed lines denote Pearson’s *r* of 0.75 and 0.25, respectively. **c**, Overlap of TWAS and fine-mapping in single-ancestry and multi-ancestry settings across the nine I-snTWAS traits. The annotated numbers in the bar plot indicate the total number of associations in each category (for example, there are 482 fine-mapped (PIP ≥ 0.5) trait–gene–cell-type combinations in EUR and 1,063 I-snTWAS significant trait–gene–cell-type combinations that are not fine-mapped, summing to a total of 1,545 I-snTWAS significant trait–gene–cell-type combinations). The *y* axis indicates the ancestry in which analysis was performed. The *x* axis indicates the number of fine-mapped and significant associations and is log_10_ scaled. ‘MA’ indicates the MA-FOCUS bi-ancestry analysis and the union of EUR and AFR I-snTWAS significant trait–gene–cell-type combinations for fine-mapping and TWAS, respectively. **d**, Overlap of fine-mapped (PIP ≥ 0.5) trait–gene–cell-type combinations (FOCUS for EUR and AFR; MA-FOCUS for bi-ancestry EUR and AFR). **e**, Bi-ancestry (EUR and AFR) fine-mapping of BD. The top ten BD-associated genes across EUR and AFR I-snTWAS analyses are visualized across class-level snTIMs. The *z*-scores are scaled across all genes within each ancestry. Asterisks indicate FDR-adjusted two-sided *P* ≤ 0.05 in EUR, AFR, or meta-analysed I-snTWAS. All data for this panel are available in Supplementary Table 30. The full names of all cell types are defined in Supplementary Table 3. LD, linkage disequilibrium. Source data

Previous work has highlighted poor portability of TIMs across ancestries as a major limitation of multi-ancestry TWAS^38,39^. To enable large-scale cross-ancestry interrogation of GReX dysregulation in NPDs and NDDs, we performed I-snTWAS in MVP using ancestry-matched individuals and snTIMs for eight disorders (fewer traits than in S-snTWAS due to sample size constraints and overlap between GWAS discovery samples and MVP). PTCA showed strong correlation of top-ranked GTAs across ancestries, comparable with the within-ancestry agreement between EUR S-snTWAS and EUR I-snTWAS (Fig. 5a and Supplementary Table 12). Pathway-level results from EUR and AFR I-snTWAS were likewise highly concordant (Fig. 5b). Despite the smaller effective sample size of MVP relative to the discovery GWASs (Supplementary Table 12), we identified significant GTAs in all ancestries (Supplementary Fig. 19 and external data 3 (ref. ^20^). Given the comparatively small and heterogeneous MVP AMR sample (*N* = 61,073; 9.3% of the total)^40^, which nevertheless showed good concordance in targeted replication^6^, we restricted in-depth I-snTWAS analyses to EUR and AFR to ensure adequate power for large-scale GReX mapping.

The high I-snTWAS PTCA concordance across ancestries (Fig. 5a) indicates broadly conserved GReX dysregulation in NPDs and NDDs, yet only 8.3% of significant AFR GTAs (4 of 48) were also significant in EUR, even when aggregating across all cell types. Because replication power is limited for many of our traits in I-snTWAS, we formally evaluated the benefit of a multi-ancestry design by comparing ancestry-specific versus bi-ancestry TWAS fine-mapping. The overall fraction of significant I-snTWAS associations that fine-mapped was similar (EUR-only FOCUS: 482 of 1,545 = 31.2%; multi-ancestry FOCUS (MA-FOCUS): 493 of 1,589 = 31.0%; PIP ≥ 0.5), but the bi-ancestry fine-mapping recovered many AFR-only fine-mapped associations that were missed in the EUR-only analysis (25 versus 3; Fig. 5c,d). These patterns were consistent when collapsing to GTAs rather than trait–cell-type–gene combinations (Supplementary Fig. 20a,b), and the distribution of bi-ancestry PIPs resembled both EUR and AFR within their respective significant associations (Supplementary Fig. 20c).

We next asked whether bi-ancestry fine-mapping better prioritizes disease-relevant genes. In excitatory neurons, *RHOBTB2* was not FDR significant in either EUR or AFR BD I-snTWAS (EUR *z*-score = −3.27, log(odds ratio (OR)) = −0.021, FDR-adjusted two-sided *P* = 0.21, effective *N* = 50,649.34; AFR *z*-score = −2.71, log(OR) = −0.032, FDR-adjusted two-sided *P* = 0.39, effective *N* = 14,164.83), yet the bi-ancestry inverse-variance meta-analysis was significant (*z*-score = −4.16, log(OR) = −0.023, FDR-adjusted two-sided *P* = 0.02; Fig. 5e, Supplementary Table 30 and Supplementary Fig. 21). Fine-mapping support increased from moderate within ancestries (FOCUS PIP_EUR_ = 0.78; PIP_AFR_ = 0.40) to near-certain in MA-FOCUS (PIP_MA_ = 0.98), prioritizing *RHOBTB2* as the probable causal BD gene at this locus. The biological plausibility of *RHOBTB2* is supported by functional studies showing that broad-complex, Tramtrack and Bric-à-brac (BTB)-domain variants increase excitability in human induced pluripotent stem cell-derived neurons and that altering the *Drosophila* sodium channel orthologue paralytic modifies RhoBTB-driven seizure-like phenotypes in vivo^41^. The improvement in fine-mapping parallels cross-ancestry shrinkage of credible SNP sets in multi-ancestry BD GWAS^42^, although to a lesser extent, consistent with the much smaller number of candidate genes versus SNPs per linkage disequilibrium block and the higher cross-ancestry concordance at the GReX level than at the SNP-effect level^43^. Collectively, these results show that multi-ancestry snTWAS fine-mapping improves both cell-type-specific gene discovery and causal gene prioritization across ancestries.

## NPD/NDD-associated GReX has pleiotropic effects

**Fig. 6:**
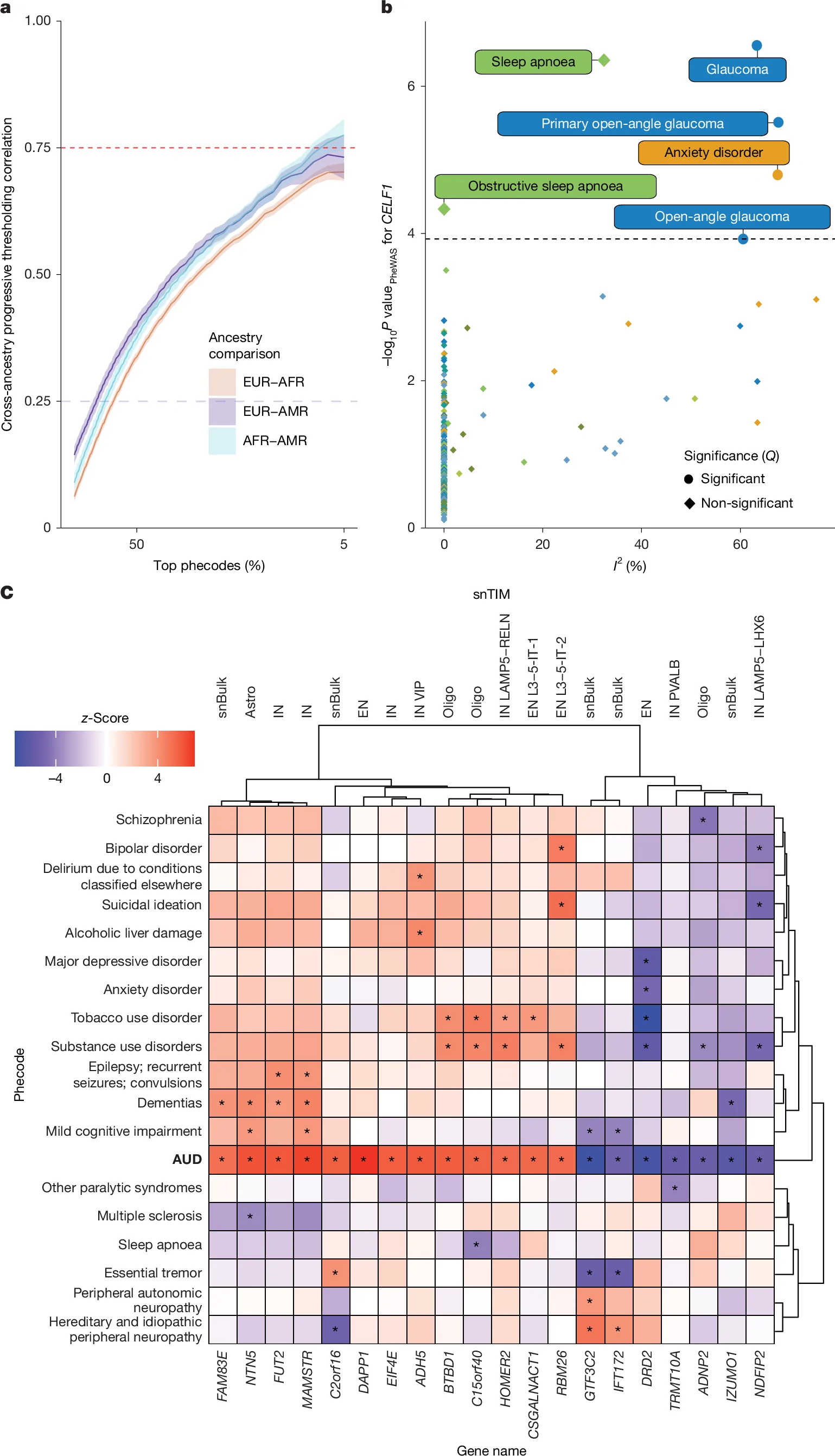
snGReX–PheWAS. **a**, Conservation of cross-ancestry correlations of snGReX–PheWAS associations among top trait–gene–cell-type combinations. We selected significant genes from our S-snTWAS and I-snTWAS (FDR-adjusted two-sided *P* ≤ 0.05), and performed PheWAS on the top associations (Bonferroni-adjusted two-sided *P* ≤ 0.05 across all confidently imputable associations within each cell type; 623 genes) to maximize analytical yield while remaining within computational constraints. For each pairwise ancestry combination, phecodes were ranked by inverse-variance weighted meta-analysis utilizing *z*-scores normalized in each ancestry to adjust for power differences across ancestries; only phecodes with at least 500 cases and 500 controls were considered. The resulting snGReX–PheWAS was then leveraged for three PTCAs corresponding to each ancestry pairwise combination among their shared gene–cell-type combinations (*N* = 1,757, 880 and 681 in individuals of EUR–AFR, EUR–AMR and AFR–AMR, respectively). Consequently, the pairwise Pearson’s correlation coefficient is visualized among decreasing numbers of top phecodes. The line represents the mean correlation between the two ancestries indicated. The shaded regions around each line represent the 95% confidence interval. The red and purple horizontal dashed lines denote Pearson’s *r* of 0.75 and 0.25, respectively. **b**, *CELF1* cell-type-specific effect size heterogeneity across phecodes. Each phecode is represented by the cell type with the lowest association *P* value indicated by its corresponding colour (Extended Data Fig. 2). To determine the significance of effect size heterogeneity (Cochran’s *Q*-test), we only considered cell types with significant associations (logistic regression FDR-adjusted two-sided *P* ≤ 0.05). The horizontal dashed line corresponds to the −log_10_(two-sided *P* value) of the weakest significant (FDR-adjusted two-sided *P* ≤ 0.05) association. Points with significant PheWAS Cochran’s *Q*-test *P* value (FDR-adjusted two-sided *P* ≤ 0.05) are double the size for better visualization. **c**, Clustering of the top relevant PheWAS associations in AUD. We selected all gene–cell-type combinations significant in the AUD S-snTWAS and I-snTWAS (logistic regression analysis; FDR-adjusted two-sided *P* ≤ 0.05). Per gene, we selected the top cell type (based on two-sided *P* value) associated with phecode 317.1 (mapping to AUD; Supplementary Table 10) to avoid clustering simply due to homogeneity of GReX across cell types. These 20 gene–cell-type combinations were significantly (FDR-adjusted two-sided *P* ≤ 0.05) associated with 18 other phecodes (among phecode categories ‘mental disorders’ and ‘neurological’ for better interpretation of disorders associated with AUD). Signficant Ward’s hierarchical agglomerative clustering was performed with Ward’s criterion preserved. Finally, we highlight the AUD association in the heatmap in bold. Asterisks indicate FDR-adjusted two-sided *P* ≤ 0.05 for the relevant combination. The full names of all cell types are defined in Supplementary Table 3. Source data

To investigate cell-type-specific pleiotropy of GReX, we performed a single-nucleus GReX phenome-wide association study (snGReX-PheWAS) in MVP for the top 623 significant snTWAS genes across all snTIMs (external data 4 (ref. ^20^). We first assessed phenome-wide conservation of GReX dysregulation across ancestries and observed strong cross-ancestry agreement by PTCA (Fig. 6a) and by association effect size sign concordance (Supplementary Fig. 22). These findings replicate and extend the cross-ancestry patterns seen in our targeted I-snTWAS analyses. We then restricted the phenome scan to neurological, mental and behavioural, and sensory organ disorders within EUR ancestry (‘focused’ PheWAS) to characterize pleiotropic effects in greater detail.

In earlier I-snTWAS analyses, we showed that effect size heterogeneity across cell types, quantified by *I*^2^_cell type_, varies by gene within a given trait (for example, AD; Fig. 3c). Using the multi-cell-type snGReX-PheWAS, we demonstrated that for a given gene (for example, *CELF1*), *I*^2^_cell type_ also varies across phenotypes (Fig. 6b; Supplementary Table 31 lists all significant associations for the genes included in the PheWAS). *CELF1*, which has no significant GTAs in snBulk, shows a wide range of *I*^2^_cell-type_ values across the relevant phenome where the GTAs are driven by different cellular populations (Fig. 6b and Supplementary Fig. 23). This complexity illustrates how cell-type-specific expression dysregulation shapes pleiotropic genetic effects that remain largely hidden in bulk-level analyses.

Overall, we detected significant heterogeneity among cell types in PheWAS associations for 80% of genes (355 of 444 EUR PheWAS genes imputable in more than one cell type). Thus, snGReX-PheWAS provides a systematic framework to map pleiotropic GReX effects with explicit cell-type specificity. As an example, performing a focused EUR snGReX-PheWAS of the top 20 AUD GTAs (from the S-snTWAS and I-snTWAS analyses; AUD is the trait with the largest number of GTAs in I-snTWAS (Supplementary Fig. 19); Fig. 6c and Supplementary Fig. 24) both validates the associations with AUD and reveals pleiotropy consistent with our shared pathway analyses. Across these 20 genes, the AUD snGReX association profile correlated strongly with those for tobacco use disorder (*r*_(18)_ = 0.85, two-sided *P* = 1.39 × 10^−5^), BD (*r*_(18)_ = 0.73, two-sided *P* = 2.59 × 10^−4^) and dementias (*r*_(18)_ = 0.68, two-sided *P* = 9.09 × 10^−4^), in line with known AUD comorbidities and complications^44–46^ (Fig. 6c).

## Discussion

Before the widespread adoption of snRNA-seq approaches, genetically regulated gene expression in human brain cell populations was examined only in low-throughput studies^5,47–49^. More recent snRNA-seq resources have enabled a more systematic mapping of cell-type-specific expression in the human brain, but existing datasets have so far included at most several hundred EUR donors (192 (ref. ^50^) and 424 (ref. ^51^) donors) and have not modelled GReX in other ancestries. Here we have reported a large-scale brain snTWAS that uses TIMs for EUR (*N* = 920), AFR (*N* = 321) and AMR (*N* = 118) ancestries to study 12 brain disorders (NPDs and NDDs), along with phenome-wide analyses in the MVP cohort (1,428 EUR, 1,057 AFR and 725 AMR phecodes). Using these snTIMs, we identified trait-associated GReX changes that are missed in homogenate tissue, resolved their cell-type specificity, characterized shared and distinct effects across disorder pairs, quantified cross-ancestry concordance and performed large-scale snGReX-PheWAS in MVP. We have provided the snTIMs, results and summary statistics as a community translational resource for target prioritization and for defining the cellular contexts in which putative risk genes act.

Class and subclass-level snTIMs imputed more genes than either pseudo-homogenate snBulk (+20% and +21%, respectively) or a similarly sized homogenate bulk model^6^ from the same region (+23% and +23%, respectively), and did so with higher prediction accuracy. In our S-snTWAS of 12 NPDs and NDDs, we found that higher cellular resolution snTIMs yield more GTAs overall, with a higher likelihood that they are novel and clinically relevant, although we note that the higher power of snBulk individually yielded more GTAs than any one other individual snTIM. Of note, S-snTWAS was FDR-adjusted across all TIMs and traits to reduce the effect of differential power. Relative to the only published brain snTWAS in MDD^51^, we observed 6,095 more imputable and 31 more significant gene–cell-type combinations. Our thorough investigation into cell-type specificity, using both a probabilistic framework (mash in S-snTWAS) and a frequentist heterogeneity metric (*I*^2^ from MVP I-snTWAS), revealed that the most distinct signals across brain cell types arise from the class-immune population, consistent with its separate erythromyeloid origin^52^. Disease-relevant pathways were preferentially, and in some cases uniquely, detected in biologically relevant cell types.

To validate and interpret these pathway-level signals, we performed a MG-focused, module-level analysis in AD using freshly isolated human MG^19^. S-snTWAS module enrichments closely tracked differentially expressed gene signatures linked to AD pathology, predominantly in class-immune, and identified three negatively enriched immune modules that were also associated with amyloid plaque burden. These modules captured altered myeloid programs and *TREM1* dysregulation and prioritized the *ZYX*–*EPHA1-AS1* locus, implicating *ZYX* as a candidate coding effector whose predicted downregulation may impair MG stress responses to β-amyloid exposure^34^. Together, this AD case study illustrates how cell-type-resolved TWAS bridges genetic signals to MG co-expression modules that are directly anchored to neuropathological readouts.

Beyond single-disorder analyses, snTWAS also aids in uncovering cell-type-specific cross-disorder biology. Among 12 NPDs and NDDs, we observed an increased likelihood of shared trait-associated genes and shared pathway dysregulation in 75.8% and 47.0% of trait pairwise comparisons, respectively, revealing convergent gene-level and pathway-level dysregulation that would otherwise go undetected in homogenate TWAS. For example, although snBulk TWAS identified no shared pathways between AD and PD, MG-level analyses revealed 47 shared pathways, in line with local genetic correlation evidence^37^ and with heritability enrichment for MG annotations in both disorders^5,53^. Finally, in our snGReX-PheWAS, we validated S-snTWAS findings, demonstrated that GReX cell-type heterogeneity can vary across traits for a given gene, and identified cell-type-specific associations with genetically correlated traits, comorbid conditions and trait-related complications.

In cross-ancestry analyses, snTIM performance for EUR, AFR and AMR scaled with their respective training sample sizes, as expected. Despite lower power in AFR and AMR, snTIMs in these ancestries captured an additional 882 and 335 genes that could not be reliably imputed by EUR snTIMs. On average, the degree to which the expression of a gene is under genetic control was similar across ancestries and cell types, and we observed strong cross-ancestry correlation and concordance among top-ranked GTAs in both snTWAS and snGReX-PheWAS. At the same time, non-EUR snTWAS identified ancestry-specific GTAs and improved multi-ancestry fine-mapping of risk genes. Most previous work has emphasized the poor portability of EUR-trained TIMs to other ancestries^38,39^. Our work eliminates the need for mismatched ancestry application of TIMs, and we found that proper ancestry-matched study design can be highly informative for cross-ancestry NPD and NDD studies even with modest sample sizes, paralleling previous diverse ancestry blood monocyte TWASs^54–56^.

Overall, our study extends previous work by demonstrating that cell-type-specific snTIMs are critical for identifying genetically driven, potentially actionable expression changes for translational applications. Compared with bulk approaches at the gene level, we did not observe obvious downsides to moving to snRNA-seq, aside from cost and specific scenarios where capturing extranuclear RNA is necessary. Despite the technological differences in sequencing, the sample overlap between bulk and snBulk enables their direct comparison (bulk *N* = 924; snBulk *N* = 920; 311 samples in common). Of note, we observed a similar number of imputable genes and significant S-snTWAS GTAs between bulk and snBulk, supporting this approach. For optimal model performance, future designs should consider not only increasing the number of donors but also optimizing the number of nuclei captured per specimen (Supplementary Note 4). Current snRNA-seq protocols also provide limited information on isoform abundance, even though isoform-level regulation can explain more of the heritability than gene-level expression^57^. Incorporating isoform-level information is likely to further increase resolution and, in so doing, improve our understanding of the cell-type-specific architecture of brain disorders.

In summary, incorporating cell-type resolution and ancestry specificity into TWAS frameworks substantially increases gene discovery, refines causal inference and provides deeper insights into the biological underpinnings of neuropsychiatric and neurodegenerative disorders. By moving beyond bulk tissue analyses, we pave the way for more targeted therapeutic interventions that consider the intricate cellular landscape of the human brain.

## Methods

### Training of snTIMs

#### PsychAD cohort

Molecular profiling efforts of the PsychAD consortium include snRNA-seq from more than 6 million nuclei from the DLPFC of 1,494 unique donors^15^. As described in the Capstone paper^11^, we utilized quadratic discriminant analysis using the 1000 Genomes Project^58,59^ reference to divide the full cohort into five discrete superpopulations based on ancestry: EUR, AFR, AMR, East Asian and South Asian. Of these, 1,359 genotyped individuals (920 EUR, 321 AFR and 118 AMR)^11^ had undergone snRNA-seq-based profiling; East Asian and South Asian were excluded from TIM building due to lack of sufficient power. Furthermore, PsychAD is composed of three ‘subcohorts’ (Mount Sinai National Institute of Health Neurobiobank, National Institute of Mental Health Intramural research program Human Brain Collection Core and Rush Alzheimer’s Disease Center (RADC)), of which RADC is predominantly AFR (Supplementary Table 41). We utilized snRNA-seq data from all 8 cell-type classes, and 23 subclasses (excluding 4 subclasses that are identical to classes). Finally, we note that we utilized release 2.5 of PsychAD.

#### Genotype and gene expression quality control for TIM training

##### Generation of an ancestry-specific common variant reference list

The merged PsychAD genotype dataset consisting of PsychAD-Mount Sinai National Institute of Health Neurobiobank SNP array, CommonMind SNP array, RADC whole-genome sequencing (WGS) and Alzheimer’s Disease sequencing project WGS samples was prepared as previously described^15^. Towards harmonizing SNP utilization across the training (merged PsychAD genotype dataset) and target (GWAS for S-TWAS) cohorts and trans-omics for precision medicine (TOPMed)-imputed SNP arrays for the MVP), we constructed ancestry-specific common variant reference panels. First, we queried all variants in the TOPMed-imputed^60–63^ MVP’s^60^ genotype release 4 and retained non-ambiguous biallelic SNPs with reference SNP cluster identification annotation included in individuals from the MVP that passed the following filters (see below for variant-level and sample-level quality control): population-specific minor allele frequency (MAF) greater than or equal to 0.01 and population-specific genotype missingness less than or equal to 0.05. Consequently, ancestry-specific SNPs were retained if they also had a matched superpopulation-specific MAF greater than or equal to 0.01 in the 1000 Genomes Project to ensure SNP overlap with GWAS used in S-snTWAS analysis^58,59^. The resulting ancestry-specific common variant reference list comprises approximately 4.5, 7.5 and 5.1 million SNPs in EUR, AFR and AMR, respectively.

##### Variant-level filtering in the training cohort (PsychAD)

Variant-level filtering on the genotypes used for the TIM training requires that all the following variant inclusion criteria were met: SNPs exist in the ancestry-specific SNP list described above, MAF ≥ 0.01, based on ancestry-specific information from the National Center for Biotechnology Information Allele Frequency Aggregator (ALFA^64^; EUR: SAMN10492695; AFR: SAMN10492698; AMR: SAMN10492700), or an in-cohort MAF ≥ 0.01 when ALFA information was not available or the variant did not pass the ALFA filter, in-sample minor allele count of 5 or greater, a Hardy–Weinberg equilibrium *P* value of 10^−6^ or greater, and not falling within areas of high linkage disequilibrium, such as the major histocompatibility complex (Supplementary Table 42). Finally, missing information for SNP predictors in existing TIMs were replaced with double the in-sample MAF (representing the in-sample average genotype), rounded to the nearest integer. Overall, we replaced about 0.050% of genotypes used for EUR snTIM training, and 0.069% each in genotypes used for AFR and AMR snTIM training.

##### Gene expression quality control

As reported in Lee et al.^11^ and Zeng et al.^12^, dreamlet was used to create pseudobulked gene expression by summing reads from the same individual. Expression for each cell type and each individual was computed as the log_2_ counts per million with a pseudocount of 0.25 after aggregating all corresponding nuclei. A precision-weighted regression model was fit for each expressed gene in each cell type using the dreamlet package^65^ using covariates for age, sex, postmortem interval, mitochondrial rate, ribosomal rate and disease status. To control for varying sequencing depth among snRNA-seq libraries, Pearson residuals (that is, residuals divided by their standard errors) were computed for each expressed gene and cell type and used in downstream analysis.

We limited genes to those within our expression annotation, Ensembl 104 (refs. ^66,67^). Second, for each ‘subcohort’ within PsychAD (see ‘PsychAD cohort’), we independently scaled gene expression, performed probabilistic estimation of expression residuals (PEER)^68^ to identify and adjust for hidden factors driving gene expression differences, and quantile normalized the resulting residualized gene expression. We utilized fastQTL^69^ to determine the number of PEER factors (ranging from 5 to 50) that yield optimal genetic signal by identifying the point at which the number of significant expression quantitative trait loci (eQTLs; FDR (Benjamini–Hochberg method^21^)-adjusted two-sided *P* ≤ 0.05) is closest to 95% of the maximum number of significant eQTLs found across any assessed number of PEER factors. Finally, we combined residualized gene expression across the three subcohorts, and repeated scaling, PEER factor optimization and quantile normalization of the combined cohort to reduce the impact of the batch effect (Supplementary Fig. 30).

##### Training of cell-type-specific PrediXcan models

We used PrediXcan^7^ to create per-cell-type TIMs in each of three ancestries (EUR, AFR and AMR) using the quality-controlled genotypes and gene expression. Pre-processing and post-processing were done using standard scripts in R, Python 2 and Python 3. Imputable genes are considered passing *R*^2^_CV_ ≥ 0.01, FDR-adjusted (across cell types but within each ancestry) *P*_CV_ ≤ 0.05 and SNPs in model > 0. *R*^2^_CV_ and FDR-adjusted *P*_CV_ values are prediction performance *R*^2^ and prediction performance *P* value from the ‘PredictDB’ software^70^. These filtering criteria are comparable with that of other published TIMs—GTEx V8 Elastic Net models and Zeng-2024: *P*_CVA_ ≤ 0.05 (refs. ^51,71^); original PrediXcan models: *R*^2^_CV_ > 0.01 (ref. ^7^).

To compare gene imputation models across individual TIMs or ancestries, we utilized *R*^2^_CV_, a proxy of variance in gene expression explained by genetic variants serving as predictors. To compare gene imputation models across ancestries, in each pair of ancestries we selected gene–cell-type combinations confidently imputed in both ancestries, and Spearman’s correlation was performed on the resulting pairwise complete cases of confidently imputed genes.

We performed linear regression to assess the extent to which major snTIM metadata predictors (sample size and median number of nuclei contributing to the pseudobulk expression from every individual) influence snTIM performance (proxied by number of confidently imputed genes). We reported the adjusted *R*^2^ as a measure of the variation explained in snTIM performance for each predictor; adjusted *R*^2^ was estimated by the stats package^72,73^.

We compared snTIMs against a DLPFC EpiXcan TIM^16^ (referred to as bulk). We compared per-gene cross-validated performance with a two-sided sign test.

### GWAS phenotypes and category definitions

Traits for S-snTWAS analysis were drawn from publicly available brain disorder GWAS. To stabilize downstream analyses, inputs were date locked in 2024. At the time of publication, the PsychAD snTIMs trained in this project will be publicly released so users can freely apply these models to newer datasets.

We began with 16 traits. Four traits showed insufficient power for S-snTWAS when analysed individually, each yielding at most one FDR-significant gene and two genome-wide significant loci in their variant association results: binge-eating disorder^74^, post-traumatic stress disorder (PTSD)^75^, anxiety^76^ and obsessive–compulsive disorder^77^. We imposed a pragmatic threshold of five genome-wide significant loci to ensure robust power for S-snTWAS analysis. Our primary analyses therefore use 12 well-powered NPD and NDD GWAS summary statistics^75,78–88^ (Supplementary Table 12).

For targeted comparisons of I-snTWAS versus S-snTWAS (for example, Fig. 5a), we retained anxiety^76^ and PTSD^75^ GWAS, as both traits had adequate power for analysis in MVP (see below). For select analyses, traits were grouped as NPDs, NDDs and substance use disorders (SUDs). These groupings are analytical and not intended to mirror formal diagnostic taxonomies. We organized traits to maximize interpretability, guided by shared genetic architecture and hypothesized dominant cell type and pathway involvement. NPDs included migraines, SCZ, BD, anorexia (anorexia nervosa), insomnia, attention-deficit/hyperactivity disorder and MDD. NDDs included AD, PD, amyotrophic lateral sclerosis and multiple sclerosis. For primary analyses, we used only AUD from the SUD group; however, for category-level comparisons, SUD representation was expanded to include tobacco use disorder and cannabis use disorder.

### S-TWAS using summary statistics

We utilized MungeSumstats^89^ to standardize GWAS format and update reference SNP cluster identification annotations, and, where possible, we used ${n}_{\mathrm{effective}}=\,\frac{2}{\frac{1}{\mathrm{cases}}+\frac{1}{\mathrm{controls}}}$ as sample size. To ensure maximum utilization of TIM SNPs, we imputed missing variants in GWAS summary statistics based on established methods^90^ (Supplementary Fig. 31). Ancestry-specific imputation linkage disequilibrium panels were built on the 1000 Genomes Project with PLINK^91^ and the following parameters: --ld-window-kb 1000000 --ld-window 1000 --maf 0.01 --ld-window-r2 0. We used run_imputez^90,92^ using a maxWindowSize of 200. To validate imputed GWAS summary statistics, we randomly sampled 1,000 SNPs per chromosome common to the GWAS and linkage disequilibrium reference panel, and correlated real and imputed *z*-scores (Supplementary Fig. 31). Only SNPs with a GWAS imputation *R*^2^ ≥ 0.7 were retained. Finally, we performed S-TWAS for our TIMs (external data 1 (ref. ^20^) using summary-PrediXcan (S-PrediXcan)^28^ and Python 2. Of note, we filtered out genes located within the major histocompatibility complex, due to high linkage disequilibrium. Significant gene–cell-type combinations were determined using the FDR-adjusted two-sided *P* value^21^ cut-off of 0.05. The FDR-adjusted two-sided *P* value was calculated across all gene–cell-type combinations for each trait. Finally, we note that we did not perform GWAS imputation for tobacco use disorder and cannabis use disorder.

### Enrichment for clinically relevant genes

To validate associations from the S-TWAS, we performed gene set enrichment analysis for genes with entries in the ‘neurologicCentralNervousSystem’ and ‘neurologicBehavioralPsychiatricManifestations’ columns of the Clinical Synopsis tables of the Online Mendelian Inheritance in Man database^22^. For our analysis, we only considered gene-specific Online Mendelian Inheritance in Man entries and not entries corresponding to large loci (for example, regions spanning multiple genes), resulting in a final set of 1,949 genes. Query TWAS genes (nominal two-sided *P* ≤ 0.01; only protein-coding genes) were tested for enrichment against this gene set using a one-sided Fisher’s exact test followed by FDR adjustment^21^.

### Enrichment of novel genes

We qualified a GTA as novel if the association was not present in bulk DLPFC S-TWAS (FDR-adjusted two-sided *P* ≤  0.05 (ref. ^21^); FDR adjustment performed across all traits to mimic correction in S-snTWAS analysis) or MAGMA analysis (FDR-adjusted two-sided *P* ≤ 0.05; FDR adjustment performed across all traits to mimic correction in S-snTWAS analysis) performed using the MAGMA^24^ SNP2GENE function on the Functional Mapping and Annotation of GWAS (FUMA) web portal^23,24^. GWAS were prepared as above and then lifted over to GRCh37 using liftover within MungeSumstats^89,93^ as required by FUMA^89,93^. FUMA default parameters were used, except for the following: ‘genetype’ was set to ‘all’; the major histocompatibility complex region was custom defined to ‘28477797–33448354’ (to match the definition used in other analyses, accounting for genome build differences); window was set to 50 kb (upstream and downstream). The union (∪) of FDR-significant S-TWAS and MAGMA hits was used to define our known list of GATs. Significant GTAs from the S-snTWAS not within this list were defined as novel. To test whether novel GTAs were enriched in snTIMs, we separated significant GTAs into two categories: significant in snBulk, and significant only in snTWAS (either class or subclass level; FDR-significant across all cellular populations). We used a two-sided Fisher’s exact test to obtain ORs and Bonferroni-corrected two-sided *P* values for the enrichment of novel GTAs in cell-type-specific TIMs.

### TWAS fine-mapping

To address potential horizontal pleiotropic effects and account for linkage disequilibrium among SNPs utilized in snTIMs, we utilized FOCUS^25^. FOCUS models the marginal TWAS *z*-scores as a multivariate Gaussian distribution given the estimated eQTL effect size and the SNP correlation and utilizes a Bayesian approach to calculate the marginal PIP for each gene, indicating its likelihood of being causal in a specific TWAS risk region. Owing to internal GWAS imputation in FOCUS, we utilized post-munging GWAS summary statistics from our pipeline before missing SNP imputation. PrediXcan-based snTIMs were converted to FOCUS-compatible format after retaining only imputable genes. FOCUS was applied both individually to each snTIM to assess effects within each cell type, and jointly (multi-cell type) for further prioritization. Unless otherwise specified, we considered TWAS-significant associations with PIP ≥ 0.5 to be fine-mapped. To quantify the proportion of S-snTWAS-significant GTAs that were fine-mapped, we summed, for each trait–snTIM combination, the number of significant GTAs with fine-mapping support and divided by the total number of significant GTAs.

We evaluated whether fine-mapped genes increase in class and subclass aggregates by assessing the total number of unique fine-mapped genes and loci in each snTIM versus the respective aggregate. Because our primary analyses utilized FOCUS applied to individual snTIMs, we repeated the analysis using multi-cell-type fine-mapping. We also applied the Wilcoxon rank-sum test to compare genes per locus in aggregates versus individual snTIMs.

To perform bi-ancestry fine-mapping for EUR and AFR I-snTWAS in MVP, we used MA-FOCUS^94^. We utilized TOPMed-imputed^61^ genotypes in MVP so that no additional imputation was needed for SNP predictors in the snTIMs; individual-level SNP missingness was handled as above. To visualize the top AFR fine-mapped associations and their concordance with EUR fine-mapped associations and MA-FOCUS, we plotted the scaled I-snTWAS *z*-scores within each cell type and ancestry (for meta-analysis, we performed inverse-variance weighted meta-analysis using I-snTWAS effect size and standard error and scaled the resulting *z*-score). We restricted the analysis to class-level snTIMs and prioritized the top ten associations by ranking each gene by the maximum scaled AFR I-snTWAS *z*-score × AFR fine-mapping PIP across all considered cell-types.

### mash Analysis

We implemented mash^26^ using the mashr package in R. Owing to differences in the imputability of genes across snTIMs at the subclass level, GReX matrices are sparse; thus, we limited analysis to class-level snTIMs (Supplementary Fig. 32). Moreover, we excluded EUR classes Endo and mural, which confidently imputed a much smaller number of genes in comparison to other class-level snTIMs. Then, we performed mash on *z*-scores set as effect size and standard errors set to 1; we did not use the reported association effect sizes due to limitations in accurate effect size estimation in S-PrediXcan^28^ and other TWAS methods^28^. We filled missing values (where we do not have a model for a given gene in a given cell type), with a *z*-score of 0 and an arbitrarily high standard error (1 × 10^10^). We ran mash using data-driven covariances as recommended by the mashr authors. The mash analysis was used to obtain the probabilities of an effect (GTA) existing in a given cell type (for example, Extended Data Fig. 3a) and to assess cell-type specificity in S-snTWAS (Fig. 3a,b). For the latter, we first limited the analysis to all S-snTWAS-significant GTAs within the six cell types utilized in the mash analysis above, and further restricted GTAs to the ones having at least one but less than six (all) cell types with a significant association (local false sign rate ≤ 0.05; due to Bayesian statistics, not all S-snTWAS significant GTAs are necessarily significant in mash results).

### Comparison between S-snTWAS and I-snTWAS using the same genetic data

I-snTWAS association analyses were performed as described above. For comparison, S-snTWAS association analyses were conducted using S-PrediXcan^28^ with genome-wide variant associations derived from the same samples, binary outcomes and covariates. The resulting *z*-scores and effect sizes were compared with Pearson’s correlation to demonstrate the limitations of S-snTWAS in estimating accurate association effect sizes.

### GTA heterogeneity analysis

I-snTWAS and snGReX-PheWAS heterogeneity statistics were calculated for S-snTWAS significant (FDR-adjusted two-sided *P* ≤ 0.05) GTA effect sizes in R using the metafor package^95^. Heterogeneity^30^ *I*^2^_cell type_ was calculated for genes imputable in at least two cell-type TIMs.

### Targeted sn-eQTL analysis

To validate heterogeneity found in the same gene across cell types, we utilized PsychAD eQTLs calculated among EUR using multivariate multiple quantitative trait loci^12^. We used the qtlPlots R package (v0.0.7) to visualize eQTLs^96^. The top SNP for each cell type was extracted and linkage disequilibrium between them was examined using LDlink^31^.

### TWAS pathway enrichment analysis

We used joint effect on phenotype of eQTLs associated with a gene in mixed cohorts-pathways (JEPEGMIX2-P)^32^ to perform linkage disequilibrium-aware competitive pathway enrichment analysis. For this analysis, we used GRCh37-aligned GWAS summary statistics (prepared as above in ‘Enrichment of novel genes’) and snTIMs converted into JEPEGMIX2-P-compatible annotation files. In addition, we incorporated biological pathways from Gene Ontology (Gene Ontology 2015), accessed through Molecular Signatures Database (MSigDB) 5.1. JEPEGMIX2-P is designed to perform pathway enrichment analysis while accounting for linkage disequilibrium structures among genetic variants. For this study, the software was enhanced to conduct competitive analyses using the correlation adjusted mean rank (CAMERA)^97^ gene set test procedure, allowing for a more refined understanding of gene–pathway associations; the publicly available binary executable file was updated to include this enhancement. The derived two-sided *P* values were FDR-adjusted^21^ among all pathways across all ancestry-specific TIMs, and an 0.05 FDR-adjusted two-sided *P* value threshold was used to determine significance. To obtain more conservative estimates of the number of significant pathways per cell type while maintaining specificity, we removed all significant pathways that were ‘parents’ of other significant pathways (referred to as pathway pruning in the main text). Finally, we note that we removed the *MAPT* locus (defined as chromosome 17: 44928498−56807609 in GRCh38) due to the presence of haplotypes that may bias results^98^. Of note, these haplotypes have been found to have a large role in AD, PD and other disorders^98^.

### Comparison of MG AD snTWAS with differential gene expression signatures associated with AD pathology

We utilized transcriptional profiling data from freshly isolated primary human MG, along with the corresponding AD-relevant differentially expressed gene signatures and hierarchical co-expression networks^19^, which were constructed using multiscale embedded gene co-expression network analysis (MEGENA)^99^. The MEGENA network was constructed from FACS-sorted CD45^+^ primary human MG from 189 autopsy samples (both with and without AD-associated pathology; comprising a partially intersecting set of the FACS-MG cohort and utilizing standard settings, including: Pearson’s correlation, un-signed and minimum module size of at least ten genes. For further analyses, we retained 306 co-expression modules with 50 or more genes each, and evaluated their enrichment for differentially expressed genes and S-snTWAS signatures using the competitive gene-set test CAMERA^97^ implemented in the limma R package^100^ with default settings, using pre-ranked *z*-scores from the differentially expressed gene and S-snTWAS analyses. The clinical dementia rating (CDR) phenotype indicates CDR ≥ 1 versus CDR = 0, and ‘Braak’ is a categorical phenotype separating high values ≥ 5 and low values ≤ 2. β-Amyloid density indicates the mean of this metric across five measured brain regions. Functional annotation enrichment of each MEGENA module using signatures from MSigDB (v7.2)^101^ was assessed using Fisher’s exact test. The relationship between differentially expressed genes and S-snTWAS was evaluated via Pearson’s correlations between the significance estimates (signed −log_10_(two-sided *P* value)) of the enrichment analyses across all modules. The modules M406, M824 and M947 were the only modules with FDR-adjusted two-sided *P* ≤ 0.05 in the S-snTWAS enrichments via CAMERA pre-ranked (CameraPR).

### PTCA

To assess and visualize the concordance of *z*-scores between two datasets (for example, TWAS or summary statistics from competitive pathway enrichment analysis) at increasing significance thresholds, we performed the following analysis, which we termed PTCA. First, we scaled *z*-scores among all values in each dataset (using R’s base scale function, to normalize the values with a mean of 0 and a standard deviation of 1). Next, we matched identifiers (for example, trait–cell-type–gene or trait–cell-type–pathway) between the two datasets and restricted the final dataset to elements common to both datasets. Then, we performed a fixed-effect inverse-variance weighted meta-analysis using the *z*-scores of the dataset (standard error is assumed to be 1), and sorted the table based on the meta-analysis two-sided *P* value (increasing order). Finally, we measured the correlation between ordered and scaled *z*-scores in a step-wise restrictive manner (assuming step size = 10 and the dataset consists of 1,000 common elements, we assessed the correlation between the top 1,000 elements, followed by the top 990 elements, and so on).

### Cross-disorder analysis

To compare snTWAS results across disorders, we used PTCA (described above) to assess how cross-trait *z*-score correlations strengthen as significance thresholds tighten, and two-sided Fisher’s exact tests to test enrichment of shared significant associations. For validation, we repeated the cross-disorder analyses with I-snTWAS in non-overlapping samples (no known overlap except for MDD) and required S-snTWAS-significant gene–cell-type associations to show concordant directions in I-snTWAS. This validation was not performed for trait pairs lacking adequate MVP power or for MDD, whose GWAS includes MVP participants. For all gene-level and pathway-level comparisons, we excluded the major histocompatibility complex and *MAPT* (GRCh38 chromosome 17: 44928498–56807609) regions due to haplotype complexity that can bias results (see ‘TWAS pathway enrichment analysis’)^98^.

### I-TWAS and snGReX-PheWAS in MVP

#### MVP genotype quality control

The data core team from the MVP handled genotyping, SNP imputation and initial quality control of genotypes. For this study, we utilized the MVP Release 4, which includes genotypes from 662,681 individuals. DNA was extracted from whole blood and genotyping was performed with the MVP 1.0 custom Axiom Array^60^. The MVP data core called genotypes using assay for transposase-accessible chromatin (ATAC) ATAC Primer Tool (v2.11.3), Affy2vcf, PLINK^91^ and MVP 1.0 array library r6. Genotyping was validated using three plates of 1000 Genome samples. Rigorous genotype quality control such as plate normalization was used to improve the accuracy of genotype calling. Genotype imputation was performed using the TOPMed^61^ reference, SHAPEIT4 (v4.1.3)^102^ and Minimac4 (ref. ^62^), and genetic principal components were generated using EIGENSOFT (v6)^103,104^. We then performed sample-level and variant-level quality control on genotypes. Our quality control pipeline is based on previously established methods for other genetic analyses and optimized for maximizing input information for GReX estimation^6,74,105,106^. We grouped samples by ancestry based on harmonized ancestry and race/ethnicity^107^ (a method that integrates and harmonizes self-identified ancestry and genetic ancestry) into three ancestries (438,582 EUR, 112,346 AFR and 48,726 AMR). Using only autosomal variants, we filtered variants for MAF (0.01 or more), Hardy–Weinberg equilibrium *P* value (less than 1 × 10^−6^) and removed high disequilibrium regions (GRCh37; chromosome 6: 25500000−33500000, chromosome 8: 8135000−12000000 and chromosome 17: 40900000−45000000)^108^. We then filtered for excess heterozygosity (four or more standard deviations from the mean), ambiguous sex (using both PLINK’s check-sex option, as well as sex chromosome aneuploidies assignments derived from plate intensity measurements) and relatedness (kinship-based inference for GWAS (KING) filter = 0.0884)^91,109^. Finally, we limited variants to biallelic SNPs for individual imputation of GReX.

#### MVP phenotypes

Phenotypes from International Classification of Diseases-9/10 codes were transformed into phecodes using Phecode Map (v1.2)^110,111^. Individuals with at least two phecodes for a given trait were considered a case for both I-snTWAS and snGReX-PheWAS analyses. Owing to the enrichment of neuropsychiatric disorders within the MVP and their high comorbidity, individuals with fewer than two phecodes were considered a control for both analyses. For the I-snTWAS analysis, only traits with at least 2% case prevalence in EUR, AFR and AMR were utilized for power considerations (Supplementary Table 12), and AD was mapped to the phecode for ‘delirium dementia and amnestic disorders’, 290, due to the way AD is routinely coded in the Veteran Affairs medical system^112^. For the snGReX-PheWAS analysis, traits with at least 500 cases and 500 controls were considered.

#### GReX imputation

We estimated GReX in the MVP on an individual level using TIM-derived SNP predictor weights. Missing genotypes were replaced with double the in-sample MAF (representing the in-sample average genotype), rounded to the nearest integer.

#### I-snTWAS association analysis

Logistic regression analysis was performed while adjusting for sex, age and the top ten genetic principal components. Multiple test correction with FDR-adjusted two-sided *P* value^21^ (0.05 or less) was performed across all cell types, ancestries and traits. For comparison of I-snTWAS with S-snTWAS, we also utilized GWAS summary statistics for anxiety^76^ and PTSD^75^ (Fig. 4c and Supplementary Table 12).

#### Gene selection for snGReX-PheWAS

Owing to computational resource limitations, PheWAS analysis was performed on the top-ranked genes from the S-snTWAS and I-snTWAS. We selected significant genes from our S-snTWAS and I-snTWAS (FDR-adjusted two-sided *P* ≤ 0.05), and performed PheWAS on the top associations (Bonferroni-adjusted two-sided *P* ≤ 0.05 across all confidently imputable associations within each cell type in S-snTWAS or I-snTWAS and at least FDR-adjusted two-sided *P* ≤ 0.05 in the complementary snTWAS analysis; 623 unique genes) to maximize analytical yield while remaining within computational constraints. S-snTWAS genes were considered across all cell types in EUR. I-snTWAS genes were considered across all cell types and ancestries. This resulted in a total of 9,611 PheWASs (approximately 6.42% of all imputable coding gene–cell-type combinations) across 623 unique genes.

#### snGReX-PheWAS association analysis

As in I-snTWAS, logistic regression analysis was performed while adjusting for sex, age and the top ten genetic principal components. FDR-adjustment was performed across all considered phecodes, 623 selected genes, 3 ancestries and all cell types for considered genes. Associations with FDR-adjusted two-sided *P* ≤ 0.05 (ref. ^21^) were considered significant.

### snGReX-PheWAS clustering

To explore the pleiotropic effects on multiple phenotypes of the top snTWAS GTAs, we performed clustering of snGReX-PheWAS results. For interpretation of associations between brain disorders, we limited phenotype categories to ‘mental disorders’ and ‘neurological’. FDR adjustment was performed across all phecodes in these categories, as well as across all 623 PheWAS genes (in all cell types and ancestries) as typical. For every trait, we extracted the top 20 S-snTWAS and I-snTWAS significant genes (FDR-adjusted two-sided *P* ≤ 0.05 (ref. ^21^)) in association with the target phecode (Supplementary Table 12). We extracted only the top associated gene–cell-type combination for each gene to prevent clustering due to homogeneity of GReX across cell types. We then extracted up to the top 20 phecodes significantly associated (FDR-adjusted two-sided *P* ≤ 0.05 (ref. ^21^)) with these gene–cell-type combinations. Ward’s hierarchical agglomerative clustering^113^ was performed on the association *z*-scores with an implementation that preserves Ward’s criterion^114^ (ward.D2 method in R). Finally, we note that we removed the *MAPT* locus (defined as chromosome 17: 44928498−56807609 in GRCh38) due to the presence of haplotypes that may bias results (see ‘TWAS pathway enrichment analysis’)^98^.

### Abundance analysis of imputable genes

We hypothesized that genes uniquely identified by snBulk versus other snTIMs were lower in abundance. To investigate this, we subsetted imputable genes into those uniquely identified by snBulk (571) and those identified by at least one other snTIM (8,388). We calculated the mean number of transcripts for every gene among all EUR individuals with available expression data for the gene. To establish the statistical significance of the difference in mean transcript count, we used the Wilcoxon rank-sum test.

### Out-of-sample validation of PsychAD snTIMs

#### Validation of class-level PsychAD snTIMs in ROSMAP

We performed external validation of EUR snTIMs using WGS and snRNA-seq from 396 ROSMAP^17^ participants of EUR. WGS aligned to GRCh37 was lifted over to GRCh38 (ref. ^93^). To determine sample ancestry, the ROSMAP genotypes underwent standard genome-wide quality control procedures^115,116^. We performed an initial round of variant quality control by removing variants with less than 0.95 call rate, to avoid biases in our sample quality control. Then, we proceeded with the removal of samples that fit any of the following criteria: call rate < 0.98, absolute value of the inbreeding coefficient < 0.2, genomic sex discrepancy with reported sex and formation of pairs with relatedness (PLINK’s $\hat{\pi }$ calculated via identity by descent) > 0.1875 (ref. ^91^). We applied variant quality control, excluding markers with call rate < 0.98, and Hardy–Weinberg equilibrium *P* < 10^−6^. For ancestry validation and assignment, the resulting dataset was subsequently merged with the 1000 Genomes Project reference panel, keeping variants with MAF > 0.01 and pruned with a window size of 100, a step size of 5 and a pairwise *R*^2^ threshold of 0.1. The samples were subsequently projected onto the eigenvectors computed on the 1000 Genomes Project samples using EIGENSOFT’s smartpca^103^. Finally, we utilized quadratic discriminant analysis-determined EUR ROSMAP samples to impute individual-level GReX using PsychAD snTIMs (the ROSMAP WGS covered 99.7% of SNPs used by the PsychAD snTIMs).

Cells from the ROSMAP snRNA-seq dataset were annotated using the PsychAD taxonomy. We first subsampled 1 million cells from both the PsychAD and the ROSMAP snRNA-seq datasets, and then used the mapping between the two datasets to infer the labels for the full dataset. We used single-cell annotation using variational inference^117^ to perform reference-based label transfer. After subsetting the genes shared between two datasets, we used the scvi-tools package^118^ to train the scVI model based on the reference dataset and inferred cell-type labels (for example, class and subclass) on the query dataset. Models were run with 5 hidden layers and 10 latent variables, and the single-cell annotation using variational inference model was trained for 20 epochs with a minimal sample of 100 cells per cluster per epoch. Last, a transfer model was trained for 100 epochs and applied to query data to assign labels based on those the model was trained on from the reference. Label transfer achieved 91.6% accuracy when evaluated using true labels from the reference dataset. ROSMAP pseudobulk data were processed identically to PsychAD.

snRNA-seq profiles were clustered according to the PsychAD taxonomy and pseudobulked per cell type. Gene expression quality control matched the PsychAD pipeline above, and pseudobulk expression was PEER residualized. For each gene–cell-type combination present in both datasets, we computed the Pearson’s correlation across individuals between observed expression and GReX, defining out-of-sample *R*^2^ as *r*^2^. Agreement with internal cross-validated performance was assessed by Spearman’s correlation of out-of-sample *R*^2^ versus *R*^2^_CV_.

#### Validation of PsychAD subclass-MG in FACS-MG

To perform out-of-sample validation in FACS-MG, individual genotypes (filtering detailed in ‘S-snTWAS validation’ below) were leveraged to calculate GReX using the PsychAD EUR subclass-MG snTIM. GReX was then compared with PEER-residualized FACS-MG RNA-seq (see ‘S-snTWAS validation’ below). For each gene present in both datasets, we computed the Pearson’s correlation across individuals between observed expression and GReX, defining out-of-sample *R*^2^ as *r*^2^. Agreement with internal cross-validated performance was assessed by Spearman’s correlation of out-of-sample *R*^2^ versus *R*^2^_CV_.

### S-snTWAS validation

We validated S-snTWAS associations using two external datasets: FACS-MG and Zeng-2024 (ref. ^51^). To prepare the FACS-MG TIM, we utilized similar protocols to the PsychAD snTIMs. FACS-MG cohort samples were derived from one of three different sub-cohort (based on the site of sample preparation). Genotypes were TOPMed-imputed and a EUR SNP reference panel similar to that used for PsychAD snTIM creation was used to initially select SNPs. The 1000 Genomes Project overlap was not implemented for the reference panel used in FACS-MG TIM creation due to the application of the TIM to a single GWAS: SNP utilization for the FACS-MG AD S-TWAS was more than 95%. Samples were filtered for missingness (0.01 or less) and relatedness (KING filter = 0.0884). To perform population stratification, variants were filtered for MAF (0.05 or more), missingness (0.01 or less) and Hardy–Weinberg equilibrium *P* value (less than 10^−10^). Variants were pruned using PLINK’s ‘--indep-pairwise’ function (1000 10 0.02). Twenty principal components were calculated and principal component analysis was used to determine EUR individuals (EUR selection ellipsoid defined using three standard deviations and three principal components). After ancestry filtering, sample-level quality control was performed. Variants were filtered for missingness (0.01 or less) before sample filtering for missingness (0.01 or less). Subsequently, only autosomal variants were retained, and variants were filtered for MAF (0.05 or more) and Hardy–Weinberg equilibrium *P* value (less than 10^−6^). High linkage disequilibrium regions were then filtered out (Supplementary Table 42) before pruning using PLINK’s ‘--indep-pairwise’ function (50 5 0.02). We then filtered for excess heterozygosity (three or more standard deviations from the mean) before filtering samples for relatedness (KING filter = 0.0884). After retaining only quality-controlled EUR samples, downstream variant filtering was performed identically to PsychAD. RNA-seq was processed identically to PsychAD snRNA-seq, including the two step PEER factor optimization on the three sub-cohorts. After filtering, we retained 271 EUR individuals with genotypes and RNA-seq. Finally, the FACS-MG TIM was filtered identically to PsychAD snTIMs.

FACS-MG TWAS was compared with EUR subclass-MG snTWAS using publicly available AD GWAS^78^. For comparison parity, FDR adjustment was applied to all confidently imputable coding genes (after removing the major histocompatibility complex locus) in FACS-MG and subclass-MG separately. We then used *z*-scores from each to compare TWAS using Pearson’s correlation.

PsychAD class-level snTIMs were compared to Zeng-2024 snTIMs (Supplementary Table 39) using the same MDD GWAS summary statistics^119^. GWAS summary statistics were imputed for missing SNPs to ensure adequate coverage of PsychAD snTIM SNPs. Zeng-2024 TWAS was publicly available, and identical snTIM filtering to PsychAD was applied (*R*^2^_CV_ ≥ 0.01, FDR-adjusted two-sided *P*_CV_ ≤ 0.05). For comparison parity, FDR adjustment was applied to all confidently imputable coding genes (after removing the major histocompatibility complex locus) in Zeng-2024 and PsychAD separately. We then used *z*-scores from each to compare TWAS using Pearson’s correlation.

### Genetic correlation analysis

We performed bivariate heritability analysis using linkage disequilibrium score regression^120,121^ to assess the genetic correlation between the aforementioned seven NPD, four NDD and three SUD GWAS summary statistics. The datasets were munged to match the HapMap3 SNP allelic information, and the linkage disequilibrium weights were pre-calculated on the 1000 Genomes Project EUR dataset^122^.

### Statistics and reproducibility

To demonstrate reproducibility, we performed multiple independent and internal validation analyses. Here we summarize five key examples. (1) We performed out-of-sample replication of PsychAD snTIMs in the ROSMAP and FACS-MG cohorts (Supplementary Fig. 26a,c). We correlated imputed GReX with observed PEER-residualized snRNA-seq expression and found strong concordance between out-of-sample performance (*R*^2^_out_) and cross-validation performance (*R*^2^_CV_; Spearman’s *ρ* = 0.63; *N* = 70,156; *P* = 3.91 × 10^−7,844^, and *ρ* = 0.55; *N* = 1,853, *P* = 1.67 × 10^−149^, respectively). (2) We compared S-snTWAS results derived from PsychAD snTIMs with those obtained using independent transcriptomic imputation models, including previously published snTIMs^51^ and a MG-specific TIM derived from FACS-MG^19^ (Supplementary Fig. 26b,d). In both cases, we observed strong concordance (Pearson’s *r*_(13,760)_ = 0.78; *P* = 1.34 × 10^−2,772^ and Pearson’s *r*_(733)_ = 0.87; *P* = 1.32 × 10^−231^, respectively). (3) To address potential bias in the cross-disorder analysis from sample overlap across GWAS datasets, we replicated the analysis replacing S-snTWAS data with independent MVP I-snTWAS data and evaluated sign concordance of results (Supplementary Fig. 14). Overall, 90% of cross-disorder associations (5,162 of 5,721) showed consistent direction of effect. (4) We further compared S-snTWAS with MVP EUR I-snTWAS (Fig. 5a). Although the overall Pearson correlation across all associations was modest, PTCA demonstrated strong concordance among top-ranked associations. (5) Finally, snTIM training incorporated fivefold cross-validation, consistent with the broader use of cross-validation for evaluating PrediXcan-style transcriptomic imputation models.

### Reporting summary

Further information on research design is available in the Nature Portfolio Reporting Summary linked to this article.

## Online content

Any methods, additional references, Nature Portfolio reporting summaries, source data, extended data, supplementary information, acknowledgements, peer review information; details of author contributions and competing interests; and statements of data and code availability are available at https://doi.org/10.1038/s41586-026-10836-6.

## Supplementary information

Supplementary Information Supplementary Notes 1–7, Supplementary Figs. 1–32, descriptions for Supplementary Tables 1–42 (tables supplied separately), legends for External Data 1–4, Supplementary References and Supplementary Acknowledgements.

Reporting Summary

Supplementary Tables Supplementary Tables 1–42

Supplementary Data Source Data for Supplementary Figs. 1–32.

Peer Review file

## Source data

Source Data Figs. 1, 2, 3, 4, 5, 6 and Source Data Extended Data Figs. 3, 4

## Data Availability

All results are included either in the main text or provided in Supplementary Tables or external data^20^. We are also making snTIMs and accompanying SNP covariance matrices publicly available on Zenodo^123^ (https://doi.org/10.5281/zenodo.17435917) and Synapse^20^ (https://doi.org/10.7303/syn63181047). The PsychAD single-nucleus transcriptomics data are available via the AD Knowledge Portal (https://doi.org/10.7303/9618136). Source data are provided with this paper.

## Code availability

This project utilized publicly available code as described in the Reporting Summary.
