# Supplementary material for: Single-nucleus transcriptome-wide association study of human brain disorders
Source: Nature. 2026 Sep 23;657(8133):1016–26. doi: 10.1038/s41586-026-10836-6 (PMC13600940; doi:10.1038/s41586-026-10836-6)
Supplement: Supplementary file 1 [file 41586_2026_10836_MOESM1_ESM.pdf]

nature portfolio

https://doi.org/10.1038/s41586-026-10836-6

Supplementary information

Single-nucleus transcriptome-wide association study of human brain disorders

In the format provided by the authors and unedited

SUPPLEMENTARY INFORMATION

Single-nucleus transcriptome-wide association study of human brain disorders

Authors: Sanan Venkatesh[1-5], Roman Kosoy[1-4]*, Zhenyi Wu[1-5]*, Marios Anyfantakis[1-5], Christian Dillard[1-4], Prashant N.M.[1-4], David Burstein[1-6], Deepika Mathur[1-6], Chris Chatzinakos[7,8], Bukola Ajanaku[1-4], Fotis Tsetsos[1-4,6], Biao Zeng[1-4], Sonali Gupta[1-4], Rachel Bercovitch[1-4], Aram Hong[1-4], Clara Casey[1-4], Marcela Alvia[1-4], Zhiping Shao[1-4], Stathis Argyriou[1-4], Karen Therrien[1-4], PsychAD Consortium**, Tim Bigdeli[7-10], Pavan Auluck[11], David A. Bennett[12], Stefano Marenco[11], Vahram Haroutunian[1-5], Kiran Girdhar[1-4], Jaroslav Bendl[1-5], Donghoon Lee[1-4], John F. Fullard[1-4], Gabriel E. Hoffman[1-6], Georgios Voloudakis[1-6,13]***, Panos Roussos[1-6]***

Affiliations:
[1]Department of Psychiatry, Icahn School of Medicine at Mount Sinai, New York, NY, USA
[2]Center for Disease Neurogenomics, Icahn School of Medicine at Mount Sinai, New York, NY USA
[3]Friedman Brain Institute, Icahn School of Medicine at Mount Sinai, New York, NY, USA
[4]Department of Genetics and Genomic Science, Icahn School of Medicine at Mount Sinai, New York, NY, USA
[5]Mental Illness Research, Education, and Clinical Center (VISN 2 South), James J. Peters VA Medical Center, Bronx, NY, USA
[6]Center for Precision Medicine and Translational Therapeutics, James J. Peters VA Medical Center, Bronx, NY, USA.
[7]Department of Psychiatry and Behavioral Sciences, SUNY Downstate Health Sciences University, Brooklyn, NY, USA
[8]Institute for Genomics in Health (IGH), SUNY Downstate Health Sciences University, Brooklyn, NY, USA
[9]VA New York Harbor Healthcare System, Brooklyn, NY, USA
[10]Department of Epidemiology and Biostatistics, School of Public Health, SUNY Downstate Health Sciences University, Brooklyn, NY, USA
[11]Human Brain Collection Core, National Institute of Mental Health-Intramural Research Program, Bethesda, MD, USA
[12]Rush Alzheimer's Disease Center, Rush University Medical Center.
[13]Department of Artificial Intelligence and Human Health, Icahn School of Medicine at Mount Sinai, New York, NY, USA
*co-second
**A list of authors and their affiliations appears at the end of the paper.
***co-senior and co-corresponding

Table of Contents

TABLE OF CONTENTS **2**
SUPPLEMENTARY NOTES **4**
Supplementary Note 1. Genes imputable only in Bulk largely reflect technological differences 4
Supplementary Note 2. Factors underlying number of confidently imputable genes in each cell-type 4
Supplementary Note 3. Out-of-sample validation of snTIMs and summary-level transcriptome-wide association studies (S-snTWAS) 4
Supplementary Note 4. Cross-validated training performance across cell types and ancestries 5
Supplementary Note 5. Expanded analysis of snTIMs across ancestries 5
Supplementary Note 6. Multi-cell-type fine-mapping is consistent with individual-cell-type fine-mapping 6
Supplementary Note 7. Comparison of neuropsychiatric disorders (NPDs), neurodegenerative disorders (NDDs), and substance use disorders (SUDs) 6
SUPPLEMENTARY FIGURES **7**
Supplementary Fig. 1 7
Supplementary Fig. 2 8
Supplementary Fig. 3 9
Supplementary Fig. 4 10
Supplementary Fig. 5 11
Supplementary Fig. 6 12
Supplementary Fig. 7 13
Supplementary Fig. 8 14
Supplementary Fig. 9 18
Supplementary Fig. 10 19
Supplementary Fig. 11 21
Supplementary Fig. 12 22
Supplementary Fig. 13 23
Supplementary Fig. 14 27
Supplementary Fig. 15 28
Supplementary Fig. 16 29
Supplementary Fig. 17 30
Supplementary Fig. 18 31
Supplementary Fig. 19 32
Supplementary Fig. 20 33
Supplementary Fig. 21 35
Supplementary Fig. 22 40
Supplementary Fig. 23 42
Supplementary Fig. 24 43
Supplementary Fig. 25 47
Supplementary Fig. 26 48
Supplementary Fig. 27 50
Supplementary Fig. 28 51
Supplementary Fig. 29 53
Supplementary Fig. 30 55

Supplementary Fig. 31 57
Supplementary Fig. 32 59
SUPPLEMENTARY TABLES 60
EXTERNAL DATA 69
SUPPLEMENTARY ACKNOWLEDGMENTS 70

Supplementary Notes

Supplementary Note 1. Genes imputable only in Bulk largely reflect technological differences

We found that 4,090 genes (1,505 coding) were imputable only in Bulk and not in single-nucleus transcriptomic imputation models (**snTIM**s). These discrepancies can be partly explained by differences in sequencing technology and by detection of extranuclear RNA in bulk homogenate, consistent with the compartment-specific localization of many long non-coding RNAs (**lncRNAs**)[124]. Transcripts unique to each Bulk and snBulk were enriched for lncRNAs (Fisher's exact test): unique to Bulk, odds ratio (**OR**) = 1.56, p-value = 3.00×10^{-13}; unique to snTIMs, OR = 5.75, p-value = 2.95×10^{-496}.

Supplementary Note 2. Factors underlying number of confidently imputable genes in each cell-type

We explored the major factors associated with European ancestry (**EUR**) snTIMs and found that the number of individual donors and median nuclei per cellular population per individual explain 66.3% of variance in the number of imputable genes (Supplementary Fig. 25; Table S38). Previously, we found that sample size was the main driver for unique gene-trait association (**GTA**) discovery in tissue homogenates[125]. However, in single-cell experiments, when sequencing depth is not a limiting factor, snTIM performance also heavily depends on the number of cells being profiled. Consequently, there is a fine balance between increasing resolution and retaining information when using standard single-nucleus RNA sequencing (**snRNA-seq**) approaches. Specifically, using a pseudobulk approach reduces measurement noise, while increasing statistical power from sparse single cell measurements[125–128]. Thus, for low abundance transcripts, imputation performance improves when we pool more cells together; for example, imputable coding genes by EUR snBulk that were not predictable via finer cell-type resolution (n = 571) were less abundant (392 mean counts per individual vs. 3,585; Wilcoxon rank-sum p-value = 1.20×10^{-170}).

Supplementary Note 3. Out-of-sample validation of snTIMs and summary-level transcriptome-wide association studies (S-snTWAS)

First, in the Religious Orders Study/Memory and Aging Project (**ROSMAP**) cohort, we applied the PsychAD cellular taxonomy to cluster and pseudobulk the snRNA-seq data. We then imputed cell-class-specific gene expression using ROSMAP genotypes and the PsychAD snTIMs. Comparing imputed genetically regulated gene expression (**GReX**) with observed gene expression in ROSMAP yielded R^2_{out} estimates that closely matched our R^2_{CV} predictions (Spearman's ρ = 0.63; N = 70,156; p-value = $3.91 \times 10^{-7,844}$; Supplementary Fig. 26A), demonstrating external validity and showing no evidence for feature-engineering-induced leakage which can inflate R^2_{CV} estimates. Next, we performed major depressive disorder (**MDD**) S-snTWAS using our PsychAD snTIMs and compared these results to a recently published independent ROSMAP-based S-snTWAS (**Zeng-2024**)[51]. After matching cell classes across cohorts (Table S39), we correlated the S-snTWAS z-scores based on

the same MDD genome-wide association study (**GWAS**) summary statistics[51]. Despite differences in cohorts (PsychAD vs. ROSMAP), cell clustering/taxonomy, TIM methods (PrediXcan vs. FUSION[8]), and sample sizes (920 vs. 424), we observed strong concordance (Pearson's $r_{(13,861)} = 0.78$, p-value = $5.71\times10^{-2,787}$) between z-score sets (Supplementary Fig. 26B). By simple counts, we observed 6,776 more imputable gene-cell-type combinations (PsychAD: 27,601; Zeng-2024: 20,825) and 31 more significant gene-cell-type associations (PsychAD: 335; Zeng-2024: 304) under the same FDR-adjusted p-value threshold, consistent with broader coverage and a higher discovery yield.

Second, to validate microglia-specific findings across different experimental designs, we compared our PsychAD microglia subclass snTIM (Subclass-MG; N = 904) with a smaller EUR TIM which we constructed with fluorescence activated cell sorting-isolated microglia (**FACS-MG**) data (N = 271)[5,18]. Although these models share only 22 donors and employ different expression-profiling methods, we observed strong per-gene consistency between R^2_{CV} and R^2_{out} estimates (Spearman's ρ = 0.55; N = 1,853, p-value = 1.67×10^{-149}; Supplementary Fig. 26C). Consequently, performing S-TWAS for AD (selected for its known microglial relevance[5,129]) with both TIMs yielded very strong z-score concordance (Pearson's $r_{(733)} = 0.87$, p-value = 1.32×10^{-231}; Supplementary Fig. 26D).

Supplementary Note 4. Cross-validated training performance across cell types and ancestries

Prediction performance across cell types: Relative to Bulk, snTIMs added 4,647 unique genes and provided superior prediction performance for >66% of shared genes (5,055 of 7,642; sign-test p-value = 1.19×10^{-178}). The largest performance gains were observed for Class and Subclass snTIMs across the full cross-validation R^2 (**R^2_{CV}**) spectrum (Fig. 1B).

Prediction performance across ancestries: As expected from differences in sample size, the number of imputable genes (Supplementary Fig. 1; Tables S4-9) and the explained variance of gene expression (R^2_{CV}) were highest in EUR, followed by AFR and AMR (Supplementary Fig. 2B). Despite lower power, AFR and AMR snTIMs reliably imputed an additional 882 and 335 genes, respectively, that were not imputable in EUR (Table S32-37).

Supplementary Note 5. Expanded analysis of snTIMs across ancestries

Linkage disequilibrium can inflate genomic correlation due to patterns of local correlation. To demonstrate the stability of gene imputability (R^2_{CV}) Spearman correlations across ancestries to linkage disequilibrium, we repeated the analysis using one random overlapping imputable gene per linkage disequilibrium block. We performed this analysis across all cell-types and using 10 iterations per cell-type. The median Spearman correlation across iterations (ρ = 0.52) was very similar to that reported in Fig. 1C (ρ = 0.54), indicating that the observed cross-ancestry pattern is not driven solely by clusters of correlated genes.

Our comparison of snTIMs across ancestries may underestimate the magnitude of the correlation due to power insufficiency; notably, we observed approximately half the number of shared coding gene-cell-type models in ancestry comparisons that included the least powered AMR snTIMss (mean Jaccard index is 0.12 and 0.16 for EUR-AMR and AFR-AMR, respectively) when contrasted against the EUR-AFR comparison (Jaccard index = 0.29) (Supplementary Fig. 2). Moreover, there are

fewer shared gene models at the subclass level (Supplementary Fig. 27) despite demonstrating similar correlation (Supplementary Fig. 3), most notably for the AFR and AMR snTIMs (Table S11).

Supplementary Note 6. Multi-cell-type fine-mapping is consistent with individual-cell-type fine-mapping

We find an increase in genes per locus when comparing class-aggregate to the individual classes (33.74%) (Wilcoxon rank-sum p-value = 9.01×10^{-9}). Notably, this increase is significant even when performing multi-cell-type fine-mapping (4.21%) (Wilcoxon rank-sum p-value = 3.03×10^{-3}; Supplementary Fig. 7, Supplementary Fig. 28).

Supplementary Note 7. Comparison of neuropsychiatric disorders (NPDs), neurodegenerative disorders (NDDs), and substance use disorders (SUDs)

Following our cross-disorder analysis, we investigated whether S-snTWAS correlation could identify differences in major classes of disorders. We classified 14 traits into NPDs, NDDs, and SUDs and analyzed trends of correlations. Overall, we found that the greatest similarity in associations is generally within each disorder category (e.g. one NPD compared with another NPD) (Supplementary Fig. 29A). Secondly, we found SUDs were more associated with NPDs and NDDs than NPDs were associated with NDDs. While this is mostly explained by genetic correlation of the underlying GWAS, the association between NDDs and SUDs is slightly stronger when comparing S-snTWAS as opposed to comparing GWAS (Supplementary Fig. 29B), even though GWAS correlation values and TWAS correlation values were themselves very correlated (Spearman's ρ = 0.79; p-value = 9.05×10^{-21}; Supplementary Fig. 29C). We found that this increase was largely explained by the association between alcohol use disorder (**AUD**) and NDDs (Table S40). Interestingly, while GWAS correlation between AUD and NDDs is generally low, we found significant enrichment when comparing AUD and Alzheimer's Disease (**AD**) (Fig. 4A; 62 gene-cell-type combinations in common; Fisher's exact test FDR-adjusted p-value = 6.63×10^{-50}). Finally, clustering analysis of TWAS correlations separates NPDs and NDDs, but SUDs are scattered throughout (Supplementary Fig. 15). Cannabis use disorder and tobacco use disorder cluster strongly with NPDs such as MDD, attention-deficit/hyperactivity disorder (**ADHD**), and insomnia, whereas AUD clusters more closely with NDDs such as AD, Parkinson's Disease (**PD**), and amyotrophic lateral sclerosis (**ALS**). This suggests that while NPDs and NDDs can be separated much more easily, SUDs cannot.

Supplementary Figures

Supplementary Fig. 1

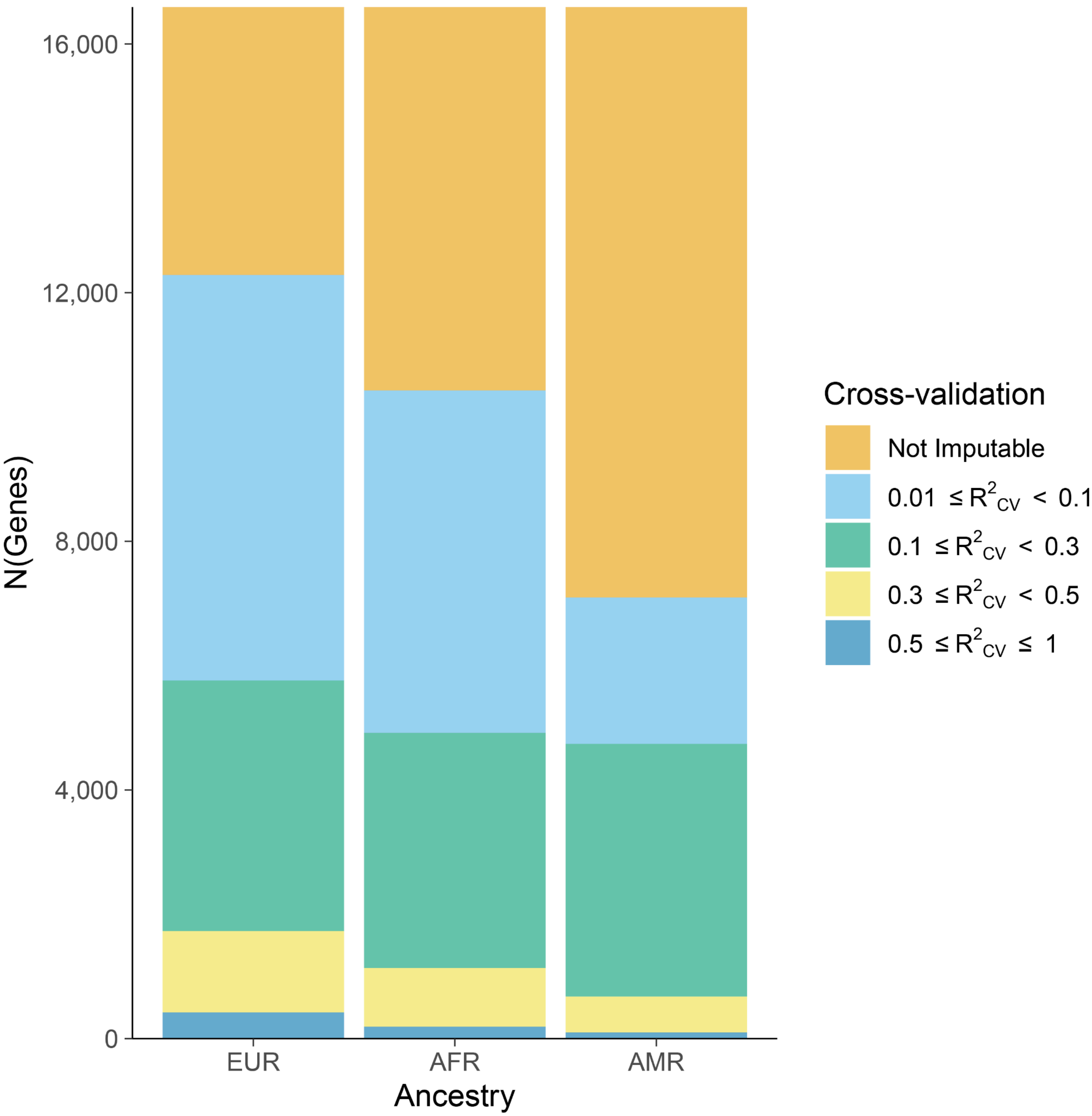

Supplementary Fig. 1 | Multi-ancestry single-nucleus transcriptomic imputation model (snTIM) training. The legend indicates gene imputability among snTIMs. The "Not Imputable" category comprises gene models with cross-validation R^2 (**R^2_{cv}**) < 0.01 or false discovery rate (**FDR**)-adjusted p-value$_{CV}$ > 0.05. The remaining categories correspond to imputable genes with progressively greater gene expression variance explained (R^2_{CV}) by single nucleotide polymorphisms (**SNP**s) in the model. The model for each gene with the greatest R^2_{CV} among all gene models (in snBulk-, Class-, and Subclass-level snTIMs) is visualized.

Supplementary Fig. 2

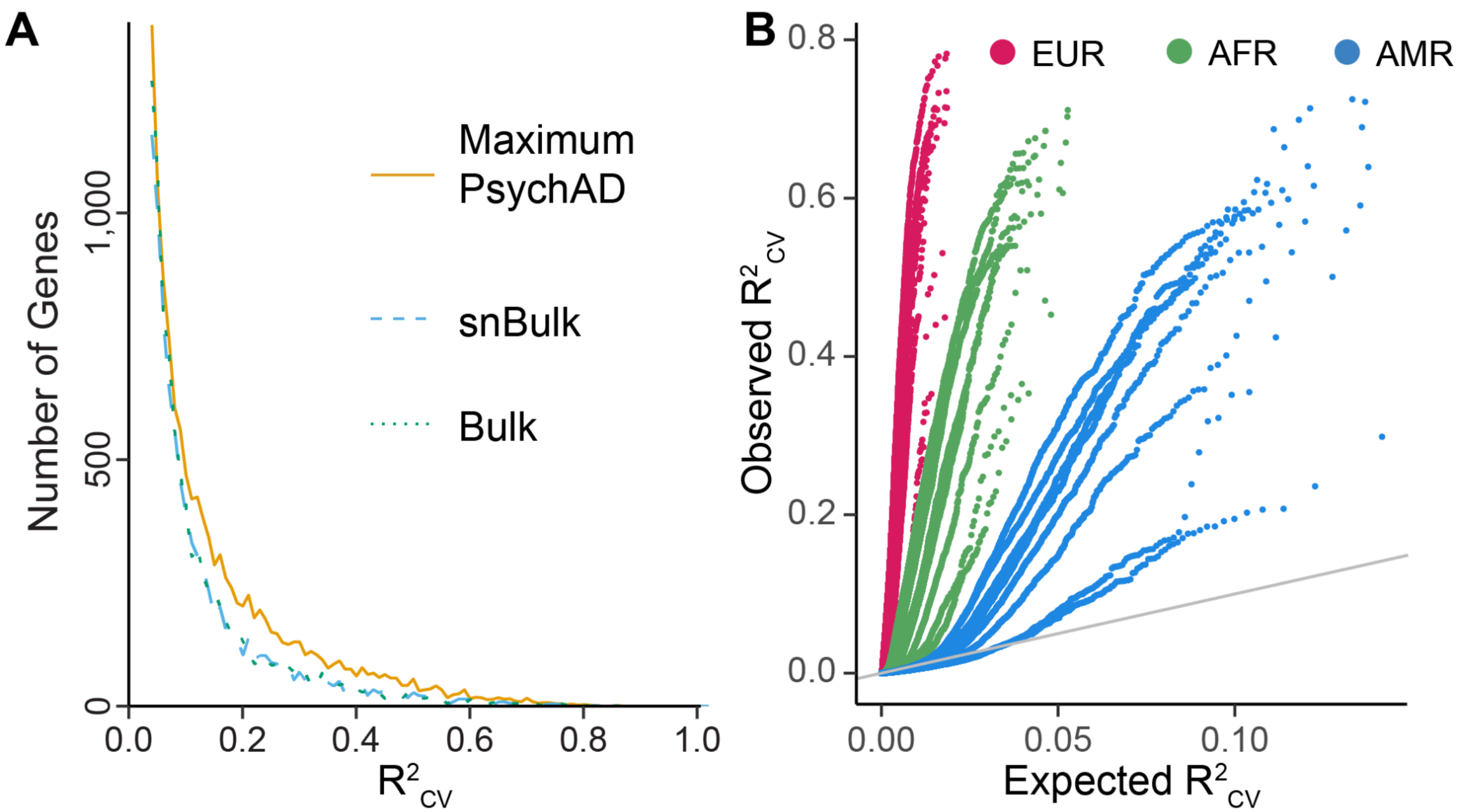

Supplementary Fig. 2 | Cross-Validation R^2 (R^2_{cv}) across cell-types and ancestries. A, Distribution of R^2_{cv} among EUR single-nucleus transcriptomic imputation models (**snTIM**s). Maximum PsychAD refers to the distribution of the maximum R^2_{CV} per gene across all EUR snTIMs (snBulk, Class and Subclass). **B,** Power comparison of snTIMs across ancestries. Quantile-quantile plot of the observed against expected performance (R^2_{CV}) for every class-level model across 3 genetic ancestries. EUR: European; AFR: African; AMR: Admixed-American. Null distribution (gray line) represents the hypothesis that gene expression is not genetically regulated.

Supplementary Fig. 3

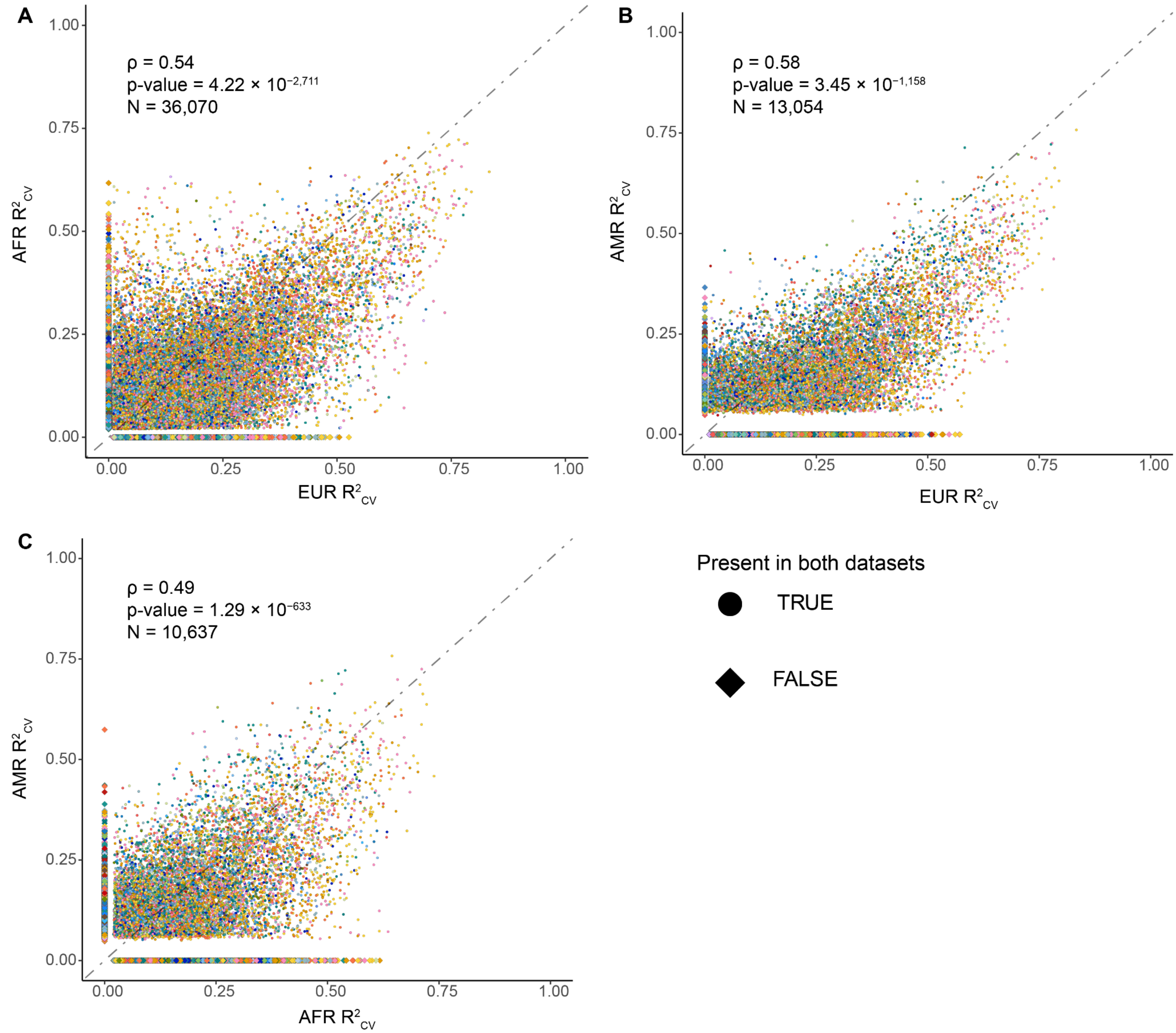

Supplementary Fig. 3 | Comparison of gene-cell-type prediction performance across ancestry-specific single-nucleus transcriptomic imputation models (snTIMs). The color of each point represents the cell-type (see Extended Data Fig. 2). Comparisons are for individuals of European ancestry (**EUR**) vs. individuals of African ancestry (**AFR**) (**A**), EUR vs. individuals of Admixed American ancestry (**AMR**) (**B**) and AFR vs. AMR (**C**) snTIMs. EUR-AMR: ρ = 0.58, p-value = $3.45 \times 10^{-1,158}$, N = 13,054; EUR-AFR: ρ = 0.54, p-value = $4.22 \times 10^{-2,711}$, N = 36,070; AFR-AMR: ρ = 0.49, p-value = 1.29×10^{-633}, N = 10,637; Spearman's correlation analysis. Similar plots for out-of-sample validation for EUR snTIMs are performed in Supplementary Fig. 27. **A.** 2,796 genes are imputed in EUR and not AFR snTIMs, and vice versa for 882 genes. **B.** 7,081 genes are imputed in EUR and not AMR snTIMs, and vice versa for 335 genes. **C.** 5,560 genes are imputed in AFR and not AMR snTIMs, and vice versa for 728 genes.

Supplementary Fig. 4

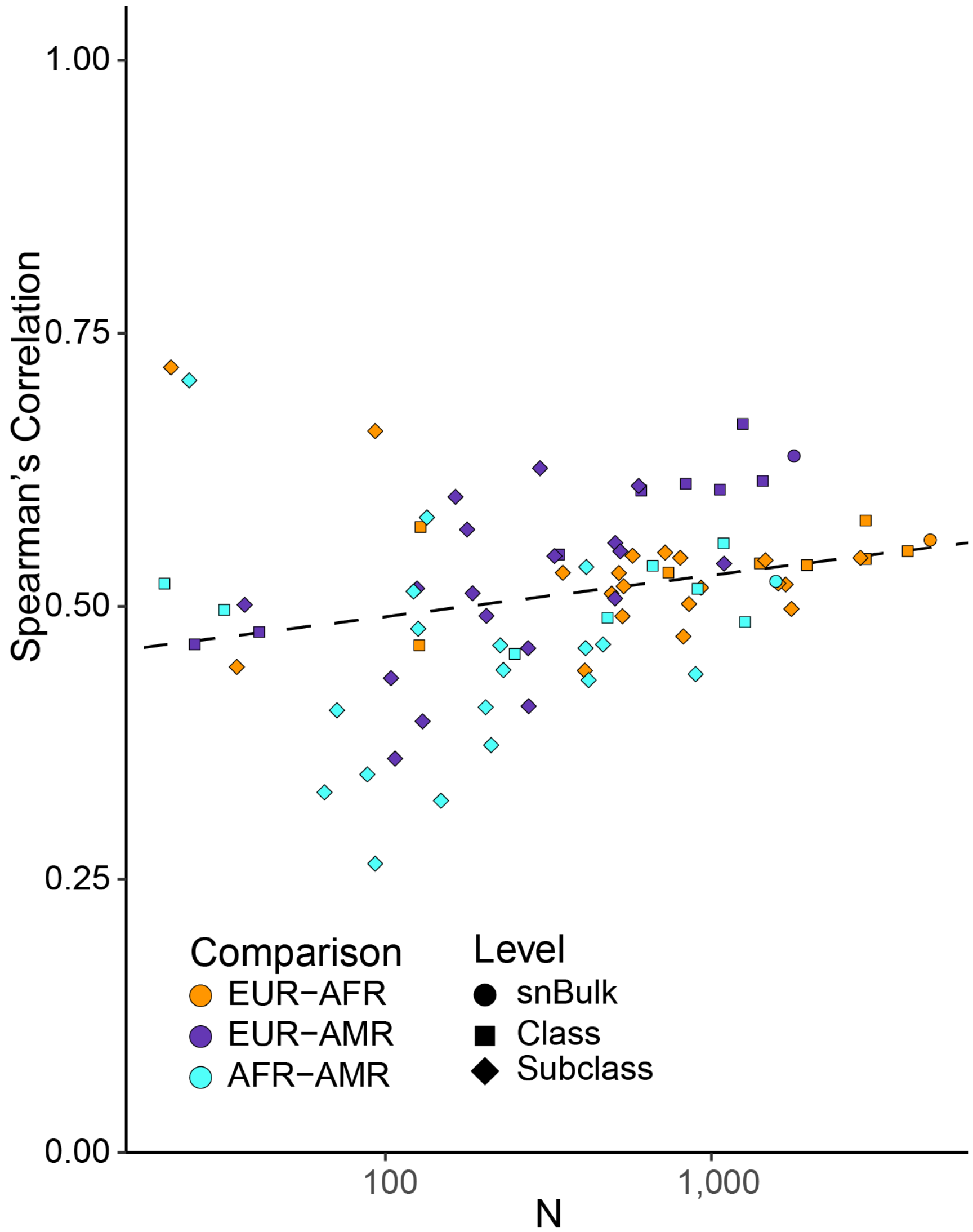

Supplementary Fig. 4 | Spearman's correlation of cross-ancestry prediction performance at different single-nucleus transcriptomic imputation model (snTIM) levels. Each point represents a pairwise analysis of cross-validation R^2 ($\mathbf{R^2_{cv}}$) across all shared imputable genes ("N") between ancestries for the same snTIM. Applicable pairwise ancestry comparisons include individuals of European ancestry (**EUR**)-individuals of African ancestry (**AFR**), EUR-individuals of Admixed American ancestry (**AMR**) and AFR-AMR colored orange, purple and light-blue, respectively. Level indicates the cell-type hierarchy of the compared cell-type across ancestries (Extended Data Fig. 2). Point shape denotes snTIM level including snBulk, Class and Subclass. The dashed line represents the linear regression line (Spearman's Correlation ~ $\log_{10}$(N)). $\log_{10}$(N) is positively associated (p-value = 0.018) with Spearman's correlation in the linear regression analysis (p-value = 0.018, adjusted R^2 = 0.06).

Supplementary Fig. 5

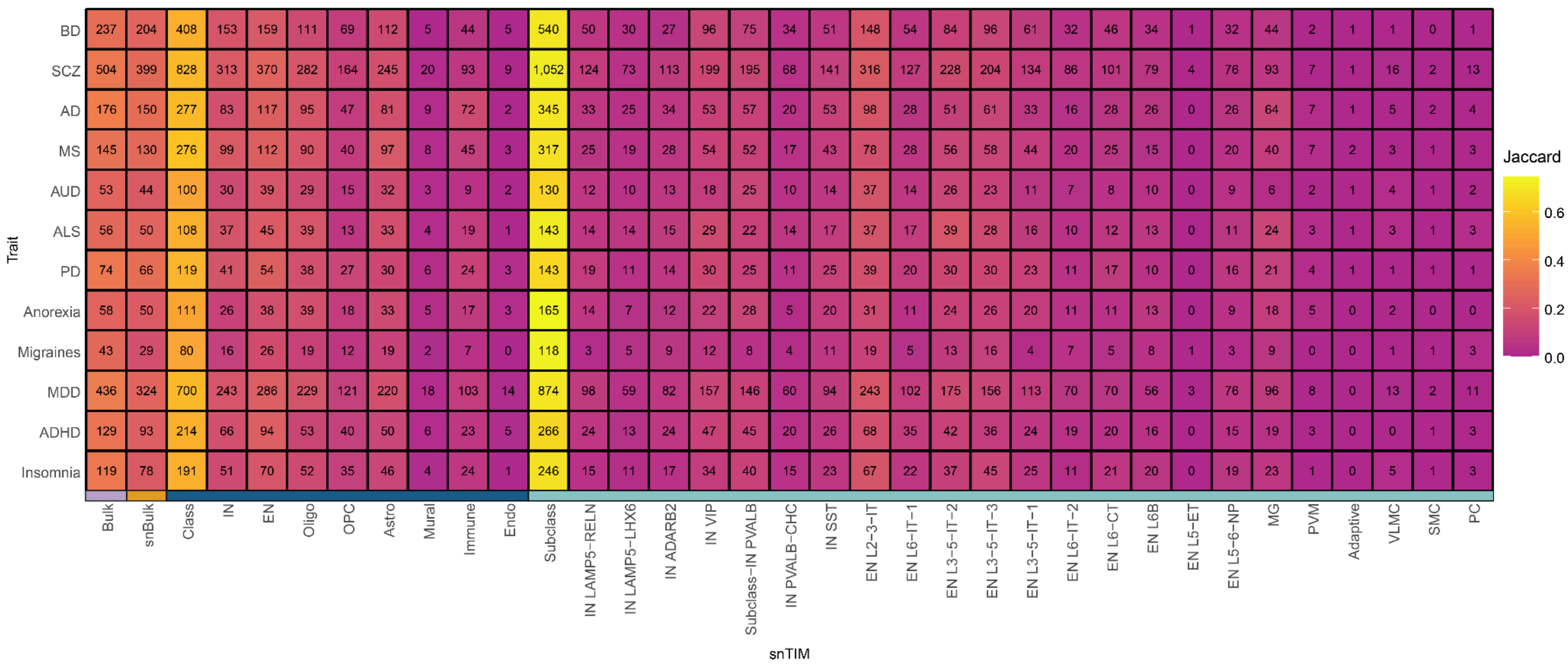

Supplementary Fig. 5 | Expanded heatmap of summary transcriptome-wide association study (S-TWAS) significant gene-trait associations (GTAs) by cell-type hierarchy level. The number within each cell represents the number of false discovery rate (**FDR**)-significant GTAs within each single-nucleus transcriptomic imputation model (**snTIM**) and level aggregate. Cell color denotes the trait-specific Jaccard index, defined as the fraction of significant GTAs at that level (*x*) that overlap the union of significant GTAs across all levels for the same trait ($\frac{|x \cap all|}{|x \cup all|}$). FDR-adjustment for class and subclass aggregate categories is performed across all participating models for that hierarchy level. Color denotes the Jaccard index ($\frac{|x \cap all|}{|x \cup all|}$) of the specific cell (*x*) against the union of all FDR-significant trait-associated genes across Bulk, snBulk, class and subclass for that trait. S-TWAS analysis is limited to individuals of European ancestry (**EUR**). Full names of disorders are described in Table S12. Full names of all cell-types are defined in Table S3.

Supplementary Fig. 6

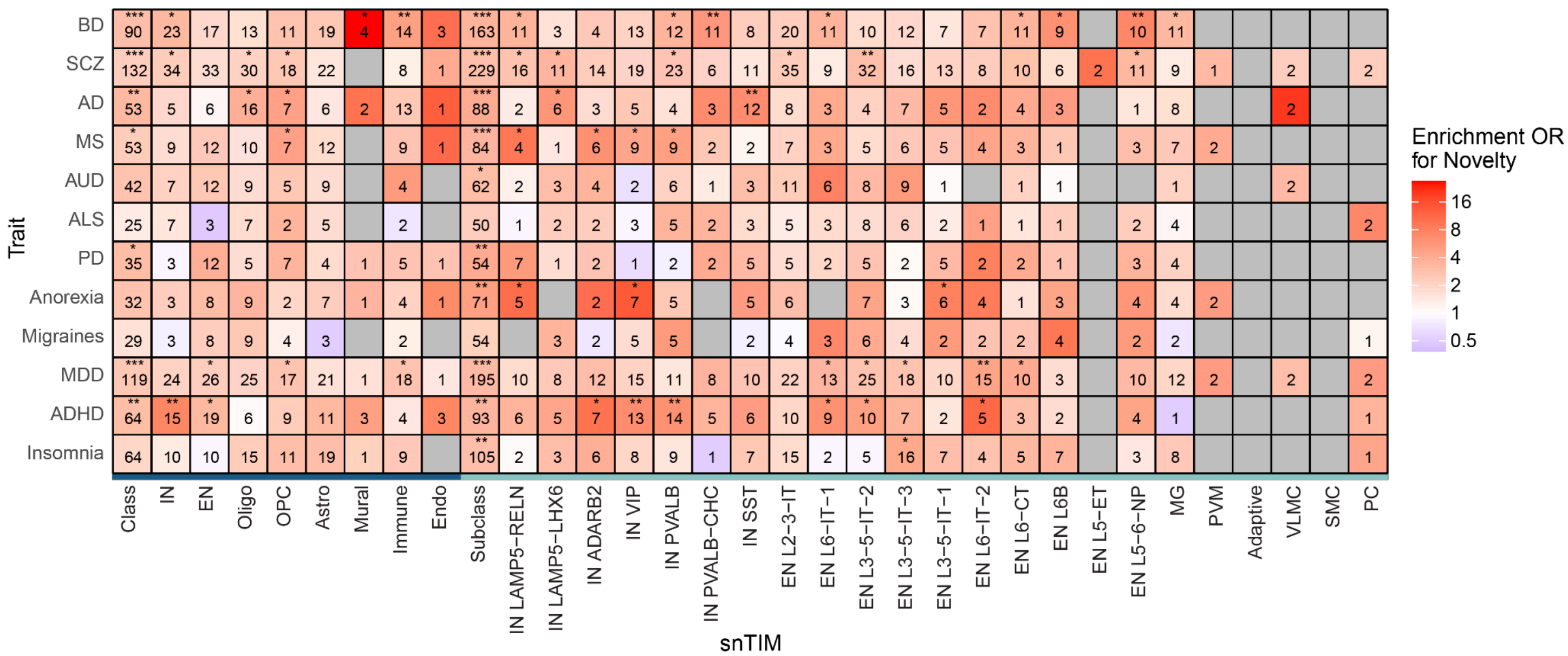

Supplementary Fig. 6 | Heatmap of novel gene-trait associations (GTAs). The number in each cell indicates the number of novel GTAs (as in Fig. 2C) found in each single-nucleus transcriptomic imputation model (**snTIM**) and level aggregates. Color of each cell indicates the effect size of the Fisher's exact test comparing each snTIM to snBulk. We performed false discovery rate (**FDR**) adjustment across all presented traits and cell-types and aggregates. Asterisks indicate significance (* FDR-adjusted p-value ≤ 0.05, ** FDR-adjusted p-value ≤ 0.01, *** FDR-adjusted p-value ≤ 0.001). Full names of disorders are described in Table S12. Full names of all cell-types are defined in Table S3.

Supplementary Fig. 7

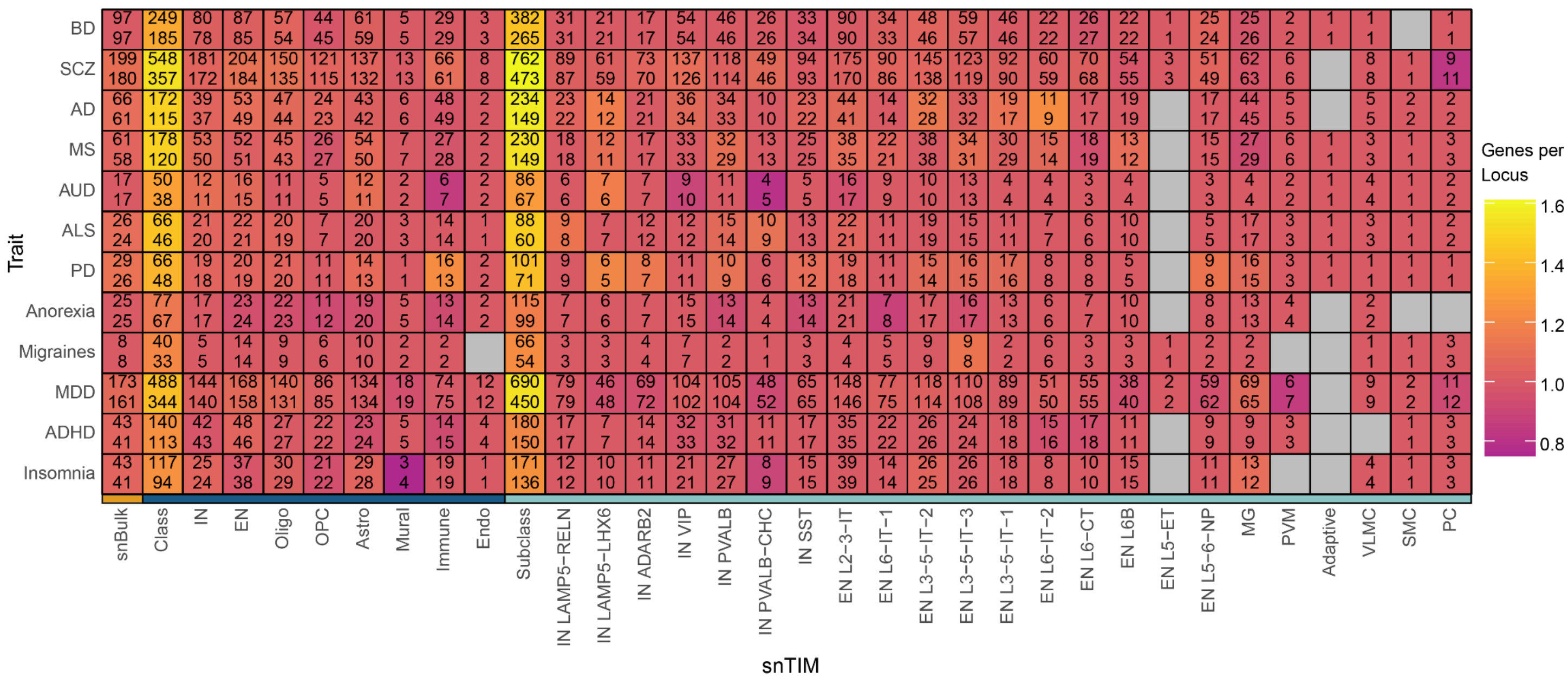

Supplementary Fig. 7 | Expanded heatmap of summary-level single-nucleus transcriptome-wide association study (S-snTWAS) individual-cell-type gene fine-mapping. The numbers at the top and bottom of each cell indicate the count of fine-mapped genes and loci (posterior inclusion probability (**PIP**) ≥ 0.5), respectively. “Genes per Locus” indicates the ratio of these numbers. Color indicates the genes per locus for every single-nucleus transcriptomic imputation model (**snTIM**)-trait combination. Fine-mapping was performed with fine-mapping of causal gene sets (**FOCUS**) for each cell-type. FOCUS performs analyses at the linkage disequilibrium (**LD**) block level rather than the standard 1 mega base pair window from the transcription start site which means that dysregulated genetically regulated gene expression (**GReX**) for a given gene-trait combination may be driven by more than one locus. In our cell-type-specific snTWAS FOCUS analysis, in most loci one gene was fine-mapped (PIP ≥ 0.5), with the exception of a few loci prioritizing more than one gene. Interestingly, in a few instances (e.g. major depressive disorder (**MDD**) Subclass-perivascular macrophage (**PVM**)), a gene was fine-mapped in two different LD blocks. Full names of disorders are described in Table S12. Full names of all cell-types are defined in Table S3.

Supplementary Fig. 8

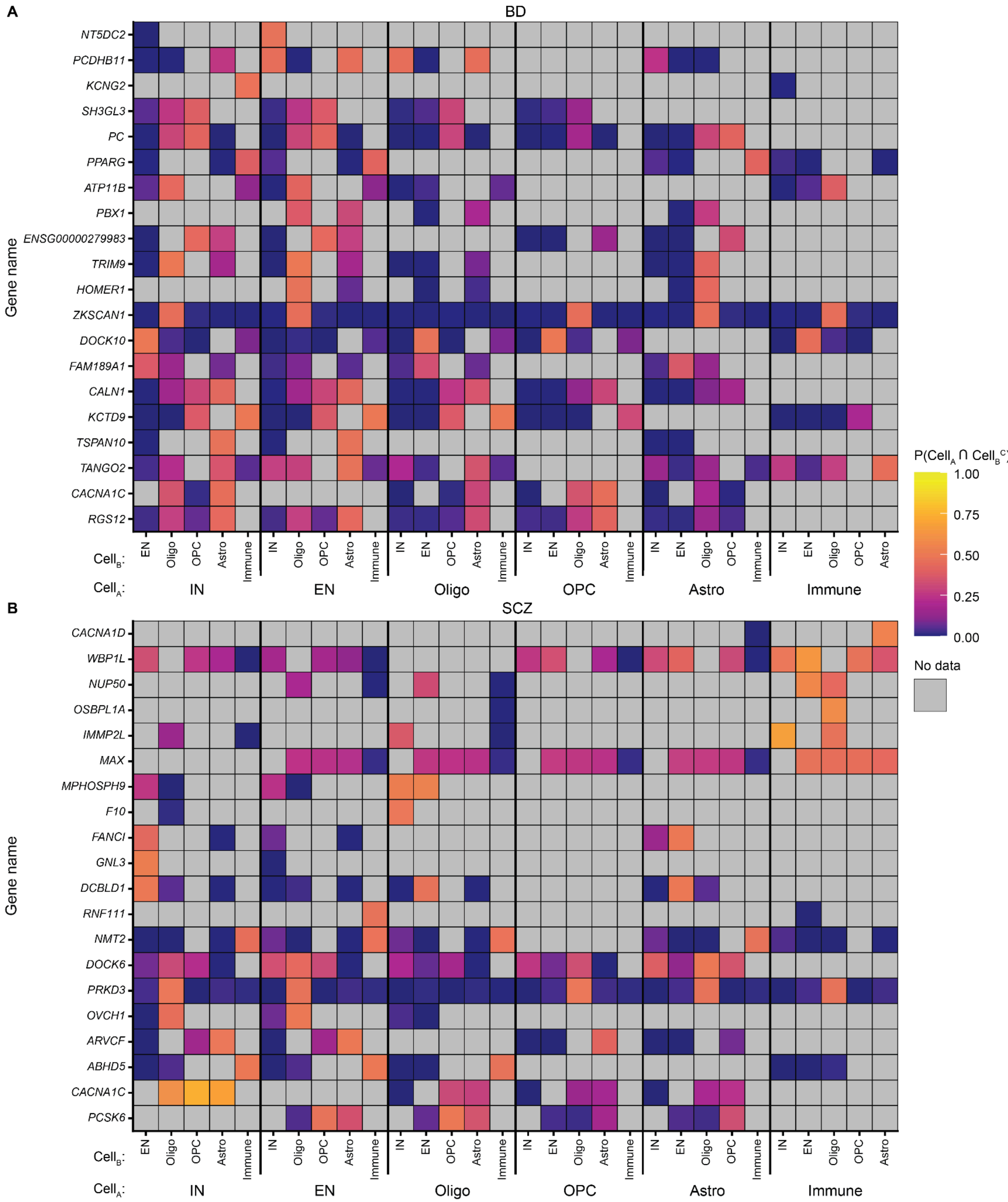

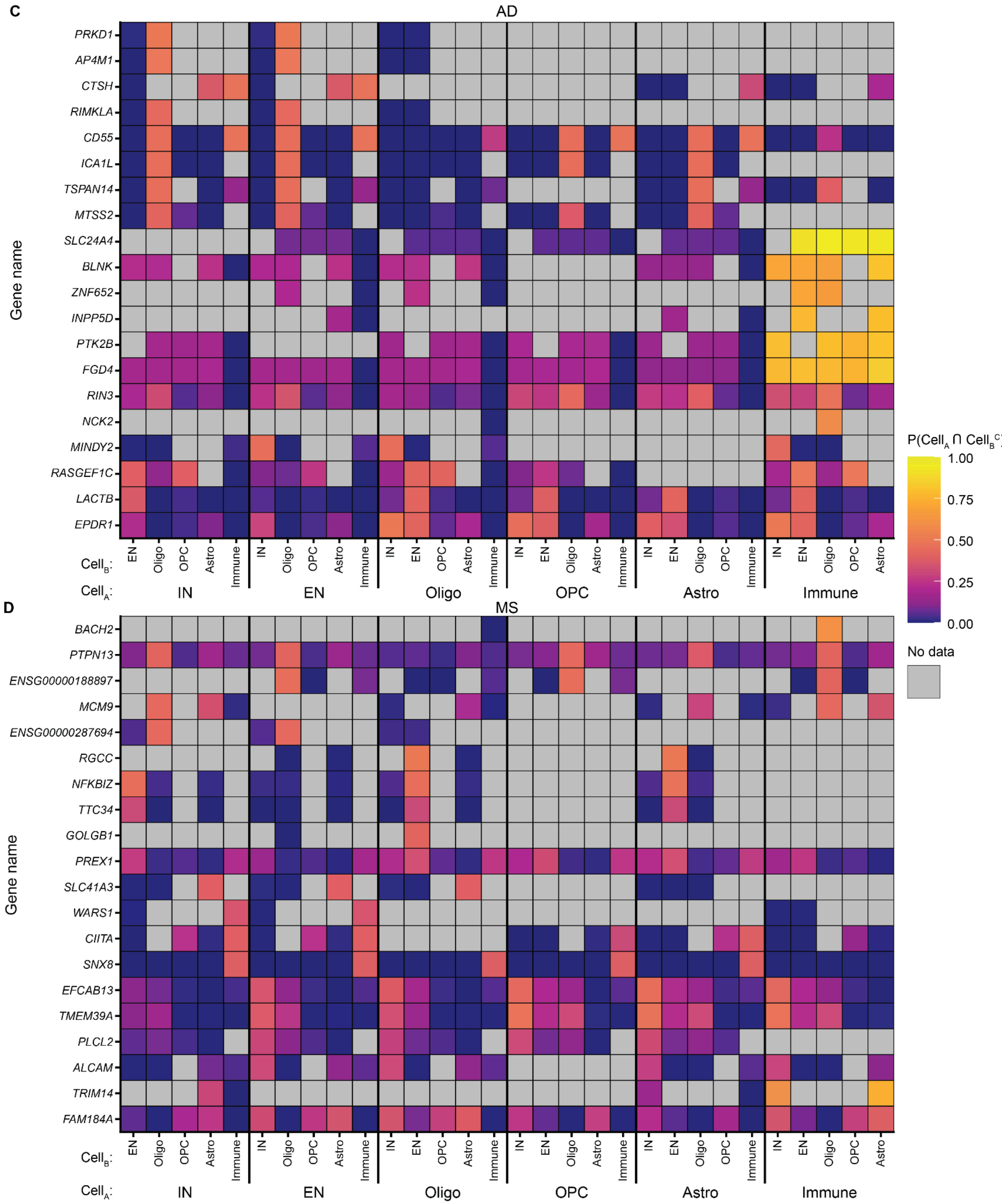
C
AD
Gene name
PRKD1
AP4M1
CTSH
RIMKLA
CD55
ICA1L
TSPAN14
MTSS2
SLC24A4
BLNK
ZNF652
INPP5D
PTK2B
FGD4
RIN3
NCK2
MINDY2
RASGEF1C
LACTB
EPDR1
$Cell_B$:
$Cell_A$:
IN
EN
Oligo
OPC
Astro
Immune
$P(Cell_A \cap Cell_B^C)$
1.00
0.75
0.50
0.25
0.00
No data
D
MS
BACH2
PTPN13
ENSG00000188897
MCM9
ENSG00000287694
RGCC
NFKBIZ
TTC34
GOLGB1
PREX1
SLC41A3
WARS1
CIITA
SNX8
EFCAB13
TMEM39A
PLCL2
ALCAM
TRIM14
FAM184A

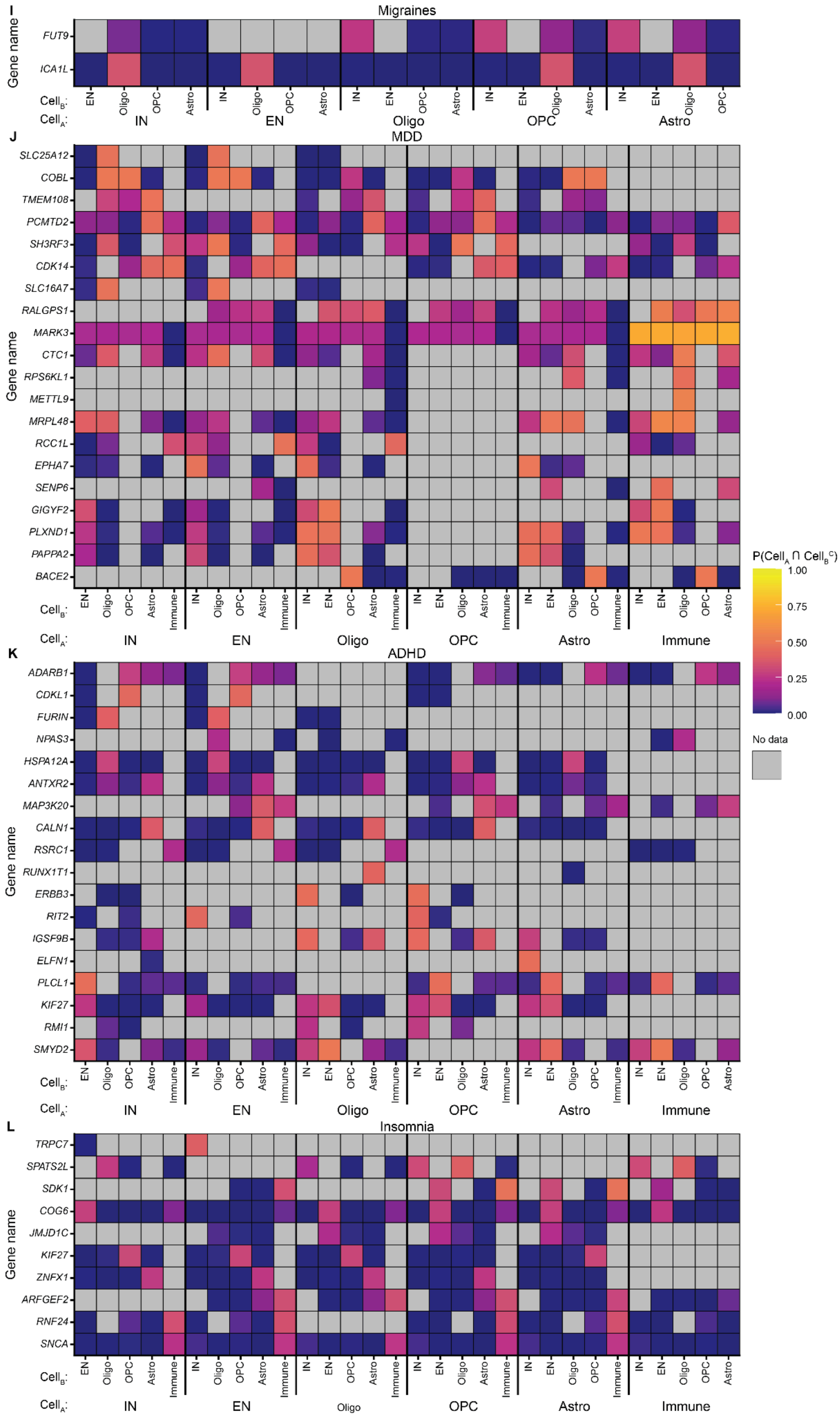
I
Migraines
Gene name
FUT9
ICA1L
Cell_B:
EN
Oligo
OPC
Astro
IN
Cell_A:
IN
EN
Oligo
OPC
Astro
J
MDD
SLC25A12
COBL
TMEM108
PCMTD2
SH3RF3
CDK14
SLC16A7
RALGPS1
MARK3
CTC1
RPS6KL1
METTL9
MRPL48
RCC1L
EPHA7
SENP6
GIGYF2
PLXND1
PAPPA2
BACE2
Immune
K
ADHD
ADARB1
CDKL1
FURIN
NPAS3
HSPA12A
ANTXR2
MAP3K20
CALN1
RSRC1
RUNX1T1
ERBB3
RIT2
IGSF9B
ELFN1
PLCL1
KIF27
RMI1
SMYD2
L
Insomnia
TRPC7
SPATS2L
SDK1
COG6
JMJD1C
KIF27
ZNFX1
ARFGEF2
RNF24
SNCA
P(Cell_A ∩ Cell_B^C)
1.00
0.75
0.50
0.25
0.00
No data

Supplementary Fig. 8 | Multivariate adaptive shrinkage (mash) analysis in summary-level single-nucleus transcriptome-wide association study (S-snTWAS) traits. Heatmaps depicting the combinatorial probability that an effect exists in cell-type A and not in cell-type B at the class level ("$P(Cell_A \cap Cell_B^C)$"). Only results with a $P(Cell_A \cap Cell_B^C) \geq 0.2$ in at least one comparison were retained for visualization. The top 20 results (ordered by the maximum row-wise combinatorial probability) are displayed for bipolar disorder (**BD**) (**A**), schizophrenia (**SCZ**) (**B**), Alzheimer's disease (**AD**) (**C**), multiple sclerosis (**MS**) (**D**), alcohol use disorder (**AUD**) (**E**), amyotrophic lateral sclerosis (**ALS**) (**F**), Parkinson's disease (**PD**) (**G**), Anorexia (**H**), Migraines (**I**), major depressive disorder (**MDD**) (**J**), attention-deficit/hyperactivity disorder (**ADHD**) (**K**), Insomnia (**L**). Cells are colored gray when the analysis is not applicable. Full names of all cell-types are defined in Table S3.

Supplementary Fig. 9

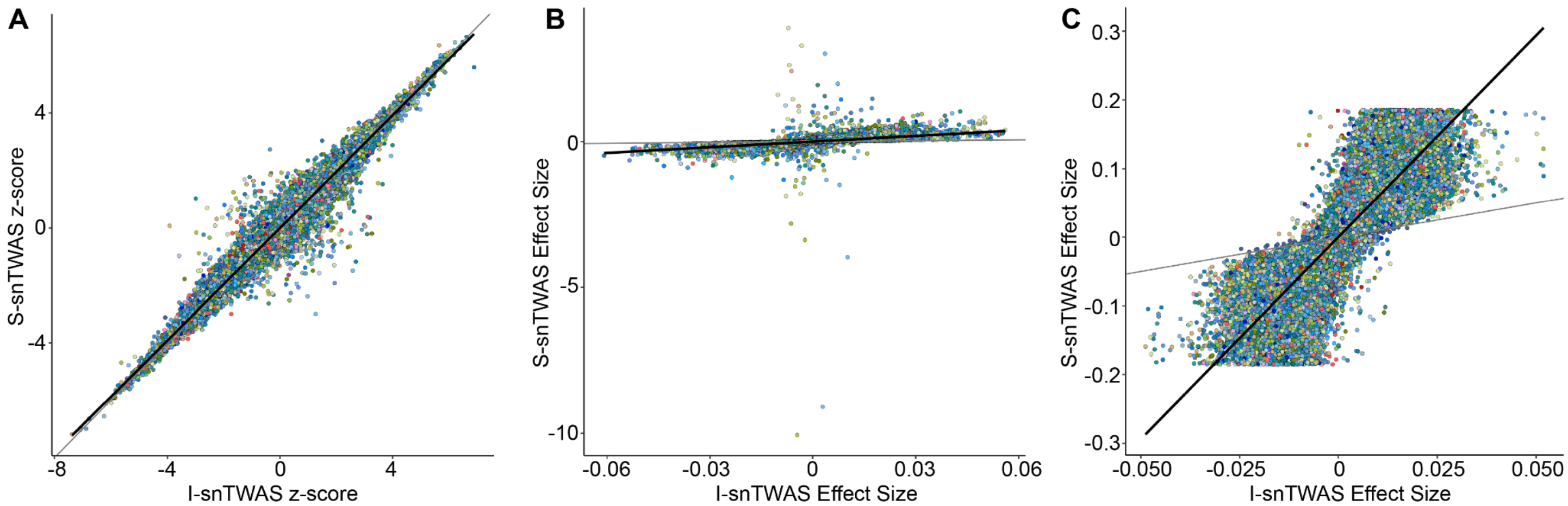

Supplementary Fig. 9 | Z-score and effect size comparisons between summary-level single-nucleus transcriptome-wide association study (S-snTWAS) and individual-level snTWAS (I-snTWAS). **A,** Z-score agreement across six overlapping traits (major depressive disorder (**MDD**) excluded due to sample overlap). For S-snTWAS, genome-wide association studies (**GWAS**s) were run in Million Veteran Program (**MVP**) using the same outcome and covariates as the individual-level analysis, and Summary-PrediXcan (**S-PrediXcan**) was applied. Pearson's correlation is 0.99 (p-value: $1.82 \times 10^{-583,345}$). The gray line represents y=x. **B,** Effect-size snTWAS agreement across the same six traits. Pearson's correlation is 0.77 (p-value = $2.87 \times 10^{-130,577}$). **C,** As in **B**, but zoomed after removing the top 1% of S-snTWAS effects by absolute value to emphasize the bulk of the distribution. The gray line represents y=x.

Supplementary Fig. 10

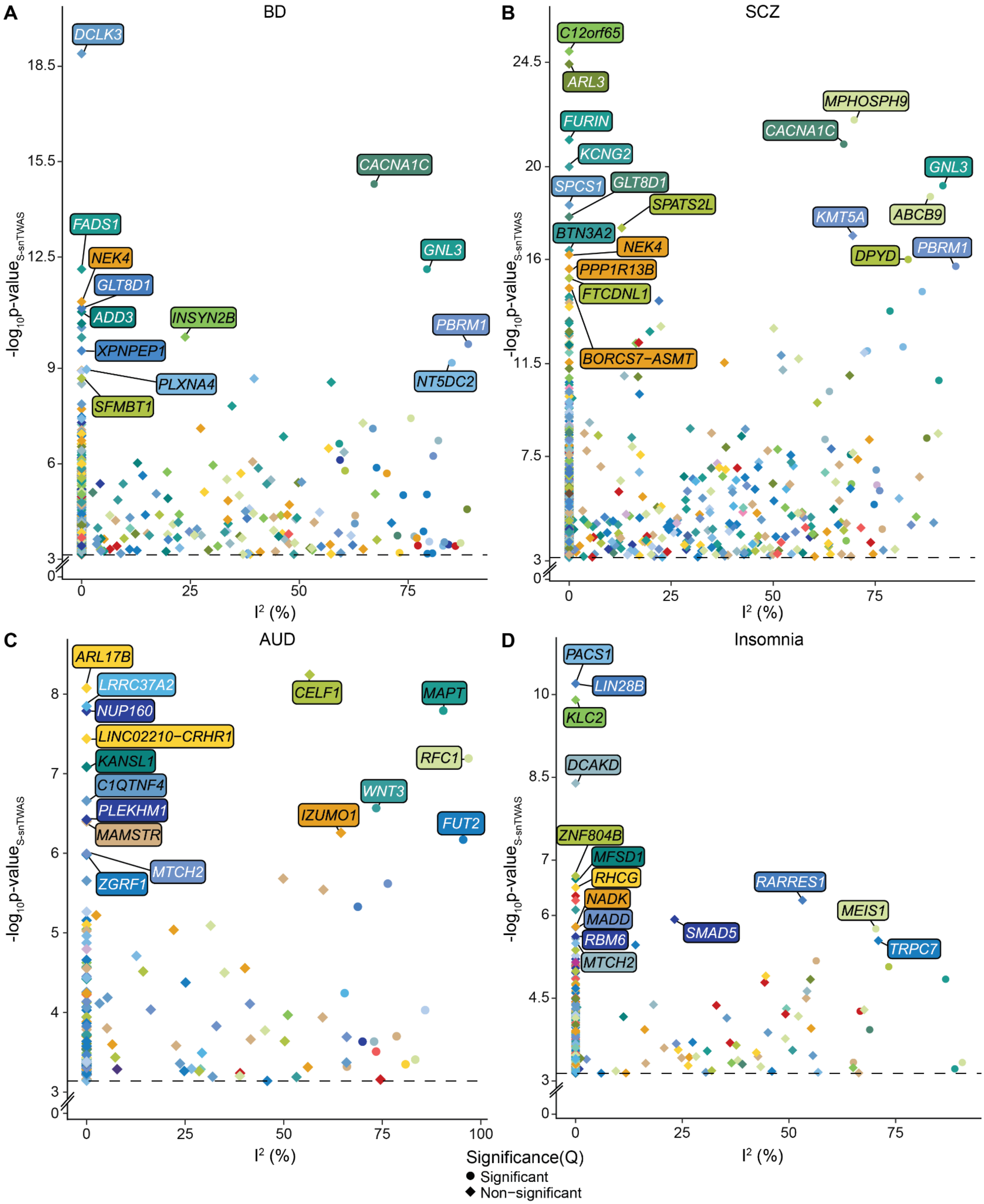

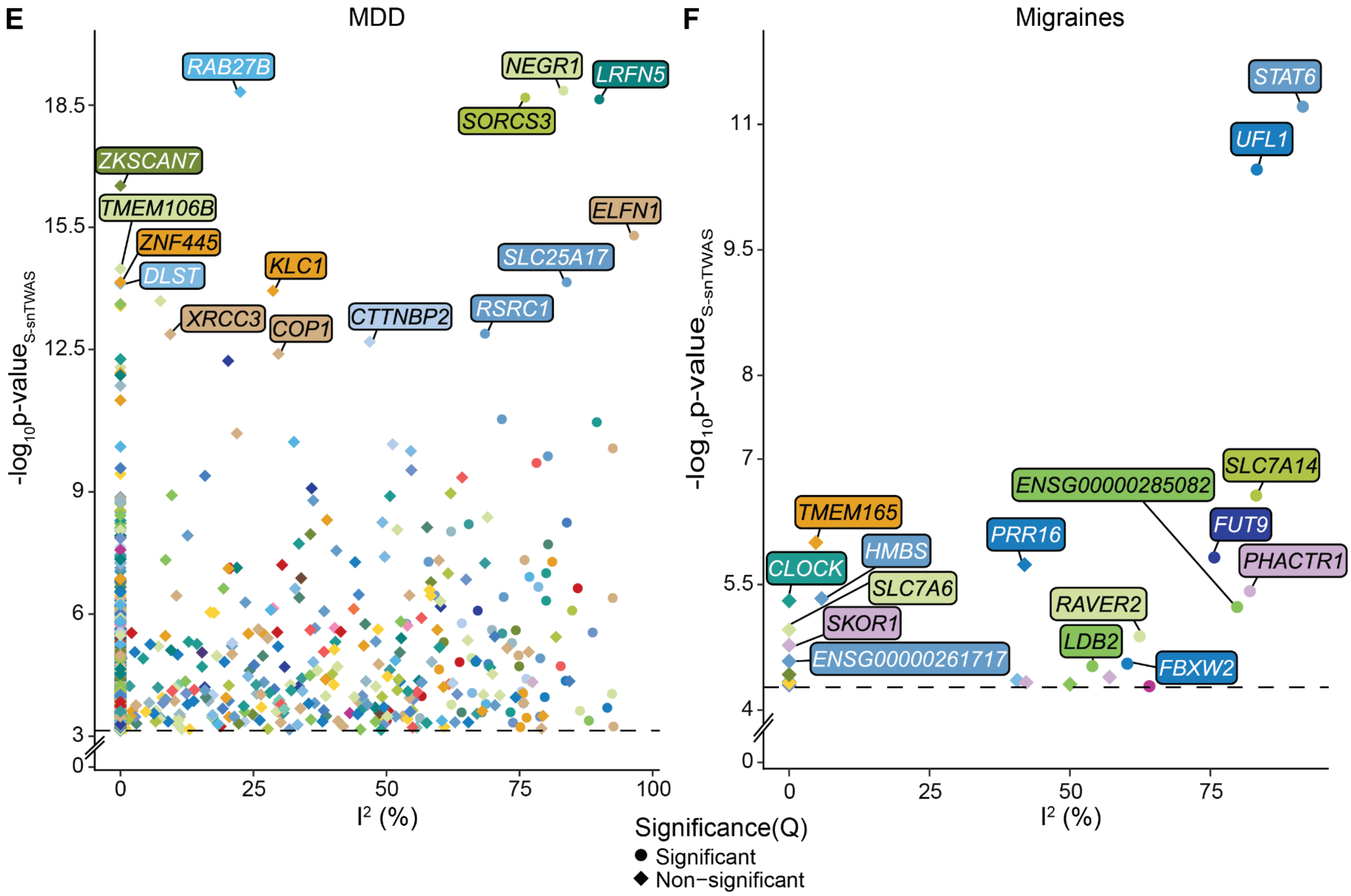

Supplementary Fig. 10 | Gene-trait association (GTA) effect size heterogeneity scatterplots. Gene association effect size heterogeneity (I^2) across cell-types for bipolar disorder (**BD**) (**A**), schizophrenia (**SCZ**) (**B**), alcohol use disorder (**AUD**) (**C**), Insomnia (**D**), major depressive disorder (**MDD**) (**E**), Migraines (**F**). Significance denotes the presence of different effects of individual cell-types in the analysis (Cochran's Q test). Only genes with significant GTAs in summary-level single-nucleus transcriptome-wide association study (**S-snTWAS**) analysis are visualized. Color corresponds to cell-type (Extended Data Fig. 2). Full names of all cell-types are defined in Table S3. Only traits with a high enough sample size in the Million Veteran Program (**MVP**) (see Methods) were evaluated for heterogeneity, due to need for effect size estimates. Alzheimer's disease (**AD**) is visualized in Fig. 3C. attention-deficit/hyperactivity disorder (**ADHD**), amyotrophic lateral sclerosis (**ALS**), Anorexia, multiple sclerosis (**MS**), and Parkinson's disease (**PD**) are excluded due to few cases in the MVP. Post-traumatic stress disorder (**PTSD**) and anxiety are excluded due to few significant S-snTWAS associations.

Supplementary Fig. 11

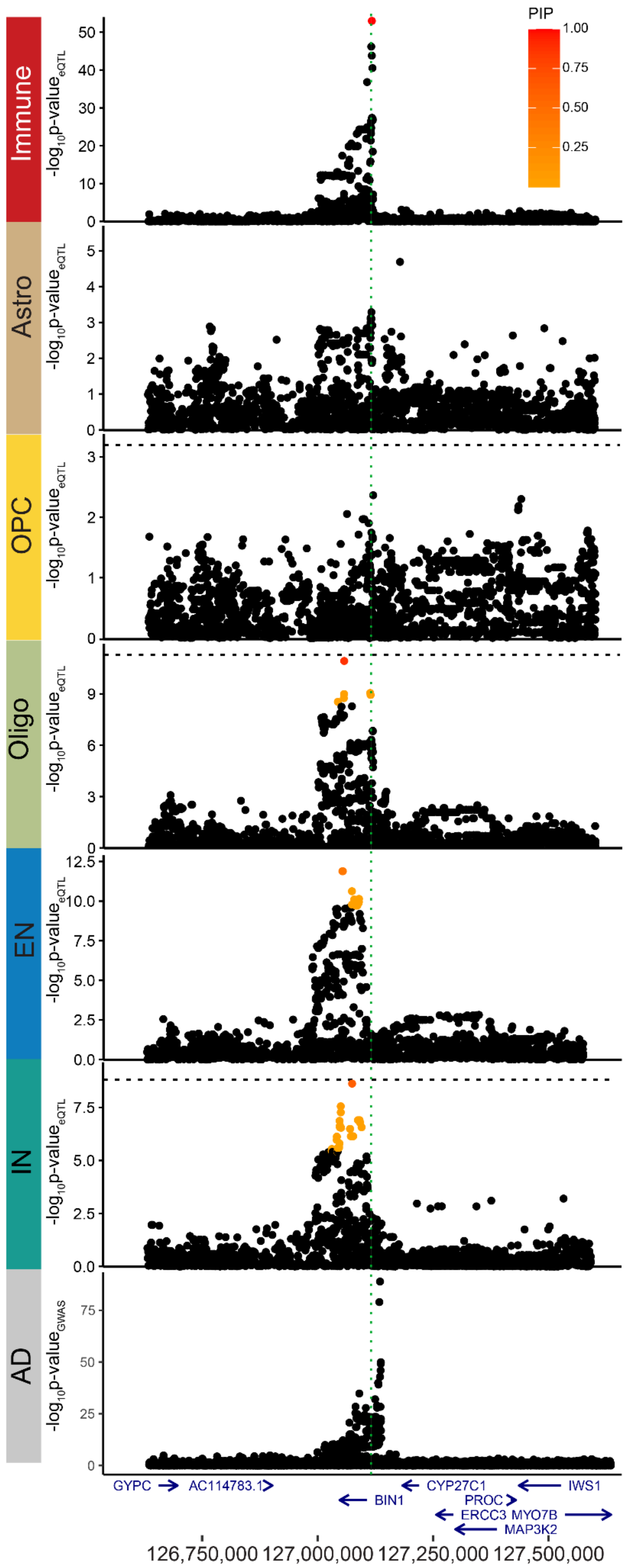

Supplementary Fig. 11 | Single-nucleus expression quantitative trait loci (Sn-eQTL) manhattan plot for *BIN1*. Color indicates posterior inclusion probability from statistical fine-mapping. Presence of horizontal dotted line indicates top variants were below significance threshold. The green dotted line shows the position of the fine-mapped (posterior inclusion probability (**PIP**) ≥ 0.5) index single nucleotide polymorphism (**SNP**) in Class-Immune. The top SNP for Class-Immune is rs6733839. Linkage disequilibrium for every eQTL SNP is indicated in Supplementary Fig. 12. Full names of all cell-types are defined in Table S3.

Supplementary Fig. 12

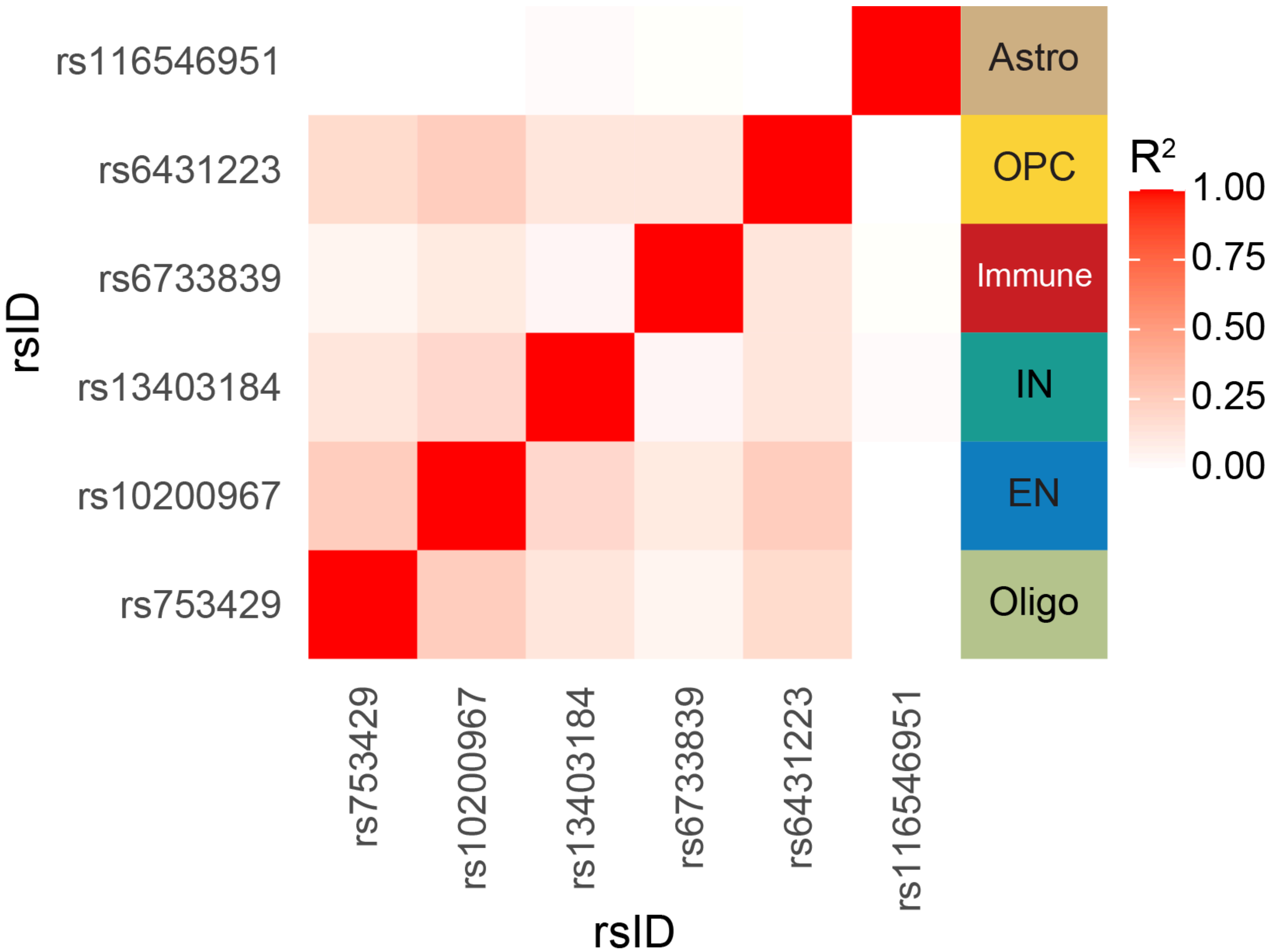

Supplementary Fig. 12 | Linkage disequilibrium heatmap for top expression quantitative trait loci (eQTL) single nucleotide polymorphisms (SNPs) of *BIN1* per cell-type. The R^2 between these SNPs is queried from publicly available information[31].

Supplementary Fig. 13

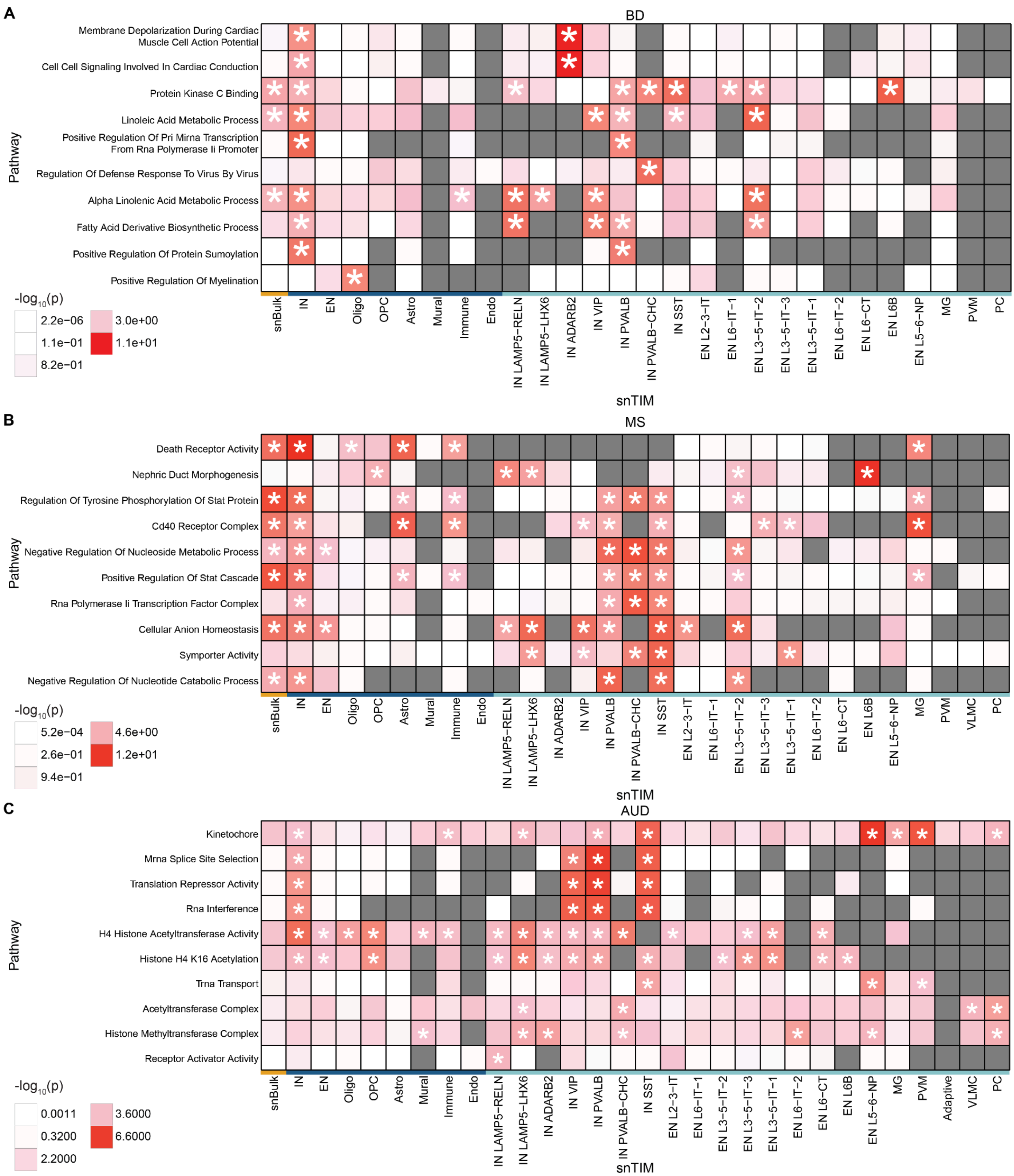

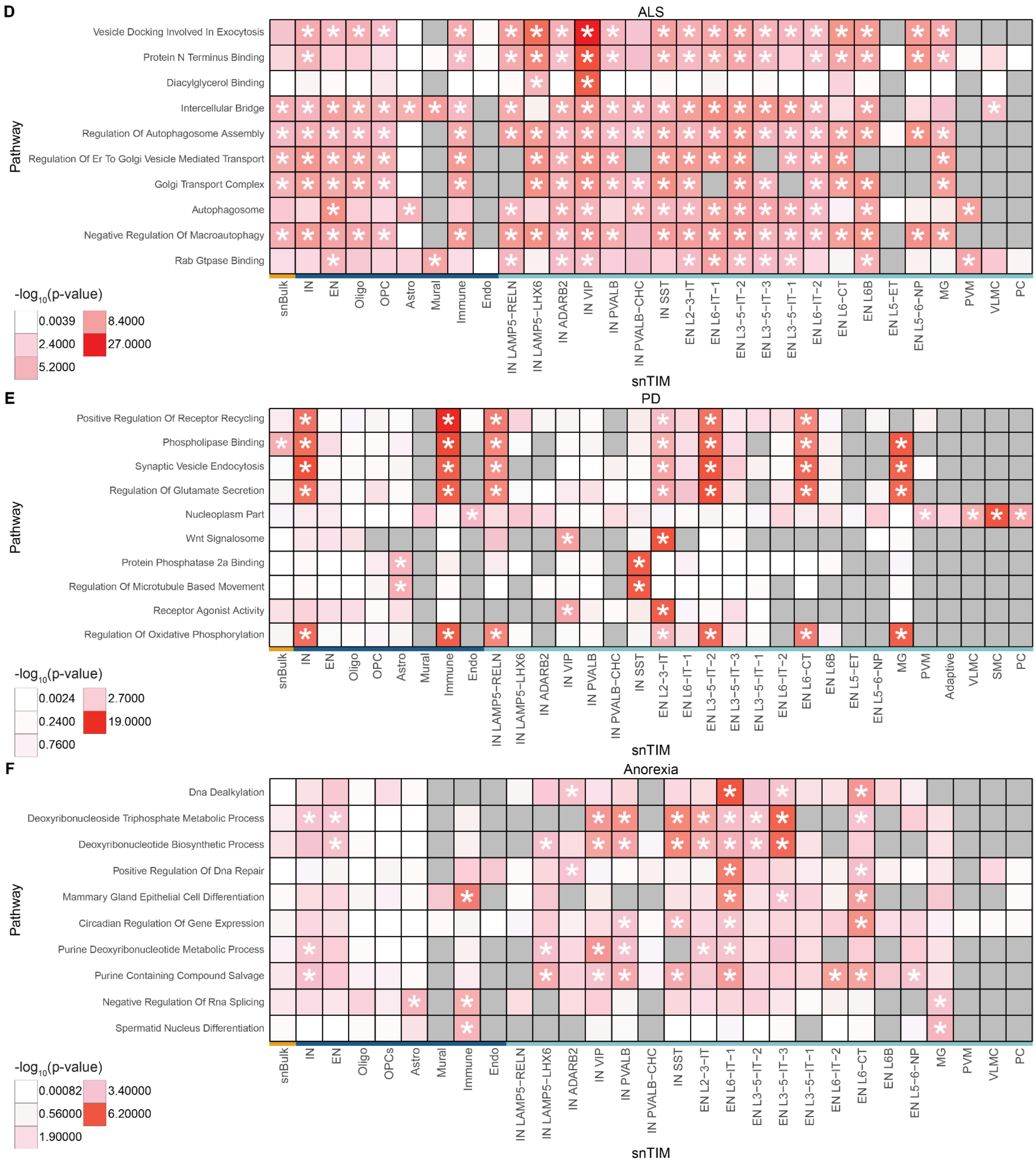
D
ALS
Pathway
Vesicle Docking Involved In Exocytosis
Protein N Terminus Binding
Diacylglycerol Binding
Intercellular Bridge
Regulation Of Autophagosome Assembly
Regulation Of Er To Golgi Vesicle Mediated Transport
Golgi Transport Complex
Autophagosome
Negative Regulation Of Macroautophagy
Rab Gtpase Binding
-log10(p-value)
0.0039
2.4000
5.2000
8.4000
27.0000
snBulk
IN
EN
Oligo
OPC
Astro
Mural
Immune
Endo
IN LAMP5−RELN
IN LAMP5−LHX6
IN ADARB2
IN VIP
IN PVALB
IN PVALB−CHC
IN SST
EN L2−3−IT
EN L6−IT−1
EN L3−5−IT−2
EN L3−5−IT−3
EN L3−5−IT−1
EN L6−IT−2
EN L6−CT
EN L6B
EN L5−ET
EN L5−6−NP
MG
PVM
VLMC
PC
snTIM
E
PD
Pathway
Positive Regulation Of Receptor Recycling
Phospholipase Binding
Synaptic Vesicle Endocytosis
Regulation Of Glutamate Secretion
Nucleoplasm Part
Wnt Signalosome
Protein Phosphatase 2a Binding
Regulation Of Microtubule Based Movement
Receptor Agonist Activity
Regulation Of Oxidative Phosphorylation
-log10(p-value)
0.0024
0.2400
0.7600
2.7000
19.0000
snBulk
IN
EN
Oligo
OPC
Astro
Mural
Immune
Endo
IN LAMP5−RELN
IN LAMP5−LHX6
IN ADARB2
IN VIP
IN PVALB
IN PVALB−CHC
IN SST
EN L2−3−IT
EN L6−IT−1
EN L3−5−IT−2
EN L3−5−IT−3
EN L3−5−IT−1
EN L6−IT−2
EN L6−CT
EN L6B
EN L5−ET
EN L5−6−NP
MG
PVM
Adaptive
VLMC
SMC
PC
snTIM
F
Anorexia
Pathway
Dna Dealkylation
Deoxyribonucleoside Triphosphate Metabolic Process
Deoxyribonucleotide Biosynthetic Process
Positive Regulation Of Dna Repair
Mammary Gland Epithelial Cell Differentiation
Circadian Regulation Of Gene Expression
Purine Deoxyribonucleotide Metabolic Process
Purine Containing Compound Salvage
Negative Regulation Of Rna Splicing
Spermatid Nucleus Differentiation
-log10(p-value)
0.00082
0.56000
1.90000
3.40000
6.20000
snBulk
IN
EN
Oligo
OPCs
Astro
Mural
Immune
Endo
IN LAMP5−RELN
IN LAMP5−LHX6
IN ADARB2
IN VIP
IN PVALB
IN PVALB−CHC
IN SST
EN L2−3−IT
EN L6−IT−1
EN L3−5−IT−2
EN L3−5−IT−3
EN L3−5−IT−1
EN L6−IT−2
EN L6−CT
EN L6B
EN L5−6−NP
MG
PVM
VLMC
PC
snTIM

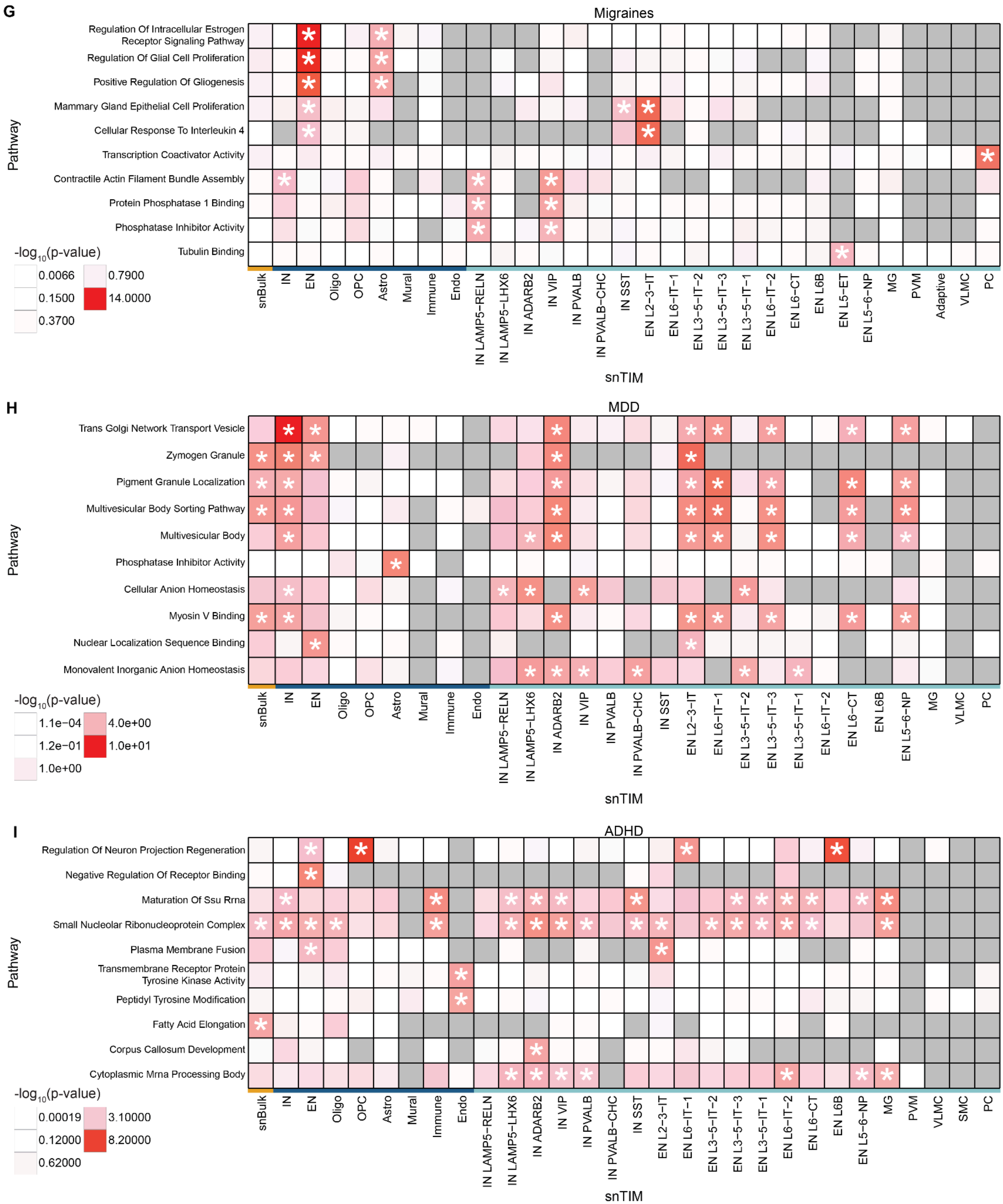
G
Migraines
Regulation Of Intracellular Estrogen Receptor Signaling Pathway
Regulation Of Glial Cell Proliferation
Positive Regulation Of Gliogenesis
Mammary Gland Epithelial Cell Proliferation
Cellular Response To Interleukin 4
Transcription Coactivator Activity
Contractile Actin Filament Bundle Assembly
Protein Phosphatase 1 Binding
Phosphatase Inhibitor Activity
Tubulin Binding
Pathway
-log10(p-value)
0.0066
0.1500
0.3700
0.7900
14.0000
snBulk
IN
EN
Oligo
OPC
Astro
Mural
Immune
Endo
IN LAMP5−RELN
IN LAMP5−LHX6
IN ADARB2
IN VIP
IN PVALB
IN PVALB−CHC
IN SST
EN L2−3−IT
EN L6−IT−1
EN L3−5−IT−2
EN L3−5−IT−3
EN L3−5−IT−1
EN L6−IT−2
EN L6−CT
EN L6B
EN L5−ET
EN L5−6−NP
MG
PVM
Adaptive
VLMC
PC
snTIM
H
MDD
Trans Golgi Network Transport Vesicle
Zymogen Granule
Pigment Granule Localization
Multivesicular Body Sorting Pathway
Multivesicular Body
Phosphatase Inhibitor Activity
Cellular Anion Homeostasis
Myosin V Binding
Nuclear Localization Sequence Binding
Monovalent Inorganic Anion Homeostasis
Pathway
-log10(p-value)
1.1e−04
1.2e−01
1.0e+00
4.0e+00
1.0e+01
snBulk
IN
EN
Oligo
OPC
Astro
Mural
Immune
Endo
IN LAMP5−RELN
IN LAMP5−LHX6
IN ADARB2
IN VIP
IN PVALB
IN PVALB−CHC
IN SST
EN L2−3−IT
EN L6−IT−1
EN L3−5−IT−2
EN L3−5−IT−3
EN L3−5−IT−1
EN L6−IT−2
EN L6−CT
EN L6B
EN L5−6−NP
MG
VLMC
PC
snTIM
I
ADHD
Regulation Of Neuron Projection Regeneration
Negative Regulation Of Receptor Binding
Maturation Of Ssu Rrna
Small Nucleolar Ribonucleoprotein Complex
Plasma Membrane Fusion
Transmembrane Receptor Protein Tyrosine Kinase Activity
Peptidyl Tyrosine Modification
Fatty Acid Elongation
Corpus Callosum Development
Cytoplasmic Mrna Processing Body
Pathway
-log10(p-value)
0.00019
0.12000
0.62000
3.10000
8.20000
snBulk
IN
EN
Oligo
OPC
Astro
Mural
Immune
Endo
IN LAMP5−RELN
IN LAMP5−LHX6
IN ADARB2
IN VIP
IN PVALB
IN PVALB−CHC
IN SST
EN L2−3−IT
EN L6−IT−1
EN L3−5−IT−2
EN L3−5−IT−3
EN L3−5−IT−1
EN L6−IT−2
EN L6−CT
EN L6B
EN L5−6−NP
MG
PVM
VLMC
SMC
PC
snTIM

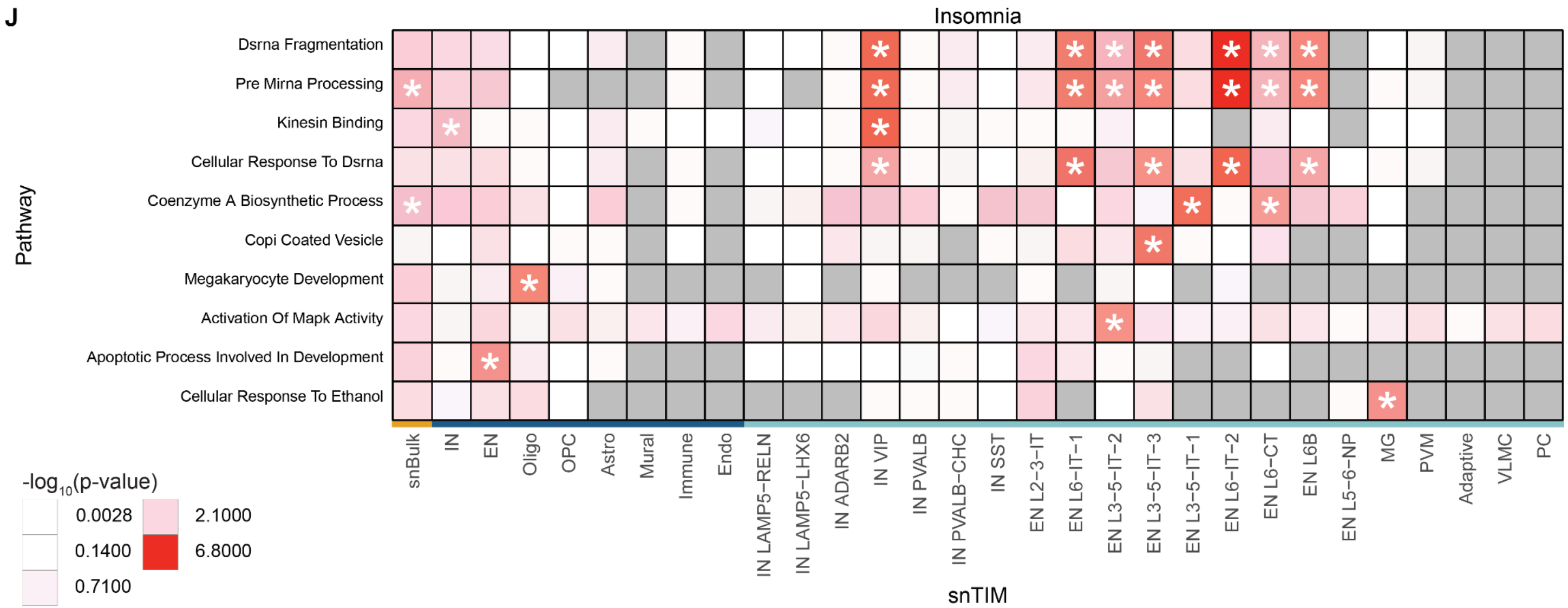

Supplementary Fig. 13 | Top 10 pathways enriched within all summary-level single-nucleus transcriptome-wide association study (S-snTWAS) traits. Pathways were prioritized by p-value. Hierarchical pruning was performed to deprioritize pathways (see Methods). For each cell-type, the top 2 significant pathways after hierarchical pruning (if any) were prioritized. We describe the following traits in each panel: (**BD**) (**A**), multiple sclerosis (**MS**) (**B**), alcohol use disorder (**AUD**) (**C**), amyotrophic lateral sclerosis (**ALS**) (**D**), Parkinson's disease (**PD**) (**E**), Anorexia (**F**), Migraines (**G**), major depressive disorder (**MDD**) (**H**), attention-deficit/hyperactivity disorder (**ADHD**) (**I**), Insomnia (**J**). Alzheimer's disease (**AD**) and schizophrenia (**SCZ**) are described within Extended Data Fig. 3C and Extended Data Fig. 3D, respectively. Full names of all cell-types are defined in Table S3.

Supplementary Fig. 14

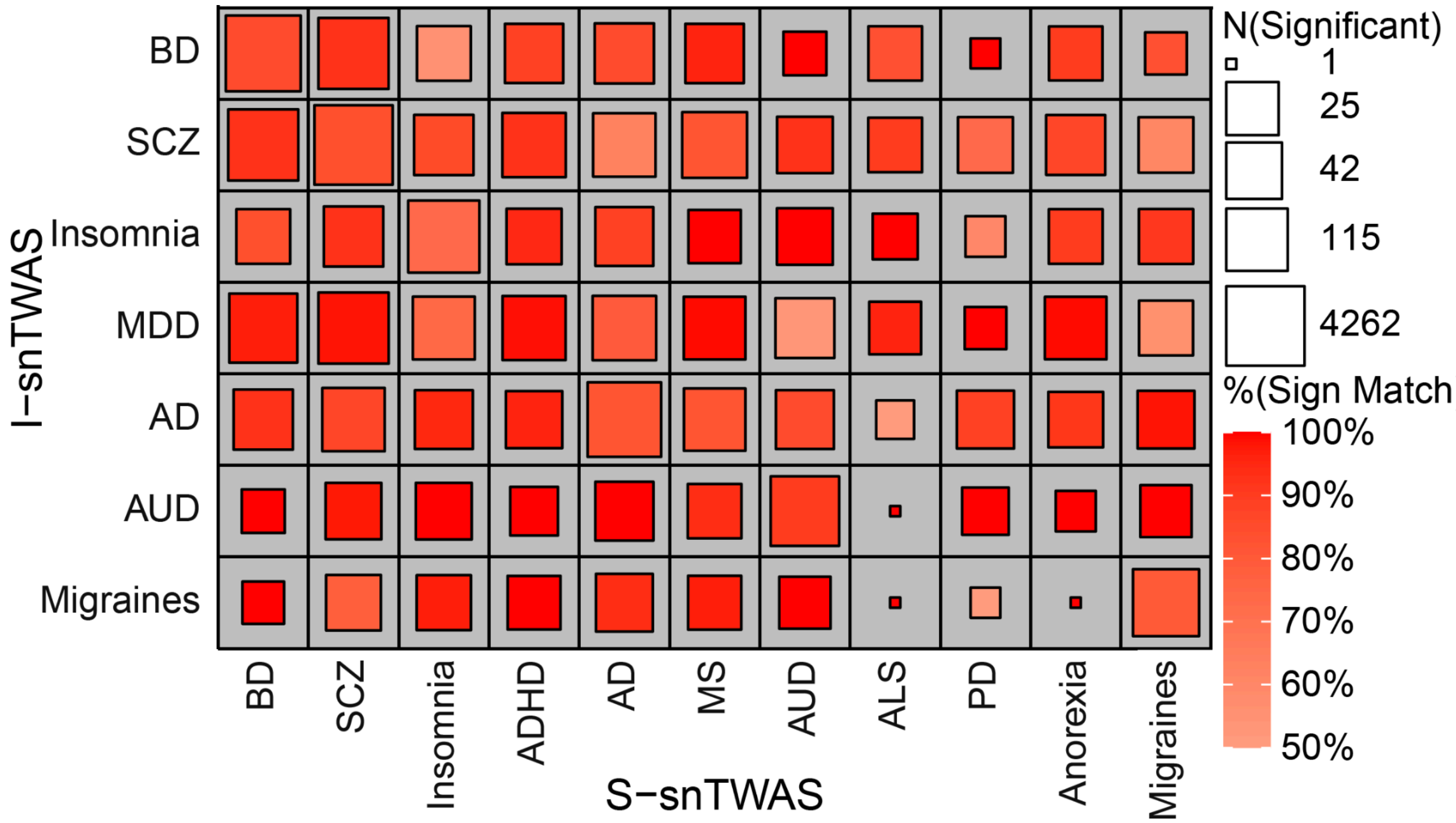

Supplementary Fig. 14 | Cross-disorder sign concordance between summary-level single-nucleus transcriptome-wide association study (S-snTWAS) and individual-level snTWAS (I-snTWAS). Because several genome-wide association study (**GWAS**) summary-statistic sets include overlapping participants (e.g., via United Kingdom Biobank), we compared S-snTWAS to the Million Veteran Program (**MVP**)-based I-snTWAS with no known sample overlap except for major depressive disorder (**MDD**) (which was excluded at the S-snTWAS level). Rows list traits analyzed with MVP I-snTWAS; columns list S-snTWAS traits (Table S12). We intersect the 12 S-snTWAS traits with the 9 sufficiently powered I-snTWAS traits. For each trait pair, we take S-snTWAS-significant gene-cell-type associations (false discovery rate (**FDR**)-adjusted p-value ≤ 0.05) and report the fraction with the same effect sign in I-snTWAS. Square size encodes the number of queried associations; color encodes percent sign match; gray cells were not evaluated. Diagonal cells (e.g., bipolar disorder (**BD**) vs. BD) reflect within-trait agreement. Overall, 90% (5,162 of 5,721) of cross-disorder associations show matching direction in the independent I-snTWAS.

Supplementary Fig. 15

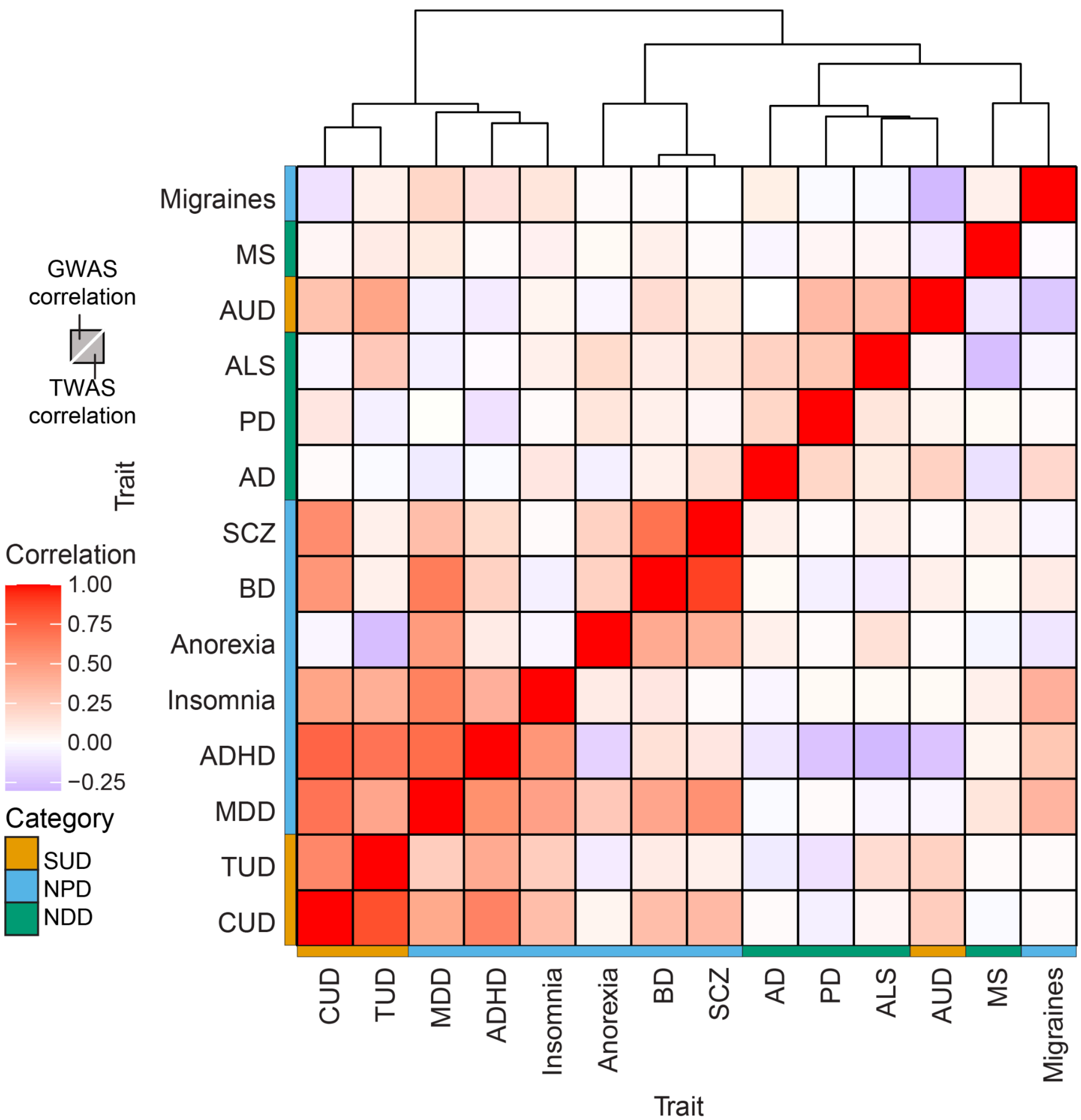

Supplementary Fig. 15 | Heatmap of summary-level single-nucleus transcriptome-wide association study (S-snTWAS) and genome-wide association study (GWAS) correlation. Heatmap is annotated by comparison category (color on x- and y-axes). The upper left triangle refers to GWAS correlation (r_g) and the lower right triangle refers to S-snTWAS Pearson's correlation. Disorders were clustered according to S-snTWAS correlation using ward D2 clustering. Full names of disorders are described in Table S12. SUD: substance use disorders. NPD: neuropsychiatric disorders. NDD: neurodegenerative disorders.

Supplementary Fig. 16

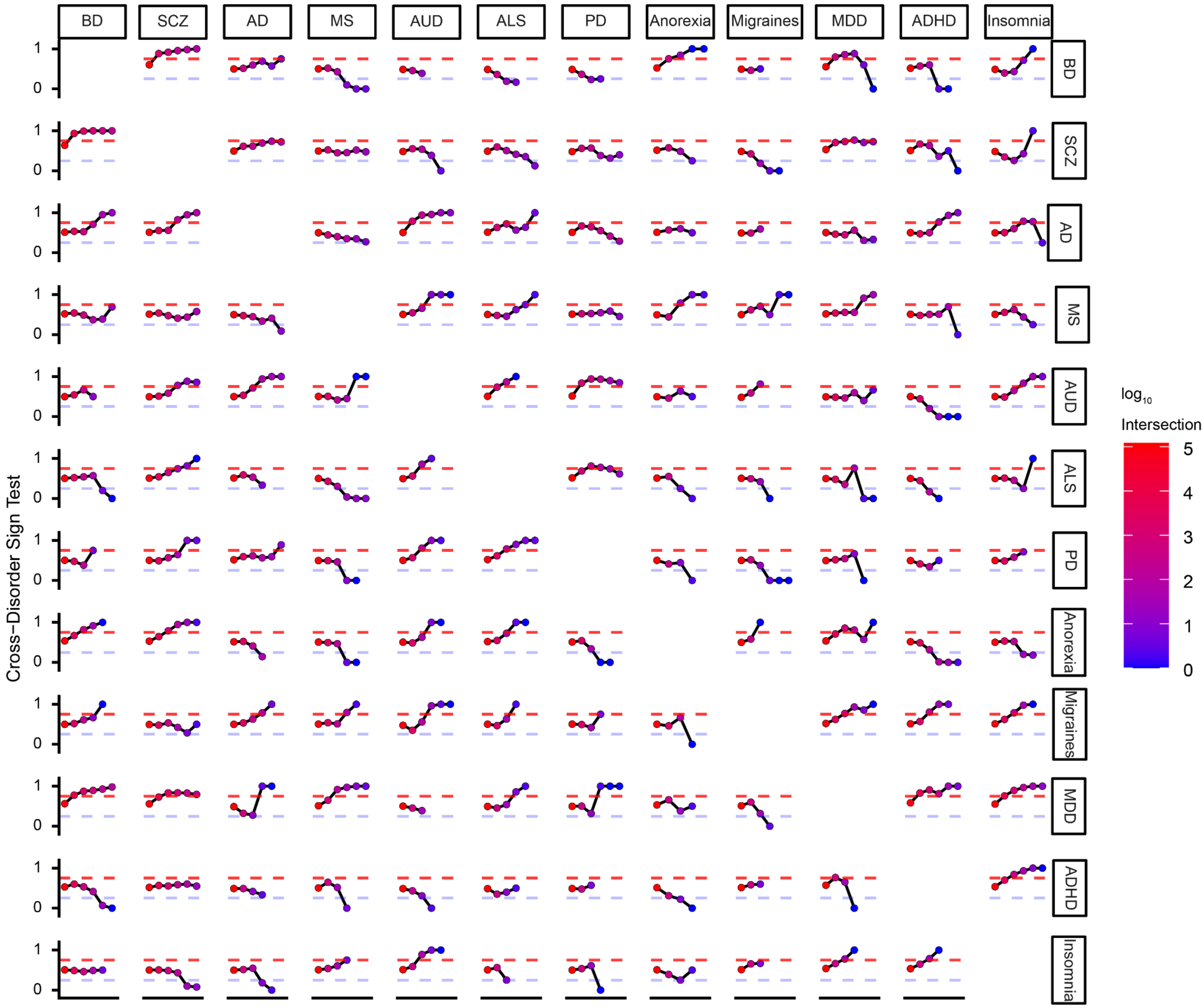

Supplementary Fig. 16 | Cross-disorder association concordance. Each point represents the percent of concordant gene-cell-type (*Lower-Left Triangle*) or pathway-cell-type (*Upper-Right Triangle*) combinations (same association z-score sign) under the appropriate p-value threshold in both disorders. Each progressive point restricts the p-value threshold 10-fold (i.e. p-value ≤ 1, p-value ≤ 1×10^{-1}, p-value ≤ 1×10^{-2}, …). Color indicates the number of intersecting combinations at the appropriate p-value threshold. Red and blue dashed horizontal lines denote a 0.75 (75%) and 0.25 (25%) sign concordance, respectively. Full names of disorders are described in Table S12.

Supplementary Fig. 17

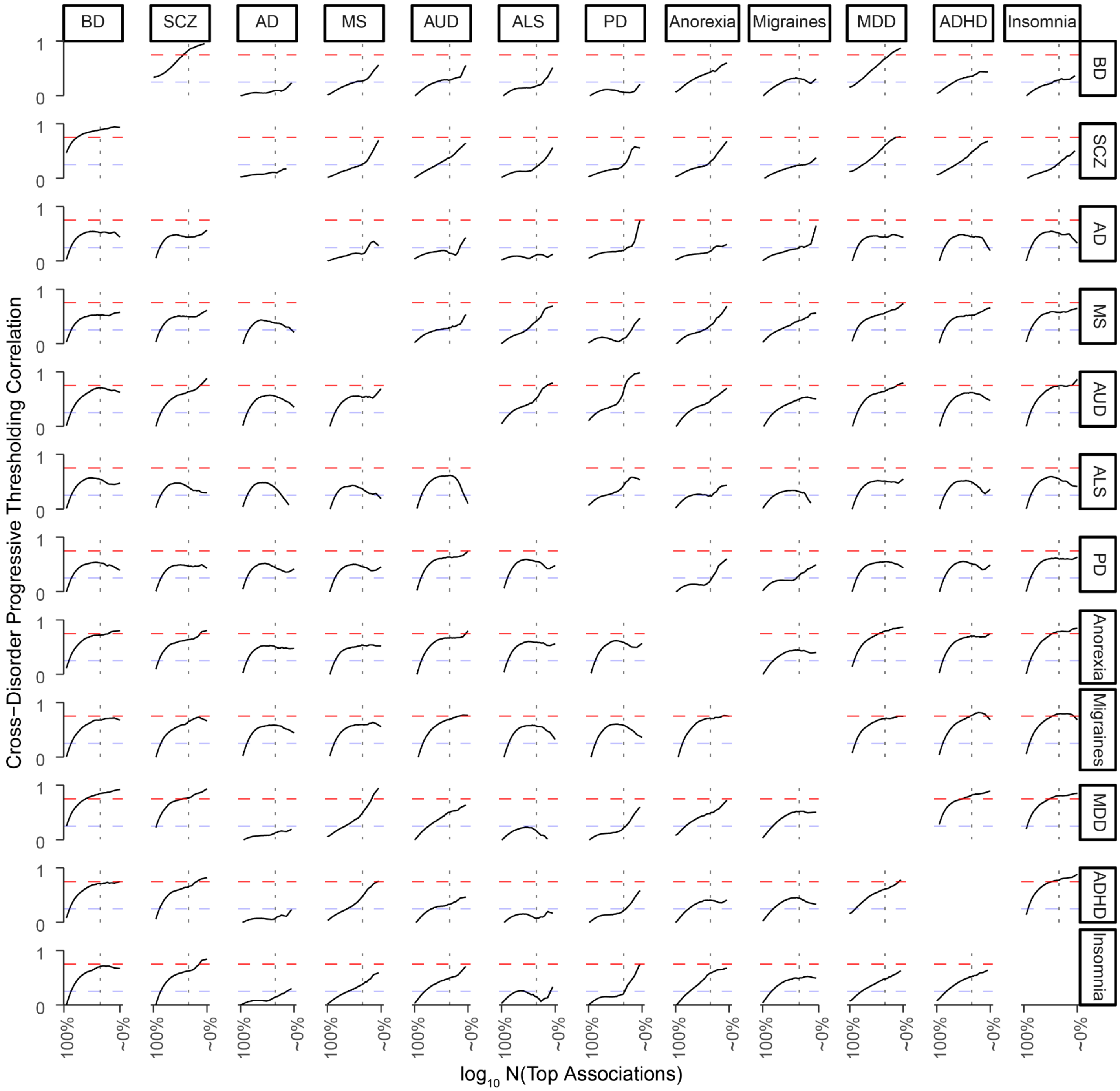

Supplementary Fig. 17 | Single-nucleus transcriptome-wide association study (snTWAS) cross-disorder progressive thresholding correlation analysis (PTCA). For each trait pair, line plots show the Pearson correlation (r) of association z-scores as increasingly stringent rank thresholds are applied (see Methods). The Lower-Left triangle summarizes gene-cell-type combinations; the Upper-Right triangle summarizes pathway-cell-type combinations. Dashed horizontal lines mark r = 0.75 (red) and r = 0.25 (blue). Vertical dotted lines indicate the top 1% threshold for each half (upper-right: gene-cell-type; lower-left: pathway-cell-type). Full names of disorders are described in Table S12.

Supplementary Fig. 18

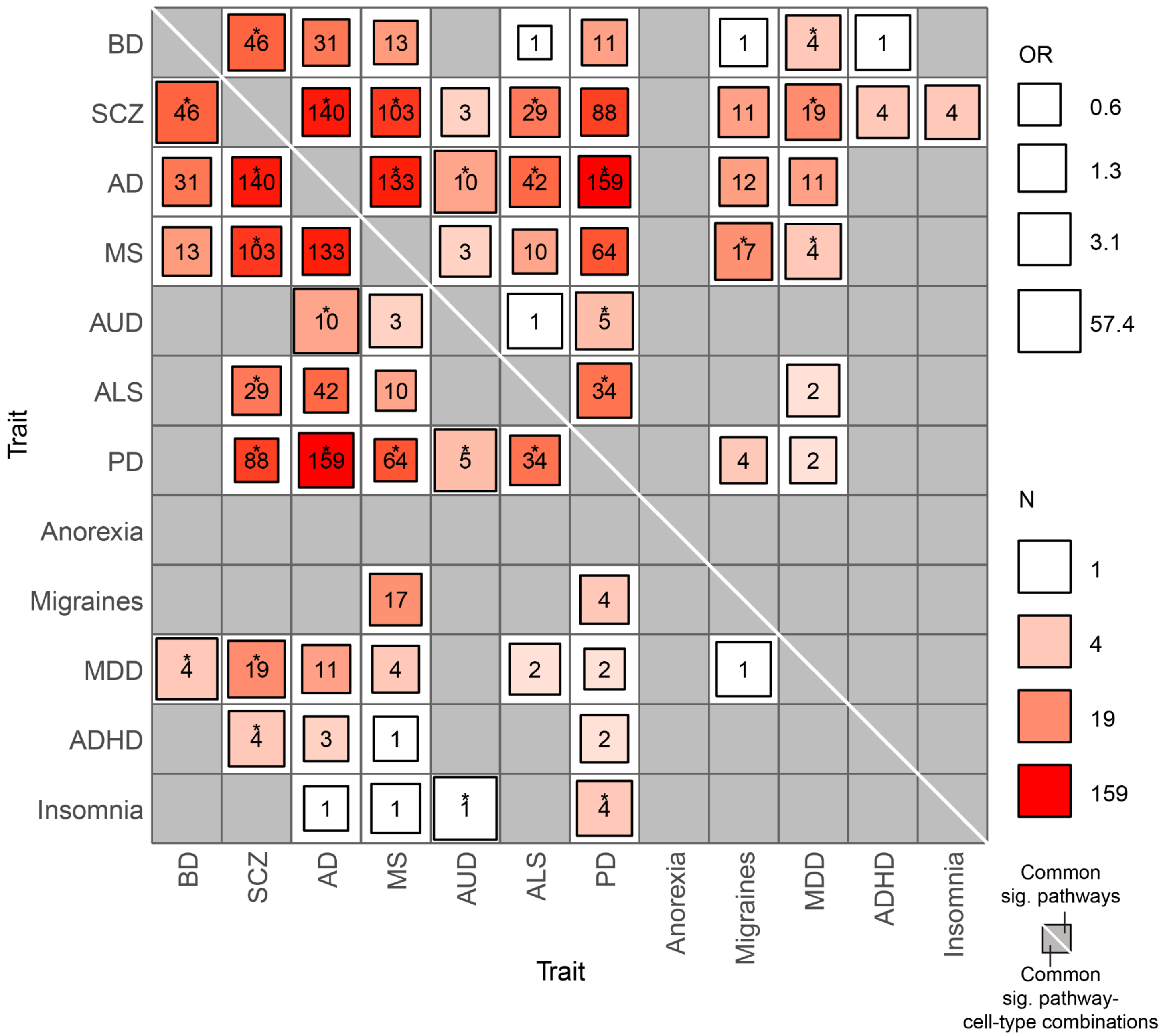

Supplementary Fig. 18 | Fisher's exact test Heatmap of cross-disorder significant gene sharing. *Lower-Left Triangle*: The number in each cell and the color describes the number of shared significant pathway-cell-type combinations (*lower-left triangle*) or pathways (*upper-right triangle*) between each pair of disorders. The size of each square corresponds to the odds ratio (**OR**). Fisher's exact test p-values were FDR-adjusted, and significant values are annotated by asterisks. Full names of disorders are described in Table S12.

Supplementary Fig. 19

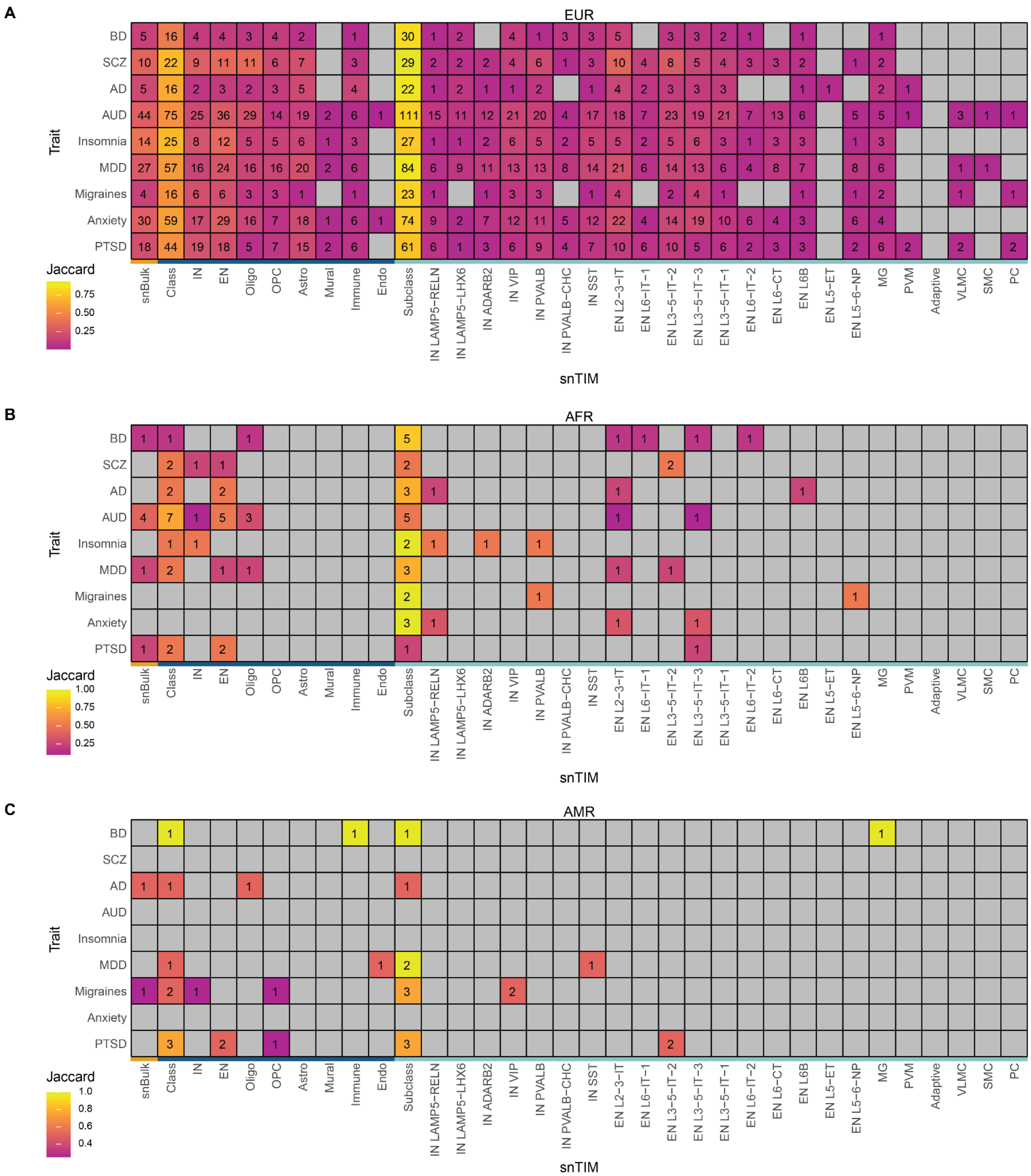

Supplementary Fig. 19 | Individual-level single-nucleus transcriptome-wide association study (I-snTWAS) gene-trait association (GTA) Heatmap for individuals of European (EUR) (A), African (AFR) (B) and Admixed American (AMR) (C) ancestries. The number within each cell represents the number of false discovery rate (**FDR**)-adjusted p-value significant gene-trait associations for the respective single-nucleus transcriptomic imputation model (**snTIM**). Cell color denotes the trait-specific Jaccard index, defined as the fraction of significant GTAs at that level (x) that overlap the union of significant GTAs across all levels for the same trait ($\frac{|x \cap all|}{|x \cup all|}$). "Class" and "Subclass" represent the number of FDR-adjusted p-value significant genes among all class- and subclass-level snTIMs, respectively. Full names of disorders are described in Table S12. Full names of all cell-types are defined in Table S3.

Supplementary Fig. 20

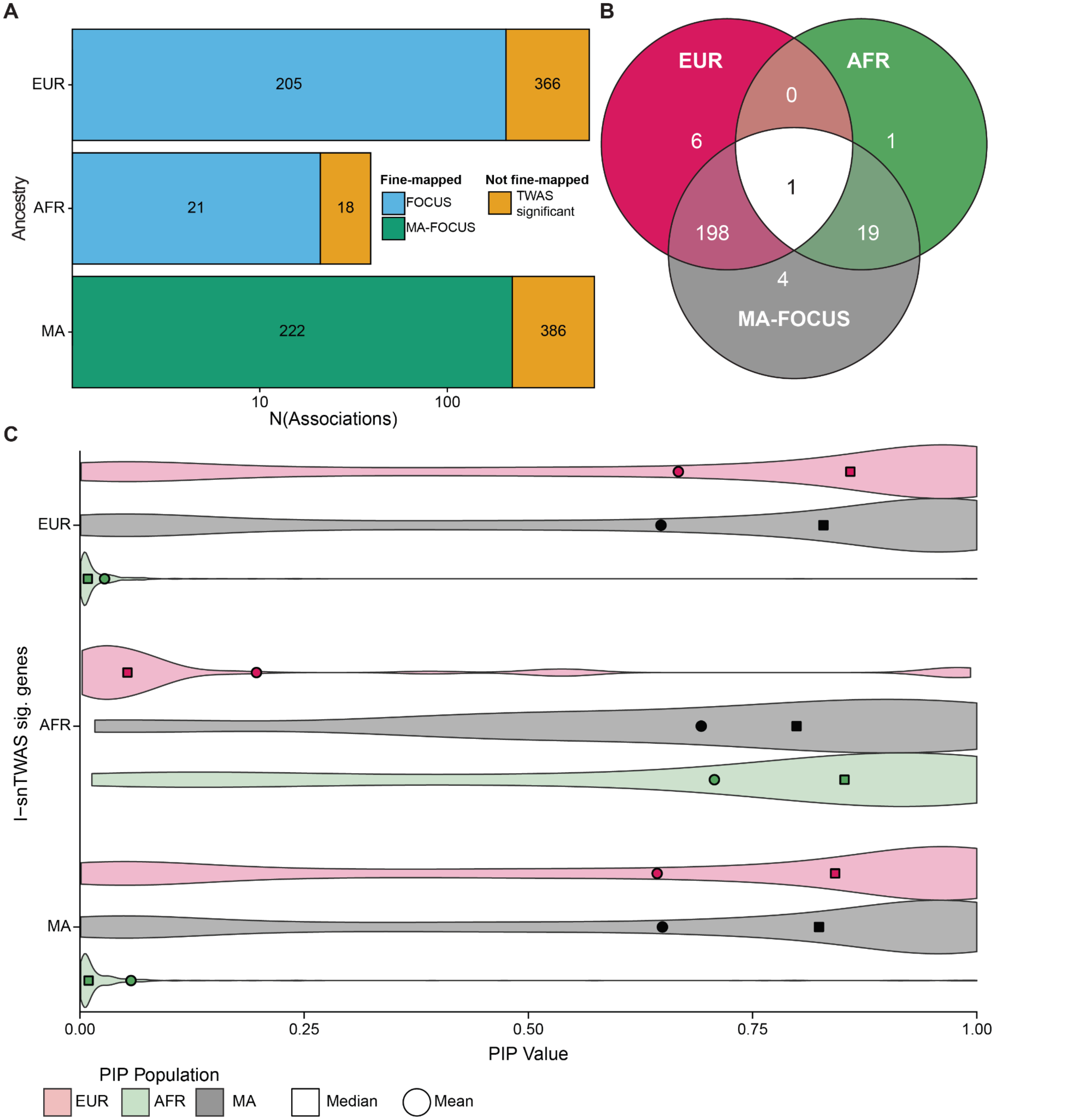

Supplementary Fig. 20 | Overlap of cross-ancestry fine-mapping. A, Overlap of transcriptome-wide association study (**TWAS**) and fine-mapping in single ancestry and multi-ancestry fine-mapping across the 9 individual-level single-nucleus TWAS **(I-snTWAS**) traits. Annotated numbers in the barplot indicate the total number of gene-trait associations (**GTA**s) in each category (i.e. there are 205 fine-mapped (posterior inclusion probability (**PIP**) ≥ 0.5) GTAs in individuals of European ancestry (**EUR**) and 801 I-snTWAS significant GTAs). Y-axis indicates the population in which analysis was performed. “MA” indicates the Multi-ancestry Fine-mapping Of CaUsal gene Sets

(**MA-FOCUS**) bi-ancestral analysis and the union of EUR and individuals of African ancestry (**AFR**) **I**-snTWAS significant trait-gene-cell-type combinations for fine-mapping and TWAS, respectively. **B,** Overlap of fine-mapped GTAs (PIP ≥ 0.5; FOCUS for EUR and AFR; MA-FOCUS for bi-ancestral EUR and AFR). **C,** Distribution of PIP values (FOCUS for EUR and AFR, MA-FOCUS for bi-ancestral EUR and AFR) amongst **I**-snTWAS significant trait-gene-cell-type combinations for each ancestry ("MA" indicates the union of EUR and AFR **I**-snTWAS significant trait-gene-cell-type combinations). Square points indicate the median values of each distribution and circular points indicate the mean values of each distribution.

Supplementary Fig. 21

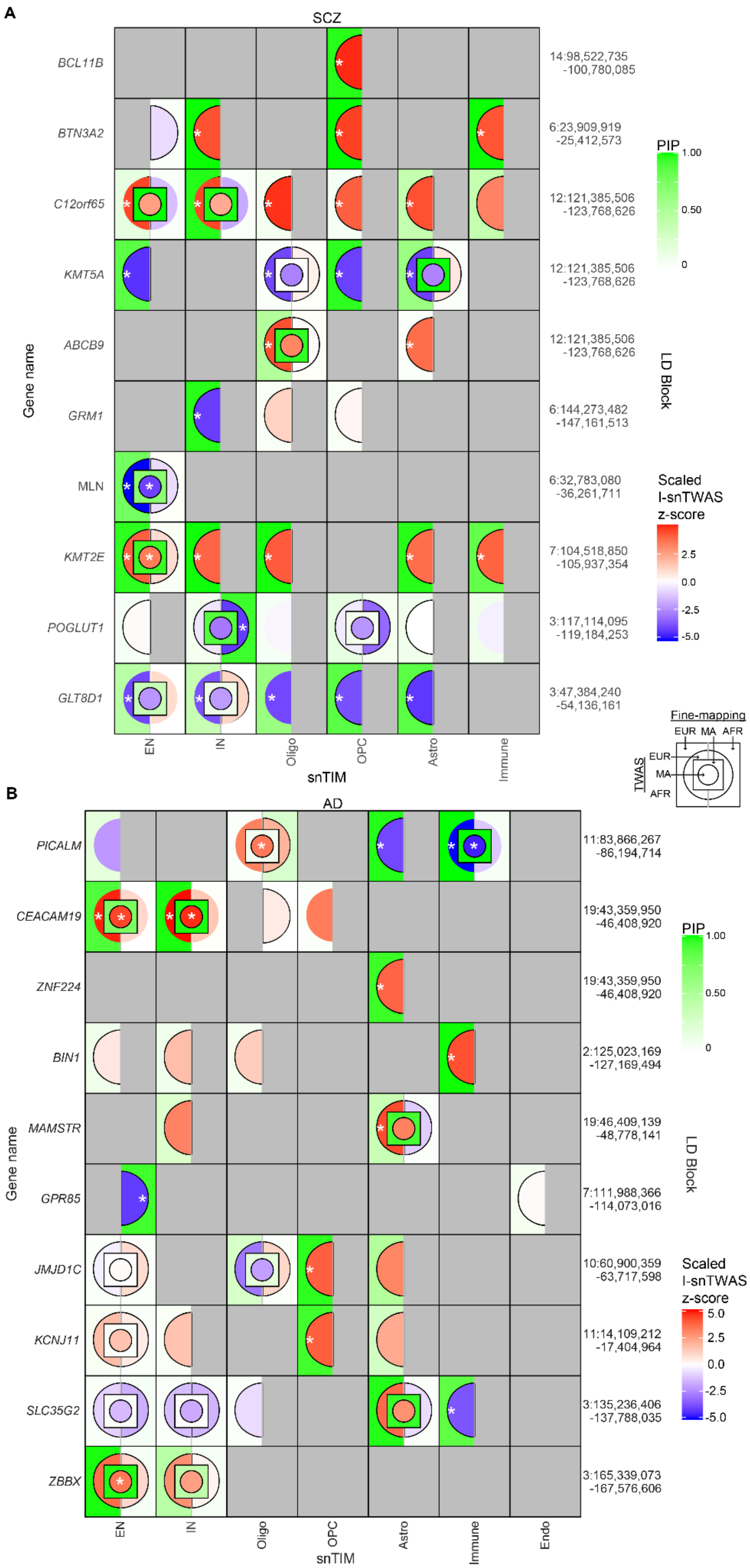

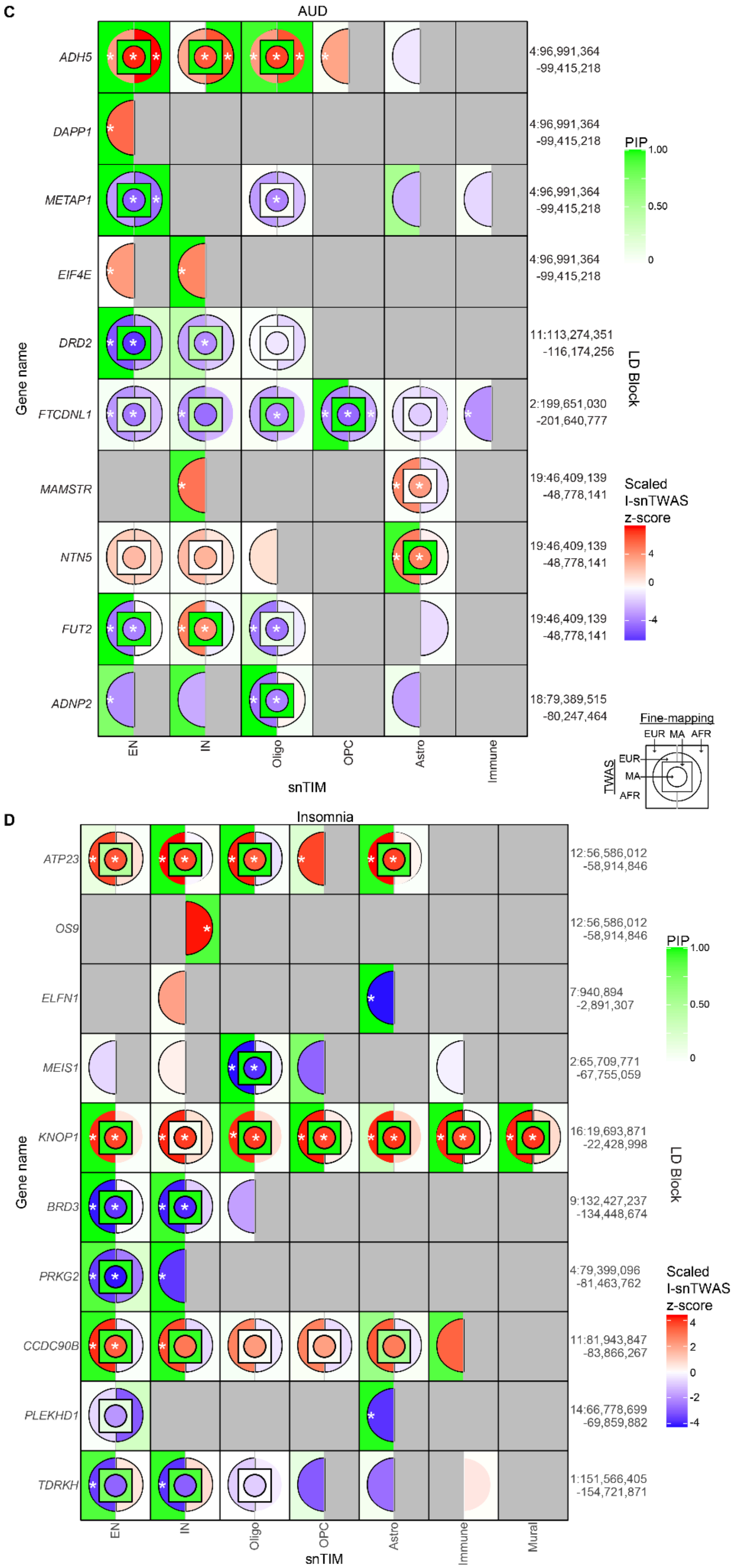

C
AUD
ADH5
DAPP1
METAP1
EIF4E
DRD2
FTCDNL1
MAMSTR
NTN5
FUT2
ADNP2
Gene name
4:96,991,364
-99,415,218
11:113,274,351
-116,174,256
2:199,651,030
-201,640,777
19:46,409,139
-48,778,141
18:79,389,515
-80,247,464
LD Block
PIP
1.00
0.50
0
Scaled
I-snTWAS
z-score
4
0
-4
EN
IN
Oligo
OPC
Astro
Immune
snTIM
Fine-mapping
EUR MA AFR
TWAS
EUR
MA
AFR
D
Insomnia
ATP23
OS9
ELFN1
MEIS1
KNOP1
BRD3
PRKG2
CCDC90B
PLEKHD1
TDRKH
Gene name
12:56,586,012
-58,914,846
7:940,894
-2,891,307
2:65,709,771
-67,755,059
16:19,693,871
-22,428,998
9:132,427,237
-134,448,674
4:79,399,096
-81,463,762
11:81,943,847
-83,866,267
14:66,778,699
-69,859,882
1:151,566,405
-154,721,871
LD Block
PIP
1.00
0.50
0
Scaled
I-snTWAS
z-score
4
2
0
-2
-4
EN
IN
Oligo
OPC
Astro
Immune
Mural
snTIM

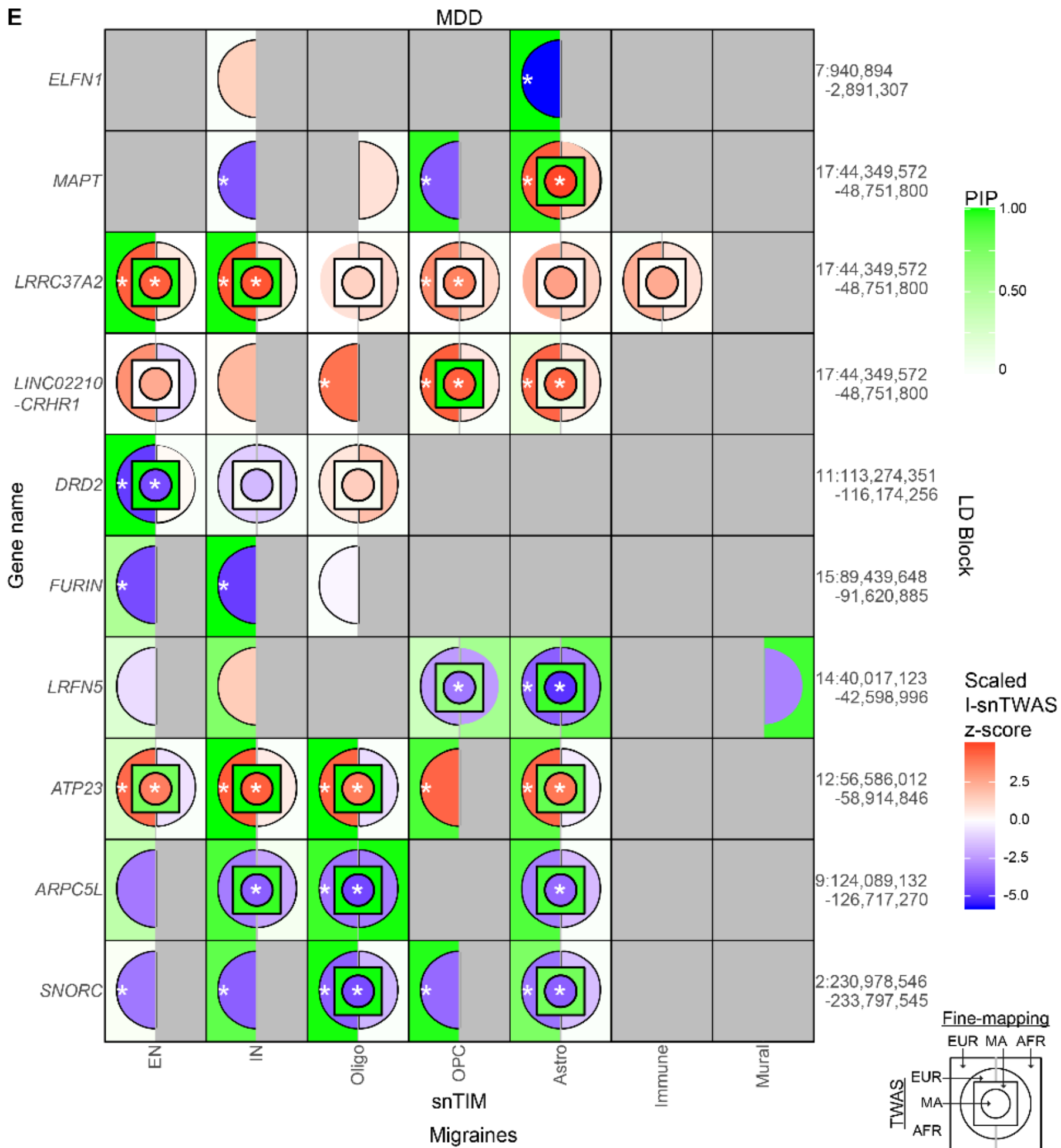
E
MDD
ELFN1
MAPT
LRRC37A2
LINC02210
-CRHR1
DRD2
FURIN
LRFN5
ATP23
ARPC5L
SNORC
Gene name
7:940,894
-2,891,307
17:44,349,572
-48,751,800
17:44,349,572
-48,751,800
17:44,349,572
-48,751,800
11:113,274,351
-116,174,256
15:89,439,648
-91,620,885
14:40,017,123
-42,598,996
12:56,586,012
-58,914,846
9:124,089,132
-126,717,270
2:230,978,546
-233,797,545
LD Block
PIP
1.00
0.50
0
Scaled
I-snTWAS
z-score
2.5
0.0
-2.5
-5.0
EN
IN
Oligo
OPC
Astro
Immune
Mural
snTIM
Fine-mapping
EUR
MA
AFR
TWAS
EUR
MA
AFR

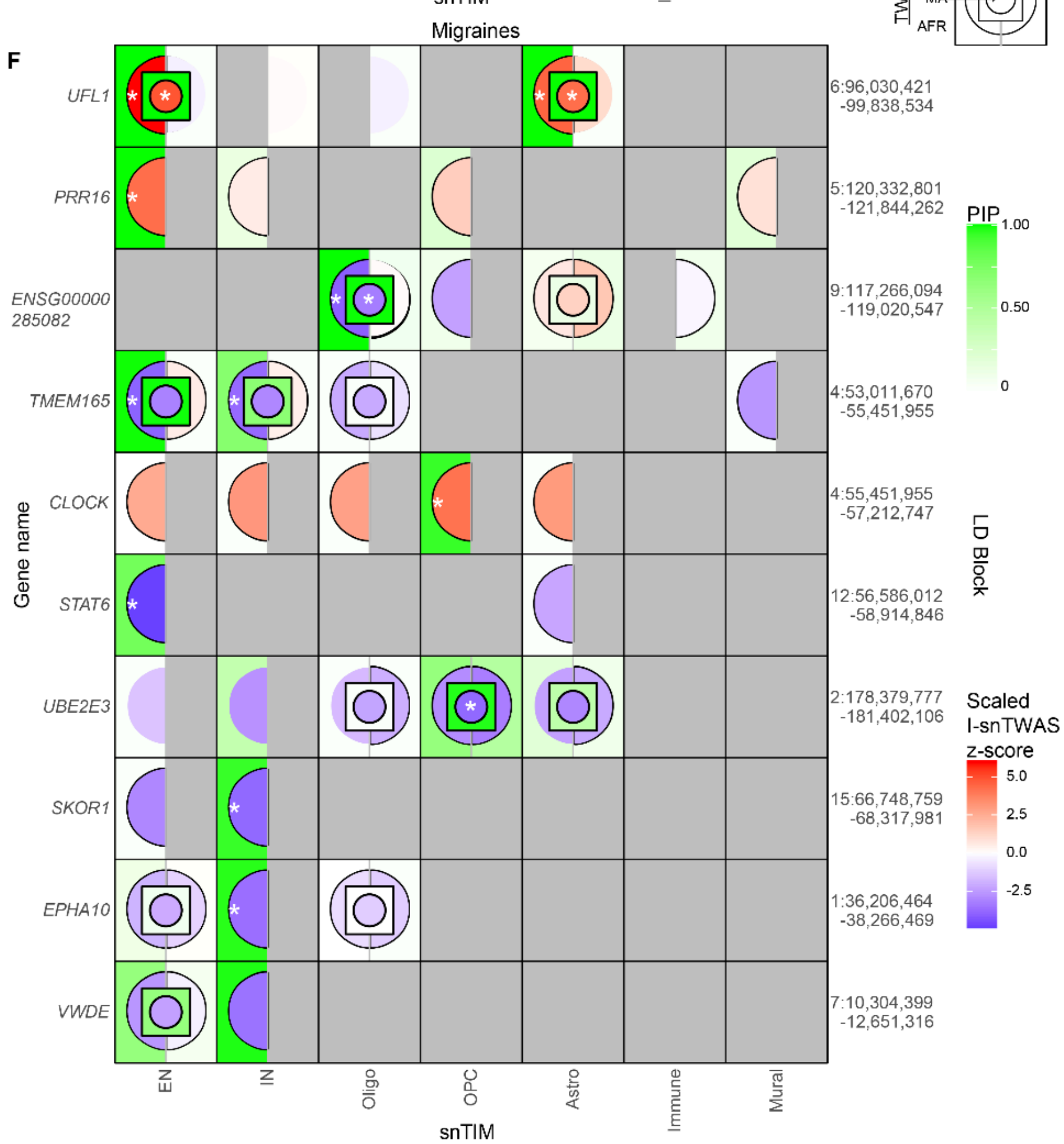
F
Migraines
UFL1
PRR16
ENSG00000
285082
TMEM165
CLOCK
STAT6
UBE2E3
SKOR1
EPHA10
VWDE
Gene name
6:96,030,421
-99,838,534
5:120,332,801
-121,844,262
9:117,266,094
-119,020,547
4:53,011,670
-55,451,955
4:55,451,955
-57,212,747
12:56,586,012
-58,914,846
2:178,379,777
-181,402,106
15:66,748,759
-68,317,981
1:36,206,464
-38,266,469
7:10,304,399
-12,651,316
LD Block
PIP
1.00
0.50
0
Scaled
I-snTWAS
z-score
5.0
2.5
0.0
-2.5
EN
IN
Oligo
OPC
Astro
Immune
Mural
snTIM

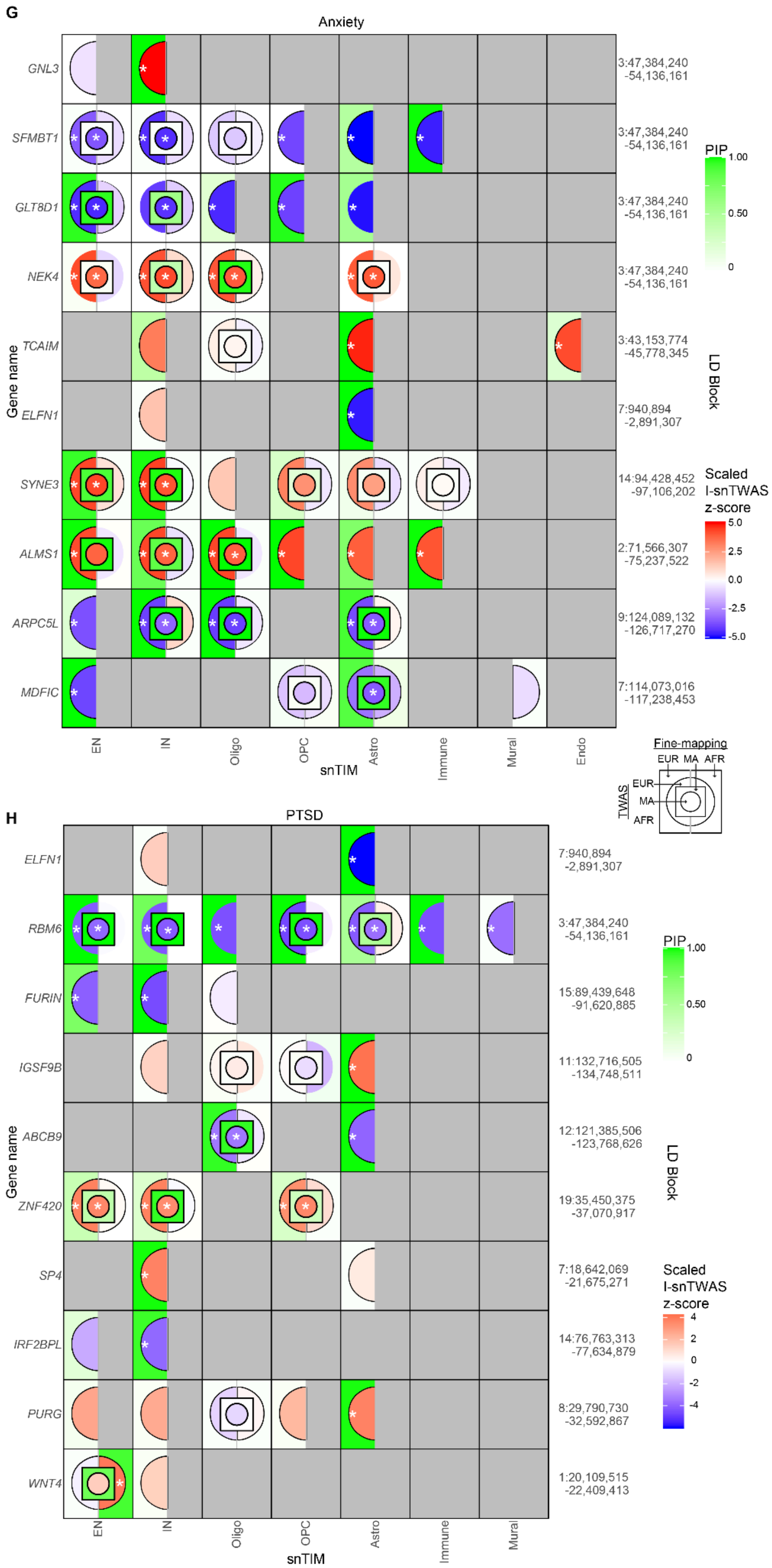

Supplementary Fig. 21 | Multi-ancestry Fine-mapping Of CaUsal gene Sets (MA-FOCUS) plots for neuropsychiatric and neurodegenerative disorders (NPD and NDDs). Bi-ancestral (individuals of European and African ancestry (**EUR** and **AFR**)) fine-mapping of schizophrenia (**SCZ**) (**A**), Alzheimer's disease (**AD**) (**B**), alcohol use disorder (**AUD**) (**C**), Insomnia (**D**), major depressive disorder (**MDD**) (**E**), Migraines (**F**), Anxiety (**G**), post-traumatic stress disorder (**PTSD**) (**H**). The top 10 trait-associated genes across EUR and AFR individual-level single-nucleus transcriptome-wide

association study (**I-snTWAS analysis**) are visualized across class level single-nucleus transcriptomic imputation models (**snTIMs**). Z-scores are scaled across all genes within each population. Bipolar disorder (**BD**) is visualized in Fig. 5E. Full names of all cell-types are defined in Table S3. PIP: posterior inclusion probability.

Supplementary Fig. 22

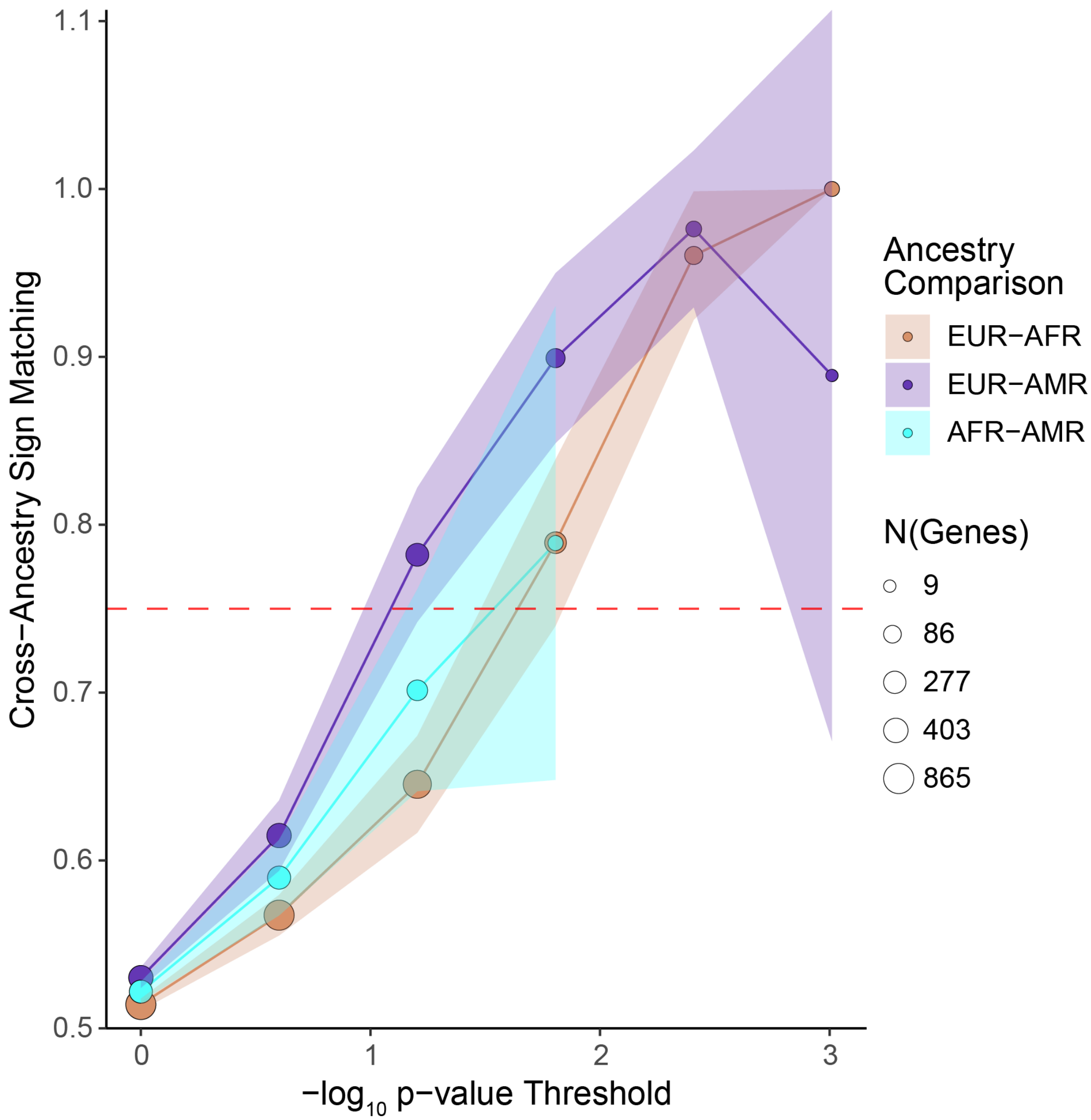

Supplementary Fig. 22 | Conservation of cross-ancestry concordance of single-nucleus GReX phenome-wide association study (snGReX-PheWAS) associations among top trait-gene-cell-type combinations. We selected significant genes from our summary-level and individual-level single-nucleus transcriptome-wide association study (**S-** and **I-snTWAS**) (false discovery rate (**FDR**)-adjusted p-value ≤ 0.05), and performed PheWAS on the top 623 associations (Bonferroni-adjusted p-value ≤ 0.05 across all confidently imputable associations within each cell-type in S- or I-snTWAS and at least FDR-adjusted p-value ≤ 0.05 in the complementary snTWAS analysis) to maximize analytical yield while remaining within computational constraints. For each pairwise ancestry combination, trait-specific PheWAS associations were binned (x-axis) based on the p-value of the shared gene-cell-type combinations in both ancestries (N=865, 403 and 324 in individuals of European ancestry (**EUR**)-individuals of African ancestry (**AFR**), EUR-individuals of Admixed American ancestry (**AMR**), and AFR-AMR, respectively); only phecodes with at least 500 cases and 500 controls were considered. The percentage of associations with concordant association direction is visualized (y-axis) at every marked threshold of p-value threshold (x-axis) for each ancestry combination (color). The shaded region around each line represents the 95% confidence interval. Size of the point represents the number of gene-cell-type combinations at that threshold. To

visualize a specific p-value threshold, we require a minimum number of observations corresponding to 2% of the applicable gene-cell-type combinations (18, 9, and 7 for EUR-AFR, EUR-AMR and AFR-AMR, respectively). Please note that we are underpowered for the AFR-AMR comparison.

Supplementary Fig. 23

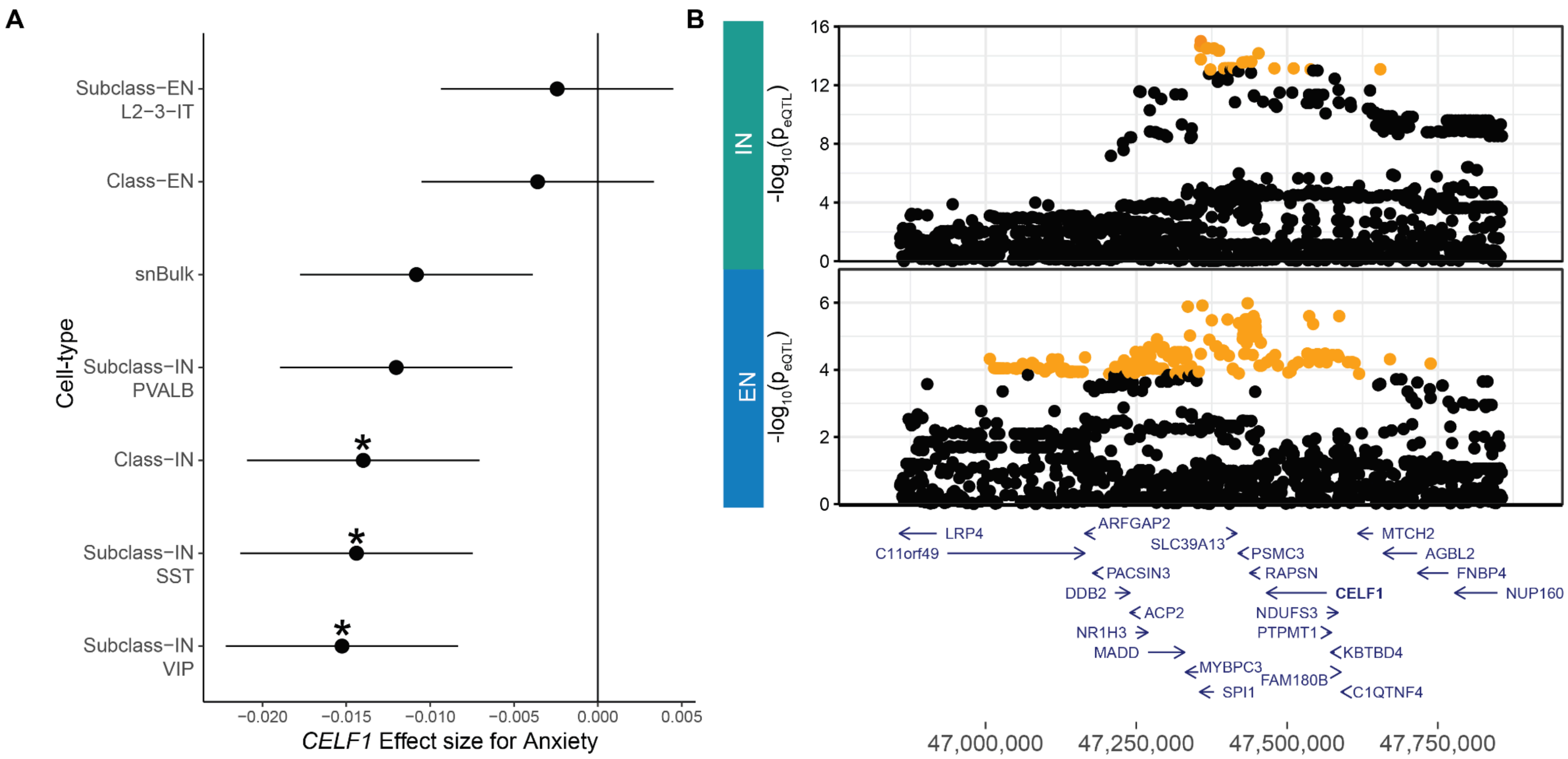

Supplementary Fig. 23 | Investigation of *CELF1* heterogeneity. A, Forest plot for the association of *CELF1* and anxiety in different cell-types. Significant associations are marked by an asterisk. Horizontal bars denote the 95% confidence interval. Full names of all cell-types are defined in Table S3. **B,** Single-nucleus expression quantitative trait loci (**sn-eQTL**) manhattan plot for *CELF1*. Color indicates posterior inclusion probability from statistical fine-mapping. The top single nucleotide polymorphism (**SNP**) for Class-Inhibitory Neurons (**IN**) is rs3824869 and the top SNP for Class-Excitatory Neurons (**EN**) is rs111228939. The R^2 between these SNPs is 0.081 in individuals of European ancestry (**EUR**)[38].

Supplementary Fig. 24

A

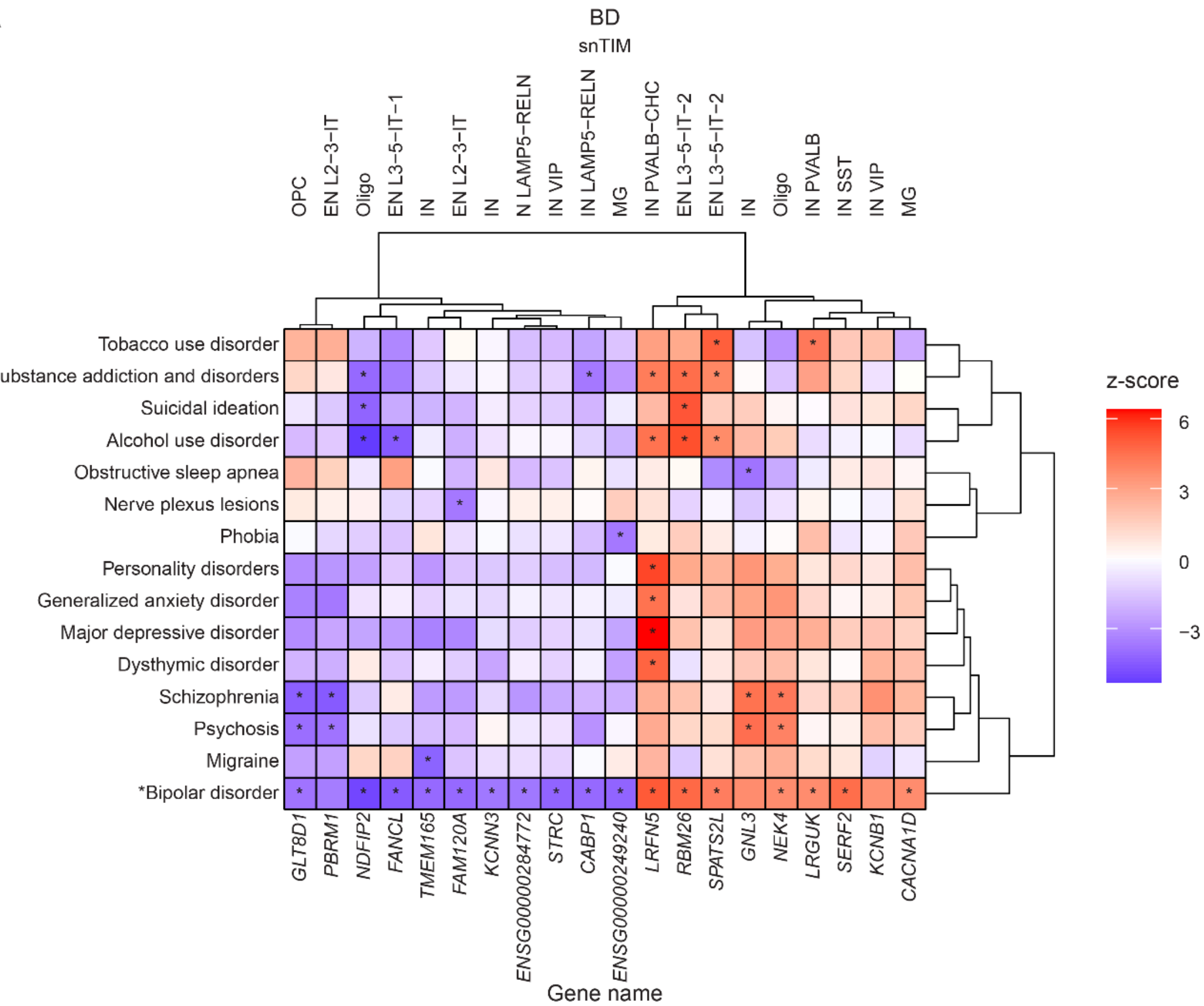

B

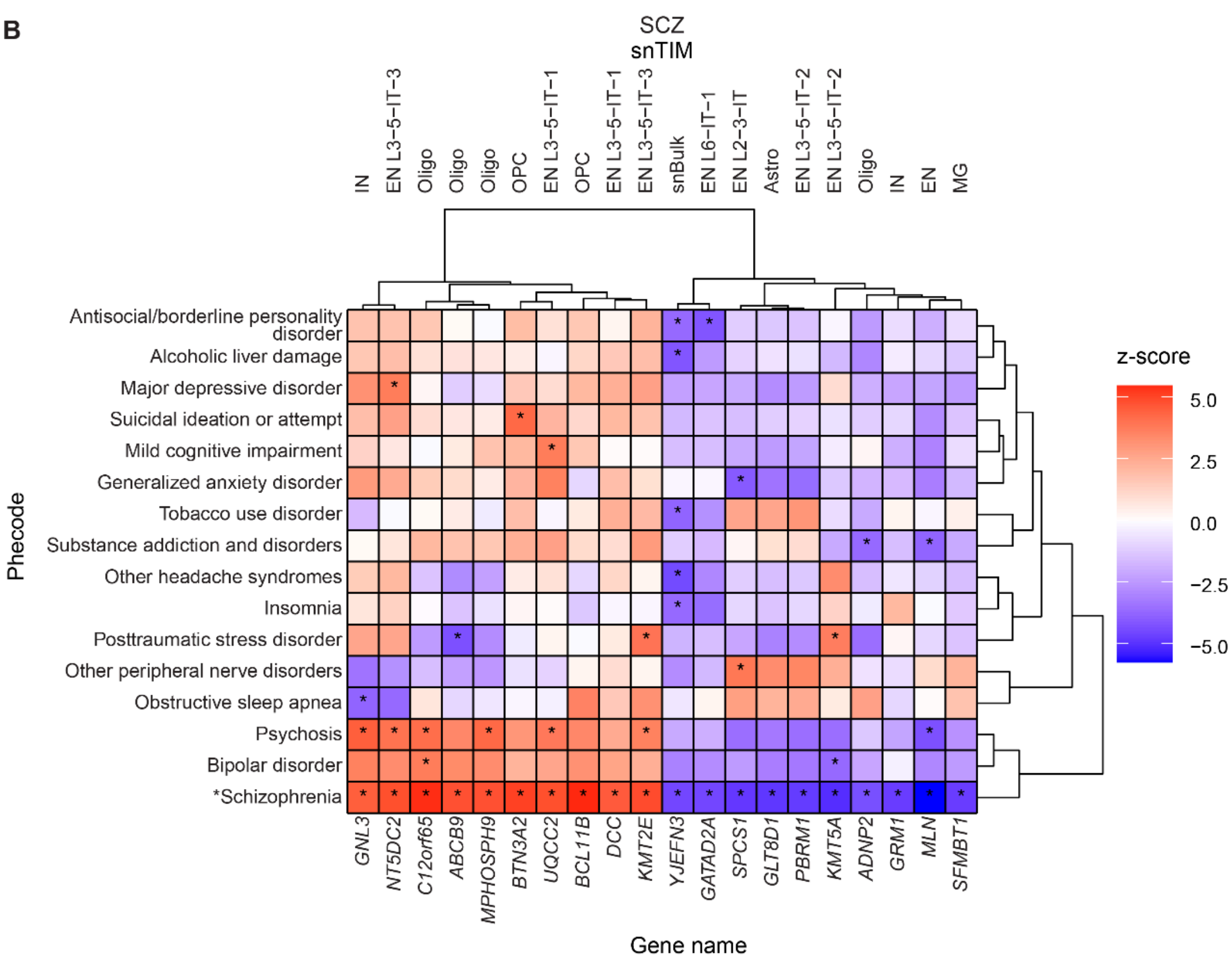

C

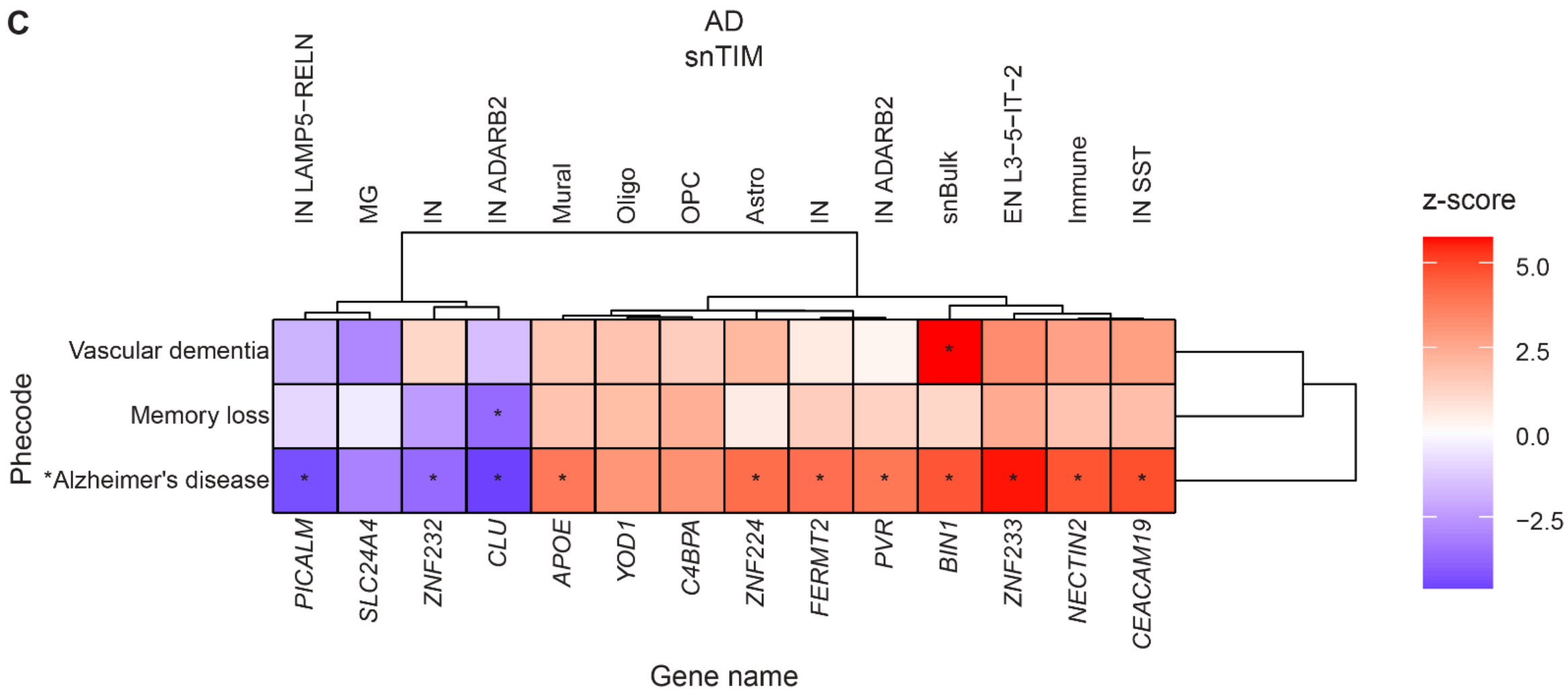
AD
snTIM
IN LAMP5−RELN
MG
IN
IN ADARB2
Mural
Oligo
OPC
Astro
IN
IN ADARB2
snBulk
EN L3−5−IT−2
Immune
IN SST
z-score
5.0
2.5
0.0
−2.5
Vascular dementia
Memory loss
*Alzheimer's disease
Phecode
PICALM
SLC24A4
ZNF232
CLU
APOE
YOD1
C4BPA
ZNF224
FERMT2
PVR
BIN1
ZNF233
NECTIN2
CEACAM19
Gene name

D

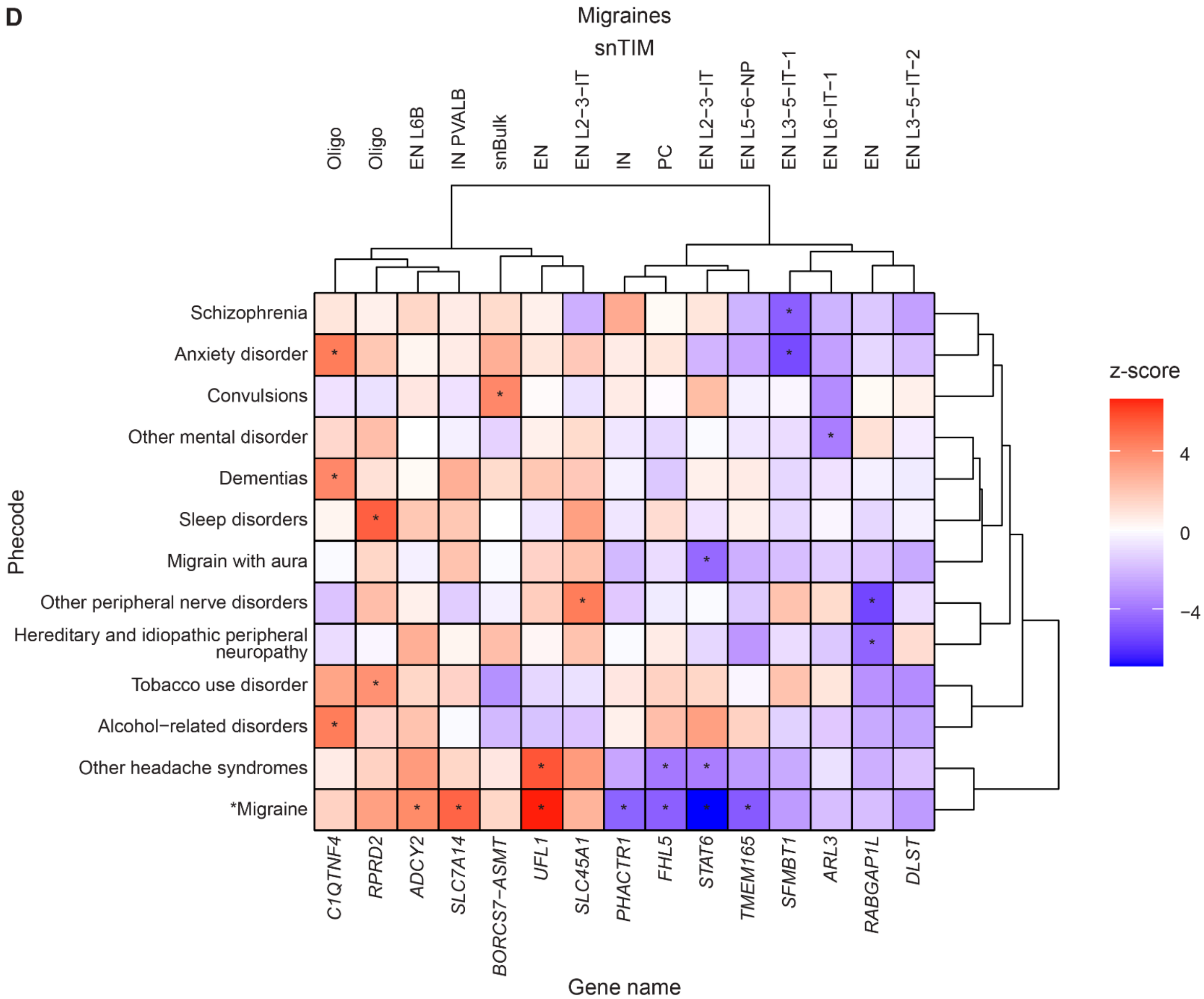
Migraines
snTIM
Oligo
Oligo
EN L6B
IN PVALB
snBulk
EN
EN L2−3−IT
IN
PC
EN L2−3−IT
EN L5−6−NP
EN L3−5−IT−1
EN L6−IT−1
EN
EN L3−5−IT−2
z-score
4
0
−4
Schizophrenia
Anxiety disorder
Convulsions
Other mental disorder
Dementias
Sleep disorders
Migrain with aura
Other peripheral nerve disorders
Hereditary and idiopathic peripheral neuropathy
Tobacco use disorder
Alcohol−related disorders
Other headache syndromes
*Migraine
Phecode
C1QTNF4
RPRD2
ADCY2
SLC7A14
BORCS7−ASMT
UFL1
SLC45A1
PHACTR1
FHL5
STAT6
TMEM165
SFMBT1
ARL3
RABGAP1L
DLST
Gene name

E

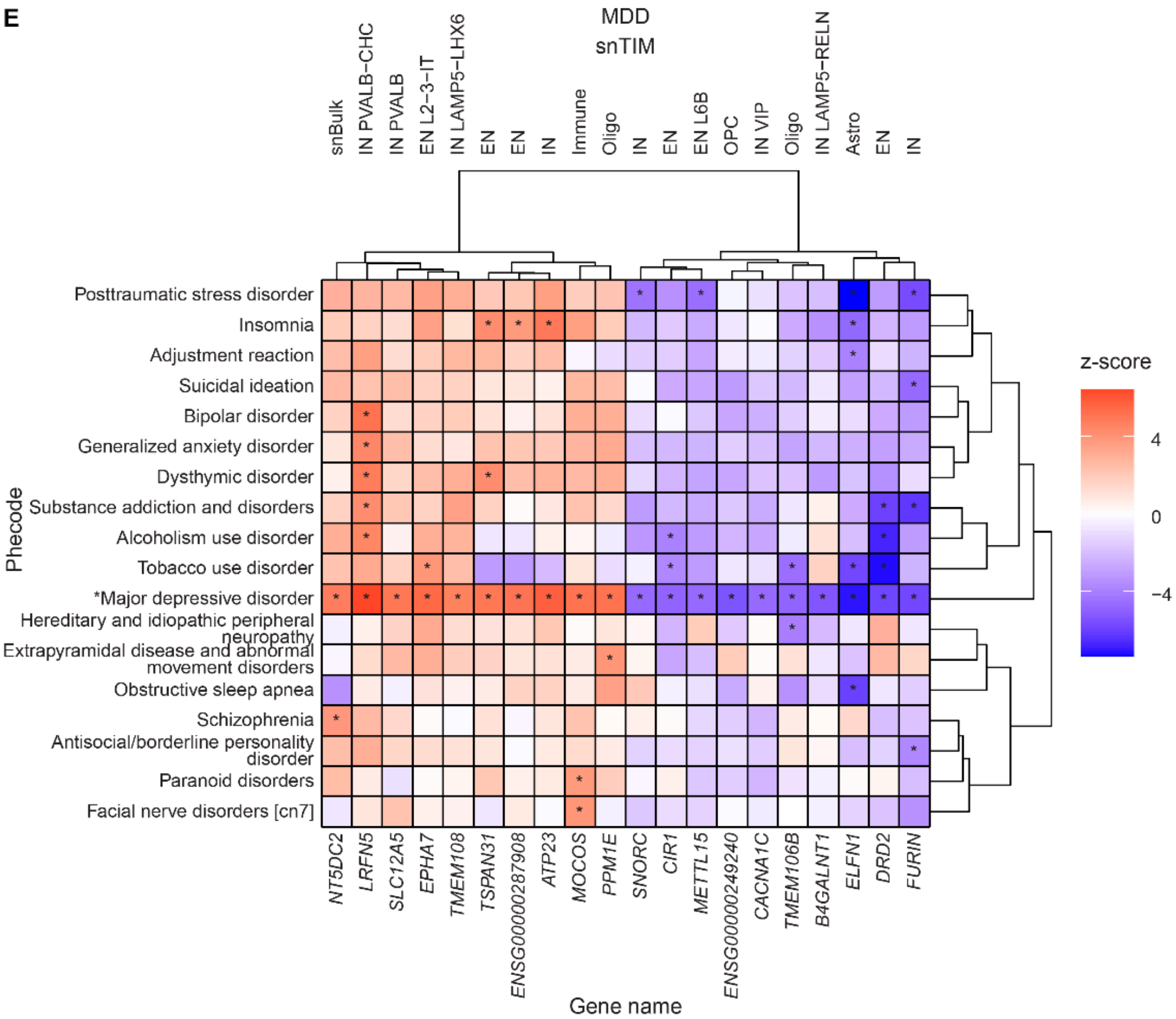
MDD
snTIM
snBulk
IN PVALB−CHC
IN PVALB
EN L2−3−IT
IN LAMP5−LHX6
EN
EN
IN
Immune
Oligo
IN
EN
EN L6B
OPC
IN VIP
Oligo
IN LAMP5−RELN
Astro
EN
IN
Posttraumatic stress disorder
Insomnia
Adjustment reaction
Suicidal ideation
Bipolar disorder
Generalized anxiety disorder
Dysthymic disorder
Substance addiction and disorders
Alcoholism use disorder
Tobacco use disorder
*Major depressive disorder
Hereditary and idiopathic peripheral neuropathy
Extrapyramidal disease and abnormal movement disorders
Obstructive sleep apnea
Schizophrenia
Antisocial/borderline personality disorder
Paranoid disorders
Facial nerve disorders [cn7]
Phecode
NT5DC2
LRFN5
SLC12A5
EPHA7
TMEM108
TSPAN31
ENSG00000287908
ATP23
MOCOS
PPM1E
SNORC
CIR1
METTL15
ENSG00000249240
CACNA1C
TMEM106B
B4GALNT1
ELFN1
DRD2
FURIN
Gene name
z-score
4
0
−4

F

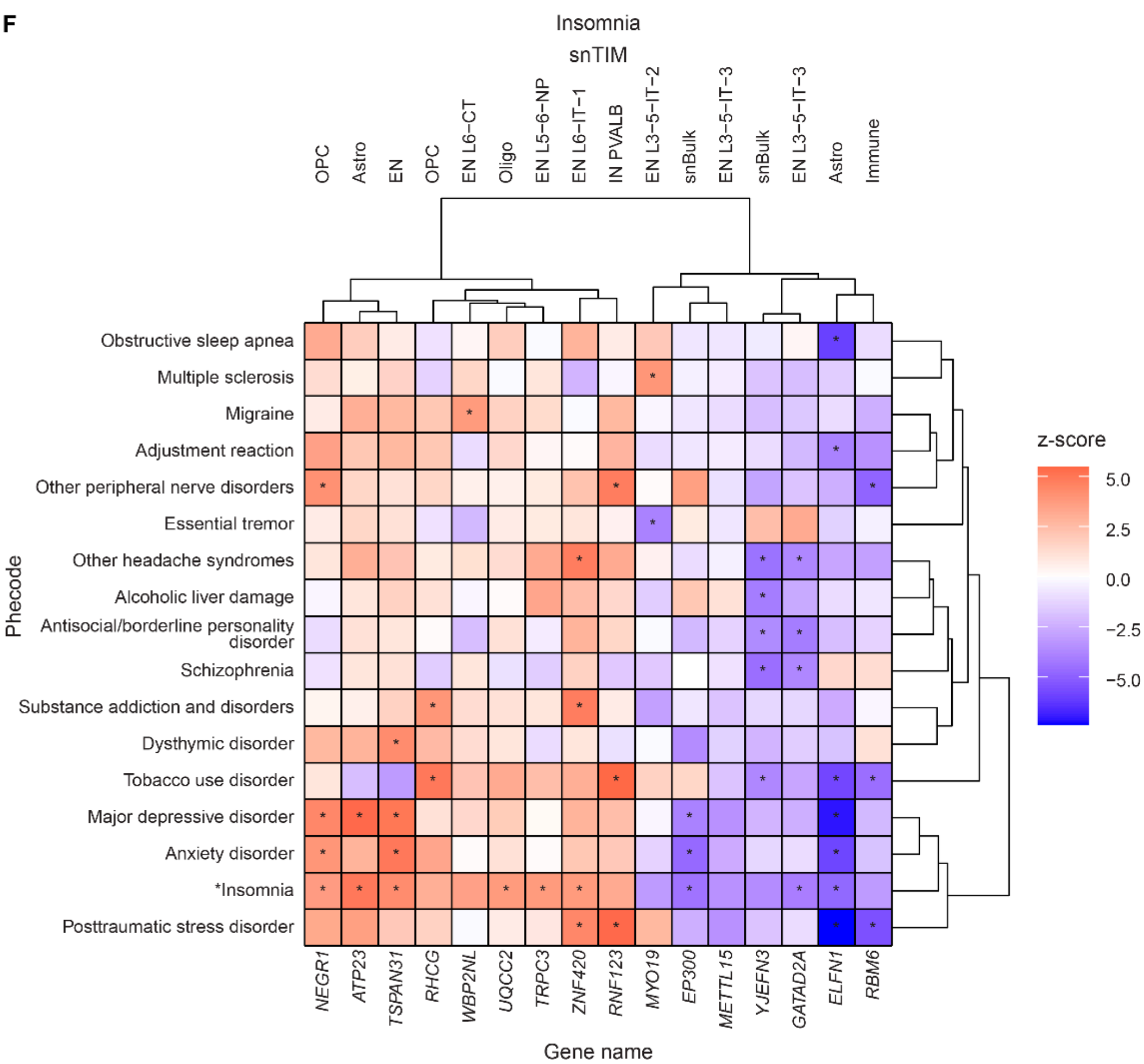
Insomnia
snTIM
OPC
Astro
EN
OPC
EN L6−CT
Oligo
EN L5−6−NP
EN L6−IT−1
IN PVALB
EN L3−5−IT−2
snBulk
EN L3−5−IT−3
snBulk
EN L3−5−IT−3
Astro
Immune
Obstructive sleep apnea
Multiple sclerosis
Migraine
Adjustment reaction
Other peripheral nerve disorders
Essential tremor
Other headache syndromes
Alcoholic liver damage
Antisocial/borderline personality disorder
Schizophrenia
Substance addiction and disorders
Dysthymic disorder
Tobacco use disorder
Major depressive disorder
Anxiety disorder
*Insomnia
Posttraumatic stress disorder
Phecode
NEGR1
ATP23
TSPAN31
RHCG
WBP2NL
UQCC2
TRPC3
ZNF420
RNF123
MYO19
EP300
METTL15
YJEFN3
GATAD2A
ELFN1
RBM6
Gene name
z-score
5.0
2.5
0.0
−2.5
−5.0

Supplementary Fig. 24 | Clustering of top phenome-wide association study (PheWAS) associations in summary-level single-nucleus transcriptome-wide association study (S-snTWAS). PheWAS for the top 20 gene-cell-type combinations ranked from the S-snTWAS and individual-level snTWAS (**I-snTWAS**) for each trait; each gene is represented by the cell-type with the lowest association p-value. In addition to each trait (mapped to the bolded italicized phecode), the top 20 (if at least 20 distinct phecodes are significant amongst the top 20 gene-cell-type combinations) phecodes among all phecode categories ranked by association p-value are visualized. Ward's hierarchical agglomerative clustering was performed with Ward's criterion preservation. We describe the following traits in each panel: bipolar disorder (**BD**) (**A**), schizophrenia (**SCZ**) (**B**), Alzheimer's disease (**AD**) (**C**), Migraines (**D**), major depressive disorder (**MDD**) (**E**), Insomnia (**F**). Alcohol use disorder (**AUD**) is described within Fig. 6C. Attention-deficit/hyperactivity disorder (**ADHD**), ALS, anorexia, multiple sclerosis (**MS**), and Parkinson's disease (**PD**) are excluded due to few cases in the Million Veteran Program (**MVP**). Post-traumatic stress disorder (**PTSD**) and anxiety are excluded due to few significant S-snTWAS associations. Full names of all cell-types are defined in Table S3.

Supplementary Fig. 25

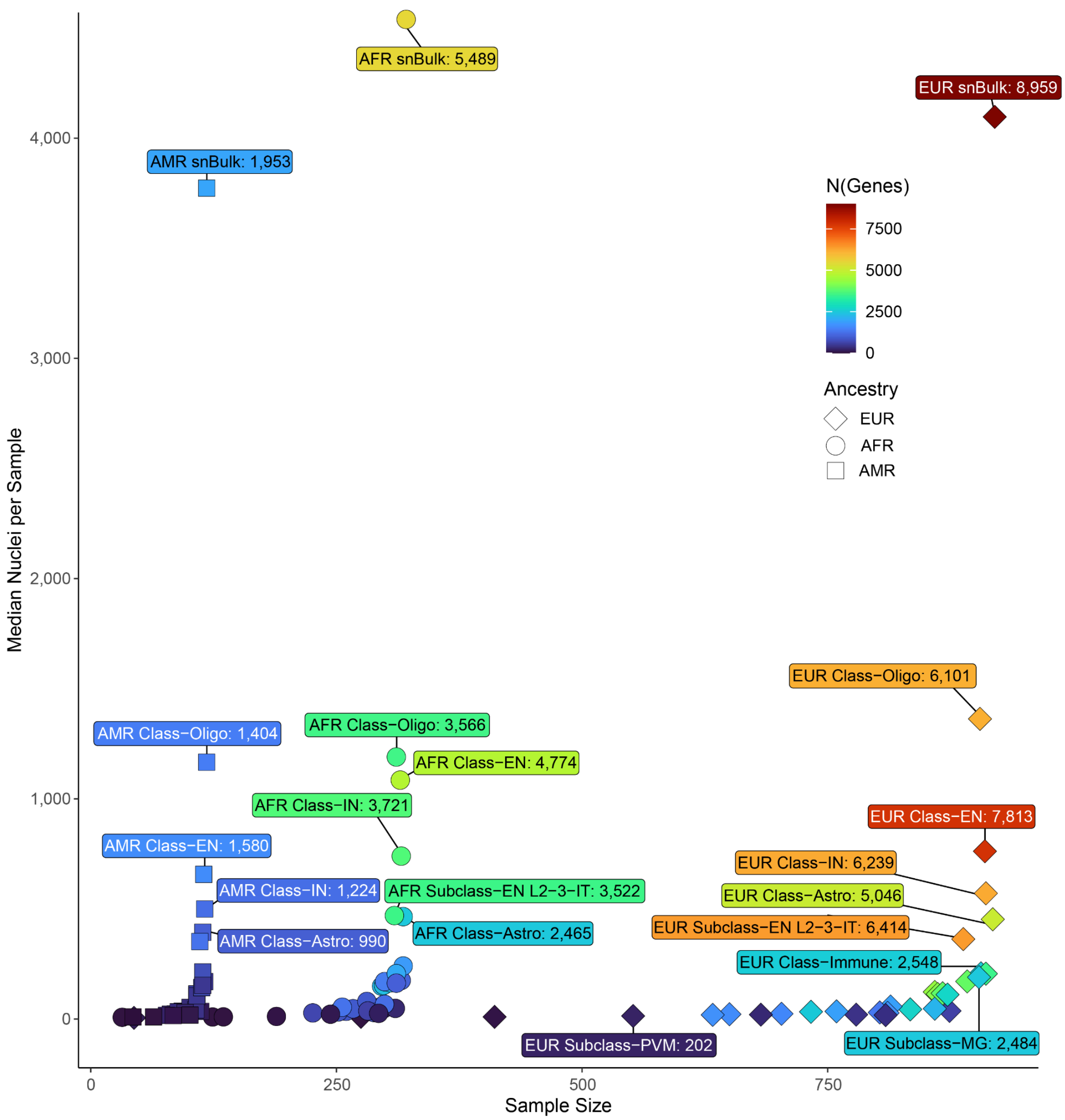

Supplementary Fig. 25 | Major predictors of number of imputable genes for single-nucleus transcriptomic imputation models (snTIMs). X-axis corresponds to the number of donors (“Sample Size”); color corresponds to the number of imputable genes; Y-axis is median number of nuclei contributing to the pseudobulk expression from every individual (“Median Nuclei per Sample”). Color indicates the number of confidently imputable genes. Shape indicates the ancestry of the snTIM. A multiple linear regression model comparing “N(Genes)” with “Sample Size” and “Median Nuclei per Sample” is significant (p-value = 1.21×10^{-22}; adjusted R^2 = 0.663; N of Imputable Genes = 3.59 × “Sample Size” + 1.26 × “Median Nuclei per Sample” − 120.95; “Sample Size” and “Median Nuclei per Sample” predictors are significant (Table S38)). Full names of all cell-types are defined in Table S3.

Supplementary Fig. 26

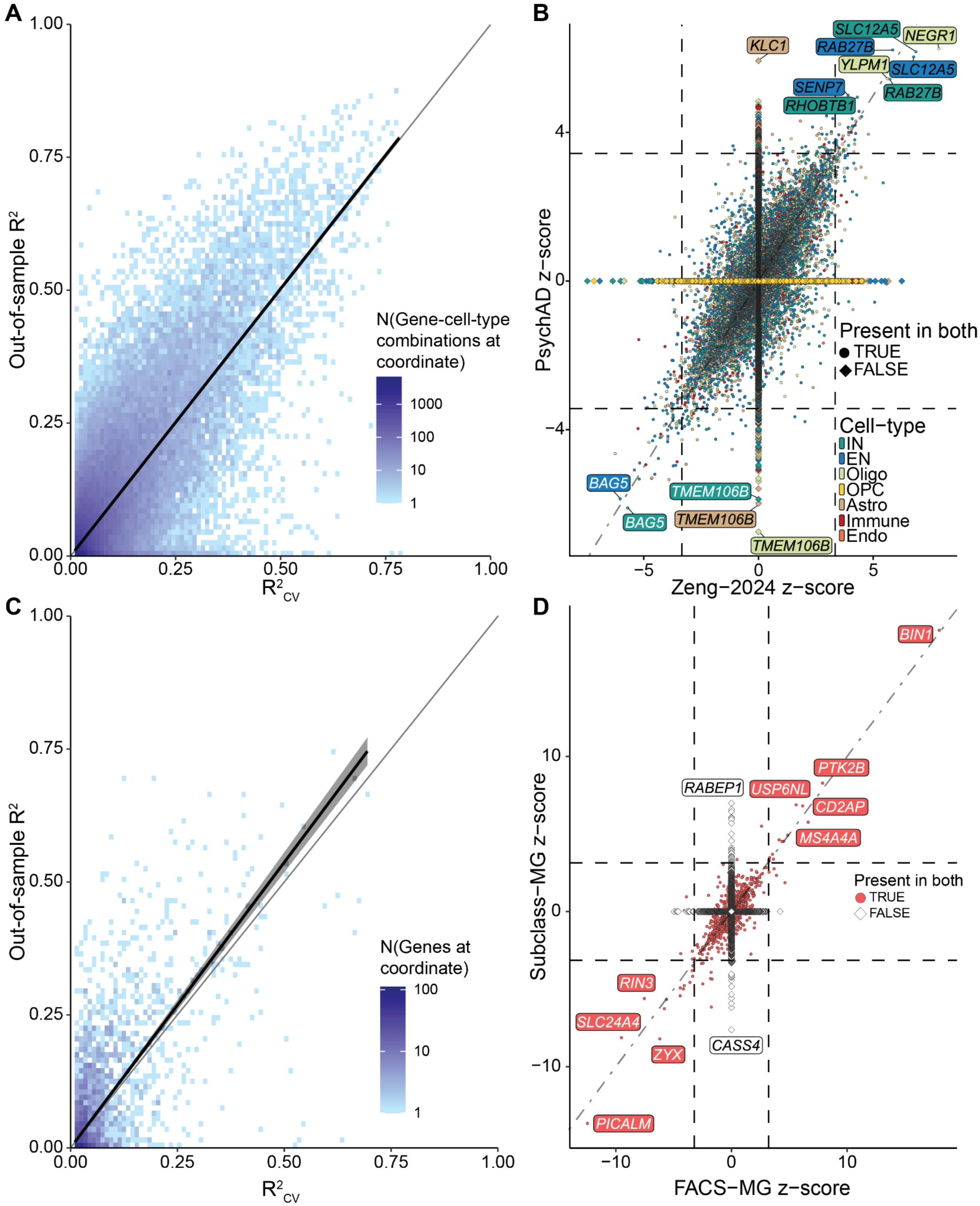

Supplementary Fig. 26 | Replication of PsychAD single-nucleus transcriptomic imputation models (snTIMs). A, Replication of PsychAD snTIMs in Religious Orders Study/Memory and Aging Project (**ROSMAP**). Binned density (0.01×0.01 bins) of cross-validation R^2 (**R^2_{CV}**) in PsychAD (x-axis) vs. out-of-sample R^2 from Pearson correlations with residualized single-nucleus RNA sequencing

(**snRNA-seq**) expression in ROSMAP (y-axis). Color indicates the number of gene-cell-type models per bin. Gray line: y = x. Black line: y = α × x fit; 95% confidence interval too narrow to visualize. Spearman's $\rho = 0.63$; $n = 70{,}156$; p-value = $3.91 \times 10^{-7,844}$. **B,** Comparison of major depressive disorder (**MDD**) summary-level single-nucleus transcriptome-wide association study **(S-snTWAS)** z-scores between Zeng-2024 (x-axis) and PsychAD snTIMs applied to the same genome-wide association study (**GWAS**) (y-axis). Cell-types were matched between studies (Table S39). Genes imputable in both TIMs are marked as points, and genes imputable in only one dataset are marked as diamonds. We observe a Pearson's $r_{(13,760)} = 0.78$, (p-value = $1.34\times10^{-2,772}$) and a Jaccard similarity index of 0.43. Full names of all cell-types are defined in Table S3. **C,** Replication of the PsychAD Subclass-Microglia (**MG**) snTIM in fluorescence-activated cell sorting-isolated microglia (**FACS-MG**). Binned density (0.01 × 0.01 bins) of R^2_{CV} (x-axis) vs. out-of-sample R^2 from residualized snRNA-seq in FACS-MG (y-axis). Gray y = x line; black y = α × x fit with 95% confidence interval (gray band). Spearman's $\rho = 0.55$; N = 1,853, p-value = 1.67×10^{-149}. **D,** Comparison of Alzheimer's disease (**AD**) S-snTWAS z-scores between FACS-MG and PsychAD Subclass-MG applied to the same GWAS. Genes imputable in both TIMs are marked as points, and genes imputable in only one dataset are marked as diamonds. We observe a Pearson's $r_{(733)} = 0.87$ (p-value = 1.32×10^{-231}) and a Jaccard similarity index of 0.23.

Supplementary Fig. 27

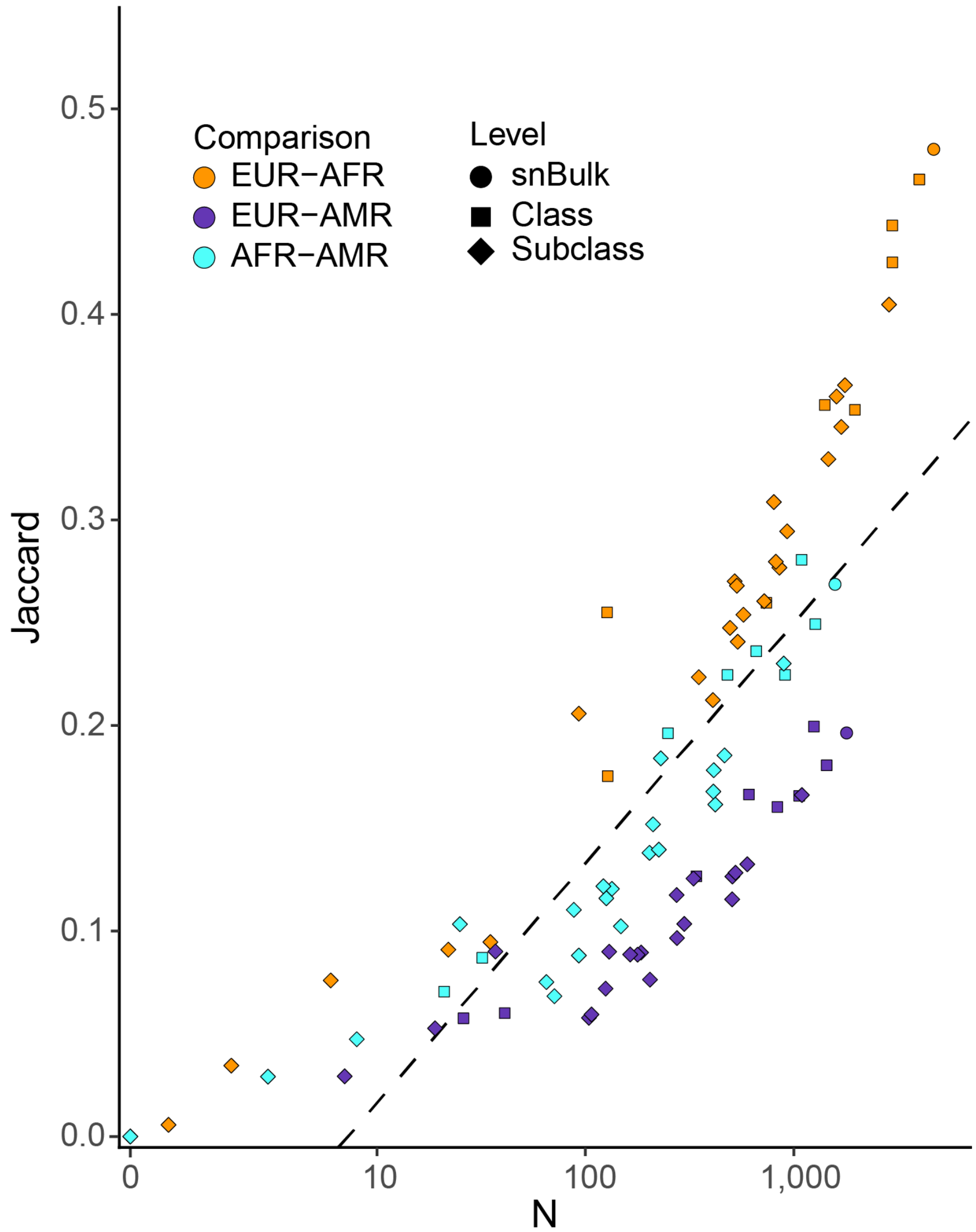

Supplementary Fig. 27 | Jaccard similarity of cross-ancestry prediction performance at different single-nucleus transcriptomic imputation models (snTIM) levels. Each point represents a pairwise analysis of cross-validation R^2 (**R^2_{cv}**) across all shared imputable genes ("N") between ancestries for the same snTIM. Applicable pairwise ancestry comparisons include individuals of European ancestry (**EUR**)-individuals of African ancestry (**AFR**), EUR-individuals of Admixed American ancestry (**AMR**) and AFR-AMR colored orange, purple and light-blue, respectively. Point shape denotes snTIM level including snBulk, Class and Subclass. The dashed line represents the linear regression line (Jaccard ~ $\log_{10}$(N)). $\log_{10}$(N) is positively associated (p-value = 1.30×10^{-21}) with the Jaccard index in a linear regression model (p-value = 1.30×10^{-21}, adjusted R^2 = 0.643).

Supplementary Fig. 28

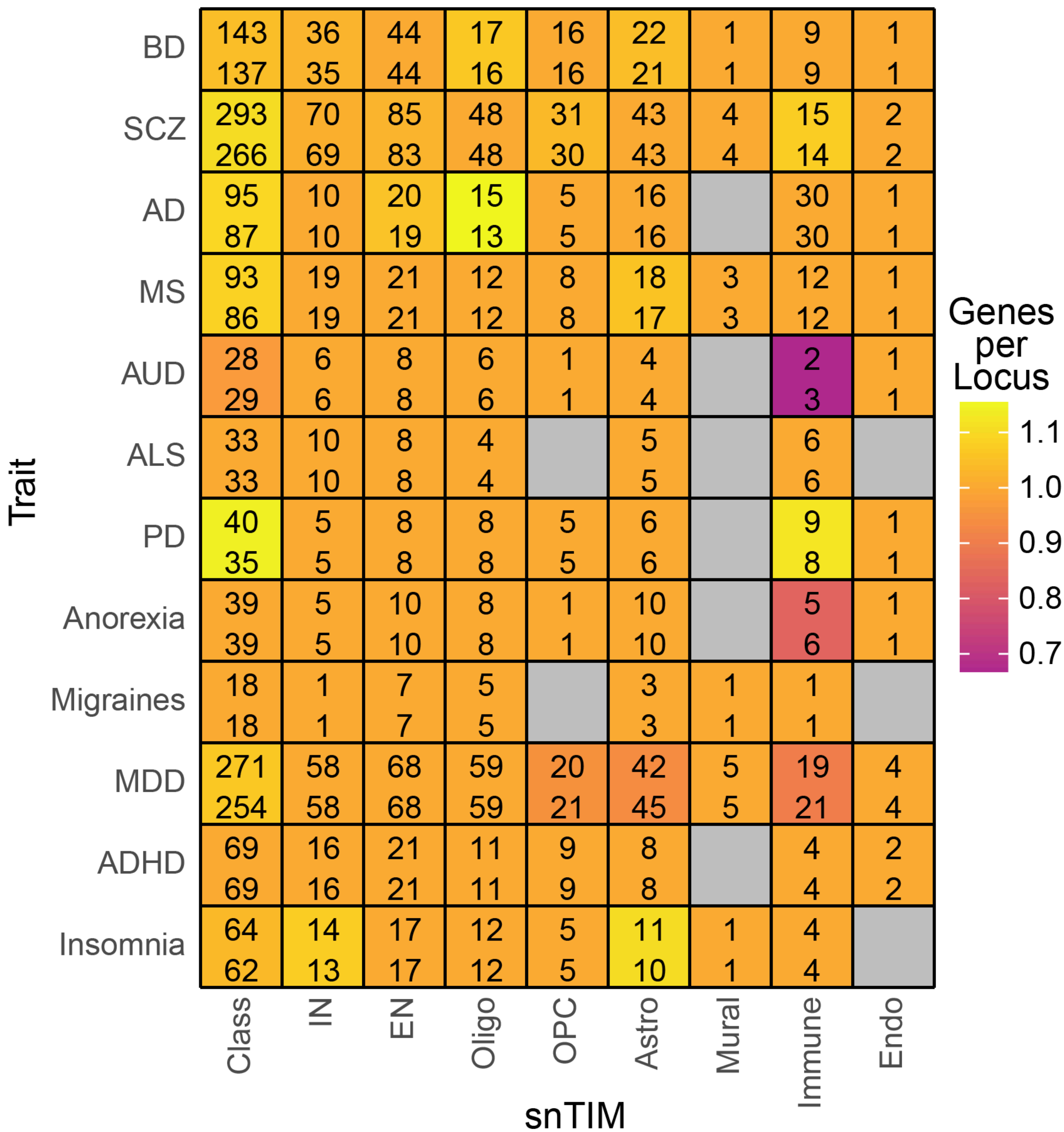

Supplementary Fig. 28 | Expanded heatmap of summary-level single-nucleus transcriptome-wide association study (S-snTWAS) multiple-cell-type gene fine-mapping. The numbers at the top and bottom of each cell indicate the count of fine-mapped genes and loci (posterior inclusion probability (**PIP**) ≥ 0.5), respectively. "Genes per Locus" indicates the ratio of these numbers. Color indicates the genes per locus for every trait-single-nucleus transcriptomic

imputation model (**snTIM**) combination. Fine-mapping was performed with Fine-mapping Of CaUsal gene Sets (**FOCUS**) while jointly considering all cell-types at the class level. Full names of all cell-types are defined in Table S3.

Supplementary Fig. 29

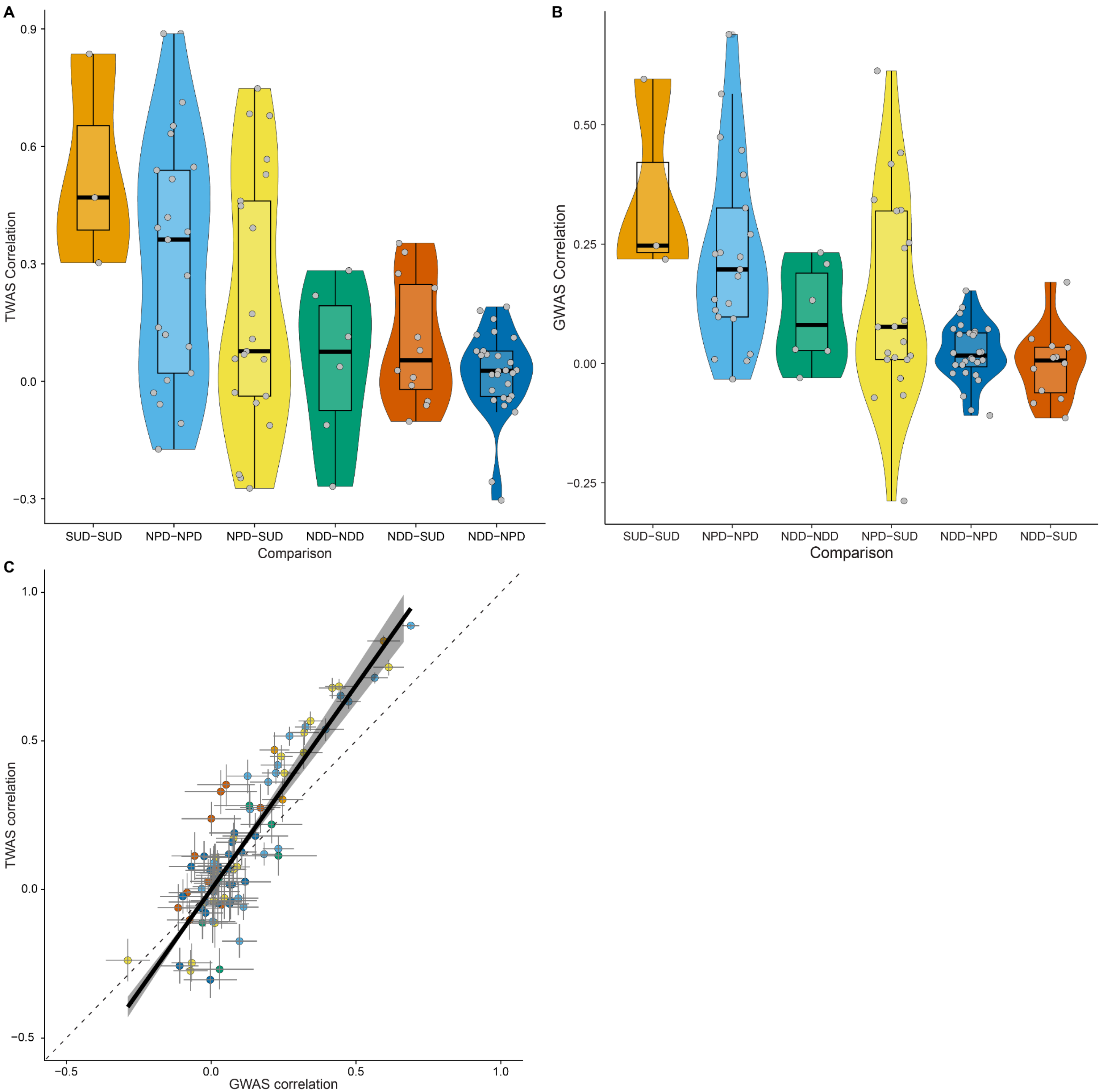

Supplementary Fig. 29 | Comparison of neuropsychiatric, neurodegenerative, and substance use disorders (NPDs, NDDs, and SUDs). A, Violin plot of summary-level single-nucleus transcriptome-wide association study **(S-snTWAS)** Pearson's correlation (y-axis) of disorders corresponding to the comparison category indicated on the x-axis. Table S12 indicates the annotated disorder category for each disorder. Internal box plot indicates the median, 25% and 75% quantiles. The vertical line ranges from median + 1.58 x IQR / sqrt(N) to median − 1.58 x IQR / sqrt(N), where IQR refers to the inter quartile range and N refers to the number of points. Violin plots have been scaled to have equivalent maximum width. **B,** Violin plot of genome-wide association study (**GWAS**)

correlation (y-axis; calculated with LD Score Regression; LD: Linkage Disequilibrium) of disorders corresponding to the comparison category indicated on the x-axis. Table S12 indicates the annotated disorder category for each disorder. Internal box plot indicates the median, 25% and 75% quantiles. The vertical line ranges from median + 1.58 x IQR / sqrt(N) to median − 1.58 x IQR / sqrt(N), where IQR refers to the inter quartile range and N refers to the number of points. Violin plots have been scaled to have equivalent maximum width. **C,** Scatterplot of GWAS correlation vs. S-snTWAS correlation. Color refers to the comparison category. Dashed line refers to the y=x line, and lines attached to each point indicate the 95% confidence interval in regards to S-snTWAS correlation (vertical) and GWAS correlation (horizontal). Black line and attached gray shaded region refer to the line of best fit (intercept set to 0) and 95% confidence interval for line of best fit, respectively. (Spearman's $\rho = 0.78$; p-value = 1.20×10^{-19}).

Supplementary Fig. 30

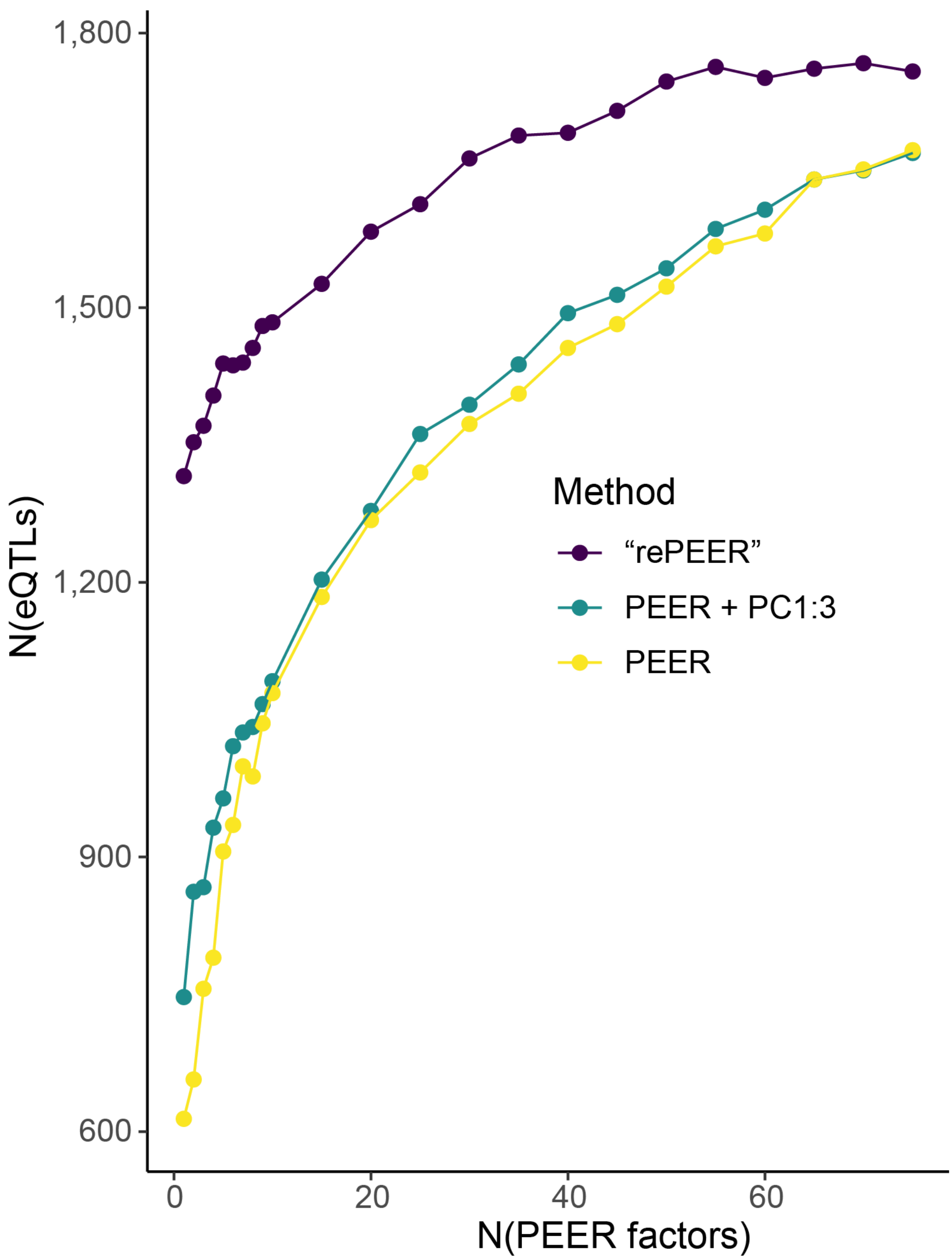

Supplementary Fig. 30 | Comparison of batch effect correction methods. To assess the impact of different batch effect correction methods on overall power, we utilized the fluorescence-activated cell sorting-isolated microglia (**FACS-MG**) cohort. "rePEER" refers to the method to perform

probabilistic estimation of expression residuals (**PEER**) on each batch separately and again altogether after aggregation of all PEER-residualized expression data. “PEER” refers to the method to just perform PEER on all expression data aggregated across all batches. “PEER+PC1:3” refers to the method to perform PEER on all expression data aggregated across all batches with genotyping principal components (**PC**) 1, 2, and 3 supplied to the PEER algorithm. Each point represents the number of significant expression quantitative trait loci (**eQTL**s) (false discovery rate (**FDR**)-adjusted p-value ≤ 0.05) found at each selected number of PEER factors used in each method (using the second round of PEER for the “rePEER” method).

Supplementary Fig. 31

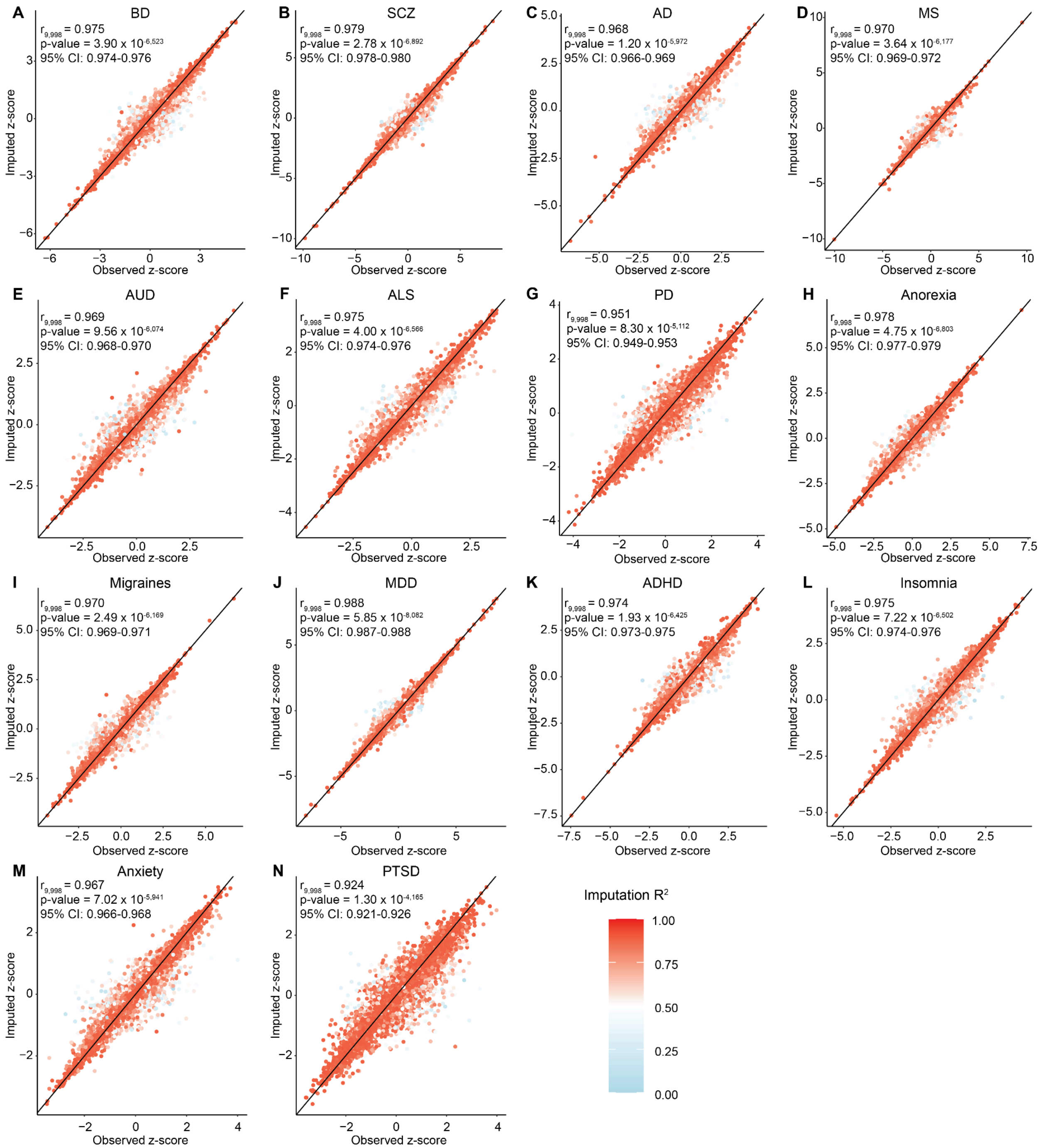

Supplementary Fig. 31 | Genome-wide association study (GWAS) imputation accuracy. We randomly selected 10,000 single nucleotide polymorphisms (**SNP**s) for each GWAS (Table S12). Only missing SNPs with imputation $R^2 \geq 0.7$ were retained for further analysis. For all selected SNPs, we performed GWAS imputation as if the SNP was missing so that we could observe the accuracy of GWAS imputation on missing data. GWAS imputation is described for each included trait: bipolar disorder (**BD**) (**A**), schizophrenia (**SCZ**) (**B**), Alzheimer's disease (**AD**) (**C**), multiple sclerosis (**MS**) (**D**), alcohol use disorder (**AUD**) (**E**), amyotrophic lateral sclerosis (**ALS**) (**F**), Parkinson's disease (**PD**) (**G**), Anorexia (**H**), Migraines (**I**), major depressive disorder (**MDD**) (**J**), attention-deficit/hyperactivity disorder (**ADHD**) (**K**), Insomnia (**L**), Anxiety (**M**), post-traumatic stress

disorder (**PTSD**) (**N**). Every plot is annotated with the Pearson's correlation coefficient (r) and two-sided p-value. The color of the point indicates the imputation R^2. Only missing SNPs with imputation $R^2 \geq 0.7$ were retained for further analysis. Black diagonal line is a reference line with a slope of 1."95% CI" indicates the 95% confidence interval.

Supplementary Fig. 32

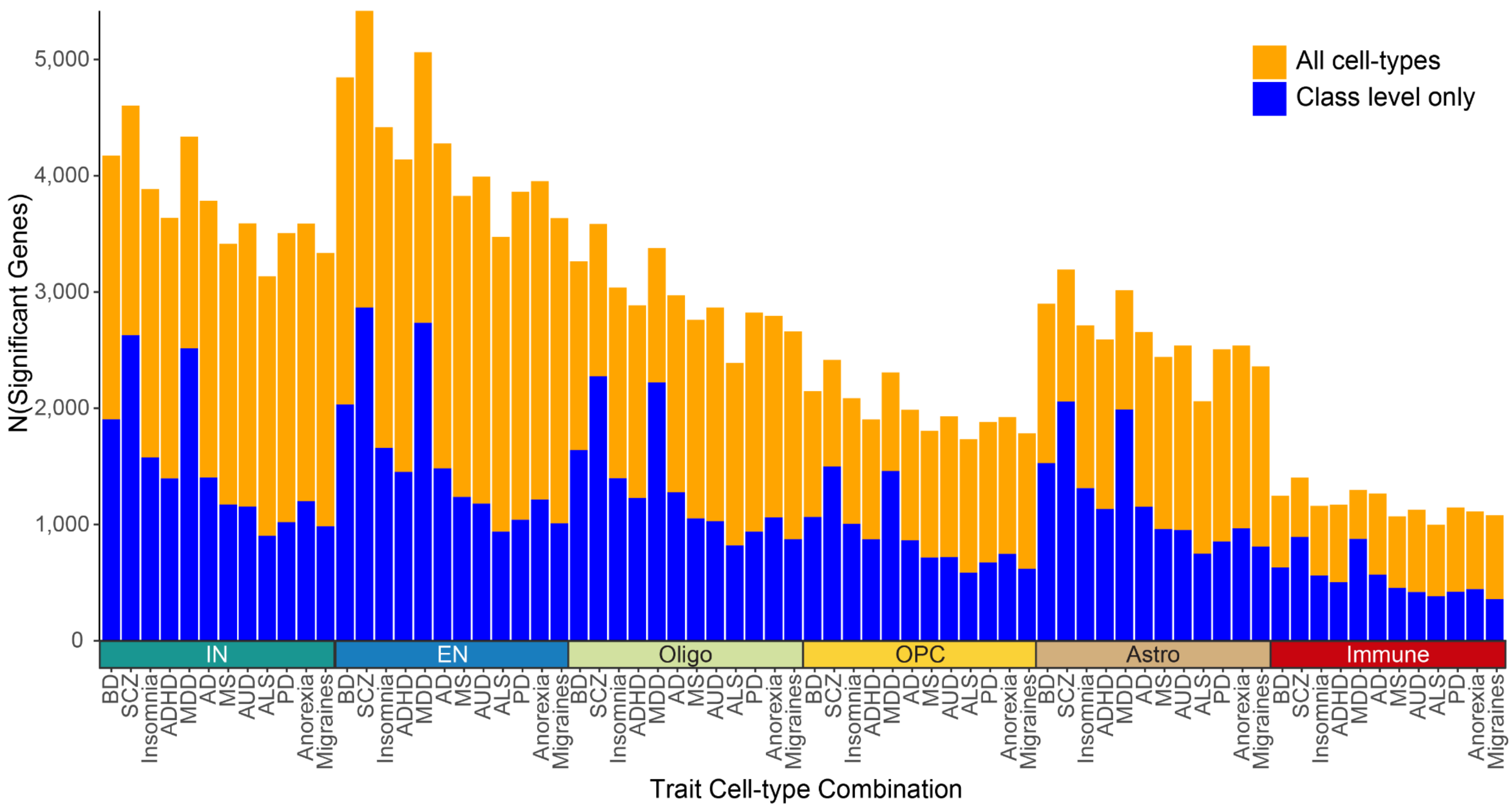

Supplementary Fig. 32 | mash fitting comparison. The blue bar indicates how many significant genes mash finds when fitted amongst the main 6 classes. The orange bar indicates how many significant genes mash finds when fitted amongst all 32 European ancestry (**EUR**) single-nucleus transcriptomic imputation models (**snTIM**s). Full names of all cell-types are defined in Table S3.

Supplementary Tables

Table S1 - Definition of all abbreviations used in the manuscript. “Abbreviation” column refers to the shortened form of the term used in the manuscript. “Full term” columns refers to the expanded form of the abbreviation. Abbreviations are split into categories for convenience.

Table S2 - Brief descriptions of all figures and tables. “Panel” and “Subpanel” columns refer to the figure/table being described. “Description” column provides a brief description of the figure/table for convenience.

Table S3 - Cell-type abbreviations used. “Abbreviation” column refers to how the cell-type is referred to within our figures and text, and is the acronym of the name in the “Long Name” column.

Table S4 - Metrics of European ancestry (EUR) single-nucleus transcriptomic imputation models (snTIMs). Each row corresponds to a different EUR snTIM (column “snTIM”; Table S3). “N(Genes)” refers to the number of confidently imputable genes in the snTIM. “%(Genes)” refers to the percentage of genes we can confidently impute out of all genes expressed in the cell-type’s residualized pseudobulk gene expression. “N(SNPs)” refers to the number of single nucleotide polymorphisms (**SNP**s) included in any of the gene prediction models in the snTIM. “Sample Size” refers to the total number of individuals used to train the snTIM. “N(PEER Factors)” refers to the final number of probabilistic estimation of expression residuals (**PEER**) factors used to residualize the data after combining the three subcohorts (Mount Sinai National Institute of Health Neurobiobank (**MSSM**), National Institute of Mental Health Intramural research program Human Brain Collection Core (**HBCC**), Rush Alzheimer’s Disease Center (**RADC**)). “N(eQTLs)” refers to the number of significant expression quantitative trait loci (**eQTL**s) found using fastQTL on the final PEER residualized gene expression. “X Sample Size” refers to the number of individuals from the X subcohort (names for each subcohort (MSSM, HBCC, RUSH) are denoted by X for brevity). “X PEER Factors” refers to the number of PEER factors used to residualize the gene expression from the X subcohort. “X eQTLs” refers to the number of significant eQTLs found using fastQTL on the X subcohort PEER residualized gene expression. “N(Cells”) refers to the total number of cells amongst all EUR individuals for the specified cell-type. “Mean(Cells per Sample)", "SD(Cells per Sample)", "Median(Cells per Sample)", "IQR(Cells per Sample)" respectively refer to the mean, standard deviation, median, and interquartile range of cells per EUR individual for the specified cell-type.

Table S5 - Metrics of African ancestry (AFR) single-nucleus transcriptomic imputation models (snTIMs). Each row corresponds to a different AFR snTIM (column “snTIM”; Table S3). “N(Genes)” refers to the number of confidently imputable genes in the snTIM. “%(Genes)” refers to the percentage of genes we can confidently impute out of all genes expressed in the cell-type’s residualized pseudobulk gene expression. “N(SNPs)” refers to the number of single nucleotide polymorphisms (**SNP**s) included in any of the gene prediction models in the snTIM. “Sample Size” refers to the total number of individuals used to train the snTIM. “N(PEER Factors)” refers to the final number of probabilistic estimation of expression residuals (**PEER**) factors used to residualize the data after combining the three subcohorts (Mount Sinai National Institute of Health Neurobiobank (**MSSM**), National Institute of Mental Health Intramural research program Human Brain Collection Core (**HBCC**), Rush Alzheimer’s Disease Center (**RADC**)). “eQTLs” refers to the number of significant expression quantitative trait loci (**eQTL**s) found using fastQTL on the final PEER residualized gene expression. “X Sample Size” refers to the number of individuals from the X subcohort (names for

each subcohort (MSSM, HBCC, RUSH) are denoted by X for brevity). “X PEER Factors” refers to the number of PEER factors used to residualize the gene expression from the X subcohort. “X eQTLs” refers to the number of significant eQTLs found using fastQTL on the X subcohort PEER residualized gene expression. “N(Cells”) refers to the total number of cells amongst all AFR individuals for the specified cell-type. “Mean(Cells per Sample)", "SD(Cells per Sample)", "Median(Cells per Sample)", "IQR(Cells per Sample)" respectively refer to the mean, standard deviation, median, and interquartile range of cells per AFR individual for the specified cell-type.

Table S6 - Metrics of Admixed American ancestry (AMR) single-nucleus transcriptomic imputation models (snTIMs). Each row corresponds to a different AMR snTIM (column “snTIM”; Table S3). “N(Genes)” refers to the number of confidently imputable genes in the snTIM. “%(Genes)” refers to the percentage of genes we can confidently impute out of all genes expressed in the cell-type’s residualized pseudobulk gene expression. “N(SNPs)” refers to the number of single nucleotide polymorphisms (**SNP**s) included in any of the gene prediction models in the snTIM. “Sample Size” refers to the total number of individuals used to train the snTIM. “N(PEER Factors)” refers to the final number of probabilistic estimation of expression residuals (**PEER**) factors used to residualize the data after combining the three subcohorts (Mount Sinai National Institute of Health Neurobiobank (**MSSM**), National Institute of Mental Health Intramural research program Human Brain Collection Core (**HBCC**), Rush Alzheimer’s Disease Center (**RADC**)). “eQTLs” refers to the number of significant expression quantitative trait loci (**eQTL**s) found using fastQTL on the final PEER residualized gene expression. “X Sample Size” refers to the number of individuals from the X subcohort (names for each subcohort (MSSM, HBCC, RUSH) are denoted by X for brevity). “X PEER Factors” refers to the number of PEER factors used to residualize the gene expression from the X subcohort. “X eQTLs” refers to the number of significant eQTLs found using fastQTL on the X subcohort PEER residualized gene expression. “N(Cells”) refers to the total number of cells amongst all AMR individuals for the specified cell-type. “Mean(Cells per Sample)", "SD(Cells per Sample)", "Median(Cells per Sample)", "IQR(Cells per Sample)" respectively refer to the mean, standard deviation, median, and interquartile range of cells per AMR individual for the specified cell-type.

Table S7 - European ancestry (EUR) single-nucleus transcriptomic imputation models (snTIMs) gene-cell-type combinations. Each row refers to a different confidently imputable gene-cell-type combination in EUR snTIMs. "SnTIM" column refers to the snTIM in question (Table S3). **“**Gene” refers to the Ensembl gene ID for the gene. “Gene name” refers to the Human Genome Organization (**HUGO**) Gene Nomenclature Committee (**HGNC**) gene symbol for the gene. "Chromosome" refers to the chromosome the gene lies on. “Window start” refers to the GRCh38 base position on the chromosome used for the start of the window for model training. “Window end” refers to the GRCh38 base position on the chromosome used for the end of the window for model training. “N(SNPs in window)” refers to the number of single nucleotide polymorphisms (**SNP**s) that were present in the window for model training for the gene-cell-type combination. “N(SNPs in model)” refers to the number of SNPs used to impute the gene-cell-type combination. “CV R2” refers to the cross-validation R^2 (**R^2_{CV}**) found for the particular gene-cell-type combination. “CV p-value” refers to the cross-validation p-value found for the particular gene-cell-type combination. “CV FDR” refers to the false discovery rate (**FDR**)-corrected cross-validation p-value found for the particular gene-cell-type combination.

Table S8 - African ancestry (AFR) single-nucleus transcriptomic imputation models (snTIMs) gene-cell-type combinations. Each row refers to a different confidently imputable gene-cell-type

combination in AFR snTIMs. The "SnTIM" column refers to the snTIM in question (Table S3). **“**Gene” refers to the Ensembl gene ID for the gene. “Gene name” refers to the Human Genome Organization (**HUGO**) Gene Nomenclature Committee (**HGNC**) gene symbol for the gene. "Chromosome" refers to the chromosome the gene lies on. “Window start” refers to the GRCh38 base position on the chromosome used for the start of the window for model training. “Window end” refers to the GRCh38 base position on the chromosome used for the end of the window for model training. “N(SNPs in window)” refers to the number of single nucleotide polymorphisms (**SNP**s) that were present in the window for model training for the gene-cell-type combination. “N(SNPs in model)” refers to the number of SNPs used to impute the gene-cell-type combination. “CV R2” refers to the cross-validation R^2 ($\mathbf{R^2_{cv}}$) found for the particular gene-cell-type combination. “CV p-value” refers to the cross-validation p-value found for the particular gene-cell-type combination. “CV FDR” refers to the false discovery rate (**FDR**)--corrected cross-validation p-value found for the particular gene-cell-type combination.

Table S9 - Admixed American ancestry (AMR) single-nucleus transcriptomic imputation models (snTIMs) gene-cell-type combinations. Each row refers to a different confidently imputable gene-cell-type combination in AMR snTIMs. "SnTIM" column refers to the snTIM in question (Table S3). **“**Gene” refers to the Ensembl gene ID for the gene. “Gene name” refers to the Human Genome Organization (**HUGO**) Gene Nomenclature Committee (**HGNC**) gene symbol for the gene. "Chromosome" refers to the chromosome the gene lies on. “Window start” refers to the GRCh38 base position on the chromosome used for the start of the window for model training. “Window end” refers to the GRCh38 base position on the chromosome used for the end of the window for model training. “N(SNPs in window)” refers to the number of single nucleotide polymorphisms (**SNP**s) that were present in the window for model training for the gene-cell-type combination. “N(SNPs in model)” refers to the number of SNPs used to impute the gene-cell-type combination. “CV R2” refers to the cross-validation R^2 ($\mathbf{R^2_{cv}}$) found for the particular gene-cell-type combination. “CV p-value” refers to the cross-validation p-value found for the particular gene-cell-type combination. “CV FDR” refers to the false discovery rate (**FDR**)-corrected cross-validation p-value found for the particular gene-cell-type combination.

Table S10 - Number of genes in European ancestry (EUR) single-nucleus transcriptomic imputation models (snTIMs). Each row corresponds to a different EUR snTIM or an aggregate across all elements of that “Level” (Table S3). “N(Total Genes)” refers to the total number of imputable coding genes in that snTIM. “N(R2 Preferred)” refers to the total number of coding genes in that snTIM that outperforms all other EUR snTIMs (based on cross-validation R^2 ($\mathbf{R^2_{cv}}$)). “N(Unique)” refers to the total number of coding genes in that snTIM that are only imputed in that snTIM (vs. all other EUR snTIMs).

Table S11 - Correlation and Jaccard similarity of gene prediction models in each cell-type across ancestries. The "snTIM" column indicates the cell-type single-nucleus transcriptomic imputation models (**snTIM**) investigated in each specified ancestry (Table S3). “Level” refers to the level of the cell-type hierarchy the specified cell-type is a part of (Extended Data Fig. 2). The “Comparison” column indicates the combination of ancestries investigated in the statistics described in the row. “Jaccard” indicates the Jaccard similarity index of the confidently imputed genes in the cell-type snTIMs of each compared ancestry. “Correlation” indicates the Spearman’s correlation of the cross-validation R^2 ($\mathbf{R^2_{cv}}$) across all confidently imputed genes in both ancestries in the specified

cell-type. “Correlation p-value” indicates the p-value of the aforementioned correlation. “N” indicates the number of shared confidently imputed genes in both ancestries in the specified cell-type.

Table S12 - Genome-wide association study (GWAS) summary statistics utilized and the sample size of the matched phenotypes in the Million Veteran Program (MVP). “Name” column refers to how the trait is referred to within our figures and text, and is the acronym of the name in the “Long Name” column. The “PMID” column refers to the relevant publication the GWAS is taken from, and the relevant publication is also cited in the text. All sample sizes reflect the relevant population in the Million Veteran Program. PheCodes were mapped to each GWAS phenotype manually based on the phenome-wide association study (**PheWAS**) catalog (https://phewascatalog.org/). “Category” refers to whether the trait was classified as a neuropsychiatric disorder, neurodegenerative disorder, or substance use disorder (**NPD**, **NDD**, or **SUD**) for the analysis in Supplementary Note 7.

Table S13 - Gene set enrichment analysis of clinically relevant genes in each trait and cell-type hierarchy level. The “Trait” column refers to the genome-wide association study (**GWAS**) used for each transcriptome-wide association study (**TWAS**) (Table S18). The “p-value” column refers to the uncorrected p-value for the gene set enrichment analysis. “OR” refers to the odds ratio for the gene set enrichment analysis. “Intersection” refers to the number of common elements between the unique significant genes in the specified transcriptome wide association study (significant at false discovery rate (**FDR**)-adjusted p-value ≤ 0.05) and the list of clinically relevant genes. “Union” refers to the number of total elements in the union of the unique significant genes in the transcriptome wide association study (significant at FDR-adjusted p-value ≤ 0.05) and the list of clinically relevant genes. “Jaccard” refers to the Jaccard similarity index between the unique significant genes in the transcriptome wide association study (significant at FDR-adjusted p-value ≤ 0.05) and the list of clinically relevant genes. “snTIM” refers to the single-nucleus transcriptomic imputation model (**snTIM**) used in the row’s analysis (Bulk, snBulk, Class, Subclass; Table S3). “FDR” refers to the FDR-adjusted p-value for the gene-set enrichment analysis. “OR lowCI” is the odds ratio at the low end of the 95% confidence interval for the gene set enrichment analysis. “OR highCI” is the odds ratio at the high end of the 95% confidence interval for the gene set enrichment analysis.

Table S14 - Bulk summary-level transcriptome-wide association study (S-TWAS) across all specified traits. Each row corresponds to a confidently imputable gene in the Bulk S-TWAS analysis. **“**Gene” refers to the Ensembl gene ID for the gene. “Gene name” refers to the Human Genome Organization (**HUGO**) Gene Nomenclature Committee (**HGNC**) gene symbol for the gene. The “Trait” column refers to the genome-wide association study (**GWAS**) used for each S-TWAS (Table S12). The “z-score” and “p-value” column correspond to the calculated z-score and p-value from SPrediXcan, respectively. The “CV R2” and “CV p-value” columns refer to the TIM’s cross-validation scores for each gene. “N(SNPs in Model)” and “N(SNPs used)” refer to the number of model single nucleotide polymorphisms (**SNP**s), and the number of model SNPs available in the GWAS (after attempting to impute missing SNPs) respectively. “FDR” refers to the false discovery rate (**FDR**)-adjusted p-value for SPrediXcan.

Table S15 - Multi-marker Analysis of GenoMic Annotation (MAGMA) analysis across all specified traits. Each row corresponds to a different gene-trait association (**GTA**) identified by the MAGMA analysis. **“**Gene” refers to the Ensembl gene ID for the gene. “Gene name” refers to the Human Genome Organization (**HUGO**) Gene Nomenclature Committee (**HGNC**) gene symbol for the gene. The “Trait” column refers to the genome-wide association study (**GWAS**) used for each transcriptome-wide association study (**TWAS**) (Table S12). "Chromosome" refers to the chromosome

each gene is on. “Start” and “Stop” refer to the start and stop position (GRCh38) of the window used for MAGMA analysis. “N(SNPs)” refers to the number of quality-controlled single nucleotide polymorphisms (**SNP**s) annotated to the gene found in the data. “N(Param)” refers to the number of parameters that MAGMA’s SNP2GENE function included in the model. “N” refers to the sample size used to analyze the GTA. “z-score” and “p-value” refer to the output statistics from MAGMA’s SNP2GENE, and “FDR” represents the across-trait false discovery rate (**FDR**)-adjusted p-value.

Table S16 - Fisher’s exact test enrichment of significant and novel gene-trait associations (GTAs) in class and subclass single-nucleus transcriptomic imputation models (snTIMs) vs. snBulk. Every row corresponds to a unique combination of trait and cell-type hierarchy level (excluding Bulk and snBulk, which are used in the comparison) to test for enrichment of novel genes. The “Trait” column refers to the genome-wide association study (**GWAS**) used for each transcriptome-wide association study (**TWAS**) (Table S12). “OR” refers to the odds ratio for Fisher's exact test (number of novel genes at the specified cell-type hierarchy level vs. the number of novel genes in snBulk), and “p-value” refers to the p-value for the same test. “FDR” refers to the false discovery rate (**FDR**)-adjusted p-value for the Fisher’s exact test. “snTIM” refers to the level of the cell-type hierarchy used for the Fisher’s exact test (Extended Data Fig. 2; Table S3). “lowCI” is the odds ratio at the low end of the 95% confidence interval for Fisher's exact test. “highCI” is the odds ratio at the high end of the 95% confidence interval for Fisher's exact test. “FinerCT Novel” refers to the number of novel GTAs at the specified level of the cell-type hierarchy. “snBulk Novel” refers to the number of novel GTAs in snBulk. “FinerCT Known” refers to the number of non-novel GTAs at the specified level of the cell-type hierarchy. “snBulk Known” refers to the number of non-novel GTAs in snBulk.

Table S17 - Fisher’s exact test enrichment of significant and novel gene-trait associations (GTAs) in finer cell-type single-nucleus transcriptomic imputation models (snTIMs) vs. snBulk. Every row corresponds to a unique combination of trait and cell-type hierarchy level (excluding Bulk and snBulk, which are used in the comparison) to test for enrichment of novel genes. The “Trait” column refers to the genome-wide association study (**GWAS**) used for each transcriptome-wide association study (**TWAS**) (Table S12). “OR” refers to the odds ratio for Fisher's exact test (number of novel genes at the specified cell-type hierarchy level vs. the number of novel genes in snBulk), and “p-value” refers to the p-value for the same test. “FDR” refers to the false discovery rate (**FDR**)-adjusted p-value for the Fisher’s exact test. “snTIM” refers to the level of the cell-type hierarchy used for the Fisher’s exact test (Extended Data Fig. 2; Table S3). “lowCI” is the odds ratio at the low end of the 95% confidence interval for Fisher's exact test. “highCI” is the odds ratio at the high end of the 95% confidence interval for Fisher's exact test. “FinerCT Novel” refers to the number of novel GTAs at the specified level of the cell-type hierarchy. “snBulk Novel” refers to the number of novel GTAs in snBulk. “FinerCT Known” refers to the number of non-novel GTAs at the specified level of the cell-type hierarchy. “snBulk Known” refers to the number of non-novel GTAs in snBulk.

Table S18 - Number of individual-cell-type fine-mapped genes and loci in summary-level transcriptome-wide association study (S-snTWAS) analysis. Each row corresponds to a unique combination of trait and single-nucleus transcriptomic imputation model **(snTIM)** (or the aggregate across multiple snTIMs). “All-Aggregate” refers to the aggregate of all European ancestry (**EUR**) snTIMs for that trait. “Class-Aggregate” refers to the aggregate of all 8 EUR class-level snTIMs. “Subclass-Aggregate” refers to the aggregate of all 23 EUR subclass-level snTIMs and the 4 EUR class-level snTIMs that represent endpoints for cell-type division (Extended Data Fig. 2). Aggregate

categories look at the number of unique fine-mapped genes and loci (posterior inclusion probability (**PIP**) ≥ 0.5), and significant genes. The “Trait” column refers to the genome-wide association study (**GWAS**) used for each TWAS (Table S12). The "snTIM" column refers to the EUR snTIMs (Table S3). “N(fine-mapped genes)” refers to the number of unique fine-mapped (PIP ≥ 0.5) genes in that trait-snTIM combination. “N(fine-mapped loci)” refers to the number of unique fine-mapped (PIP of at least one gene ≥ 0.5) loci in that trait-snTIM combination. “N(significant genes)” refers to the number of unique genes in that trait-snTIM combination from the S-snTWAS analysis.

Table S19 - Classification of cell-type-specific gene-trait associations (GTAs) determined by mash algorithm. Each row quantifies the number of cell-type-specific GTAs in the cell-types listed (column “snTIMs”; Table S3), and whether those GTAs are significant in the snBulk summary-level transcriptome-wide association study **(S-snTWAS)**. For rows where the “snTIMs” listed are “ALL”, the number of GTAs specific to the number of cell-types specified in the “N(snTIMs)” column are listed. For rows where both “snTIMs” and “N(snTIMs)” are “ALL”, the number indicates the number of cell-type-specific GTAs across all trait-specific mash analyses. Finally, the number of S-snTWAS significant genes for each trait are listed in the “Total S-snTWAS Significant” column, for reference.

Table S20 - All mash results. Each row describes a summary-level transcriptome-wide association study **(S-snTWAS)** significant gene-trait association (**GTA**) and its mash probabilities. mash is fit across the 6 major European ancestry (**EUR**) single-nucleus transcriptomic imputation models **(snTIMs)**, and each cell-type column indicates the p-value that the GTA is significant in that cell-type (Extended Data Fig. 2; Table S3). “NA” values indicate that the gene was not confidently imputed in that cell-type. “Gene name” is the Human Genome Organization (**HUGO**) Gene Nomenclature Committee (**HGNC**) symbol for that gene. The “Trait” column refers to the genome-wide association study (**GWAS**) used for each TWAS (Table S12). The “Significant In” column indicates which cell-type(s) the GTA is present in according to the mash fitting. The “Not significant In” column indicates which cell-type(s) the GTA is not present in according to the mash fitting. The “N(Significant snTIMs)” column indicates the number of cell-types the GTA is present in according to the mash fitting. The “N(Not significant snTIMs)” column indicates the number of cell-types the GTA is not present in according to the mash fitting. The “Combinatorial Probability” column indicates the combined probability that the GTA is present in at least one of the “Significant in” cell-types and none of the “Not significant in” cell-types. The “Significant” column indicates that the GTA was significant in at least one of the 6 chosen cell-types according to the mash fitting.

Table S21 - Comparison of SPrediXcan using MVP variant associations vs. individual-level transcriptome-wide association studies (I-snTWAS). “Comparison” details the value used for Pearson’s correlation. “df” is the degrees of freedom from the Pearson’s correlation. “Pearson’s correlation”, “p-value”, “lowCI”, ”highCI” are the Pearson’s correlation estimate, p-value, lower 95% confidence interval and higher 95% confidence interval, respectively.

Table S22 - All cell-type heterogeneity analysis results. Each row corresponds to a summary-level transcriptome-wide association study **(S-snTWAS)** significant gene-trait association (**GTA**). **“**Gene” refers to the Ensembl gene ID for the gene. “Gene name” refers to the Human Genome Organization (**HUGO**) Gene Nomenclature Committee (**HGNC**) gene symbol for the gene. The “N(snTIMs)” column indicates how many European ancestry (**EUR**) single-nucleus transcriptomic imputation models **(snTIM**s**)** the gene can be confidently imputed in. The “Trait” column refers to the genome-wide association study (**GWAS**) used for each TWAS (Table S12). The “Q p-value” and “Q FDR” indicate the p-value and false discovery rate (**FDR**)-adjusted p-value, respectively, from the Cochran’s Q

computation across all cell-types for each GTA. The “I2”, “I2 lowCI” and “I2 highCI” represent the I^2 statistic calculated from the Q statistic, and the lower and upper bound of its 95% confidence interval.

Table S23 - Multiscale Embedded Gene Co-expression Network Analysis (MEGENA) module membership. Each row corresponds to a gene that is a member of at least one of the three MEGENA modules (M947, M842, M406), significantly enriched for Class-Immune summary-level transcriptome-wide association study **(S-snTWAS)** genes (false discovery rate (**FDR**)-adjusted p-value ≤ 0.05). The first 7 columns correspond to gene descriptors (Ensembl gene ID, chromosome, start, stop, strand, Human Genome Organization (**HUGO**) Gene Nomenclature Committee (**HGNC**) gene symbol, gene type). The next three columns describe to which module the gene belongs.. The final 12 columns correspond to z-score, p-value, and FDR-adjusted p-value for the gene in various comparisons (Class-Immune S-snTWAS, or differentially expressed genes for clinical dementia rating (**CDR**), Braak, β-amyloid).

Table S24 - Pathway dictionary for Extended Data Fig. 4C. “Original Name” corresponds to the original name of pathways. “Publication Name” corresponds to the name of the pathway used in Extended Data Fig. 4C.

Table S25 - Shared significant gene-cell-type associations from summary-level transcriptome-wide association study (S-snTWAS) in each pair of traits. “Trait1” and “Trait2” columns represent the two traits being compared in each row. The “p-value” column refers to the uncorrected p-value for Fisher's exact test. “OR” refers to the odds ratio for Fisher's exact test. The “FDR” column refers to the false discovery rate (**FDR**)-adjusted p-value. “lowCI” is the odds ratio at the low end of the 95% confidence interval for Fisher's exact test. “highCI” is the odds ratio at the high end of the 95% confidence interval for Fisher's exact test. “N(Significant in Both)” represents the gene-cell-type combinations significant in both “Trait1” and “Trait2”. “N(Significant only in Trait1)” represents the gene-cell-type combinations significant only in “Trait1”. “N(Significant only in Trait2)” represents the gene-cell-type combinations significant only in “Trait2”. “Not Significant” represents the gene-cell-type combinations not significant in either trait.

Table S26 - Shared significant genes from summary-level transcriptome-wide association study (S-snTWAS) in each pair of traits. “Trait1” and “Trait2” columns represent the two traits being compared in each row. The “p-value” column refers to the uncorrected p-value for Fisher's exact test. “OR” refers to the odds ratio for Fisher's exact test. The “FDR” column refers to the false discovery rate (**FDR**)-adjusted p-value. “OR lowCI” is the odds ratio at the low end of the 95% confidence interval for Fisher's exact test. “OR highCI” is the odds ratio at the high end of the 95% confidence interval for Fisher's exact test. “N(Significant in Both)” represents the genes (regardless of cell-type) significant in both “Trait1” and “Trait2”. “N(Significant only in Trait1)” represents the genes significant only in “Trait1”. “N(Significant only in Trait2)” represents the genes significant only in “Trait2”. “Not Significant” represents the genes not significant in either trait.

Table S27 - Shared significant pathway-cell-type associations from summary-level transcriptome-wide association study (S-snTWAS) pathway analysis in each pair of traits. “Trait1” and “Trait2” columns represent the two traits being compared in each row. The “p-value” column refers to the uncorrected p-value for Fisher's exact test. “OR” refers to the odds ratio for Fisher's exact test. The “FDR” column refers to the false discovery rate (**FDR**)-adjusted p-value. “OR lowCI” is the odds ratio at the low end of the 95% confidence interval for Fisher's exact test. “OR highCI” is the odds ratio at the high end of the 95% confidence interval for Fisher's exact test. “N(Significant in Both)” represents the pathway-cell-type combinations significant in both “Trait1” and

“Trait2”. “N(Significant only in Trait1)” represents the pathway-cell-type combinations significant only in “Trait1”. “N(Significant only in Trait2)” represents the pathway-cell-type combinations significant only in “Trait2”. “Not Significant” represents the pathway-cell-type combinations not significant in either trait.

Table S28 - Shared significant pathway associations from summary-level transcriptome-wide association study (S-snTWAS) pathway analysis in each pair of traits. “Trait1” and “Trait2” columns represent the two traits being compared in each row. The “p-value” column refers to the uncorrected p-value for Fisher's exact test. “OR” refers to the odds ratio for Fisher's exact test. The “FDR” column refers to the false discovery rate (**FDR**)-adjusted p-value. “OR lowCI” is the odds ratio at the low end of the 95% confidence interval for Fisher's exact test. “OR highCI” is the odds ratio at the high end of the 95% confidence interval for Fisher's exact test. “N(Significant in Both)” represents the pathways (regardless of cell-type) significant in both “Trait1” and “Trait2”. “N(Significant only in Trait1)” represents the pathways significant only in “Trait1”. “N(Significant only in Trait2)” represents the pathways significant only in “Trait2”. “Not Significant” represents the pathways not significant in either trait.

Table S29 - In-depth explanation of the number of shared pathways in each pair of traits in each cell-type from summary-level transcriptome-wide association study (S-snTWAS) pathway analysis. Each row corresponds to a different cell-type-trait-trait combination. The column “N(Shared Pathways in snTIM)” describes the number of shared pathways between the two specified traits in the cell-type specified. “N(Shared Pathways)” describes the total number of shared pathways between the two traits. “P(Shared Pathways in snTIM)” is the percentage of “N(Shared Pathways in snTIM)” over “N(Shared Pathways)”. snTIM: single-nucleus transcriptomic imputation model**.**

Table S30 - Multi-ancestry fine-mapping of individual-level transcriptome-wide association studies (I-snTWAS) significant gene-trait associations (GTAs). Each row corresponds to a different significant I-snTWAS gene-cell-type-trait linkage disequilibrium (**LD**) block combination. “Gene name” describes the Human Genome Organization (**HUGO**) Gene Nomenclature Committee (**HGNC**) gene symbol.The “LD Block” column refers to the LD block for which the posterior inclusion probability is specified. Because a gene can stretch over multiple LD blocks, there is a uniquely calculated value for each LD block a gene exists in. The "X PIP" column represents the posterior inclusion probability (**PIP**) calculated by FOCUS, where "X" denotes the ancestry in which the PIP was computed ("EUR" for European, "AFR" for African, and "MA" for multi-ancestry). The “Trait” column refers to the genome-wide association study (**GWAS**) used for each TWAS (Table S12). The "snTIM" column refers to the single-nucleus transcriptomic-imputation model (**snTIM**) used across ancestries (Table S3). “The I-snTWAS Significance” column corresponds to the ancestry in which the gene-cell-type trait combination was significant.

Table S31 - Single-nucleus genetically regulated gene expression phenome-wide association study (snGReX-PheWAS) heterogeneity. Each row describes heterogeneity amongst every significant gene-trait association from snGReX-PheWAS analysis (significant in at least 1 cell-type). “Ancestry” indicates the ancestry in which the gene-trait association was significant and the ancestry where heterogeneity was investigated. “Gene name” describes the Human Genome Organization (**HUGO**) Gene Nomenclature Committee (**HGNC**) gene symbol. “Phecode number” and “Phecode name” describe the number and definition of the phecode from the PheWAS catalog (https://phewascatalog.org/) for the phecode. “N(snTIMs)” describes the number of ancestry specific single-nucleus transcriptomic imputation models **(snTIMs)** the gene is confidently imputable in (Table

S3). The "Q p-value" and "Q FDR" indicate the p-value and false discovery rate (**FDR**)-adjusted p-value, respectively, from the Cochran's Q computation across all cell-types for each gene-trait association (**GTA**). The "I2", "I2 lowCI" and "I2 highCI" represent the I^2 statistic calculated from the Q statistic, and the lower and upper bound of its 95% confidence interval. "Cases" and "Controls" describe the number of cases and controls within the specified ancestry, respectively, for the relevant phecode. "Phenotype Category" describes the overall category for the phecode. Categories are mostly extracted from the PheWAS catalog, with some additional manual curation.

Table S32 - Confidently imputable genes in African ancestry (AFR) single-nucleus transcriptomic imputation models (snTIMs) that cannot be confidently imputed in European ancestry (EUR) snTIMs. Each entry corresponds to the Human Genome Organization (**HUGO**) Gene Nomenclature Committee (**HGNC**) gene symbol for the gene.

Table S33 - Confidently imputable genes in Admixed American ancestry (AMR) single-nucleus transcriptomic imputation models (snTIMs) that cannot be confidently imputed in European ancestry (EUR) snTIMs. Each entry corresponds to the Human Genome Organization (**HUGO**) Gene Nomenclature Committee (**HGNC**) gene symbol for the gene.

Table S34 - Confidently imputable genes in European ancestry (EUR) single-nucleus transcriptomic imputation models (snTIMs) that cannot be confidently imputed in African ancestry (AFR) snTIMs. Each entry corresponds to the Human Genome Organization (**HUGO**) Gene Nomenclature Committee (**HGNC**) gene symbol for the gene.

Table S35 - Confidently imputable genes in Admixed American ancestry (AMR) single-nucleus transcriptomic imputation models (snTIMs) that cannot be confidently imputed in African ancestry (AFR) snTIMs. Each entry corresponds to the Human Genome Organization (**HUGO**) Gene Nomenclature Committee (**HGNC**) gene symbol for the gene.

Table S36 - Confidently imputable genes in European ancestry (EUR) single-nucleus transcriptomic imputation models (snTIMs) that cannot be confidently imputed in Admixed American ancestry (AMR) snTIMs. Each entry corresponds to the Human Genome Organization (**HUGO**) Gene Nomenclature Committee (**HGNC**) gene symbol for the gene.

Table S37 - Confidently imputable genes in African ancestry (AFR) single-nucleus transcriptomic imputation models (snTIMs) that cannot be confidently imputed in Admixed American ancestry (AMR) snTIMs. Each entry corresponds to the Human Genome Organization (**HUGO**) Gene Nomenclature Committee (**HGNC**) gene symbol for the gene.

Table S38 - Major predictors of the number of confidently imputable genes in European ancestry (EUR) snTIMs. Columns "Effect size", "SE", "t-value", and "p-value" indicate the regression coefficient, standard error, t-test statistic and p-value for the association (Number of confidently imputable coding genes ~ Sample Size + Median Nuclei per Sample; linear regression). The "Predictor" column indicates the regression term described in the row. "Sample Size" refers to the number of donors in each snTIM (Table S3). "Median Nuclei per Sample" refers to the median number of per-donor nuclei contributing to the pseudobulk expression for each snTIM. Additionally, we describe the regression F-statistic (including degrees of freedom) and the adjusted R^2.

Table S39 - Cell-type name translation between our study and Zeng-2024. "Zeng-2024" column refers to how the cell-type is referred to in the Zeng-2024 study. The PsychAD column refers to how the cell-type is referred to in this study.

Table S40 - Summary-level transcriptome-wide association study (S-snTWAS) and genome-wide association study (GWAS) correlation across traits and disorder categories.

Each row compares a different combination of traits ("Trait1" and "Trait2"). For each combination, we compute the S-snTWAS Pearson's correlation ("TWAS Correlation" and "TWAS Correlation lowCI" and "TWAS Correlation highCI" for the 95% confidence interval) as well as the genetic correlation computed by LD Score Regression ("GWAS Correlation" and "GWAS Correlation lowCI" and "GWAS Correlation highCI" for the 95% confidence interval; LD: linkage disequilibrium). We also annotate each row with the disorder categories for the indicated traits ("Trait1 Category" and "Trait2 Category") as well as their combination ("Category Combination").

Table S41 - Breakdown of each subcohort by ancestry. Each row describes the sample size ("N") of the specified ancestry ("Ancestry") in the specified subcohort ("Subcohort"). East Asian (**EAS**) and South Asian (**SAS**) are excluded due to low sample size and lack of consideration in analyses.

Table S42 - Chromosomal regions excluded due to high LD. Chromosomal regions (GRCh38) from which single nucleotide polymorphisms (**SNP**s) were excluded SNPs from for model training. Each row describes the combination of "Chromosome", "Start" and "Stop" of the excised window.

External Data (ref. 20)

External Data 1 - All summary-level transcriptome-wide association study (S-snTWAS) results. Each row refers to a different gene-cell-type-trait combination in S-snTWAS results. **"**Gene" refers to the Ensembl gene ID for the gene. "Gene name" refers to the Human Genome Organization (**HUGO**) Gene Nomenclature Committee (**HGNC**) gene symbol for the gene. "snTIM" column refers to the single-nucleus transcriptomic imputation model (**snTIM**) in question (Table S3). The "Trait" column refers to the genome-wide association study (**GWAS**) used for each TWAS (Table S12). The "z-score" and "p-value" column correspond to the calculated z-score and p-value from SPrediXcan, respectively. The "CV R2" and "CV p-value" columns refer to the TIM's cross-validation scores for each gene. "N(SNPs in model)" and "N(SNPs used)" refer to the number of model single nucleotide polymorphisms (**SNP**s), and the number of model SNPs available in the GWAS (after attempting to impute missing SNPs) respectively. "FDR" refers to the false discovery rate (**FDR**)-adjusted p-value for SPrediXcan (across all traits and cell-types). "Novel" refers to whether the gene is significant in Bulk S-TWAS or Multi-marker Analysis of GenoMic Annotation (**MAGMA**) ("No") or not ("Yes"). Finally, we also include the chromosome ("Chromosome") and GRCh38 gene start ("Start").

External Data 2 - All summary-level transcriptome-wide association study (S-snTWAS) pathway analysis results. "snTIM" column refers to the single-nucleus transcriptomic imputation model (**snTIM**) in question and the "Trait" column refers to the genome-wide association study (**GWAS**) used for each TWAS (Table S3; Table S12). The "Pathway name" and "Pathway ID" columns refer to the official name and ID for the pathway from the Gene Ontology (**GO**) consortium (https://geneontology.org/). "Chi-square", "df", and "p-value" correspond to the chi-square statistic, degrees of freedom, and p-value directly calculated by JEPEGMIX2-P, and "FDR" is the false discovery rate (**FDR**)-adjusted p-value across all snTIMs and traits.

External Data 3 - All individual-level transcriptome-wide association studies (I-snTWAS) results. Each row refers to a different gene-cell-type-trait combination in I-snTWAS results. **"**Gene" refers to the Ensembl gene ID for the gene. "Gene name" refers to the Human Genome Organization (**HUGO**) Gene Nomenclature Committee (**HGNC**) gene symbol for the gene. "snTIM" column refers to the single-nucleus transcriptomic imputation model (**snTIM**) in question (Table S3). The "Ancestry" column refers to the ancestry used for the analysis (either individuals of European, African or

Admixed American ancestry (**EUR**, **AFR**, or **AMR**))). The “Trait” column refers to the genome-wide association study (**GWAS**) used for each TWAS (Table S12). The “z-score”, “Effect size”, “SE” and “p-value” columns correspond to the calculated z-score, log odds ratio, standard error, and p-value from association analysis, respectively. The “CV R2” and “CV p-value” columns refer to the TIM’s cross-validation scores for each gene. “N(SNPs in model)” refers to the number of model single nucleotide polymorphisms (**SNP**s). “FDR” refers to the false discovery rate (**FDR**)-adjusted p-value from association analysis. Finally, we also include the chromosome ("Chromosome") and GRCh38 gene start (“Start”).

External Data 4 - All single-nucleus genetically regulated gene expression phenome-wide association study (snGReX-PheWAS) results. Each row represents a different ancestry-gene-cell-type trait combination from snGReX-PheWAS analysis. The “Ancestry” column refers to the ancestry used for the analysis (either individuals of European, African or Admixed American ancestry (**EUR**, **AFR**, or **AMR**)). “Gene name” refers to the Human Genome Organization (**HUGO**) Gene Nomenclature Committee (**HGNC**) gene symbol for the gene. "snTIM" column refers to the single-nucleus transcriptomic imputation model (**snTIM**) in question (Table S3). “Phecode number” and “Phecode name” describe the number and definition of the phecode from the PheWAS catalog (https://phewascatalog.org/) number for the phecode. “Category” describes the overall category for the phecode. Categories are mostly exacted from the PheWAS catalog, with some additional manual curation. The “Cases” and “Controls” column describes the number of cases and controls within the specified ancestry, respectively, for the relevant phecode. The “z-score”, “Effect size”, “SE” and “p-value” columns correspond to the calculated z-score, log odds ratio, standard error, and p-value from association analysis, respectively. “FDR” refers to the false discovery rate (**FDR**)-adjusted p-value from association analysis (across all genes, snTIMs, and phenotypes included in the table).

Supplementary References

124. Weissbrod, O. *et al.* Functionally informed fine-mapping and polygenic localization of complex trait heritability. *Nature Genetics* **52**, 1355–1363 (2020).

125. Crowell, H. L. *et al.* muscat detects subpopulation-specific state transitions from multi-sample multi-condition single-cell transcriptomics data. *Nat. Commun.* **11**, 6077 (2020).

126. Squair, J. W. *et al.* Confronting false discoveries in single-cell differential expression. *Nat. Commun.* **12**, 5692 (2021).

127. Zimmerman, K. D., Espeland, M. A. & Langefeld, C. D. A practical solution to pseudoreplication bias in single-cell studies. *Nat. Commun.* **12**, 738 (2021).

128. Murphy, A. E., Fancy, N. & Skene, N. Avoiding false discoveries in single-cell RNA-seq by revisiting the first Alzheimer’s disease dataset. *Elife* **12**, RP90214 (2023).

129. Nott, A. *et al.* Brain cell type-specific enhancer-promoter interactome maps and disease-risk association.

Science **366**, 1134–1139 (2019).

Supplementary Acknowledgements

PsychAD Consortium Authors
Aram Hong (1, 4, 6, 7); Athan Z. Li (10, 12); Biao Zeng (1, 4, 6, 7); Chenfeng He (9, 12); Chirag Gupta (9, 12); Christian Porras (1, 4, 6, 7); Clara Casey (1, 4, 6, 7); Colleen A. McClung (18); Collin Spencer (1, 4, 6, 7); Daifeng Wang (9, 10, 12); David A. Bennett (19); David Burstein (1, 2, 4, 6, 7, 8); Deepika Mathur (1, 4, 6, 7); Donghoon Lee (1, 4, 6, 7); Fotios Tsetsos (1, 2, 4, 6, 7); Gabriel E. Hoffman (1, 2, 4, 6, 7, 8); Genadi Ryan (13, 17); Georgios Voloudakis (1, 2, 3, 4, 6, 7, 8); Hui Yang (1, 4, 6, 7); Jaroslav Bendl (1, 4, 6, 7); Jerome J. Choi (11, 12); John F. Fullard (1, 4, 6, 7); Kalpana H. Arachchilage (9, 12); Karen Therrien (1, 4, 6, 7); Kiran Girdhar (1, 4, 6, 7); Lars J. Jensen (21); Lisa L. Barnes (19); Logan C. Dumitrescu (22, 23); Lyra Sheu (1, 4, 6, 7); Madeline R. Scott (18); Marcela Alvia (1, 4, 6, 7); Marios Anyfantakis (1, 4, 6, 7); Maxim Signaevsky (6, 7); Mikaela Koutrouli (1, 4, 6, 7, 21); Milos Pjanic (1, 4, 6, 7); Monika Ahirwar (13, 17); Nicolas Y. Masse (1, 4, 6, 7); Noah Cohen Kalafut (10, 12); Panos Roussos (1, 2, 4, 6, 7, 8); Pavan K. Auluck (20); Pavel Katsel (6); Pengfei Dong (1, 4, 6, 7); Pramod B. Chandrashekar (9, 12); Prashant N.M. (1, 4, 6, 7); Rachel Bercovitch (1, 4, 6, 7); Roman Kosoy (1, 4, 6, 7); Sanan Venkatesh (1, 2, 4, 6, 7); Saniya Khullar (9, 12); Sayali A. Alatkar (10, 12); Seon Kinrot (1, 4, 6, 7); Stathis Argyriou (1, 4, 6, 7); Stefano Marenco (20); Steven Finkbeiner (13, 14, 15, 16, 17); Steven P. Kleopoulos (1, 4, 6, 7); Tereza Clarence (1, 4, 6, 7); Timothy J. Hohman (22, 23); Ting Jin (9, 12); Vahram Haroutunian (5, 6, 7, 8); Vivek G. Ramaswamy (13, 17); Xiang Huang (12); Xinyi Wang (1, 4, 6, 7); Zhenyi Wu (1, 4, 6, 7); Zhiping Shao (1, 4, 6, 7)

PsychAD Consortium Affiliations
1: Center for Disease Neurogenomics, Icahn School of Medicine at Mount Sinai, New York, NY, USA
2: Center for Precision Medicine and Translational Therapeutics, James J. Peters VA Medical Center, Bronx, NY, USA
3: Department of Artificial Intelligence and Human Health, Icahn School of Medicine at Mount Sinai, New York, NY, USA
4: Department of Genetics and Genomic Sciences, Icahn School of Medicine at Mount Sinai, New York, NY, USA
5: Department of Neuroscience, Icahn School of Medicine at Mount Sinai, New York, NY, USA
6: Department of Psychiatry, Icahn School of Medicine at Mount Sinai, New York, NY, USA
7: Friedman Brain Institute, Icahn School of Medicine at Mount Sinai, New York, NY, USA
8: Mental Illness Research, Education and Clinical Center VISN2, James J. Peters VA Medical Center, Bronx, NY, USA
9: Department of Biostatistics and Medical Informatics, University of Wisconsin-Madison, Madison, WI, USA
10: Department of Computer Sciences, University of Wisconsin-Madison, Madison, WI, USA
11: Department of Population Health Sciences, University of Wisconsin-Madison, Madison, WI, USA
12: Waisman Center, University of Wisconsin-Madison, Madison, WI, USA
13: Center for Systems and Therapeutics, Gladstone Institutes, San Francisco, CA, USA
14: Department of Neurology, University of California San Francisco, San Francisco, CA, USA

15: Department of Physiology, University of California San Francisco, San Francisco, CA, USA
16: Neuroscience and Biomedical Sciences Graduate Programs, University of California San Francisco, San Francisco, CA, USA
17: Taube/Koret Center for Neurodegenerative Disease Research, Gladstone Institutes, San Francisco, CA, USA
18: Department of Psychiatry, University of Pittsburgh School of Medicine, Pittsburgh, PA, USA
19: Rush Alzheimer's Disease Center and Department of Neurological Sciences, Rush University Medical Center, Chicago, IL, USA
20: Human Brain Collection Core, National Institute of Mental Health-Intramural Research Program, Bethesda, MD, USA
21: Novo Nordisk Foundation Center for Protein Research, Faculty of Health and Medical Sciences, University of Copenhagen, Copenhagen, Denmark
22: Vanderbilt Genetics Institute, Vanderbilt University Medical Center, Nashville, TN, USA
23: Vanderbilt Memory & Alzheimer's Center, Vanderbilt University Medical Center, Nashville, TN, USA
