# Supplementary material for: Single-nucleus transcriptome-wide association study of human brain disorders
Source: Nature. 2026 Sep 23;657(8133):1016–26. doi: 10.1038/s41586-026-10836-6 (PMC13600940; doi:10.1038/s41586-026-10836-6)
Supplement: Supplementary file 2 [file 41586_2026_10836_MOESM2_ESM.pdf]

nature portfolio

Corresponding author(s): Georgios Voloudakis; Panos Roussos

Last updated by author(s): Mar 10, 2026

Reporting Summary

Nature Portfolio wishes to improve the reproducibility of the work that we publish. This form provides structure for consistency and transparency in reporting. For further information on Nature Portfolio policies, see our Editorial Policies and the Editorial Policy Checklist.

Statistics

For all statistical analyses, confirm that the following items are present in the figure legend, table legend, main text, or Methods section.

n/a	Confirmed	
☐	☒	The exact sample size (*n*) for each experimental group/condition, given as a discrete number and unit of measurement
☐	☒	A statement on whether measurements were taken from distinct samples or whether the same sample was measured repeatedly
☐	☒	The statistical test(s) used AND whether they are one- or two-sided<br>*Only common tests should be described solely by name; describe more complex techniques in the Methods section.*
☐	☒	A description of all covariates tested
☐	☒	A description of any assumptions or corrections, such as tests of normality and adjustment for multiple comparisons
☐	☒	A full description of the statistical parameters including central tendency (e.g. means) or other basic estimates (e.g. regression coefficient) AND variation (e.g. standard deviation) or associated estimates of uncertainty (e.g. confidence intervals)
☐	☒	For null hypothesis testing, the test statistic (e.g. *F*, *t*, *r*) with confidence intervals, effect sizes, degrees of freedom and *P* value noted<br>*Give P values as exact values whenever suitable.*
☐	☒	For Bayesian analysis, information on the choice of priors and Markov chain Monte Carlo settings
☐	☒	For hierarchical and complex designs, identification of the appropriate level for tests and full reporting of outcomes
☐	☒	Estimates of effect sizes (e.g. Cohen's *d*, Pearson's *r*), indicating how they were calculated

Our web collection on statistics for biologists contains articles on many of the points above.

Software and code

Policy information about availability of computer code

Data collection	No software was used for data collection.
Data analysis	Python code used v3.8.2 for python3 code, and v2.7.16 for python2 code. R code used v4.2.0 for the majority of analyses, with v3.5.3, v4.3.0, and v4.5.0 used for select analyses (select prediXcan scripts were run in R v3.5.3, and analyses using PsychAD counts per million were performed in R v4.3.0). The following versions of software were used in R v3.5.3: AnnotationDbi v1.44.0, assertthat v0.2.1, Biobase v2.42.0, BiocGenerics v0.28.0, biomaRt v2.38.0, bit v1.1-14, bit64 v0.9-7, bitops v1.0-6, blob v1.2.1, cachem v1.0.6, codetools v0.2-16, colorspace v1.4-1, compiler v3.5.3, crayon v1.5.1, data.table v1.14.2, DBI v1.0.0, dplyr v1.0.7, edgeR v3.24.3, ellipsis v0.3.2, fansi v1.0.3, fastmap v1.1.0, foreach v1.4.4, generics v0.1.2, ggplot2 v3.3.3, ggrepel v0.8.1, glmnet v2.0-18, glue v1.6.2, grid v3.5.3, gridExtra v2.3, gtable v0.3.0, hms v0.4.2, httr v1.4.3, IRanges v2.16.0, iterators v1.0.10, lattice v0.20-38, lifecycle v1.0.1, limma v3.38.3, locfit v1.5-9.1, magrittr v2.0.3, Matrix v1.6-5, memoise v2.0.1, munsell v0.5.0, pbmcapply v1.4.1, peer v1.0, pillar v1.7.0, pkgconfig v2.0.3, plyr v1.8.4, preprocessCore v1.44.0, prettyunits v1.0.2, progress v1.2.2, purrr v0.3.4, R6 v2.5.1, Rcpp v1.0.8.3, RCurl v1.95-4.12, readr v1.3.1, reshape2 v1.4.3, rlang v0.4.10, RSQLite v2.1.1, S4Vectors v0.20.1, scales v1.0.0, stats4 v3.5.3, stringi v1.4.3, stringr v1.4.0, tibble v3.0.3, tidyselect v1.1.0, tools v3.5.3, utf8 v1.2.2, vctrs v0.3.8, viridis v0.5.1, viridisLite v0.3.0, withr v2.5.0, XML v3.98-1.19. The following versions of software were used in R v4.5.0: abind v1.4-8, Biobase v2.68.0, BiocGenerics v0.54.0, compiler v4.5.0, crayon v1.5.3, DelayedArray v0.34.1, generics v0.1.4, GenomeInfoDb v1.44.3, GenomeInfoDbData v1.2.14, GenomicRanges v1.60.0, grid v4.5.0, httr v1.4.7, IRanges v2.42.0, jsonlite v2.0.0, lattice v0.22-7, Matrix v1.7-4, MatrixGenerics v1.20.0, matrixStats v1.5.0, R6 v2.6.1, S4Arrays v1.8.1, S4Vectors v0.46.0, SingleCellExperiment v1.30.1, SparseArray v1.8.1, SummarizedExperiment v1.38.1, tools v4.5.0, UCSC.utils v1.4.0, XVector v0.48.0. The followings version of software was used in R v4.3.0: limma v3.5.4. The following versions of software were used in R v4.2.0: abind v1.4-8, ashr v2.2-63, assertthat v0.2.1, BiocGenerics v0.44.0, bit v4.5.0, bit64 v4.5.2, bitops v1.0-8, blob v1.2.4, cachem v1.1.0, calibrate v1.7.7, cli v3.6.5, colorspace v2.1-1, compiler v4.2.0, corrplot v0.94, cowplot v1.1.3, crayon v1.5.3, data.table v1.16.0, data.table v1.18.0, DBI v1.2.3, dplyr v1.1.4, egg v0.4.5, EnvStats v3.0.0, fansi v1.0.6, fastmap v1.2.0, generics v0.1.3, GenomeInfoDb v1.34.9, GenomeInfoDbData v1.2.9, GenomicRanges v1.50.2, ggdendro v0.2.0,

ggnewscale v0.5.0, ggpattern v1.1.1, ggplot2 v3.5.1, ggplot2 v4.0.1, ggrepel v0.9.6, ggwordcloud v0.5.0, glue v1.8.0, gridExtra v2.3, gtable v0.3.5, hms v1.1.3, imputez v1.1.1, invgamma v1.1, IRanges v2.32.0, irlba v2.3.5.1, lattice v0.20-45, lifecycle v1.0.4, magrittr v2.0.3, mashr v0.2.79, MASS v7.3-56, mathjaxr v1.6-0, Matrix v1.6-5, mclust v6.1.1, memoise v2.0.1, metadat v1.2-0, metafor v4.6-0, metaforest v0.1.4, mixsqp v0.3-54, MultiWAS v0.1.0, munsell v0.5.1, mvtnorm v1.3-1, nlme v3.1-157, numDeriv v2016.8-1.1, pbmcapply v1.5.1, pillar v1.9.0, pkgconfig v2.0.3, plyr v1.8.7, plyr v1.8.9, png v0.1-8, prettyunits v1.2.0, progress v1.2.3, R6 v2.5.1, ranger v0.16.0, rbibutils v2.2.16, Rcpp v1.0.14, RCurl v1.98-1.16, Rdpack v2.6.1, remaCor v0.0.18, reshape v0.8.9, reshape2 v1.4.4, rlang v1.1.6, rmeta v3.0, RSQLite v2.3.7, S4Vectors v0.36.2, scales v1.3.0, scales v1.4.0, SQUAREM v2021.1, stats4 v4.2.0, stringi v1.8.4, stringr v1.5.1, tibble v3.2.1, tidyselect v1.2.1, tools v4.2.0, truncnorm v1.0-9, utf8 v1.2.4, variancePartition v1.28.9, vctrs v0.6.5, viridis v0.6.5, viridisLite v0.4.2, withr v3.0.1, XVector v0.38.0, zlibbioc v1.44.0. We also used JEPEGMIX2-P v1.0.1, FOCUS v0.803, LDSC v1.0.1 and MEGENA v1.3.6. The MVP data core used APT version 2.11.3, Affy2vcf, PLINK1.9, SHAPEIT4 v4.1.3, Minimac4, and EIGENSOFT v.6.

For manuscripts utilizing custom algorithms or software that are central to the research but not yet described in published literature, software must be made available to editors and reviewers. We strongly encourage code deposition in a community repository (e.g. GitHub). See the Nature Portfolio guidelines for submitting code & software for further information.

Data

Policy information about availability of data

All manuscripts must include a data availability statement. This statement should provide the following information, where applicable:

- Accession codes, unique identifiers, or web links for publicly available datasets
- A description of any restrictions on data availability
- For clinical datasets or third party data, please ensure that the statement adheres to our policy

All results are included either in the main text or provided in Supplementary Tables or Data. We are also making snTIMs and accompanying SNP covariance matrices publicly available on Zenodo (doi: 10.5281/zenodo.17435917) and Synapse (https://doi.org/10.7303/syn63181047).
PsychAD single nucleus transcriptomics data is available via the AD Knowledge Portal (https://adknowledgeportal.org) and Synapse (https://doi.org/10.7303/syn60084804).

Research involving human participants, their data, or biological material

Policy information about studies with human participants or human data. See also policy information about sex, gender (identity/presentation), and sexual orientation and race, ethnicity and racism.

Reporting on sex and gender	Sex was used as a covariate for I-snTWAS and PheWAS analyses and sex effect was evaluated where applicable. We confirmed the match between one's self-reported and genetically inferred sex in all applicable cohorts (MVP, ROSMAP, and FACS-MG).
Reporting on race, ethnicity, or other socially relevant groupings	Genetic ancestry was inferred for all applicable cohorts (MVP, ROSMAP, and FACS-MG). Due to low sample size in other ancestries, only results for individuals of European ancestry, African ancestry, and Admixed American ancestry were reported. Except where indicated, analyses were performed separately on each of the indicated ancestries.
Population characteristics	Sex, age, and top ten genetic principal components were used for I-snTWAS and PheWAS regression analyses.
Recruitment	All brain specimens and genotypes were obtained through informed consent via brain donation programs at the respective organizations.
Ethics oversight	All procedures and research protocols were approved by the respective ethical committees of our collaborator's institutions. Ethics committee/IRB of Mount Sinai gave ethical approval for this work. Ethics committee/IRB of James J. Peters Department of Veterans Affairs Medical Center gave ethical approval for this work. Ethics committee/IRB of Rush Alzheimer's Disease Center gave ethical approval for this work. Ethics committee/IRB of National Institute of Mental Health Human Brain Collection Core gave ethical approval for this work.

Note that full information on the approval of the study protocol must also be provided in the manuscript.

Field-specific reporting

Please select the one below that is the best fit for your research. If you are not sure, read the appropriate sections before making your selection.

☒ Life sciences ☐ Behavioural & social sciences ☐ Ecological, evolutionary & environmental sciences

For a reference copy of the document with all sections, see nature.com/documents/nr-reporting-summary-flat.pdf

Life sciences study design

All studies must disclose on these points even when the disclosure is negative.

Sample size	From PsychAD, no sample-size calculation was performed. Our goal was to assemble the most extensive datasets possible on human PFC. Therefore, we included as many brain specimens in our study as our budget would permit.<br><br>For S-snTWAS analysis, we selected publicly available brain disorder GWAS with sufficient power(> 1 FDR-significant gene-trait association in S-snTWAS analysis and five genome-wide significant loci in GWAS analysis). For the I-snTWAS analysis only traits with at least 2% case

	prevalence in EUR, AFR and AMR were utilized for power considerations. For the snGReX-PheWAS analysis, traits with at least 500 cases and 500 controls were considered.
Data exclusions	No data were excluded from the analysis except for sample size considerations as stated above.
Replication	Positive replication of PsychAD snTIMs and downstream S-snTWAS was determined via out-of-sample validation in ROSMAP and FACS-MG cohorts. We also replicated cross-disorder associations using MVP associations, and S-snTWAS with I-snTWAS.
Randomization	Covariates were accounted for in regression analyses and no randomization was required to split samples into groups. Randomization was used to determine training and testing sets for transcriptomic imputation models, and for a supplementary analysis showing that model performance was consistent across ancestries even when accounting for linkage disequilibrium.
Blinding	No blinding of data was necessary due to previous investigators having organized the PsychAD cohort.

Reporting for specific materials, systems and methods

We require information from authors about some types of materials, experimental systems and methods used in many studies. Here, indicate whether each material, system or method listed is relevant to your study. If you are not sure if a list item applies to your research, read the appropriate section before selecting a response.

Materials & experimental systems

n/a	Involved in the study
☒	☐ Antibodies
☒	☐ Eukaryotic cell lines
☒	☐ Palaeontology and archaeology
☒	☐ Animals and other organisms
☒	☐ Clinical data
☒	☐ Dual use research of concern
☒	☐ Plants

Methods

n/a	Involved in the study
☒	☐ ChIP-seq
☒	☐ Flow cytometry
☒	☐ MRI-based neuroimaging

Plants

Seed stocks	n/a
Novel plant genotypes	n/a
Authentication	n/a
