# Supplementary material for: Single-nucleus transcriptome-wide association study of human brain disorders
Source: Nature. 2026 Sep 23;657(8133):1016–26. doi: 10.1038/s41586-026-10836-6 (PMC13600940; doi:10.1038/s41586-026-10836-6)
Supplement: Supplementary file 3 [file 41586_2026_10836_MOESM5_ESM.pdf]

nature portfolio

Peer Review File

Single-nucleus transcriptome-wide association study of human brain disorders

Corresponding Author: Professor Panos Roussos

Version 0:

Reviewer comments:

Referee #1

(Remarks to the Author)
In this article, Ventakesh et al. describe an interesting approach to performing single-nucleus transcriptomic wide association studies and apply this concept to 12 human brain disorders. I do not have all the statistical skills to fully evaluate this complex article. However, several points can be made:

Is the combination of R2cv≥0.1 and pcv≤0.05 stringent enough? In other words, to what extent does the subsequent analysis rely on the low quality of the transcriptomic expression imputation? Although the authors describe several approaches to control for this, the question can of course be raised with an additional 11,266 genes that were not initially imputed in bulk. It will be interesting to see how these numbers change with different selection thresholds. It is important to understand the sensitivity of the approach to such selection criteria.

The evaluation of brain snTIMs should also be made more precise by excluding lncRNAs and focusing on coding genes. At the biological level, this is currently the most informative information, as the relevance of non-coding transcripts is not yet well understood. This will also significantly improve the evaluation and comparison of the expression quality of transcriptomic imputations by targeting obvious biological targets.

In general, this article presents too much data and it is very difficult to understand the main message that the authors want to convey, in particular how these results can be translated to the biological level. Indeed, although the approach is very interesting, its application to 12 different brain disorders remains very superficial and ultimately does not allow us to assess the strength of the approach compared to more traditional approaches. It would have been useful to present an in-depth comparison for a specific pathology in order to test the interest of this work.

Due to its complexity, this article is primarily aimed at specialists who will be able to juggle multi-layered analyses. In addition, the authors should make an effort to make their data more accessible, as, for example, there are no titles and explanations associated with the various supplementary tables.

Referee #3

(Remarks to the Author)
In this study, Sanan Venkatesh et al. have made significant progress in addressing the current limitations of TWAS pipelines. They have worked extensively to improve both the resolution of results and the transferability of findings across different ancestries, which is a major challenge in the field. Their research includes important validations at various levels of resolution, tested in both dependent and independent datasets. They have also employed new methods to explore the pleiotropy of cell type-specific gene expression associations with traits, which has valuable implications for neuropsychiatric and neurodegenerative diseases. This approach is applicable to other diseases as single-cell data becomes more widely available.
While the study is strong, some revisions could further enhance the manuscript:

1. Fig 1, Panel C. The QQ plot in Fig 1, Panel C shows early deviation of R2cv values from expected values, with a small range on the x-axis. It would be helpful if the authors could clarify whether this is due to bias or the expected distribution is not a good match for R2cv values.
2. Fig 1, Panel D. Could the authors confirm whether Spearman correlation was performed only on significant R2cv values? If not, clarification on how non-significant values were handled (e.g., NaN or 0) would be useful, as imputing with zeros can inflate correlation. A sparse matrix correlation might also be worth considering.

3. The text states “More genes were identified in Subclass despite the greater number of unique imputable genes for Class (Fig. 1A), suggesting greater importance of finer resolution versus the number of imputable genes”, but Fig 1A shows the opposite. Although the conclusion is valid.
4. The statement “Furthermore, for commonly identified associations, we observed a 24% increase in power9 utilizing PsychAD snTIMs.” lacks data support. Providing a reference to relevant tables or figures is helpful.
5. MAGMA Analysis has not been introduced before “We classified significant genes as “novel” if the GTA was not identified by Bulk S-TWAS (Table S20) or MAGMA gene-based analysis (Table S21)”, it will be helpful to introduce and describe it earlier in the manuscript for better context.
6. Figure 2C Labelling. Figure 2C is described as a comparison between snTIMs and snBulk, but it actually shows Class vs Subclass.
7. “Approximation of S-TWAS GTA effect sizes with existing methods37,45 hinders our ability to assess the cell-type heterogeneity across GTAs independent of power considerations. “ This sentence needs more clarification. Could the authors elaborate on this point?
8. Resolution Choices in MASH Analysis: More explanation on why class-level resolution was chosen over subclass in MASH analysis would be valuable for understanding the decision-making process for each part of the paper.
9. The statement “largely driven by cell-type specific single-nucleus expression quantitative trait loci (sn-eQTL; Extended Data Fig. 5B)” would benefit from more quantitative backing. Additionally, more clarification is required on whether these are cell-type-specific eQTLs and whether SNPs are non-overlapping.
10. The legend panels for Extended Data Fig 3 are not aligned with the figure. A is B and vice versa.
11. Figure 3C - Readability:
Increasing the size of symbols in Figure 3C will improve readability, making it easier to differentiate significant and non-significant dots.

Referee #4

(Remarks to the Author)
This is a very well written piece, reporting on results from a timely endeavor for the field of psychiatric genetics. I’m not aware of similar studies in neuropsychiatry of this scale and scope. The methods are sound and the authors guard against overinterpreting findings by conducting a range of validation/sensitivity analyses for most findings. The findings are strengthened not only by the large number of nuclei and human donors but also the validation in an external dataset. I have mostly minor comments/questions/suggestions.

In the abstract it’s unclear whether the donors are healthy individuals. It’s also unclear what the ‘associations’ are, with what?

Have the authors examined to what degree their findings are specific to neuropsychiatric disorders and how they are different between the broad category of neurological and the broad categories of substance use disorders and psychiatric disorders as cell type enrichment studies indicate there may be category-level differences in involvement of brain cells? Similarly, where are the definitions of NPDs and NDDs given? I would recommend adding a short section with a specific header about the phenotypes to the methods, including a rationale for the use of the phenotypes selected by the authors. From the reference list 79-90 I see the phenotypes. OCD seems missing. Although important updates of BIP, OCD and MDD GWAS are to be published in the coming months I see how the authors may not have excess to these phenotypes. I also see how often updated GWASs in psychiatry are published and it’s impossible to have used the latest upon publication of new paper like the current. However, as these three GWASs show significantly increased SNPbased h2 relative to previous GWASs one may consider trying to get access to one or more of them (all PGC), particularly as the current paper will be published later. Alternatively, the authors may simply add OCD to their analyses as well as psychiatric cross-disorder sum stats to their analyses, as the latter captures the 8 main psychiatric disorders and has good statistical power.

Have the authors considered comparing these DLPFC findings w/ previous findings from other brain regions, eg either in an analysis when such data are available or in the discussion by comparisons with findings from the literature? How are the findings to be interpreted in light of single cell findings of donors with psychiatric disorders? Similarly, how about findings in mouse models? Have the authors considered providing a short, (supplemental) discussion about alignment with findings from mouse donors?

This taps into general comment about the balancing between results and discussion. The results section is lengthy, detailed and largely technical. A discussion section with more room for the implications of the findings (research + clinical) and the interpretation in light of the current literature may help balance the piece. Generally, the discussion falls short on research and clinical implications of the findings and continues along the same, technical lines.

Some of the abbreviations are not explained upon their first occurrence, eg ‘R2
94 CV ≥ 0.01, pCV ≤ 0.05’, which sometimes makes reading difficult. Here, not only may these abbreviations be best explained in the results, but also their meaning (‘Imputable genes are considered passing cross-validation R2 (R2 CV) ≥ 0.01, pCV ≤ 0.05 and
SNPs in model > 0. R2 CV and pCV values are prediction performance R2 and prediction performance value from the “PredictDB” software75.’).

Here and there the authors report in their methods the statistical test they apply but not how to interpret the results. Are they significant from a certain p-value threshold (corrected for multiple testing when applicable) or do they caution against using a

p-value to infer significance and prefer to give an ES? Eg 'We performed linear regression to assess the extent to which major snTIM metadata predictors (sample size and median number of nuclei contributing to the pseudobulk expression from every individual) influence snTIM performance (proxied by number of confidently imputed genes). We report the adjusted R2 as a measure of the variation explained in snTIM performance for each predictor;
adjusted R2 was estimated by the stats package76,77.' And 'We compared whether genes were more strongly imputed in psychAD snTIMs or Bulk using a sign test. To more
accurately calculate the sign test p-value, we utilized Ramanujan's factorial approximation.' Generally, I would be expecting the authors could give confidence intervals and SE more often, e.g. when they report an OR. These may be better estimates of precision than p-values for some inferential stats.

The first two, lengthy sections of the results give me the impression this is a methodological paper. If this is the authors' intention and the authors and editors deem these sections ('Brain snTIMs capture brain GReX with cell-type specificity across ancestries' and 'Brain snTWAS increases power for both known and novel gene-trait 144 association discovery.') indispensable for the general understanding of the piece, I would keep. Alternatively, one may consider shortening these sections a bit, moving them to the supplements or merging them, using one, more general header that may also be more understandable for non-experts. My personal impression is that the exciting part of the paper lies more in the results discussed in the abstract that could me emphasized more, e.g. by moving them up in the results section. However, if the authors deem these methodological parts of their work just as valuable they may consider outlining somewhere in the intro that there were two general goals to this work and clearly structuring the piece accordingly.

I suggest the first paragraph of the discussion discusses not only the main methods (as in its last sentence) but then summarizes the results in 1-2 additional sentences.

It's helpful to read about the performance in comparison to bulk for each phenotype under investigation. Again, many details are given. Can the authors compute a pooled estimate for the enrichment OR across diseases to obtain a summary estimate for this goal of comparing the yield of their method to bulk and other methods? It would be good to see precision estimates here for all phenotypes separately and then jointly. Generally, some of the results sections may benefit from a one or two-sentence summary in the end to highlight the most relevant of findings and bridge the gap to the next section.

Figures: great graphics.
1: see comment above about the first two headers. I wonder whether this figure is needed and would prefer the methodological figure as figure 1 (now ED F1), also considering not every reader of Nature may be familiar w/ all methods.
2: can the authors compute a pooled estimate for the enrichment OR across diseases or wouldn't this be methodologically sound (see above)?
3: if B are stacked bar charts why is the ordering from bottom to up discrepant across stacks? The figure is hard to grasp currently. Why not order from bottom to up equally for all phenotypes, so blue would always be up? As suggested above, I would be interested in seeing neurological, SUD and psychiatric phenotypes as three separate categories.
6: 'alcoholism' is a bit of an archaic term that is more adequately written in the legend with "" than in B. is it defined somewhere? In C it has an asterisk that I was unable to locate in the caption. I generally recommend using up-to-date idiom like alcohol use disorder whena applicable or 'alcohol-related disorders' or something of the like and explaining in methods their meanings.

Referee #5

(Remarks to the Author)
In "Single-nucleus transcriptome-wide association study of human brain disorders," Venkatesh et al. present results of a cell type specific transcriptome wide association study performed in single-nucleus sequencing data derived from dorsolateral prefrontal cortex samples collected from ~1500 donors. They used the estimated snp effects to derive expression prediction models and then performed analyses of genetically predicted cell type specific gene expression for a range of psychiatric and neurodegeration traits to discover novel gene-trait associations. MVP was used to validate findings. This is an interesting paper and the suggestion that gene trait associations estimated from genetic regulation of expression in bulk measures from tissues comprising heterogeneous tissue types (effectively "masking" cell type specific effects in bulk analyses) is a sensible premise warranting exactly this kind of comparative analysis. However, I have some statistical/bioinformatic concerns that may bias some of the main observations in the manuscript, and it will be important to address these (or at least clarify why they are not relevant in the main text for the reader) the ensure the findings are robust.

A major comment I have is that in our hands, we have found that ten fold cross validation R2 estimates can be inflated in certain circumstances. I'm concerned that the dependence of the process of distinguishing the cellular populations prior to the cell type specific model building (which uses the global expression patterns) may exacerbate this problem. Similarly, use of corrections that utilize the full data (e.g. PEER). This kind of feature engineering can be problematic and cause data leakage in k-fold performance estimation. Given that the sample size is fairly robust here, it would be reassuring if an experiment was performed in a hold out set that is uncontaminated (separate QC, separate normalization, etc) to assess performance of the snTIMs and show that the cv R2 estimates are largely similar to performance in independent data. This could be done as a sensitivity analysis so as not to interfere with the main results of the paper. The reason this matters so much is because of the comparisons to the ten fold cross validation performance of the bulk models- if the authors' models are differently biased/inflated by the shared additional data processing of the snRNAseq data (aka data leakage), these results could be invalid.

If this experiment were performed, it would also be helpful to see how the models trained in each ancestry-specific data set

perform on the cell type specific measured expression of that ancestry's hold out set compared to the other ancestries cell type specific measured expression, rather than comparing the R2cv.

Could the performance estimates also be inflated by the approach to selecting the best gene models in the snRNAseq? Many more models are constructed here, potentially aggregating the type 1 error. If the authors have mitigated this risk, eg with more stringency on test corrections, it would be helpful to the reader to note these methodological considerations in the sections where the results are presented.

As nearly all of the trait GWASs considered analyzed UK Biobank data, there is likely substantial overlap in cases and controls in the effect estimates across studies, and this could be a major source of confounding in the cross-disorder investigations. This would bias the genetic correlation analyses. If adjustments were made to account for sample overlap between these studies, it should be noted in the results.

Can the authors clarify that all of the 12 EUR GWAS used in the S-snTWAS analyses did not include MVP?

"Unsurprisingly, across NPD and NDDs, we found a similar number of associated genes in snBulk (2,472) and Bulk (2,287)." With how many genes overlapping?

Minor, but please note explicitly what is correlated when reporting results, e.g. it would be helpful if line 164 notes that z scores were compared.

The FDR correction of the S-snTWAS was calculated across all gene-cell-type combinations for each trait. Some of the excess of findings in these analyses may be explained by inadequate control of type 1 error given the multiple testing; how many results survive an experiment wide FDR correction across all traits? Relatedly, in the section on AD (line 222-237) please clarify what is meant by FDR = 3.76 × 10-62, are these q values? These are not comparatively adjusted, as the authors note that multiple test correction with FDR (≤ 0.05) was performed within each snTIM rather than across relevant snTIMs in these analyses. Having lower power isn't a good justification for less stringent test corrections, and the lack of consistency is potentially misleading to the reader.

95% confidence intervals (CIs) should be included to express the uncertainty associated with I2 estimates.

Very minor- when multiple significance matrices are shown in a single figure, please label them in the figure as well as in the legend (eg extended data 5)

Version 1:

Reviewer comments:

Referee #1

(Remarks to the Author)
The authors have done a very good job of taking my comments into account and have made their article more relevant and accessible.

Referee #3

(Remarks to the Author)
The authors have adequately addressed my concerns and questions in the revised manuscript

Referee #4

(Remarks to the Author)
The authors have very adequately processed my suggestions and I commend them on a great manuscript. I have no further concerns or questions.

Referee #5

(Remarks to the Author)
Single-nucleus transcriptome-wide association study of human brain disorders

Venkatesh et al. present a single-nucleus TWAS, using ancestry-specific models they developed from 27 dorsolateral prefrontal cortex cell populations from ~1,500 donors, comprising three ancestry groupings (European, African, and Admixed American). As proof of principle, and to demonstrate advantages of single-nucleus models, they applied these models for GReX association analyses of 12 neuropsychiatric and neurodegenerative traits and identified more than 4,000 gene-trait associations that were not identified in their pseudo-bulk-tissue models. This paper is presenting a tremendous amount of

work, which will be of high interest to many readers working in complex trait genetics. My main critique is that the paper is so dense, with references to extended/supplementary material, that it disrupts the flow and makes it somewhat difficult to read. I also have some minor concerns about some of the interpretations made.

Major comments:
1. To improve the readability of the Main section, could the authors move some of the references to extended material into the Methods sections, or write some out in the text? An example of the latter suggestion, for: "Using genotype and snRNA-seq data from the PsychAD Consortium, we trained 94 snTIMs across three hierarchical levels of cell-type resolution, encompassing 32 cellular populations (27 non-overlapping across 4 classes and 23 subclasses) in the DLPFC (Extended Data Fig. 2; Table S1)."

Possible edit: "Using genotype and snRNA-seq data from the PsychAD Consortium, we trained 94 snTIMs across three hierarchical levels of cell-type resolution, encompassing 32 cellular populations (27 non-overlapping across 4 classes and 23 subclasses) in the DLPFC. Cellular populations and class groupings are shown in Extended Data Fig. 2, and Table S1 gives the abbreviations for each cellular population used throughout."

Additionally, there are many acronyms and abbreviations throughout the paper, and it would be helpful to have a central reference that readers can refer to.
2. Please comment on variables relating to the sampling that could impact variation in transcript measures, such as time from death to transcript preservation, and how these variables could be affecting the model, and justify this choice in the text.
3. Was there any adjustment for PEER factors in the model construction process?
4. Some interpretations seem either a bit overstated or to not fully capture the nuance of the data, e.g.:
a. In "Brain snTIMs capture brain GReX with cell-type-specificity across ancestries", the statement: "Despite differences in training sample size, shared gene-cell-type models showed strong correlations in R2CV…suggesting that genetic regulation of expression is broadly consistent across ancestries."
I have two concerns, the first being that genes are not independent measures, so unless the data was filtered to remove genes with correlated expression first, the correlation values will be inflated. The second is that the stats presented are showing that the model quality is similar across ancestries, but not that the models themselves are similar.
I have similar concerns about the statement: "However, here we demonstrate that ancestry-matched experimental designs are very effective for cross-ancestry NPD/NDD studies even with limited sample sizes, paralleling prior diverse ancestry blood monocyte TWASs."
The "ancestry-matched experimental designs are very effective for cross-ancestry NPD/NDD studies" part is a bit vague, it would be helpful to clarify some and this might address my concerns. Also, though, given that what was presented doesn't clarify the similarity of the models themselves, just their performance, it's not clear to me that the data that's presented effectively supports this statement.
b. In "Brain snTWAS increases power for both known and novel gene-trait
association discovery", the statement: "Higher cell-type resolution increased the gene-to-locus ratio, indicating that distinct cellular populations may contribute uniquely within shared loci and warrant further functional study."
This is one possible explanation, but there are other possible explanations, especially that differences in power or reduced control for multiple testing, given the increase in number of test sin the cell-type-specific models, could explain this. It would be helpful to acknowledge this possibility here, and throughout the paper.
c. Similarly, in the Discussion, the statement: "Compared with bulk approaches, there seems to be no downside at the gene level in transitioning to snRNA-seq beyond cost and instances where capturing extranuclear RNA might prove desirable."
I have a few concerns, including the same concern as above, that the difference in multiple testing control given the increased number of tests is not acknowledged here. Also, the study does not make the comparison that would be most helpful in clarifying this point, which would be to perform actual bulk RNA-seq, rather than the pseudo-bulk (which is a helpful comparison point as well, but not quite the same), in the same study participants and to compare not only the number of genes and genes per locus identified, but also the actual genes themselves, and their abundance and predictive models. If the authors could show that the snRNA-seq approach identifies all of the genes identified by the bulk approach, this would better support the point they're making here. This concern could also be addressed just by changing the language a bit, perhaps to "our study did not find downsides at the gene level…".
5. It is surprising to me that several of the original 16 traits were considered underpowered for S-snTWAS (binge-eating disorder, post-traumatic stress disorder, anxiety, and obsessive-compulsive disorder). The sample sizes are similar to the GWAS that were included. Do the authors have a proposed explanation for why they were insufficiently powered?
6. In the PheWAS, the definition of controls as "individuals with fewer than 2 phecodes" is unusual. Typically people with a single phecode would be excluded from the analysis, or possibly considered cases. This is not a major problem, because it would mostly impact power, but it is probably worth giving a justification.
7. In Figure 4A, is it possible that the definitions of the lower-left triangle and upper-right triangle are reversed? It doesn't make sense to me that the number of shared genes would be higher when requiring the cell type to be shared between the traits as well.
8. Please make a figure similar to Supplementary Figure 3 that compares the R2CV to the R2 of the models applied to the independent datasets. (Apologies if I missed this.)
Minor comments:
1. In "Brain snTIMs capture brain GReX with cell-type-specificity across ancestries", it would be helpful to note the ancestry of participants in the ROSMAP data.
2. Clarity could be improved in several places, including:
a. The way that genes that were implicated as potentially causal by fine-mapping are referenced is not very clear, in several places. E.g., "Notably, most of these key genes were also fine-mapped in the relevant cell-types (Table S16), supporting their causal involvement." I think the authors are referring to genes that had PIP > 0.95 in fine-mapping, but that's not clear

from the statement.
b. In “Brain snTWAS increases power for both known and novel gene-trait association discovery”, the statement “Genes were classified as “novel” if the GTA was not identified by Bulk S-TWAS (Table S12; region-matched analysis of the same GWAS using homogenate models) or by MAGMA…” I’m not sure what is meant by a “region-matched analysis”
c. In “Ancestry-specific snTWAS uncovers shared biology and aids in fine-mapping of risk genes”, the statement “RHOBTB2’s plausibility is reinforced by functional work showing that BTB-domain variants increase excitability in human iPSC-derived neurons and genetically interact with voltage-gated sodium channel pathways in Drosophila”. I’m not sure what “genetically interact” means here, can you be more specific?
d. In the Discussion, “Compared to the only published snTWAS in MDD, we noted 6,095/31 more imputable/significant gene-cell-type combinations.” The way the numbers are presented here is not very clear. This could probably be improve by listing them as “6,095 more imputable and 31 more significant gene-cell-type combinations”.
e. There are still a few instances of acronyms being referenced prior to, or without, definitions (e.g., MSSM, HBCC, RADC, ADSP).
f. There are also some analyses or details in the Methods that are referenced before they are defined. It would be helpful to move the definition of the MHC region up to where it is first referenced, and the description of the PCTA methods up to the first time it is used.
g. I believe the references to removal of the MAPT locus due to potential for bias are specific to Alzheimer’s disease. It would be helpful to clarify this for the broader readership.
h. In “Million Veteran Program Genotype Quality Control”, “high disequilibrium regions” needs to be defined.
i. In “GReX-PheWAS association analysis”, “top ten ancestral principal components”, it would be more accurate to call them “genetic principal components”, or even just “principal components”.
j. In “S-snTWAS validation”, “Genotypes were TOPMed-imputed and only common SNPs were utilized for model building.” This needs either references to further details or the details given in text, including the MAF threshold used to define common variants.
k. The figure legends and figures themselves contain many acronyms/abbreviations that are not defined in the figure legends. Definitions should be added to the legends as much as possible, or refer to a table elsewhere if needed.
3. In the statement “Collectively, these results indicate that trans-ancestry snTWAS fine-mapping improves both cell-type-specific gene discovery and causal-gene prioritization across ancestries.” Change “trans-ancestry” to “multi-ancestry” to reflect currently acceptable terminology.
4. In “Variant-level filtering in the training cohort (PsychAD)”, “missing information for SNP predictors in existing TIMs were replaced with double the in-sample MAF (representing the in-sample average genotype), rounded to the nearest integer.” It would be helpful to give the proportion of variants where this was needed. If the proportion is high it would make more sense to perform imputation, if low, this is probably fine.
5. In “Training of cell-type-specific PrediXcan models”, to help readers evaluate how the definition of imputable genes in this work compares to commonly used publicly available models, such as the GTEx v8 models released through PredictDB.
6. For Figure 5E, while I appreciate that the figure is trying to present a very complex, it is hard to interpret. Please ensure that this data is presented in Table format as well, for additional evidence of these findings. For Figure 5C, the scale of the plot is confusing. Why are the I-snTWAS significant “No” sections so small?

Version 2:

Reviewer comments:

Referee #5

(Remarks to the Author)
The authors have done a really nice job responding thoroughly to our prior review. I have only a few very minor remaining comments:

REF5-A-4a "Despite differences in training sample size, shared gene-cell-type models showed strong correlations in R2CV ..., suggesting that genetic regulation of expression is broadly consistent across ancestries, even when the contributing single nucleotide polymorphisms (SNPs) are different (Supplementary Notes 3 and 4)."

I think this is still a little misleading. I think it's fine to say the predictive performance of the models is similar, or that the variance of gene expression levels explained by genetic variants is similar across ancestries. Or maybe you could simply add the word "extent" : "suggesting that the extent of genetic regulation of expression is broadly consistent across ancestries..." As is, it still seems that you are saying that the genetic regulation itself is similar across ancestries, which your analyses seem to show is actually quite dissimilar.

This is also still a little confusing to me, “While most prior work has emphasized the poor portability of EUR-trained models to other ancestries17,18, our results show that ancestry-matched designs can be highly informative for cross-ancestry NPD and NDD studies even with modest sample sizes, paralleling prior diverse ancestry blood monocyte TWASs19–21.” I think the use of matched-ancestry and cross-ancestry referring to analyses of the same datasets is getting me tangled up. I'm not sure how this reflects your finding that mismatches in ancestry when applying snTIMs to GWAS may lead to misidentified biological signals.

PIP of 0.5 is pretty low (I feel like it is more common to see 0.8 or even higher?). Might be worth mentioning as a limitation.

No need to change, but just a comment for the future- King does not optimize for sample retention when removing individuals to minimize relatedness. See for example, PMID: 22996348.

Open Access This Peer Review File is licensed under a Creative Commons Attribution 4.0 International License, which permits use, sharing, adaptation, distribution and reproduction in any medium or format, as long as you give appropriate credit to the original author(s) and the source, provide a link to the Creative Commons license, and indicate if changes were made.
In cases where reviewers are anonymous, credit should be given to 'Anonymous Referee' and the source.
The images or other third party material in this Peer Review File are included in the article's Creative Commons license, unless indicated otherwise in a credit line to the material. If material is not included in the article's Creative Commons license and your intended use is not permitted by statutory regulation or exceeds the permitted use, you will need to obtain permission directly from the copyright holder.
To view a copy of this license, visit https://creativecommons.org/licenses/by/4.0/

Response to Reviewers' comments

Referee #1 (Remarks to the Author): 3
Summary 3
Comments 3
REF1-1. SnTIM filtering 3
REF1-2. Focus on coding genes 3
REF1-3. Pathology-focused case study 4
REF1-4. Improve clarity and accessibility 6
Referee #3 (Remarks to the Author): 7
Summary 7
Comments 7
REF3-1. QQ plot R2CV clarification 7
REF3-2. Spearman correlation inclusion 8
REF3-3. Text vs Fig. 1A consistency 9
REF3-4. Power analysis correction 9
REF3-5. Introduce MAGMA earlier 10
REF3-6. Fig. 2C labeling 11
REF3-7. TWAS effect size clarity 11
REF3-8. mash resolution choice 14
REF3-9. Cell-type sn-eQTL quantification 15
REF3-10. Extended Data Fig. 3 legend 17
REF3-11. Fig. 3C readability 17
Referee #4 (Remarks to the Author): 18
Summary 18
Comments 18
REF4-1. Abstract clarity: donors, associations 18
REF4-2. Disorder category definitions 19
REF4-3. Cross-region and mouse alignment 23
REF4-4. Research and clinical implications 24
REF4-5. Define abbreviations 26
REF4-6. Thresholds and precision reporting 26
REF4-7. Streamline early Results 27
REF4-8. Discussion results summary 27
REF4-9. Pooled enrichment OR 28
REF4-10. Fig. 1 organization 28
REF4-11. Pooled OR in Fig. 2 28
REF4-12. Fig. 3B: Stacked bar ordering 29
REF4-13. Update AUD terminology 29
Referee #5 (Remarks to the Author): 30

Summary.. 30
Comments... 30
REF5-1. External snTIM validation... 30
REF5-2. Out-of-sample validation of non-EUR snTIMs.. 33
REF5-3. Model selection stringency.. 33
REF5-4. GWAS sample overlap.. 34
REF5-5. Addressing whether GWASs used contained MVP participants.......................... 35
REF5-6. Bulk vs snBulk overlap.. 36
REF5-7. Specify correlated metrics.. 36
REF5-8. Experiment-wide FDR control.. 36
REF5-9. I2 confidence intervals... 37
REF5-10. Figure significance labels... 37
References.. 37

Referee #1 (Remarks to the Author):

Summary

In this article, Ventakesh et al. describe an interesting approach to performing single-nucleus transcriptomic wide association studies and apply this concept to 12 human brain disorders. I do not have all the statistical skills to fully evaluate this complex article. However, several points can be made:

Comments

**REF1-1. SnTIM filtering**	
Is the combination of R2cv≥0.1 and pcv≤0.05 stringent enough? In other words, to what extent does the subsequent analysis rely on the low quality of the transcriptomic expression imputation? Although the authors describe several approaches to control for this, the question can of course be raised with an additional 11,266 genes that were not initially imputed in bulk. It will be interesting to see how these numbers change with different selection thresholds. It is important to understand the sensitivity of the approach to such selection criteria.	
**Response**	We thank the reviewer for this helpful suggestion to improve the accuracy of our reported gene imputation models. In TWAS, the most important criterion for retaining a gene model is whether the predicted expression shows a statistically significant association with the observed expression in cross-validation tests[1]. This is addressed by filtering the model's p-values ($FDR_{CV} \leq 0.05$)[2]. Many groups, including ours, also apply a small performance threshold ($R^2_{CV} \geq 0.01$)[3,1], which corresponds to a predicted-observed correlation of roughly 0.1 or higher, to exclude very weak models.
**Action**	In response to the reviewer's comment, we increased the stringency of our filters from $p_{CV} \leq 0.05$ to $FDR_{CV} \leq 0.05$, aligning with stricter TWAS practices; we retained the $R^2_{CV} \geq 0.01$ which is what most groups apply when needed. This change had only a minor impact on the number of imputable genes (in EUR, from 20,189 unique genes across 128,198 gene-cell-type combinations to 20,181 unique genes across 128,061 combinations).

**REF1-2. Focus on coding genes**	
The evaluation of brain snTIMs should also be made more precise by excluding lncRNAs and focusing on coding genes. At the biological level, this is currently the most informative information, as the relevance of non-coding transcripts is not yet well understood. This will also significantly improve the evaluation and comparison of the expression quality of transcriptomic imputations by targeting obvious biological targets.	
**Response**	We thank the reviewer for this suggestion to improve the interpretability of our findings. Although our snTIMs include noncoding genes (for those interested in exploring them), we

	agree that focusing on coding genes in this manuscript is most appropriate for relating our findings to disease status.
**Action**	To enhance the mechanistic relevance of our results, we now restrict the main figures and discussion to protein-coding genes, moving analyses of all transcripts to the Supplement. We still include all transcript models when performing multiple-testing correction as outlined in REF1-1 to identify imputable transcripts; however, only protein-coding genes are carried forward into downstream analyses and visualizations. In EUR snTIMs, 12,289 of 20,181 unique imputable genes (60.9%) are coding. While this filter reduces the number of imputable genes that are considered, we observed improved performance in several analyses, including increased enrichment for novel genes.

**REF1-3. Pathology-focused case study**	
In general, this article presents too much data and it is very difficult to understand the main message that the authors want to convey, in particular how these results can be translated to the biological level. Indeed, although the approach is very interesting, its application to 12 different brain disorders remains very superficial and ultimately does not allow us to assess the strength of the approach compared to more traditional approaches. It would have been useful to present an in-depth comparison for a specific pathology in order to test the interest of this work.	
**Response**	We agree that the original submission emphasized the magnitude of the advancement for transitioning from homogenate to cell-resolved GReX when studying brain disease at the expense of demonstrating its translational potential in specific examples.
**Action**	We added a focused Alzheimer's disease (AD) case study that links cell-type-resolved genetic signals to pathology-anchored microglial programs (Extended Data Fig. 4A-D). Specifically, we tested enrichment of AD S-snTWAS genes in a published microglial co-expression network through competitive enrichment. Concordance was specific to Class-Immune and tracked DEG signatures associated with Clinical Dementia Rating (CDR) scores and Braak AD pathology stages. Three immune modules (M824; parent-child M406→M947) showed negative enrichment and association with β-amyloid plaque density, highlighting myeloid programs and *TREM1* dysregulation. This analysis prioritized the *EPHA1* locus: *ZYX* is predicted to be downregulated in AD and fine-maps with near-complete support, despite not reaching significance in DEG analyses. Together, this targeted pathology comparison demonstrates the translational utility of our approach and provides an in-depth evaluation for a case study.
**Excerpt**	"While pathway-level analyses revealed broad cell-type-specific perturbations, we next examined whether S-snTWAS recapitulate and extend known associations with brain pathology, using AD as a case study. To assess microglial contributions, we leveraged a hierarchical co-expression network analysis generated in freshly isolated human microglia spanning healthy and neurodegenerative aging[26]. We first tested microglial co-expression network modules for competitive enrichment of AD S-snTWAS signals (see Methods).

	Class-Immune AD associations showed the strongest correspondence with AD pathology-linked differential expressed gene (**DEG**) signatures[26] (Extended Data Fig. 4A) that was not present in other cell-types. Three modules (FDR-adjusted p-value ≤ 0.05) were significantly enriched for both AD-related differential expression and β-amyloid plaque density, and all exhibited negative enrichment (reduced predicted or observed expression in AD; Extended Data Fig. 4B; Table S21). Functional annotation of these modules highlighted immune and myeloid activation programs, including module M824, which mapped to myeloid gene expression, and a parent-child pair (M406→M947) pointing to *TREM1* dysregulation (Extended Data Fig. 4C; Table S22). Both M406 and M947 contained *ZYX* (Zyxin), which our S-snTWAS predicts to be downregulated in AD (Class-Immune: FDR-adjusted p-value = 9.18×10^{-14}, z-score = −8.29; Subclass-MG: FDR-adjusted p-value = 1.61×10^{-13}, z-score = −8.21) and fine-mapped with essentially complete posterior support (FOCUS PIP = 1.0 and 1.0; Table S16). Notably, *ZYX* did not emerge in differential-expression analyses (Extended Data Fig. 4D). The same modules also included *EPHA1-AS1*, recently linked to AD[26], which is located at the same GWAS locus as *ZYX*, underscoring the genetic and functional complexity of this region. Together, these results prioritize *ZYX* as a putative coding effector at this locus and link its downregulation to impaired microglial stress responses to β-amyloid[41]."<br><br>"To validate and interpret these pathway-level signals, we performed a microglia-focused, module-level analysis in AD using a published MEGENA[57] network from freshly isolated human microglia[26]. S-snTWAS module enrichments closely tracked DEG signatures associated with AD pathology, predominantly in Class-Immune and for CDR and Braak, identifying three negatively enriched immune modules also associated with amyloid plaque burden. These modules highlighted altered myeloid programs and *TREM1* dysregulation and prioritized the *ZYX*/*EPHA1-AS1* locus, implicating *ZYX* as a putative coding effector whose predicted downregulation may predispose cells to impaired stress responses to β-amyloid exposure[41]. Together, this AD case study shows how cell-type-resolved TWAS bridges genetic signals to microglial co-expression modules anchored to AD pathology."<br><br>**Extended Data Fig. 4**

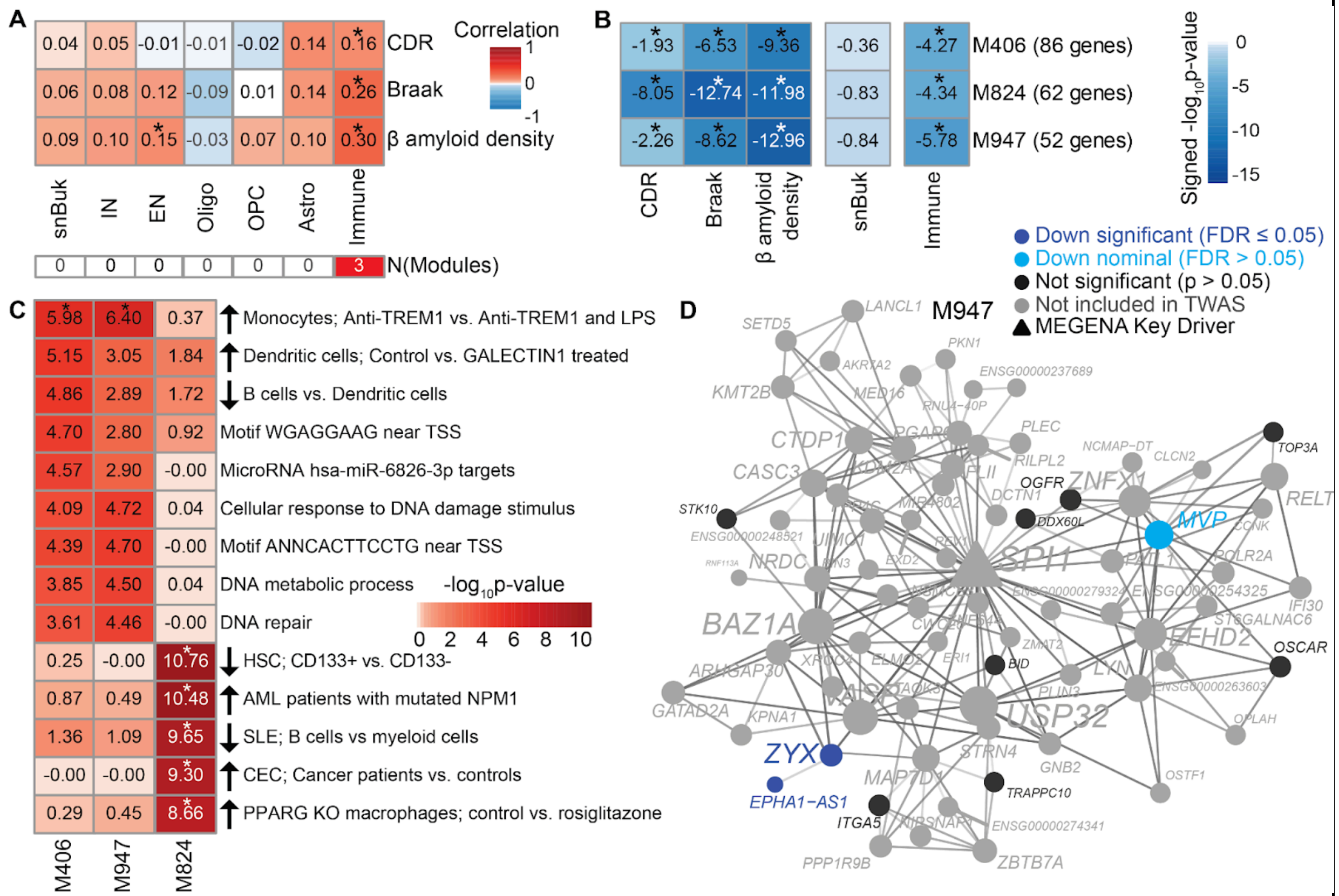

Extended Data Fig. 4 | Relation of Alzheimer's Disease (AD) summary-level single-nucleus transcriptome-wide association study (S-snTWAS) genes to AD associated changes in Microglia. A, Relationship of AD S-snTWAS z-scores with MEGENA co-expression modules identified in freshly isolated primary human microglia. Top heatmap indicates Pearson's correlation of module enrichment scores for S-snTWAS z-scores (X-axis) and module enrichment scores for AD associated neuropathology measures (Y-axis) across all 306 modules containing at least 50 genes. Asterisks indicate FDR-adjusted p-value ≤ 0.05. The bottom heatmap indicated the number of individual MEGENA modules significantly (FDR-adjusted p-value ≤ 0.05) enriched for S-snTWAS signatures. **B,** Heatmap indicating the enrichment of neuropathology and S-snTWAS signatures in the 3 FDR significant modules identified in Extended Data Fig. 4A, bottom plot. Asterisks indicate FDR-adjusted p-value ≤ 0.05. In selected cells the text was colored white to enhance visibility. **C,** Heatmap of top pathways enriched in the 3 FDR significant MEGENA modules. Asterisks indicate FDR-adjusted p-value ≤ 0.05. Pathways were renamed for greater clarity of the underlying biology. Upward facing arrow indicates upregulation and downward facing arrow indicates downregulation in the differential expressed gene (**DEG**) generated signatures from MSigDB C4 Immune dataset. Aliases for each pathway are detailed in Table S22. **D,** Gene-gene diagram showing TWAS signature annotation for module 947.

REF1-4. Improve clarity and accessibility

Due to its complexity, this article is primarily aimed at specialists who will be able to juggle multi-layered analyses. In addition, the authors should make an effort to make their data more accessible, as, for example, there are no titles and explanations associated with the various supplementary tables.		
**Response**	We appreciate this feedback and have revised the manuscript to enhance readability and accessibility.	
**Action**	We relocated detailed methodological sections to the Supplementary Results, allowing specialists to explore them in depth without interrupting the main narrative. We also added descriptive titles and expanded explanatory captions to all Supplementary Tables.	

Referee #3 (Remarks to the Author):

Summary

In this study, Sanan Venkatesh et al. have made significant progress in addressing the current limitations of TWAS pipelines. They have worked extensively to improve both the resolution of results and the transferability of findings across different ancestries, which is a major challenge in the field. Their research includes important validations at various levels of resolution, tested in both dependent and independent datasets. They have also employed new methods to explore the pleiotropy of cell type-specific gene expression associations with traits, which has valuable implications for neuropsychiatric and neurodegenerative diseases. This approach is applicable to other diseases as single-cell data becomes more widely available.
While the study is strong, some revisions could further enhance the manuscript:

Comments

**REF3-1. QQ plot R^2_{CV} clarification**	
Fig 1, Panel C. The QQ plot in Fig 1, Panel C shows early deviation of R2cv values from expected values, with a small range on the x-axis. It would be helpful if the authors could clarify whether this is due to bias or the expected distribution is not a good match for R2cv values.	
**Response**	We thank the reviewer for noting the missing clarification on how to interpret this plot.<br><br>Briefly, the QQ plot compares each ancestry's observed cross-validated R^2_{CV} to a theoretical null in which no correlation exists between genetically predicted and observed expression ($\rho=0$; gray line). Under this null, and given our out-of-fold effective sample sizes (n), most expected R^2 values lie near zero explaining the narrow x-axis. Intuitively, with larger n the probability of observing a large R^2 by chance diminishes; the upper tail of the null R^2 distribution shrinks roughly in proportion to $1/(n-3)$. The early upward deviations reflect genuine predictive signals (widespread genetic regulation of expression), not bias.

	In more detail, to construct the null under ρ=0 with out-of-fold effective sample size n, we use the standard approach Fisher approach[7]:<br>1. Work with the expected order statistics of the Fisher-transformed correlation, which under ρ=0 follows a normal distribution with mean 0 and variance 1/(n-3).<br>2. Convert these values back to correlation coefficients using the inverse of Fisher z-transform.<br>3. Because R^2 depends only on correlation magnitude, retain the positive half of this symmetric distribution and square the correlations to obtain expected R^2 values.<br>4. Sort these expected R^2 values to align with the ranks of the observed points, yielding the gray reference line.<br>Following reviewer feedback about the manuscript's technical density, this panel has been moved to Supplementary Fig. 2B.
**Action**	We have clarified in the caption of Supplementary Fig. 2B that the null distribution assumes that gene expression is not genetically regulated.
**Excerpt**	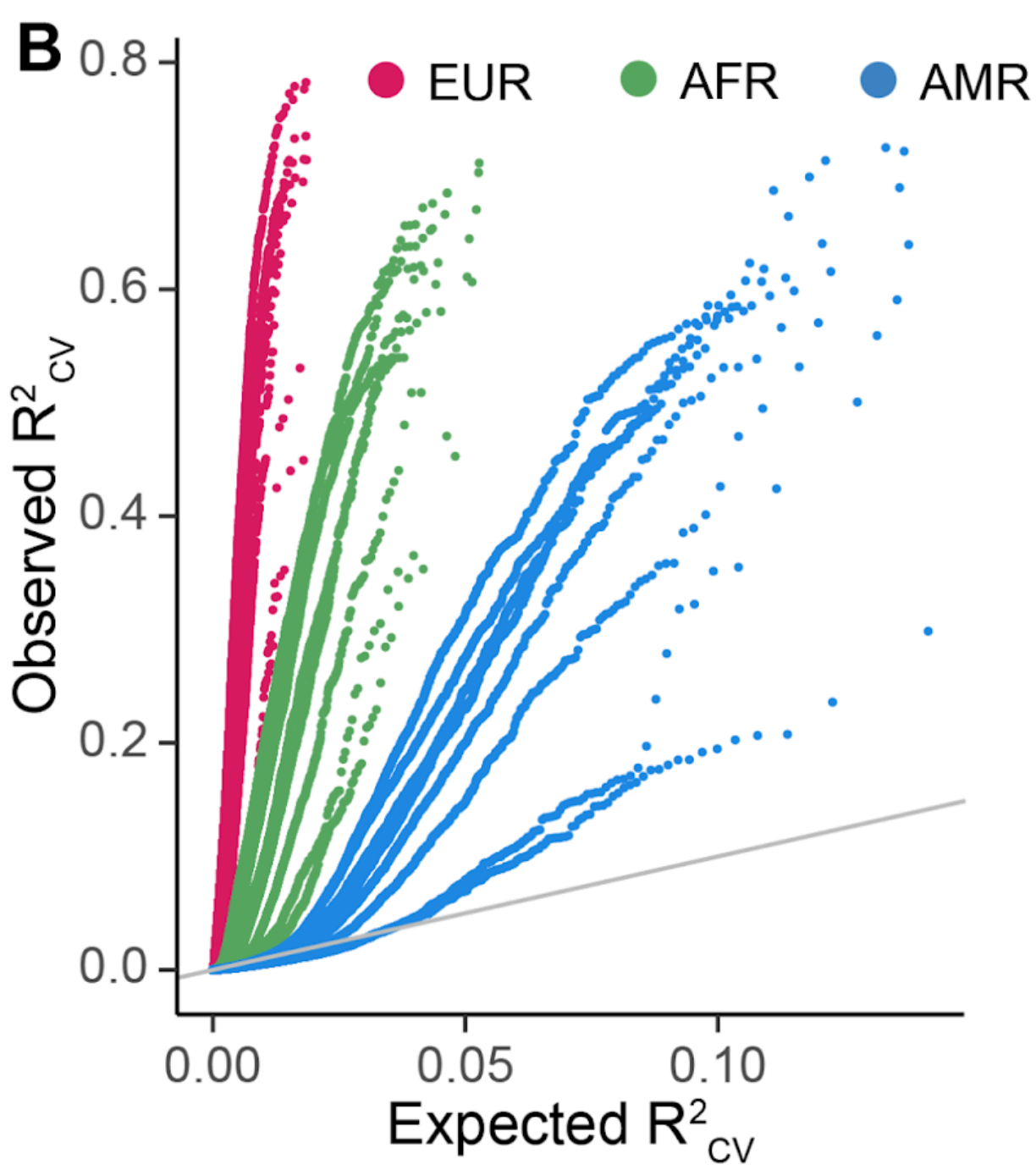 **Supplementary Fig. 2 | Cross Validation R^2 across cell-types and ancestries. B,** Power comparison of snTIMs across ancestries. Quantile-quantile plot of the observed against expected performance (R^2_{CV}) for every class-level model across 3 genetic ancestries. EUR: European; AFR: African; AMR: Admixed-American. Null distribution (gray line) represents the hypothesis that gene expression is not genetically regulated.

**REF3-2. Spearman correlation inclusion**

Fig 1, Panel D. Could the authors confirm whether Spearman correlation was performed only on significant R2cv values? If not, clarification on how non-significant values were handled (e.g., NaN or 0) would be useful, as imputing with zeros can inflate correlation. A sparse matrix correlation might also be worth considering.	
**Response**	We confirm that Spearman correlation was computed only for genes significantly imputed in both ancestries (R^2_{CV} ≥ 0.01 and FDR_{CV} ≤ 0.05; now Fig. 1C). Non-significant genes were excluded rather than set to 0 or NaN. Correlations were calculated on pairwise complete cases of shared genes across ancestries. Because the resulting vectors are dense, sparse-matrix computation was unnecessary.
**Action**	We have added a clarification to the caption of Fig. 1C.
**Excerpt**	"For each cell-type, Spearman's correlation is assessed only amongst genes confidently imputed in both the ancestry's snTIMs."

**REF3-3. Text vs Fig. 1A consistency**	
The text states "More genes were identified in Subclass despite the greater number of unique imputable genes for Class (Fig. 1A), suggesting greater importance of finer resolution versus the number of imputable genes", but Fig 1A shows the opposite. Although the conclusion is valid.	
**Response**	We thank the reviewer for pointing out the lack of clarity in this sentence, and have revised it to specify which metric is being referenced to identify genes.
**Action**	We have replaced this sentence in the main text. In the original sentence, the first "genes" referred to significant gene-trait associations in Fig. 2A, whereas the second referred to the number of imputable genes shown in Fig. 1B (previously Fig. 1A). The new sentence avoids this confusing language.
**Excerpt**	"Across traits, Bulk and snBulk yielded comparable numbers of associated genes (1,494 vs. 1,254). Among genes imputable by both models, 882 were significant in snBulk and 963 in Bulk, with 529 overlapping (Jaccard = 0.40). Extending this analysis across levels of resolution, subclass-level models yielded the greatest number of unique significant associations (3,003 genes across all traits), followed by Class (2,470) and snBulk (1,254), indicating that increased cell-type resolution enhances gene-trait discovery (Fig. 2A; Supplementary Fig. 5)."

**REF3-4. Power analysis correction**
The statement "Furthermore, for commonly identified associations, we observed a 24% increase in power9 utilizing PsychAD snTIMs." lacks data support. Providing a reference to relevant tables or figures is helpful.

**Response**	We appreciate the request for substantiation. Our original "24% increase in power" was derived from a descriptive χ^2-based comparison we have used previously[8]: we aligned the intersection of gene-trait-cell-type combinations, retained pairs with χ^2 (= z^2) ≥ 1 in both PsychAD and Zeng-2024, computed χ^2 per pair and method, and interpreted the percent increase in mean χ^2 for PsychAD relative to Zeng-2024 as an approximate percent gain in effective sample size (power). Because this comparison is descriptive: *(i)* it can be disproportionately influenced by a few very strong GTAs, and *(ii)* it does not provide formal inference or account for dependence, multiple testing, or winner's-curse; thus, we have removed the "24%" claim in the revision. We recognize that there is no universally accepted metric for comparing the power of TWAS analyses and have therefore omitted this metric in the resubmission.
**Action**	Instead of the χ^2-based comparison, we now present side-by-side counts of significant and imputable genes for PsychAD and Zeng-2024, which provides a more transparent and standardized comparison.
**Excerpt**	First, in the ROSMAP cohort, we applied the PsychAD cellular taxonomy to cluster and pseudobulk the snRNA-seq data. We then imputed cell-class-specific gene expression using ROSMAP genotypes and the PsychAD snTIMs. Comparing imputed GReX with observed gene expression in ROSMAP yielded R^2_{out} estimates that closely matched our R^2_{CV} predictions (Spearman's ρ = 0.63; N = 70,156; p-value = $3.91 \times 10^{-7,844}$; Supplementary Fig. 27A), demonstrating external validity and showing no evidence for feature-engineering-induced leakage which can inflate R^2_{CV} estimates. Next, we performed MDD S-snTWAS using our PsychAD snTIMs and compared these results to a recently published independent ROSMAP-based S-snTWAS (**Zeng-2024**)[21]. After matching cell classes across cohorts (Table S37), we correlated the S-snTWAS z-scores based on the same MDD GWAS summary statistics[129]. Despite differences in cohorts (PsychAD vs. ROSMAP), cell clustering/taxonomy, TIM methods (PrediXcan vs. FUSION[130]), and sample sizes (920 vs. 424), we observed strong concordance (Pearson's $r_{(13,760)}$ = 0.78, p-value = $1.34 \times 10^{-2,772}$) between z-score sets (Supplementary Fig. 27B). By simple counts, we observed 6,095 more imputable gene-cell-type combinations (PsychAD: 26,920; Zeng-2024: 20,825) and 31 more significant gene-cell-type associations (PsychAD: 335; Zeng-2024: 304) under the same FDR-adjusted p-value threshold, consistent with broader coverage and a higher discovery yield.

**REF3-5. Introduce MAGMA earlier**	
MAGMA Analysis has not been introduced before "We classified significant genes as "novel" if the GTA was not identified by Bulk S-TWAS (Table S21) or MAGMA gene-based analysis (Table S22)", it will be helpful to introduce and describe it earlier in the manuscript for better context.	
**Response**	We appreciate this comment, especially regarding accessibility for readers less familiar with gene-based association methods.

**Action**	We added a short introduction to MAGMA in the Introduction and expanded its description in the Results section where MAGMA-based comparisons are first used.
**Excerpt**	"While gene-based tests, such as MAGMA, aggregate GWAS summary statistics for common variants, transcriptome-wide association studies (**TWAS**) integrate these data with predictive models of genetically regulated gene expression (**GReX**) to identify genes whose expression changes may drive disease susceptibility[10–12]."<br><br>"... by MAGMA gene-based analysis (Table S13)[28,29], which detects genes through common variant aggregation of GWAS summary statistics alone. "

**REF3-6. Fig. 2C labeling**	
Figure 2C Labelling. Figure 2C is described as a comparison between snTIMs and snBulk, but it actually shows Class vs Subclass.	
**Response**	Thank you for catching this labeling error. We have corrected it to ensure consistency between the figure and its description.
**Action**	Fig. 2C's caption and legend now correctly indicate that dark blue represents "Class vs snBulk" and light blue represents "Subclass vs snBulk."

**REF3-7. TWAS effect size clarity**	
"Approximation of S-TWAS GTA effect sizes with existing methods37,45 hinders our ability to assess the cell-type heterogeneity across GTAs independent of power considerations. " This sentence needs more clarification. Could the authors elaborate on this point?	
**Response**	We thank the reviewer for prompting clarification. By "independent of power considerations" we mean that assessing cell-type heterogeneity across gene-trait associations (GTAs) relies on effect size magnitudes rather than on test statistics. Even when z-scores are well calibrated and power is high, summary TWAS effect sizes can be systematically mis-scaled, which biases cross-cell-type comparisons (e.g., I^2) regardless of sample size. While no single publication explicitly concludes that summary-TWAS effect-size estimates are mis-calibrated, this inference follows directly from the mathematical derivations in MetaXcan/S-PrediXcan and TWAS/FUSION which depend on reference-panel variances. The original papers frame the z-score as the primary outcome.<br><br>Additional sources of inaccuracy in summary TWAS effect size estimation (beyond those affecting z-scores) are illustrated below for the S-PrediXcan method which was used in our study:

	1. <u>Reference panel scaling of predicted expression variance (direct magnitude bias).</u> The effect size of the predicted gene expression is yielded by the following approximation: $\hat{\gamma}_g \approx \frac{Z_g}{\sqrt{N\,\sigma^2_{g,\mathrm{ref}}}}$, with $\sigma^2_{g,\mathrm{ref}} = \mathbf{w}^\top \boldsymbol{\Sigma}^{(\mathrm{ref})}\mathbf{w}$. Where:<br>- $\hat{\gamma}_g$: The estimated gene-level effect size (what we are trying to calculate).<br>- Z_g: The gene-level association z-score calculated from SNP-level GWAS summary statistics and TIM prediction weights.<br>- N: The GWAS sample size used in the SNP-level association analysis.<br>- $\sigma^2_{g,\mathrm{ref}}$: The variance of the predicted expression of gene computed from the reference panel (e.g., the training genotype-gene expression panel).<br>- $\mathbf{w}$ are the prediction weights and $\boldsymbol{\Sigma}^{(\mathrm{ref})}$ is the LD from a reference panel (training cohort), not the GWAS cohort.<br>Any mismatch between $\boldsymbol{\Sigma}^{(\mathrm{ref})}$ and the study LD rescales $\hat{\gamma}_g$. This scale drift produces uncalibrated effect size estimates.<br>2. <u>No intrinsic propagation of prediction-model uncertainty (attenuation).</u> S-PrediXcan treats the prediction weights $\mathbf{w}$ as fixed plug-ins, even though they are estimated from a training panel. Let $\widehat{\mathbf{w}} = \mathbf{w}^* + \boldsymbol{\eta}$, where $\boldsymbol{\eta}$ is the estimation error. The imputed expression is $\widehat{T}_g = \mathbf{G}\widehat{\mathbf{w}} = T^*_g + U$ with extra noise $U = \mathbf{G}\boldsymbol{\eta}$, where $\mathbf{G}$ is the study genotype matrix. Regressing the trait on $\widehat{T}_g$ without modeling U produces classical measurement-error attenuation of the estimated effect size $\hat{\gamma}_g$ toward zero. This occurs because the variance term in $\sigma^2_{g,\mathrm{ref}} = \mathbf{w}^\top \boldsymbol{\Sigma}^{(\mathrm{ref})}\mathbf{w}$ does not include the additional variance from U. By contrast, the z-score remains well calibrated because any multiplicative scaling of the weight vector $\widehat{\mathbf{w}}$ affects both the numerator and denominator, so the magnitude stays the same: $Z_g = \frac{\widehat{\mathbf{w}}^\top \mathbf{z}}{\sqrt{\widehat{\mathbf{w}}^\top \boldsymbol{\Sigma}^{(\mathrm{ref})}\,\widehat{\mathbf{w}}}}$.<br>3. <u>SNP coverage and harmonization gaps (implementation bias).</u> S-PrediXcan uses overlapping SNPs; non-overlapping or misaligned variants alter $\mathbf{w}^\top\mathbf{z}$ (which affects both z-score and effect size estimates) and $\sigma^2_{g,\mathrm{ref}}$ (which impacts only effect size estimates). The latter further perturbs the effect size scale relative to an individual-level analysis with fully imputed $\widehat{T}_g$. In our manuscript, we mitigated this by performing ancestry-specific imputation of missing SNPs in the GWAS summary statistics to maximize overlap with TIM SNPs.<br>Note: In our tests below we found direct magnitude bias to be stronger than attenuation bias.<br><br><u>Empirical evidence from MVP (EUR) supporting the above points.</u><br>Across nine NPD/NDD traits (AUD, Anxiety, BD, AD, Insomnia, MDD, Migraine, PTSD, SCZ) in MVP, we compared summary-level S-TWAS with I-TWAS. Starting with the same individuals, genetic data and case/control status information we performed: (*i*) S-TWAS: GWAS first, then S-PrediXcan and *(ii)* I-TWAS: GReX imputation first, then diagnosis status ~

	GReX association analysis. For each regression we used the same individuals/outcome/covariates for the association analysis. We then observed the following:<br>- Z-scores: Pearson's $r_{(668860)}$ = 0.99 (95% CI: 0.99047 - 0.99054)<br>- Effect sizes: Pearson's $r_{(668860)}$ = 0.67475 (95% CI: 0.67368 - 0.67581)<br>- Mean % change (I-TWAS vs. S-TWAS): z-scores 1.90% (95% CI: 0.63% - 3.16%) vs. effect sizes 22.20% (95% CI: 8.40% - 35.99%).<br>These results demonstrate that while z-scores replicate almost perfectly, effect sizes are substantially distorted. Therefore, summary-level effect sizes should not be used to quantify cell-type heterogeneity across GTAs. Instead, we impute GReX at the individual level and estimate effects with I-TWAS. Consistent with this, we do not rely on S-TWAS effect sizes for any heterogeneity analyses; all effect-size-based results use I-TWAS.
**Action**	The corresponding section in the main manuscript has been revised to explicitly reference the calibration issues, and we have added a new Supplementary Fig. 8 illustrating the empirical impact of these biases which we believe will be helpful to the broader readership. We do not reproduce the derivations in the main text, as our conclusions follow directly from the equations presented in the original S-PrediXcan publication (part of the MetaXcan framework)[2].
**Excerpt**	"Existing S-TWAS methods[33,34] produce well-calibrated association z-scores that closely match results from individual-level analyses (Table S19; Supplementary Fig. 9A). In contrast, their effect size estimates are less dependable because they rely on reference panel quantities rather than the GWAS cohort, specifically the single nucleotide polymorphism (**SNP**) linkage disequilibrium used to scale the variance of predicted expression and allele frequencies/SNP coverage used for harmonization, and they do not propagate uncertainty in the prediction weights[33] (Table S19; Supplementary Fig. 9B-C). As a result, summary-based effect sizes should not be used to quantify cell-type heterogeneity, and aggregation approaches that rely on z-scores or p-values can blur true effect magnitude with sampling precision and power[35,36]."<br><br>**Supplementary Fig. 9**<br>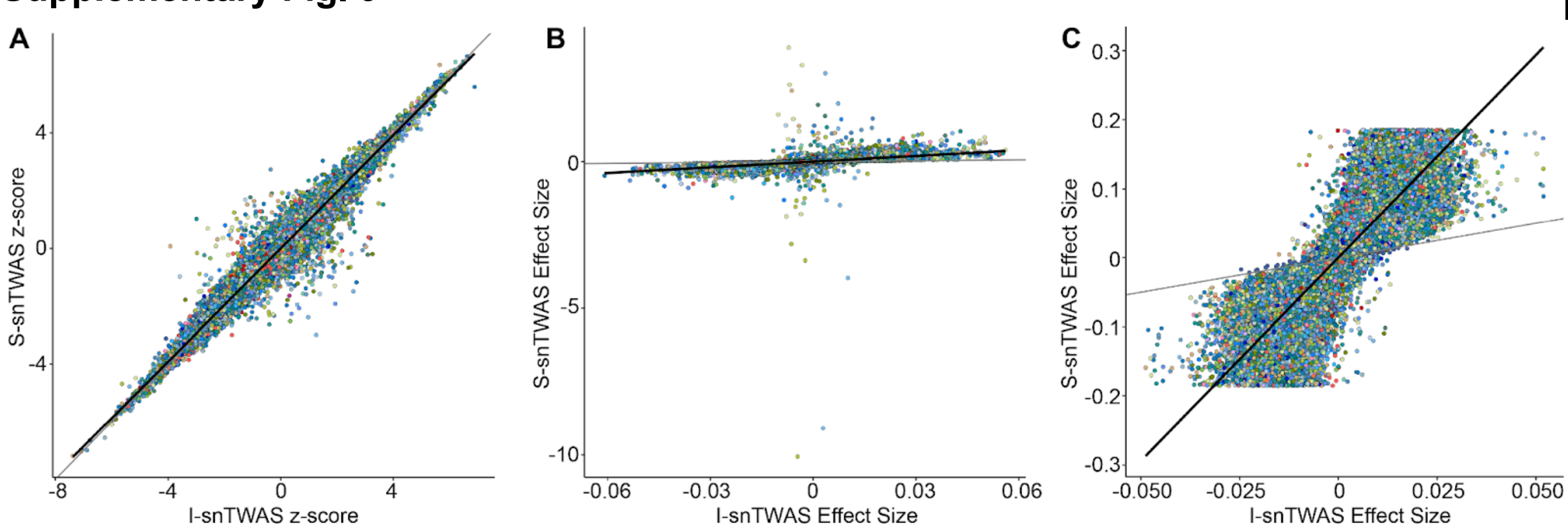<br><br>**Supplementary Fig. 9 | Z-score and effect size comparisons between S-snTWAS and I-snTWAS**. **A,** Z-score agreement across six overlapping traits (MDD excluded due to sample

	overlap). For S-snTWAS, GWAS were run in MVP using the same outcome and covariates as the individual-level analysis, and S-PrediXcan was applied. Pearson's correlation is 0.99 (p-value: $1.82 \times 10^{-583,345}$). The gray line represents y=x. **B,** Effect-size snTWAS agreement across the same six traits. Pearson's correlation is 0.77 (p-value: $2.87 \times 10^{-130,577}$). **C,** As in **B**, but zoomed after removing the top 1% of S-snTWAS effects by absolute value to emphasize the bulk of the distribution. The gray line represents y=x.

**REF3-8. mash resolution choice**	
Resolution Choices in MASH Analysis: More explanation on why class-level resolution was chosen over subclass in MASH analysis would be valuable for understanding the decision-making process for each part of the paper.	
**Response**	We recognize how important these methodological details are and have added Supplementary Fig. 32 comparing the mash analysis across all cell types versus only the six major classes.
**Action**	As shown in the accompanying figure, applying mash across all cell types identifies approximately 1.55 times (95% CI: 1.41-1.68) more significant associations per cell type than when applied only to the six major class-level cell types. To remain conservative, we therefore limited the mash analysis to these class-level cell types. We also note that we do not use significant gene-trait associations from mash for analyses beyond cell-type specificity identification, and we further restrict genes with identified cell-type specificity to those that are also significant in our TWAS analysis.
**Excerpt**	**Supplementary Fig. 32** 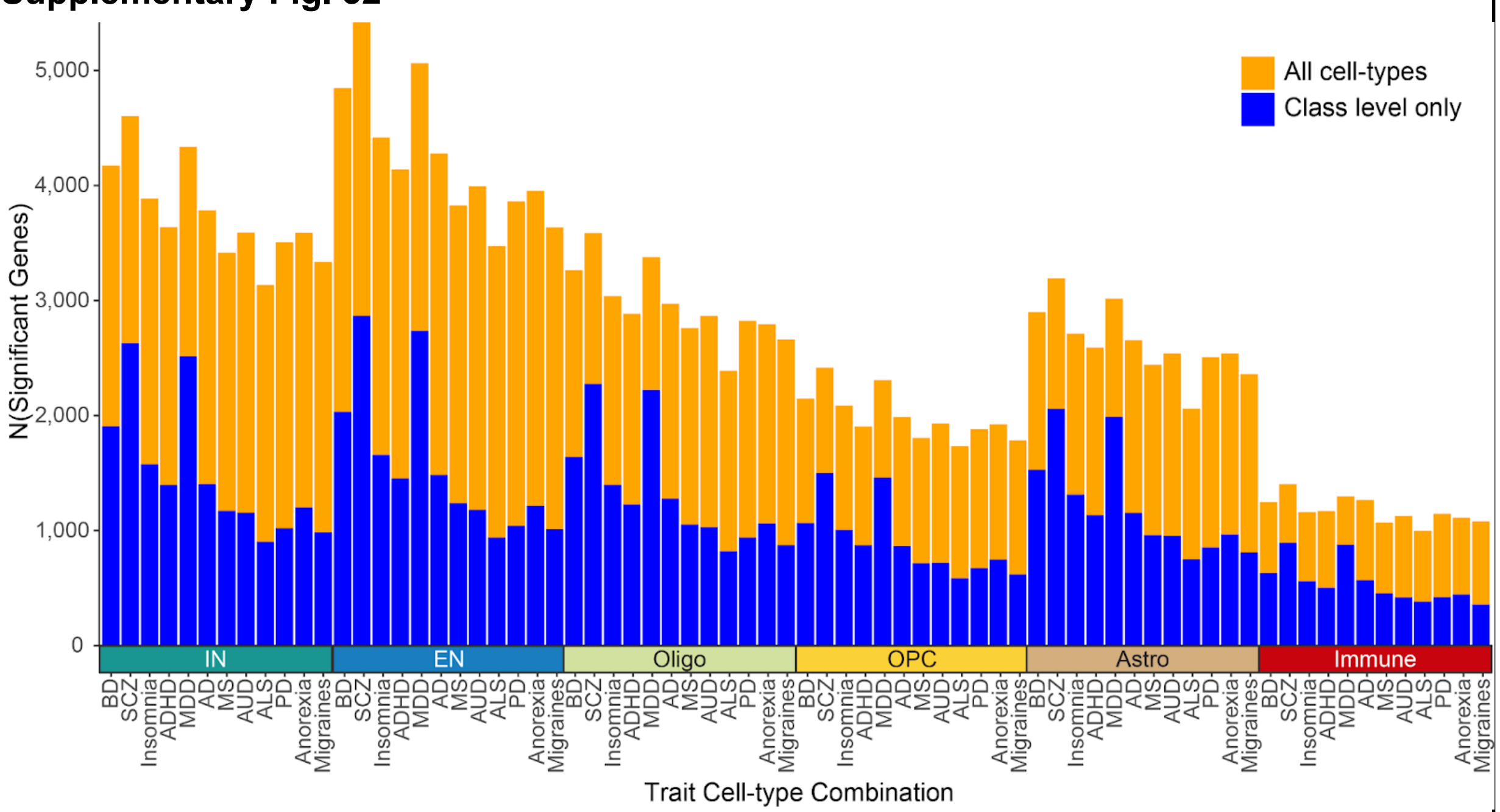

	**Supplementary Fig. 32 | mash fitting comparison.** The blue bar indicates how many significant genes mash finds when fitted amongst the main 6 classes. The orange bar indicates how many significant genes mash finds when fitted amongst all 32 EUR snTIMs.

**<u>REF3-9. Cell-type sn-eQTL quantification</u>**	
The statement “largely driven by cell-type specific single-nucleus expression quantitative trait loci (sn-eQTL; Extended Data Fig. 5B)” would benefit from more quantitative backing. Additionally, more clarification is required on whether these are cell-type-specific eQTLs and whether SNPs are non-overlapping.	
**Response**	We appreciate the reviewer’s point and acknowledge that quantitative support strengthens this statement. We have refined the analyses using established eQTL quantification methods and now explicitly clarify that the observed signals represent cell-type-specific sn-eQTLs, supported by largely non-overlapping lead SNPs across cell types. We also note this figure has been moved to Supplementary Fig. 11.
**Action**	We applied mmQTL to identify and fine-map *BIN1* eQTLs in the PsychAD EUR cohort. While some QTLs show minor overlap across cell types, the lead SNPs have low linkage disequilibrium (the maximum R^2 that rs6733839, the lead SNP for Class-Immune, has with any other lead SNP is 0.126), and the significance patterns vary markedly between cell types (Extended Data Fig. 3B). These findings illustrate quantitative support that *BIN1* expression regulation is heterogeneous across cell types (Fig. 3C).
**Excerpt**	**Supplementary Fig. 11**

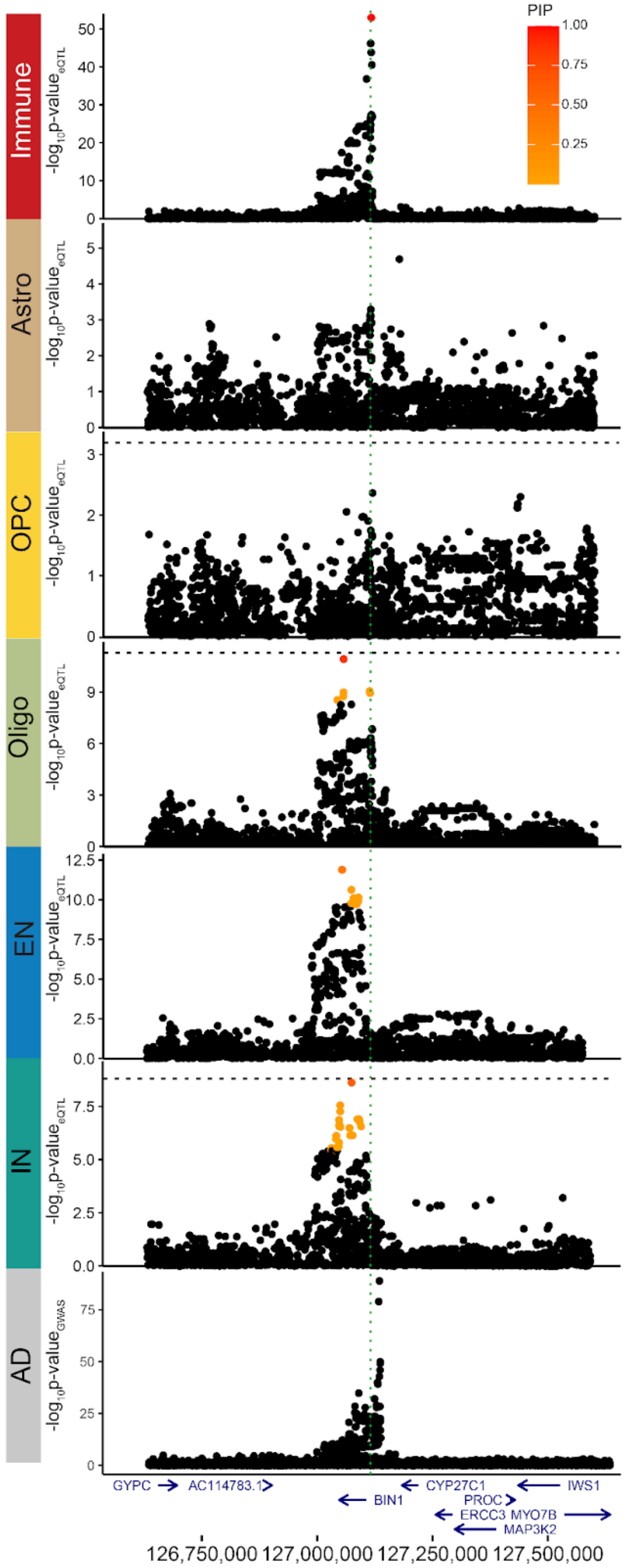

Supplementary Fig. 11 | Sn-eQTL manhattan plot for *BIN1*. Color indicates posterior inclusion probability from statistical fine-mapping. Presence of horizontal dotted line indicates top variants were below significance threshold. The green dotted line shows the position of the fine-mapped index SNP in Class-Immune. The top SNP for Class-Immune is rs6733839.

	Linkage disequilibrium for every eQTL SNP is indicated in Supplementary Fig. 12.<br><br>**Supplementary Fig. 12**<br><br>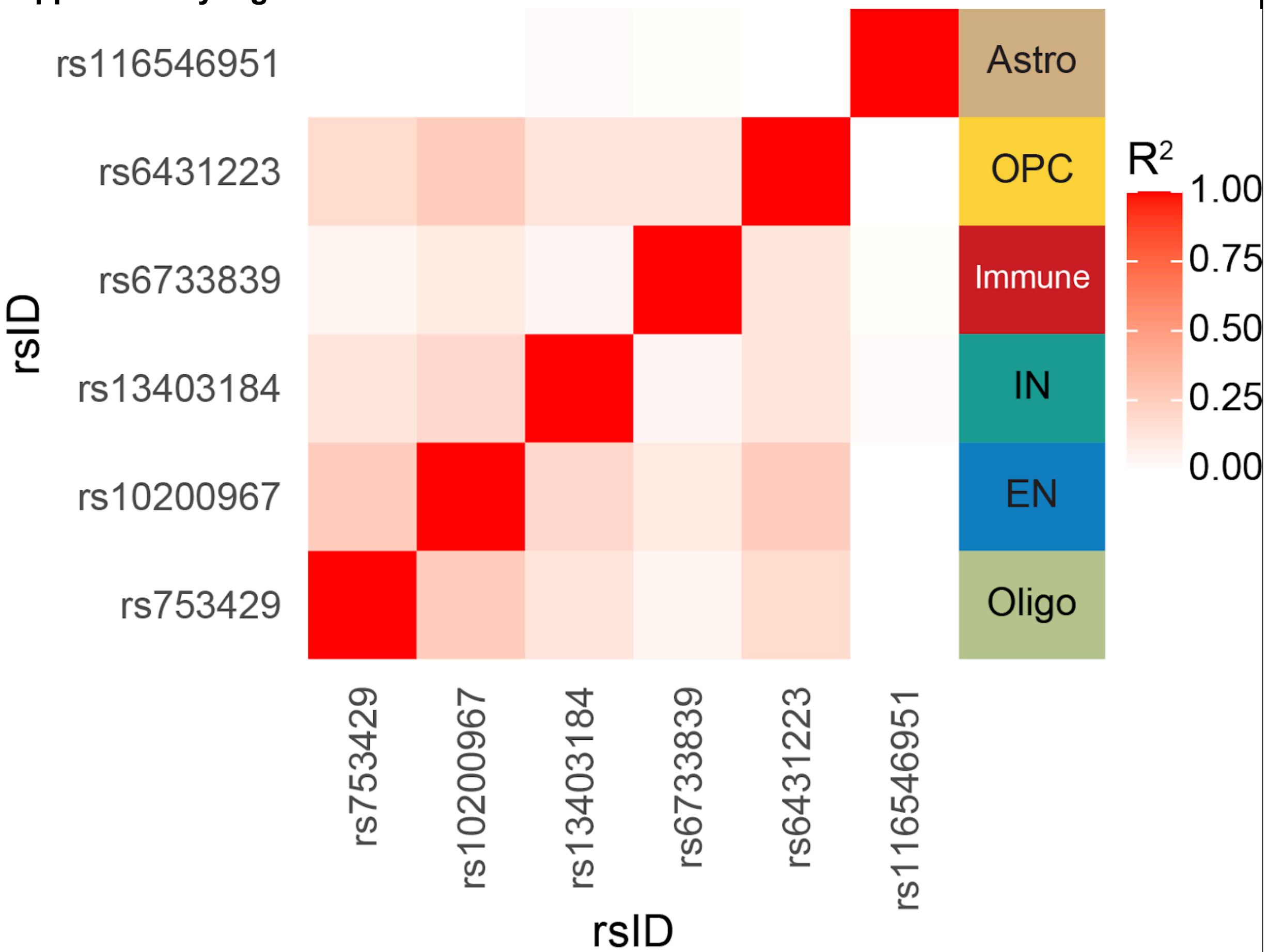<br><br>**Supplementary Fig. 12 | Linkage disequilibrium heatmap for top eQTL SNPs of *BIN1* per cell-type.** The R^2 between these SNPs is queried from publicly available information[37].

**REF3-10. Extended Data Fig. 3 legend**	
The legend panels for Extended Data Fig 3 are not aligned with the figure. A is B and vice versa.	
**Response**	Thank you for pointing out this labeling mismatch.
**Action**	We have corrected mismatched legends. The panels have also changed location. The previous Extended Data Fig. 3A is now Supplementary Fig. 1, and the previous Extended Data Fig. 3B is now Supplementary Fig. 26.

**REF3-11. Fig. 3C readability**

Increasing the size of symbols in Figure 3C will improve readability, making it easier to differentiate significant and non-significant dots.	
**Response**	We thank the reviewer for this valid suggestion to improve figure readability.
**Action**	We have implemented graphical changes to the plot in response.
**Excerpt**	“Points with significant heterogeneity (Q FDR-adjusted p-value ≤ 0.05; circles) are double the size, for better visualization.”

Referee #4 (Remarks to the Author):

Summary

This is a very well written piece, reporting on results from a timely endeavor for the field of psychiatric genetics. I’m not aware of similar studies in neuropsychiatry of this scale and scope. The methods are sound and the authors guard against overinterpreting findings by conducting a range of validation/sensitivity analyses for most findings. The findings are strengthened not only by the large number of nuclei and human donors but also the validation in an external dataset. I have mostly minor comments/questions/suggestions.

Comments

**<u>REF4-1. Abstract clarity: donors, associations</u>**	
In the abstract it’s unclear whether the donors are healthy individuals. It’s also unclear what the ‘associations’ are, with what?	
**Response**	This is a valid point. PsychAD includes a mixture of donors with various neuropsychiatric conditions (e.g., Alzheimer’s disease, schizophrenia, bipolar disorder) as well as unaffected controls[20]. Although disease status could theoretically influence genetic regulation of expression, prior studies have shown this to be minimal in eQTL and GReX analyses[21,22]. In addition, our pipeline applies PEER[23] residualization to remove hidden covariates, further reducing potential disease-related confounding.<br>We also acknowledge the ambiguity regarding the term ‘association’ and address it below.
**Action**	We revised the abstract to clarify both points. We now explicitly note that PsychAD includes donors both with and without neuropsychiatric diagnoses and that the term ‘association’ is used only in two contexts: *(i)* referring to the transcriptome-wide association study (TWAS) method, and *(ii)* denoting gene-trait associations, defined as the relationship between a gene’s genetically predicted expression and the corresponding trait.

**REF4-2. Disorder category definitions**	
Have the authors examined to what degree their findings are specific to neuropsychiatric disorders and how they are different between the broad category of neurological and the broad categories of substance use disorders and psychiatric disorders as cell type enrichment studies indicate there may be category-level differences in involvement of brain cells? Similarly, where are the definitions of NPDs and NDDs given? I would recommend adding a short section with a specific header about the phenotypes to the methods, including a rationale for the use of the phenotypes selected by the authors. From the reference list 79-90 I see the phenotypes. OCD seems missing. Although important updates of BIP, OCD and MDD GWAS are to be published in the coming months I see how the authors may not have excess to these phenotypes. I also see how often updated GWASs in psychiatry are published and it's impossible to have used the latest upon publication of new paper like the current. However, as these three GWASs show significantly increased SNPbased h2 relative to previous GWASs one may consider trying to get access to one or more of them (all PGC), particularly as the current paper will be published later. Alternatively, the authors may simply add OCD to their analyses as well as psychiatric cross-disorder sum stats to their analyses, as the latter captures the 8 main psychiatric disorders and has good statistical power.	
**Response**	We thank the reviewer for these thoughtful suggestions.<br>*Comparison of findings between disorder categories:* We agree that the inclusion of additional substance use disorders (**SUDs**) would enable a broader category-level comparison among neuropsychiatric disorders (**NPDs**), neurodegenerative disorders (**NDDs**), and SUDs. In response, we have now extended our analyses to include tobacco use disorder and cannabis use disorder alongside alcohol use disorder, the three SUDs with the most well-powered GWASs based on the number of genome-wide significant loci. The revised manuscript includes a supplementary analysis that contrasts NPDs, NDDs, and SUDs at both the gene and pathway levels.<br>*Utilization of novel upcoming GWAS:* As the reviewer notes, it is not trivial to update the study design to include newer GWASs as they become available, since at this scale multiple dependencies must be harmonized across data layers. As a good practice, we implement a GWAS version-control policy, using the newest public releases available before our predefined analysis freeze date. While we aimed to analyze a breadth of NPDs and NDDs, we did not aim for an exhaustive list since our manuscript focuses on high-level insights with a closer look at case studies, such as Alzheimer's disease, bipolar disorder, and alcohol use disorder. Beyond expanding the list of SUD GWASs to enable a comparative supplementary analysis for broad disorder categories (Supplementary Note 7), the improved GWAS summary statistics were not yet available by the GWAS freeze date. To ensure that researchers gain access to appropriate data for their analyses, we will release the snTIMs publicly so that others can integrate newer GWASs, including the latest PGC releases, using standard tools.
**Action**	Action items:<br>1. Added a Methods subsection: "Phenotypes and Category Definitions."<br>a. Defined NPDs, NDDs, and SUD categories.<br>b. List all traits analyzed and map each to a category.

	c. State inclusion/exclusion criteria and data sources; justify chosen phenotypes.<br>2. Clarify phenotype selection rationale (availability, sample size, power, relevance) and acknowledge rapid GWAS updates; state that analyses are date-locked.<br>3. Added a sentence in the Results (with full analysis in the Supplement and accompanying figures) on category-level specificity, explicitly comparing enrichment/signals across NPD/NDD/SUD. SUD now includes AUD, tobacco, and cannabis use disorders<br>We have added a brief analysis comparing NPDs, NDDs, and substance use disorders (**SUD**s).
**Excerpt**	**Supplementary Note 7. Comparison of NPDs, NDDs, and substance use disorders**<br>Following our cross-disorder analysis, we investigated whether S-snTWAS correlation could identify differences in major classes of disorders. We classified 14 traits into NPDs, NDDs, and substance use disorders (**SUD**s) and analyzed trends of correlations. Overall, we found that the greatest similarity in associations is generally within each disorder category (e.g. one NPD compared with another NPD) (Supplementary Fig. 29A). Secondly, we found SUDs were more associated with NPDs and NDDs than NPDs were associated with NDDs. While this is mostly explained by genetic correlation of the underlying GWAS, the association between NDDs and SUDs is slightly stronger when comparing S-snTWAS as opposed to comparing GWAS (Supplementary Fig. 29B), even though GWAS correlation values and TWAS correlation values were themselves very correlated (Spearman's $\rho = 0.78$; p-value = 1.20×10^{-19}; Supplementary Fig. 29C). We found that this increase was largely explained by the association between AUD and NDDs (Table S38). Interestingly, while GWAS correlation between AUD and NDDs is generally low, we found significant enrichment when comparing AUD and AD (Fig. 4A; 62 gene-cell-type combinations in common; Fisher's exact test FDR-adjusted p-value = 1.95×10^{-49}). Finally, clustering analysis of TWAS correlations separates NPDs and NDDs, but SUDs are scattered throughout (Supplementary Fig. 14). Cannabis use disorder and tobacco use disorder cluster strongly with NPDs such as MDD, ADHD, and insomnia, whereas AUD clusters more closely with NDDs such as AD, PD, and ALS. This suggests that while NPDs and NDDs can be separated much more easily, SUDs cannot.<br><br>**Methods: GWAS phenotypes and category definitions**<br>Traits for S-snTWAS analysis were drawn from publicly available brain disorder GWAS. To stabilize downstream analyses, inputs were date-locked in 2024. At the time of publication the PsychAD snTIMs trained in this project will be publicly released so users can freely apply these models to newer datasets.<br>We began with 16 traits. Four showed insufficient power for S-snTWAS when analyzed individually, each yielding at most one FDR-significant gene: binge-eating disorder[79], post-traumatic stress disorder (**PTSD**)[80], anxiety[81], and obsessive-compulsive disorder[82]. Our primary analyses therefore use 12 well-powered NPD and NDD GWAS summary statistics[80,83–93] (Table S10).

	For targeted comparisons of I-snTWAS versus S-snTWAS (e.g. Fig. 5A), we retained anxiety[81] and PTSD[80] GWAS, since both traits had adequate power for analysis in MVP (see below). For select analyses, traits were grouped as neuropsychiatric disorders (**NPD**s), neurodegenerative disorders (**NDD**s), and substance use disorders (**SUD**s). These groupings are analytical and not intended to mirror formal diagnostic taxonomies. We organized traits to maximize interpretability, guided by shared genetic architecture and hypothesized dominant cell-type and pathway involvement. NPDs included migraines, schizophrenia (**SCZ**), bipolar disorder (**BD**), anorexia (anorexia nervosa), insomnia, attention-deficit/hyperactivity disorder (**ADHD**), and major depressive disorder (**MDD**). NDDs included Alzheimer's disease (**AD**), Parkinson's disease (**PD**), amyotrophic lateral sclerosis (**ALS**), and multiple sclerosis (**MS**). For primary analyses we used only alcohol use disorder (**AUD**) from the SUD group; however, for category-level comparisons, SUD representation was expanded to include tobacco use disorder (**TUD**) and cannabis use disorder (**CUD**).<br><br>**Supplementary Fig. 29**

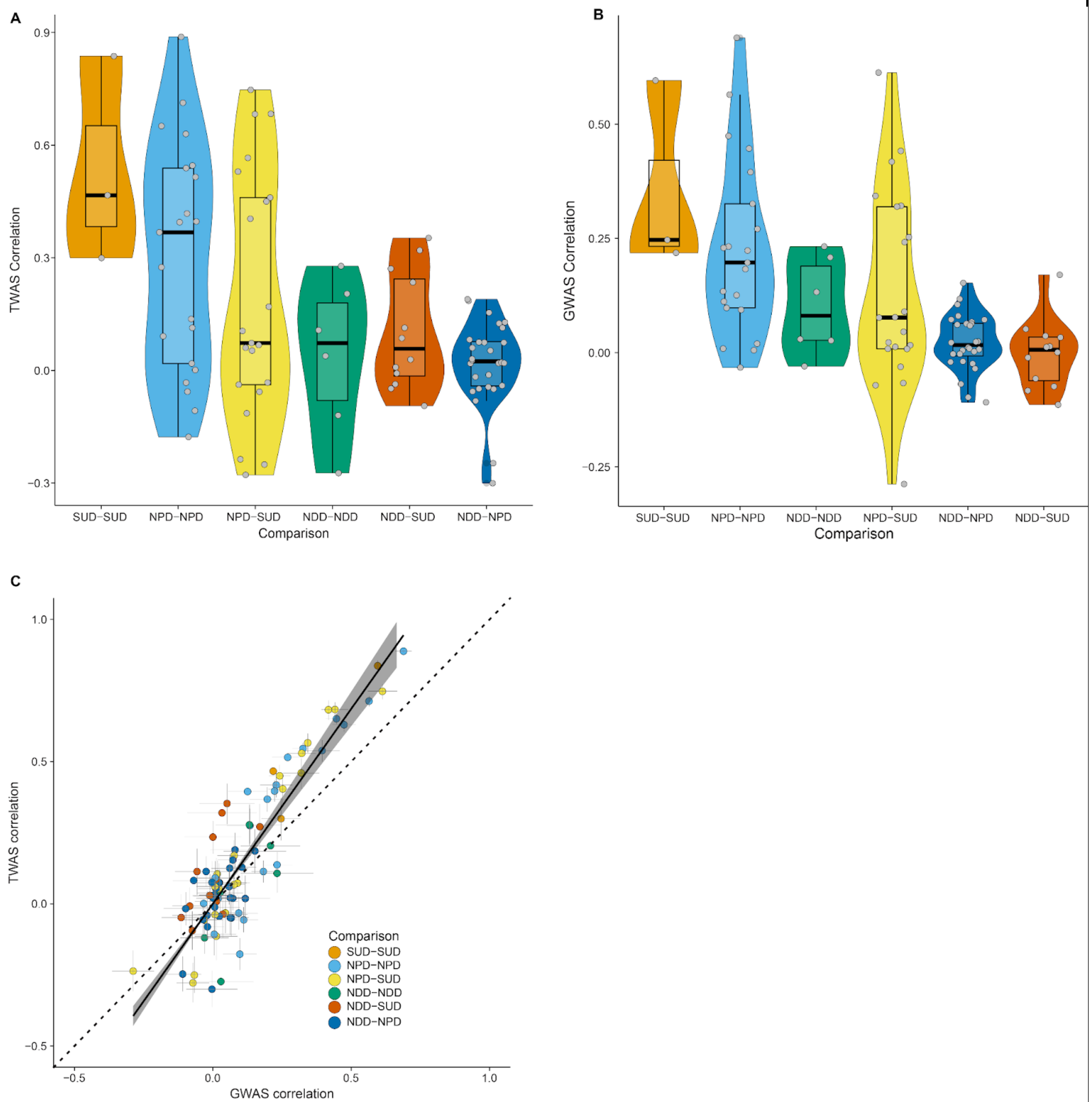

Supplementary Fig. 29 | Comparison of NPDs, NDDs, and substance use disorders. A, Violin plot of S-snTWAS Pearson's correlation (y-axis) of disorders corresponding to the comparison category indicated on the x-axis. Table S10 indicates the annotated disorder category for each disorder. Internal box plot indicates the median, 25% and 75% quantiles. The vertical line ranges from median + 1.58 x IQR / sqrt(N) to median − 1.58 x IQR / sqrt(N), where IQR refers to the inter quartile range and N refers to the number of points. Violin plots have been scaled to have equivalent maximum width. **B,** Violin plot of GWAS correlation (y-axis; calculated with LDSC) of disorders corresponding to the comparison category indicated on the x-axis. Table S10 indicates the annotated disorder category for each disorder. Internal box plot indicates the median, 25% and 75% quantiles. The vertical line ranges from median + 1.58 x IQR / sqrt(N) to median − 1.58 x IQR / sqrt(N), where IQR

	refers to the inter quartile range and N refers to the number of points. Violin plots have been scaled to have equivalent maximum width. **C,** Scatterplot of GWAS correlation versus S-snTWAS correlation. Color refers to the comparison category. Dashed line refers to the y=x line, and lines attached to each point indicate the confidence interval in regards to S-snTWAS correlation (vertical) and GWAS correlation (horizontal). Black line and attached gray shaded region refer to the line of best fit (intercept set to 0) and confidence interval for line of best fit, respectively. (Spearman's $\rho = 0.78$; p-value = 1.20×10^{-19}).

**<u>REF4-3. Cross-region and mouse alignment</u>**	
Have the authors considered comparing these DLPFC findings w/ previous findings from other brain regions, eg either in an analysis when such data are available or in the discussion by comparisons with findings from the literature? How are the findings to be interpreted in light of single cell findings of donors with psychiatric disorders? Similarly, how about findings in mouse models? Have the authors considered providing a short, (supplemental) discussion about alignment with findings from mouse donors?	
**Response**	We thank the reviewer for the opportunity to clarify the scope of our DLPFC-focused analyses and the rationale for our choices; below we address cross-region context, disease-state single-cell findings, and mouse alignment within the bounds of the current study.<br><br>*<u>Scope (brain regions).</u>* Our study was designed around DLPFC single-nucleus RNA-seq with matched genotypes, and all snTIMs/TWAS results derive from this region. To our knowledge, sufficiently powered genotype-linked snRNA-seq resources for other brain regions are not yet publicly available at a scale comparable to PsychAD, so we did not perform cross-region analyses in this work. Prior studies suggest that much apparent cortex-to-cortex variation reflects differences in cell-type composition rather than distinct cis-genetic control[39], with potentially different patterns in subcortex[40]. As region-matched datasets emerge, we plan to evaluate portability and to train region-specific snTIMs to test generalizability beyond DLPFC.<br><br>*<u>Relation to disease-state single-cell findings.</u>* By design, TWAS/GReX models estimate only the *cis*-genetic component of expression and do not capture environment, medication, or disease-state effects (see left panel of included figure below). Consistent with this framework, we treated differential gene expression (**DGE**) comparisons as conceptually distinct and did not conduct a systematic alignment to disease-state snRNA-seq DGE signatures across psychiatric cohorts in this manuscript. In prior DLPFC homogenate studies, global correlation between GReX[8] and DGE[41] were not significant (p-value = 0.14, Pearson's correlation; see right panel of included figure below), which is expected given the different quantities estimated. We did not extend this comparison to single-nucleus datasets here; based on observed R^2_{CV} of snGReX models, we similarly expect GReX to explain a modest fraction of total expression variance.

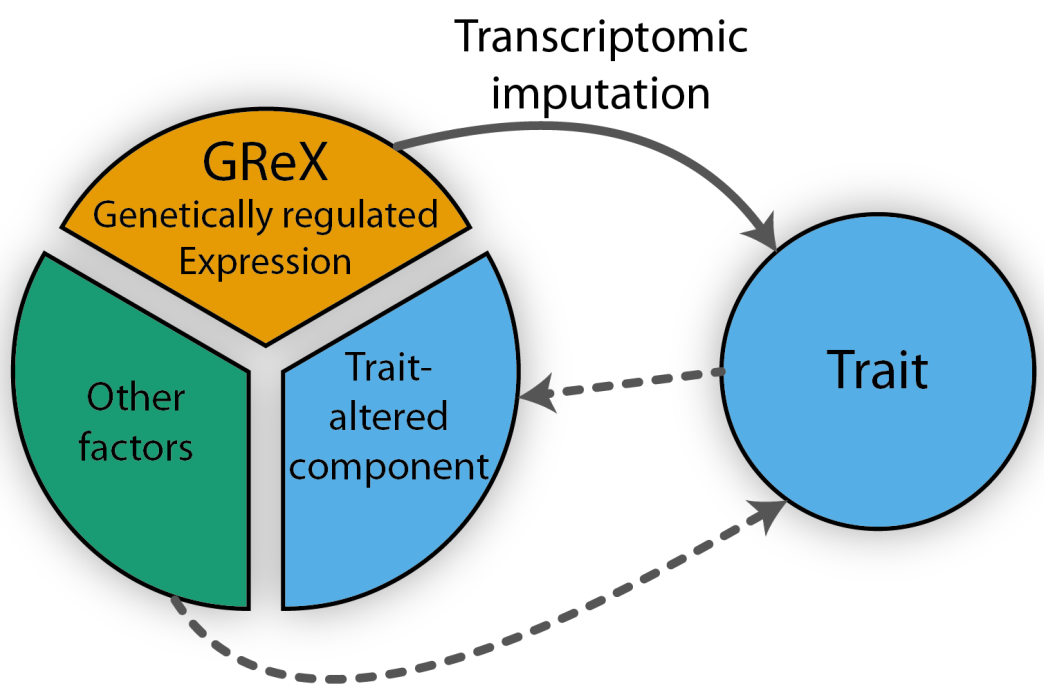

Imputation of genomic features. Conceptual decomposition of the expression level of a gene into three components: a genetically regulated expression (**GReX**) component, a component altered by the trait itself and a component determined by the remaining factors (including environment).

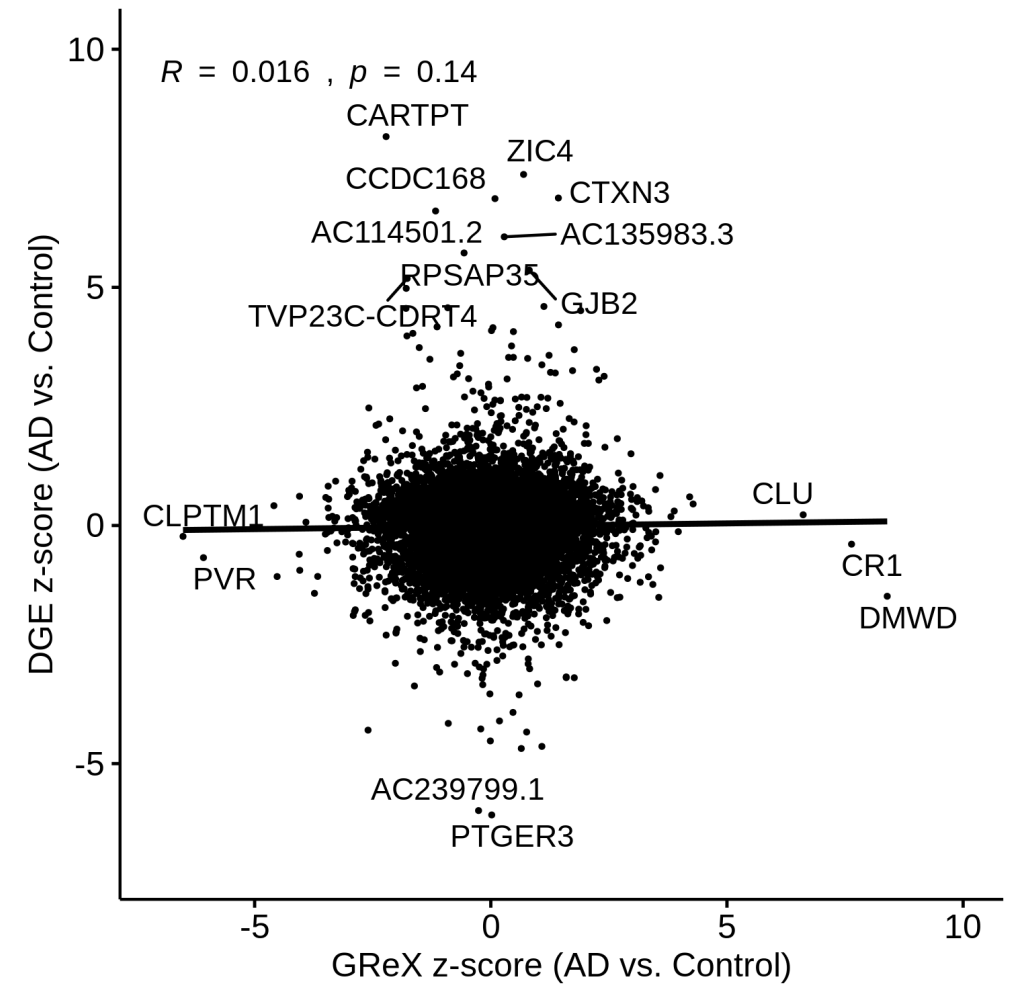

Comparison of GReX[8] and differential gene expression (**DGE**)[41] in AD at the RNA level. Genes with absolute z-score values greater than 5 are labeled. R and p are provided for Pearson's correlation.

Instead of disease-state alignment, we prioritized validation that our snTIMs capture genetically regulated signal out-of-sample: *(i)* agreement between internal cross-validation and ROSMAP snRNA-seq, and *(ii)* concordant signals between PsychAD microglia snTIMs and an independent FACS-sorted microglia TIM (including highly correlated AD snTWAS z-scores). These observations mirror prior reports of GReX reproducibility in tissue homogenates[8]. We also report close S- vs I-snTWAS agreement when using the same genetic data, supporting the calibration of summary-level z-scores.
Together, these results indicate our models recover GReX accurately; divergence from DGE is expected.

Mouse alignment. We did not include a new cross-species alignment in this study. In prior bulk DLPFC TWAS work[8], enrichment of human TWAS hits among mouse phenotype orthologs within matching broad categories was weak, whereas enrichment against human clinical gene sets was clearer. Given the anticipated low yield and scope limits here, we did not pursue mouse alignment analyses for snTWAS.

**Action**	We now state explicitly that all models are DLPFC-derived and validated out-of-sample (ROSMAP snRNA-seq; FACS-microglia), and that S- and I-snTWAS z-scores agree closely while effect-size estimates from summary methods should not be used for heterogeneity tests.

REF4-4. Research and clinical implications

This taps into general comment about the balancing between results and discussion. The results section is lengthy, detailed and largely technical. A discussion section with more room for the implications of the findings (research + clinical) and the interpretation in light of the current literature may help balance the piece. Generally, the discussion falls short on research and clinical implications of the findings and continues along the same, technical lines.	
**Response**	We appreciate this insightful comment and similar feedback from other reviewers. We agree that the original Results section was overly technical and that the Discussion did not sufficiently emphasize the broader research and clinical implications of our findings. We have addressed this by expanding the Discussion to highlight the interpretive and translational significance of our results and by improving the overall balance between sections.
**Action**	We implemented several changes to address this comment:<br>1. Expanded Discussion: We added new paragraphs emphasizing how our findings inform mechanistic insight and potential clinical applications across neuropsychiatric and neurodegenerative disorders.<br>2. Integration with prior work: We compared AD S-snTWAS results with human microglial co-expression networks, using the CAMERA method to demonstrate that overlapping modules capture convergent, disease-relevant biology.<br>3. Improved readability: Approximately one page of highly technical results was moved to the Supplementary Information to streamline the main text and better emphasize overarching conclusions.
**Excerpt**	Expanded Discussion<br>To validate and interpret these pathway-level signals, we performed a microglia-focused, module-level analysis in AD using a published MEGENA[57] network from freshly isolated human microglia[26]. S-snTWAS module enrichments closely tracked DEG signatures associated with AD pathology, predominantly in Class-Immune and for CDR and Braak, identifying three negatively enriched immune modules also associated with amyloid plaque burden. These modules highlighted altered myeloid programs and *TREM1* dysregulation and prioritized the *ZYX*/*EPHA1-AS1* locus, implicating *ZYX* as a putative coding effector whose predicted downregulation may predispose cells to impaired stress responses to β-amyloid exposure[41]. Together, this AD case study shows how cell-type-resolved TWAS bridges genetic signals to microglial co-expression modules anchored to AD pathology.<br><br>Integration with prior work<br>While pathway-level analyses revealed broad cell-type-specific perturbations, we next examined whether S-snTWAS recapitulate and extend known associations with brain pathology, using AD as a case study. To assess microglial contributions, we leveraged a hierarchical co-expression network analysis generated in freshly isolated human microglia spanning healthy and neurodegenerative aging[26]. We first tested microglial co-expression network modules for competitive enrichment of AD S-snTWAS signals (see Methods). Class-Immune AD associations showed the strongest correspondence with AD pathology-linked differential expressed gene (**DEG**) signatures[26] (Extended Data Fig. 4A) that

	was not present in other cell-types. Three modules (FDR-adjusted p-value ≤ 0.05) were significantly enriched for both AD-related differential expression and β-amyloid plaque density, and all exhibited negative enrichment (reduced predicted or observed expression in AD; Extended Data Fig. 4B; Table S21). Functional annotation of these modules highlighted immune and myeloid activation programs, including module M824, which mapped to myeloid gene expression, and a parent-child pair (M406→M947) pointing to *TREM1* dysregulation (Extended Data Fig. 4C; Table S22). Both M406 and M947 contained *ZYX* (Zyxin), which our S-snTWAS predicts to be downregulated in AD (Class-Immune: FDR-adjusted p-value = 9.18×10^{-14}, z-score = −8.29; Subclass-MG: FDR-adjusted p-value = 1.61×10^{-13}, z-score = −8.21) and fine-mapped with essentially complete posterior support (FOCUS PIP = 1.0 and 1.0; Table S16). Notably, *ZYX* did not emerge in differential-expression analyses (Extended Data Fig. 4D). The same modules also included *EPHA1-AS1*, recently linked to AD[26], which is located at the same GWAS locus as *ZYX*, underscoring the genetic and functional complexity of this region. Together, these results prioritize *ZYX* as a putative coding effector at this locus and link its downregulation to impaired microglial stress responses to β-amyloid[41].

**REF4-5. Define abbreviations**	
Some of the abbreviations are not explained upon their first occurrence, eg ‘R2<br>94 CV ≥ 0.01, pCV ≤ 0.05’, which sometimes makes reading difficult. Here, not only may these abbreviations be best explained in the results, but also their meaning (‘Imputable genes are considered passing cross-validation R2 (R2 CV) ≥ 0.01, pCV ≤ 0.05 and<br>SNPs in model > 0. R2 CV and pCV values are prediction performance R2 and prediction performance value from the “PredictDB” software75.’).	
**Response**	We agree that the original manuscript did not consistently define abbreviations upon first use, which could impede readability.
**Action**	We have reviewed the entire text to ensure that each abbreviation is defined and explained at its first occurrence. We also added brief contextual descriptions in the Results section clarifying their meaning and relevance.

**REF4-6. Thresholds and precision reporting**
Here and there the authors report in their methods the statistical test they apply but not how to interpret the results. Are they significant from a certain p-value threshold (corrected for multiple testing when applicable) or do they caution against using a p-value to infer significance and prefer to give an ES? Eg ‘We performed linear regression to assess the extent to which major snTIM metadata predictors (sample size and median number of nuclei contributing to the pseudobulk expression from every individual) influence snTIM performance (proxied by number of confidently imputed genes). We report the adjusted R2 as a measure of the variation explained in snTIM performance for each predictor;

adjusted R2 was estimated by the stats package76,77.' And 'We compared whether genes were more strongly imputed in psychAD snTIMs or Bulk using a sign test. To more accurately calculate the sign test p-value, we utilized Ramanujan's factorial approximation.' Generally, I would be expecting the authors could give confidence intervals and SE more often, e.g. when they report an OR. These may be better estimates of precision than p-values for some inferential stats.	
**Response**	We appreciate this helpful comment. We agree that our original manuscript did not always make explicit how statistical results should be interpreted, nor did it consistently report measures of precision beyond p-values.
**Action**	We revised the Methods and Results sections to clarify the interpretation framework for all statistical analyses. Specifically:<br>1. Significance thresholds: We now explicitly state the p-value and FDR thresholds used to determine statistical significance for each test.<br>2. Effect sizes and precision: Where applicable, we added effect sizes, confidence intervals, and/or standard errors alongside p-values (for example, for odds ratios, regression coefficients, and enrichment tests) to better convey precision and magnitude of effects.

**REF4-7. Streamline early Results**	
The first two, lengthy sections of the results give me the impression this is a methodological paper. If this is the authors' intention and the authors and editors deem these sections ('Brain snTIMs capture brain GReX with cell-type specificity across ancestries' and 'Brain snTWAS increases power for both known and novel gene-trait 144 association discovery.') indispensable for the general understanding of the piece, I would keep. Alternatively, one may consider shortening these sections a bit, moving them to the supplements or merging them, using one, more general header that may also be more understandable for non-experts. My personal impression is that the exciting part of the paper lies more in the results discussed in the abstract that could me emphasized more, e.g. by moving them up in the results section. However, if the authors deem these methodological parts of their work just as valuable they may consider outlining somewhere in the intro that there were two general goals to this work and clearly structuring the piece accordingly.	
**Response**	We appreciate this thoughtful suggestion aimed at readability for non-specialists.
**Action**	We streamlined the first two Results sections by tightening narrative text and relocating highly technical descriptions (e.g., model-training/validation particulars and implementation details) to the Supplementary Results/Methods. The revised sections now foreground their key take-home messages (performance needed to support discovery and added power at single-nucleus resolution) and more directly point readers to the subsequent biological findings highlighted in the Abstract. If the editor prefers further condensation, we are happy to merge these two sections under a single, general header without changing content.

**REF4-8. Discussion results summary**

I suggest the first paragraph of the discussion discusses not only the main methods (as in its last sentence) but then summarizes the results in 1-2 additional sentences.	
**Response**	We agree and appreciate the suggestion.
**Action**	We revised the opening paragraph of the Discussion to retain brief methodological context and immediately add one to two sentences that summarize the principal results, namely overall model performance and validation plus the key biological and trait-level discoveries, which provides a clearer bridge from Results to interpretation.

**REF4-9. Pooled enrichment OR**	
It's helpful to read about the performance in comparison to bulk for each phenotype under investigation. Again, many details are given. Can the authors compute a pooled estimate for the enrichment OR across diseases to obtain a summary estimate for this goal of comparing the yield of their method to bulk and other methods? It would be good to see precision estimates here for all phenotypes separately and then jointly. Generally, some of the results sections may benefit from a one or two-sentence summary in the end to highlight the most relevant of findings and bridge the gap to the next section.	
**Response**	We agree this will make the results more immediately interpretable.
**Action**	We now report a pooled enrichment estimate across diseases in each panel of Fig. 2, alongside precision estimates (per-phenotype and pooled). The pooling procedure and computation of confidence intervals are described in REF4-11. We also added brief, 1-2 sentence takeaways at the end of longer Results subsections to highlight the key findings and provide smoother transitions.

**REF4-10. Fig. 1 organization**	
Fig 1. see comment above about the first two headers. I wonder whether this figure is needed and would prefer the methodological figure as figure 1 (now ED F1), also considering not every reader of Nature may be familiar w/ all methods.	
**Response**	We appreciate this suggestion to improve accessibility for a broad readership.
**Action**	We streamlined Fig. 1 by removing two panels, simplifying annotations and caption text, and moving several dense explanatory blocks to the Supplementary Notes. We retained the detailed methodological schematic as ED Fig. 1 for readers seeking depth. If the editor prefers, we are happy to promote ED Fig. 1 to the main figure set without further changes.

**REF4-11. Pooled OR in Fig. 2**

Fig 2. can the authors compute a pooled estimate for the enrichment OR across diseases or wouldn't this be methodologically sound (see above)?	
**Response**	We agree that a pooled summary is useful and methodologically sound when aggregating trait-specific enrichments.
**Action**	For panels that report enrichment odds ratios (Fig. 2B and 2C), we now include a pooled estimate across diseases. We pool log ORs using inverse-variance weighting and report 95% confidence intervals alongside the per-phenotype estimates. We document the model choice and heterogeneity statistics in the Supplementary Methods. For panels that present counts or averages rather than ORs (Fig. 2A and 2D), we added All or Average summaries to aid interpretation. We also added one to two sentence takeaways at the end of the relevant Results subsection to highlight the main points.

**REF4-12. Fig. 3B: Stacked bar ordering**	
Fig 3. if B are stacked bar charts why is the ordering from bottom to up discrepant across stacks? The figure is hard to grasp currently. Why not order from bottom to up equally for all phenotypes, so blue would always be up? As suggested above, I would be interested in seeing neurological, SUD and psychiatric phenotypes as three separate categories.	
**Response**	Fig. 3B uses within-column ordering from the most to the least represented cell type so that the dominant cellular contributors appear at the top of each phenotype. Colors map consistently to cell types across all stacks. We did not separate neuropsychiatric, neurodegenerative, and substance use disorders in this specific figure. Category-level comparisons for these groups are presented using TWAS and GWAS correlation analyses in Supplementary Fig. 15. Disorders in that analysis are ordered by Ward's hierarchical agglomerative clustering on S-snTWAS Pearson correlations.
**Action**	We added clear language to the Fig. 3B caption explaining the ordering rationale and noting the fixed color-to-cell-type mapping. We also added a pointer to the category-level comparison described in the response to REF4-2 and cited in the section "Comparison of NPDs, NDDs, and substance use disorders" in the Supplementary Information.
**Excerpt**	**Fig. 3B Caption:**<br>Added "Cell-types are ordered by their frequency in trait-specific mash GTAs."

**REF4-13. Update AUD terminology**
Fig 6. 'alcoholism' is a bit of an archaic term that is more adequately written in the legend with "" than in B. is it defined somewhere? In C it has an asterisk that I was unable to locate in the caption. I generally

recommend using up-to-date idiom like alcohol use disorder whena applicable or ‘alcohol-related disorders’ or something of the like and explaining in methods their meanings.	
**Response**	Thank you for flagging this. We used “alcoholism” to mirror the original MVP phecode label, but we agree it is outdated and may be confusing.
**Action**	We replaced all instances of “alcoholism” in the text, figures, and tables with “alcohol use disorder” (AUD). We updated Fig. 6 panel labels, legends, and captions accordingly, including panel B. We removed the stray asterisk in Fig. 6C and ensured all annotations are defined in the caption. We added a brief terminology note in the Methods and a supplementary table that maps MVP phecodes to the clinical terms used in the manuscript for clarity and traceability.

Referee #5 (Remarks to the Author):

Summary

In "Single-nucleus transcriptome-wide association study of human brain disorders," Venkatesh et al. present results of a cell type specific transcriptome wide association study performed in single-nucleus sequencing data derived from dorsolateral prefrontal cortex samples collected from ~1500 donors. They used the estimated snp effects to derive expression prediction models and then performed analyses of genetically predicted cell type specific gene expression for a range of psychiatric and neurodegeration traits to discover novel gene-trait associations. MVP was used to validate findings. This is an interesting paper and the suggestion that gene trait associations estimated from genetic regulation of expression in bulk measures from tissues comprising heterogeneous tissue types (effectively “masking” cell type specific effects in bulk analyses) is a sensible premise warranting exactly this kind of comparative analysis. However, I have some statistical/bioinformatic concerns that may bias some of the main observations in the manuscript, and it will be important to address these (or at least clarify why they are not relevant in the main text for the reader) the ensure the findings are robust.

Comments

**<u>REF5-1. External snTIM validation</u>**
A major comment I have is that in our hands, we have found that ten fold cross validation R2 estimates can be inflated in certain circumstances. I'm concerned that the dependence of the process of distinguishing the cellular populations prior to the cell type specific model building (which uses the global expression patterns) may exacerbate this problem. Similarly, use of corrections that utilize the full data (e.g. PEER). This kind of feature engineering can be problematic and cause data leakage in k-fold performance estimation. Given that the sample size is fairly robust here, it would be reassuring if an experiment was performed in a hold out set that is uncontaminated (separate QC, separate normalization, etc) to assess performance of the

snTIMs and show that the cv R2 estimates are largely similar to performance in independent data. This could be done as a sensitivity analysis so as not to interfere with the main results of the paper. The reason this matters so much is because of the comparisons to the ten fold cross validation performance of the bulk models- if the authors' models are differently biased/inflated by the shared additional data processing of the snRNAseq data (aka data leakage), these results could be invalid.	
**Response**	We agree that external validation is the most rigorous way to assess generalizability and to guard against leakage.
**Action**	In response, we performed out-of-sample validation using two independent cohorts: *(i)* ROSMAP snRNA-seq and *(ii)* FACS-isolated microglia (FACS-MG). Each dataset was processed with separate QC and normalization pipelines. For both, we compared predicted genetically regulated expression (GReX) from PsychAD snTIMs with observed residualized expression, finding strong concordance between cross-validated (R^2_{CV}) and out-of-sample (R^2_{out}) performance (Spearman ρ = 0.63 for ROSMAP, ρ = 0.55 for FACS-MG). We found no evidence to indicate feature-engineering leakage or inflation of R^2_{CV} estimates; in fact, the α of the linear regression (y = α × x) tilted in favor of R^2_{out} for microglia (see figure below).
**Excerpt**	**Supplementary Note 5. Out-of-sample validation of snTIMs and S-snTWAS**<br>First, in the ROSMAP cohort, we applied the PsychAD cellular taxonomy to cluster and pseudobulk the snRNA-seq data. We then imputed cell-class-specific gene expression using ROSMAP genotypes and the PsychAD snTIMs. Comparing imputed GReX with observed gene expression in ROSMAP yielded R^2_{out} estimates that closely matched our R^2_{CV} predictions (Spearman's ρ = 0.63; N = 70,156; p-value = $3.91 \times 10^{-7,844}$; Supplementary Fig. 27A), demonstrating external validity and showing no evidence for feature-engineering-induced leakage which can inflate R^2_{CV} estimates. Next, we performed MDD S-snTWAS using our PsychAD snTIMs and compared these results to a recently published independent ROSMAP-based S-snTWAS (**Zeng-2024**)[21]. After matching cell classes across cohorts (Table S37), we correlated the S-snTWAS z-scores based on the same MDD GWAS summary statistics[129]. Despite differences in cohorts (PsychAD vs. ROSMAP), cell clustering/taxonomy, TIM methods (PrediXcan vs. FUSION[130]), and sample sizes (920 vs. 424), we observed strong concordance (Pearson's $r_{(13,760)}$ = 0.78, p-value = $1.34\times10^{-2,772}$) between z-score sets (Supplementary Fig. 27B). By simple counts, we observed 6,095 more imputable gene-cell-type combinations (PsychAD: 26,920; Zeng-2024: 20,825) and 31 more significant gene-cell-type associations (PsychAD: 335; Zeng-2024: 304) under the same FDR-adjusted p-value threshold, consistent with broader coverage and a higher discovery yield.<br>Second, to validate microglia-specific findings across different experimental designs, we compared our PsychAD microglia subclass snTIM (Subclass-MG; N = 904) with a smaller EUR TIM which we constructed with FACS-MG data (N = 271)[127,128]. Although these models share only 22 donors and employ different expression-profiling methods, we observed strong per-gene consistency between R^2_{CV} and R^2_{out} estimates (Spearman's ρ = 0.55; N = 1,853, p-value = 1.67×10^{-149}; Supplementary Fig. 27C). Consequently, performing S-TWAS for AD

(selected for its known microglial relevance[131,132]) with both TIMs yielded very strong z-score concordance (Pearson's $r_{(733)} = 0.87$, p-value = 1.32×10^{-231}; Supplementary Fig. 27D).

Supplementary Fig. 27

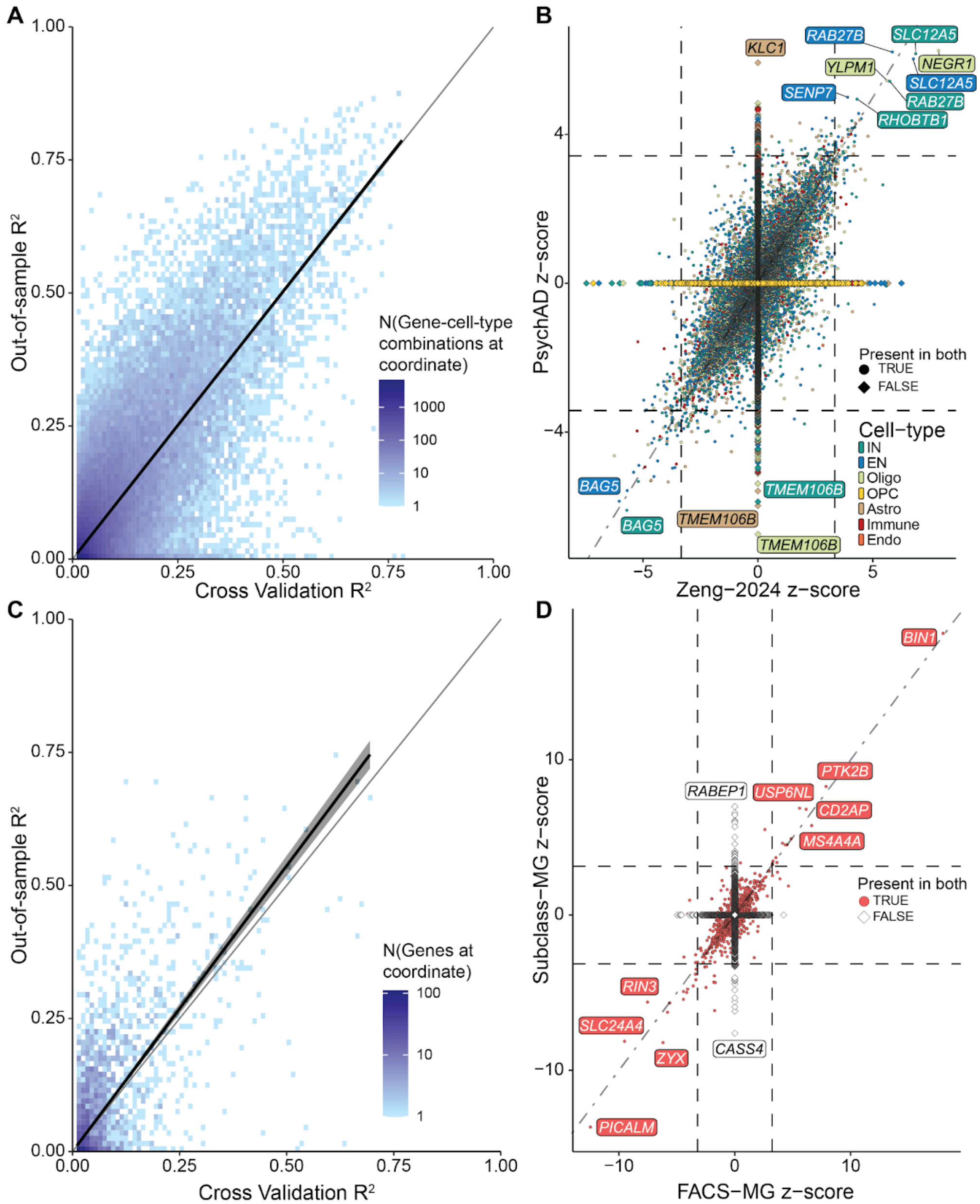

Supplementary Fig. 27 | A, Replication of PsychAD snTIMs in ROSMAP. Binned density (0.01×0.01 bins) of cross-validated R^2_{CV} in PsychAD (x-axis) versus out-of-sample R^2 from Pearson correlations with residualized snRNA-seq expression in ROSMAP (y-axis). Color indicates the number of gene-cell-type models per bin. Gray line: y = x. Black line: y = α × x fit; 95% CI too narrow to visualize. Spearman's ρ = 0.63; n = 70,156; p = $3.91 \times 10^{-7,844}$. **B,** Comparison of major depressive disorder (MDD) S-snTWAS z-scores between Zeng-2024 (x-axis) and PsychAD snTIMs applied to the same GWAS (y-axis). Cell-types were matched

	between studies (Table S37). Genes imputable in both TIMs are marked as points, and genes imputable in only one dataset are marked as diamonds. We observe a Pearson's $r_{(13,760)}$ = 0.78, (p-value = $1.34\times10^{-2,772}$) and a Jaccard similarity index of 0.43. **C,** Replication of the PsychAD Subclass-MG snTIM in FACS-MG. Binned density (0.01 × 0.01 bins) of R^2_{CV} (x-axis) vs. out-of-sample R^2 from residualized snRNA-seq in FACS-MG (y-axis). Gray y = x line; black y = α × x fit with 95% CI (gray band). Spearman's ρ = 0.55; N = 1,853, p-value = 1.67×10^{-149}. **D,** Comparison of AD S-snTWAS z-scores between FACS-MG and PsychAD Subclass-MG applied to the same GWAS. Genes imputable in both TIMs are marked as points, and genes imputable in only one dataset are marked as diamonds. We observe a Pearson's $r_{(733)}$ = 0.87 (p-value = 1.32×10^{-231}) and a Jaccard similarity index of 0.23.

**REF5-2. Out-of-sample validation of non-EUR snTIMs**	
If this experiment were performed, it would also be helpful to see how the models trained in each ancestry-specific data set perform on the cell type specific measured expression of that ancestry's hold out set compared to the other ancestries cell type specific measured expression, rather than comparing the R2cv.	
**Response**	We agree that ancestry-matched out-of-sample testing would be the most direct assessment. At present, external genotype-linked single-nucleus datasets of sufficient scale exist for European ancestry (ROSMAP) and purified microglia RNA-seq, which we used for out-of-sample validation and found close agreement between cross-validated and external performance. Comparable large-scale resources for African and Admixed American donors are not yet publicly available, so we could not perform ancestry-matched external validation for those groups in this study.

**REF5-3. Model selection stringency**	
Could the performance estimates also be inflated by the approach to selecting the best gene models in the snRNAseq? Many more models are constructed here, potentially aggregating the type 1 error. If the authors have mitigated this risk, eg with more stringency on test corrections, it would be helpful to the reader to note these methodological considerations in the sections where the results are presented.	
**Response**	We agree that choosing a "best" model from many candidates can inflate cross-validated performance estimates and increase type I error if not controlled.
**Action**	*Filtering of snTIMs during training.* Imputable models must satisfy both cross-validation FDR at or below 0.05 (more stringent than original submission as per REF1-1) and R^2_{CV} at or above 0.01 within each ancestry, which controls multiplicity from building many gene-cell-type models. This is relevant for the number of imputable genes and the information is stated in the Main Text Methods and shown in Supplementary Fig. 1.<br>*External checks against inflation.* Out-of-sample validation in ROSMAP snRNA-seq and FACS-microglia (REF5-1) shows that out-of-sample R^2 (R^2_{out}) tracks R^2_{CV} closely, supporting

	that cross-validated performance is not inflated by model selection. The figures and text report the concordance and the fitted relationship between R^2_{out} and R^2_{CV}.<br>*Use of "best" models in Fig. 1 is descriptive only.* The "best" per-gene model (max R^2_{CV}) is used for Fig. 1 summaries to illustrate which resolution best predicts a given gene. While the 'best-of' display can be theoretically optimistic, our external calibration (REF5-1) indicates that any bias in R^2_{CV} is negligible in practice, with R^2_{out} essentially matching R^2_{CV} across independent cohorts (ROSMAP and FACS-MG). Even if we wanted to control for multiplicity, there is no universally applicable closed-form adjustment and defining a good null distribution is challenging (REF3-1).<br>*Multiple-testing control for associations.* For snTWAS and downstream summaries, the 'best-of' selection does not affect inference which is performed at the gene-cell-type level. We apply FDR across genes and cell types, and where indicated across traits as well, which avoids inflation from testing many models.

**REF5-4. GWAS sample overlap**	
As nearly all of the trait GWASs considered analyzed UK Biobank data, there is likely substantial overlap in cases and controls in the effect estimates across studies, and this could be a major source of confounding in the cross-disorder investigations. This would bias the genetic correlation analyses. If adjustments were made to account for sample overlap between these studies, it should be noted in the results.	
**Response**	We agree that shared UK Biobank participants across discovery GWAS can inflate cross-disorder comparisons.
**Action**	To mitigate this, our cross-disorder inferences are validated against individual-level TWAS in MVP (I-snTWAS), which uses an independent cohort with no sample overlap with the discovery GWAS used for S-snTWAS; MDD GWAS is the only GWAS with MVP samples and its S-snTWAS is excluded from the analyses. We observe 90% sign concordance (5,162/5,721) between S-snTWAS and MVP I-snTWAS, supporting that our cross-disorder patterns are not driven by shared samples. We have included this analysis in the manuscript.
**Excerpt**	**Supplementary Fig. 14**

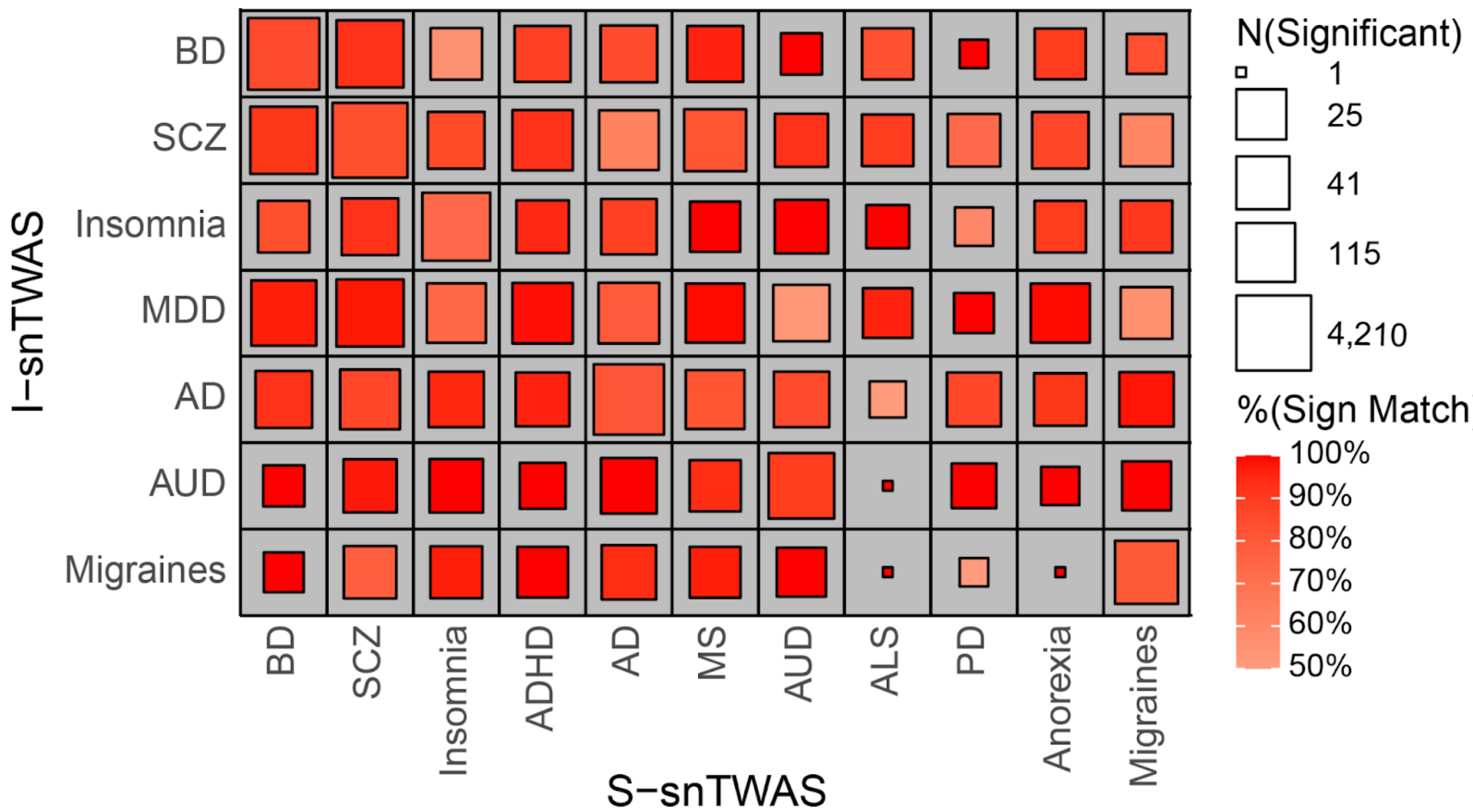

Supplementary Fig. 14 | Cross-disorder sign concordance between S-snTWAS and I-snTWAS. Because several GWAS summary-statistic sets include overlapping participants (e.g., via UK Biobank), we compared S-snTWAS to the MVP-based I-snTWAS with no known sample overlap except for MDD (which was excluded at the S-snTWAS level). Rows list traits analyzed with MVP I-snTWAS; columns list S-snTWAS traits. We intersect the 12 S-snTWAS traits with the 9 sufficiently powered I-snTWAS traits. For each trait pair, we take S-snTWAS-significant gene-cell-type associations (FDR-adjusted p-value ≤ 0.05) and report the fraction with the same effect sign in I-snTWAS. Square size encodes the number of queried associations; color encodes percent sign match; gray cells were not evaluated. Diagonal cells (e.g., BD vs. BD) reflect within-trait agreement. Overall, 90% (5,162 of 5,721) of cross-disorder associations show matching direction in the independent I-snTWAS.

<u>REF5-5. Addressing whether GWASs used contained MVP participants</u>

Can the authors clarify that all of the 12 EUR GWAS used in the S-snTWAS analyses did not include MVP?

**Response**	With the sole exception of the MDD GWAS, none of the other European-ancestry GWAS used in the S-snTWAS analyses included MVP participants.
**Action**	We have explicitly clarified this in the Methods and Results and have excluded MDD from all analyses comparing S-snTWAS with MVP I-snTWAS to ensure that validation results are based on fully independent samples (REF5-4).

**REF5-6. Bulk vs snBulk overlap**	
"Unsurprisingly, across NPD and NDDs, we found a similar number of associated genes in snBulk (2,472) and Bulk (2,287)." With how many genes overlapping?	
**Response & Action**	We agree this overlap is important to report and have added it.
**Excerpt**	Across traits, Bulk and snBulk yielded comparable numbers of associated genes (1,494 vs. 1,254). Among genes imputable by both models, 882 were significant in snBulk and 963 in Bulk, with 529 overlapping (Jaccard = 0.40).

**REF5-7. Specify correlated metrics**	
Minor, but please note explicitly what is correlated when reporting results, e.g. it would be helpful if line 164 notes that z scores were compared.	
**Response**	We thank the reviewer for this helpful suggestion to improve clarity in result reporting.
**Action**	We have revised the text in the Results to explicitly state that z-scores were compared in the relevant correlation analyses and added corresponding clarifications in the Methods.

**REF5-8. Experiment-wide FDR control**	
The FDR correction of the S-snTWAS was calculated across all gene-cell-type combinations for each trait. Some of the excess of findings in these analyses may be explained by inadequate control of type 1 error given the multiple testing; how many results survive an experiment wide FDR correction across all traits? Relatedly, in the section on AD (line 222-237) please clarify what is meant by FDR = 3.76 × 10-62, are these q values? These are not comparatively adjusted, as the authors note that multiple test correction with FDR (≤ 0.05) was performed within each snTIM rather than across relevant snTIMs in these analyses. Having lower power isn't a good justification for less stringent test corrections, and the lack of consistency is potentially misleading to the reader.	
**Response**	We appreciate this important point about multiple-testing rigor and consistent terminology. We have re-analyzed results using experiment-wide FDR control and standardized our terminology to report FDR-adjusted p-values (q values) to ensure consistent, transparent error control.
**Action**	• S-snTWAS (EUR only): We now apply Benjamini-Hochberg FDR across all gene-cell-type-trait tests (experiment-wide). The Results now report how many associations per trait survive this experiment-wide correction, with full counts in the updated Supplementary tables.<br>• I-snTWAS (MVP): We now use FDR across all genes, cell-types, ancestries, and traits (gene-cell-type-trait-ancestry combinations).

	● Pathway enrichment: For each trait, JEPEGMIX2-P results are now corrected across all pathway-cell-type combinations jointly. We explicitly label these as q-values and clarify the correction scope in the text and figure captions.<br>● PheWAS: We now apply FDR across all genes, cell-types, ancestries, and phecodes (phecodes are appropriately filtered for relevance to particular analyses).<br>● snTIM filtering: To define confidently imputable genes, we now apply FDR across all cell-types within an ancestry (retaining the $R^2_{CV} \geq 0.01$ threshold). We do not pool across ancestries for this filtering because downstream analyses are ancestry-stratified and R^2_{CV} is typically more stringent than p_{CV}.<br>● Text edits for clarity: We revised the AD section to state explicitly that the reported "FDR-adjusted p-values" are q-values, and we now specify in all correlation/comparison statements exactly which statistics were compared and the multiple-testing universe used for FDR.

**REF5-9. I^2 confidence intervals**	
95% confidence intervals (CIs) should be included to express the uncertainty associated with I2 estimates.	
**Response**	We agree that reporting confidence intervals improves transparency.
**Action**	We added 95% confidence intervals to all reported I^2 estimates in the text and figures.

**REF5-10. Figure significance labels**	
Very minor- when multiple significance matrices are shown in a single figure, please label them in the figure as well as in the legend (eg extended data 5)	
**Response**	Thank you for the suggestion to improve clarity.
**Action**	We added in-figure labels for all significance matrices and ensured that asterisks and other symbols are defined consistently in the corresponding captions, including Extended Data Fig. 5 (now Extended Data Fig. 3).

References

1. Gamazon, E. R. *et al.* A gene-based association method for mapping traits using reference transcriptome data. *Nature Genetics* **47**, 1091–1098 (2015).
2. Barbeira, A. N. *et al.* Exploring the phenotypic consequences of tissue specific gene expression variation inferred from GWAS summary statistics. *Nature Communications* **9**, 1–20 (2018).

3. Rowland, B. *et al.* Transcriptome-wide association study in UK Biobank Europeans identifies associations with blood cell traits. *Hum Mol Genet* **31**, 2333–2347 (2022).
4. Kosoy, R. *et al.* Alzheimer's disease transcriptional landscape in ex vivo human microglia. *Nat. Neurosci.* (2025) doi:10.1038/s41593-025-02020-2.
5. Lanni, C. *et al.* Zyxin is a novel target for β-amyloid peptide: characterization of its role in Alzheimer's pathogenesis. *J. Neurochem.* **125**, 790–799 (2013).
6. Song, W.-M. & Zhang, B. Multiscale Embedded Gene Co-expression Network Analysis. *PLoS Comput. Biol.* **11**, e1004574 (2015).
7. Fisher, R. A. Frequency distribution of the values of the correlation coeffients in samples from an indefinitely large popu;Ation. *Biometrika* **10**, 507–521 (1915).
8. Zhang, W. *et al.* Integrative transcriptome imputation reveals tissue-specific and shared biological mechanisms mediating susceptibility to complex traits. *Nat. Commun.* **10**, 3834 (2019).
9. Zeng, L. *et al.* A single-nucleus transcriptome-wide association study implicates novel genes in depression pathogenesis. *medRxiv* (2023) doi:10.1101/2023.03.27.23286844.
10. Wray, N. R. *et al.* Genome-wide association analyses identify 44 risk variants and refine the genetic architecture of major depression. *Nat. Genet.* **50**, 668–681 (2018).
11. Gusev, A. *et al.* Integrative approaches for large-scale transcriptome-wide association studies. *Nat. Genet.* **48**, 245–252 (2016).
12. Huckins, L. M. *et al.* Analysis of Genetically Regulated Gene Expression Identifies a Prefrontal PTSD Gene, SNRNP35, Specific to Military Cohorts. *Cell Rep.* **31**, 107716 (2020).
13. Voloudakis, G. *et al.* A translational genomics approach identifies IL10RB as the top candidate gene target for COVID-19 susceptibility. *NPJ Genom Med* **7**, 52 (2022).
14. Wainberg, M. *et al.* Opportunities and challenges for transcriptome-wide association studies. *Nat. Genet.* **51**, 592–599 (2019).
15. Watanabe, K., Taskesen, E., van Bochoven, A. & Posthuma, D. Functional mapping and annotation of genetic associations with FUMA. *Nat. Commun.* **8**, 1826 (2017).

16. de Leeuw, C. A., Mooij, J. M., Heskes, T. & Posthuma, D. MAGMA: generalized gene-set analysis of GWAS data. *PLoS Comput. Biol.* **11**, e1004219 (2015).
17. Yoon, S., Baik, B., Park, T. & Nam, D. Powerful p-value combination methods to detect incomplete association. *Sci. Rep.* **11**, 6980 (2021).
18. Higgins, J. P. T. & Thompson, S. G. Quantifying heterogeneity in a meta-analysis. *Stat. Med.* **21**, 1539–1558 (2002).
19. Machiela, M. J. & Chanock, S. J. LDlink: a web-based application for exploring population-specific haplotype structure and linking correlated alleles of possible functional variants. *Bioinformatics* **31**, 3555–3557 (2015).
20. Fullard, J. F. *et al.* Population-scale cross-disorder atlas of the human prefrontal cortex at single-cell resolution. *Sci. Data* **12**, 954 (2025).
21. Zeng, B. *et al.* Genetic regulation of cell type-specific chromatin accessibility shapes brain disease etiology. *Science* **384**, eadh4265 (2024).
22. Jajoo, A. *et al.* Leveraging genomic and transcriptomic data of diverse ancestry to uncover mechanisms of psychiatric risk in the adult and developing brain. *Research Square* (2025) doi:10.21203/rs.3.rs-5890702/v1.
23. Stegle, O., Parts, L., Piipari, M., Winn, J. & Durbin, R. Using probabilistic estimation of expression residuals (PEER) to obtain increased power and interpretability of gene expression analyses. *Nat. Protoc.* **7**, 500–507 (2012).
24. Burstein, D. *et al.* Genome-wide analysis of a model-derived binge eating disorder phenotype identifies risk loci and implicates iron metabolism. *Nat. Genet.* (2023) doi:10.1038/s41588-023-01464-1.
25. Nievergelt, C. M. *et al.* International meta-analysis of PTSD genome-wide association studies identifies sex- and ancestry-specific genetic risk loci. *Nat. Commun.* **10**, 4558 (2019).
26. Dönertaş, H. M., Fabian, D. K., Valenzuela, M. F., Partridge, L. & Thornton, J. M. Common genetic associations between age-related diseases. *Nat Aging* **1**, 400–412 (2021).

27. International Obsessive Compulsive Disorder Foundation Genetics Collaborative (IOCDF-GC) and OCD Collaborative Genetics Association Studies (OCGAS). Revealing the complex genetic architecture of obsessive-compulsive disorder using meta-analysis. *Mol. Psychiatry* **23**, 1181–1188 (2018).

28. Bellenguez, C. *et al.* New insights into the genetic etiology of Alzheimer's disease and related dementias. *Nat. Genet.* **54**, 412–436 (2022).

29. van Rheenen, W. *et al.* Common and rare variant association analyses in amyotrophic lateral sclerosis identify 15 risk loci with distinct genetic architectures and neuron-specific biology. *Nat. Genet.* **53**, 1636–1648 (2021).

30. Watson, H. J. *et al.* Genome-wide association study identifies eight risk loci and implicates metabo-psychiatric origins for anorexia nervosa. *Nat. Genet.* **51**, 1207–1214 (2019).

31. Demontis, D. *et al.* Genome-wide analyses of ADHD identify 27 risk loci, refine the genetic architecture and implicate several cognitive domains. *Nat. Genet.* **55**, 198–208 (2023).

32. Mullins, N. *et al.* Genome-wide association study of more than 40,000 bipolar disorder cases provides new insights into the underlying biology. *Nat. Genet.* **53**, 817–829 (2021).

33. Jansen, P. R. *et al.* Genome-wide analysis of insomnia in 1,331,010 individuals identifies new risk loci and functional pathways. *Nat. Genet.* **51**, 394–403 (2019).

34. Als, T. D. *et al.* Depression pathophysiology, risk prediction of recurrence and comorbid psychiatric disorders using genome-wide analyses. *Nat. Med.* **29**, 1832–1844 (2023).

35. International Multiple Sclerosis Genetics Consortium. Multiple sclerosis genomic map implicates peripheral immune cells and microglia in susceptibility. *Science* **365**, (2019).

36. Nalls, M. A. *et al.* Identification of novel risk loci, causal insights, and heritable risk for Parkinson's disease: a meta-analysis of genome-wide association studies. *Lancet Neurol.* **18**, 1091–1102 (2019).

37. Trubetskoy, V. *et al.* Mapping genomic loci implicates genes and synaptic biology in schizophrenia. *Nature* **604**, 502–508 (2022).

38. Sanchez-Roige, S. *et al.* Genome-Wide Association Study Meta-Analysis of the Alcohol Use Disorders Identification Test (AUDIT) in Two Population-Based Cohorts. *Am. J. Psychiatry* **176**, 107–118 (2019).
39. Dong, P. *et al.* A multi-regional human brain atlas of chromatin accessibility and gene expression facilitates promoter-isoform resolution genetic fine-mapping. *Nat. Commun.* **15**, 10113 (2024).
40. Siletti, K. *et al.* Transcriptomic diversity of cell types across the adult human brain. *Science* **382**, eadd7046 (2023).
41. Wang, M. *et al.* The Mount Sinai cohort of large-scale genomic, transcriptomic and proteomic data in Alzheimer’s disease. *Sci. Data* **5**, 180185 (2018).
42. Humphrey, J. *et al.* Long-read RNA-seq atlas of novel microglia isoforms elucidates disease-associated genetic regulation of splicing. *medRxiv* (2023) doi:10.1101/2023.12.01.23299073.
43. Kosoy, R. *et al.* Genetics of the human microglia regulome refines Alzheimer’s disease risk loci. *Nat. Genet.* **54**, 1145–1154 (2022).
44. Nott, A. *et al.* Brain cell type-specific enhancer-promoter interactome maps and disease-risk association. *Science* **366**, 1134–1139 (2019).

Response to Reviewers' comments

Table Of Contents:

Referee #1 (Remarks to the Author): 2
Referee #3 (Remarks to the Author): 2
Referee #4 (Remarks to the Author): 2
Referee #5 (Remarks to the Author): 2
Major comments: 3
REF5-A-1. Improved readability of main text and abbreviations 3
REF5-A-2. Sampling covariates and snTIM technical variance 4
REF5-A-3. PEER factor adjustment in snTIM models 9
REF5-A-4. Clarify overall conclusions 10
REF5-A-4a. Cross-ancestry snTIM performance interpretation 10
REF5-A-4b. Gene-to-locus ratio and power considerations 13
REF5-A-4c. snRNA-seq versus bulk RNA-seq conclusions 17
REF5-A-5. Power threshold for S-snTWAS trait inclusion 19
REF5-A-6. PheWAS control definition (0 vs. <2 phecodes) 20
REF5-A-7. Interpretation of overlap metrics in Fig. 4A 22
REF5-A-8. R2CV versus external R2 validation comparison 23
Minor comments: 24
REF5-B-1. Specify ROSMAP ancestry in validation analyses 24
REF5-B-2. General clarity and wording improvements 24
REF5-B-2a. Explicit fine-mapping PIP ≥ 0.5 definition 24
REF5-B-2b. Clarify "region-matched" Bulk S-TWAS phrasing 25
REF5-B-2c. Clarify RHOBTB2-paralytic interaction description 25
REF5-B-2d. Clarify "6,095/31" gene-count comparison 26
REF5-B-2e. Define acronyms such as MSSM, HBCC, RADC, ADSP 26
REF5-B-2f. Move MHC and PTCA method definitions earlier 27
REF5-B-2g. Justify exclusion of the MAPT locus 27
REF5-B-2h. Define high linkage disequilibrium regions 27
REF5-B-2i. Rename ancestry PCs as genetic principal components 28
REF5-B-2j. Detail TOPMed imputation and common SNP thresholds 28
REF5-B-2k. Expand acronyms in figure legends and add table 29
REF5-B-3. Replace "trans-ancestry" with "multi-ancestry" 29
REF5-B-4. Report proportion of replaced SNP genotypes 30
REF5-B-5. Compare imputable gene filters with GTEx and others 30
REF5-B-6. Add table for Fig. 5E and clarify log scaling 31
References 31

Referee #1 (Remarks to the Author):

**REF1.** No further action required	
The authors have done a very good job of taking my comments into account and have made their article more relevant and accessible.	
**Response**	We thank Referee #1 for their positive assessment and are pleased that the revisions made the article more relevant and accessible. We have not introduced additional changes beyond those detailed in our point-by-point responses

Referee #3 (Remarks to the Author):

**REF3.** No further action required	
The authors have adequately addressed my concerns and questions in the revised manuscript	
**Response**	We thank Referee #3 for confirming that our revisions adequately addressed their concerns and questions. We have maintained the clarified text and analyses introduced in the previous round and only made minor wording edits in response to other reviewers.

Referee #4 (Remarks to the Author):

**REF4.** No further action required	
The authors have very adequately processed my suggestions and I commend them on a great manuscript. I have no further concerns or questions.	
**Response**	We are grateful to Referee #4 for their enthusiastic evaluation and for the constructive suggestions that helped improve the manuscript. No further methodological or analytical changes were required in this round beyond the clarifications already incorporated.

Referee #5 (Remarks to the Author):

Summary:

**REF5.** Summary of edits

Venkatesh et al. present a single-nucleus TWAS, using ancestry-specific models they developed from 27 dorsolateral prefrontal cortex cell populations from ~1,500 donors, comprising three ancestry groupings (European, African, and Admixed American). As proof of principle, and to demonstrate advantages of single-nucleus models, they applied these models for GReX association analyses of 12 neuropsychiatric and neurodegenerative traits and identified more than 4,000 gene-trait associations that were not identified in their pseudo-bulk-tissue models. This paper is presenting a tremendous amount of work, which will be of high interest to many readers working in complex trait genetics. My main critique is that the paper is so dense, with references to extended/supplementary material, that it disrupts the flow and makes it somewhat difficult to read. I also have some minor concerns about some of the interpretations made.

**Response**	We thank Referee #5 for their thorough and constructive review and for highlighting both the strengths and the density of the manuscript. In response, we have streamlined the Main text, reduced reliance on parenthetical references to Extended Data and Supplementary material, centralized definitions of acronyms and technical terms, and clarified or tempered several interpretational statements, as detailed in our point-by-point responses to comments REF5-A-1 through REF5-B-6.

Major comments:

**<u>REF5-A-1.</u>** Improved readability of main text and abbreviations
To improve the readability of the Main section, could the authors move some of the references to extended material into the Methods sections, or write some out in the text? An example of the latter suggestion, for: “Using genotype and snRNA-seq data from the PsychAD Consortium, we trained 94 snTIMs across three hierarchical levels of cell-type resolution, encompassing 32 cellular populations (27 non-overlapping across 4 classes and 23 subclasses) in the DLPFC (Extended Data Fig. 2; Table S1).”<br><br>Possible edit: “Using genotype and snRNA-seq data from the PsychAD Consortium, we trained 94 snTIMs across three hierarchical levels of cell-type resolution, encompassing 32 cellular populations (27 non-overlapping across 4 classes and 23 subclasses) in the DLPFC. Cellular populations and class groupings are shown in Extended Data Fig. 2, and Table S1 gives the abbreviations for each cellular population used throughout.”<br><br>Additionally, there are many acronyms and abbreviations throughout the paper, and it would be helpful to have a central reference that readers can refer to.

**Response**	We thank the reviewer for this helpful suggestion. We understand the concern that (i) the Main text relied heavily on parenthetical references to Extended Data figures and tables

	without briefly indicating what they contain, and (ii) acronyms were numerous and lacked a single, central place for readers to look them up. We agree that addressing both issues improves readability and navigation. In revising the manuscript, we adopted a compromise that adds short descriptive phrases and centralized references while keeping the Main text within space constraints.
**Action**	To address this, we implemented several changes.<br>1. We revised the Main text passages that previously only cited Extended Data material so that they now briefly indicate the content of the referenced figure or table, while moving detailed methodological descriptions and implementation specifics to the Methods and Supplementary Results.<br>2. We reduced the use of abbreviations in the Main text by spelling out rarely used terms and ensuring that all remaining abbreviations are defined when they first appear.<br>3. We created two centralized supplementary resources: one table (Table S1) providing the expansion of every acronym used in the work, and a second table (Table S2) listing every figure (main, Extended Data, and Supplementary) with a brief plain-language description. These tables give readers a single reference point for navigating both figures and abbreviations.<br>4. In the specific example highlighted by the reviewer, the Main text now reads: "Using quality-controlled (see Methods) genotype and snRNA-seq data from the PsychAD Consortium[1,2], we trained 94 snTIMs across three ancestries and 32 cellular populations in the DLPFC (Extended Data Fig. 2; Table S3)." Extended Data Fig. 2 and Table S3 together now provide a clear graphical overview of the cell-type hierarchy and a central reference for all cell-type abbreviations, while the new tables described above serve as global guides to figures and acronyms across the manuscript.

**<u>REF5-A-2.</u>** Sampling covariates and snTIM technical variance	
Please comment on variables relating to the sampling that could impact variation in transcript measures, such as time from death to transcript preservation, and how these variables could be affecting the model, and justify this choice in the text.	
**Response**	We thank the reviewer for raising this important point about sampling-related variables such as postmortem interval (**PMI**) and tissue handling, and how they could affect transcript measures and our transcriptomic imputation models.<br><br>The PsychAD cohort and the single-nucleus RNA sequencing (**snRNA-seq**) dataset are fully described in our companion PsychAD Capstone Manuscript (Nature, In Press, see preprint in [3]. Briefly, we performed a variance partition analysis of stacked pseudobulk gene expression that included cell-type, donor, tissue source, technical replicate, age, sex, ancestry, diagnosis, PMI, and technical metrics related to sequencing depth and mitochondrial or ribosomal content as covariates. As summarized in Fig. R1 below (reproduced from that

	manuscript), cell-type explains about 50% of the total variance in gene expression. Interindividual variation explains roughly 10%, whereas sampling variables such as PMI, tissue source, technical variables (such as sequencing depth) each explain less than 1% of expression variance on average, with the remainder attributed to residual variation.<br><br>When performing the variance partition within each cell-type, mitochondrial measures explain about 5.4% of the total variance (Fig. R2). These findings are consistent with prior work in single-cell and snRNA-seq identifying mitochondrial RNA fraction (and related measures) as one of the strongest and most reproducible readouts of such pre-analytical effects: damaged or dying cells, or cells from tissue that experienced prolonged warm ischemia, lose cytosolic RNA while retaining mitochondrial transcripts, leading to an elevated mitochondrial proportion that is widely used as a marker of low-quality libraries[4–8]. Finally, PMI, tissue source, and technical variables such as sequencing depth still explain less than 1% of the variance on average, even when accounting for all other covariates used to derive expression residuals.<br><br>Importantly, motivated by these findings, our PsychAD transcriptomic imputation model (**TIM**) pipeline trains models on gene expression that has been residualized for the identified covariates, as well as for the sample pool and disease status. Prior to model training, residualized pseudobulked gene expression for each cell-type and subcohort (Mount Sinai National Institute of Health Neurobiobank (**MSSM**), National Institute of Mental Health Intramural research program Human Brain Collection Core (**HBCC**), Rush Alzheimer's Disease Center (**RADC**)) was transformed into Pearson residuals (i.e., residuals divided by their standard errors) to control for the impact of varying sequencing depth. Finally, we perform a two-step probabilistic estimation of expression residuals (**PEER**) correction to account for the effects of hidden factors[9]. We provide greater details on this process in the subsequent comment.<br><br>To optimize these selections, we benchmarked the power to detect a higher number of expression quantitative trait loci (**eQTL**s) as also described in the companion manuscript (Nature Genetics, Under Revision, see preprint in [10]. For example, using the Pearson residuals identified 3.9 to 10.5% more genome-wide significant eGenes (genes that are significantly associated with at least one genetic variant) than using raw residuals (Fig. R3).

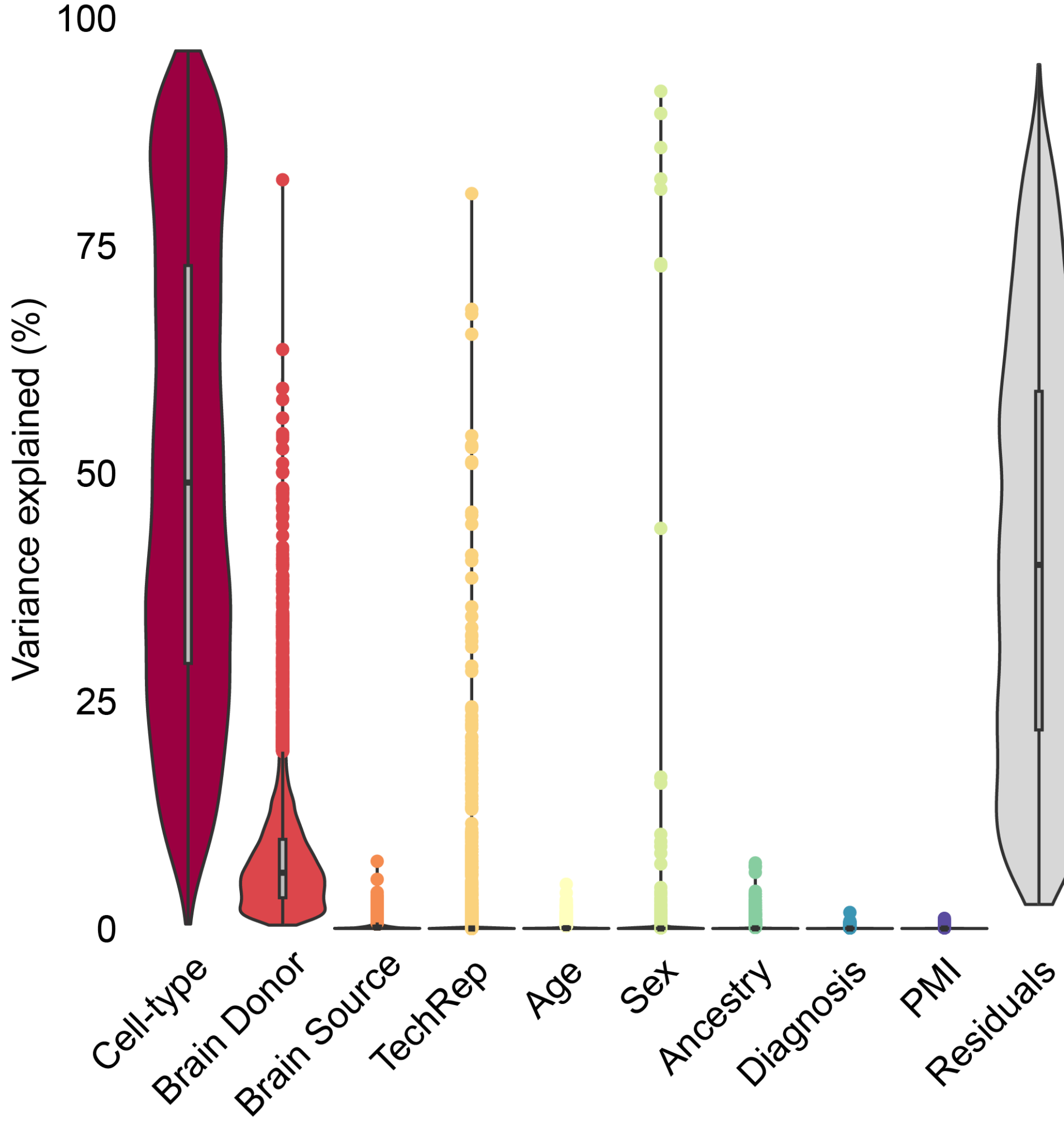

Fig. R1 | Variance partition of the transcriptome in stacked pseudobulk. Partition of transcriptomic variation by technical and clinical covariates. n=34,890 genes examined over 2,924 libraries with 1,494 donors across 27 subclasses. n=9,256 protein-coding genes visualized. Standard box plots are displayed, where the box indicates the first and third quartiles, the center line represents the median, and the whiskers extend to the minimum and maximum values. “Brain Source” refers to the three subcohorts (Mount Sinai National Institute of Health Neurobiobank (**MSSM**), National Institute of Mental Health Intramural research program Human Brain Collection Core (**HBCC**), Rush Alzheimer’s Disease Center (**RADC**)). “TechRep” refers to snRNA-seq technical replicates. “PMI” stands for post-mortem interval.

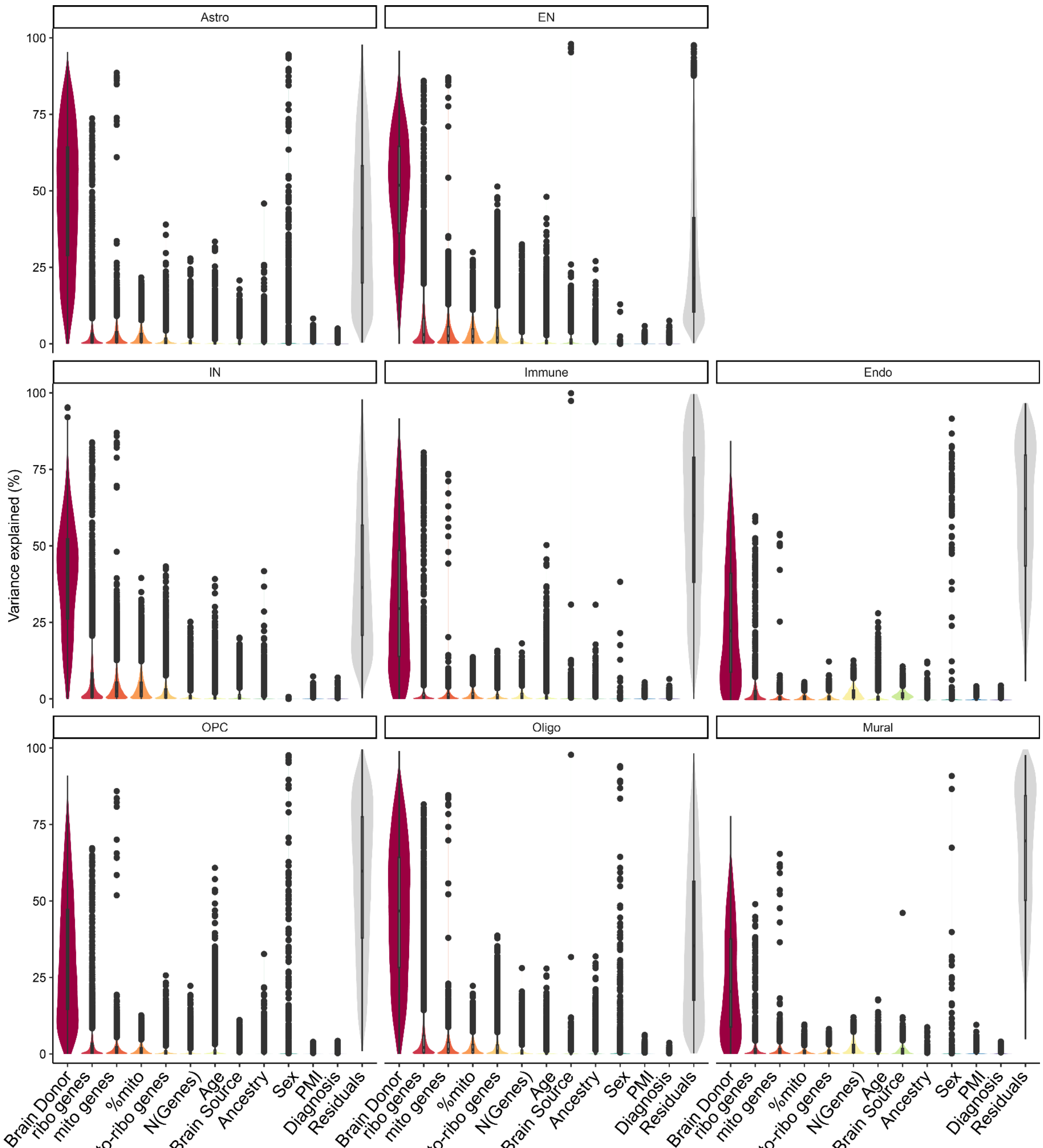

Fig. R2 | Variance partition of the transcriptome at the class level. Partition of transcriptomic variation by technical and clinical covariates. n=34,890 genes examined over 2,924 libraries with 1,494 donors across 8 classes. After filtering under-represented and low variance genes, n=24,552 genes were retained for variance partition analysis, and n=15,271 protein-coding genes were used for visualization. Standard box plots are displayed, where the box indicates the first and third quartiles, the center line represents the median, and the whiskers extend to the minimum and maximum values. “Ribo genes”, “mito genes”, and “mito-ribo genes” refer to the enrichment of ribosomal genes, mitochondrial genes, and

	non-chromosome M mitochondrial ribosomal genes. “%mito” refers to the percent of mitochondrial genes to all genes. “Brain Source” refers to the three subcohorts (Mount Sinai National Institute of Health Neurobiobank (**MSSM**), National Institute of Mental Health Intramural research program Human Brain Collection Core (**HBCC**), Rush Alzheimer’s Disease Center (**RADC**)) . “TechRep” refers to snRNA-seq technical replicates. “PMI” stands for post-mortem interval.<br>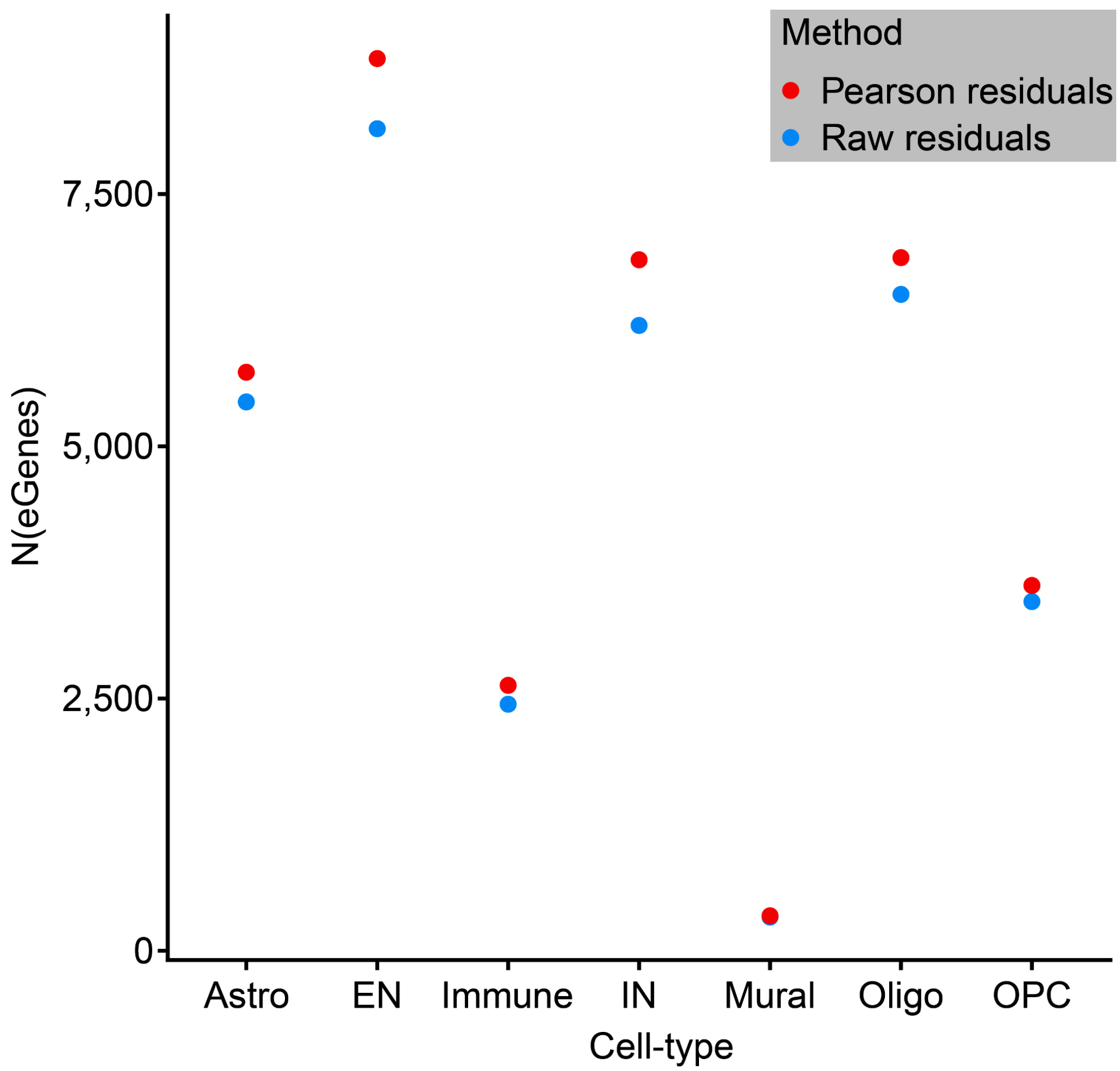<br><br>**Fig. R3 | Comparison of normalization strategies for expression quantitative trait loci (eQTL) detection across cell-types.** We compare the number of genome-wide significant eGenes (genes that are significantly associated with at least one genetic variant) detected using Pearson residuals (as in the main analysis) to those detected using raw residuals after accounting for covariates from a precision-weighted regression model. For each method, eQTL analysis is performed independently in eight cell classes using an identical statistical framework and significance threshold. The number of genome-wide significant eGenes detected per cell class is shown for each normalization strategy.
**Action**	1. We added a new paragraph to the methods section titled “Genotype and gene expression quality control for TIM training” underneath the subheader “Gene expression quality control”.

	2. We also added a clarifying statement to the main text and a reference to the methods section to clear this up.
**Excerpt**	"As reported in Lee et al. and Zeng et al., dreamlet was used to create pseudobulked gene expression by summing reads from the same individual[1,10]. Expression for each cell type and each individual was computed as the log2 counts per million (CPM) with a pseudocount of 0.25 after aggregating all corresponding nuclei. A precision-weighted regression model was fit for each expressed gene in each cell type using the dreamlet package[11] using covariates for age, sex, postmortem interval, mitochondrial rate, ribosomal rate, and disease status. To control for varying sequencing depth amongst snRNA-seq libraries, Pearson residuals (i.e., residuals divided by their standard errors) were computed for each expressed gene and cell type and used in downstream analysis."<br><br>"Using quality-controlled (see Methods) genotype and snRNA-seq data from the PsychAD Consortium[1,2] …"

**<u>REF5-A-3.</u>** PEER factor adjustment in snTIM models	
Was there any adjustment for PEER factors in the model construction process?	
**Response**	Yes, we performed a two-step PEER factor correction to the pseudobulked gene expression to adjust both for hidden covariates within each cohort and between cohorts. For each cell-type and subcohort (MSSM, HBCC, RADC), we calculated PEER factors and performed optimization to choose the ideal number of PEER factors between 5 and 50 (optimum signal was designated to be at 95% of the maximum number of significant (false discovery rate (**FDR**)-adjusted p-value ≤ 0.05) eQTLs as calculated by fastQTL). We then performed PEER a second time for each cell-type after integrating the three subcohorts together, and performed an identical PEER factor optimization.
**Action**	We have added a clarifying statement to the main text and a reference to the methods section to clear this up.
**Excerpt**	"Using quality-controlled (see Methods) genotype and snRNA-seq data from the PsychAD Consortium[1,2] …"<br><br>"We limited genes to those within our expression annotation, Ensembl 104[12,13]. Second, for each "subcohort" within PsychAD (see "PsychAD Cohort"), we independently scaled gene expression, performed probabilistic estimation of expression residuals (**PEER**)[9] to identify and adjust for hidden factors driving gene expression differences, and quantile-normalized the resulting residualized gene expression. We utilized fastQTL[14] to determine the number of PEER factors (ranging from 5 to 50) that yield optimal genetic signal by identifying the point at which the number of significant expression quantitative trait loci (**eQTL**s; false discovery rate (**FDR**; Benjamini-Hochberg method)-adjusted p-value[15] ≤ 0.05) is closest to 95% of the maximum number of significant eQTLs found across any assessed number of PEER factors.

	Finally, we combined residualized gene expression across the 3 "subcohorts", and repeated scaling, PEER factor optimization, and quantile-normalization of the combined cohort to reduce the impact of the batch effect (Supplementary Fig. 30)."

**REF5-A-4.** Clarify overall conclusions	
Some interpretations seem either a bit overstated or to not fully capture the nuance of the data, e.g.:	
**Response**	We thank the reviewer for helping us communicate our work appropriately.
**Action**	We have addressed individual points in the comments below.

**REF5-A-4a.** Cross-ancestry snTIM performance interpretation	
In "Brain snTIMs capture brain GReX with cell-type-specificity across ancestries", the statement: "Despite differences in training sample size, shared gene-cell-type models showed strong correlations in R2CV…suggesting that genetic regulation of expression is broadly consistent across ancestries."<br><br>I have two concerns, the first being that genes are not independent measures, so unless the data was filtered to remove genes with correlated expression first, the correlation values will be inflated. The second is that the stats presented are showing that the model quality is similar across ancestries, but not that the models themselves are similar.<br><br>I have similar concerns about the statement: "However, here we demonstrate that ancestry-matched experimental designs are very effective for cross-ancestry NPD/NDD studies even with limited sample sizes, paralleling prior diverse ancestry blood monocyte TWASs."<br><br>The "ancestry-matched experimental designs are very effective for cross-ancestry NPD/NDD studies" part is a bit vague, it would be helpful to clarify some and this might address my concerns. Also, though, given that what was presented doesn't clarify the similarity of the models themselves, just their performance, it's not clear to me that the data that's presented effectively supports this statement.	
**Response & Action**	We thank the reviewer for their thoughtful comments on our cross-ancestry snTIM comparison.<br><br>First, we agree that genes are not independent observations and that this can inflate correlations if not handled carefully. Our goal was to compare the relative performance of models across ancestries rather than to interpret the absolute magnitude of the correlation.

	To reduce sensitivity to non-independence and outliers, we used Spearman's correlation of R^2_{CV} values instead of Pearson's correlation. In addition, we repeated the analysis after thinning to one random overlapping imputable gene per linkage disequilibrium block (all cell-types, 10 iterations per cell-type). The median Spearman's correlation across iterations (0.52) was very similar to that reported in Fig. 1C (0.54), indicating that the observed cross-ancestry pattern is not driven solely by clusters of correlated genes. We now describe this LD-thinned sensitivity analysis in the Supplementary Notes (Supplementary Note 5).<br><br>Second, we agree that the statistics presented speak to model quality and to the extent to which gene expression variance is genetically regulated across ancestries rather than to the similarity of the underlying single nucleotide polymorphism (**SNP**) weight vectors. To make this distinction explicit, we quantified the overlap of SNP predictors for the same gene-cell-type combination across ancestries and found low concordance (median Jaccard index = 0.019). We have revised the text to avoid implying that the models themselves are similar and instead emphasize that the performance profiles are similar despite different SNP sets.<br><br>Thirdly, we demonstrate the reduction in cross-ancestry concordance of single-nucleus transcriptome-wide association studies (**snTWAS**) when using ancestry-mismatched snTIMs and GWAS. We applied EUR snTIMs to AFR variant association results to create an ancestry-mismatched individual-level snTWAS (**I-snTWAS**). We correlated the results with the EUR I-snTWAS results and contrasted this correlation with the traditional EUR vs. AFR I-snTWAS approach. Progressive thresholding correlation analysis (**PTCA**) shows a reduction in correlation amongst top gene-trait associations (Fig. R4). These results indicate that mismatches in ancestry when applying snTIMs to GWAS may lead to misidentified biological signals. These conclusions are supported by other recent TWAS literature on bulk brain tissue [16].<br><br>Finally, we have softened and clarified the more general statement about experimental design to tie it directly to what we actually show (model performance and cross-ancestry snTWAS yield) rather than to unmeasured model similarity.

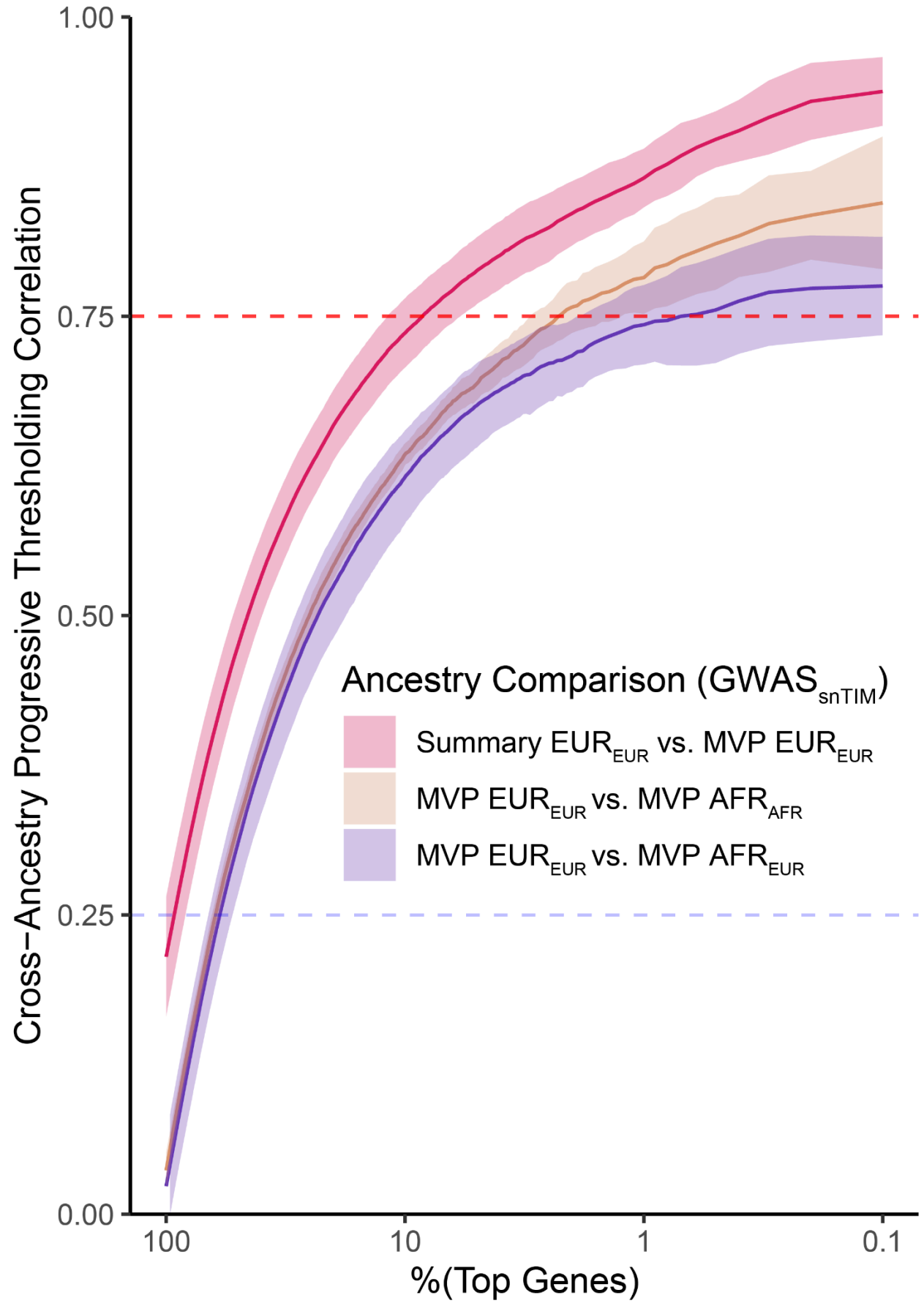

Fig. R4 | Cross-ancestry progressive thresholding correlation analysis (PTCA) of single-nucleus transcriptome-wide association studies (snTWAS). The y-axis shows the Pearson's correlation of TWAS z-scores between two designs as a function of the top-ranked gene-trait associations retained on the x-axis (from 100 percent down to 0.1 percent of genes). Each snTWAS design is denoted as $GWAS_{snTIM}$ to indicate the ancestry of the genome-wide association study (**GWAS**) source and the single-nucleus transcriptomic imputation model (**snTIM**) reference panel. The purple line shows the mean correlation between an ancestry-matched European (**EUR**) individual-level snTWAS (**I-snTWAS**) (MVP EUR_{EUR}) and an ancestry-mismatched I-snTWAS in which EUR snTIMs are applied to African (**AFR**) GWAS results (MVP AFR_{EUR}). The peach line shows the mean correlation between standard cross-ancestry I-snTWAS designs, comparing MVP EUR_{EUR} with MVP AFR_{AFR}. The red line shows the mean correlation between EUR summary-level snTWAS (**S-snTWAS**) (Summary EUR_{EUR}) and EUR individual-level snTWAS (MVP EUR_{EUR}). All curves represent the mean across 6 traits analyzed in both the I-snTWAS and S-snTWAS frameworks (excluding major depressive disorder due to sample overlap), and shaded areas indicate 95% confidence intervals. Red and blue dashed horizontal lines indicate the reference Pearson's correlations of r = 0.75 and r = 0.25, respectively.

**Excerpt**	"Despite differences in training sample size, shared gene-cell-type models showed strong correlations in R^2_{CV} ..., suggesting that genetic regulation of expression is broadly consistent

	across ancestries, even when the contributing single nucleotide polymorphisms (**SNP**s) are different (Supplementary Notes 3 and 4)."<br><br>"Together with the cross-ancestry snTWAS results, these findings indicate that training snTIMs in ancestry-matched reference panels can provide effective models for multi-ancestry NPD/NDD studies, provided that each ancestry has sufficient sample size for model building."<br><br>"While most prior work has emphasized the poor portability of EUR-trained models to other ancestries[17,18], our results show that ancestry-matched designs can be highly informative for cross-ancestry NPD and NDD studies even with modest sample sizes, paralleling prior diverse ancestry blood monocyte TWASs[19–21]."

**<u>REF5-A-4b.</u>** Gene-to-locus ratio and power considerations	
In "Brain snTWAS increases power for both known and novel gene-trait association discovery", the statement: "Higher cell-type resolution increased the gene-to-locus ratio, indicating that distinct cellular populations may contribute uniquely within shared loci and warrant further functional study." This is one possible explanation, but there are other possible explanations, especially that differences in power or reduced control for multiple testing, given the increase in number of test sin the cell-type-specific models, could explain this. It would be helpful to acknowledge this possibility here, and throughout the paper.	
**Response & Action**	We thank the reviewer for raising this important caveat about our interpretation of the gene-to-locus ratio. We note first that all snTIM analyses (EUR single-nucleus-derived pseudobulk model ("**snBulk**"; all nuclei pooled), cellular classes ("**Class**"), cellular subclasses ("**Subclass**")) use a single multiple-testing correction across all cell-types, genes and traits, so finer cell-type resolution does not benefit from a more permissive threshold. To directly assess whether differences in multiple testing or power could explain the observed pattern, we repeated the full analysis under a stringent Bonferroni correction across all cell-types, genes and traits, followed by fine-mapping, and summarized the results in new Supplementary Figs R5-R8. Under both FDR and Bonferroni correction, we see the same qualitative pattern: the number of significant genes and fine-mapped genes increases from snBulk to Class to Subclass, the number of fine-mapped loci is comparable or higher, and the average number of fine-mapped genes per locus also increases. This indicates that the higher gene-to-locus ratio at finer cell-type resolution is not simply an artifact of weaker multiple-testing control or of inflating discoveries through more tests.<br><br>At the same time, we agree that differences in power between models (for example, differences in cell counts across cell-types) and model properties could contribute to the observed pattern. We have therefore softened and clarified the relevant text in the Results and Discussion to state that higher cell-type resolution is consistent with distinct cellular

populations contributing unique gene-trait associations within shared loci, while explicitly acknowledging that residual differences in power and model architecture may also play a role.

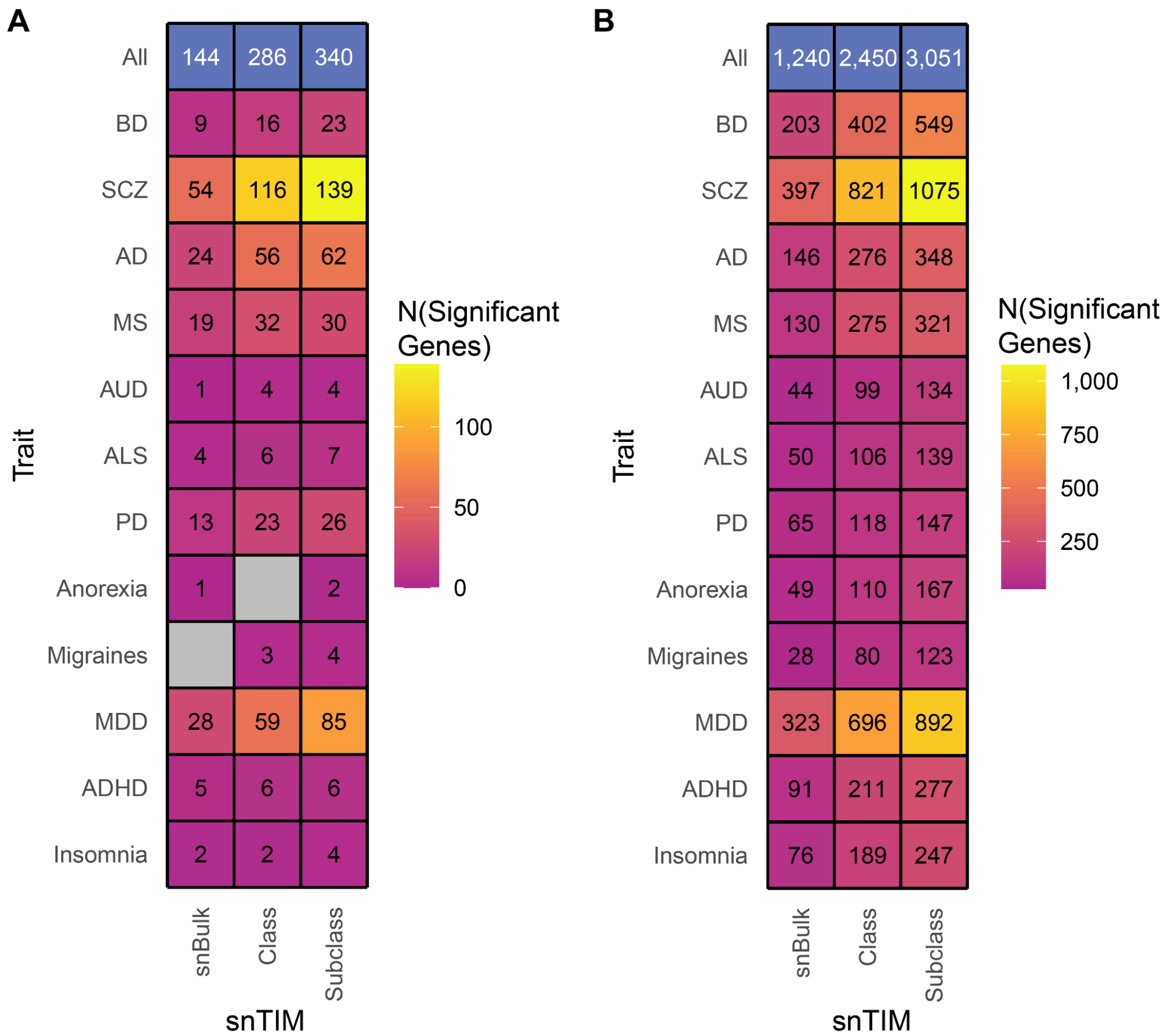

Fig. R5 | Number of significant genes. Heatmap of significant gene-trait associations (**GTA**s) by model resolution (snBulk, Class, Subclass). Numbers in cells and square size represent significant GTA counts under **(A)** Bonferroni adjustment and **(B)** false discovery rate (**FDR**) adjustment. All p-value adjustments are performed across all traits and single-nucleus transcriptomic imputation models (**snTIM**s) for parity). "All" refers to the union of all traits at the snTIM level. Cell color denotes the number of significant genes (ignoring the "All" category, for scaling).

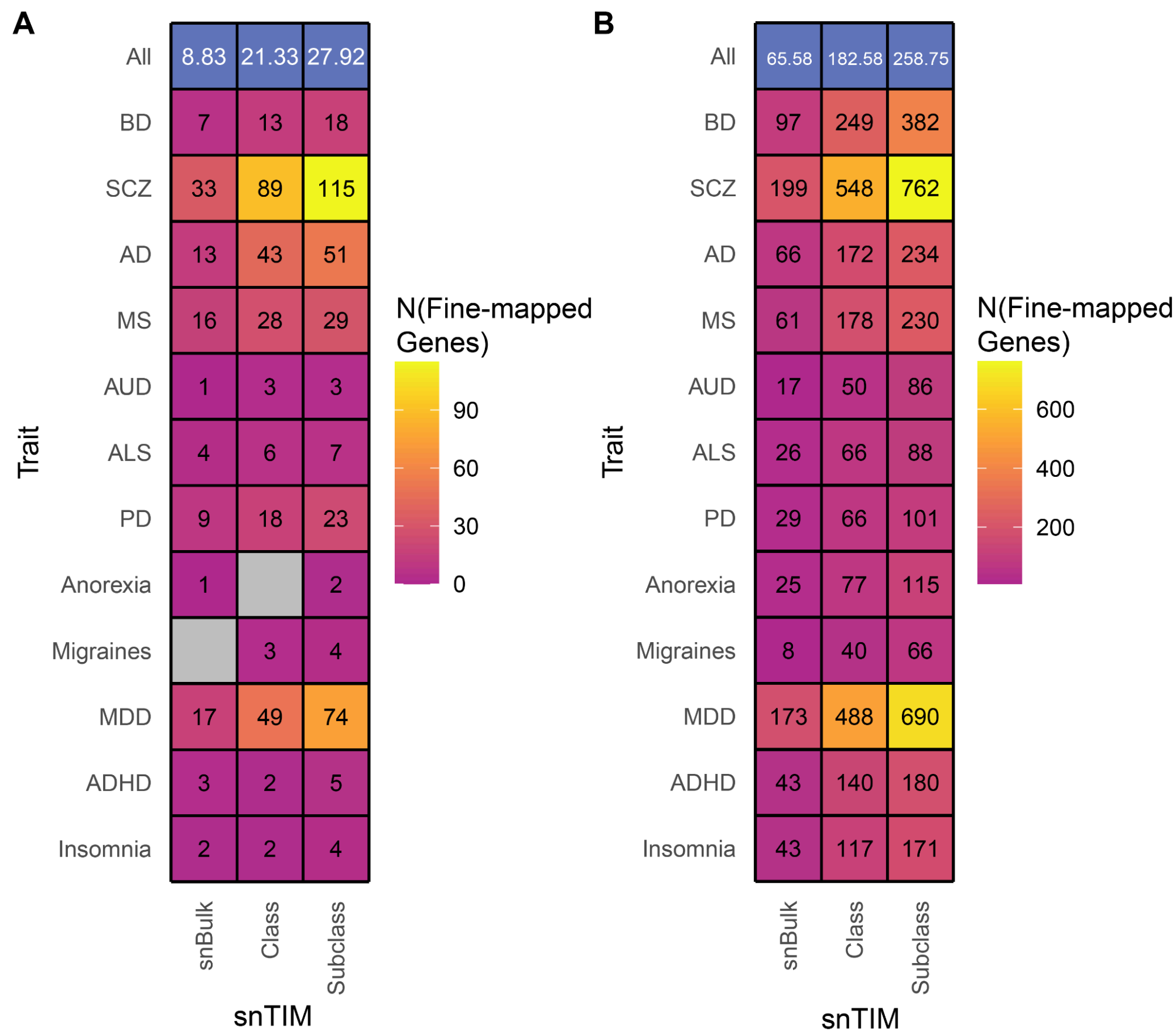

Fig. R6 | Number of fine-mapped genes. Heatmap of fine-mapped gene-trait associations (**GTA**s) by model resolution (snBulk, Class, Subclass). Numbers in cells and square size represent fine-mapped GTA counts under **(A)** Bonferroni adjustment and **(B)** false discovery rate (**FDR)** adjustment. All p-value adjustments are performed across all traits and single-nucleus transcriptomic imputation models (**snTIM**s) for parity). “All” refers to the average of all traits at the snTIM level. Cell color denotes the number of fine-mapped genes (ignoring the “All” category, for scaling).

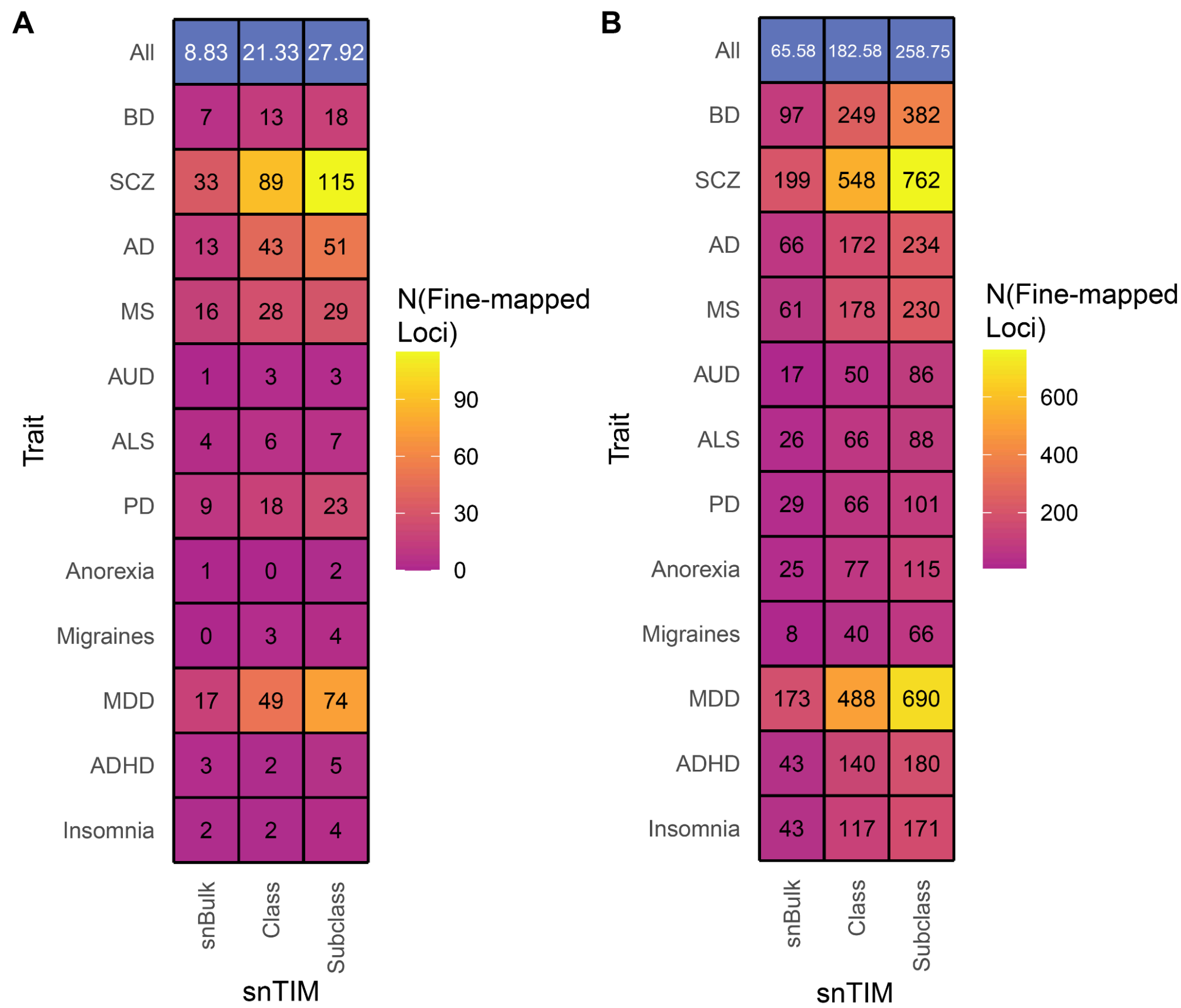

Fig. R7 | Number of fine-mapped loci. Heatmap of fine-mapped loci by model resolution (snBulk, Class, Subclass). Numbers in cells and square size represent fine-mapped loci counts under **(A)** Bonferroni adjustment and **(B)** false discovery rate (**FDR)** adjustment. All p-value adjustments are performed across all traits and single-nucleus transcriptomic imputation models (**snTIM**s) for parity). “All” refers to the average of all traits at the snTIM level. Cell color denotes the number of fine-mapped loci (ignoring the “All” category, for scaling).

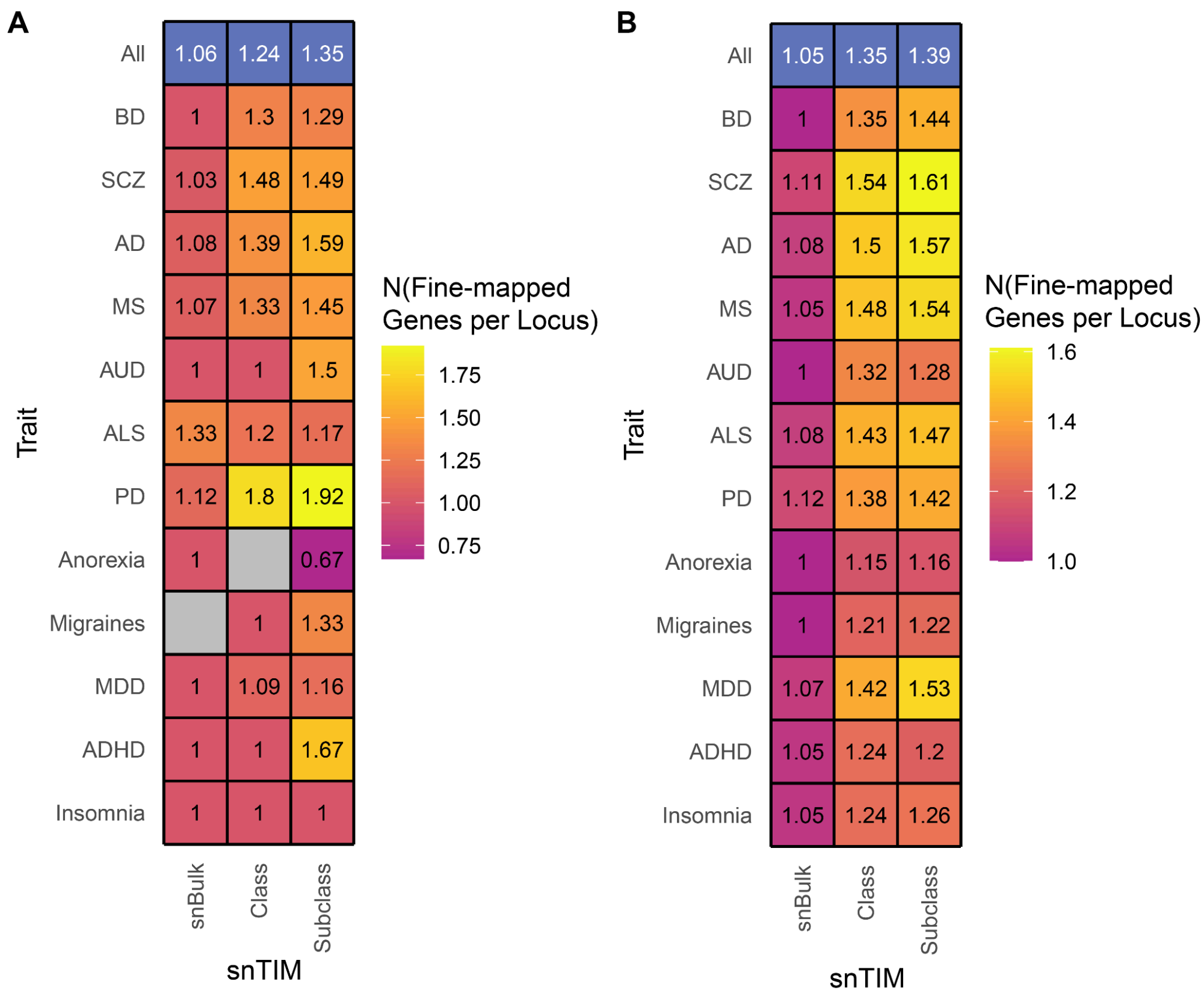

Fig. R8 | Number of fine-mapped genes per locus. Heatmap of fine-mapped gene-trait associations (**GTA**s) per locus by model resolution (snBulk, Class, Subclass). Numbers in cells and square size represent fine-mapped GTA per locus under **(A)** Bonferroni adjustment and **(B)** false discovery rate (**FDR**) adjustment. All p-value adjustments are performed across all traits and single-nucleus transcriptomic imputation models (**snTIM**s) for parity. “All” refers to the average of all traits at the snTIM level. Cell color denotes the number of fine-mapped genes per locus (ignoring the “All” category, for scaling).

REF5-A-4c. snRNA-seq versus bulk RNA-seq conclusions

Similarly, in the Discussion, the statement: “Compared with bulk approaches, there seems to be no downside at the gene level in transitioning to snRNA-seq beyond cost and instances where capturing extranuclear RNA might prove desirable.”

I have a few concerns, including the same concern as above, that the difference in multiple testing control given the increased number of tests is not acknowledged here. Also, the study does not make the comparison that would be most helpful in clarifying this point, which would be to perform actual bulk RNA-seq, rather than the pseudo-bulk (which is a helpful comparison point as well, but not quite the same), in the same study participants and to compare not only the number of genes and genes per locus identified, but also the actual genes themselves, and their abundance and

predictive models. If the authors could show that the snRNA-seq approach identifies all of the genes identified by the bulk approach, this would better support the point they're making here. This concern could also be addressed just by changing the language a bit, perhaps to "our study did not find downsides at the gene level…".	
**Response**	We thank the reviewer for their careful comments on our comparison between snRNA-seq and bulk RNA-seq based models.<br><br>**Multiple testing correction**<br>First, we agree that differences in multiple testing could, in principle, bias comparisons between bulk and single-nucleus approaches. To mitigate this, we applied comparable FDR-based criteria wherever possible. For model training, we used FDR on cross-validation p-values to define confidently imputable genes for the DLPFC EpiXcan TIM ("**Bulk**"). For PsychAD snTIMs, we applied FDR jointly across all snTIMs within each ancestry. For the S-snTWAS comparison in Fig. 2A, we further controlled FDR across Bulk S-TWAS and PsychAD S-snTWAS together so that significant gene-trait associations are called under a single joint correction. Thus, the observed differences are not driven by using a more permissive threshold for cell-type models.<br><br>**Bulk versus single-nucleus comparison**<br>Second, we agree that the ideal experiment to evaluate downsides of snRNA-seq would be a fully matched bulk RNA-seq and snRNA-seq design in the same individuals. This is not possible with the current datasets. We do not have bulk RNA-seq for all PsychAD participants and, even if we were to train models only on the 311 donors shared between our Bulk TIM and the EUR PsychAD snTIMs (33.7% of the Bulk cohort), differences in dissection, sample preparation and other technical parameters would still prevent a clean comparison where the only difference is library preparation and downstream bioinformatics.<br><br>However, we did leverage the partially matched comparison between Bulk and EUR snBulk, which have similar total donor counts (924 vs. 920), matched ancestry and the same brain region (DLPFC). In this setting, Bulk and snBulk impute a comparable number of genes (8,959 vs. 9,147), with substantial but incomplete overlap (6,102 shared), and they yield similar numbers of S-snTWAS significant gene-trait associations, with 529 overlapping among genes imputable by both models. We also found that genes uniquely imputable in Bulk or snBulk are enriched for long non-coding RNAs (**lncRNA**s), consistent with known technological differences between RNA-seq and snRNA-seq, and we explicitly acknowledge that snRNA-seq cannot capture extranuclear RNA.<br><br>Taken together, these results support a more modest statement: within this partially matched DLPFC EUR comparison, we do not observe a systematic loss of gene-level signal when moving from homogenate bulk RNA-seq to snRNA-seq derived snBulk, while recognizing that snRNA-seq has clear technological limitations (extranuclear RNA, isoforms) and higher cost. A definitive, fully powered head-to-head comparison would require bulk RNA-seq and

	snRNA-seq generated in the same individuals, which is beyond the scope of the present study.
**Action**	We have revised the Discussion to (i) make explicit that multiple testing is controlled jointly across Bulk and PsychAD S-snTWAS when comparing discovery yield, (ii) highlight the partial sample overlap between Bulk and EUR snBulk and the quantitative similarities and differences in imputable genes and S-snTWAS results, and (iii) incorporate the reviewer's suggested wording by softening the original claim to state that our analyses did not identify clear gene-level disadvantages of snRNA-seq relative to bulk in this setting, while explicitly noting the technological limitations of snRNA-seq and the absence of a fully matched bulk-versus-single-nucleus experiment.
**Excerpt**	"The EUR single-nucleus-derived pseudobulk model ("**snBulk**"; all nuclei pooled) and a similarly sized EUR homogenate-based model from the same region ("**Bulk**"; DLPFC EpiXcan TIM[22]; N = 924; 311 individuals in common with EUR snBulk) imputed a comparable number of genes (9,147 vs. 8,959) with substantial but incomplete overlap (6,102 shared; 66.7%−68.1%; Fig. 1A; Supplementary Note 1)."<br><br>"Across traits, Bulk and snBulk yielded comparable numbers of significant GTAs (1,494 vs. 1,254). Among genes imputable by both models, 882 were significant in snBulk and 963 in Bulk, with 529 overlapping (Jaccard = 0.40)."<br><br>"To determine whether the greater number of GTAs identified by cell-type-specific TWAS is clinically relevant, we performed gene set enrichment analysis for genes known to be associated with central nervous system-related neurological and behavioral/psychiatric symptoms in the Online Mendelian Inheritance in Man database[23]. Bulk and snBulk analyses revealed significant enrichment for genes linked to 5 and 4 of the 12 NPDs and NDDs, respectively …"<br><br>"Compared to bulk approaches at the gene level, we did not observe obvious downsides to moving to snRNA-seq, aside from cost and specific scenarios where capturing extranuclear RNA is necessary."

**REF5-A-5.** Power threshold for S-snTWAS trait inclusion	
It is surprising to me that several of the original 16 traits were considered underpowered for S-snTWAS (binge-eating disorder, post-traumatic stress disorder, anxiety, and obsessive-compulsive disorder). The sample sizes are similar to the GWAS that were included. Do the authors have a proposed explanation for why they were insufficiently powered?	
**Response**	We thank the reviewer for their interest in our trait selection. While the sample sizes and SNP-heritability estimates for binge-eating disorder, post-traumatic stress

	disorder, anxiety, and obsessive-compulsive disorder are similar to those of several traits we retained, these GWASs yielded very few genome-wide significant loci (0 for all four traits except binge-eating disorder, which had 2), whereas the traits included in our S-snTWAS analyses had at least 9 genome-wide significant loci. Because single-nucleus TWAS can detect signals even in regions with only suggestive GWAS associations, we did not require a large number of loci, but we did impose a pragmatic minimum of 5 genome-wide significant loci as a baseline power threshold to avoid traits with essentially no robust GWAS signal. To our knowledge, there is no commonly accepted field-wide standard for a minimum locus count in TWAS studies, so this cutoff should be viewed as an arbitrary, study-specific choice. At the time we froze our GWAS summary statistic collection and finalized the analysis set, these four traits did not meet that threshold; more recent and better powered GWASs may now be available, but incorporating them would require a new analysis cycle that is beyond the scope of the present work.
**Excerpt**	"We began with 16 traits. Four showed insufficient power for S-snTWAS when analyzed individually, each yielding at most one FDR-significant gene and two genome-wide significant loci in their variant association results: binge-eating disorder[24], post-traumatic stress disorder (**PTSD**)[25], anxiety[26], and obsessive-compulsive disorder[27]. We imposed a pragmatic threshold of five genome-wide significant loci to ensure robust power for S-snTWAS analysis. Our primary analyses therefore use 12 well-powered NPD and NDD GWAS summary statistics[25,28–38] (Table S12)."

**<u>REF5-A-6.</u>** PheWAS control definition (0 vs. <2 phecodes)	
In the PheWAS, the definition of controls as "individuals with fewer than 2 phecodes" is unusual. Typically people with a single phecode would be excluded from the analysis, or possibly considered cases. This is not a major problem, because it would mostly impact power, but it is probably worth giving a justification.	
**Response**	We thank the reviewer for highlighting this point about our phenome-wide association study (**PheWAS**) control definition, which we agree should have been explained more clearly. In practice, the reviewer's intuition is correct: we see essentially identical results when using the canonical definition compared with our "2 versus <2" implementation, and the main effect of our choice is on power and pipeline simplicity.<br><br>In more detail, the canonical PheWAS implementation, for example, in the R PheWAS package, defines cases as individuals with at least two occurrences of a phecode-specific international classification of diseases (**ICD**) code, defines controls as individuals with no occurrences, and treats individuals with exactly one code as indeterminate "possible cases" who are typically excluded from analysis. Methodology reviews of PheWAS document substantial heterogeneity in how investigators define cases and controls, including different

	ICD code aggregation schemes, minimum code-count thresholds such as the “rule-of-two,” and study-specific exclusion rules, rather than a single field-wide standard [39,40].<br><br>As the reviewer notes, including single-code individuals in the reference (control) group increases the effective control sample size, so any misclassification introduced by coding a small fraction of true cases as controls is expected primarily to attenuate associations toward the null rather than create spurious positives, consistent with standard results for non-differential misclassification of binary outcomes [41,42]. To empirically verify this, we repeated the focused (brain traits) single-nucleus genetically regulated gene expression PheWAS (**snGReX-PheWAS**) using the canonical approach that excludes individuals with exactly one phecode and compared it directly with our “2 versus <2” implementation (Fig. R9). As shown in Fig. R8, the resulting z-scores are almost identical (Pearson’s correlation = 0.99569; 95% confidence interval: 0.99568-0.99570), supporting that our control definition has a negligible impact on inference and primarily affects power. A simple power calculation, using the same descriptive χ^2-based comparison we used previously[43], indicated that our control definition sacrifices only 1.6% statistical power compared with the stricter 0-phecode control definition. Specifically, we aligned overlapping snGReX-PheWAS associations under the two control definitions, computed χ^2 $(= z^2)$ for each association, and interpreted the percent decrease in mean χ^2 (when $\chi^2 \geq 1$) for the 0-vs-<2 definition as an approximate percent loss in effective power, which we considered negligible given the simplification to the analysis pipeline.

	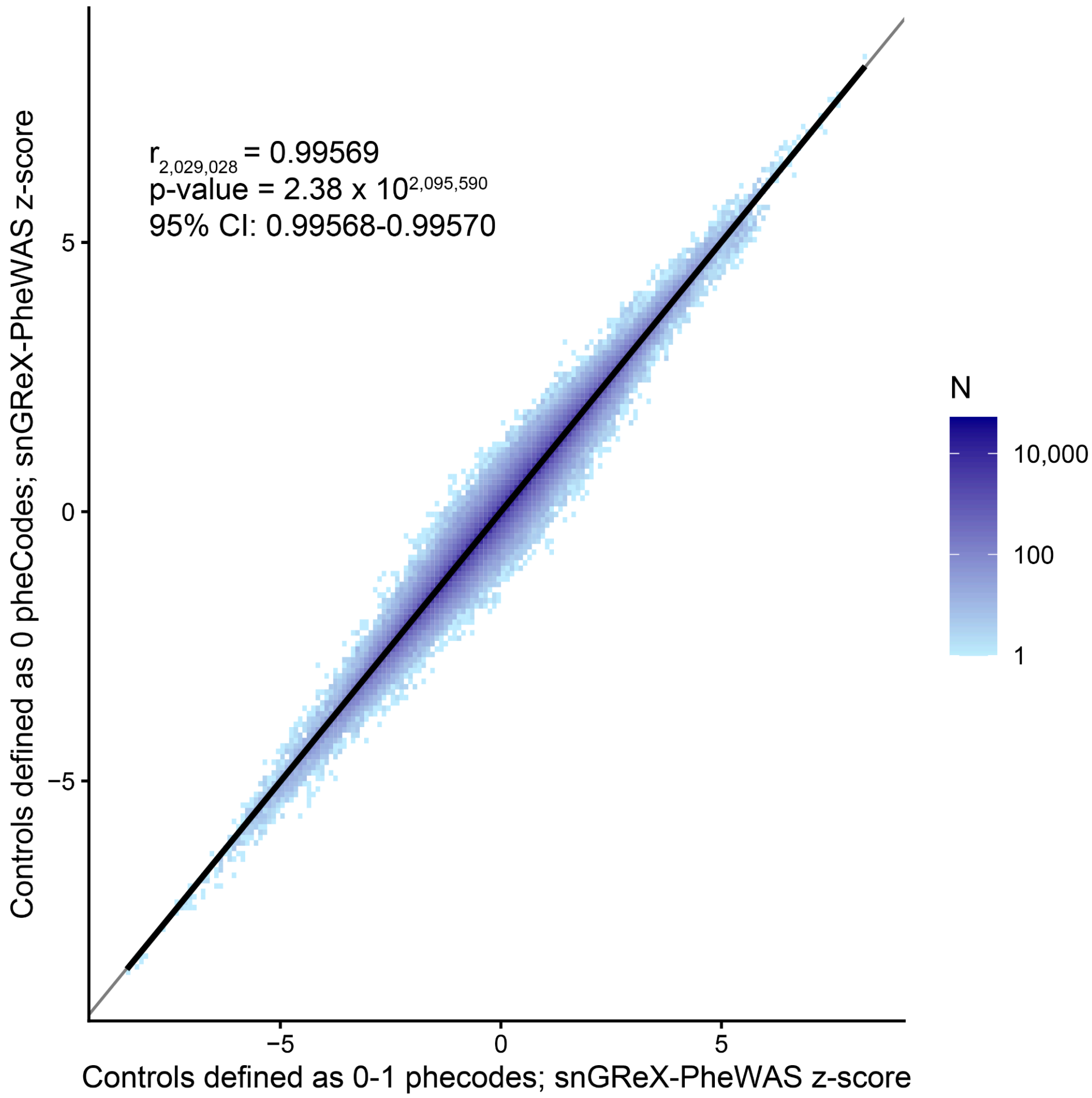<br><br>**Fig. R9 | Comparison of snGReX-PheWAS results under 0 or <2 phecodes phenotype control definition.** The x-axis shows snGReX-PheWAS z-scores using our original control definition (0 to 1 phecodes). The y-axis shows matched snGReX-PheWAS z-scores using the canonical control definition (strictly 0 phecodes, with individuals with 1 phecode removed). Color scale (“N”) indicates the number of associations falling in each 2D histogram bin (bin width 0.1 z-score). The annotated Pearson’s correlation coefficient (r), two-sided p value, and 95% confidence interval (“95% CI”) summarize the concordance between the two analyses.
**Action**	We have added this justification to the methods section.
**Excerpt**	“Individuals with at least 2 phecodes for a given trait were considered a case for both I-snTWAS and snGReX-PheWAS analyses. Due to the enrichment of neuropsychiatric disorders within the MVP and their high comorbidity, individuals with fewer than 2 phecodes were considered a control for both analyses.”

REF5-A-7. Interpretation of overlap metrics in Fig. 4A

In Figure 4A, is it possible that the definitions of the lower-left triangle and upper-right triangle are reversed? It doesn't make sense to me that the number of shared genes would be higher when requiring the cell-type to be shared between the traits as well.	
**Response**	We thank the reviewer for carefully checking the interpretation of Fig. 4A. The definitions and labeling of the lower-left and upper-right triangles are correct, and the apparent discrepancy arises from how overlaps are counted in each panel rather than from the cell-type requirement itself. In the lower-left triangle, we count gene-by-cell-type overlaps: the same gene can contribute multiple times if it is significant in the same cell-type for a given pair of disorders. For example, *CACNA1C* is significant in both bipolar disorder (**BD**) and schizophrenia (**SCZ**) in Class-Inhibitory Neurons (**IN**), and also significant in both BD and SCZ in Subclass-IN Adenosine Deaminase RNA Specific B2 expressing (**ADARB2**) and thus it contributes two overlaps (Class-IN and Subclass-IN ADARB2). In contrast, the upper-right triangle counts gene-level overlaps across any cell-types: a gene is counted once per disorder pair if it is significant in at least one cell-type in each disorder, regardless of whether the cell-types match. For example, *STAT6* is significant in major depressive disorder (**MDD**) only in Subclass-Perivascular Macrophage (**PVM**) and in Migraines in 4 cell-types (Class-Excitatory Neurons (**EN**), Class-Astrocytes (**Astro**), Subclass-IN Somatostatin expressing (**SST**), and Subclass-EN Layer 2-3 Intratelencephalic (**L2-3-IT**)) and despite the non-overlapping cell-types, it contributes one overlap. Because the lower-left triangle counts gene-cell-type combinations and the upper-right triangle collapses across cell-types to unique genes per disorder pair, the lower-left counts are often larger, although when genes tend to be implicated in different cell-types across disorders the upper-right counts can exceed the lower-left counts.
**Action**	We have clarified the Fig. 4A caption to explicitly state that the lower-left triangle reports counts of overlapping gene-cell-type associations (genes can appear multiple times across cell-types for a disorder pair), whereas the upper-right triangle reports counts of overlapping genes per disorder pair after collapsing across cell-types.
**Excerpt**	"The number and color in each cell indicate either the number of shared significant gene-cell-type combinations (*lower-left triangle*; genes can appear multiple times across cell-types for a disorder pair) or the number of shared significant genes regardless of cell-type origin (*upper-right triangle*) between each pair of disorders."

**REF5-A-8.** R^2_{CV} versus external R^2 validation comparison	
Please make a figure similar to Supplementary Figure 3 that compares the R2CV to the R2 of the models applied to the independent datasets. (Apologies if I missed this.)	
**Response**	We thank the reviewer for this suggestion and for their careful reading of the Supplementary material. A comparison of R^2_{CV} to out-of-sample R^2 in independent datasets is provided in Supplementary Fig. 27A,C.

**Action**	We have updated the caption for Supplementary Fig. 3 to explicitly note that a corresponding comparison of R^2_{CV} with out-of-sample R^2 in independent datasets is shown in Supplementary Fig. 27A,C.
**Excerpt**	"Similar plots for out-of-sample validation for EUR snTIMs are performed in Supplementary Fig. 27."

Minor comments:

**<u>REF5-B-1.</u>** Specify ROSMAP ancestry in validation analyses	
In "Brain snTIMs capture brain GReX with cell-type-specificity across ancestries", it would be helpful to note the ancestry of participants in the ROSMAP data.	
**Response**	We thank the reviewer for pointing this out. The Religious Orders Study/Memory and Aging Project (**ROSMAP**) participants used for external validation are EUR, and in this section, we compare them specifically to the EUR PsychAD snTIMs.
**Action**	We have revised the first mention of ROSMAP in the Results subsection "Brain snTIMs capture brain GReX with cell-type-specificity across ancestries" and in the Methods to state explicitly that the ROSMAP cohort used for validation consists of EUR individuals and that these analyses use the EUR PsychAD snTIMs.
**Excerpt**	"To validate snTIM performance out of sample, we used two independent genotype-gene expression datasets: snRNA-seq from the Religious Orders Study/Memory and Aging Project (**ROSMAP**)[44] EUR cohort, and bulk RNA-seq data from fluorescence-activated cell sorting-isolated microglia (**FACS-MG**)[45,46]."<br><br>"We performed external validation of EUR snTIMs using WGS and snRNA-seq from 396 Religious Orders Study/Memory and Aging Project (**ROSMAP**)[44] participants of European ancestry."

**<u>REF5-B-2.</u>** General clarity and wording improvements	
Clarity could be improved in several places, including	
**Response**	We thank the reviewer for carefully pointing out places where the clarity of our manuscript can be improved.
**Action**	We address each of the reviewer's specific comments on clarity in the responses below.

**<u>REF5-B-2a.</u>** Explicit fine-mapping PIP ≥ 0.5 definition

The way that genes that were implicated as potentially causal by fine-mapping are referenced is not very clear, in several places. E.g., “Notably, most of these key genes were also fine-mapped in the relevant cell-types (Table S16), supporting their causal involvement.” I think the authors are referring to genes that had PIP > 0.95 in fine-mapping, but that’s not clear from the statement.	
**Response**	We thank the reviewer for highlighting this ambiguity. Throughout the manuscript, we define “fine-mapped” genes as those with posterior inclusion probability (**PIP**) ≥ 0.5, not 0.95, and we agree that this threshold should be stated explicitly wherever we refer to fine-mapped genes.
**Action**	We have revised the text to specify the PIP threshold wherever fine-mapped genes are mentioned.
**Excerpt**	“Notably, most of these key genes were also fine-mapped in the relevant cell types (Table S18; PIP ≥ 0.5), supporting their likely causal role.”<br><br>“In each heatmap cell, the top and bottom numbers give the count of fine-mapped genes and loci (posterior inclusion probability (**PIP**) ≥ 0.5), respectively (after FDR adjustment).”

**<u>REF5-B-2b.</u>** Clarify “region-matched” Bulk S-TWAS phrasing	
In “Brain snTWAS increases power for both known and novel gene-trait association discovery”, the statement “Genes were classified as “novel” if the GTA was not identified by Bulk S-TWAS (Table S12; region-matched analysis of the same GWAS using homogenate models) or by MAGMA…” I’m not sure what is meant by a “region-matched analysis”	
**Response**	We thank the reviewer for pointing out this unclear phrasing. By “region-matched analysis” we meant that the Bulk S-TWAS comparator used homogenate DLPFC expression models applied to the same GWAS, matching the cortical region used to train the PsychAD snTIMs. We agree that this term is vague and have removed it in favor of a more explicit description.
**Action**	We have revised the sentence to remove “region-matched” and to state explicitly that the Bulk S-TWAS used homogenate DLPFC models.
**Excerpt**	“Genes were classified as “novel” if the GTA was not identified by Bulk S-TWAS (Table S14; homogenate DLPFC TWAS analysis of the same GWAS) or by Multi-marker Analysis of GenoMic Annotation (**MAGMA**) gene-based analysis (Table S15)[47,48], which detects genes through common variant aggregation of GWAS summary statistics alone.“

**<u>REF5-B-2c.</u>** Clarify RHOBTB2-paralytic interaction description
In “Ancestry-specific snTWAS uncovers shared biology and aids in fine-mapping of risk genes”, the statement “RHOBTB2’s plausibility is reinforced by functional work showing that BTB-domain

variants increase excitability in human iPSC-derived neurons and genetically interact with voltage-gated sodium channel pathways in Drosophila". I'm not sure what "genetically interact" means here, can you be more specific?	
**Response**	We thank the reviewer for asking us to be more precise about what we mean by "genetically interact." In the sentence in question, we are referring to Langhammer et al. 2025[49], who used both human induced pluripotent stemm cell (**iPSC**)-derived neurons and Drosophila to dissect RHOBTB2 function. In Drosophila, pan-neuronal overexpression of the RhoBTB ortholog induces seizure-like bang-sensitivity and wing phenotypes; a subsequent genetic interaction screen showed that altering the dosage of the voltage-gated sodium channel paralytic by RNA interference (**RNAi**) or overexpression had little effect alone but produced antagonistic or synergistic, non-additive modification of the RhoBTB overexpression phenotypes, often ameliorating bang-sensitivity and in some contexts exacerbating it. The authors interpret these findings as strong evidence for a functional *in vivo* genetic interaction between RhoBTB and paralytic rather than independent effects.
**Action**	To eliminate ambiguity, we have replaced the phrase "genetically interact with voltage-gated sodium channel pathways in Drosophila" with a more explicit statement.
**Excerpt**	"The biological plausibility of *RHOBTB2* is supported by functional studies showing that BTB-domain variants increase excitability in human induced pluripotent stem cell derived neurons and that altering the Drosophila sodium channel ortholog paralytic modifies RhoBTB-driven seizure-like phenotypes *in vivo*[49]."

**REF5-B-2d.** Clarify "6,095/31" gene-count comparison	
In the Discussion, "Compared to the only published snTWAS in MDD, we noted 6,095/31 more imputable/significant gene-cell-type combinations." The way the numbers are presented here is not very clear. This could probably be improve by listing them as "6,095 more imputable and 31 more significant gene-cell-type combinations".	
**Response & Action**	We thank the reviewer and have implemented this change.
**Excerpt**	"Relative to the only published brain snTWAS in major depressive disorder (**MDD**)[50], we observed 6,095 more imputable and 31 more significant gene-cell-type combinations."

**REF5-B-2e.** Define acronyms such as MSSM, HBCC, RADC, ADSP	
There are still a few instances of acronyms being referenced prior to, or without, definitions (e.g., MSSM, HBCC, RADC, ADSP).	
**Response**	We thank the reviewer for pointing out the use of acronyms without prior definition.

**Action**	We have expanded all acronyms we have identified in the text. We are also including an additional supplementary table to list all acronyms.

**<u>REF5-B-2f.</u>** Move MHC and PTCA method definitions earlier	
There are also some analyses or details in the Methods that are referenced before they are defined. It would be helpful to move the definition of the MHC region up to where it is first referenced, and the description of the PCTA methods up to the first time it is used.	
**Response**	We thank the reviewer for pointing out that these technical terms were not clearly defined.
**Action**	We have moved the definitions so that they appear at first use in the text. The Major histocompatibility complex (**MHC**) region is now defined where it is first mentioned, and the PTCA method is introduced earlier in the Methods section.
**Excerpt**	"Major histocompatibility complex region was custom defined to "28477797-33448354" (to match the definition used in other analyses, accounting for genome build differences); …"

**<u>REF5-B-2g.</u>** Justify exclusion of the MAPT locus	
I believe the references to removal of the MAPT locus due to potential for bias are specific to Alzheimer's disease. It would be helpful to clarify this for the broader readership.	
**Response**	We thank the reviewer for asking us to clarify our handling of the MAPT locus. While MAPT is best known in the context of Alzheimer's disease (**AD**), MAPT inversion haplotypes have strong effects across multiple neurodegenerative disorders, including Parkinson's disease (**PD**) and frontotemporal lobar degeneration[51]. Because this locus shows very strong, pleiotropic signals across several disorders included in our study, we removed it from cross-disorder analyses to avoid locus-specific biases.
**Action**	We have expanded the rationale for excluding the MAPT region in the Methods and explicitly noted a few relevant disorders.
**Excerpt**	"Finally, we note that we removed the *MAPT* locus (defined as chromosome 17:44928498−56807609 in GRCh38) due to the presence of haplotypes that may bias results[51]. Notably, these haplotypes have been found to play a large role in AD, PD, and other disorders[51], and thus inclusion of this region may bias results, especially when performing cross-disorder analysis."

**<u>REF5-B-2h.</u>** Define high linkage disequilibrium regions

In “Million Veteran Program Genotype Quality Control”, “high disequilibrium regions” needs to be defined.	
**Response**	We agree with the reviewer that “high disequilibrium regions” needed to be defined more clearly.
**Action**	We have added explicit genomic coordinates in parentheses when the term is first used. Please note that the list is not exhaustive, but includes frequently used regions.
**Excerpt**	“Using only autosomal variants, we filtered variants for MAF (≥ 0.01), Hardy-Weinberg equilibrium p-value ($< 1 \times 10^{-6}$), and removed high disequilibrium regions (GRCh37; chr6:25,500,000−33,500,000, chr8:8,135,000−12,000,000, and chr17:40,900,000−45,000,000)[52].”

**<u>REF5-B-2i.</u>** Rename ancestry PCs as genetic principal components	
In “GReX-PheWAS association analysis”, “top ten ancestral principal components”, it would be more accurate to call them “genetic principal components”, or even just “principal components”.	
**Response & Action**	We thank the reviewer for this terminology suggestion and have updated the phrasing accordingly.
**Excerpt**	“As in I-snTWAS, logistic regression analysis was performed while adjusting for sex, age, and top ten genetic principal components.”

**<u>REF5-B-2j.</u>** Detail TOPMed imputation and common SNP thresholds	
In “S-snTWAS validation”, “Genotypes were TOPMed-imputed and only common SNPs were utilized for model building.” This needs either references to further details or the details given in text, including the MAF threshold used to define common variants.	
**Response**	We agree with the reviewer that additional detail is needed regarding genotype quality control and the definition of “common” SNPs.
**Action**	We have expanded the “S-snTWAS validation” Methods section to describe imputation, minor allele frequency (**MAF**) thresholds and other quality control criteria, and to make explicit that common variants were defined using MAF cutoffs.
**Excerpt**	“Consequently, ancestry-specific SNPs were retained if they also had a matched superpopulation-specific MAF greater than or equal to 0.01 in 1000 Genomes Project to ensure SNP overlap with GWAS used in summary-level single-nucleus transcriptome-wide association study (**S-snTWAS**) analysis[53,54].”

	"To prepare the FACS-MG TIM, we utilized similar protocols to the PsychAD snTIMs. FACS-MG cohort samples were derived from one of three different sub-cohort (based on site of sample preparation). Genotypes were TOPMed-imputed and a EUR SNP reference panel similar to that used for PsychAD snTIM creation was used to initially select SNPs. 1000 Genomes Project overlap was not implemented for the reference panel used in FACS-MG TIM creation due to the application of the TIM to a single GWAS – SNP utilization for the FACS-MG AD S-TWAS was > 95%. Samples were filtered for missingness (≤ 0.01) and relatedness (KING filter = 0.0884). To perform population stratification, variants were filtered for MAF (≥ 0.05), missingness (≤ 0.01) and Hardy-Weinberg equilibrium p-value ($< 10^{-10}$). Variants were pruned using plink's "--indep-pairwise" function (1000 10 0.02). Twenty principal components were calculated and principal component analysis was used to determine EUR individuals (EUR selection ellipsoid defined using 3 standard deviations and 3 principal components). After ancestry filtering, sample-level quality control was performed. Variants were filtered for missingness (≤ 0.01) prior to sample filtering for missingness (≤ 0.01). Subsequently, only autosomal variants were retained, and variants were filtered for MAF (≥ 0.05) and Hardy-Weinberg equilibrium p-value ($< 10^{-6}$). High LD regions were then filtered out (Table S42) prior to pruning using plink's "--indep-pairwise" function (50 5 0.02). We then filtered for excess heterozygosity (≥ 3 standard deviations from the mean) prior to filtering samples for relatedness (KING filter = 0.0884). After retaining only quality-controlled EUR samples, downstream variant filtering was performed identically to PsychAD. RNA-seq was processed identically to PsychAD snRNA-seq, including the two step PEER factor optimization on the three sub-cohorts. After filtering, we retained 271 EUR individuals with genotypes and RNA-seq. Finally, the FACS-MG TIM was filtered identically to PsychAD snTIMs."

**REF5-B-2k.** Expand acronyms in figure legends and add table	
The figure legends and figures themselves contain many acronyms/abbreviations that are not defined in the figure legends. Definitions should be added to the legends as much as possible, or refer to a table elsewhere if needed.	
**Response**	We thank the reviewer for highlighting the heavy use of acronyms and abbreviations in the figures and figure legends. We agree that reducing and clarifying these improves readability.
**Action**	We have expanded or defined acronyms and abbreviations directly in each figure legend wherever possible, and we provide a separate table (Table S1) that lists and defines every acronym used in the manuscript.

**REF5-B-3.** Replace "trans-ancestry" with "multi-ancestry"

In the statement “Collectively, these results indicate that trans-ancestry snTWAS fine-mapping improves both cell-type-specific gene discovery and causal-gene prioritization across ancestries.” Change “trans-ancestry” to “multi-ancestry” to reflect currently acceptable terminology.	
**Response & Action**	We appreciate the reviewer’s suggestion about terminology and have replaced “trans-ancestry” with “multi-ancestry” throughout the manuscript where appropriate.
**Excerpt**	“Collectively, these results show that multi-ancestry snTWAS fine-mapping improves both cell-type-specific gene discovery and causal gene prioritization across ancestries.”

**<u>REF5-B-4.</u>** Report proportion of replaced SNP genotypes	
In “Variant-level filtering in the training cohort (PsychAD)”, “missing information for SNP predictors in existing TIMs were replaced with double the in-sample MAF (representing the in-sample average genotype), rounded to the nearest integer.” It would be helpful to give the proportion of variants where this was needed. If the proportion is high it would make more sense to perform imputation, if low, this is probably fine.	
**Response & Action**	We agree with the reviewer that it is important to report how often genotype replacement was required and to explain why this approach is appropriate. We now state that only 0.050% of genotypes used for EUR snTIM training and 0.069% of genotypes used for AFR and Admixed American (**AMR**) snTIM training required replacement. These instances correspond to donor-SNP pairs where genotype imputation failed, so further reduction would require improved upstream imputation rather than different downstream handling. Because elastic net requires a complete predictor matrix, in contrast to single-variant GWAS models that can simply omit missing SNPs on a test-by-test basis, we opted to replace this very small fraction of missing genotypes with twice the in-sample MAF (the expected genotype count) rather than exclude them.
**Excerpt**	“Overall, we replace about 0.050% of genotypes used for EUR snTIM training, and 0.069% each in genotypes used for AFR and AMR snTIM training.”

**<u>REF5-B-5.</u>** Compare imputable gene filters with GTEx and others	
In “Training of cell-type-specific PrediXcan models”, to help readers evaluate how the definition of imputable genes in this work compares to commonly used publicly available models, such as the GTEx v8 models released through PredictDB.	
**Response & Action**	We agree with the suggestion to provide context and have added a brief comparison of our filtering criteria with those used in Genotype-Tissue Expression (**GTEx**) v8, Zeng 2024 and the original PrediXcan models in the “Training of cell-type-specific PrediXcan models” section.

**Excerpt**	“These filtering criteria are comparable to that of other published TIMs – Genotype-Tissue Expression (**GTEx**) V8 Elastic Net models and Zeng-2024: CV p-value ≤ 0.05[50,55]; original PrediXcan models: CV $R^2_{CV} > 0.01$[56].”

**REF5-B-6.** Add table for Fig. 5E and clarify log scaling	
For Figure 5E, while I appreciate that the figure is trying to present a very complex, it is hard to interpret. Please ensure that this data is presented in Table format as well, for additional evidence of these findings. For Figure 5C, the scale of the plot is confusing. Why are the I-snTWAS significant “No” sections so small?	
**Response & Action**	We thank the reviewer for raising these points about Fig. 5. To improve interpretability of Fig. 5E, we have added a reference to Table S30 in the figure caption and added Table S2 to summarize all other tables and figures for greater accessibility. Table S30 reports the underlying counts and statistics in tabular form, providing an explicit numerical summary of the patterns shown in the figure. For Fig. 5C, the apparently small size of the “No” I-snTWAS significant sections is due to the x-axis being plotted on a log10 scale in order to accommodate the much smaller number of associations in AFR relative to EUR; we have now stated this clearly in the figure caption so that the scaling choice and its impact on the visual impression are transparent.
**Excerpt**	“The x-axis indicates the number of fine-mapped and significant associations and is log10 scaled.”

References

1. Lee, D. *et al.* Single-cell atlas of transcriptomic vulnerability across multiple neurodegenerative and neuropsychiatric diseases. *Nature* In Press.
2. Fullard, J. F. *et al.* Population-scale cross-disorder atlas of the human prefrontal cortex at single-cell resolution. *Sci. Data* (Under Revision).
3. Lee, D. *et al.* Single-cell atlas of transcriptomic vulnerability across multiple neurodegenerative and neuropsychiatric diseases. *medRxiv* 2024.10.31.24316513 (2024) doi:10.1101/2024.10.31.24316513.
4. Osorio, D. & Cai, J. J. Systematic determination of the mitochondrial proportion in human and mice tissues for single-cell RNA-sequencing data quality control. *Bioinformatics* **37**,

963–967 (2021).

5. Luecken, M. D. & Theis, F. J. Current best practices in single-cell RNA-seq analysis: a tutorial. *Mol. Syst. Biol.* **15**, e8746 (2019).
6. Ilicic, T. *et al.* Classification of low quality cells from single-cell RNA-seq data. *Genome Biol.* **17**, 29 (2016).
7. Hippen, A. A. *et al.* miQC: An adaptive probabilistic framework for quality control of single-cell RNA-sequencing data. *PLoS Comput. Biol.* **17**, e1009290 (2021).
8. Kavaliauskaite, G. & Madsen, J. G. S. Automatic quality control of single-cell and single-nucleus RNA-seq using valiDrops. *NAR Genom. Bioinform.* **5**, lqad101 (2023).
9. Stegle, O., Parts, L., Piipari, M., Winn, J. & Durbin, R. Using probabilistic estimation of expression residuals (PEER) to obtain increased power and interpretability of gene expression analyses. *Nat. Protoc.* **7**, 500–507 (2012).
10. Zeng, B. *et al.* Single-Nucleus Atlas of Cell-Type Specific Genetic Regulation in the Human Brain. *medRxiv* 2024.11.02.24316590 (2024) doi:10.1101/2024.11.02.24316590.
11. Hoffman, G. E. *et al.* Efficient differential expression analysis of large-scale single cell transcriptomics data using dreamlet. *bioRxivorg* (2024) doi:10.1101/2023.03.17.533005.
12. Cunningham, F. *et al.* Ensembl 2022. *Nucleic Acids Res.* **50**, D988–D995 (2022).
13. Nassar, L. R. *et al.* The UCSC Genome Browser database: 2023 update. *Nucleic Acids Res.* **51**, D1188–D1195 (2023).
14. Ongen, H., Buil, A., Brown, A. A., Dermitzakis, E. T. & Delaneau, O. Fast and efficient QTL mapper for thousands of molecular phenotypes. *Bioinformatics* **32**, 1479–1485 (2016).
15. Benjamini, Y., Drai, D., Elmer, G., Kafkafi, N. & Golani, I. Controlling the false discovery rate in behavior genetics research. *Behav. Brain Res.* **125**, 279–284 (2001).
16. Jajoo, A. *et al.* Leveraging genomic and transcriptomic data of diverse ancestry to uncover mechanisms of psychiatric risk in the adult and developing brain. *Nat. Commun.* **17**, 1179 (2025).

17. Bhattacharya, A. *et al.* Best practices for multi-ancestry, meta-analytic transcriptome-wide association studies: Lessons from the Global Biobank Meta-analysis Initiative. *Cell Genom* **2**, (2022).
18. Keys, K. L. *et al.* On the cross-population generalizability of gene expression prediction models. *PLoS Genet.* **16**, e1008927 (2020).
19. Kachuri, L. *et al.* Gene expression in African Americans, Puerto Ricans and Mexican Americans reveals ancestry-specific patterns of genetic architecture. *Nat. Genet.* **55**, 952–963 (2023).
20. Mogil, L. S. *et al.* Genetic architecture of gene expression traits across diverse populations. *PLoS Genet.* **14**, e1007586 (2018).
21. Ping, J. *et al.* Using genome and transcriptome data from African-ancestry female participants to identify putative breast cancer susceptibility genes. *Nat. Commun.* **15**, 3718 (2024).
22. Fullard, J. F. *et al.* Single-nucleus transcriptome analysis of human brain immune response in patients with severe COVID-19. *Genome Med.* **13**, 118 (2021).
23. McKusick, V. A. *Mendelian Inheritance in Man: A Catalog of Human Genes and Genetic Disorders*. (JHU Press, 1998).
24. Burstein, D. *et al.* Genome-wide analysis of a model-derived binge eating disorder phenotype identifies risk loci and implicates iron metabolism. *Nat. Genet.* (2023) doi:10.1038/s41588-023-01464-1.
25. Nievergelt, C. M. *et al.* International meta-analysis of PTSD genome-wide association studies identifies sex- and ancestry-specific genetic risk loci. *Nat. Commun.* **10**, 4558 (2019).
26. Dönertaş, H. M., Fabian, D. K., Valenzuela, M. F., Partridge, L. & Thornton, J. M. Common genetic associations between age-related diseases. *Nat Aging* **1**, 400–412 (2021).
27. International Obsessive Compulsive Disorder Foundation Genetics Collaborative

(IOCDF-GC) and OCD Collaborative Genetics Association Studies (OCGAS). Revealing the complex genetic architecture of obsessive-compulsive disorder using meta-analysis. *Mol. Psychiatry* **23**, 1181–1188 (2018).

28. Bellenguez, C. *et al.* New insights into the genetic etiology of Alzheimer's disease and related dementias. *Nat. Genet.* **54**, 412–436 (2022).
29. van Rheenen, W. *et al.* Common and rare variant association analyses in amyotrophic lateral sclerosis identify 15 risk loci with distinct genetic architectures and neuron-specific biology. *Nat. Genet.* **53**, 1636–1648 (2021).
30. Watson, H. J. *et al.* Genome-wide association study identifies eight risk loci and implicates metabo-psychiatric origins for anorexia nervosa. *Nat. Genet.* **51**, 1207–1214 (2019).
31. Demontis, D. *et al.* Genome-wide analyses of ADHD identify 27 risk loci, refine the genetic architecture and implicate several cognitive domains. *Nat. Genet.* **55**, 198–208 (2023).
32. Mullins, N. *et al.* Genome-wide association study of more than 40,000 bipolar disorder cases provides new insights into the underlying biology. *Nat. Genet.* **53**, 817–829 (2021).
33. Jansen, P. R. *et al.* Genome-wide analysis of insomnia in 1,331,010 individuals identifies new risk loci and functional pathways. *Nat. Genet.* **51**, 394–403 (2019).
34. Als, T. D. *et al.* Depression pathophysiology, risk prediction of recurrence and comorbid psychiatric disorders using genome-wide analyses. *Nat. Med.* **29**, 1832–1844 (2023).
35. International Multiple Sclerosis Genetics Consortium. Multiple sclerosis genomic map implicates peripheral immune cells and microglia in susceptibility. *Science* **365**, (2019).
36. Nalls, M. A. *et al.* Identification of novel risk loci, causal insights, and heritable risk for Parkinson's disease: a meta-analysis of genome-wide association studies. *Lancet Neurol.* **18**, 1091–1102 (2019).
37. Trubetskoy, V. *et al.* Mapping genomic loci implicates genes and synaptic biology in schizophrenia. *Nature* **604**, 502–508 (2022).
38. Sanchez-Roige, S. *et al.* Genome-Wide Association Study Meta-Analysis of the Alcohol

Use Disorders Identification Test (AUDIT) in Two Population-Based Cohorts. *Am. J. Psychiatry* **176**, 107–118 (2019).

39. Hebbring, S. J. The challenges, advantages and future of phenome-wide association studies. *Immunology* **141**, 157–165 (2014).
40. Wang, L. *et al.* Methodology in phenome-wide association studies: a systematic review. *J. Med. Genet.* **58**, 720–728 (2021).
41. Jurek, A. M., Greenland, S., Maldonado, G. & Church, T. R. Proper interpretation of non-differential misclassification effects: expectations vs observations. *Int. J. Epidemiol.* **34**, 680–687 (2005).
42. Burstein, D. *et al.* Detecting and Adjusting for Hidden Biases due to Phenotype Misclassification in Genome-Wide Association Studies. *medRxiv* (2023) doi:10.1101/2023.01.17.23284670.
43. Zhang, W. *et al.* Integrative transcriptome imputation reveals tissue-specific and shared biological mechanisms mediating susceptibility to complex traits. *Nat. Commun.* **10**, 3834 (2019).
44. Mathys, H. *et al.* Single-cell atlas reveals correlates of high cognitive function, dementia, and resilience to Alzheimer's disease pathology. *Cell* **186**, 4365–4385.e27 (2023).
45. Humphrey, J. *et al.* Long-read RNA-seq atlas of novel microglia isoforms elucidates disease-associated genetic regulation of splicing. *medRxiv* (2023) doi:10.1101/2023.12.01.23299073.
46. Kosoy, R. *et al.* Alzheimer's disease transcriptional landscape in ex vivo human microglia. *Nat. Neurosci.* (2025) doi:10.1038/s41593-025-02020-2.
47. Watanabe, K., Taskesen, E., van Bochoven, A. & Posthuma, D. Functional mapping and annotation of genetic associations with FUMA. *Nat. Commun.* **8**, 1826 (2017).
48. de Leeuw, C. A., Mooij, J. M., Heskes, T. & Posthuma, D. MAGMA: generalized gene-set analysis of GWAS data. *PLoS Comput. Biol.* **11**, e1004219 (2015).

49. Langhammer, F. *et al.* Deregulated ion channels contribute to RHOBTB2-associated developmental and epileptic encephalopathy. *Hum. Mol. Genet.* **34**, 639–650 (2025).
50. Zeng, L. *et al.* A single-nucleus transcriptome-wide association study implicates novel genes in depression pathogenesis. *Biol. Psychiatry* **96**, 34–43 (2024).
51. Pedicone, C., Weitzman, S. A., Renton, A. E. & Goate, A. M. Unraveling the complex role of MAPT-containing H1 and H2 haplotypes in neurodegenerative diseases. *Molecular Neurodegeneration* **19**, 43 (2024).
52. Jiang, L. *et al.* Genome-wide association analyses of common infections in a large practice-based biobank. *BMC Genomics* **23**, 672 (2022).
53. Fairley, S., Lowy-Gallego, E., Perry, E. & Flicek, P. The International Genome Sample Resource (IGSR) collection of open human genomic variation resources. *Nucleic Acids Res.* **48**, D941–D947 (2020).
54. 1000 Genomes Project Consortium *et al.* A global reference for human genetic variation. *Nature* **526**, 68–74 (2015).
55. Mancuso, N. *et al.* Large-scale transcriptome-wide association study identifies new prostate cancer risk regions. *Nat. Commun.* **9**, 4079 (2018).
56. Gamazon, E. R. *et al.* A gene-based association method for mapping traits using reference transcriptome data. *Nat. Genet.* **47**, 1091–1098 (2015).

Referee Comments

Referee #5 (Remarks to the Author):

The authors have done a really nice job responding thoroughly to our prior review. I have only a few very minor remaining comments:

<table>
<tr><th colspan="2">Wording of Discussions Statement on cross-ancestry snTIM comparison</th></tr>
<tr><td colspan="2">REF5-A-4a "Despite differences in training sample size, shared gene-cell-type models showed strong correlations in R2CV ..., suggesting that genetic regulation of expression is broadly consistent across ancestries, even when the contributing single nucleotide polymorphisms (SNPs) are different (Supplementary Notes 3 and 4)."

I think this is still a little misleading. I think it's fine to say the predictive performance of the models is similar, or that the variance of gene expression levels explained by genetic variants is similar across ancestries. Or maybe you could simply add the word "extent" : "suggesting that the extent of genetic regulation of expression is broadly consistent across ancestries..." As is, it still seems that you are saying that the genetic regulation itself is similar across ancestries, which your analyses seem to show is actually quite dissimilar.</td></tr>
<tr><td>Response & Action</td><td>We agree with the reviewer that the wording is misleading, and we have integrated their recommendation to say that the “extent to which gene expression is under genetic regulation” is broadly consistent across ancestries, rather than the gene expression itself.</td></tr>
<tr><td>Excerpt</td><td>“Despite differences in training sample size, shared gene-cell-type models showed strong correlations in R^2_{CV} across ancestries (EUR-AMR: ρ = 0.58, two-sided p-value = 3.45 × $10^{-1,158}$, N = 13,054; EUR-AFR: ρ = 0.54, two-sided p-value = 4.22 × $10^{-2,711}$, N = 36,070; AFR-AMR: ρ = 0.49, two-sided p-value = 1.29 × 10^{-633}, N = 10,637; Spearman; Fig. 1C; Supplementary Fig. 2B, 3, and 4; Table S11), indicating that the extent to which gene expression is under genetic regulation is broadly consistent across ancestries, even when the underlying single nucleotide polymorphisms (SNPs) which serve as gene expression predictors differ (Supplementary Notes 4 and 5). “</td></tr>
</table>

Wording of Discussions Statement on ancestry-matched study design
This is also still a little confusing to me, “While most prior work has emphasized the poor portability of EUR-trained models to other ancestries17,18, our results show that ancestry-matched designs can be highly informative for cross-ancestry NPD and NDD studies even with modest sample sizes, paralleling prior diverse ancestry blood monocyte TWASs19–21.” I think the use of matched-ancestry and cross-ancestry referring to analyses of the same datasets is getting me tangled up. I'm not sure how this reflects your finding that mismatches in ancestry when applying snTIMs to GWAS may lead to misidentified biological signals.

**Response & Action**	We thank the reviewer for their insight on the confusing wording used. We have revised the statement to clarify that **(1)** prior work in the field has found that EUR-trained TIMs do not perform accurately in other ancestries (PMID: 36341024, 32797036), and that **(2)** PsychAD snTIMs eliminates the need to apply EUR TIMs to AFR and AMR populations.
**Excerpt**	"Most prior work has emphasized the poor portability of EUR-trained TIMs to other ancestries[1,2]. Our work eliminates the need for mismatched ancestry application of TIMs, and we find that proper ancestry-matched study design can be highly informative for cross-ancestry NPD and NDD studies even with modest sample sizes, paralleling prior diverse ancestry blood monocyte TWASs[3–5]."

**Posterior Inclusion Probability threshold used**	
PIP of 0.5 is pretty low (I feel like it is more common to see 0.8 or even higher?). Might be worth mentioning as a limitation.	
**Response**	We thank the reviewer for their input in regards to PIP thresholding. We acknowledge that there exists a wide range of accepted PIP thresholds in fine-mapping literature. We based our chosen PIP threshold based on the thresholds most commonly chosen[6–10] and based on the logic that it indicated a greater likelihood than not that the association was causal (50%).

**KING algorithm and maximizing sample retention**	
No need to change, but just a comment for the future- King does not optimize for sample retention when removing individuals to minimize relatedness. See for example, PMID: 22996348.	
**Response**	We thank the reviewer for this information for our future work, and will take this into account.

Editorial Comments

**Data Presentation**	
Please ensure that data presented in a plot, chart or other visual representation format shows data distribution clearly (e.g. dot plots, box-and-whisker plots). When using bar charts, please overlay the corresponding data points (as dot plots) whenever possible and always for n ≤ 10. (Please see the following editorial for the rationale behind this request and an example https://www.nature.com/articles/s41551-017-0079).	
**Response**	We affirm that data distribution is shown for all plots and charts. For bar charts, individual data points are overlaid or provided in an accompanying supplementary figure wherever possible. There are no bar charts with n ≤ 10.

<table>
<tr><td colspan="2">Statistics</td></tr>
<tr><td colspan="2">Wherever statistics have been derived (e.g. error bars, box plots, statistical significance) the legend needs to provide and define the n number (i.e. the sample size used to derive statistics) as a precise value (not a range), using the wording “n=X biologically independent samples/animals/cells/independent experiments/n= X cells examined over Y independent experiments” etc. as applicable.

Please note that this information is missing in the legend(s) of figure(s) 2B, C; extended data figure(s) 3B</td></tr>
<tr><td>Response & Action</td><td>We acknowledge this oversight, and we have added the information in the captions for the relevant figures.</td></tr>
<tr><td>Excerpt</td><td>Fig. 2B caption: The horizontal line around each point reflects the 95% confidence interval. Accompanying statistics, including sample sizes used to derive confidence intervals (column “Intersection”) and exact p-value (column “p-value”) are provided in Table S13.

Fig. 2C caption: The horizontal line around each point reflects the 95% confidence interval. Accompanying statistics, including sample sizes used to derive confidence intervals (column “FinerCT Novel”) and exact p-value (column “p-value”) are provided in Table S17.

Extended Data Fig. 3B caption: “Analysis is performed on 37,038 AD cases and 401,544 controls (Table S12).”</td></tr>
</table>

<table>
<tr><td colspan="2">Replicates & Error Bars</td></tr>
<tr><td colspan="2">Please note that statistics such as error bars significance and p values cannot be derived from n<3 and must be removed from all such cases. We strongly discourage deriving statistics from technical replicates, unless there is a clear scientific justification for why providing this information is important. Conflating technical and biological variability, e.g., by pooling technically replicates samples across independent experiments is strongly discouraged. (For examples of expected description of statistics in figure legends, please see the following https://www.nature.com/articles/s41467-019-11636-5 or https://www.nature.com/articles/s41467-019-11510-4).

All error bars need to be defined in the legends (e.g. SD, SEM) together with a measure of centre (e.g. mean, median). For example, the legends should state something along the lines of “Data are presented as mean values +/- SEM” as appropriate. All box plots need to be defined in the legends in terms of minima, maxima, centre, bounds of box and whiskers and percentile.

Legends requiring revision:
1. Please note that the error bars/error bands need to be defined in the legend(s) of figure(s) 2B,C; 5B
2. Please note that the measure of centre for the error bars/error bands needs to be defined in the legend(s) of figure(s) 6A; extended data figure(s) 3B</td></tr>
<tr><td>Response & Action</td><td>We have added the appropriate information to the captions and indicated the adjusted section below.</td></tr>
</table>

**Excerpt**	Fig. 2B: “The horizontal line around each point reflects the 95% confidence interval.”<br>Fig. 2C: “The horizontal line around each point reflects the 95% confidence interval.”<br>Fig. 5B: “The shaded area represents the 95% confidence interval.”<br>Fig. 6A: “The line represents the mean correlation between the two ancestries indicated.”<br>Extended Data Fig. 3B: “The circle represents the effect size, the horizontal bars represent the 95% confidence intervals, and asterisks mark false discovery rate (**FDR**)-significant associations.”

**Indication of statistical test used**	
The figure legends must indicate the statistical test used. Where appropriate, please indicate in the figure legends whether the statistical tests were one-sided or two-sided and whether adjustments were made for multiple comparisons. For null hypothesis testing, please indicate the test statistic (e.g. F, t, r) with confidence intervals, effect sizes, degrees of freedom and P values noted. Please provide the test results (e.g. P values) as exact values whenever possible and with confidence intervals noted.<br><br>Legends requiring revision:<br>1. Please indicate the statistical test used for data analysis and where appropriate, please specify whether it was one-sided or two-sided and whether adjustments were made for multiple comparisons, in the legend(s) of figure(s) 2A, B, C; 3C; 6B, C; extended data figure(s) 3C, D; 4B, C<br>2. Please note that the information on whether the statistical test used was one-sided or two-sided, where appropriate, is missing in the legend(s) of figure(s) 4B, C; 6A; extended data figure(s) 4A<br>3. Please note that the exact p value should be provided, when possible, in the legend(s) of figure(s) 2B, C	
**Response & Action**	We appreciate the clear documentation of these oversights, and we have corrected them accordingly.
**Excerpt**	**Figure Changes**<br>Fig. 2A: “(logistic regression analysis; false discovery rate (**FDR**)-adjusted two-sided p-value ≤ 0.05; FDR adjustment is performed across all traits and TIMs for parity)”<br><br>Fig. 2B:<br>“fisher’s exact test”; “(* FDR-adjusted two-sided p-value ≤ 0.05, ** FDR-adjusted two-sided p-value ≤ 0.01, *** FDR-adjusted two-sided p-value ≤ 0.001)”<br>“The horizontal line around each point reflects the 95% confidence interval. Accompanying statistics, including sample sizes used to derive confidence intervals (column “Intersection”) and exact p-value (column “p-value”) are provided in Table S13.”<br><br>Fig. 2C:<br>“fisher’s exact test”

	"FDR-adjusted two-sided p-value ≤ 0.05"<br>"The horizontal line around each point reflects the 95% confidence interval. Accompanying statistics, including sample sizes used to derive confidence intervals (column "FinerCT Novel") and exact p-value (column "p-value") are provided in Table S17."<br><br>Fig. 3C:<br>"logistic regression analysis; false discovery rate (**FDR**)-adjusted two-sided p-value ≤ 0.05"<br>"Cochran's Q test false discovery rate (**FDR**)-adjusted two-sided p-value ≤ 0.05"<br><br>Fig. 6A:<br>"We selected significant genes from our summary-level and individual-level single-nucleus transcriptome-wide association studies (**S-snTWAS** and **I-snTWAS**; false discovery rate (**FDR**)-adjusted two-sided p-value ≤ 0.05), and performed PheWAS on the top associations (Bonferroni-adjusted two-sided p-value ≤ 0.05 across all confidently imputable associations within each cell-type; 623 genes) to maximize analytical yield while remaining within computational constraints. "<br><br>Fig. 6B:<br>"logistic regression FDR-adjusted two-sided p-value ≤ 0.05"<br>"Cochran's Q test p-value (FDR-adjusted two-sided p-value ≤ 0.05)"<br><br>Fig. 6C:<br>"logistic regression analysis; FDR-adjusted two-sided p-value ≤ 0.05"<br><br>**Extended Data Figure Changes**<br>Extended data fig. 3C:<br>"Correlation Adjusted MEan RAnk (**CAMERA**) two-sided p-value; asterisks indicate FDR-adjusted two-sided p-value ≤ 0.05"<br><br>Extended data fig. 3D:<br>"Statistical test, p-value correction, etc.. are performed identically to panel C."<br><br>Extended data fig. 4A:<br>"Asterisks mark false discovery rate (**FDR**)-adjusted two-sided p-value ≤ 0.05."<br><br>Extended data fig. 4B:<br>"Heatmap for the Correlation Adjusted MEan RAnk (**CAMERA**) enrichment"<br>"Asterisks indicate FDR-adjusted two-sided p-value ≤ 0.05"<br><br>Extended data fig. 4C:<br>"Asterisks indicate Fisher's exact test FDR-adjusted two-sided p-value ≤ 0.05."

Reproducibility

Please state in the legends how many times each experiment was repeated independently with similar results. This is needed for all experiments, but is particularly important wherever results from representative experiments (such as micrographs) are shown. If space in the legends is limiting, this information can be included in a section titled “Statistics and Reproducibility” in the methods section.

**Response & Action**	While we do not do traditional wet-lab biological replications, we utilize data using technical replicates and provide appropriate citations in text. Additionally, we have added a methods section entitled “Statistics and Reproducibility” where we document replication of results using multiple cohorts.
**Excerpt**	“Statistics and Reproducibility<br>To demonstrate reproducibility, we performed multiple independent and internal validation analyses. Here, we summarize 5 key examples. **(1)** We performed out-of-sample replication of PsychAD snTIMs in the ROSMAP and FACS-MG cohorts (Supplementary Fig. 26A and 26C). We correlated imputed GReX with observed PEER-residualized snRNA-seq expression and found strong concordance between out-of-sample performance (R^2_{out}) and cross-validation performance (R^2_{CV}) (Spearman’s $\rho = 0.63$; n = 70,156; p-value = $3.91 \times 10^{-7,844}$, and $\rho = 0.55$; N = 1,853, p-value = 1.67×10^{-149}, respectively). **(2)** We compared S-snTWAS results derived from PsychAD snTIMs with those obtained using independent transcriptomic imputation models, including previously published snTIMs[11] and a microglia-specific TIM derived from FACS-MG[12] (Supplementary Fig. 26B and 26D). In both cases, we observed strong concordance (Pearson’s $r_{(13,760)} = 0.78$; p-value = $1.34 \times 10^{-2,772}$ and Pearson’s $r_{(733)} = 0.87$; p-value = 1.32×10^{-231}, respectively). **(3)** To address potential bias in the cross-disorder analysis from sample overlap across GWAS datasets, we replicated the analysis replacing S-snTWAS data with independent MVP I-snTWAS data and evaluated sign concordance of results (Supplementary Fig. 14). Overall, 90% of cross-disorder associations (5,162 of 5,721) showed consistent direction of effect. **(4)** We further compared S-snTWAS with MVP EUR I-snTWAS (Fig. 5A). Although the overall Pearson correlation across all associations was modest, progressive thresholding correlation analysis (PTCA) demonstrated strong concordance among top-ranked associations. **(5)** Finally, snTIM training incorporated 5-fold cross-validation, consistent with the broader use of cross-validation for evaluating PrediXcan-style transcriptomic imputation models.”

**Data Availability**
This journal strongly supports public availability of data and custom code associated with the paper in a persistent repository where they can be freely and enduringly accessed or as a supplementary data file when no appropriate repository is available. If data and code can only be shared on request, please explain why in your data Availability Statement, and also in the correspondence with your editor. For more information, please refer to https://www.nature.com/nature-research/editorial-policies/reporting-standards#availability-of-data

Please ensure that datasets deposited in public repositories are now publicly accessible, and that accession codes or DOI are provided in the "Data Availability" section. As long as these datasets are not public, we cannot proceed with the acceptance of your paper. For data that have been obtained from publicly available sources, please provide a URL and the specific data product name in the data availability statement. Data with a DOI should be further cited in the methods reference section.	
**Response**	We have made the Zenodo link (doi: 10.5281/zenodo.17435917) public, and added a parallel portal for data access on Synapse (https://doi.org/10.7303/syn63181047).

Reporting Summary Comments

**Statistics**	
Please see the extended comments document for a list of figures and figure legends that require additional information.	
**Response & Action**	Thank you for the highlighted list of figure/figure legends. We acknowledge these issues and we document our actions taken above.

**Software and code**	
Please ensure the following software/packages/tools/algorithms are mentioned in the manuscript as well, since they have been listed in the reporting summary: python, etc.<br><br>Please ensure all the data collection/data analysis software/tools/algorithms/packages mentioned in the manuscript are also listed in the reporting summary (with version numbers): EIGENSOFT, etc.	
**Response & Action**	We have added the appropriate information in the manuscript and reporting summary where indicated. We have also added related details to software used by the MVP data core to the reporting summary, and appropriately described it in the methods section of the manuscript.
**Excerpt**	"Pre- and post-processing was done using standard scripts in R, python2, and python3."<br><br>"Finally, we performed S-TWAS for our TIMs (Data S1) using Summary-PrediXcan (**S-PrediXcan**)[13] and python2."<br><br>"The MVP data core called genotypes using Assay for Transposase-Accessible Chromatin (**ATAC**) ATAC Primer Tool (**APT**) version 2.11.3, Affy2vcf, plink[14], and MVP 1.0 array library r6. Genotyping was validated using three plates of 1000 Genome samples. Rigorous genotype quality control, such as plate normalization, was used to improve the accuracy of genotype calling. Genotype imputation was performed using the TOPMed[15] reference, SHAPEIT4 v4.1.3[16], and Minimac4[17], and genetic principal components were generated using EIGENSOFT v.6[18,19]."

Data	
Please provide a valid and accessible web-link for PsychAD snTIMs and associated data here in the RS and the data availability section of the manuscript. Also, please ensure that this data is publicly released by the time you resubmit your final manuscript.<br><br>Please mention all the databases/datasets used in the study along with appropriately accessible links/accession-codes in the manuscript under the “Data availability” section as well as in this reporting summary: GRCh38 , etc<br><br>Please provide a complete data availability statement here in the reporting summary, as provided in the manuscript.	
**Response & Action**	We have made links for all associated data (snTIMs and associated covariance matrices) public. We have also amended the data availability statement to make references to all available PsychAD data.
**Excerpt**	“All results are included either in the main text or provided in Supplementary Tables or Data. We are also making snTIMs and accompanying SNP covariance matrices publicly available on Zenodo (doi: 10.5281/zenodo.17435917) and Synapse (https://doi.org/10.7303/syn63181047).<br>PsychAD single nucleus transcriptomics data is available via the AD Knowledge Portal (https://adknowledgeportal.org) and Synapse (https://doi.org/10.7303/syn60084804). ”

Life sciences study design: Sample size	
Since no sample size calculation was done, please provide a rationale for why these sample sizes are sufficient for PsychAD cohort experiments.<br><br>For all other experiments, Please describe how the number of datasets involved in the study was determined and explain why these are sufficient for the analysis.	
**Response & Action**	We utilized all available samples from PsychAD per budget availability. No samples were removed. The high out-of-sample replication of psychAD snTIMs in ROSMAP demonstrates a sufficient sample size for accurate snTIM creation.<br><br>For the GWAS data utilized in this study, we employed a pragmatic threshold of five genome-wide significant loci to ensure adequate power for S-snTWAS analysis. This threshold resulted in a minimum of 80 significant snTWAS genes at the class-level.
**Excerpt**	“We began with 16 traits. Four showed insufficient power for S-snTWAS when analyzed individually, each yielding at most one FDR-significant gene and two genome-wide significant loci in their variant association results: binge-eating disorder[20], post-traumatic stress disorder

	(**PTSD**)[21], anxiety[22], and obsessive-compulsive disorder[23]. We imposed a pragmatic threshold of five genome-wide significant loci to ensure robust power for S-snTWAS analysis. Our primary analyses therefore use 12 well-powered NPD and NDD GWAS summary statistics[21,24–34] (Table S12)."

**Life sciences study design: Replication**	
For experiments mentioned here, Please state how often the replication was performed.<br><br>Please state how often the experiments other than those mentioned in the reporting summary were replicated or performed independently and also confirm if all attempts at replication were successful.	
**Response & Action**	We have added a section to the methods entitled "Statistics and Reproducibility" to summarize all analyses we reproduced.
**Excerpt**	"Statistics and Reproducibility<br>To demonstrate reproducibility, we performed multiple independent and internal validation analyses. Here, we summarize 5 key examples. **(1)** We performed out-of-sample replication of PsychAD snTIMs in the ROSMAP and FACS-MG cohorts (Supplementary Fig. 26A and 26C). We correlated imputed GReX with observed PEER-residualized snRNA-seq expression and found strong concordance between out-of-sample performance (R^2_{out}) and cross-validation performance (R^2_{CV}) (Spearman's ρ = 0.63; n = 70,156; p-value = $3.91 \times 10^{-7,844}$, and ρ = 0.55; N = 1,853, p-value = 1.67×10^{-149}, respectively). **(2)** We compared S-snTWAS results derived from PsychAD snTIMs with those obtained using independent transcriptomic imputation models, including previously published snTIMs[11] and a microglia-specific TIM derived from FACS-MG[12] (Supplementary Fig. 26B and 26D). In both cases, we observed strong concordance (Pearson's $r_{(13,760)}$ = 0.78; p-value = $1.34\times10^{-2,772}$ and Pearson's $r_{(733)}$ = 0.87; p-value = 1.32×10^{-231}, respectively). **(3)** To address potential bias in the cross-disorder analysis from sample overlap across GWAS datasets, we replicated the analysis replacing S-snTWAS data with independent MVP I-snTWAS data and evaluated sign concordance of results (Supplementary Fig. 14). Overall, 90% of cross-disorder associations (5,162 of 5,721) showed consistent direction of effect. **(4)** We further compared S-snTWAS with MVP EUR I-snTWAS (Fig. 5A). Although the overall Pearson correlation across all associations was modest, progressive thresholding correlation analysis (PTCA) demonstrated strong concordance among top-ranked associations. **(5)** Finally, snTIM training incorporated 5-fold cross-validation, consistent with the broader use of cross-validation for evaluating PrediXcan-style transcriptomic imputation models."

**Life sciences study design: Blinding**
For experiments other than those mentioned here, please describe whether the investigators were blinded to group allocation during data collection and/or analysis. If blinding was not possible, describe why OR explain why blinding was not relevant to those experiments.

**Response**	No blinding was performed for the analyses described in this study. Blinding was not applicable because the study consisted exclusively of computational analyses performed on pre-existing genomic and transcriptomic datasets without experimental intervention or subjective outcome assessment.

References

1. Bhattacharya, A. *et al.* Best practices for multi-ancestry, meta-analytic transcriptome-wide association studies: Lessons from the Global Biobank Meta-analysis Initiative. *Cell Genom* **2**, (2022).
2. Keys, K. L. *et al.* On the cross-population generalizability of gene expression prediction models. *PLoS Genet.* **16**, e1008927 (2020).
3. Kachuri, L. *et al.* Gene expression in African Americans, Puerto Ricans and Mexican Americans reveals ancestry-specific patterns of genetic architecture. *Nat. Genet.* **55**, 952–963 (2023).
4. Mogil, L. S. *et al.* Genetic architecture of gene expression traits across diverse populations. *PLoS Genet.* **14**, e1007586 (2018).
5. Ping, J. *et al.* Using genome and transcriptome data from African-ancestry female participants to identify putative breast cancer susceptibility genes. *Nat. Commun.* **15**, 3718 (2024).
6. Strober, B. J., Zhang, M. J., Amariuta, T., Rossen, J. & Price, A. L. Fine-mapping causal tissues and genes at disease-associated loci. *Nat. Genet.* **57**, 42–52 (2025).
7. Zhang, J., Fang, X., Ye, Q., Yin, X. & Ye, D. The shared genetic architecture underlying the autoimmune and cardiovascular disease: a multivariate genome-wide analysis. *Cardiovasc. Diabetol.* **25**, 44 (2026).
8. McManus, J. N. J., Lovelett, R. J., Lowengrub, D. & Christensen, S. A unifying statistical framework to discover disease genes from GWASs. *Cell Genom.* **3**, 100264 (2023).

9. Morozova, O., Levina, O., Uusküla, A. & Heimer, R. Comparison of subset selection methods in linear regression in the context of health-related quality of life and substance abuse in Russia. *BMC Med. Res. Methodol.* **15**, 71 (2015).

10. Zeng, B. & Gibson, G. PolyQTL: Bayesian multiple eQTL detection with control for population structure and sample relatedness. *Bioinformatics* **35**, 1061–1063 (2019).

11. Zeng, L. *et al.* A single-nucleus transcriptome-wide association study implicates novel genes in depression pathogenesis. *Biol. Psychiatry* **96**, 34–43 (2024).

12. Kosoy, R. *et al.* Alzheimer's disease transcriptional landscape in ex vivo human microglia. *Nat. Neurosci.* (2025) doi:10.1038/s41593-025-02020-2.

13. Barbeira, A. N. *et al.* Exploring the phenotypic consequences of tissue specific gene expression variation inferred from GWAS summary statistics. *Nat. Commun.* **9**, 1825 (2018).

14. Purcell, S. *et al.* PLINK: a tool set for whole-genome association and population-based linkage analyses. *Am. J. Hum. Genet.* **81**, 559–575 (2007).

15. Taliun, D. *et al.* Sequencing of 53,831 diverse genomes from the NHLBI TOPMed Program. *Nature* **590**, 290–299 (2021).

16. Delaneau, O., Zagury, J.-F., Robinson, M. R., Marchini, J. L. & Dermitzakis, E. T. Accurate, scalable and integrative haplotype estimation. *Nat. Commun.* **10**, 5436 (2019).

17. Das, S. *et al.* Next-generation genotype imputation service and methods. *Nat. Genet.* **48**, 1284–1287 (2016).

18. Patterson, N., Price, A. L. & Reich, D. Population structure and eigenanalysis. *PLoS Genet.* **2**, e190 (2006).

19. Price, A. L. *et al.* Principal components analysis corrects for stratification in genome-wide association studies. *Nat. Genet.* **38**, 904–909 (2006).

20. Burstein, D. *et al.* Genome-wide analysis of a model-derived binge eating disorder phenotype identifies risk loci and implicates iron metabolism. *Nat. Genet.* (2023)

doi:10.1038/s41588-023-01464-1.

21. Nievergelt, C. M. *et al.* International meta-analysis of PTSD genome-wide association studies identifies sex- and ancestry-specific genetic risk loci. *Nat. Commun.* **10**, 4558 (2019).
22. Dönertaş, H. M., Fabian, D. K., Valenzuela, M. F., Partridge, L. & Thornton, J. M. Common genetic associations between age-related diseases. *Nat Aging* **1**, 400–412 (2021).
23. International Obsessive Compulsive Disorder Foundation Genetics Collaborative (IOCDF-GC) and OCD Collaborative Genetics Association Studies (OCGAS). Revealing the complex genetic architecture of obsessive-compulsive disorder using meta-analysis. *Mol. Psychiatry* **23**, 1181–1188 (2018).
24. Bellenguez, C. *et al.* New insights into the genetic etiology of Alzheimer's disease and related dementias. *Nat. Genet.* **54**, 412–436 (2022).
25. van Rheenen, W. *et al.* Common and rare variant association analyses in amyotrophic lateral sclerosis identify 15 risk loci with distinct genetic architectures and neuron-specific biology. *Nat. Genet.* **53**, 1636–1648 (2021).
26. Watson, H. J. *et al.* Genome-wide association study identifies eight risk loci and implicates metabo-psychiatric origins for anorexia nervosa. *Nat. Genet.* **51**, 1207–1214 (2019).
27. Demontis, D. *et al.* Genome-wide analyses of ADHD identify 27 risk loci, refine the genetic architecture and implicate several cognitive domains. *Nat. Genet.* **55**, 198–208 (2023).
28. Mullins, N. *et al.* Genome-wide association study of more than 40,000 bipolar disorder cases provides new insights into the underlying biology. *Nat. Genet.* **53**, 817–829 (2021).
29. Jansen, P. R. *et al.* Genome-wide analysis of insomnia in 1,331,010 individuals identifies new risk loci and functional pathways. *Nat. Genet.* **51**, 394–403 (2019).
30. Als, T. D. *et al.* Depression pathophysiology, risk prediction of recurrence and comorbid psychiatric disorders using genome-wide analyses. *Nat. Med.* **29**, 1832–1844 (2023).
31. International Multiple Sclerosis Genetics Consortium. Multiple sclerosis genomic map

implicates peripheral immune cells and microglia in susceptibility. *Science* **365**, (2019).

32. Nalls, M. A. *et al.* Identification of novel risk loci, causal insights, and heritable risk for Parkinson's disease: a meta-analysis of genome-wide association studies. *Lancet Neurol.* **18**, 1091–1102 (2019).

33. Trubetskoy, V. *et al.* Mapping genomic loci implicates genes and synaptic biology in schizophrenia. *Nature* **604**, 502–508 (2022).

34. Sanchez-Roige, S. *et al.* Genome-Wide Association Study Meta-Analysis of the Alcohol Use Disorders Identification Test (AUDIT) in Two Population-Based Cohorts. *Am. J. Psychiatry* **176**, 107–118 (2019).
