# Supplementary material for: Single-nucleus transcriptome-wide association study of human brain disorders
Source: Nature. 2026 Sep 23;657(8133):1016–26. doi: 10.1038/s41586-026-10836-6 (PMC13600940; doi:10.1038/s41586-026-10836-6)
Supplement: Supplementary file 5 [file 41586_2026_10836_MOESM4_ESM.zip › 2024-09-19723C-s4/FigS1.pdf]

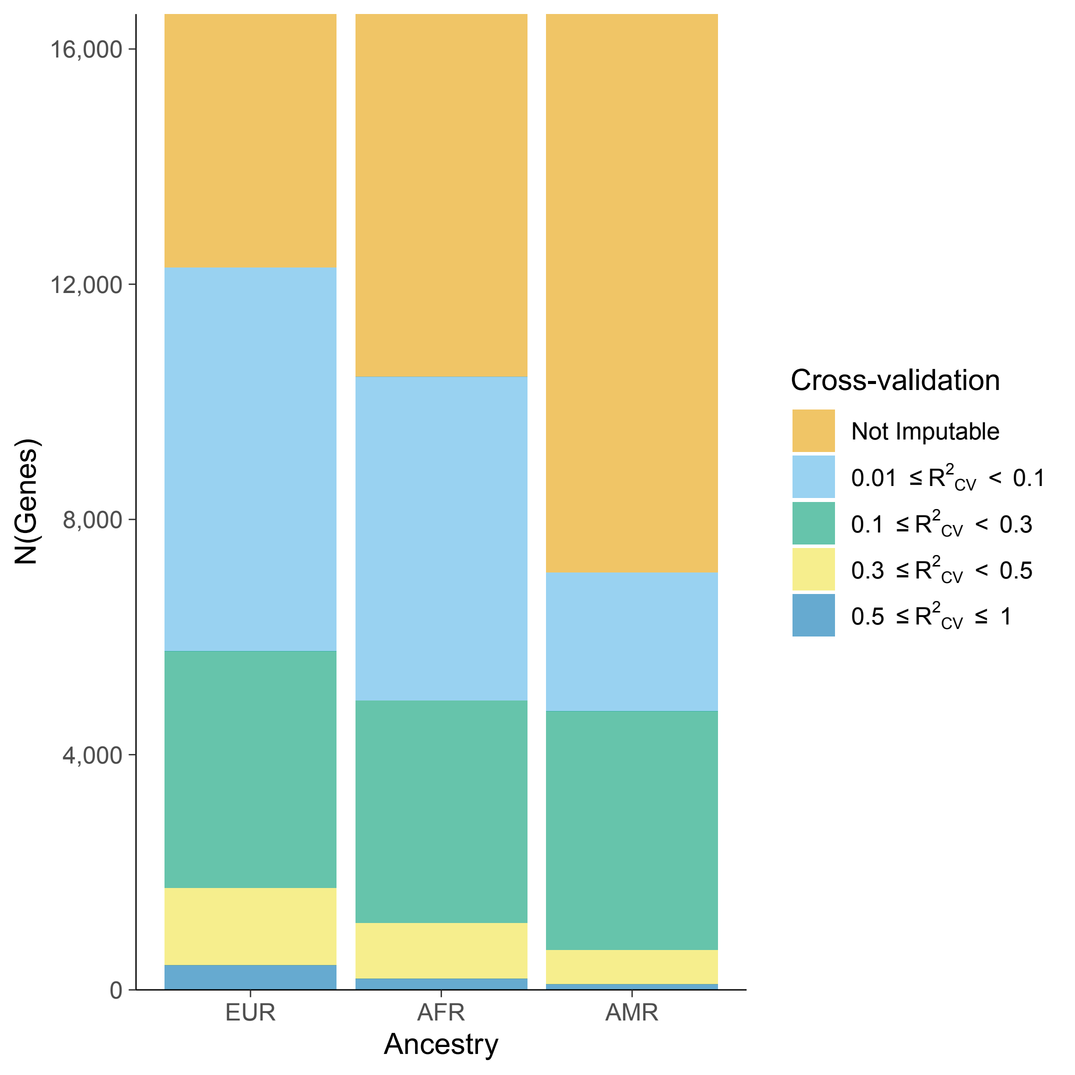

16,000
12,000
8,000
4,000
0
N(Genes)
EUR
AFR
AMR
Ancestry
Cross-validation
Not Imputable
0.01 ≤ R^2_{CV} < 0.1
0.1 ≤ R^2_{CV} < 0.3
0.3 ≤ R^2_{CV} < 0.5
0.5 ≤ R^2_{CV} ≤ 1
