# Supplementary material for: Single-nucleus transcriptome-wide association study of human brain disorders
Source: Nature. 2026 Sep 23;657(8133):1016–26. doi: 10.1038/s41586-026-10836-6 (PMC13600940; doi:10.1038/s41586-026-10836-6)
Supplement: Supplementary file 5 [file 41586_2026_10836_MOESM4_ESM.zip › 2024-09-19723C-s4/FigS10_part1.pdf]

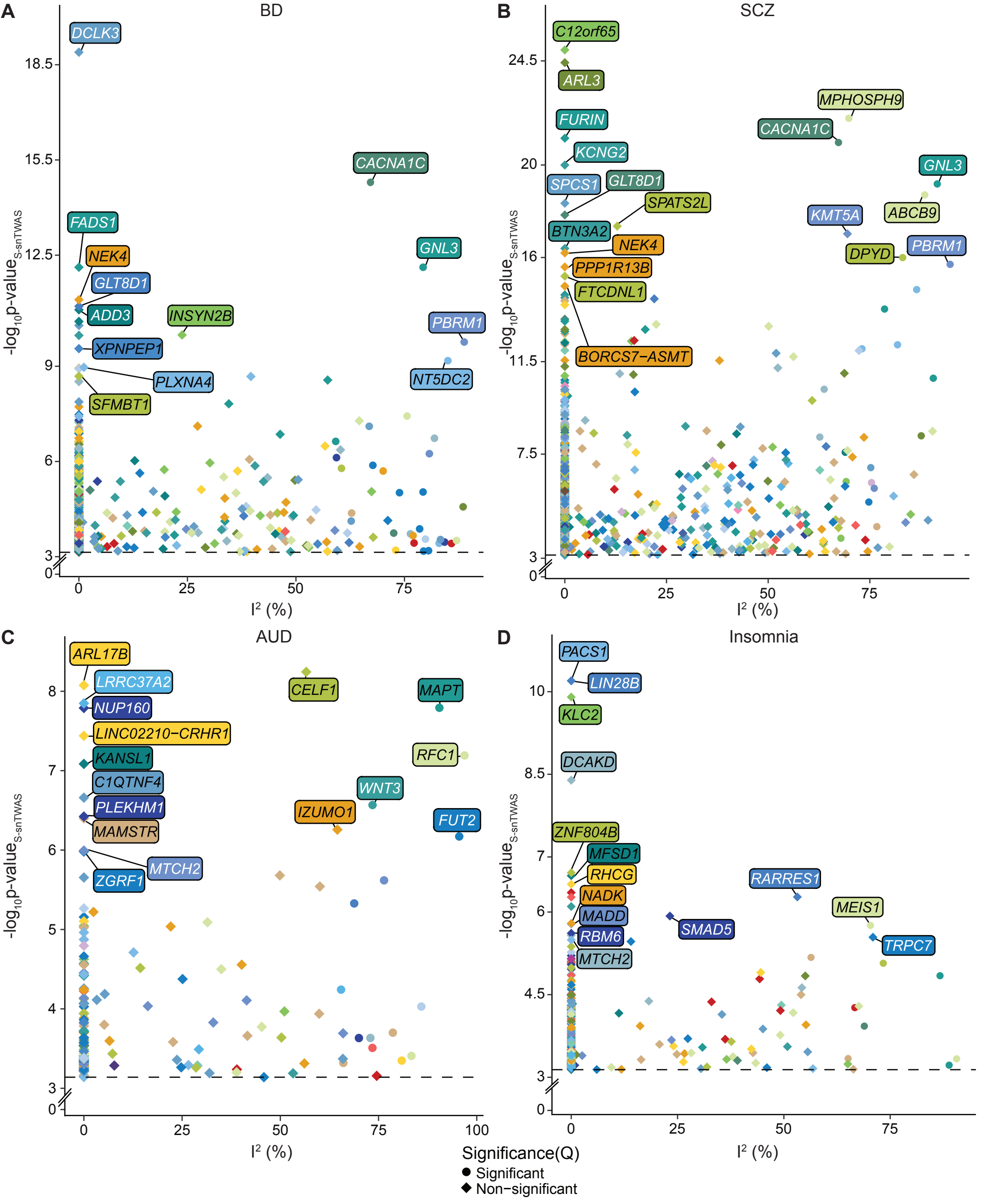

A
BD
DCLK3
CACNA1C
FADS1
NEK4
GLT8D1
ADD3
INSYN2B
GNL3
PBRM1
XPNPEP1
PLXNA4
NT5DC2
SFMBT1
-log10p-value S-snTWAS
I² (%)
B
SCZ
C12orf65
ARL3
MPHOSPH9
FURIN
CACNA1C
KCNG2
GNL3
SPCS1
GLT8D1
SPATS2L
ABCB9
KMT5A
BTN3A2
NEK4
DPYD
PBRM1
PPP1R13B
FTCDNL1
BORCS7−ASMT
C
AUD
ARL17B
LRRC37A2
CELF1
MAPT
NUP160
LINC02210−CRHR1
RFC1
KANSL1
C1QTNF4
WNT3
PLEKHM1
IZUMO1
FUT2
MAMSTR
MTCH2
ZGRF1
D
Insomnia
PACS1
LIN28B
KLC2
DCAKD
ZNF804B
MFSD1
RHCG
RARRES1
NADK
MEIS1
MADD
SMAD5
RBM6
TRPC7
MTCH2
Significance(Q)
Significant
Non−significant
