# Supplementary material for: Single-nucleus transcriptome-wide association study of human brain disorders
Source: Nature. 2026 Sep 23;657(8133):1016–26. doi: 10.1038/s41586-026-10836-6 (PMC13600940; doi:10.1038/s41586-026-10836-6)
Supplement: Supplementary file 5 [file 41586_2026_10836_MOESM4_ESM.zip › 2024-09-19723C-s4/FigS10_part2.pdf]

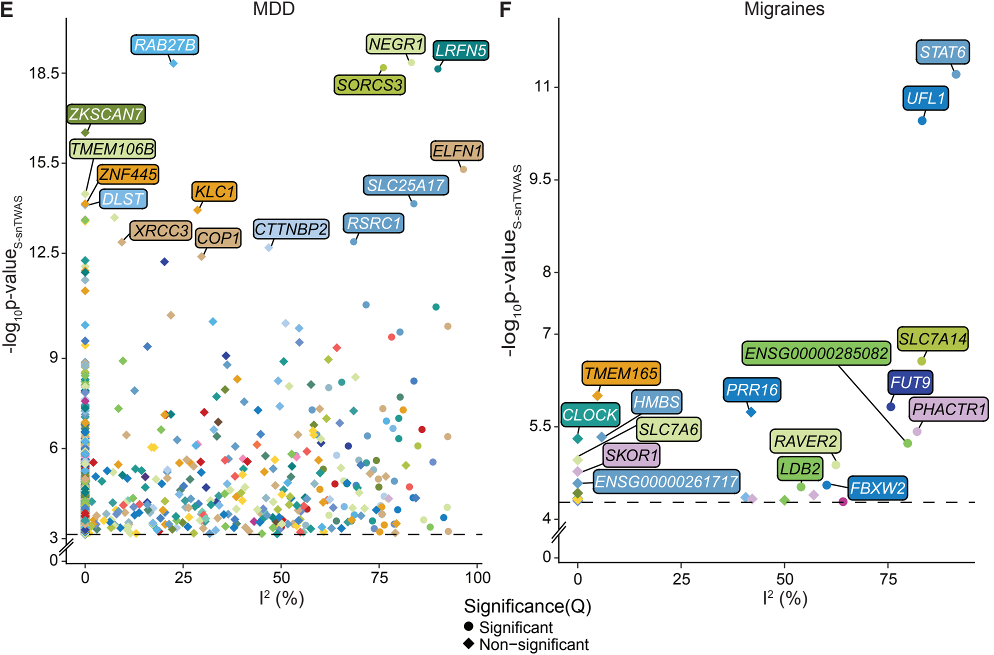
E
MDD
-log10p-value S-snTWAS
I² (%)
RAB27B
NEGR1
LRFN5
SORCS3
ZKSCAN7
TMEM106B
ZNF445
DLST
KLC1
ELFN1
SLC25A17
XRCC3
COP1
CTTNBP2
RSRC1
F
Migraines
STAT6
UFL1
SLC7A14
ENSG00000285082
TMEM165
PRR16
FUT9
HMBS
CLOCK
PHACTR1
SLC7A6
RAVER2
SKOR1
LDB2
ENSG00000261717
FBXW2
Significance(Q)
Significant
Non−significant
