# Supplementary material for: Single-nucleus transcriptome-wide association study of human brain disorders
Source: Nature. 2026 Sep 23;657(8133):1016–26. doi: 10.1038/s41586-026-10836-6 (PMC13600940; doi:10.1038/s41586-026-10836-6)
Supplement: Supplementary file 5 [file 41586_2026_10836_MOESM4_ESM.zip › 2024-09-19723C-s4/FigS11.pdf]

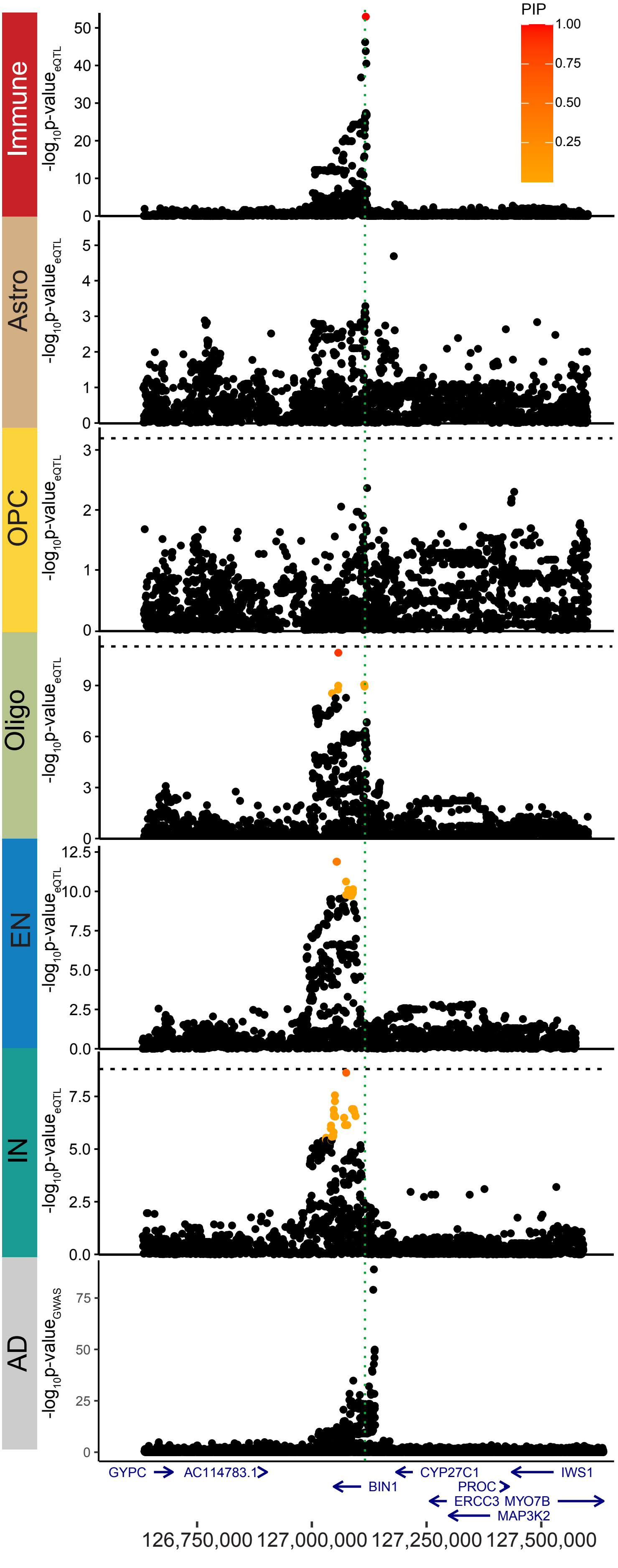

Immune
Astro
OPC
Oligo
EN
IN
AD
PIP
1.00
0.75
0.50
0.25
-log10p-valueeQTL
-log10p-valueGWAS
GYPC
AC114783.1
BIN1
CYP27C1
PROC
ERCC3 MYO7B
MAP3K2
IWS1
126,750,000
127,000,000
127,250,000
127,500,000
