# Supplementary material for: Single-nucleus transcriptome-wide association study of human brain disorders
Source: Nature. 2026 Sep 23;657(8133):1016–26. doi: 10.1038/s41586-026-10836-6 (PMC13600940; doi:10.1038/s41586-026-10836-6)
Supplement: Supplementary file 5 [file 41586_2026_10836_MOESM4_ESM.zip › 2024-09-19723C-s4/FigS12.pdf]

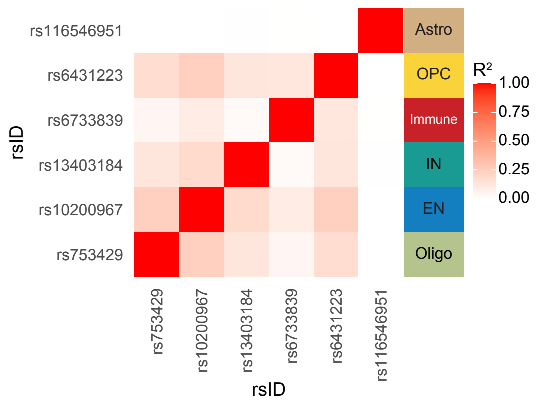

rs116546951
rs6431223
rs6733839
rs13403184
rs10200967
rs753429
rsID
Astro
OPC
Immune
IN
EN
Oligo
R^2
1.00
0.75
0.50
0.25
0.00
rs753429
rs10200967
rs13403184
rs6733839
rs6431223
rs116546951
rsID
