# Supplementary material for: Single-nucleus transcriptome-wide association study of human brain disorders
Source: Nature. 2026 Sep 23;657(8133):1016–26. doi: 10.1038/s41586-026-10836-6 (PMC13600940; doi:10.1038/s41586-026-10836-6)
Supplement: Supplementary file 5 [file 41586_2026_10836_MOESM4_ESM.zip › 2024-09-19723C-s4/FigS13_part1.pdf]

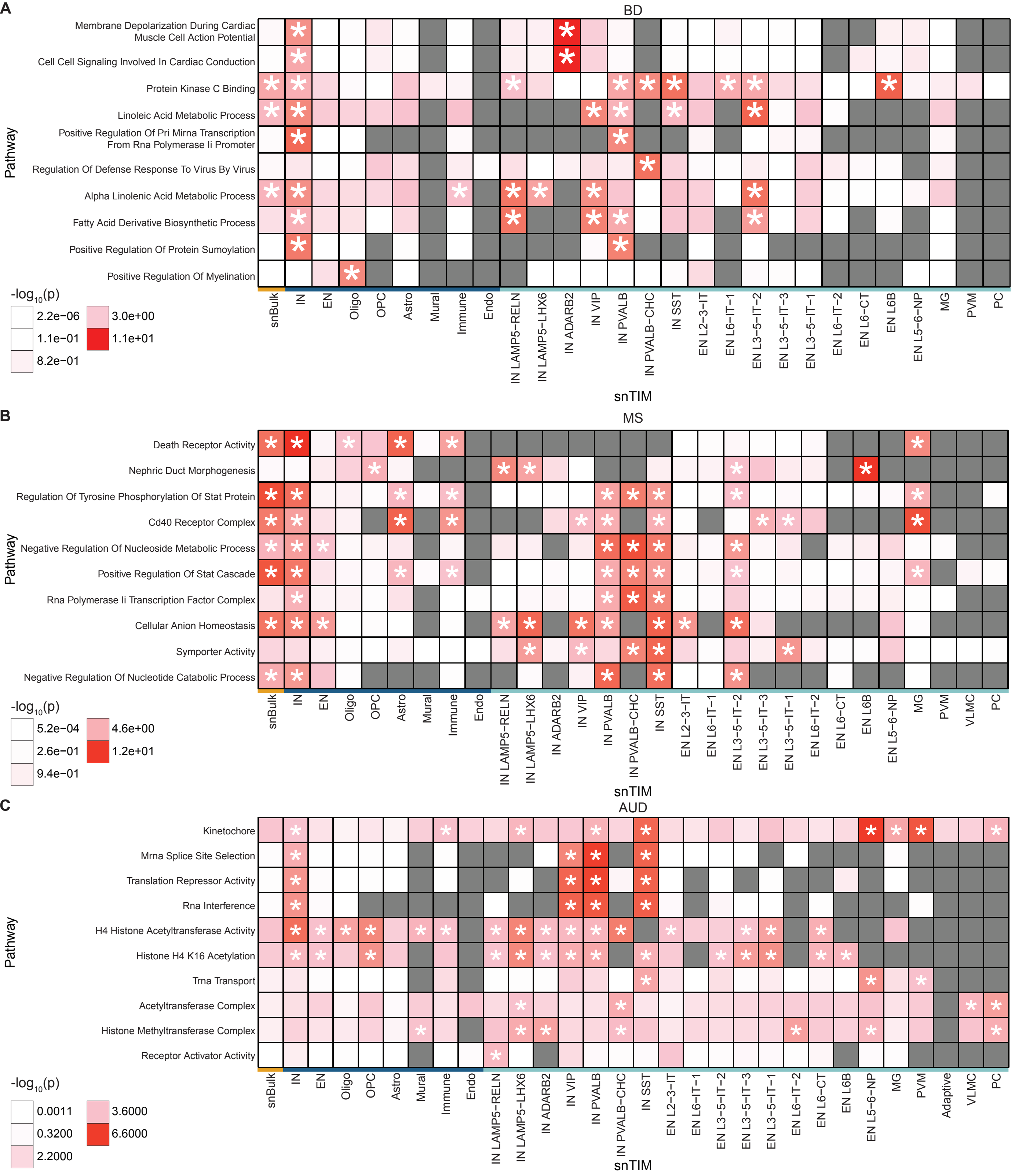
A
BD
Membrane Depolarization During Cardiac Muscle Cell Action Potential
Cell Cell Signaling Involved In Cardiac Conduction
Protein Kinase C Binding
Linoleic Acid Metabolic Process
Positive Regulation Of Pri Mirna Transcription From Rna Polymerase Ii Promoter
Regulation Of Defense Response To Virus By Virus
Alpha Linolenic Acid Metabolic Process
Fatty Acid Derivative Biosynthetic Process
Positive Regulation Of Protein Sumoylation
Positive Regulation Of Myelination
Pathway
-log10(p)
2.2e−06
1.1e−01
8.2e−01
3.0e+00
1.1e+01
snBulk
IN
EN
Oligo
OPC
Astro
Mural
Immune
Endo
IN LAMP5−RELN
IN LAMP5−LHX6
IN ADARB2
IN VIP
IN PVALB
IN PVALB−CHC
IN SST
EN L2−3−IT
EN L6−IT−1
EN L3−5−IT−2
EN L3−5−IT−3
EN L3−5−IT−1
EN L6−IT−2
EN L6−CT
EN L6B
EN L5−6−NP
MG
PVM
PC
snTIM
B
MS
Death Receptor Activity
Nephric Duct Morphogenesis
Regulation Of Tyrosine Phosphorylation Of Stat Protein
Cd40 Receptor Complex
Negative Regulation Of Nucleoside Metabolic Process
Positive Regulation Of Stat Cascade
Rna Polymerase Ii Transcription Factor Complex
Cellular Anion Homeostasis
Symporter Activity
Negative Regulation Of Nucleotide Catabolic Process
Pathway
-log10(p)
5.2e−04
2.6e−01
9.4e−01
4.6e+00
1.2e+01
snBulk
IN
EN
Oligo
OPC
Astro
Mural
Immune
Endo
IN LAMP5−RELN
IN LAMP5−LHX6
IN ADARB2
IN VIP
IN PVALB
IN PVALB−CHC
IN SST
EN L2−3−IT
EN L6−IT−1
EN L3−5−IT−2
EN L3−5−IT−3
EN L3−5−IT−1
EN L6−IT−2
EN L6−CT
EN L6B
EN L5−6−NP
MG
PVM
VLMC
PC
snTIM
C
AUD
Kinetochore
Mrna Splice Site Selection
Translation Repressor Activity
Rna Interference
H4 Histone Acetyltransferase Activity
Histone H4 K16 Acetylation
Trna Transport
Acetyltransferase Complex
Histone Methyltransferase Complex
Receptor Activator Activity
Pathway
-log10(p)
0.0011
0.3200
2.2000
3.6000
6.6000
snBulk
IN
EN
Oligo
OPC
Astro
Mural
Immune
Endo
IN LAMP5−RELN
IN LAMP5−LHX6
IN ADARB2
IN VIP
IN PVALB
IN PVALB−CHC
IN SST
EN L2−3−IT
EN L6−IT−1
EN L3−5−IT−2
EN L3−5−IT−3
EN L3−5−IT−1
EN L6−IT−2
EN L6−CT
EN L6B
EN L5−6−NP
MG
PVM
Adaptive
VLMC
PC
snTIM
