# Supplementary material for: Single-nucleus transcriptome-wide association study of human brain disorders
Source: Nature. 2026 Sep 23;657(8133):1016–26. doi: 10.1038/s41586-026-10836-6 (PMC13600940; doi:10.1038/s41586-026-10836-6)
Supplement: Supplementary file 5 [file 41586_2026_10836_MOESM4_ESM.zip › 2024-09-19723C-s4/FigS13_part2.pdf]

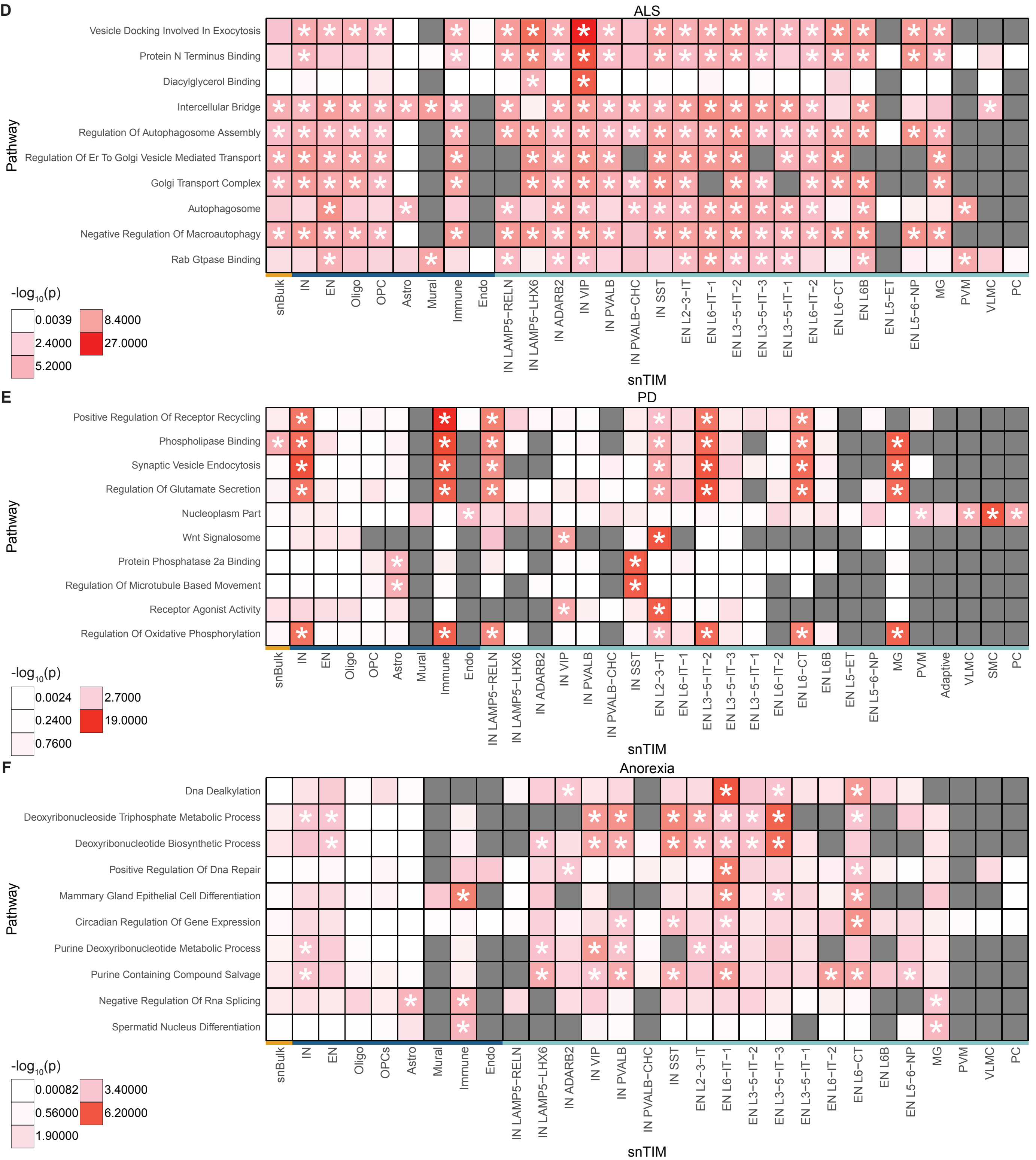
D
ALS
Vesicle Docking Involved In Exocytosis
Protein N Terminus Binding
Diacylglycerol Binding
Intercellular Bridge
Regulation Of Autophagosome Assembly
Regulation Of Er To Golgi Vesicle Mediated Transport
Golgi Transport Complex
Autophagosome
Negative Regulation Of Macroautophagy
Rab Gtpase Binding
Pathway
snBulk IN EN Oligo OPC Astro Mural Immune Endo IN LAMP5−RELN IN LAMP5−LHX6 IN ADARB2 IN VIP IN PVALB IN PVALB−CHC IN SST EN L2−3−IT EN L6−IT−1 EN L3−5−IT−2 EN L3−5−IT−3 EN L3−5−IT−1 EN L6−IT−2 EN L6−CT EN L6B EN L5−ET EN L5−6−NP MG PVM VLMC PC
snTIM
-log10(p)
0.0039
2.4000
5.2000
8.4000
27.0000
E
PD
Positive Regulation Of Receptor Recycling
Phospholipase Binding
Synaptic Vesicle Endocytosis
Regulation Of Glutamate Secretion
Nucleoplasm Part
Wnt Signalosome
Protein Phosphatase 2a Binding
Regulation Of Microtubule Based Movement
Receptor Agonist Activity
Regulation Of Oxidative Phosphorylation
Pathway
snBulk IN EN Oligo OPC Astro Mural Immune Endo IN LAMP5−RELN IN LAMP5−LHX6 IN ADARB2 IN VIP IN PVALB IN PVALB−CHC IN SST EN L2−3−IT EN L6−IT−1 EN L3−5−IT−2 EN L3−5−IT−3 EN L3−5−IT−1 EN L6−IT−2 EN L6−CT EN L6B EN L5−ET EN L5−6−NP MG PVM Adaptive VLMC SMC PC
snTIM
-log10(p)
0.0024
0.2400
0.7600
2.7000
19.0000
F
Anorexia
Dna Dealkylation
Deoxyribonucleoside Triphosphate Metabolic Process
Deoxyribonucleotide Biosynthetic Process
Positive Regulation Of Dna Repair
Mammary Gland Epithelial Cell Differentiation
Circadian Regulation Of Gene Expression
Purine Deoxyribonucleotide Metabolic Process
Purine Containing Compound Salvage
Negative Regulation Of Rna Splicing
Spermatid Nucleus Differentiation
Pathway
snBulk IN EN Oligo OPCs Astro Mural Immune Endo IN LAMP5−RELN IN LAMP5−LHX6 IN ADARB2 IN VIP IN PVALB IN PVALB−CHC IN SST EN L2−3−IT EN L6−IT−1 EN L3−5−IT−2 EN L3−5−IT−3 EN L3−5−IT−1 EN L6−IT−2 EN L6−CT EN L6B EN L5−6−NP MG PVM VLMC PC
snTIM
-log10(p)
0.00082
0.56000
1.90000
3.40000
6.20000
