# Supplementary material for: Single-nucleus transcriptome-wide association study of human brain disorders
Source: Nature. 2026 Sep 23;657(8133):1016–26. doi: 10.1038/s41586-026-10836-6 (PMC13600940; doi:10.1038/s41586-026-10836-6)
Supplement: Supplementary file 5 [file 41586_2026_10836_MOESM4_ESM.zip › 2024-09-19723C-s4/FigS13_part3.pdf]

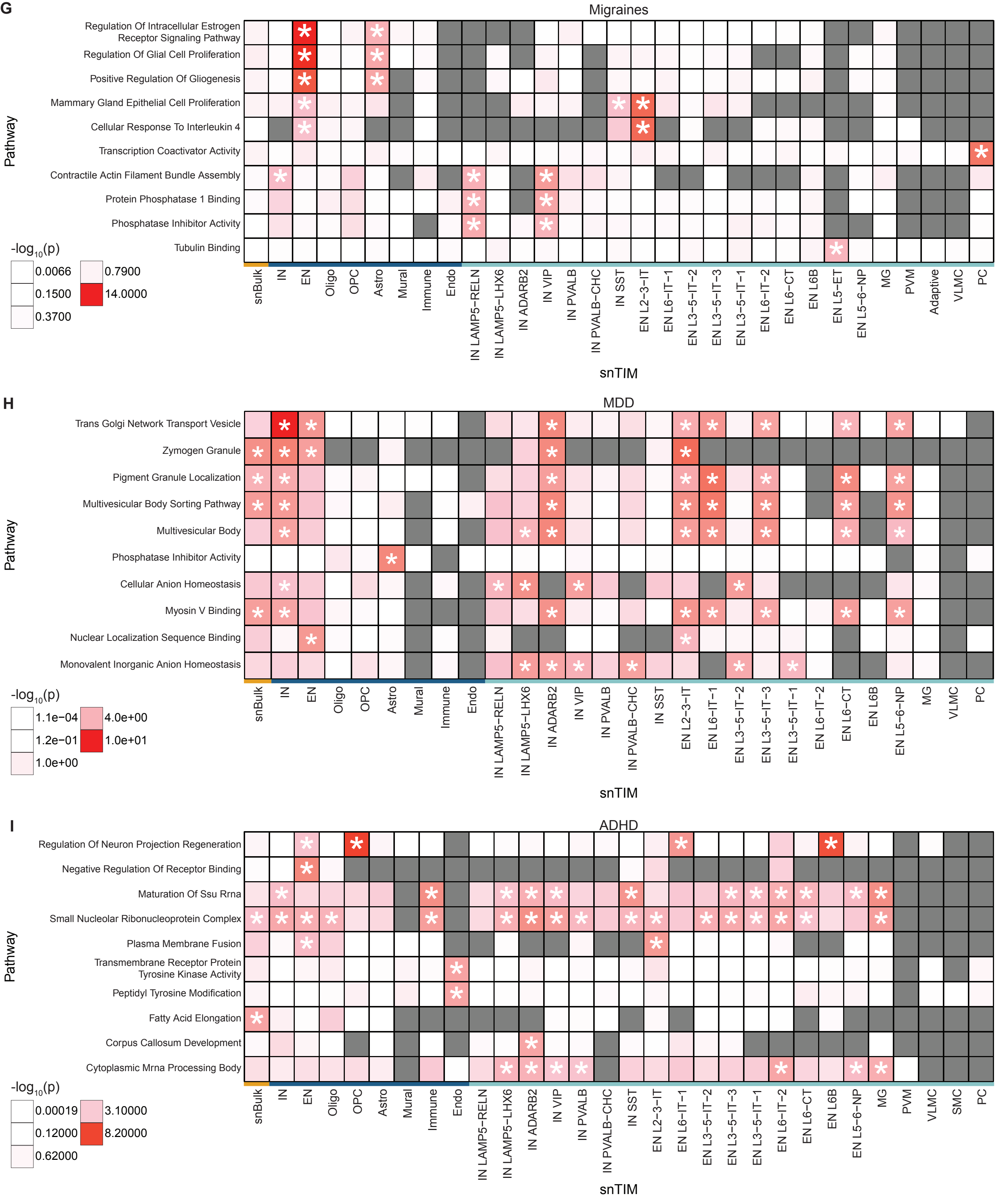
G
Migraines
Pathway
Regulation Of Intracellular Estrogen Receptor Signaling Pathway
Regulation Of Glial Cell Proliferation
Positive Regulation Of Gliogenesis
Mammary Gland Epithelial Cell Proliferation
Cellular Response To Interleukin 4
Transcription Coactivator Activity
Contractile Actin Filament Bundle Assembly
Protein Phosphatase 1 Binding
Phosphatase Inhibitor Activity
Tubulin Binding
-log10(p)
0.0066
0.1500
0.3700
0.7900
14.0000
snBulk
IN
EN
Oligo
OPC
Astro
Mural
Immune
Endo
IN LAMP5−RELN
IN LAMP5−LHX6
IN ADARB2
IN VIP
IN PVALB
IN PVALB−CHC
IN SST
EN L2−3−IT
EN L6−IT−1
EN L3−5−IT−2
EN L3−5−IT−3
EN L3−5−IT−1
EN L6−IT−2
EN L6−CT
EN L6B
EN L5−ET
EN L5−6−NP
MG
PVM
Adaptive
VLMC
PC
snTIM
H
MDD
Pathway
Trans Golgi Network Transport Vesicle
Zymogen Granule
Pigment Granule Localization
Multivesicular Body Sorting Pathway
Multivesicular Body
Phosphatase Inhibitor Activity
Cellular Anion Homeostasis
Myosin V Binding
Nuclear Localization Sequence Binding
Monovalent Inorganic Anion Homeostasis
-log10(p)
1.1e−04
1.2e−01
1.0e+00
4.0e+00
1.0e+01
snBulk
IN
EN
Oligo
OPC
Astro
Mural
Immune
Endo
IN LAMP5−RELN
IN LAMP5−LHX6
IN ADARB2
IN VIP
IN PVALB
IN PVALB−CHC
IN SST
EN L2−3−IT
EN L6−IT−1
EN L3−5−IT−2
EN L3−5−IT−3
EN L3−5−IT−1
EN L6−IT−2
EN L6−CT
EN L6B
EN L5−6−NP
MG
VLMC
PC
snTIM
I
ADHD
Pathway
Regulation Of Neuron Projection Regeneration
Negative Regulation Of Receptor Binding
Maturation Of Ssu Rrna
Small Nucleolar Ribonucleoprotein Complex
Plasma Membrane Fusion
Transmembrane Receptor Protein Tyrosine Kinase Activity
Peptidyl Tyrosine Modification
Fatty Acid Elongation
Corpus Callosum Development
Cytoplasmic Mrna Processing Body
-log10(p)
0.00019
0.12000
0.62000
3.10000
8.20000
snBulk
IN
EN
Oligo
OPC
Astro
Mural
Immune
Endo
IN LAMP5−RELN
IN LAMP5−LHX6
IN ADARB2
IN VIP
IN PVALB
IN PVALB−CHC
IN SST
EN L2−3−IT
EN L6−IT−1
EN L3−5−IT−2
EN L3−5−IT−3
EN L3−5−IT−1
EN L6−IT−2
EN L6−CT
EN L6B
EN L5−6−NP
MG
PVM
VLMC
SMC
PC
snTIM
