# Supplementary material for: Single-nucleus transcriptome-wide association study of human brain disorders
Source: Nature. 2026 Sep 23;657(8133):1016–26. doi: 10.1038/s41586-026-10836-6 (PMC13600940; doi:10.1038/s41586-026-10836-6)
Supplement: Supplementary file 5 [file 41586_2026_10836_MOESM4_ESM.zip › 2024-09-19723C-s4/FigS13_part4.pdf]

J

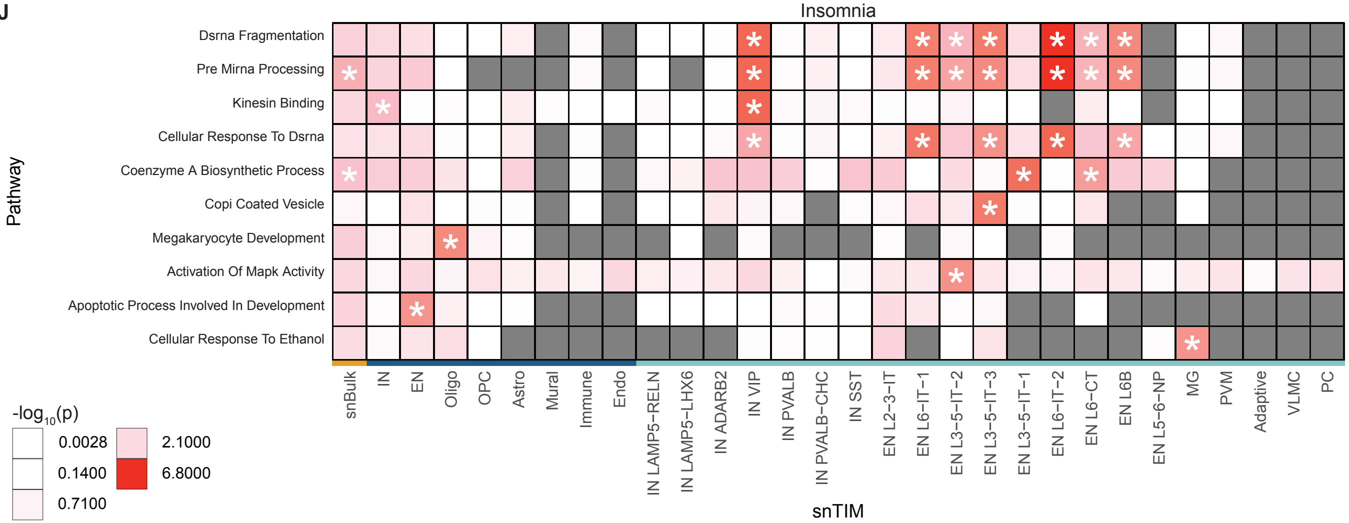

Insomnia
Pathway
Dsrna Fragmentation
Pre Mirna Processing
Kinesin Binding
Cellular Response To Dsrna
Coenzyme A Biosynthetic Process
Copi Coated Vesicle
Megakaryocyte Development
Activation Of Mapk Activity
Apoptotic Process Involved In Development
Cellular Response To Ethanol
snBulk
IN
EN
Oligo
OPC
Astro
Mural
Immune
Endo
IN LAMP5−RELN
IN LAMP5−LHX6
IN ADARB2
IN VIP
IN PVALB
IN PVALB−CHC
IN SST
EN L2−3−IT
EN L6−IT−1
EN L3−5−IT−2
EN L3−5−IT−3
EN L3−5−IT−1
EN L6−IT−2
EN L6−CT
EN L6B
EN L5−6−NP
MG
PVM
Adaptive
VLMC
PC
snTIM
-log10(p)
0.0028
0.1400
0.7100
2.1000
6.8000
