# Supplementary material for: Single-nucleus transcriptome-wide association study of human brain disorders
Source: Nature. 2026 Sep 23;657(8133):1016–26. doi: 10.1038/s41586-026-10836-6 (PMC13600940; doi:10.1038/s41586-026-10836-6)
Supplement: Supplementary file 5 [file 41586_2026_10836_MOESM4_ESM.zip › 2024-09-19723C-s4/FigS14.pdf]

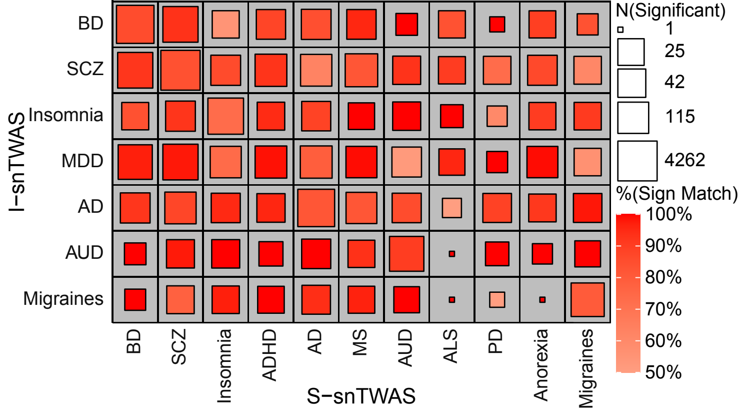

I−snTWAS
BD
SCZ
Insomnia
MDD
AD
AUD
Migraines
BD
SCZ
Insomnia
ADHD
AD
MS
AUD
ALS
PD
Anorexia
Migraines
S−snTWAS
N(Significant)
1
25
42
115
4262
%(Sign Match)
100%
90%
80%
70%
60%
50%
