# Supplementary material for: Single-nucleus transcriptome-wide association study of human brain disorders
Source: Nature. 2026 Sep 23;657(8133):1016–26. doi: 10.1038/s41586-026-10836-6 (PMC13600940; doi:10.1038/s41586-026-10836-6)
Supplement: Supplementary file 5 [file 41586_2026_10836_MOESM4_ESM.zip › 2024-09-19723C-s4/FigS15.pdf]

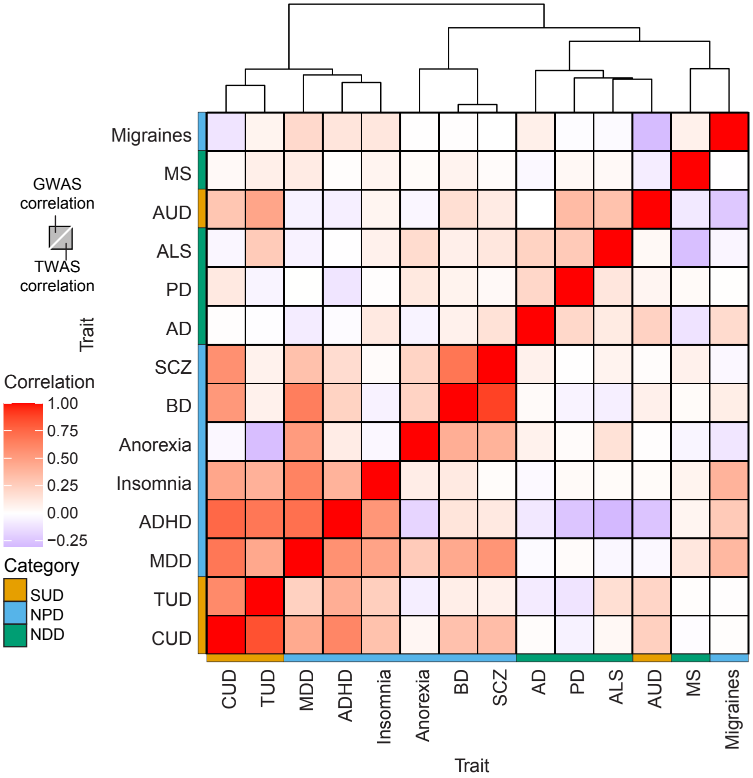

GWAS correlation
TWAS correlation
Trait
Correlation
1.00
0.75
0.50
0.25
0.00
−0.25
Category
SUD
NPD
NDD
Migraines
MS
AUD
ALS
PD
AD
SCZ
BD
Anorexia
Insomnia
ADHD
MDD
TUD
CUD
CUD
TUD
MDD
ADHD
Insomnia
Anorexia
BD
SCZ
AD
PD
ALS
AUD
MS
Migraines
Trait
