# Supplementary material for: Single-nucleus transcriptome-wide association study of human brain disorders
Source: Nature. 2026 Sep 23;657(8133):1016–26. doi: 10.1038/s41586-026-10836-6 (PMC13600940; doi:10.1038/s41586-026-10836-6)
Supplement: Supplementary file 5 [file 41586_2026_10836_MOESM4_ESM.zip › 2024-09-19723C-s4/FigS18.pdf]

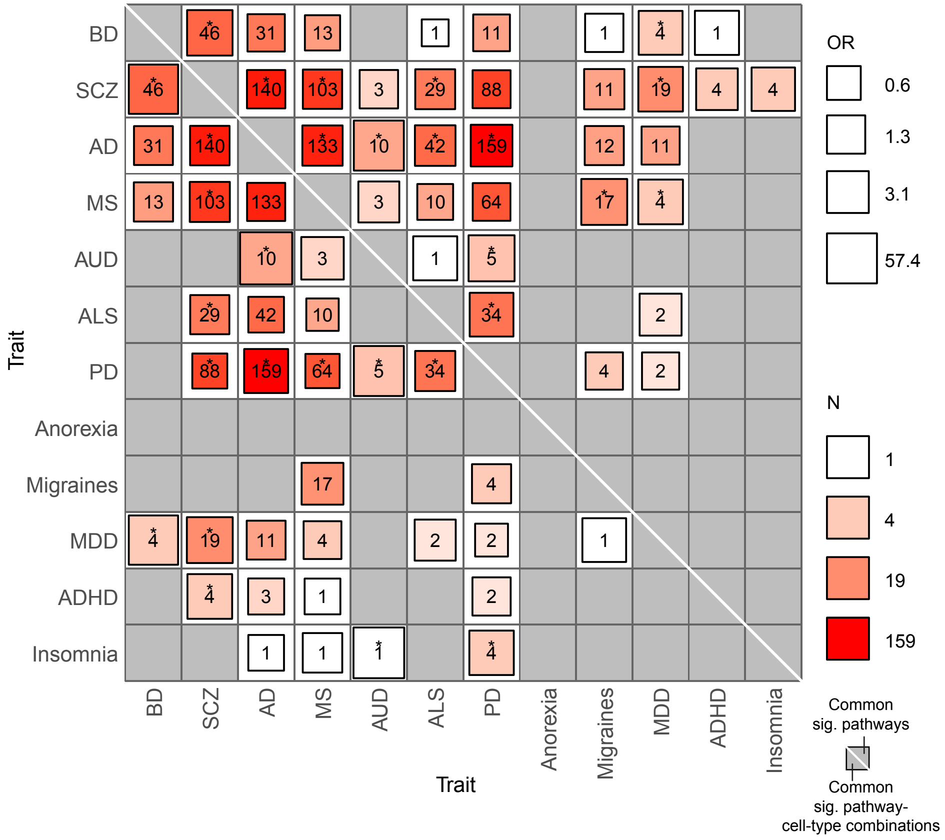
BD
SCZ
AD
MS
AUD
ALS
PD
Anorexia
Migraines
MDD
ADHD
Insomnia
Trait
Trait
OR
0.6
1.3
3.1
57.4
N
1
4
19
159
Common sig. pathways
Common sig. pathway-cell-type combinations
