# Supplementary material for: Single-nucleus transcriptome-wide association study of human brain disorders
Source: Nature. 2026 Sep 23;657(8133):1016–26. doi: 10.1038/s41586-026-10836-6 (PMC13600940; doi:10.1038/s41586-026-10836-6)
Supplement: Supplementary file 5 [file 41586_2026_10836_MOESM4_ESM.zip › 2024-09-19723C-s4/FigS19.pdf]

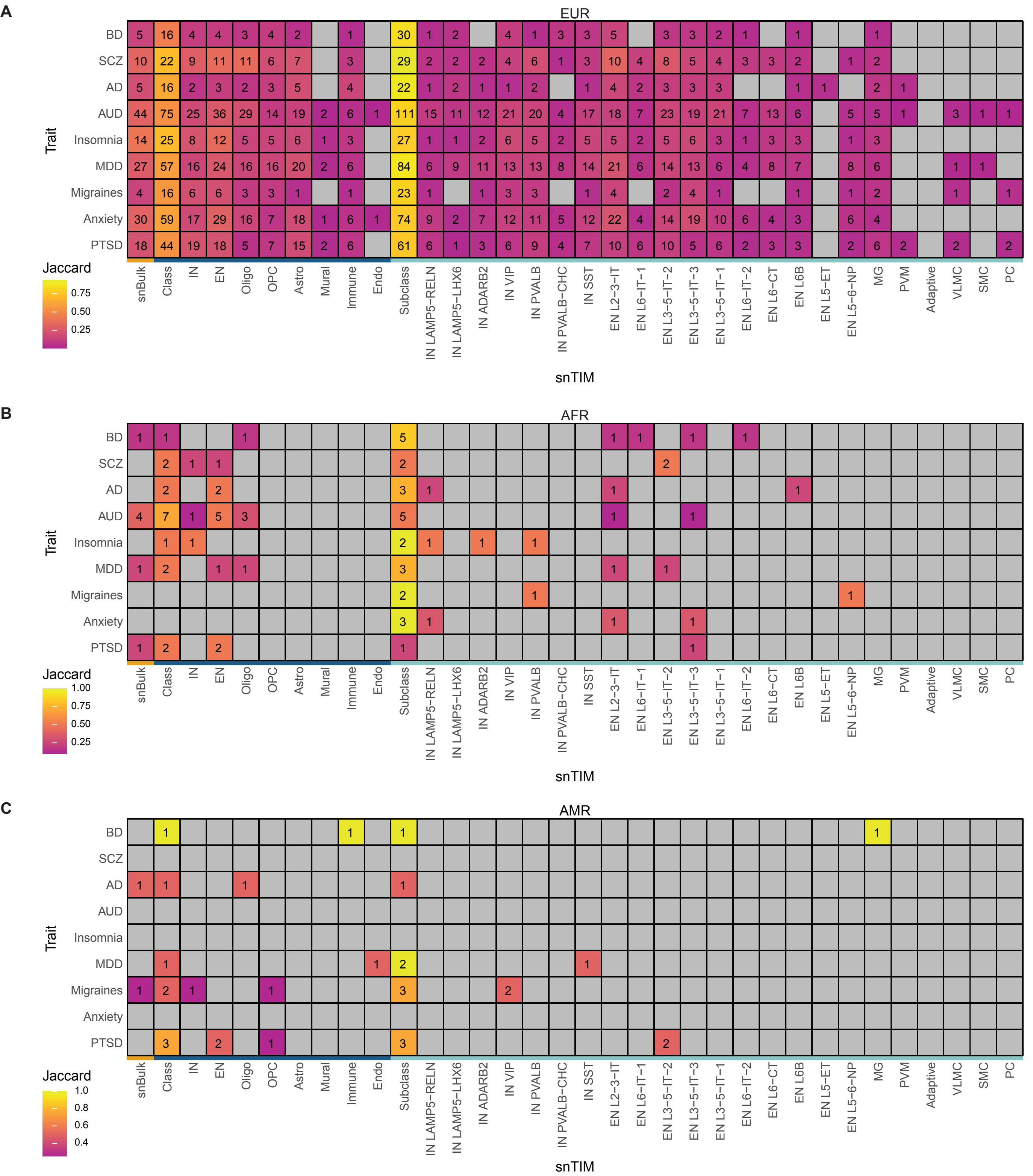
A
EUR
Trait
BD
SCZ
AD
AUD
Insomnia
MDD
Migraines
Anxiety
PTSD
snBulk
Class
IN
EN
Oligo
OPC
Astro
Mural
Immune
Endo
Subclass
IN LAMP5−RELN
IN LAMP5−LHX6
IN ADARB2
IN VIP
IN PVALB
IN PVALB−CHC
IN SST
EN L2−3−IT
EN L6−IT−1
EN L3−5−IT−2
EN L3−5−IT−3
EN L3−5−IT−1
EN L6−IT−2
EN L6−CT
EN L6B
EN L5−ET
EN L5−6−NP
MG
PVM
Adaptive
VLMC
SMC
PC
Jaccard
0.75
0.50
0.25
snTIM
B
AFR
Jaccard
1.00
0.75
0.50
0.25
snTIM
C
AMR
Jaccard
1.0
0.8
0.6
0.4
snTIM
