# Supplementary material for: Single-nucleus transcriptome-wide association study of human brain disorders
Source: Nature. 2026 Sep 23;657(8133):1016–26. doi: 10.1038/s41586-026-10836-6 (PMC13600940; doi:10.1038/s41586-026-10836-6)
Supplement: Supplementary file 5 [file 41586_2026_10836_MOESM4_ESM.zip › 2024-09-19723C-s4/FigS2.pdf]

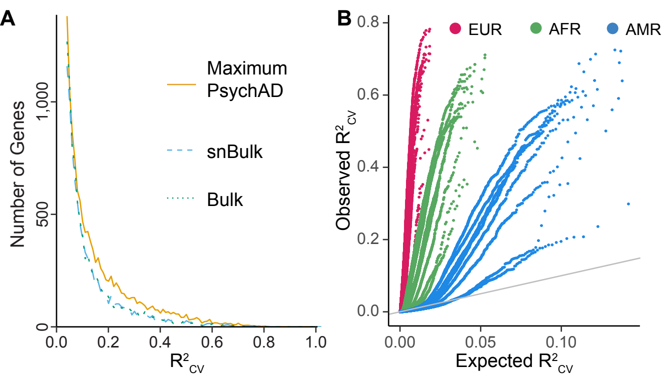
A
Number of Genes
1,000
500
0
0.0
0.2
0.4
0.6
0.8
1.0
R^2_{CV}
Maximum PsychAD
snBulk
Bulk
B
EUR
AFR
AMR
Observed R^2_{CV}
0.8
0.6
0.4
0.2
0.0
0.00
0.05
0.10
Expected R^2_{CV}
