# Supplementary material for: Single-nucleus transcriptome-wide association study of human brain disorders
Source: Nature. 2026 Sep 23;657(8133):1016–26. doi: 10.1038/s41586-026-10836-6 (PMC13600940; doi:10.1038/s41586-026-10836-6)
Supplement: Supplementary file 5 [file 41586_2026_10836_MOESM4_ESM.zip › 2024-09-19723C-s4/FigS20.pdf]

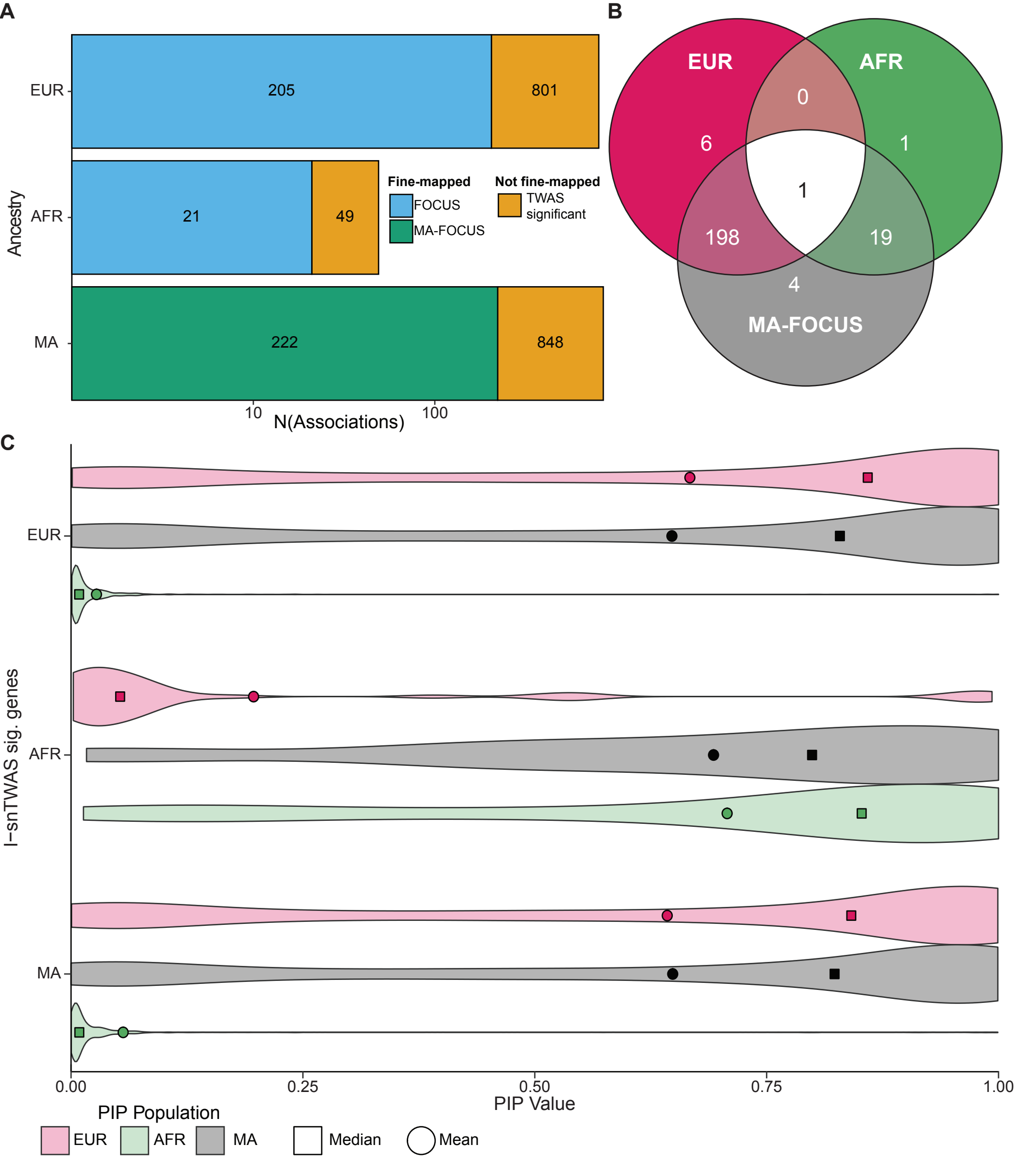

A
EUR
AFR
MA
205
801
21
49
222
848
Ancestry
Fine-mapped
FOCUS
MA-FOCUS
Not fine-mapped
TWAS significant
10
100
N(Associations)
B
EUR
AFR
MA-FOCUS
0
6
1
1
198
19
4
C
I−snTWAS sig. genes
EUR
AFR
MA
0.00
0.25
0.50
0.75
1.00
PIP Value
PIP Population
EUR
AFR
MA
Median
Mean
