# Supplementary material for: Single-nucleus transcriptome-wide association study of human brain disorders
Source: Nature. 2026 Sep 23;657(8133):1016–26. doi: 10.1038/s41586-026-10836-6 (PMC13600940; doi:10.1038/s41586-026-10836-6)
Supplement: Supplementary file 5 [file 41586_2026_10836_MOESM4_ESM.zip › 2024-09-19723C-s4/FigS21_part1.pdf]

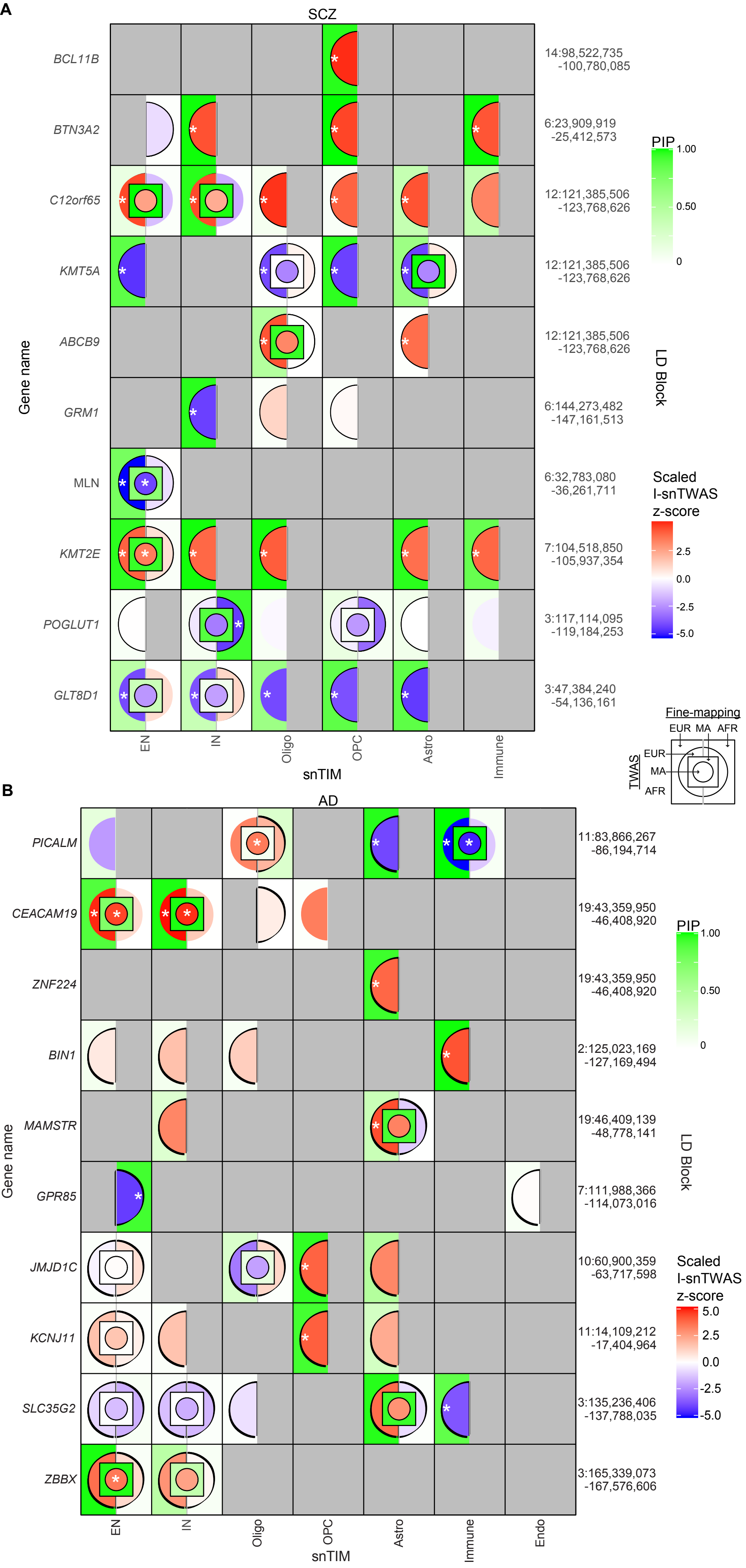
A
SCZ
BCL11B
BTN3A2
C12orf65
KMT5A
ABCB9
GRM1
MLN
KMT2E
POGLUT1
GLT8D1
Gene name
14:98,522,735 -100,780,085
6:23,909,919 -25,412,573
12:121,385,506 -123,768,626
12:121,385,506 -123,768,626
12:121,385,506 -123,768,626
6:144,273,482 -147,161,513
6:32,783,080 -36,261,711
7:104,518,850 -105,937,354
3:117,114,095 -119,184,253
3:47,384,240 -54,136,161
EN
IN
Oligo
OPC
Astro
Immune
snTIM
PIP
1.00
0.50
0
LD Block
Scaled I-snTWAS z-score
2.5
0.0
-2.5
-5.0
Fine-mapping
EUR MA AFR
TWAS
EUR
MA
AFR
B
AD
PICALM
CEACAM19
ZNF224
BIN1
MAMSTR
GPR85
JMJD1C
KCNJ11
SLC35G2
ZBBX
Gene name
11:83,866,267 -86,194,714
19:43,359,950 -46,408,920
19:43,359,950 -46,408,920
2:125,023,169 -127,169,494
19:46,409,139 -48,778,141
7:111,988,366 -114,073,016
10:60,900,359 -63,717,598
11:14,109,212 -17,404,964
3:135,236,406 -137,788,035
3:165,339,073 -167,576,606
EN
IN
Oligo
OPC
Astro
Immune
Endo
snTIM
PIP
1.00
0.50
0
LD Block
Scaled I-snTWAS z-score
5.0
2.5
0.0
-2.5
-5.0
