# Supplementary material for: Single-nucleus transcriptome-wide association study of human brain disorders
Source: Nature. 2026 Sep 23;657(8133):1016–26. doi: 10.1038/s41586-026-10836-6 (PMC13600940; doi:10.1038/s41586-026-10836-6)
Supplement: Supplementary file 5 [file 41586_2026_10836_MOESM4_ESM.zip › 2024-09-19723C-s4/FigS21_part2.pdf]

C

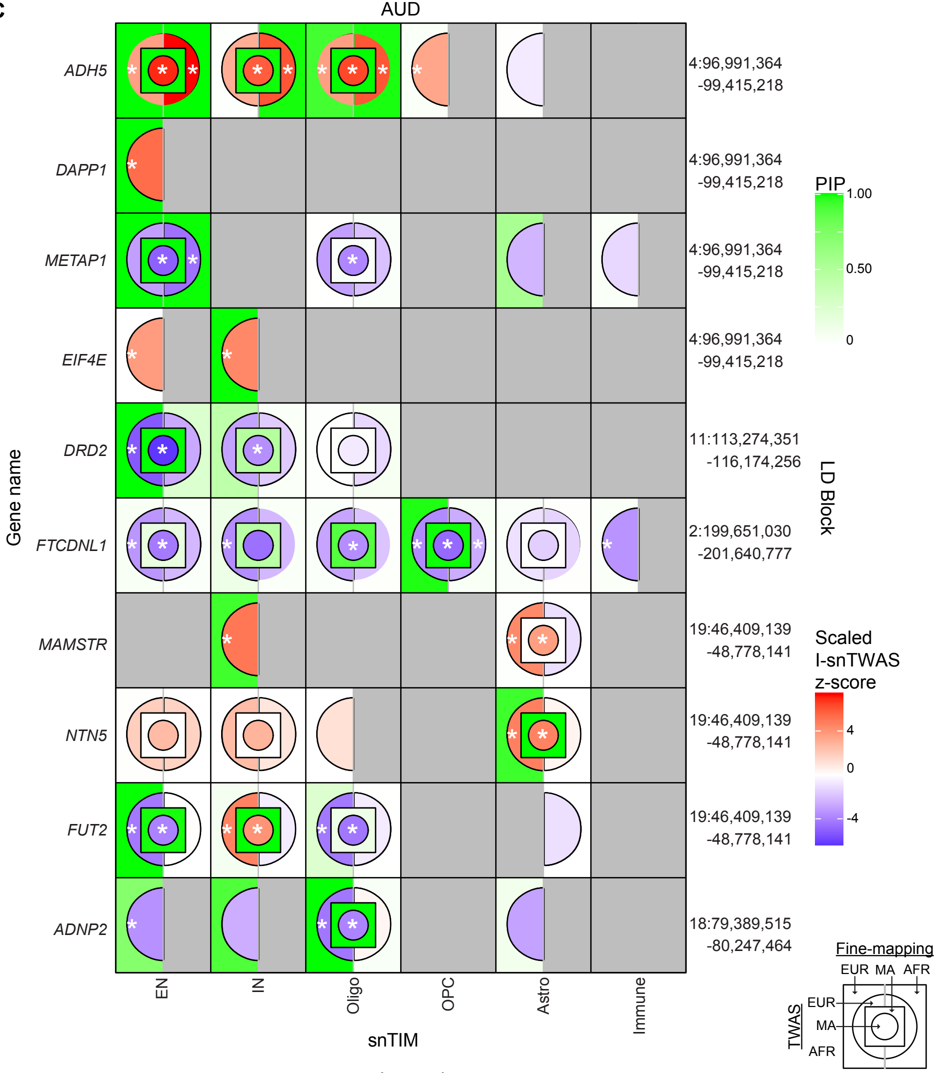
AUD
ADH5
DAPP1
METAP1
EIF4E
DRD2
FTCDNL1
MAMSTR
NTN5
FUT2
ADNP2
Gene name
4:96,991,364
-99,415,218
11:113,274,351
-116,174,256
2:199,651,030
-201,640,777
19:46,409,139
-48,778,141
18:79,389,515
-80,247,464
LD Block
EN
IN
Oligo
OPC
Astro
Immune
snTIM
PIP
1.00
0.50
0
Scaled
I-snTWAS
z-score
4
0
-4
Fine-mapping
EUR
MA
AFR
TWAS
EUR
MA
AFR

D

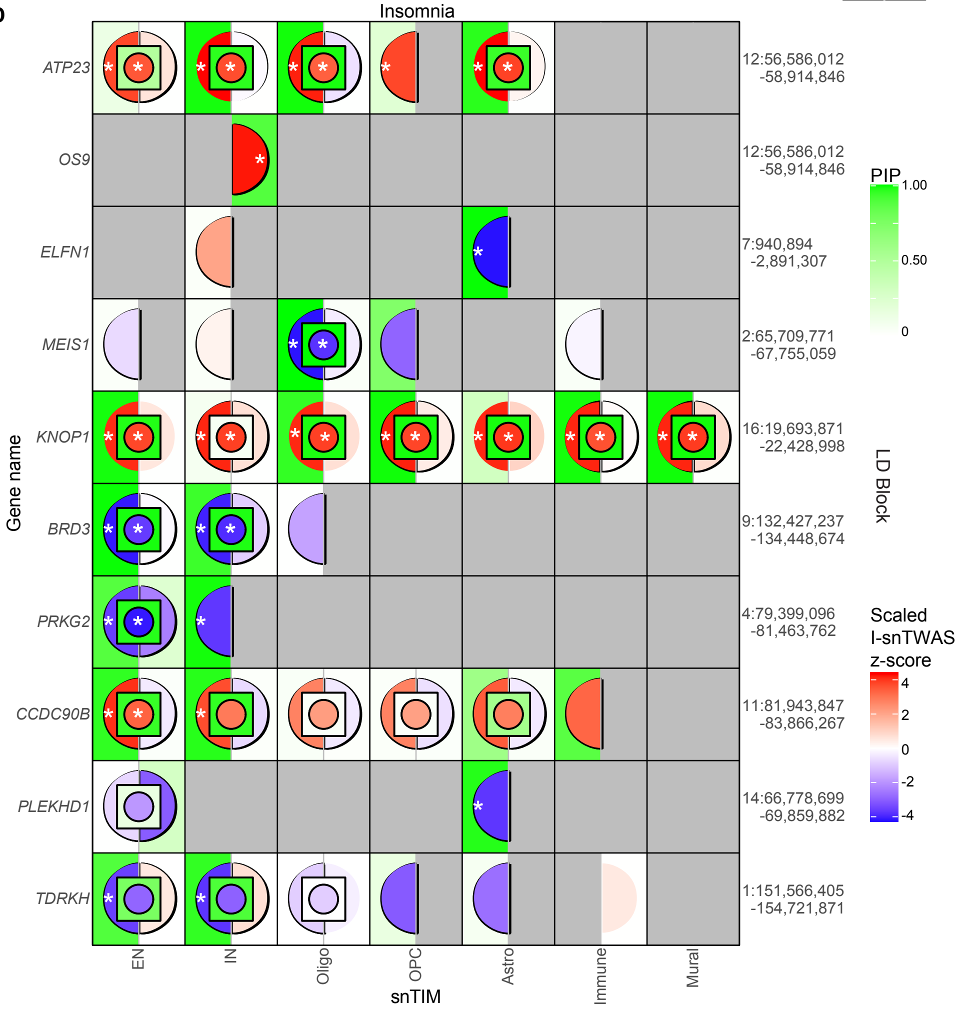
Insomnia
ATP23
OS9
ELFN1
MEIS1
KNOP1
BRD3
PRKG2
CCDC90B
PLEKHD1
TDRKH
Gene name
12:56,586,012
-58,914,846
7:940,894
-2,891,307
2:65,709,771
-67,755,059
16:19,693,871
-22,428,998
9:132,427,237
-134,448,674
4:79,399,096
-81,463,762
11:81,943,847
-83,866,267
14:66,778,699
-69,859,882
1:151,566,405
-154,721,871
LD Block
EN
IN
Oligo
OPC
Astro
Immune
Mural
snTIM
PIP
1.00
0.50
0
Scaled
I-snTWAS
z-score
4
2
0
-2
-4
