# Supplementary material for: Single-nucleus transcriptome-wide association study of human brain disorders
Source: Nature. 2026 Sep 23;657(8133):1016–26. doi: 10.1038/s41586-026-10836-6 (PMC13600940; doi:10.1038/s41586-026-10836-6)
Supplement: Supplementary file 5 [file 41586_2026_10836_MOESM4_ESM.zip › 2024-09-19723C-s4/FigS21_part3.pdf]

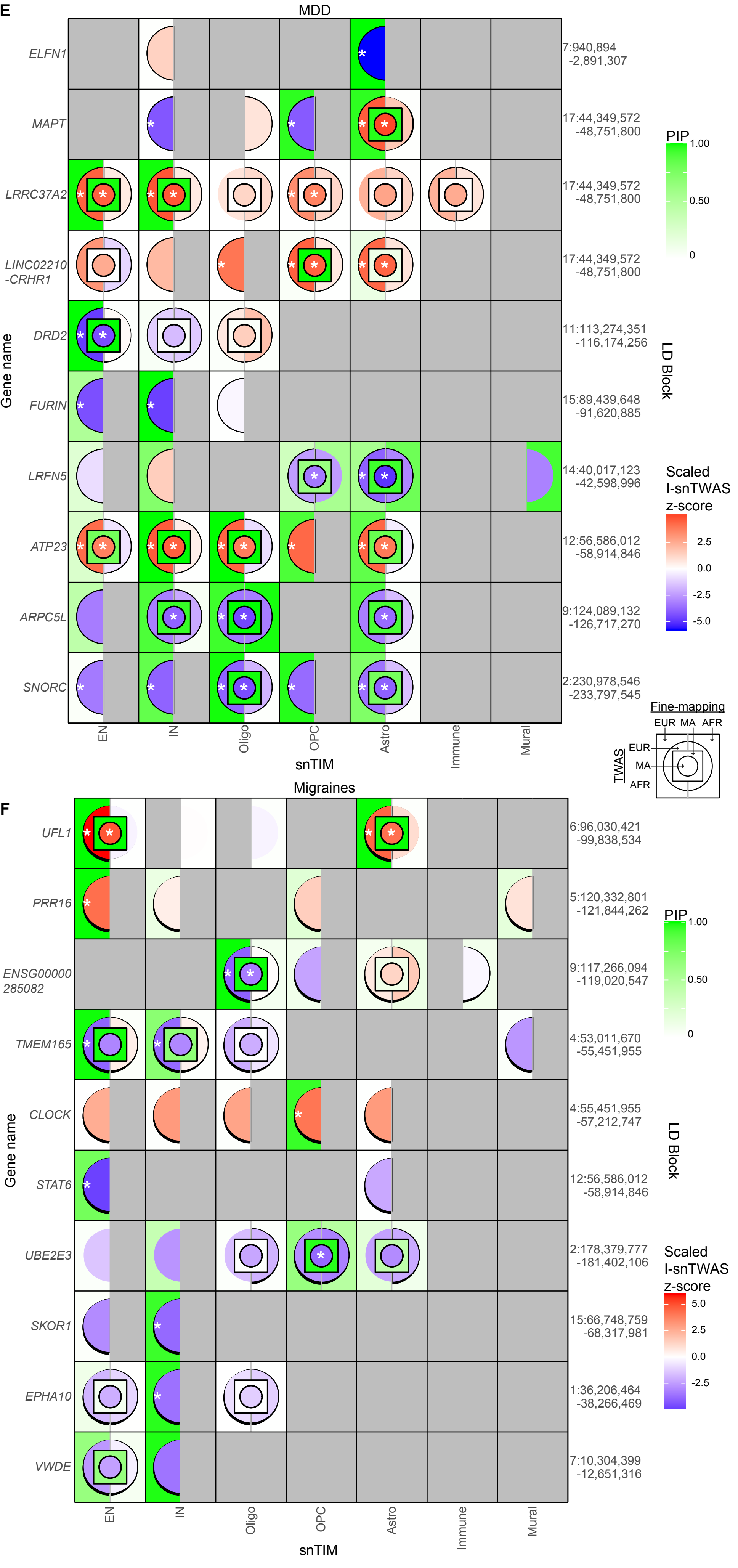
E
MDD
ELFN1
MAPT
LRRC37A2
LINC02210
-CRHR1
DRD2
FURIN
LRFN5
ATP23
ARPC5L
SNORC
Gene name
7:940,894
-2,891,307
17:44,349,572
-48,751,800
17:44,349,572
-48,751,800
17:44,349,572
-48,751,800
11:113,274,351
-116,174,256
15:89,439,648
-91,620,885
14:40,017,123
-42,598,996
12:56,586,012
-58,914,846
9:124,089,132
-126,717,270
2:230,978,546
-233,797,545
LD Block
PIP
1.00
0.50
0
Scaled
I-snTWAS
z-score
2.5
0.0
-2.5
-5.0
EN
IN
Oligo
OPC
Astro
Immune
Mural
snTIM
Fine-mapping
EUR MA AFR
TWAS
EUR
MA
AFR
F
Migraines
UFL1
PRR16
ENSG00000
285082
TMEM165
CLOCK
STAT6
UBE2E3
SKOR1
EPHA10
VWDE
Gene name
6:96,030,421
-99,838,534
5:120,332,801
-121,844,262
9:117,266,094
-119,020,547
4:53,011,670
-55,451,955
4:55,451,955
-57,212,747
12:56,586,012
-58,914,846
2:178,379,777
-181,402,106
15:66,748,759
-68,317,981
1:36,206,464
-38,266,469
7:10,304,399
-12,651,316
LD Block
PIP
1.00
0.50
0
Scaled
I-snTWAS
z-score
5.0
2.5
0.0
-2.5
EN
IN
Oligo
OPC
Astro
Immune
Mural
snTIM
