# Supplementary material for: Single-nucleus transcriptome-wide association study of human brain disorders
Source: Nature. 2026 Sep 23;657(8133):1016–26. doi: 10.1038/s41586-026-10836-6 (PMC13600940; doi:10.1038/s41586-026-10836-6)
Supplement: Supplementary file 5 [file 41586_2026_10836_MOESM4_ESM.zip › 2024-09-19723C-s4/FigS21_part4.pdf]

G

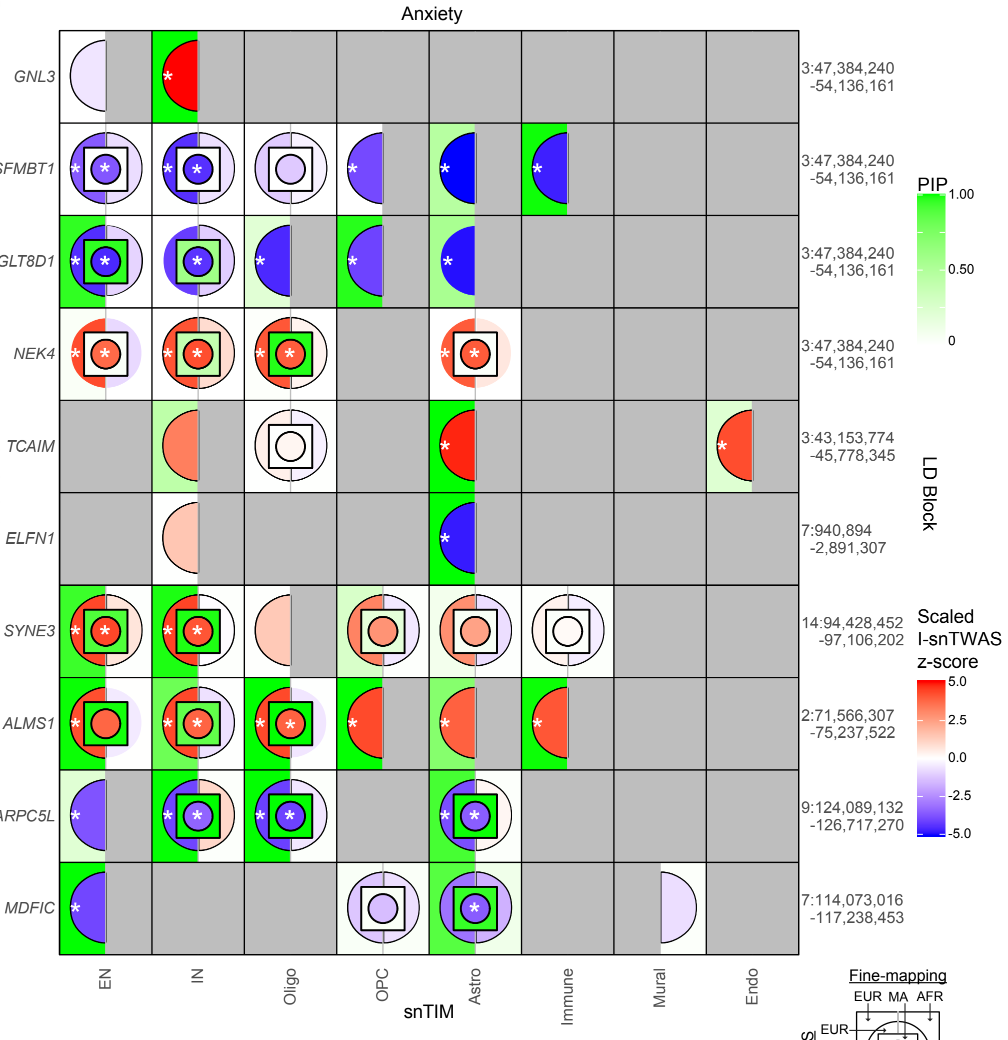
Anxiety
GNL3
SFMBT1
GLT8D1
NEK4
TCAIM
ELFN1
SYNE3
ALMS1
ARPC5L
MDFIC
Gene name
3:47,384,240 -54,136,161
3:47,384,240 -54,136,161
3:47,384,240 -54,136,161
3:47,384,240 -54,136,161
3:43,153,774 -45,778,345
7:940,894 -2,891,307
14:94,428,452 -97,106,202
2:71,566,307 -75,237,522
9:124,089,132 -126,717,270
7:114,073,016 -117,238,453
LD Block
PIP
1.00
0.50
0
Scaled I-snTWAS z-score
5.0
2.5
0.0
-2.5
-5.0
EN
IN
Oligo
OPC
Astro
Immune
Mural
Endo
snTIM
Fine-mapping
EUR
MA
AFR
TWAS
EUR
MA
AFR

H

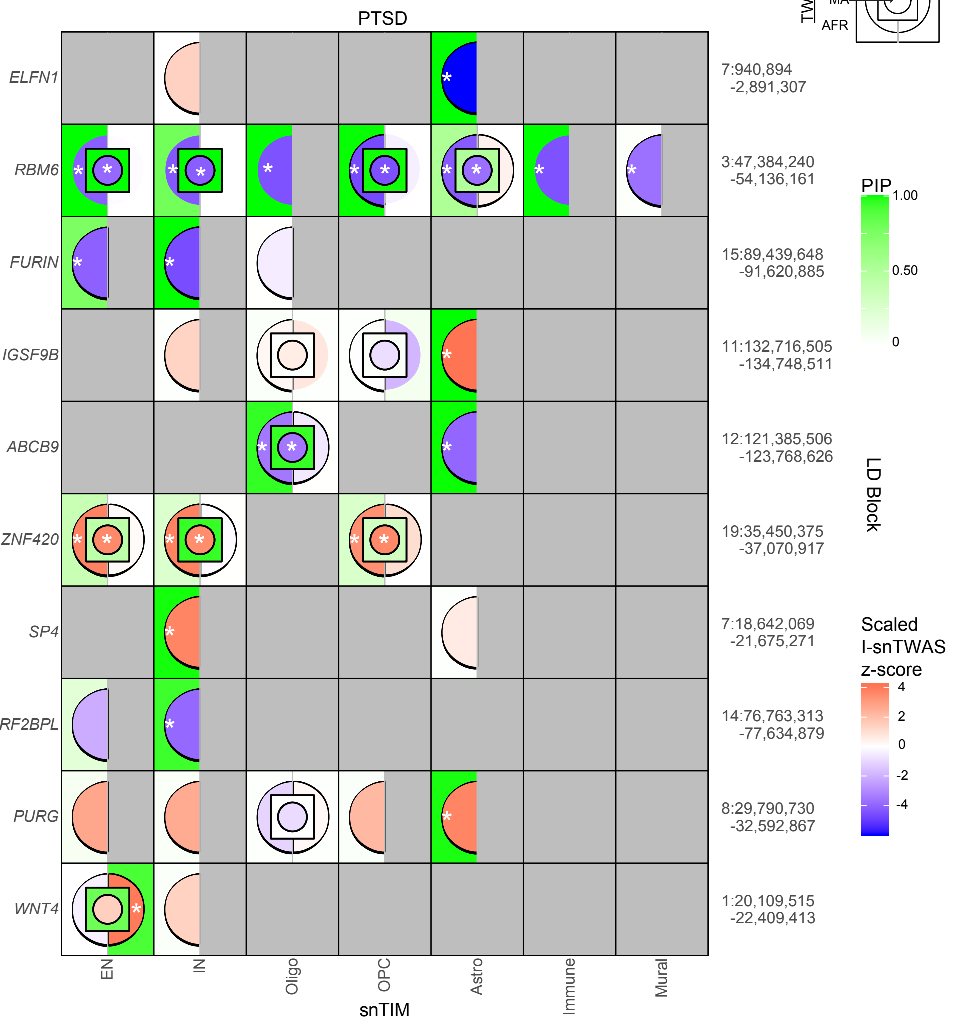
PTSD
ELFN1
RBM6
FURIN
IGSF9B
ABCB9
ZNF420
SP4
IRF2BPL
PURG
WNT4
Gene name
7:940,894 -2,891,307
3:47,384,240 -54,136,161
15:89,439,648 -91,620,885
11:132,716,505 -134,748,511
12:121,385,506 -123,768,626
19:35,450,375 -37,070,917
7:18,642,069 -21,675,271
14:76,763,313 -77,634,879
8:29,790,730 -32,592,867
1:20,109,515 -22,409,413
LD Block
PIP
1.00
0.50
0
Scaled I-snTWAS z-score
4
2
0
-2
-4
EN
IN
Oligo
OPC
Astro
Immune
Mural
snTIM
