# Supplementary material for: Single-nucleus transcriptome-wide association study of human brain disorders
Source: Nature. 2026 Sep 23;657(8133):1016–26. doi: 10.1038/s41586-026-10836-6 (PMC13600940; doi:10.1038/s41586-026-10836-6)
Supplement: Supplementary file 5 [file 41586_2026_10836_MOESM4_ESM.zip › 2024-09-19723C-s4/FigS22.pdf]

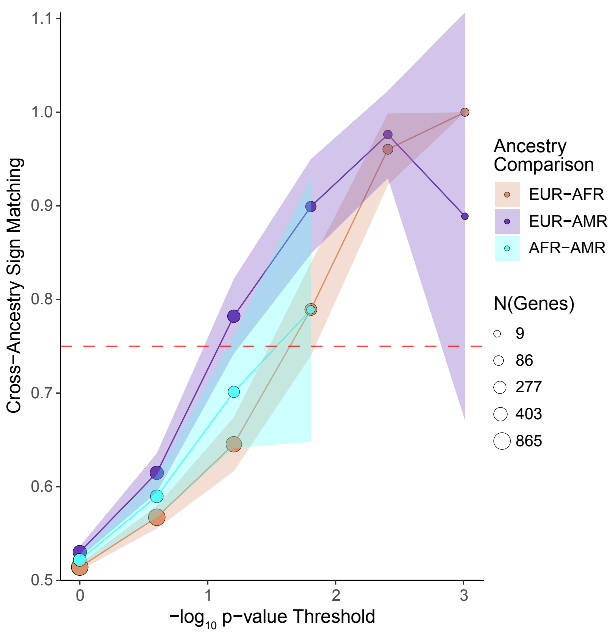
Cross−Ancestry Sign Matching
−log10 p−value Threshold
Ancestry Comparison
EUR−AFR
EUR−AMR
AFR−AMR
N(Genes)
9
86
277
403
865
0.5
0.6
0.7
0.8
0.9
1.0
1.1
0
1
2
3
