# Supplementary material for: Single-nucleus transcriptome-wide association study of human brain disorders
Source: Nature. 2026 Sep 23;657(8133):1016–26. doi: 10.1038/s41586-026-10836-6 (PMC13600940; doi:10.1038/s41586-026-10836-6)
Supplement: Supplementary file 5 [file 41586_2026_10836_MOESM4_ESM.zip › 2024-09-19723C-s4/FigS23.pdf]

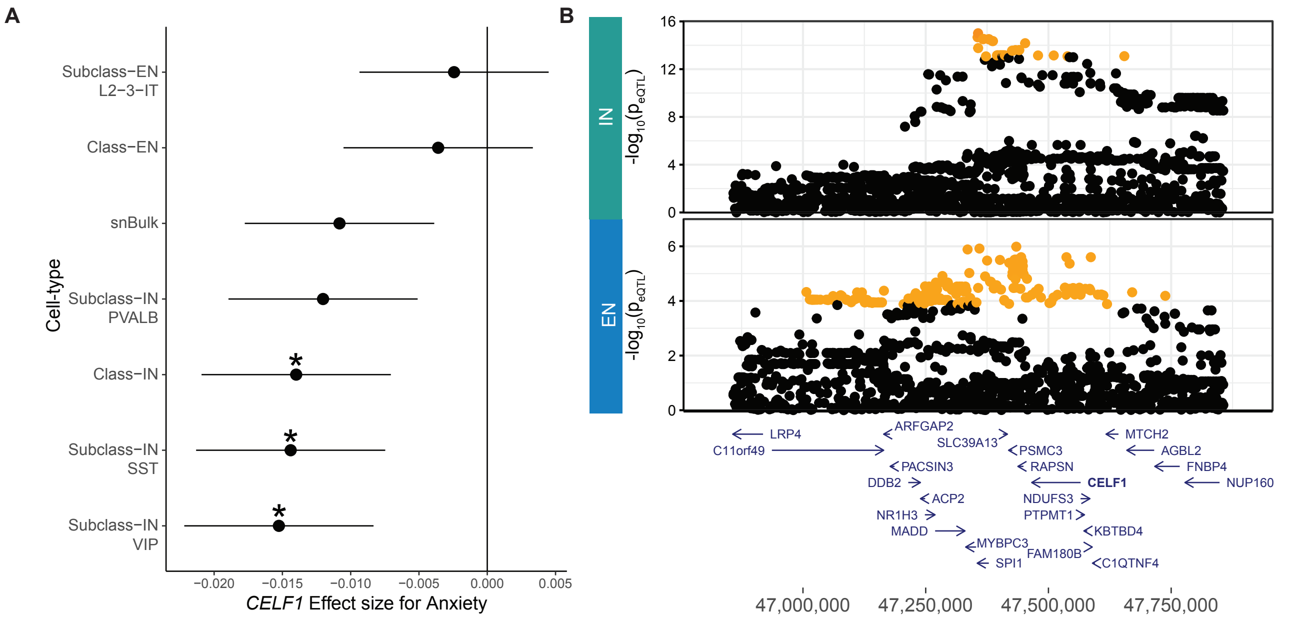
A
Subclass−EN L2−3−IT
Class−EN
snBulk
Subclass−IN PVALB
Class−IN
Subclass−IN SST
Subclass−IN VIP
Cell-type
*
*
*
−0.020
−0.015
−0.010
−0.005
0.000
0.005
CELF1 Effect size for Anxiety
B
IN
EN
-log10(peQTL)
-log10(peQTL)
16
12
8
4
0
6
4
2
0
LRP4
C11orf49
ARFGAP2
SLC39A13
PACSIN3
DDB2
ACP2
NR1H3
MADD
MYBPC3
SPI1
PSMC3
RAPSN
CELF1
NDUFS3
PTPMT1
KBTBD4
FAM180B
C1QTNF4
MTCH2
AGBL2
FNBP4
NUP160
47,000,000
47,250,000
47,500,000
47,750,000
