# Supplementary material for: Single-nucleus transcriptome-wide association study of human brain disorders
Source: Nature. 2026 Sep 23;657(8133):1016–26. doi: 10.1038/s41586-026-10836-6 (PMC13600940; doi:10.1038/s41586-026-10836-6)
Supplement: Supplementary file 5 [file 41586_2026_10836_MOESM4_ESM.zip › 2024-09-19723C-s4/FigS24_part1.pdf]

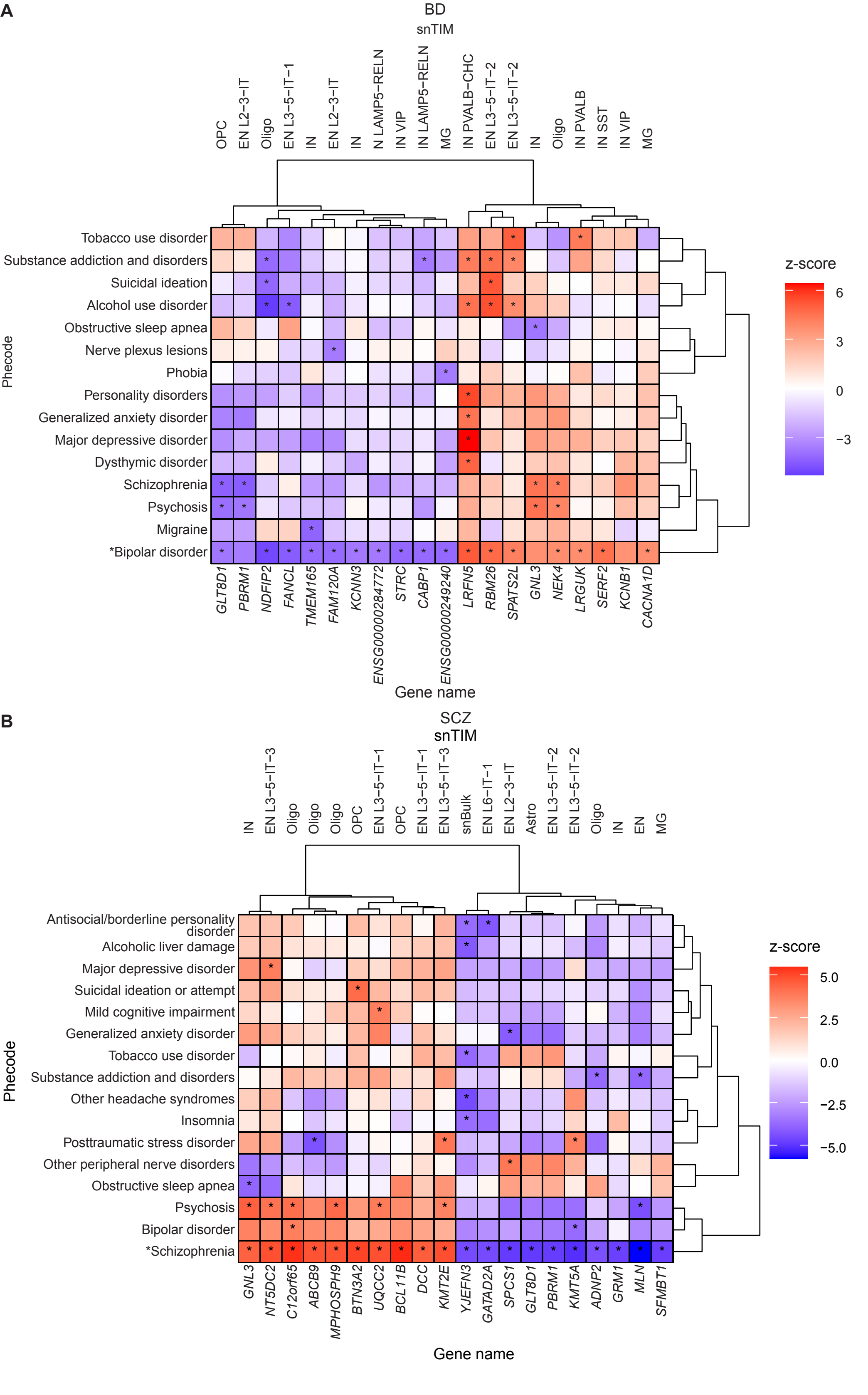

A
BD
snTIM
OPC
EN L2-3-IT
Oligo
EN L3-5-IT-1
IN
EN L2-3-IT
IN
N LAMP5-RELN
IN VIP
IN LAMP5-RELN
MG
IN PVALB-CHC
EN L3-5-IT-2
EN L3-5-IT-2
IN
Oligo
IN PVALB
IN SST
IN VIP
MG
Tobacco use disorder
Substance addiction and disorders
Suicidal ideation
Alcohol use disorder
Obstructive sleep apnea
Nerve plexus lesions
Phobia
Personality disorders
Generalized anxiety disorder
Major depressive disorder
Dysthymic disorder
Schizophrenia
Psychosis
Migraine
*Bipolar disorder
Phecode
GLT8D1
PBRM1
NDFIP2
FANCL
TMEM165
FAM120A
KCNN3
ENSG00000284772
STRC
CABP1
ENSG00000249240
LRFN5
RBM26
SPATS2L
GNL3
NEK4
LRGUK
SERF2
KCNB1
CACNA1D
Gene name
z-score
6
3
0
−3
B
SCZ
snTIM
IN
EN L3-5-IT-3
Oligo
Oligo
Oligo
OPC
EN L3-5-IT-1
OPC
EN L3-5-IT-1
EN L3-5-IT-3
snBulk
EN L6-IT-1
EN L2-3-IT
Astro
EN L3-5-IT-2
EN L3-5-IT-2
Oligo
IN
EN
MG
Antisocial/borderline personality disorder
Alcoholic liver damage
Major depressive disorder
Suicidal ideation or attempt
Mild cognitive impairment
Generalized anxiety disorder
Tobacco use disorder
Substance addiction and disorders
Other headache syndromes
Insomnia
Posttraumatic stress disorder
Other peripheral nerve disorders
Obstructive sleep apnea
Psychosis
Bipolar disorder
*Schizophrenia
Phecode
GNL3
NT5DC2
C12orf65
ABCB9
MPHOSPH9
BTN3A2
UQCC2
BCL11B
DCC
KMT2E
YJEFN3
GATAD2A
SPCS1
GLT8D1
PBRM1
KMT5A
ADNP2
GRM1
MLN
SFMBT1
Gene name
z-score
5.0
2.5
0.0
−2.5
−5.0
