# Supplementary material for: Single-nucleus transcriptome-wide association study of human brain disorders
Source: Nature. 2026 Sep 23;657(8133):1016–26. doi: 10.1038/s41586-026-10836-6 (PMC13600940; doi:10.1038/s41586-026-10836-6)
Supplement: Supplementary file 5 [file 41586_2026_10836_MOESM4_ESM.zip › 2024-09-19723C-s4/FigS24_part2.pdf]

C

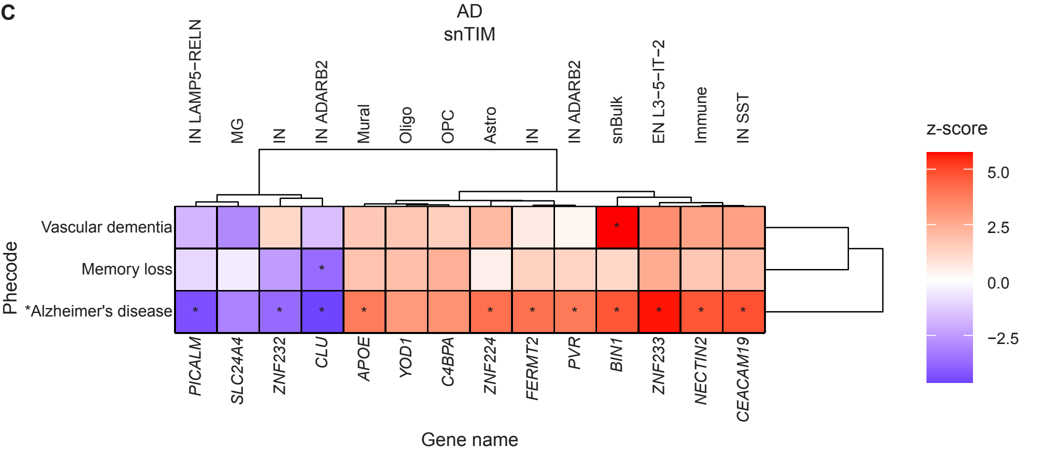

AD
snTIM
IN LAMP5−RELN
MG
IN
IN ADARB2
Mural
Oligo
OPC
Astro
IN
IN ADARB2
snBulk
EN L3−5−IT−2
Immune
IN SST
z-score
5.0
2.5
0.0
−2.5
Phecode
Vascular dementia
Memory loss
*Alzheimer's disease
PICALM
SLC24A4
ZNF232
CLU
APOE
YOD1
C4BPA
ZNF224
FERMT2
PVR
BIN1
ZNF233
NECTIN2
CEACAM19
Gene name

D

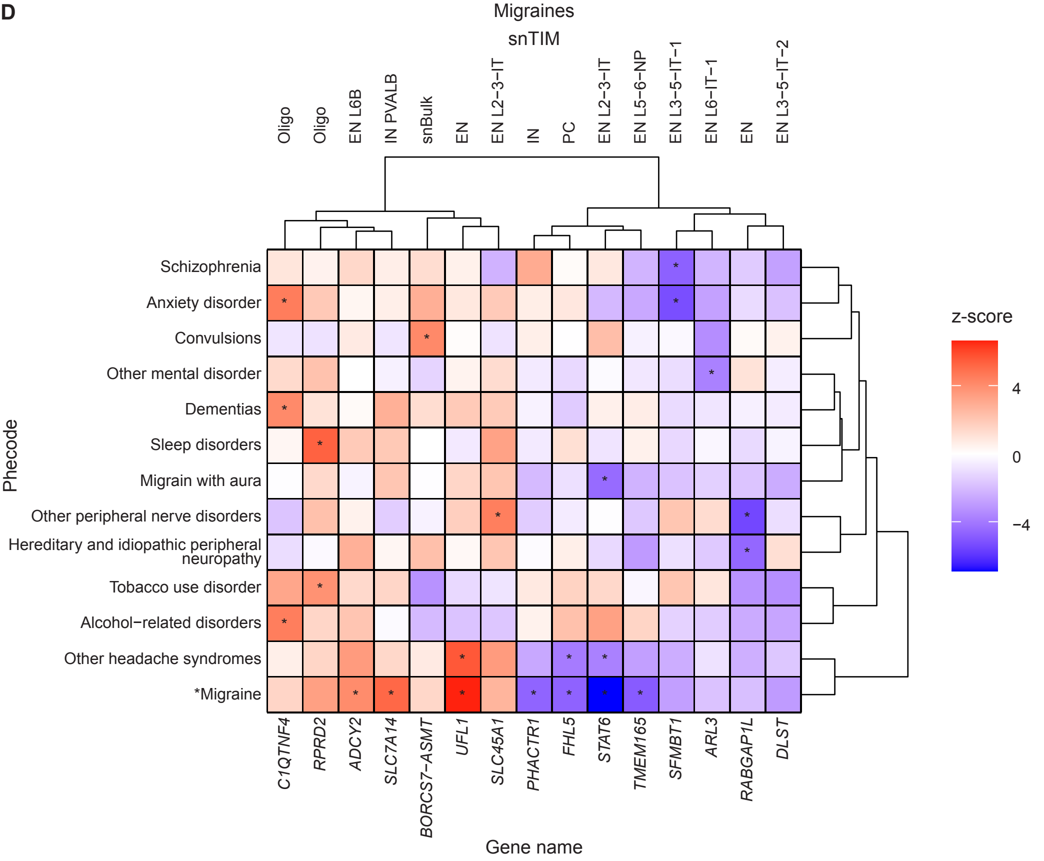

Migraines
snTIM
Oligo
Oligo
EN L6B
IN PVALB
snBulk
EN
EN L2−3−IT
IN
PC
EN L2−3−IT
EN L5−6−NP
EN L3−5−IT−1
EN L6−IT−1
EN
EN L3−5−IT−2
z-score
4
0
−4
Phecode
Schizophrenia
Anxiety disorder
Convulsions
Other mental disorder
Dementias
Sleep disorders
Migrain with aura
Other peripheral nerve disorders
Hereditary and idiopathic peripheral neuropathy
Tobacco use disorder
Alcohol−related disorders
Other headache syndromes
*Migraine
C1QTNF4
RPRD2
ADCY2
SLC7A14
BORCS7−ASMT
UFL1
SLC45A1
PHACTR1
FHL5
STAT6
TMEM165
SFMBT1
ARL3
RABGAP1L
DLST
Gene name
