# Supplementary material for: Single-nucleus transcriptome-wide association study of human brain disorders
Source: Nature. 2026 Sep 23;657(8133):1016–26. doi: 10.1038/s41586-026-10836-6 (PMC13600940; doi:10.1038/s41586-026-10836-6)
Supplement: Supplementary file 5 [file 41586_2026_10836_MOESM4_ESM.zip › 2024-09-19723C-s4/FigS24_part3.pdf]

E

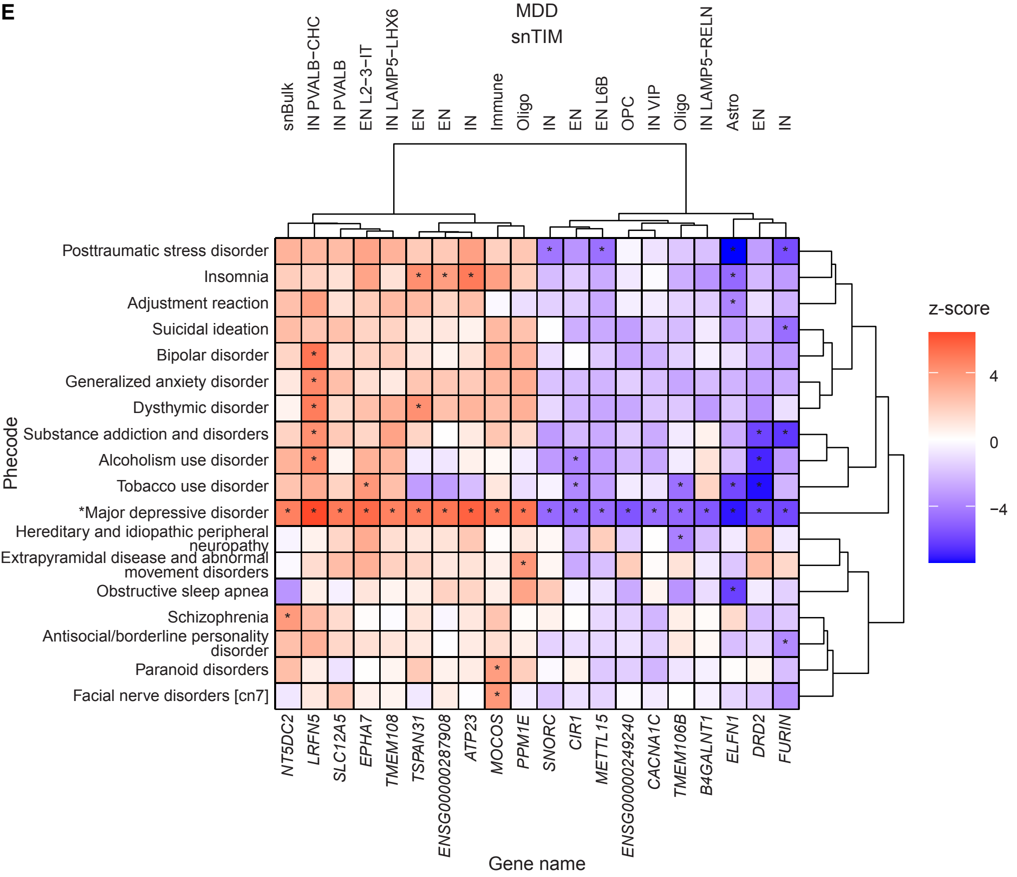
MDD
snTIM
snBulk
IN PVALB−CHC
IN PVALB
EN L2−3−IT
IN LAMP5−LHX6
EN
EN
IN
Immune
Oligo
IN
EN
EN L6B
OPC
IN VIP
Oligo
IN LAMP5−RELN
Astro
EN
IN
Posttraumatic stress disorder
Insomnia
Adjustment reaction
Suicidal ideation
Bipolar disorder
Generalized anxiety disorder
Dysthymic disorder
Substance addiction and disorders
Alcoholism use disorder
Tobacco use disorder
*Major depressive disorder
Hereditary and idiopathic peripheral neuropathy
Extrapyramidal disease and abnormal movement disorders
Obstructive sleep apnea
Schizophrenia
Antisocial/borderline personality disorder
Paranoid disorders
Facial nerve disorders [cn7]
Phecode
NT5DC2
LRFN5
SLC12A5
EPHA7
TMEM108
TSPAN31
ENSG00000287908
ATP23
MOCOS
PPM1E
SNORC
CIR1
METTL15
ENSG00000249240
CACNA1C
TMEM106B
B4GALNT1
ELFN1
DRD2
FURIN
Gene name
z-score
4
0
−4

F

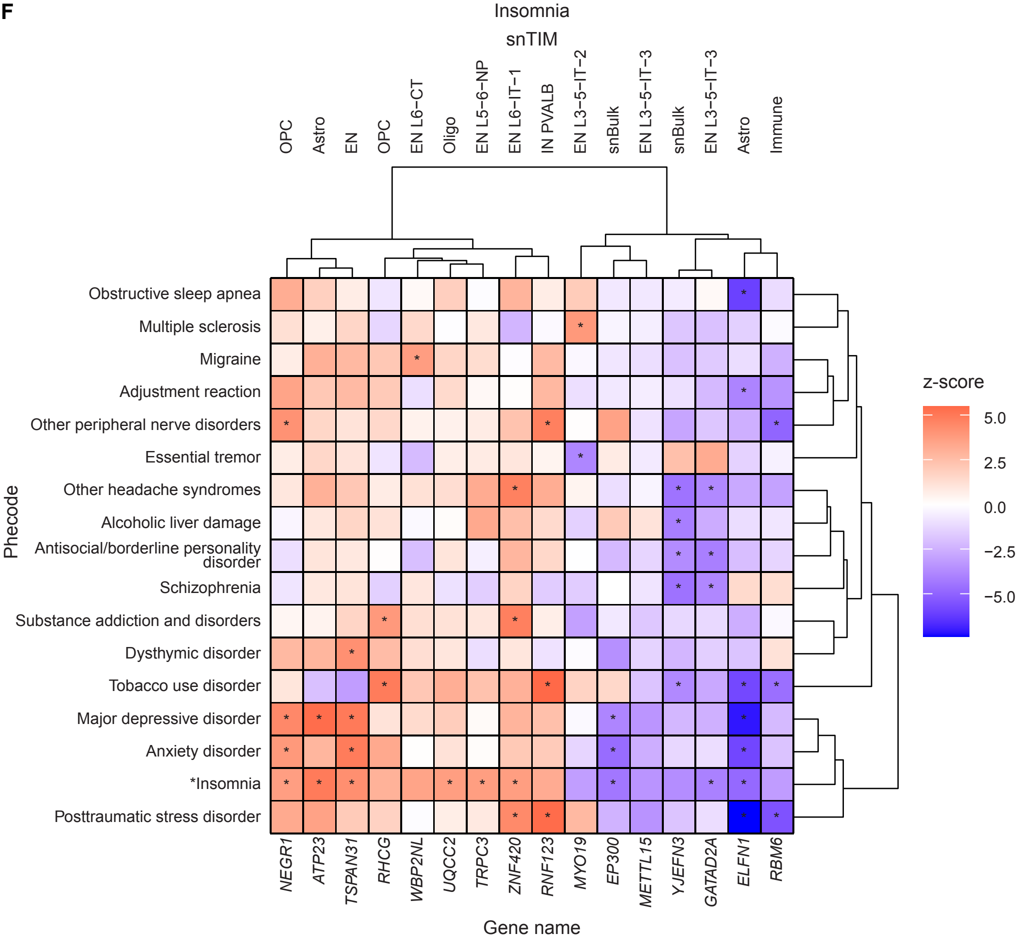
Insomnia
snTIM
OPC
Astro
EN
OPC
EN L6-CT
Oligo
EN L5−6−NP
EN L6−IT−1
IN PVALB
EN L3−5−IT−2
snBulk
EN L3−5−IT−3
snBulk
EN L3−5−IT−3
Astro
Immune
Obstructive sleep apnea
Multiple sclerosis
Migraine
Adjustment reaction
Other peripheral nerve disorders
Essential tremor
Other headache syndromes
Alcoholic liver damage
Antisocial/borderline personality disorder
Schizophrenia
Substance addiction and disorders
Dysthymic disorder
Tobacco use disorder
Major depressive disorder
Anxiety disorder
*Insomnia
Posttraumatic stress disorder
Phecode
NEGR1
ATP23
TSPAN31
RHCG
WBP2NL
UQCC2
TRPC3
ZNF420
RNF123
MYO19
EP300
METTL15
YJEFN3
GATAD2A
ELFN1
RBM6
Gene name
z-score
5.0
2.5
0.0
−2.5
−5.0
