# Supplementary material for: Single-nucleus transcriptome-wide association study of human brain disorders
Source: Nature. 2026 Sep 23;657(8133):1016–26. doi: 10.1038/s41586-026-10836-6 (PMC13600940; doi:10.1038/s41586-026-10836-6)
Supplement: Supplementary file 5 [file 41586_2026_10836_MOESM4_ESM.zip › 2024-09-19723C-s4/FigS25.pdf]

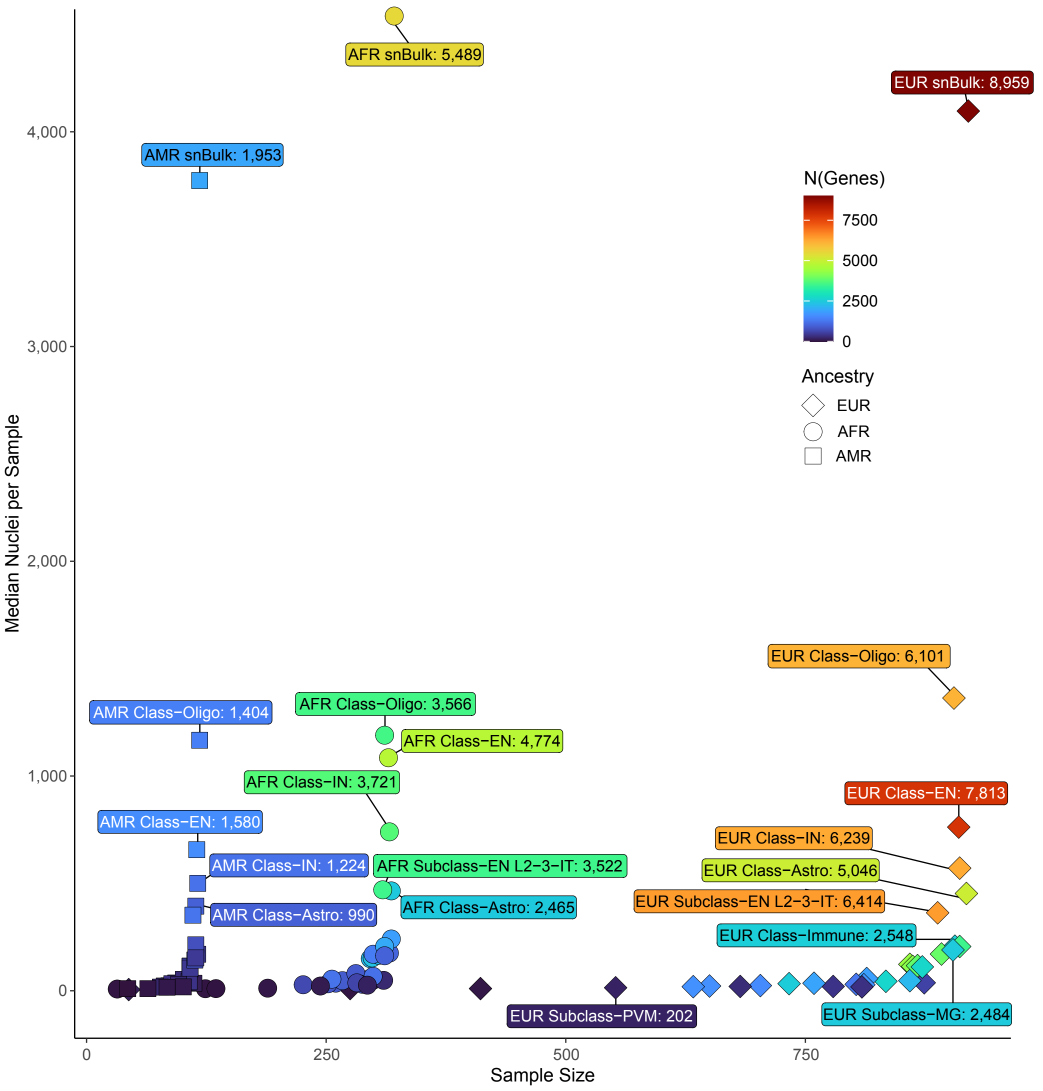
AFR snBulk: 5,489
EUR snBulk: 8,959
AMR snBulk: 1,953
N(Genes)
7500
5000
2500
0
Ancestry
EUR
AFR
AMR
EUR Class−Oligo: 6,101
AFR Class−Oligo: 3,566
AMR Class−Oligo: 1,404
AFR Class−EN: 4,774
AFR Class−IN: 3,721
EUR Class−EN: 7,813
AMR Class−EN: 1,580
EUR Class−IN: 6,239
AMR Class−IN: 1,224
AFR Subclass−EN L2−3−IT: 3,522
EUR Class−Astro: 5,046
EUR Subclass−EN L2−3−IT: 6,414
AMR Class−Astro: 990
AFR Class−Astro: 2,465
EUR Class−Immune: 2,548
EUR Subclass−PVM: 202
EUR Subclass−MG: 2,484
Median Nuclei per Sample
4,000
3,000
2,000
1,000
0
250
500
750
Sample Size
