# Supplementary material for: Single-nucleus transcriptome-wide association study of human brain disorders
Source: Nature. 2026 Sep 23;657(8133):1016–26. doi: 10.1038/s41586-026-10836-6 (PMC13600940; doi:10.1038/s41586-026-10836-6)
Supplement: Supplementary file 5 [file 41586_2026_10836_MOESM4_ESM.zip › 2024-09-19723C-s4/FigS26.pdf]

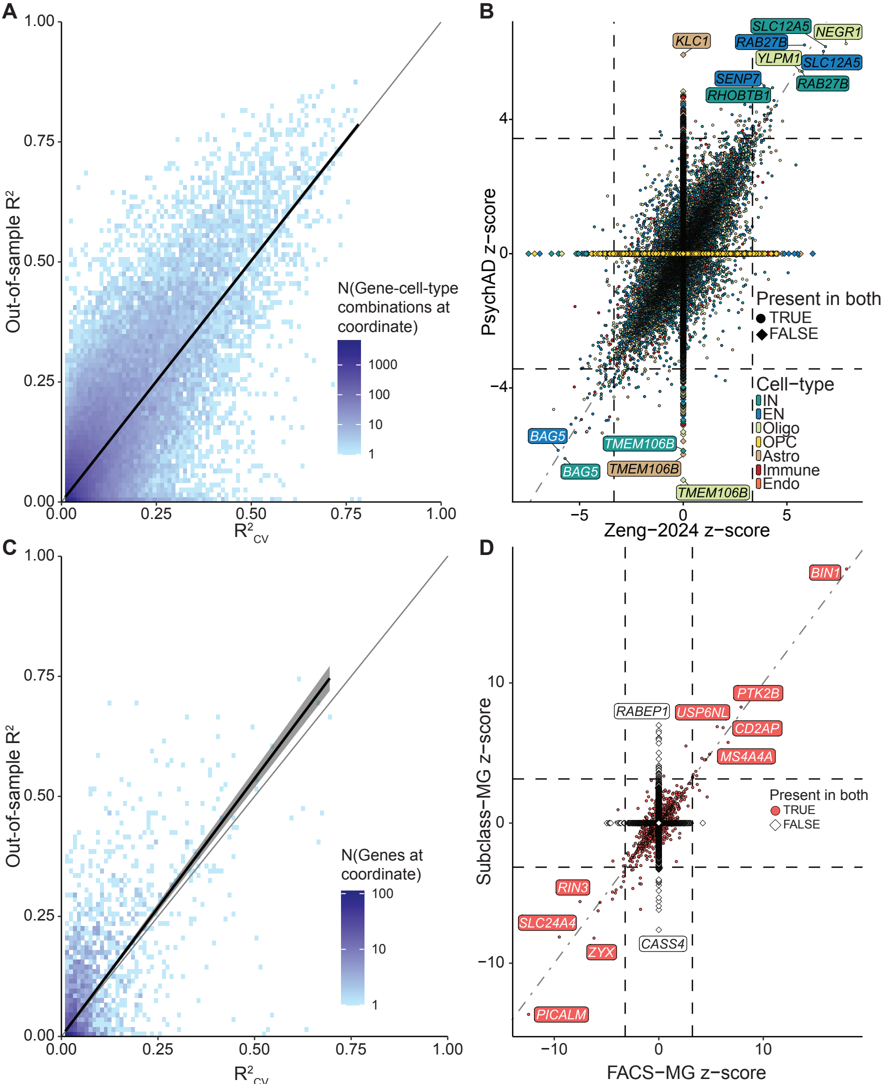

A
1.00
0.75
0.50
0.25
0.00
Out-of-sample R^2
0.00
0.25
0.50
0.75
1.00
R^2_{CV}
N(Gene-cell-type combinations at coordinate)
1000
100
10
1
B
KLC1
SLC12A5
NEGR1
RAB27B
YLPM1
SLC12A5
RAB27B
SENP7
RHOBTB1
BAG5
BAG5
TMEM106B
TMEM106B
TMEM106B
PsychAD z−score
Zeng−2024 z−score
4
0
−4
−5
0
5
Present in both
TRUE
FALSE
Cell−type
IN
EN
Oligo
OPC
Astro
Immune
Endo
C
1.00
0.75
0.50
0.25
0.00
Out-of-sample R^2
0.00
0.25
0.50
0.75
1.00
R^2_{CV}
N(Genes at coordinate)
100
10
1
D
BIN1
PTK2B
RABEP1
USP6NL
CD2AP
MS4A4A
RIN3
SLC24A4
ZYX
CASS4
PICALM
Subclass−MG z−score
FACS−MG z−score
10
0
−10
Present in both
TRUE
FALSE
