# Supplementary material for: Single-nucleus transcriptome-wide association study of human brain disorders
Source: Nature. 2026 Sep 23;657(8133):1016–26. doi: 10.1038/s41586-026-10836-6 (PMC13600940; doi:10.1038/s41586-026-10836-6)
Supplement: Supplementary file 5 [file 41586_2026_10836_MOESM4_ESM.zip › 2024-09-19723C-s4/FigS28.pdf]

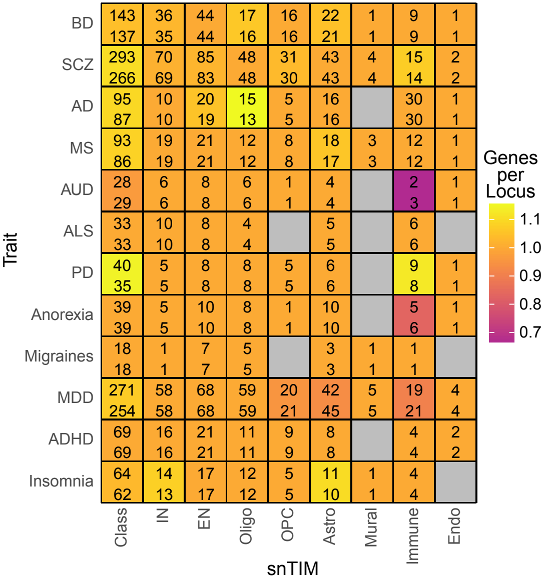
BD 143 36 44 17 16 22 1 9 1
137 35 44 16 16 21 1 9 1
SCZ 293 70 85 48 31 43 4 15 2
266 69 83 48 30 43 4 14 2
AD 95 10 20 15 5 16 30 1
87 10 19 13 5 16 30 1
MS 93 19 21 12 8 18 3 12 1
86 19 21 12 8 17 3 12 1
AUD 28 6 8 6 1 4 2 1
29 6 8 6 1 4 3 1
ALS 33 10 8 4 5 6
33 10 8 4 5 6
PD 40 5 8 8 5 6 9 1
35 5 8 8 5 6 8 1
Anorexia 39 5 10 8 1 10 5 1
39 5 10 8 1 10 6 1
Migraines 18 1 7 5 3 1 1
18 1 7 5 3 1 1
MDD 271 58 68 59 20 42 5 19 4
254 58 68 59 21 45 5 21 4
ADHD 69 16 21 11 9 8 4 2
69 16 21 11 9 8 4 2
Insomnia 64 14 17 12 5 11 1 4
62 13 17 12 5 10 1 4
Trait
Class
IN
EN
Oligo
OPC
Astro
Mural
Immune
Endo
snTIM
Genes per Locus
1.1
1.0
0.9
0.8
0.7
