# Supplementary material for: Single-nucleus transcriptome-wide association study of human brain disorders
Source: Nature. 2026 Sep 23;657(8133):1016–26. doi: 10.1038/s41586-026-10836-6 (PMC13600940; doi:10.1038/s41586-026-10836-6)
Supplement: Supplementary file 5 [file 41586_2026_10836_MOESM4_ESM.zip › 2024-09-19723C-s4/FigS29.pdf]

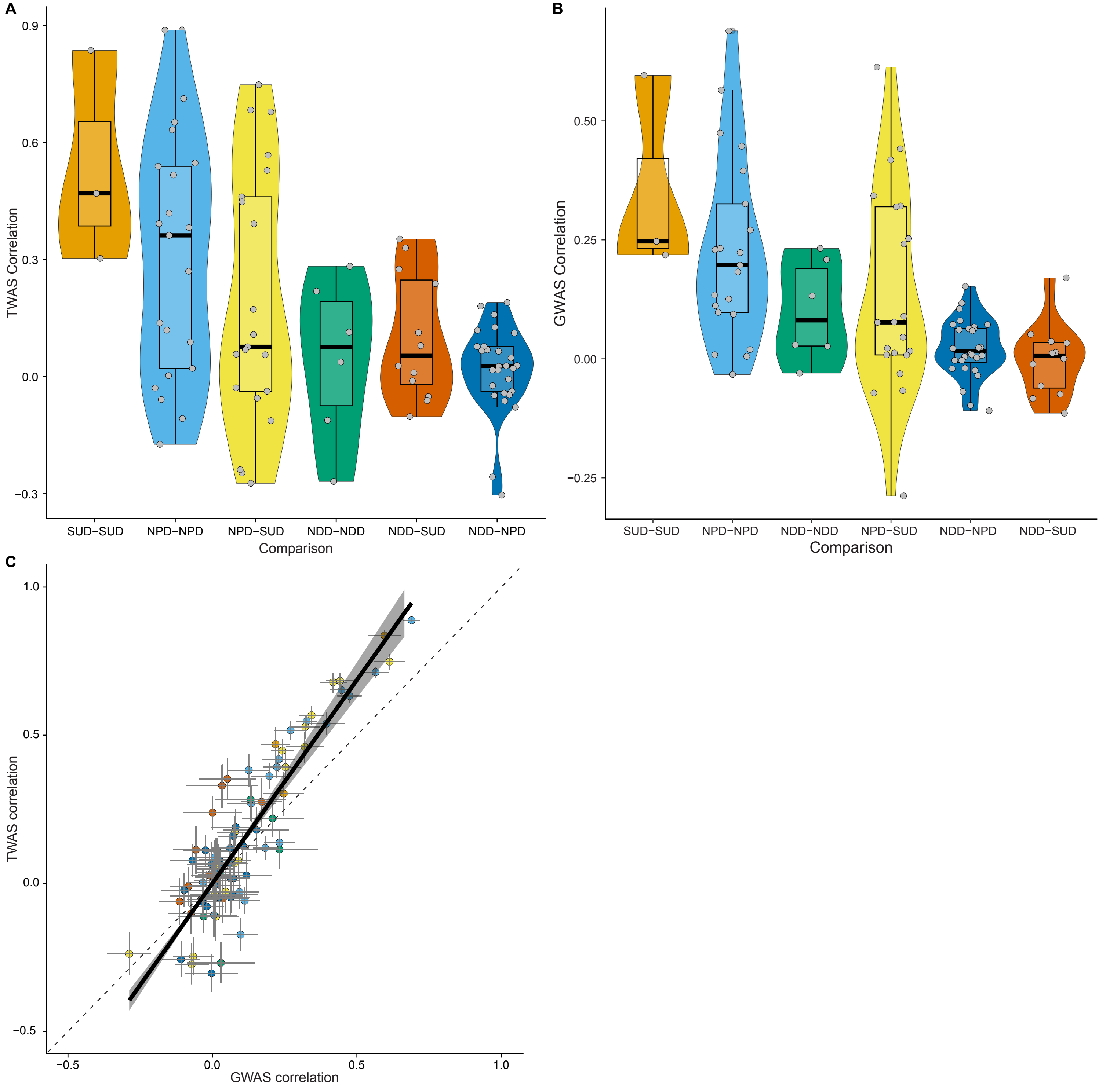

A
TWAS Correlation
0.9
0.6
0.3
0.0
−0.3
SUD−SUD
NPD−NPD
NPD−SUD
NDD−NDD
NDD−SUD
NDD−NPD
Comparison
B
GWAS Correlation
0.50
0.25
0.00
−0.25
SUD−SUD
NPD−NPD
NDD−NDD
NPD−SUD
NDD−NPD
NDD−SUD
Comparison
C
TWAS correlation
1.0
0.5
0.0
−0.5
GWAS correlation
−0.5
0.0
0.5
1.0
