# Supplementary material for: Single-nucleus transcriptome-wide association study of human brain disorders
Source: Nature. 2026 Sep 23;657(8133):1016–26. doi: 10.1038/s41586-026-10836-6 (PMC13600940; doi:10.1038/s41586-026-10836-6)
Supplement: Supplementary file 5 [file 41586_2026_10836_MOESM4_ESM.zip › 2024-09-19723C-s4/FigS30.pdf]

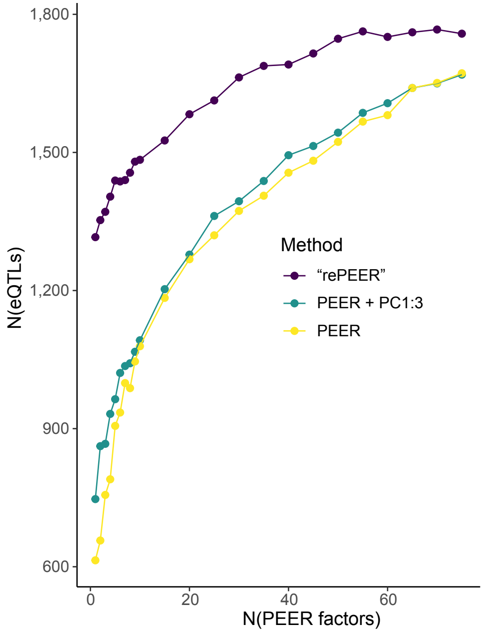

N(eQTLs)
1,800
1,500
1,200
900
600
0
20
40
60
N(PEER factors)
Method
“rePEER”
PEER + PC1:3
PEER
