# Supplementary material for: Single-nucleus transcriptome-wide association study of human brain disorders
Source: Nature. 2026 Sep 23;657(8133):1016–26. doi: 10.1038/s41586-026-10836-6 (PMC13600940; doi:10.1038/s41586-026-10836-6)
Supplement: Supplementary file 5 [file 41586_2026_10836_MOESM4_ESM.zip › 2024-09-19723C-s4/FigS31.pdf]

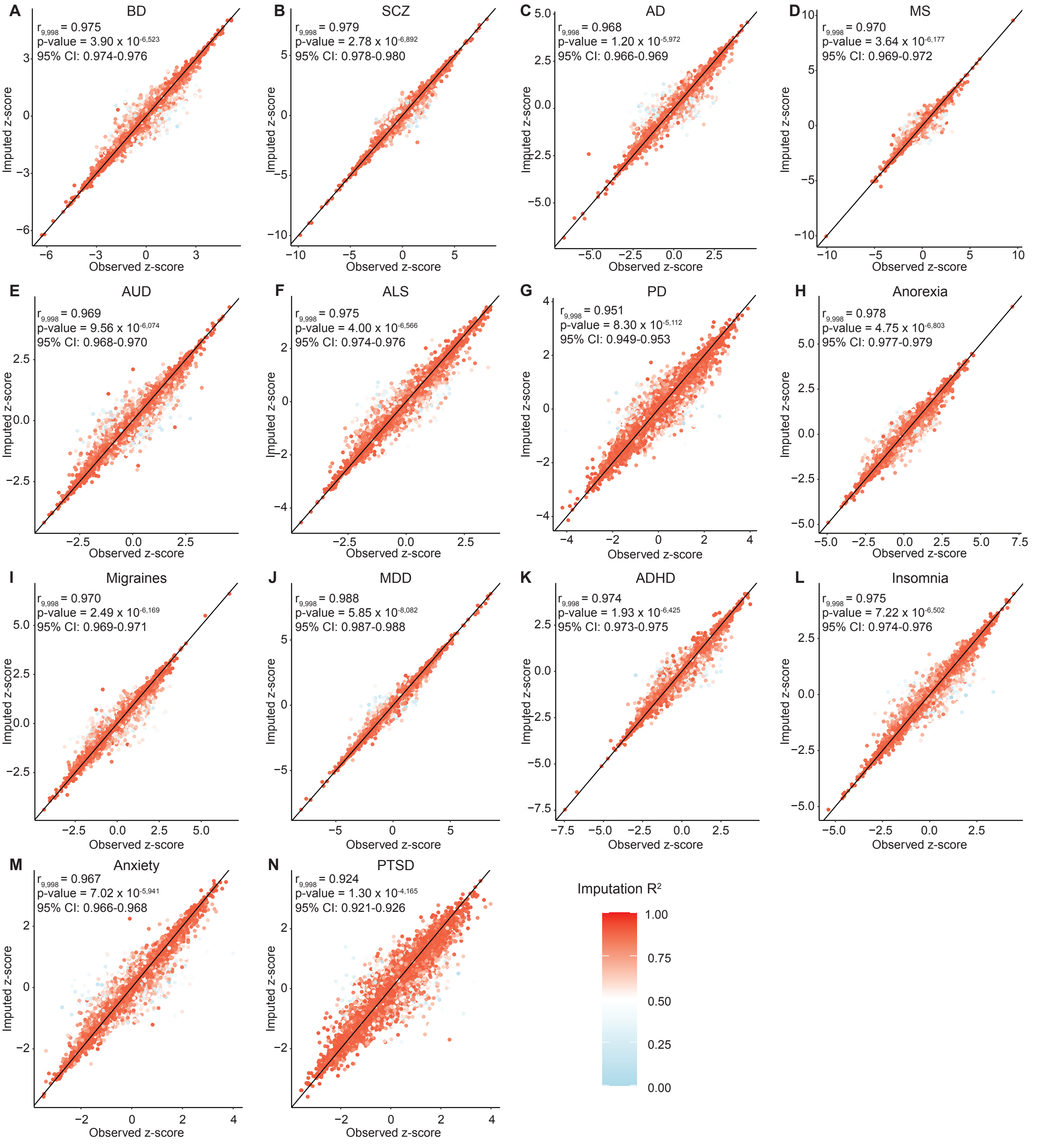
A
BD
r9,998 = 0.975
p-value = 3.90 x 10-6,523
95% CI: 0.974-0.976
B
SCZ
r9,998 = 0.979
p-value = 2.78 x 10-6,892
95% CI: 0.978-0.980
C
AD
r9,998 = 0.968
p-value = 1.20 x 10-5,972
95% CI: 0.966-0.969
D
MS
r9,998 = 0.970
p-value = 3.64 x 10-6,177
95% CI: 0.969-0.972
E
AUD
r9,998 = 0.969
p-value = 9.56 x 10-6,074
95% CI: 0.968-0.970
F
ALS
r9,998 = 0.975
p-value = 4.00 x 10-6,566
95% CI: 0.974-0.976
G
PD
r9,998 = 0.951
p-value = 8.30 x 10-5,112
95% CI: 0.949-0.953
H
Anorexia
r9,998 = 0.978
p-value = 4.75 x 10-6,803
95% CI: 0.977-0.979
I
Migraines
r9,998 = 0.970
p-value = 2.49 x 10-6,169
95% CI: 0.969-0.971
J
MDD
r9,998 = 0.988
p-value = 5.85 x 10-8,082
95% CI: 0.987-0.988
K
ADHD
r9,998 = 0.974
p-value = 1.93 x 10-6,425
95% CI: 0.973-0.975
L
Insomnia
r9,998 = 0.975
p-value = 7.22 x 10-6,502
95% CI: 0.974-0.976
M
Anxiety
r9,998 = 0.967
p-value = 7.02 x 10-5,941
95% CI: 0.966-0.968
N
PTSD
r9,998 = 0.924
p-value = 1.30 x 10-4,165
95% CI: 0.921-0.926
Imputed z-score
Observed z-score
Imputation R2
1.00
0.75
0.50
0.25
0.00
