# Supplementary material for: Single-nucleus transcriptome-wide association study of human brain disorders
Source: Nature. 2026 Sep 23;657(8133):1016–26. doi: 10.1038/s41586-026-10836-6 (PMC13600940; doi:10.1038/s41586-026-10836-6)
Supplement: Supplementary file 5 [file 41586_2026_10836_MOESM4_ESM.zip › 2024-09-19723C-s4/FigS32.pdf]

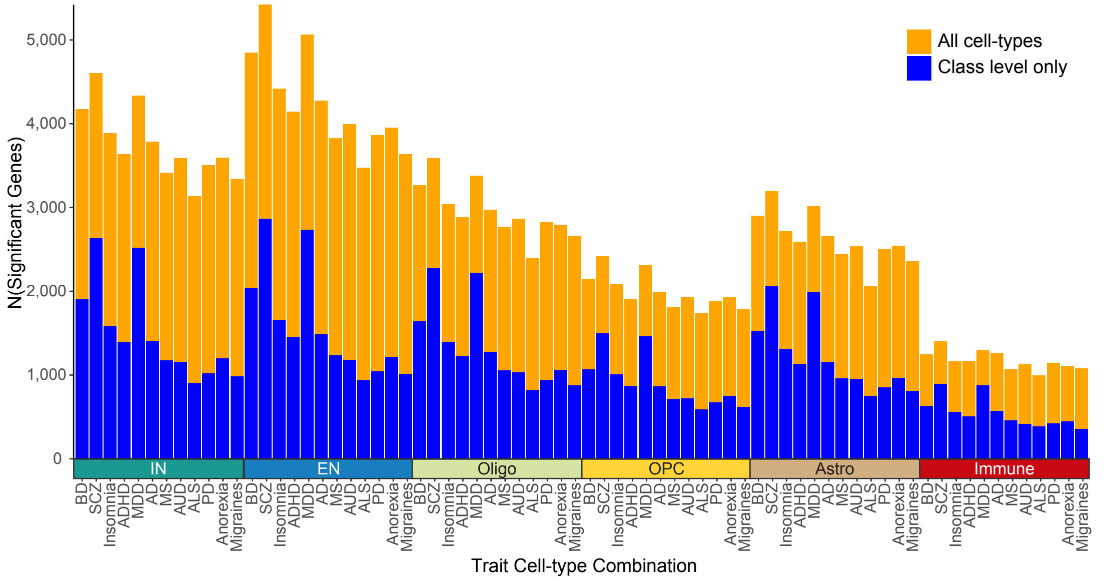
N(Significant Genes)
5,000
4,000
3,000
2,000
1,000
0
All cell-types
Class level only
IN
EN
Oligo
OPC
Astro
Immune
BD
SCZ
Insomnia
ADHD
MDD
AD
MS
AUD
ALS
PD
Anorexia
Migraines
Trait Cell-type Combination
