# Supplementary material for: Single-nucleus transcriptome-wide association study of human brain disorders
Source: Nature. 2026 Sep 23;657(8133):1016–26. doi: 10.1038/s41586-026-10836-6 (PMC13600940; doi:10.1038/s41586-026-10836-6)
Supplement: Supplementary file 5 [file 41586_2026_10836_MOESM4_ESM.zip › 2024-09-19723C-s4/FigS5.pdf]

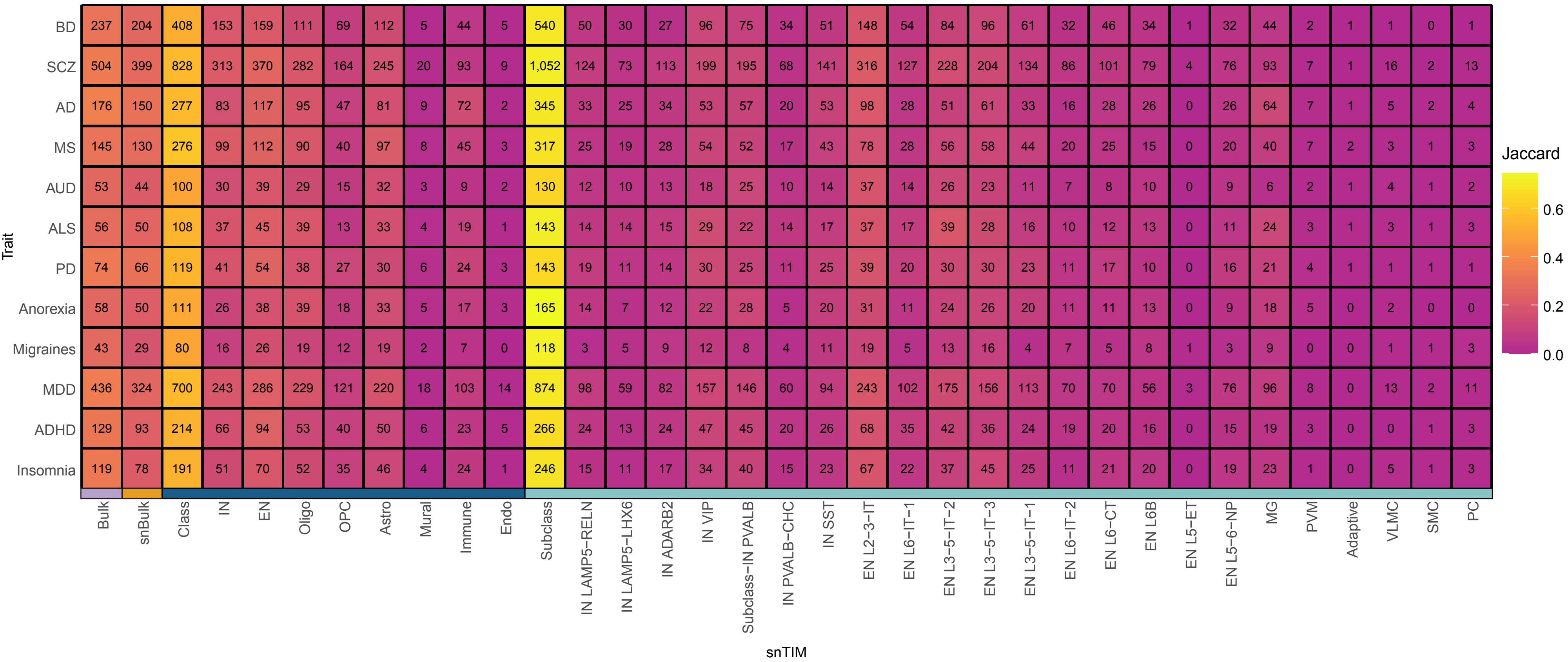
Trait
snTIM
Jaccard
0.0
0.2
0.4
0.6
Bulk
snBulk
Class
IN
EN
Oligo
OPC
Astro
Mural
Immune
Endo
Subclass
IN LAMP5−RELN
IN LAMP5−LHX6
IN ADARB2
IN VIP
Subclass−IN PVALB
IN PVALB−CHC
IN SST
EN L2−3−IT
EN L6−IT−1
EN L3−5−IT−2
EN L3−5−IT−3
EN L3−5−IT−1
EN L6−IT−2
EN L6−CT
EN L6B
EN L5−ET
EN L5−6−NP
MG
PVM
Adaptive
VLMC
SMC
PC
BD 237 204 408 153 159 111 69 112 5 44 5 540 50 30 27 96 75 34 51 148 54 84 96 61 32 46 34 1 32 44 2 1 1 0 1
SCZ 504 399 828 313 370 282 164 245 20 93 9 1,052 124 73 113 199 195 68 141 316 127 228 204 134 86 101 79 4 76 93 7 1 16 2 13
AD 176 150 277 83 117 95 47 81 9 72 2 345 33 25 34 53 57 20 53 98 28 51 61 33 16 28 26 0 26 64 7 1 5 2 4
MS 145 130 276 99 112 90 40 97 8 45 3 317 25 19 28 54 52 17 43 78 28 56 58 44 20 25 15 0 20 40 7 2 3 1 3
AUD 53 44 100 30 39 29 15 32 3 9 2 130 12 10 13 18 25 10 14 37 14 26 23 11 7 8 10 0 9 6 2 1 4 1 2
ALS 56 50 108 37 45 39 13 33 4 19 1 143 14 14 15 29 22 14 17 37 17 39 28 16 10 12 13 0 11 24 3 1 3 1 3
PD 74 66 119 41 54 38 27 30 6 24 3 143 19 11 14 30 25 11 25 39 20 30 30 23 11 17 10 0 16 21 4 1 1 1 1
Anorexia 58 50 111 26 38 39 18 33 5 17 3 165 14 7 12 22 28 5 20 31 11 24 26 20 11 11 13 0 9 18 5 0 2 0 0
Migraines 43 29 80 16 26 19 12 19 2 7 0 118 3 5 9 12 8 4 11 19 5 13 16 4 7 5 8 1 3 9 0 0 1 1 3
MDD 436 324 700 243 286 229 121 220 18 103 14 874 98 59 82 157 146 60 94 243 102 175 156 113 70 70 56 3 76 96 8 0 13 2 11
ADHD 129 93 214 66 94 53 40 50 6 23 5 266 24 13 24 47 45 20 26 68 35 42 36 24 19 20 16 0 15 19 3 0 0 1 3
Insomnia 119 78 191 51 70 52 35 46 4 24 1 246 15 11 17 34 40 15 23 67 22 37 45 25 11 21 20 0 19 23 1 0 5 1 3
