# Supplementary material for: Single-nucleus transcriptome-wide association study of human brain disorders
Source: Nature. 2026 Sep 23;657(8133):1016–26. doi: 10.1038/s41586-026-10836-6 (PMC13600940; doi:10.1038/s41586-026-10836-6)
Supplement: Supplementary file 5 [file 41586_2026_10836_MOESM4_ESM.zip › 2024-09-19723C-s4/FigS6.pdf]

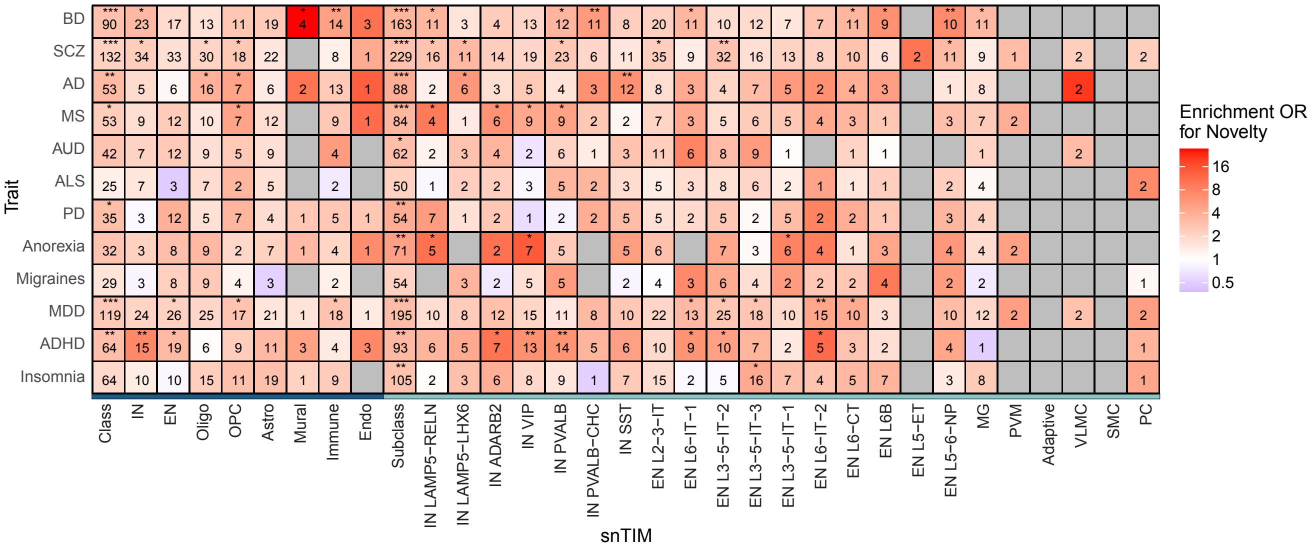
Enrichment OR for Novelty
16
8
4
2
1
0.5
Trait
BD
SCZ
AD
MS
AUD
ALS
PD
Anorexia
Migraines
MDD
ADHD
Insomnia
snTIM
Class
IN
EN
Oligo
OPC
Astro
Mural
Immune
Endo
Subclass
IN LAMP5−RELN
IN LAMP5−LHX6
IN ADARB2
IN VIP
IN PVALB
IN PVALB−CHC
IN SST
EN L2−3−IT
EN L6−IT−1
EN L3−5−IT−2
EN L3−5−IT−3
EN L3−5−IT−1
EN L6−IT−2
EN L6−CT
EN L6B
EN L5−ET
EN L5−6−NP
MG
PVM
Adaptive
VLMC
SMC
PC
90 23 17 13 11 19 4 14 3 163 11 3 4 13 12 11 8 20 11 10 12 7 7 11 9 10 11
132 34 33 30 18 22 8 1 229 16 11 14 19 23 6 11 35 9 32 16 13 8 10 6 2 11 9 1 2 2
53 5 6 16 7 6 2 13 1 88 2 6 3 5 4 3 12 8 3 4 7 5 2 4 3 1 8 2
53 9 12 10 7 12 9 1 84 4 1 6 9 9 2 2 7 3 5 6 5 4 3 1 3 7 2
42 7 12 9 5 9 4 62 2 3 4 2 6 1 3 11 6 8 9 1 1 1 1 2
25 7 3 7 2 5 2 50 1 2 2 3 5 2 3 5 3 8 6 2 1 1 1 2 4 2
35 3 12 5 7 4 1 5 1 54 7 1 2 1 2 2 5 5 2 5 2 5 2 2 1 3 4
32 3 8 9 2 7 1 4 1 71 5 2 7 5 5 6 7 3 6 4 1 3 4 4 2
29 3 8 9 4 3 2 54 3 2 5 5 2 4 3 6 4 2 2 2 4 2 2 1
119 24 26 25 17 21 1 18 1 195 10 8 12 15 11 8 10 22 13 25 18 10 15 10 3 10 12 2 2 2
64 15 19 6 9 11 3 4 3 93 6 5 7 13 14 5 6 10 9 10 7 2 5 3 2 4 1 1
64 10 10 15 11 19 1 9 105 2 3 6 8 9 1 7 15 2 5 16 7 4 5 7 3 8 1
