# Supplementary material for: Single-nucleus transcriptome-wide association study of human brain disorders
Source: Nature. 2026 Sep 23;657(8133):1016–26. doi: 10.1038/s41586-026-10836-6 (PMC13600940; doi:10.1038/s41586-026-10836-6)
Supplement: Supplementary file 5 [file 41586_2026_10836_MOESM4_ESM.zip › 2024-09-19723C-s4/FigS7.pdf]

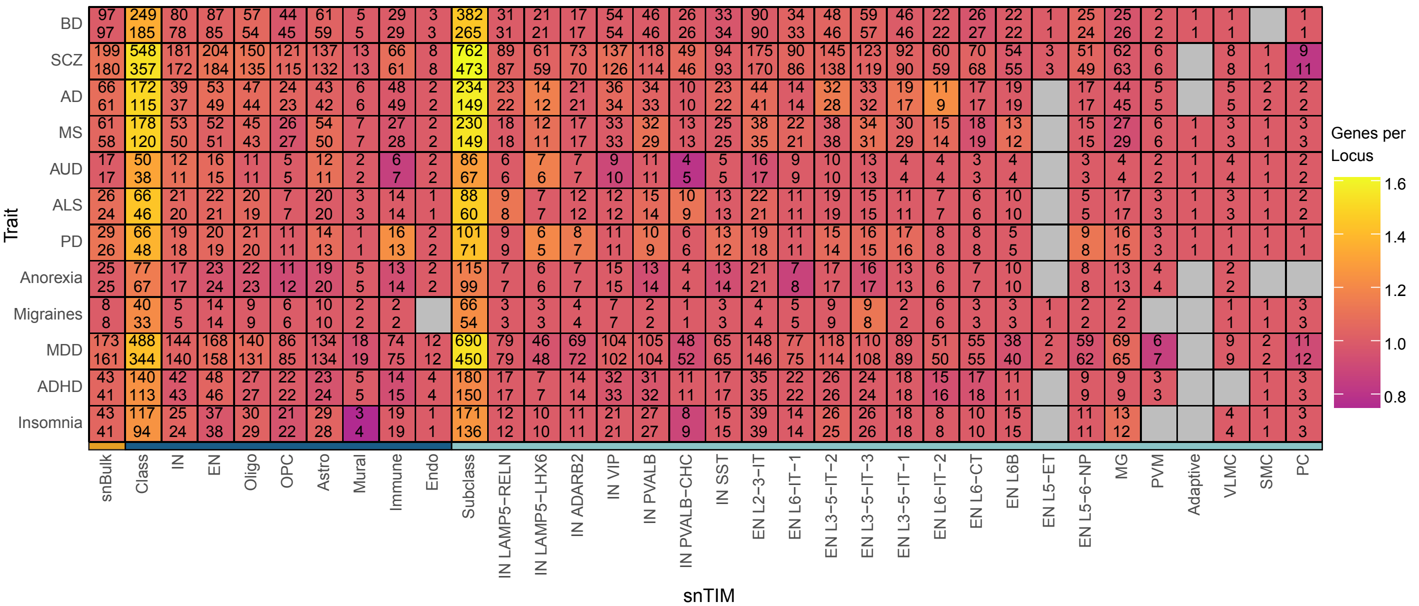
Trait
snTIM
Genes per Locus
1.6
1.4
1.2
1.0
0.8
snBulk Class IN EN Oligo OPC Astro Mural Immune Endo Subclass IN LAMP5−RELN IN LAMP5−LHX6 IN ADARB2 IN VIP IN PVALB IN PVALB−CHC IN SST EN L2−3−IT EN L6−IT−1 EN L3−5−IT−2 EN L3−5−IT−3 EN L3−5−IT−1 EN L6−IT−2 EN L6−CT EN L6B EN L5−ET EN L5−6−NP MG PVM Adaptive VLMC SMC PC
BD
97 249 80 87 57 44 61 5 29 3 382 31 21 17 54 46 26 33 90 34 48 59 46 22 26 22 1 25 25 2 1 1 1
97 185 78 85 54 45 59 5 29 3 265 31 21 17 54 46 26 34 90 33 46 57 46 22 27 22 1 24 26 2 1 1 1
SCZ
199 548 181 204 150 121 137 13 66 8 762 89 61 73 137 118 49 94 175 90 145 123 92 60 70 54 3 51 62 6 8 1 9
180 357 172 184 135 115 132 13 61 8 473 87 59 70 126 114 46 93 170 86 138 119 90 59 68 55 3 49 63 6 8 1 11
AD
66 172 39 53 47 24 43 6 48 2 234 23 14 21 36 34 10 23 44 14 32 33 19 11 17 19 17 44 5 5 2 2
61 115 37 49 44 23 42 6 49 2 149 22 12 21 34 33 10 22 41 14 28 32 17 9 17 19 17 45 5 5 2 2
MS
61 178 53 52 45 26 54 7 27 2 230 18 12 17 33 32 13 25 38 22 38 34 30 15 18 13 15 27 6 1 3 1 3
58 120 50 51 43 27 50 7 28 2 149 18 11 17 33 29 13 25 35 21 38 31 29 14 19 12 15 29 6 1 3 1 3
AUD
17 50 12 16 11 5 12 2 6 2 86 6 7 7 9 11 4 5 16 9 10 13 4 4 3 4 3 4 2 1 4 1 2
17 38 11 15 11 5 11 2 7 2 67 6 6 7 10 11 5 5 17 9 10 13 4 4 3 4 3 4 2 1 4 1 2
ALS
26 66 21 22 20 7 20 3 14 1 88 9 7 12 12 15 10 13 22 11 19 15 11 7 6 10 5 17 3 1 3 1 2
24 46 20 21 19 7 20 3 14 1 60 8 7 12 12 14 9 13 21 11 19 15 11 7 6 10 5 17 3 1 3 1 2
PD
29 66 19 20 21 11 14 1 16 2 101 9 6 8 11 10 6 13 19 11 15 16 17 8 8 5 9 16 3 1 1 1 1
26 48 18 19 20 11 13 1 13 2 71 9 5 7 11 9 6 12 18 11 14 15 16 8 8 5 8 15 3 1 1 1 1
Anorexia
25 77 17 23 22 11 19 5 13 2 115 7 6 7 15 13 4 13 21 7 17 16 13 6 7 10 8 13 4 2
25 67 17 24 23 12 20 5 14 2 99 7 6 7 15 14 4 14 21 8 17 17 13 6 7 10 8 13 4 2
Migraines
8 40 5 14 9 6 10 2 2 66 3 3 4 7 2 1 3 4 5 9 9 2 6 3 3 1 2 2 1 1 3
8 33 5 14 9 6 10 2 2 54 3 3 4 7 2 1 3 4 5 9 8 2 6 3 3 1 2 2 1 1 3
MDD
173 488 144 168 140 86 134 18 74 12 690 79 46 69 104 105 48 65 148 77 118 110 89 51 55 38 2 59 69 6 9 2 11
161 344 140 158 131 85 134 19 75 12 450 79 48 72 102 104 52 65 146 75 114 108 89 50 55 40 2 62 65 7 9 2 12
ADHD
43 140 42 48 27 22 23 5 14 4 180 17 7 14 32 31 11 17 35 22 26 24 18 15 17 11 9 9 3 1 3
41 113 43 46 27 22 24 5 15 4 150 17 7 14 33 32 11 17 35 22 26 24 18 16 18 11 9 9 3 1 3
Insomnia
43 117 25 37 30 21 29 3 19 1 171 12 10 11 21 27 8 15 39 14 26 26 18 8 10 15 11 13 4 1 3
41 94 24 38 29 22 28 4 19 1 136 12 10 11 21 27 9 15 39 14 25 26 18 8 10 15 11 12 4 1 3
