# Supplementary material for: Single-nucleus transcriptome-wide association study of human brain disorders
Source: Nature. 2026 Sep 23;657(8133):1016–26. doi: 10.1038/s41586-026-10836-6 (PMC13600940; doi:10.1038/s41586-026-10836-6)
Supplement: Supplementary file 5 [file 41586_2026_10836_MOESM4_ESM.zip › 2024-09-19723C-s4/FigS8_part1.pdf]

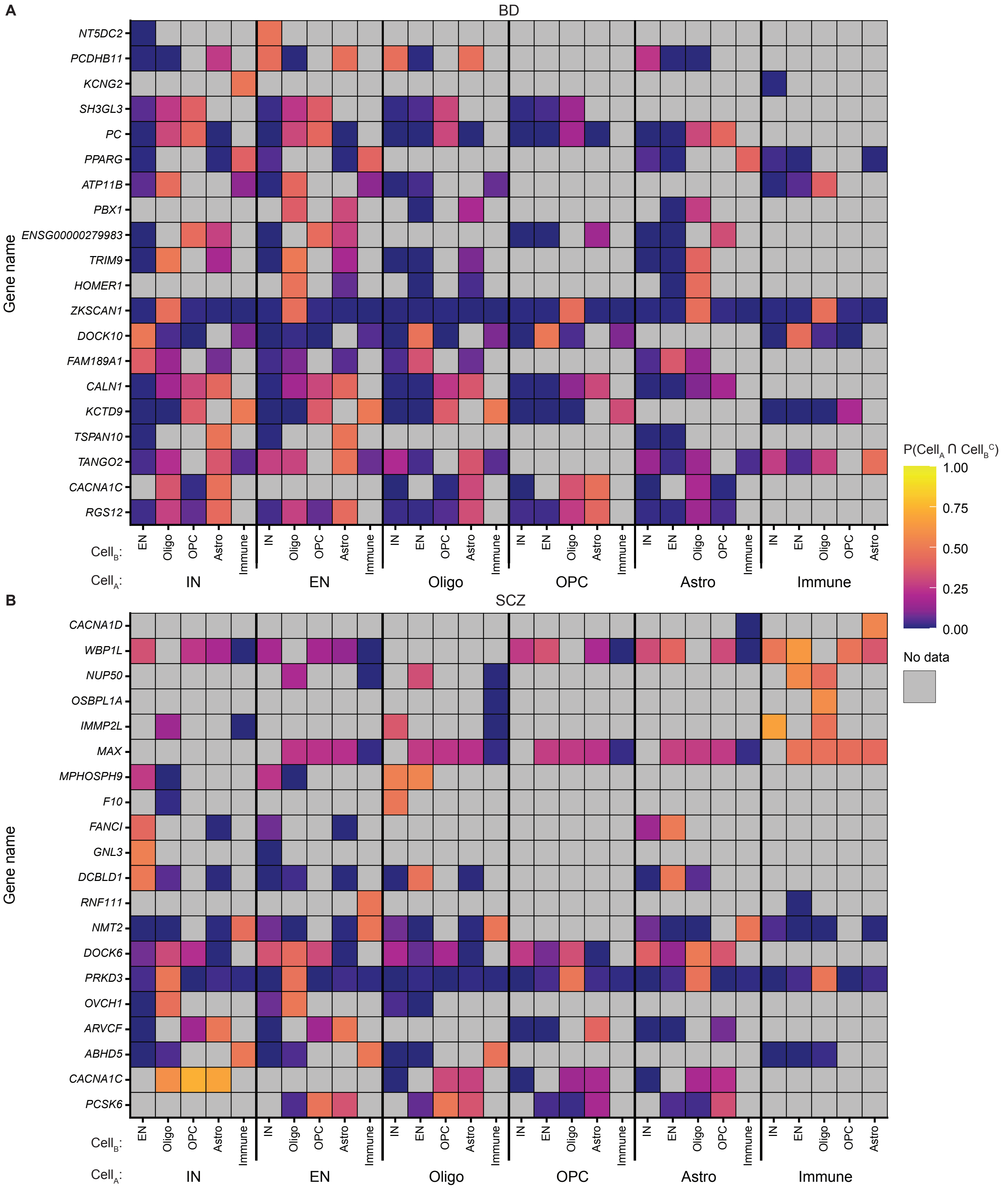
A
BD
B
SCZ
Gene name
NT5DC2
PCDHB11
KCNG2
SH3GL3
PC
PPARG
ATP11B
PBX1
ENSG00000279983
TRIM9
HOMER1
ZKSCAN1
DOCK10
FAM189A1
CALN1
KCTD9
TSPAN10
TANGO2
CACNA1C
RGS12
CACNA1D
WBP1L
NUP50
OSBPL1A
IMMP2L
MAX
MPHOSPH9
F10
FANCI
GNL3
DCBLD1
RNF111
NMT2
DOCK6
PRKD3
OVCH1
ARVCF
ABHD5
CACNA1C
PCSK6
Cell$_B$:
Cell$_A$:
IN
EN
Oligo
OPC
Astro
Immune
P(Cell$_A$ ∩ Cell$_B^C$)
1.00
0.75
0.50
0.25
0.00
No data
