# Supplementary material for: Single-nucleus transcriptome-wide association study of human brain disorders
Source: Nature. 2026 Sep 23;657(8133):1016–26. doi: 10.1038/s41586-026-10836-6 (PMC13600940; doi:10.1038/s41586-026-10836-6)
Supplement: Supplementary file 5 [file 41586_2026_10836_MOESM4_ESM.zip › 2024-09-19723C-s4/FigS8_part2.pdf]

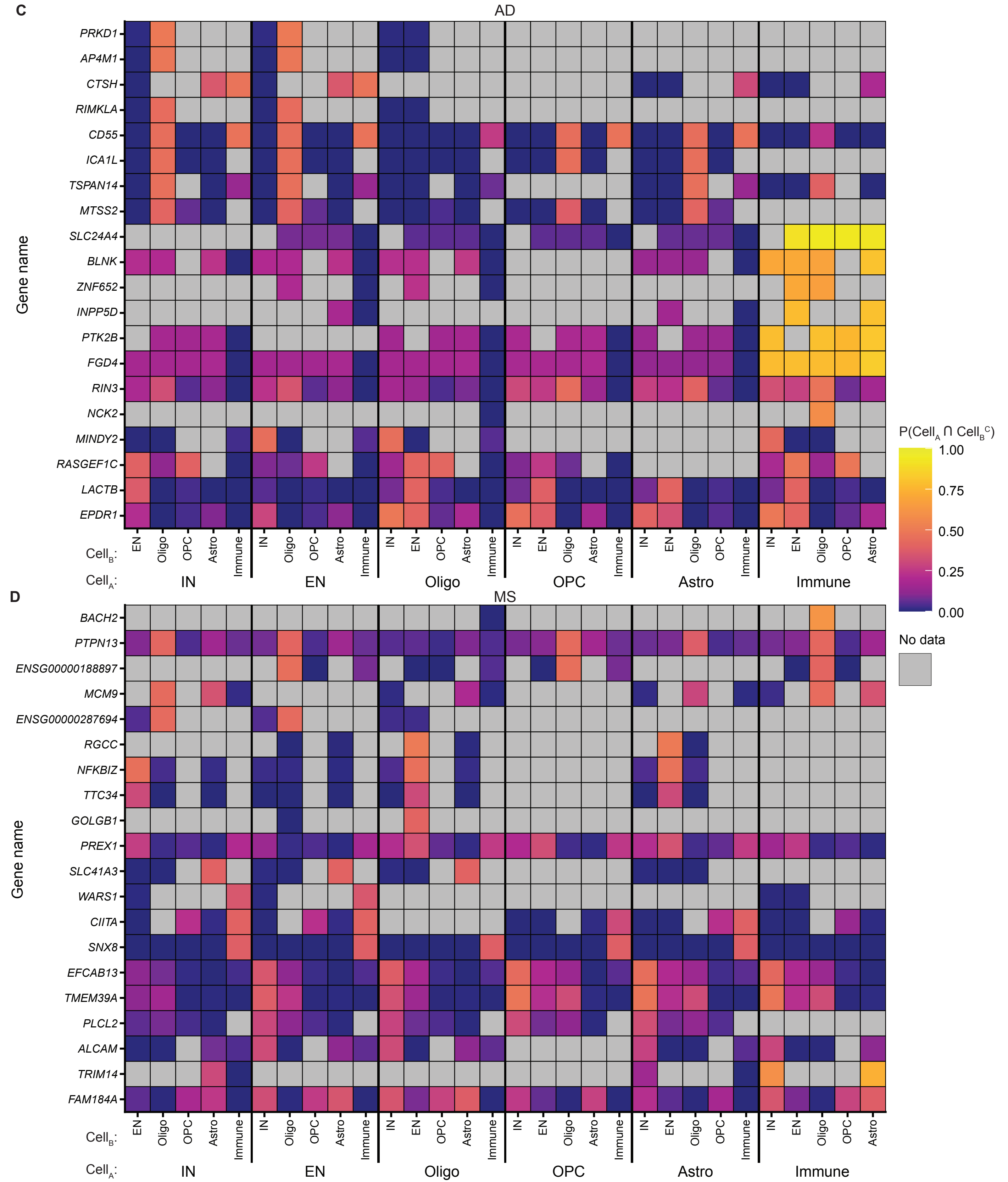
C
AD
PRKD1
AP4M1
CTSH
RIMKLA
CD55
ICA1L
TSPAN14
MTSS2
SLC24A4
BLNK
ZNF652
INPP5D
PTK2B
FGD4
RIN3
NCK2
MINDY2
RASGEF1C
LACTB
EPDR1
Gene name
Cell_B:
Cell_A:
IN
EN
Oligo
OPC
Astro
Immune
P(Cell_A ∩ Cell_B^C)
1.00
0.75
0.50
0.25
0.00
No data
D
MS
BACH2
PTPN13
ENSG00000188897
MCM9
ENSG00000287694
RGCC
NFKBIZ
TTC34
GOLGB1
PREX1
SLC41A3
WARS1
CIITA
SNX8
EFCAB13
TMEM39A
PLCL2
ALCAM
TRIM14
FAM184A
Gene name
Cell_B:
Cell_A:
IN
EN
Oligo
OPC
Astro
Immune
