# Supplementary material for: Single-nucleus transcriptome-wide association study of human brain disorders
Source: Nature. 2026 Sep 23;657(8133):1016–26. doi: 10.1038/s41586-026-10836-6 (PMC13600940; doi:10.1038/s41586-026-10836-6)
Supplement: Supplementary file 5 [file 41586_2026_10836_MOESM4_ESM.zip › 2024-09-19723C-s4/FigS8_part3.pdf]

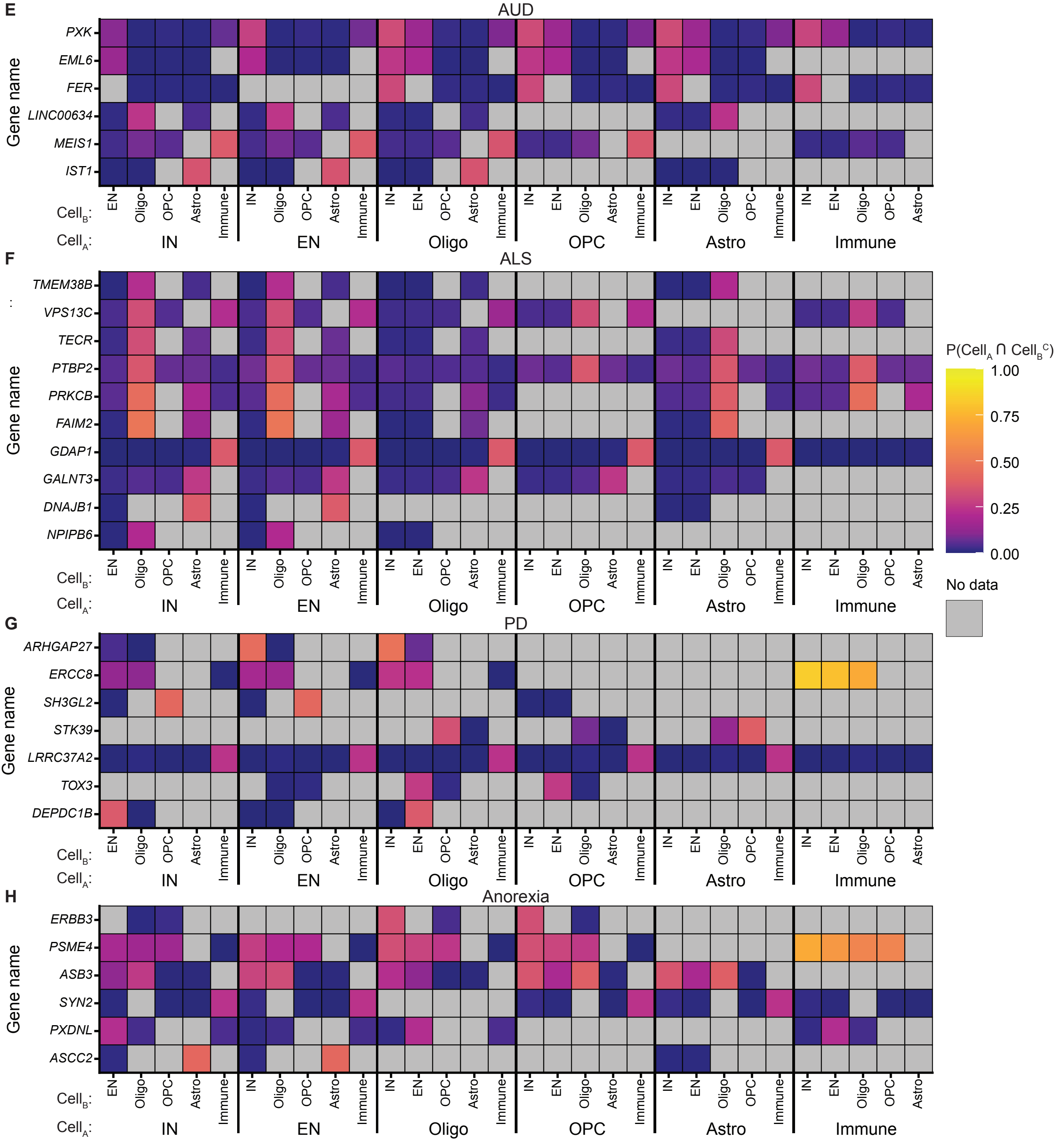
E
AUD
PXK
EML6
FER
LINC00634
MEIS1
IST1
Gene name
Cell_B:
Cell_A:
IN
EN
Oligo
OPC
Astro
Immune
F
ALS
TMEM38B
VPS13C
TECR
PTBP2
PRKCB
FAIM2
GDAP1
GALNT3
DNAJB1
NPIPB6
P(Cell_A ∩ Cell_B^C)
1.00
0.75
0.50
0.25
0.00
No data
G
PD
ARHGAP27
ERCC8
SH3GL2
STK39
LRRC37A2
TOX3
DEPDC1B
H
Anorexia
ERBB3
PSME4
ASB3
SYN2
PXDNL
ASCC2
