# Supplementary material for: Single-nucleus transcriptome-wide association study of human brain disorders
Source: Nature. 2026 Sep 23;657(8133):1016–26. doi: 10.1038/s41586-026-10836-6 (PMC13600940; doi:10.1038/s41586-026-10836-6)
Supplement: Supplementary file 5 [file 41586_2026_10836_MOESM4_ESM.zip › 2024-09-19723C-s4/FigS8_part4.pdf]

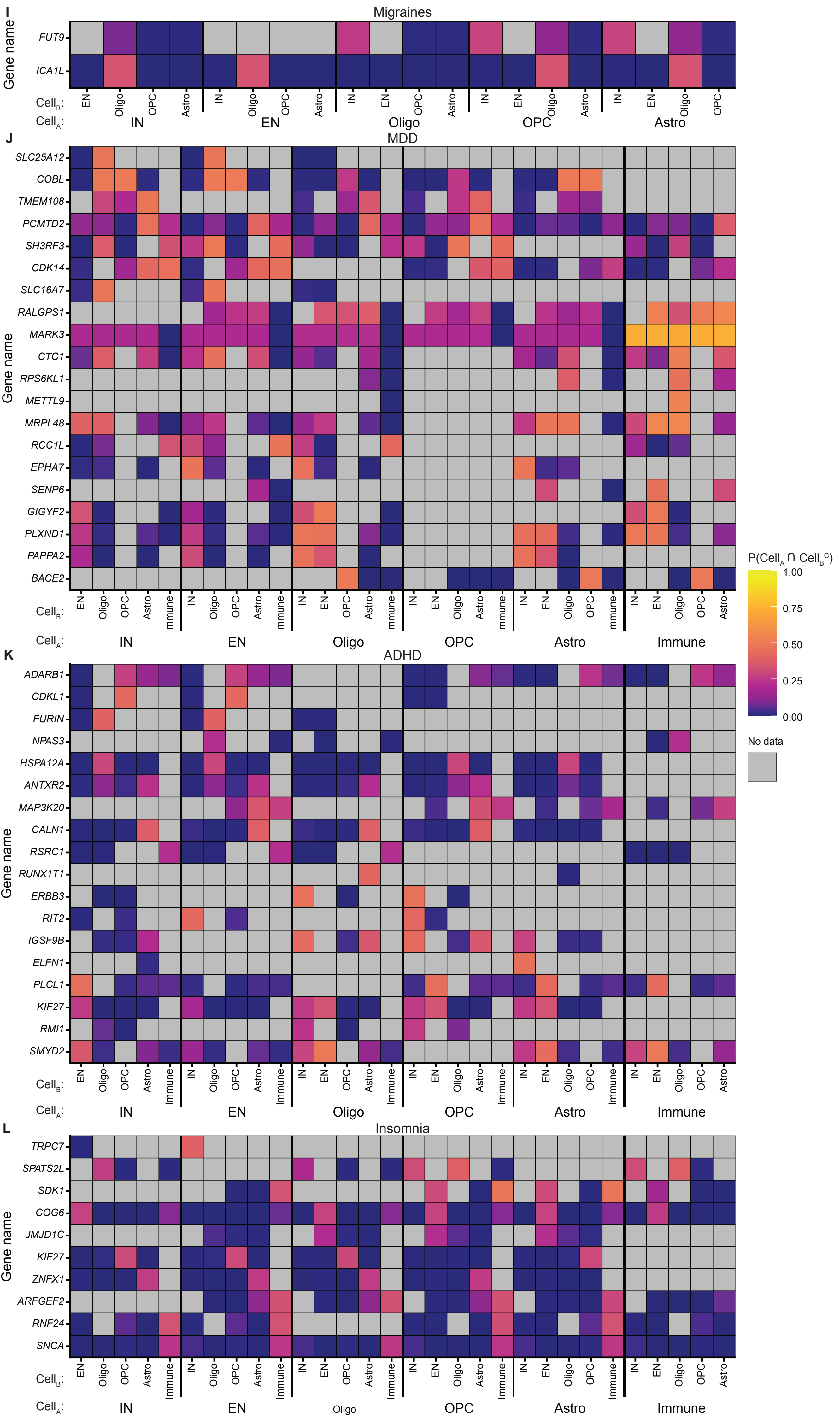
I
Migraines
Gene name
FUT9
ICA1L
Cell$_B$:
EN Oligo OPC Astro
IN Oligo OPC Astro
IN EN OPC Astro
IN EN Oligo Astro
IN EN Oligo OPC
Cell$_A$:
IN EN Oligo OPC Astro
J
MDD
SLC25A12
COBL
TMEM108
PCMTD2
SH3RF3
CDK14
SLC16A7
RALGPS1
MARK3
CTC1
RPS6KL1
METTL9
MRPL48
RCC1L
EPHA7
SENP6
GIGYF2
PLXND1
PAPPA2
BACE2
Cell$_A$:
IN EN Oligo OPC Astro Immune
K
ADHD
ADARB1
CDKL1
FURIN
NPAS3
HSPA12A
ANTXR2
MAP3K20
CALN1
RSRC1
RUNX1T1
ERBB3
RIT2
IGSF9B
ELFN1
PLCL1
KIF27
RMI1
SMYD2
L
Insomnia
TRPC7
SPATS2L
SDK1
COG6
JMJD1C
KIF27
ZNFX1
ARFGEF2
RNF24
SNCA
Immune
$P(Cell_A \cap Cell_B^C)$
1.00
0.75
0.50
0.25
0.00
No data
