# Supplementary material for: Single-nucleus transcriptome-wide association study of human brain disorders
Source: Nature. 2026 Sep 23;657(8133):1016–26. doi: 10.1038/s41586-026-10836-6 (PMC13600940; doi:10.1038/s41586-026-10836-6)
Supplement: Supplementary file 5 [file 41586_2026_10836_MOESM4_ESM.zip › 2024-09-19723C-s4/FigS9.pdf]

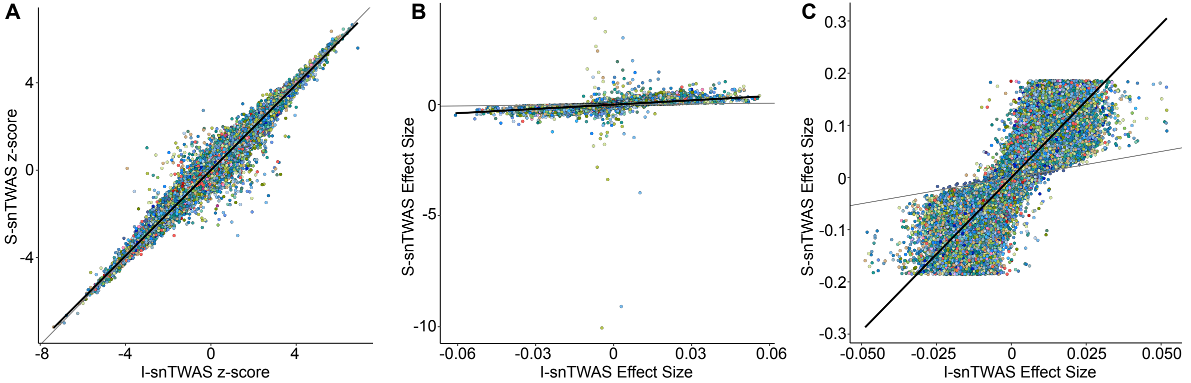

A
S-snTWAS z-score
I-snTWAS z-score
4
0
-4
-8
-4
0
4
B
S-snTWAS Effect Size
I-snTWAS Effect Size
0
-5
-10
-0.06
-0.03
0
0.03
0.06
C
S-snTWAS Effect Size
I-snTWAS Effect Size
0.3
0.2
0.1
0
-0.1
-0.2
-0.3
-0.050
-0.025
0
0.025
0.050
